# Widespread *cis*-regulation of RNA editing in a large mammal

**DOI:** 10.1261/rna.066902.118

**Published:** 2019-03

**Authors:** Thomas J. Lopdell, Victoria Hawkins, Christine Couldrey, Kathryn Tiplady, Stephen R. Davis, Bevin L. Harris, Russell G. Snell, Mathew D. Littlejohn

**Affiliations:** 1 Research and Development, Livestock Improvement Corporation, Hamilton 3296, New Zealand; 2 School of Biological Sciences, University of Auckland, Auckland 1071, New Zealand

**Keywords:** RNA editing, GWAS, milk, QTL mapping, RNA sequencing, genome sequencing

## Abstract

Post-transcriptional RNA editing may regulate transcript expression and diversity in cells, with potential impacts on various aspects of physiology and environmental adaptation. A small number of recent genome-wide studies in *Drosophila*, mouse, and human have shown that RNA editing can be genetically modulated, highlighting loci that quantitatively impact editing of transcripts. The potential gene expression and physiological consequences of these RNA-editing quantitative trait loci (edQTL), however, are almost entirely unknown. Here, we present analyses of RNA editing in a large domestic mammal (*Bos taurus*), where we use whole-genome and high-depth RNA sequencing to discover, characterize, and conduct genetic mapping studies of novel transcript edits. Using a discovery population of nine deeply sequenced cows, we identify 2413 edit sites in the mammary transcriptome, the majority of which are adenosine to inosine edits (98.6%). Most sites are predicted to reside in double-stranded secondary structures (85.1%), and quantification of the rates of editing in an additional 355 cows reveals editing is negatively correlated with gene expression in the majority of cases. Genetic analyses of RNA editing and gene expression highlight 152 *cis*-regulated edQTL, of which 15 appear to cosegregate with expression QTL effects. Trait association analyses in a separate population of 9989 lactating cows also shows 12 of the *cis*-edQTL coincide with at least one cosegregating lactation QTL. Together, these results enhance our understanding of RNA-editing dynamics in mammals, and suggest mechanistic links by which loci may impact phenotype through RNA editing mediated processes.

## INTRODUCTION

The process of gene expression involves transcribing the information stored in DNA into messenger RNA (mRNA). In Eukaryotes, most mRNA sequences differ to those of DNA, primarily due to RNA splicing. However, the process of RNA editing can add additional diversity, whereby bases in the transcript are altered in situ by direct enzymatic modification. In metazoan cells, the most common form of RNA editing is deamination of adenosine (A) nucleotides, forming inosine (I), catalyzed by enzymes from the adenosine deaminase acting on the RNA (*ADAR*) family (Savva et al. 2012).

A key function of mRNA editing by ADAR1 is to prevent immune sensing of endogenous dsRNA by MDA5 (Liddicoat et al. 2015; Heraud-Farlow and Walkley 2016). Depending on the location of edits within the pre-mRNA transcript, other potential consequences of RNA editing can include changes to the coding sequence, the creation or destruction of splice sites (Nishikura 2010), triggering of nuclear retention mechanisms of edited transcripts (Zhang and Carmichael 2001; Prasanth et al. 2005), or the creation or destruction of microRNA (miRNA) binding sites within the 3′-UTR (Liang and Landweber 2007; Wang et al. 2013). These changes in turn can affect gene expression, either as part of normal regulation (Goldstein et al. 2017), in a pathogenic context such as cancer (Zhang et al. 2016; Baysal et al. 2017), or as a mechanism to regulate alternative splicing (Solomon et al. 2013).

Genetic regulation of gene expression, whether operating through polymorphic variation in *cis* or *trans* regulatory elements, or through other mechanisms such as DNA methylation, is thought to account for the majority of genetic variance in phenotypic traits (Nicolae et al. 2010). Identification of expression quantitative trait loci (eQTL), therefore, provides insight into causative mechanisms for colocating QTL for more broadly defined physiological traits, where these methods have been applied to identify causative genes for various characters and diseases in humans (Li et al. 2015; Zhu et al. 2016; Li and Huang 2018), model species (Parks et al. 2013, 2015), and agricultural and domestic species (Li et al. 2013; Littlejohn et al. 2016; Lopdell et al. 2017).

Since numerous regulatory effects have been attributed to RNA editing, genetic regulation of editing poses another potential mechanism to explain impacts on physiological traits. In three recent studies conducted in *Drosophila* (Ramaswami et al. 2015), mice (Gu et al. 2016), and humans (Park et al. 2017), researchers demonstrated the application of QTL mapping approaches to reveal widespread genetic modulation of RNA editing. In the current study, we aimed to build on these studies by characterizing the genetic landscape of RNA editing in cattle, and more specifically, use these data to investigate potential regulatory effects of identified loci on gene expression and complex quantitative traits. Utilizing whole-genome sequencing (WGS), high-depth mammary RNA sequencing, and genome-wide association approaches in outbred cattle populations, we report the de novo discovery of RNA edits, RNA-editing QTL (edQTL), and a number of colocating, cosegregating gene expression and lactation impacts as potential consequences of these modifications.

## RESULTS

### Discovery and molecular context of edited sites

To identify candidate RNA editing sites, we performed WGS of nine animals for which high-depth RNA-seq data was also available. Animals were sequenced at an average 22-fold read depth for genomic sequence and 104 million read pairs for RNA-seq, with variants called for both DNA and RNA sequence alignments (Materials and Methods). Variants that were identified from RNA data but found to be absent from DNA data for the same animal were considered candidate sites (*N* = 9520). After applying further quality filtering to the variants (including visual inspection of alignments, see Materials and Methods), a total of 2413 edited sites were identified. Editing sites in lactating mammary tissue have not been previously reported in bovine studies (or any other species to our knowledge), though of the recent bovine studies examining editing in other tissues, our data set includes 121 of the 1600 sites reported by Bakhtiarizadeh et al. (2018), and 92 of the 671 sites reported by Chen et al. (2016). Between all three studies, a total of 35 sites are shared.

The 2413 edit sites mapped to a total of 649 genes (median 2.0 sites per gene, mean 3.7) with the majority of sites (84.4%) contained in intronic sequences (Table 1). Edits locating to the 3′-UTR were the next most common class (7.3%), with comparatively few edited sites in the 5′-UTR or coding exons. Relatively few sites were predicted to impact protein sequences (21 missense, 21 synonymous). These distributions are in broad agreement with previous reports of the distribution of edits in the human transcriptome (Chen 2013).

**TABLE 1.**
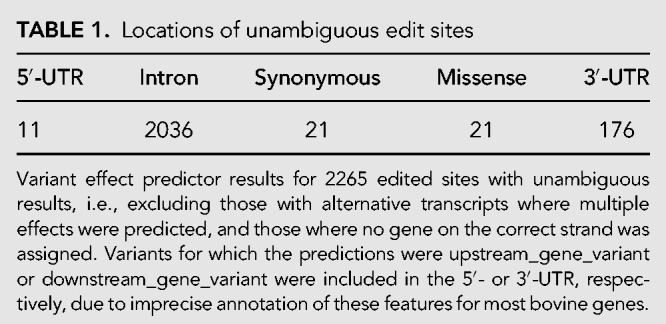
Locations of unambiguous edit sites

**FIGURE 1.**
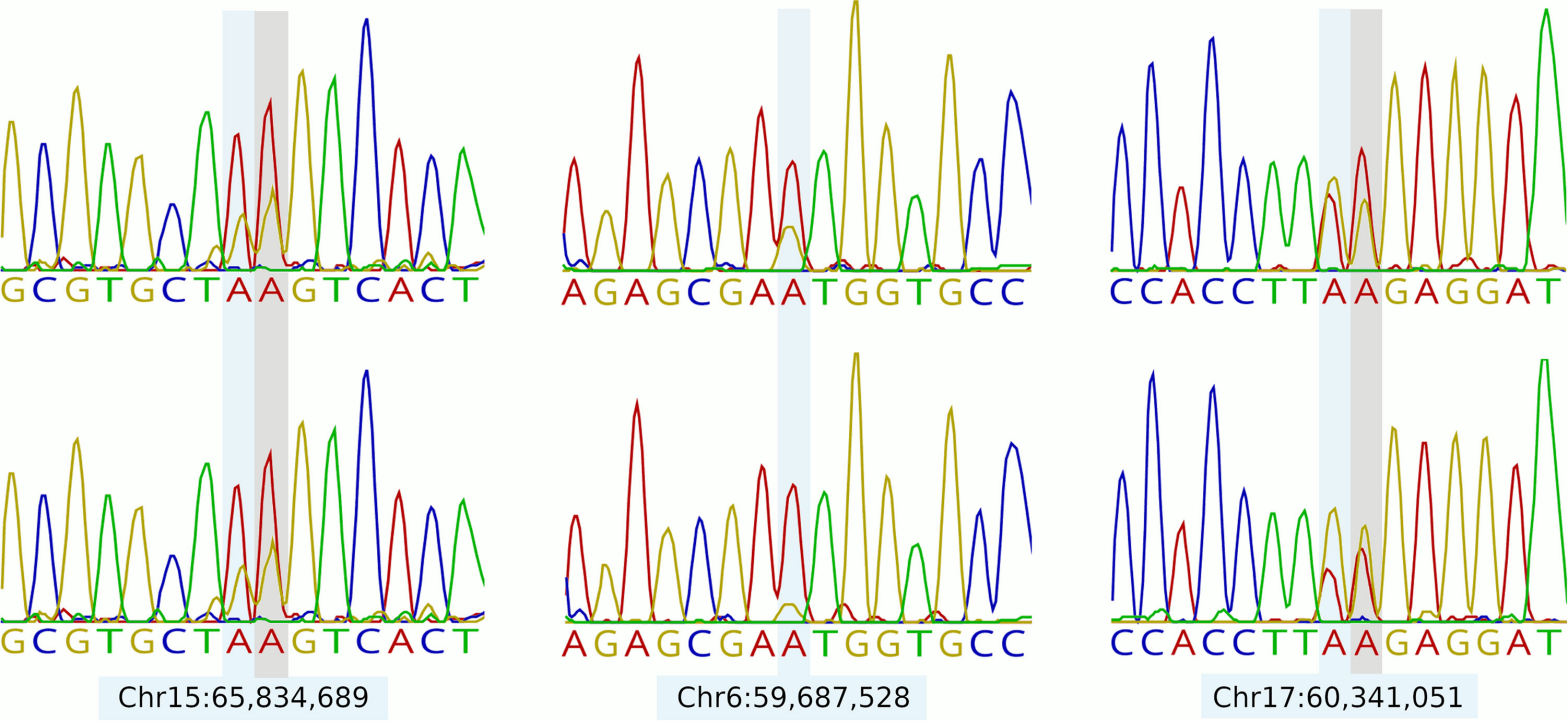
Traces for three RNA editing sites targeted for verification by Sanger sequencing, shown for two of the four animals resequenced. Locations of the targeted sites are indicated in blue. Adjacent edit sites that map within the sequence traces but were not targeted for verification are shown in gray.

To confirm the validity of a subset of the candidate edit sites using an alternative technology platform, eight sites were targeted for Sanger sequencing. Sites were selected for validation based on having satisfied several criteria in accordance with sensitivity expectations of the Sanger method, though were otherwise chosen randomly (see Materials and Methods). Following RT-PCR and sequencing of amplicons representing four of the animals used for RNA-seq-based edit discovery, five of the eight sites yielded high-quality Sanger traces. Figure 1 shows representative chromatograms for several of these sites, with Supplemental Figure S1 showing data for all successfully sequenced sites and animals. Importantly, edits were confirmed in at least three of the four sequenced animals for all five sites. An additional two RNA editing sites were also captured in the validation amplicons, and although not targeted for that purpose, represented sites that had also been detected using our RNA-seq-based pipeline (Fig. 1). These data suggest that, at least for transcripts that satisfied our validation selection criteria, a large proportion of the identified sites are likely to be real.

Of the different classes of base substitutions, A-to-I edits were by far the most common (98.6% of sites; Table 2). Interestingly, however, the A-to-I edit class was much less dominant when only exonic sites are considered (56.1%, similar to the 40.7% reported elsewhere for a much larger sample; Chen 2013). In fact, 18 of the 33 non-A-to-I edited sites identified were exonic, raising the possibility that reads containing these edits arise from the expression of near-duplicate genes or pseudogenes, and have been incorrectly mapped. The most prevalent non-A-to-I edits were U-to-C and G-to-A, concordant with previous literature for both humans (Chen 2013) and cattle (Chen et al. 2016). As A-to-I edits are catalyzed by the ADAR1 and ADAR2 enzymes, we confirmed the expression of the corresponding genes in mammary tissue, where the *ADAR1* gene was approximately 1.5-fold more highly expressed than *ADAR2* (*ADAR1* = 4.1 TPM; *ADAR2* = 2.8 TPM; Table 3). Minimal levels of expression were observed for homologs to human *APOBEC* genes, which have been implicated in non-A-to-I edits.

**TABLE 2.**
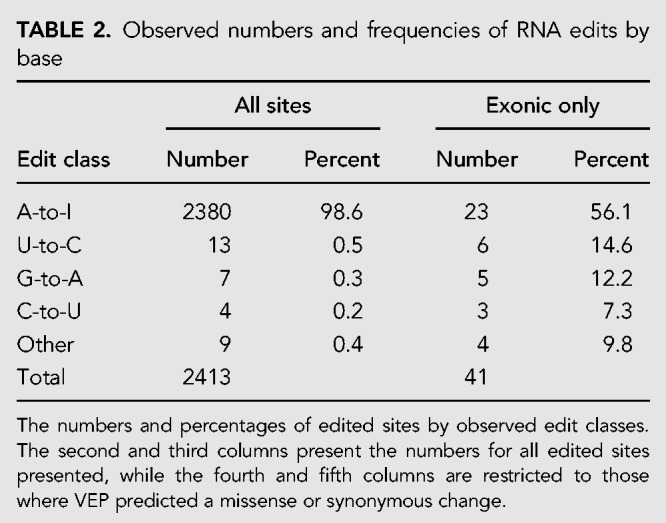
Observed numbers and frequencies of RNA edits by base

**TABLE 3.**
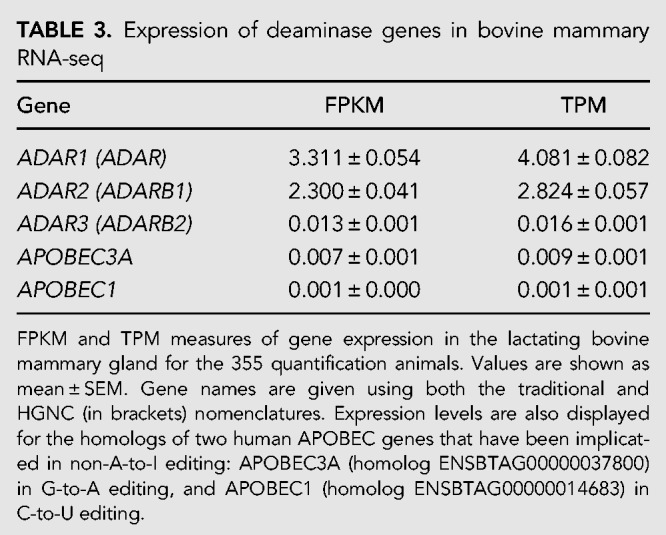
Expression of deaminase genes in bovine mammary RNA-seq

**FIGURE 2.**
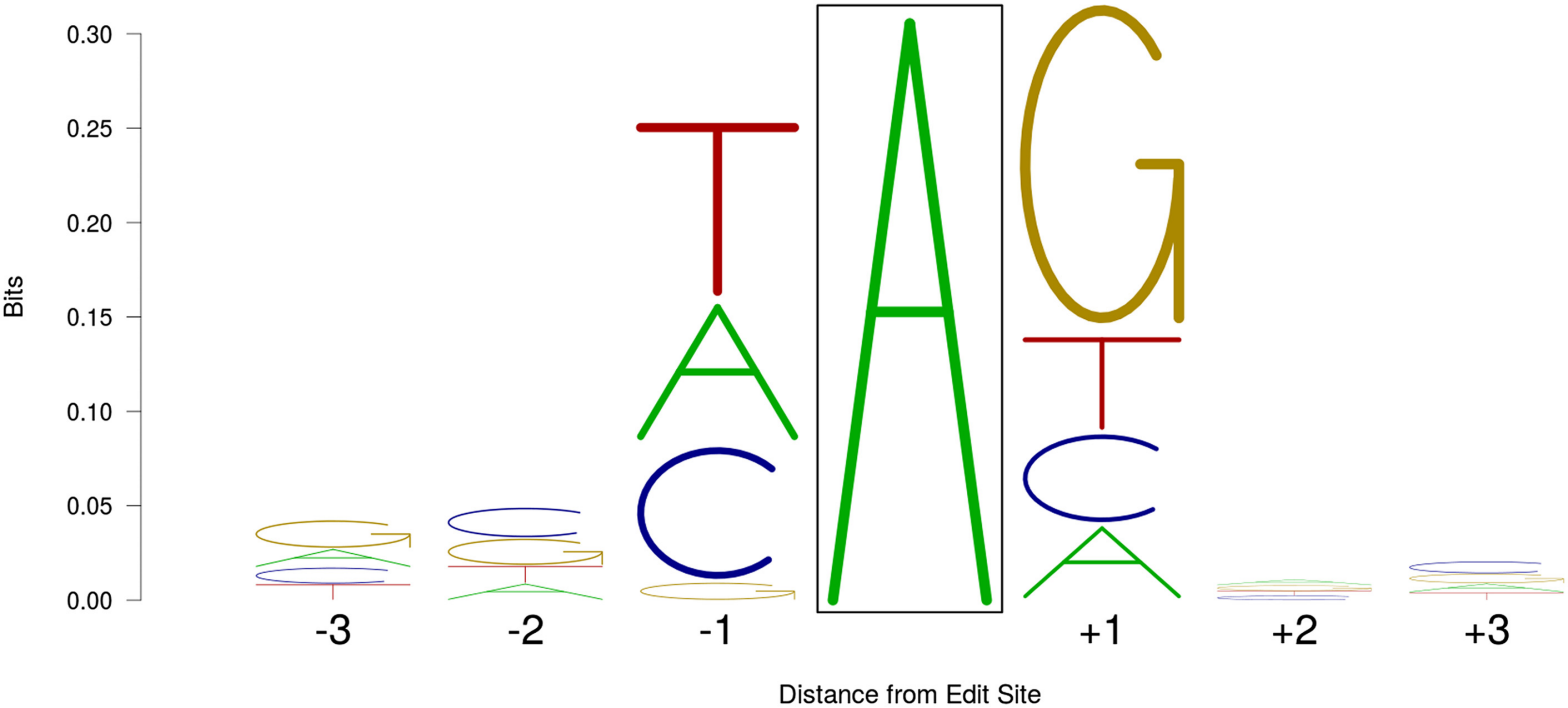
A sequence logo (Schneider and Stephens 1990) showing the edit-containing consensus sequence based on all 2380 A-to-I RNA edit sites identified in the current study. The proportion of each column occupied by each letter represents the frequency of that base at that position, while the total height of each column is equal to the information theoretical entropy (bits) at each position, with calculations as previously described (Schneider et al. 1986). For clarity of presentation, the edit site (boxed) is not shown at its actual height of 1.999 bits.

Nonuniform base usage was seen for bases directly adjacent to RNA editing sites (Table 4). In particular, guanosine was significantly underrepresented at the position immediately upstream of edit sites (3.8% of bases; *P* = 8.8 × 10^−134^), but significantly enriched at positions immediately downstream (56.1% of bases; *P* = 1.1 × 10^−270^). Similar patterns of upstream underrepresentation and downstream enrichment for guanosine have been reported in the literature (Lehmann and Bass 2000; Ramaswami et al. 2013; Bazak et al. 2014; Porath et al. 2014). No motifs were observed at positions more distant than one nucleotide (Fig. 2), an observation reported previously (Peng et al. 2012).

**TABLE 4.**
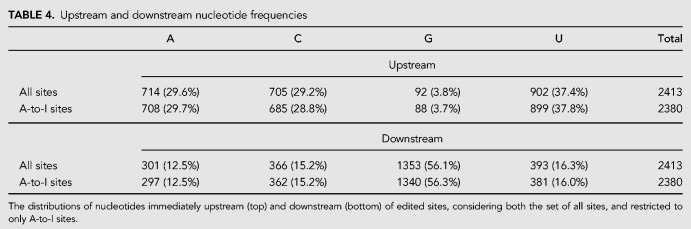
Upstream and downstream nucleotide frequencies

In humans, the majority of RNA editing sites occur within *Alu* short interspersed nuclear elements (SINEs; Athanasiadis et al. 2004). To test whether edit sites were prevalent in SINEs in cattle, the list of RNA edit sites was compared to annotations produced by RepeatMasker. This analysis showed that RNA edit sites were predominantly located within the SINEs Bov-tA2 (43.9%), Bov-tA1 (20.8%), BOV-A2 (9.2%), and Bov-tA3 (6.5%), with 3.1% mapping to BovB LINEs. In total, 2248 of the 2413 edit sites (93.2%) were contained within a repeat element.

### Predicted edit sites occur predominantly in double-stranded regions

Since the ADAR1 and ADAR2 enzymes target double-stranded sections of RNA (Lehmann and Bass 1999, 2000), it is expected that most edited sites will map to sequences able to form double-stranded structures. To test this hypothesis, double-stranded regions of pre-mRNA transcripts were computationally predicted using R (R Core Team 2017) to produce dot-plots (Supplemental Fig. S2). Visual examination of the predicted structures confirmed that the majority of candidate edited sites (85.1%; 2053 of 2413) are located within regions of RNA with the potential to form double-stranded helices. When only A-to-I edits were considered, a slightly higher percentage (86.0%; 2047 of 2380) were predicted to occur in such regions. Although some proportion of the 14.9% of sites not observed to reside in double-stranded regions could be assumed to be false-positive edit sites, these inconsistencies could arise from failure to accurately identify base-paired structures, or where the paired strand was more than 1500 base pairs from any edit sites within the gene and therefore not included within the plotted region (see Materials and Methods for a description of double-stranded prediction methodology). Images of the predicted double-stranded secondary structures are displayed in Supplemental Figure S3.

Within double-stranded regions, 58.4% of A-to-I sites (*n* = 1195) were predicted to base-pair with a uridine residue, in accordance with standard Watson–Crick base-pairing rules (assuming an adenosine reference allele). The majority of the remaining sites were situated opposite to a cytidine residue (*n* = 760; 37.1%), in agreement with previous literature (Wong et al. 2001; Källman et al. 2003). This arrangement would allow wobble base-pairing between the cytidine and inosine nucleotides after editing. The non-A-to-I edit sites were much more sparsely represented within double-stranded regions, with only 18% (6 of 33) of the sites situated within these regions. This suggests that either the non-A-to-I sites are edited by mechanisms which do not require double-stranded RNA, and/or that these sites have a considerably higher false-positive rate than the A-to-I edits.

### Proportions of reads edited

**FIGURE 3.**
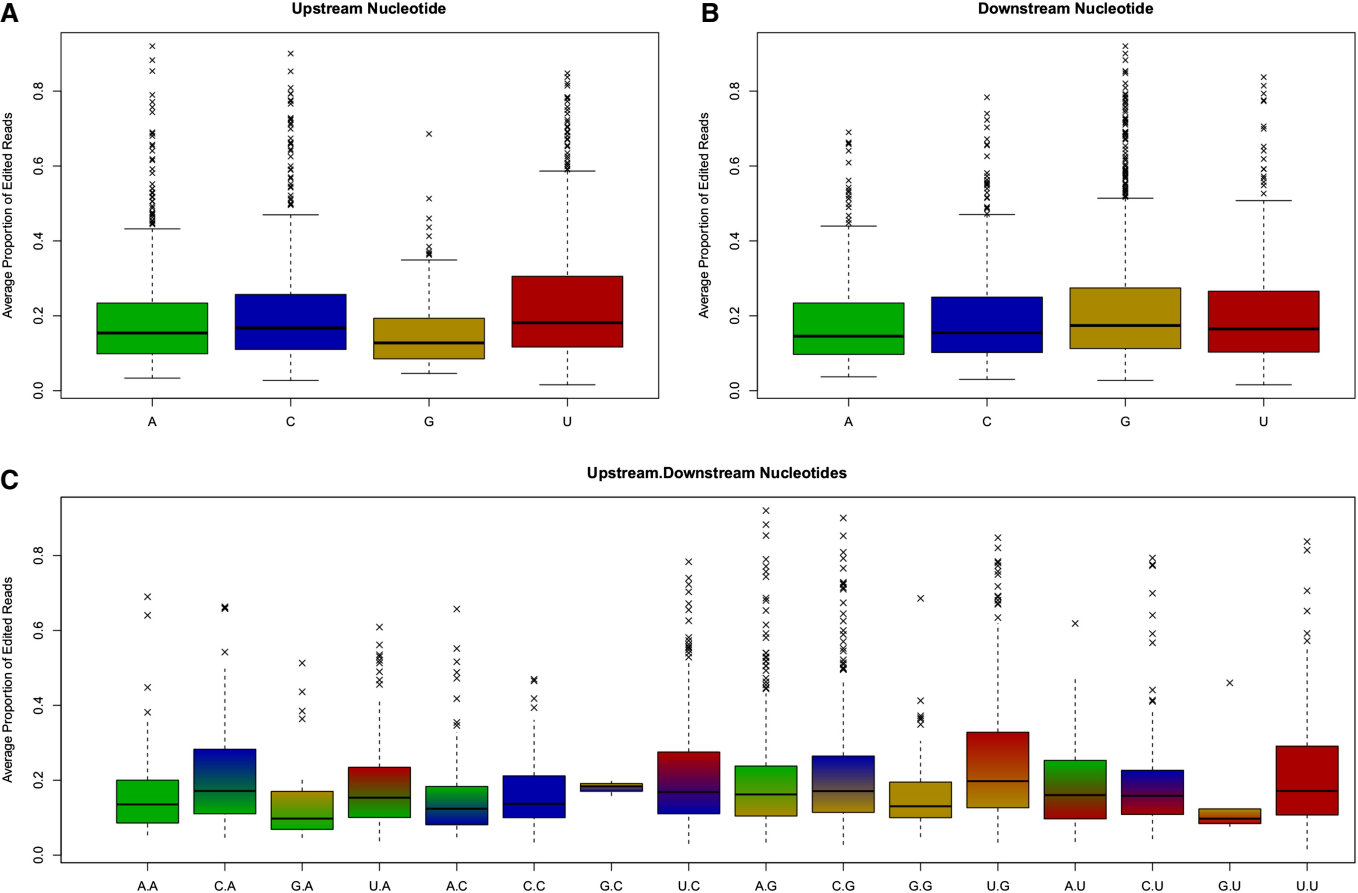
Distributions of the proportion of edited reads (Φ) for A-to-I RNA editing sites, by (*A*) upstream nucleotide; (*B*) downstream nucleotide; (*C*) both upstream and downstream nucleotides. Proportions at each site are averaged across 355 animals in the quantification data set.

To provide a quantitative assessment of editing in a larger population of animals, the base composition of candidate sites identified in the nine “discovery” animals was assessed in 355 additional animals for which RNA-seq data were available. The proportion of reads edited in these “quantification” animals was defined as phi (Φ; Park et al. 2017). Phi values varied widely across sites: from 1.6% to 92.0% for A-to-I reads (median 16.5, mean 21.0). A significant association was observed between the upstream nucleotide at an editing site and the proportion of reads edited (*P* = 4.2 × 10^−9^, see Fig. 3A). This was due primarily to an increase in editing where the upstream nucleotide is uridine (mean = 23.1%), with a decrease observed for upstream guanosine (mean = 16.3%). In contrast, a much less significant association was observed for the downstream adjacent nucleotide (*P* = 0.004, Fig. 3B), with an increase in editing observed for downstream guanosine (mean = 21.8%). Considering both the upstream and downstream bases simultaneously (Fig. 3C), the highest rates of editing were observed at U.G and U.U sites, and the lowest at G.A and A.A sites, where the dot represents the editing site. Mean and median Φ values are displayed in Supplemental Table S1 for all A-to-I edit sites.

Within double-stranded regions, lower values of Φ were observed (median 6.5% of reads edited) where the predicted base pair of the edit site was a guanosine, compared to other bases. These edit sites were also the least frequently observed, with only 24 observations among the 2047 within double-stranded regions. Conversely, the most stable modification is expected when the paired base is a cytidine, and a higher median Φ value was observed for these sites (15.9% of reads). Values for paired adenosine and uridine bases were intermediate, at 15.2% and 11.9% of reads edited, respectively.

### Relationship between RNA editing and transcript abundance

Given the observation of widespread editing across diverse mammary transcripts, we wondered what physiological effects might be attributable to these modifications. Since editing has previously been proposed as a mechanism to modulate gene expression through microRNA-based mechanisms (Liang and Landweber 2007; Wang et al. 2013; Brümmer et al. 2017), through nuclear retention (Zhang and Carmichael 2001; Prasanth et al. 2005), or by preventing activation of the MDA5-MAVS interferon response (Sharpnack et al. 2018), we looked at the relationship between Φ-values and transcript abundance by calculating Pearson correlation coefficients for each implicated gene. For this analysis, we were particularly interested in the impacts on mRNA, so to best represent spliced transcripts, transcript abundance was quantified using reads that either mapped wholly within exons, or mapped across exon–exon boundaries (see Materials and Methods). Strikingly, we noted significant correlations for a large proportion of edited transcripts (*N* = 117 after Bonferroni adjustment; Supplemental Fig. S4), with the distribution of effects showing a strong bias toward genes whose expression was negatively correlated with Φ (Supplemental Fig. S4). To assess the potential role of *ADAR* expression in this relationship, we also repeated this analysis after adjusting for expression of the *ADAR* gene. This analysis had no obvious impacts on the strength of correlation (Supplemental Fig. S5), suggesting that these impacts were unlikely to be modulated by *ADAR* expression per se. Although it is unknown whether editing is driving these effects, these observations highlight a potential mechanism by which RNA editing may be impacting lactation phenotypes through regulation of mRNA abundance.

### Genome-wide association analysis of RNA edits

Having defined Φ values for all animals and all curated sites, we next used these data as phenotypes for genome-wide association studies (GWAS), with the aim of discovering RNA editing QTL. These models comprised generalized least squares (GLS) models, modeling the covariance between animals using a numerator relationship matrix based on pedigree records to account for underlying population structure and relatedness between animals (Materials and Methods). Using 630,774 genotypes from the Illumina BovineHD marker panel and logit-transformed Φ-values as phenotypes, 187 of the 2413 RNA editing sites exhibited edQTL that were significant after Bonferroni adjustment for multiple testing (threshold 0.05/630,774 = 7.93 × 10^−8^). Of these 187 edQTL, 134 sites harbored the top associated variant within 500 kbp of the editing site, and could therefore be assumed to be regulated in *cis*. These sites mapped to a total of 89 genes, with the *CD46*, *CSN3*, *ELF5*, *HOOK3*, and *HPSE* genes each containing five or more associated sites. The full list of 134 sites is detailed in Supplemental Table S2. Low levels of inflation in the test statistics were observed across the 187 edQTL GWAS (mean = 1.09, median = 1.06; ideal value = 1.0), indicating that the GLS models were adequately controlling for relatedness between the animals.

**FIGURE 4.**
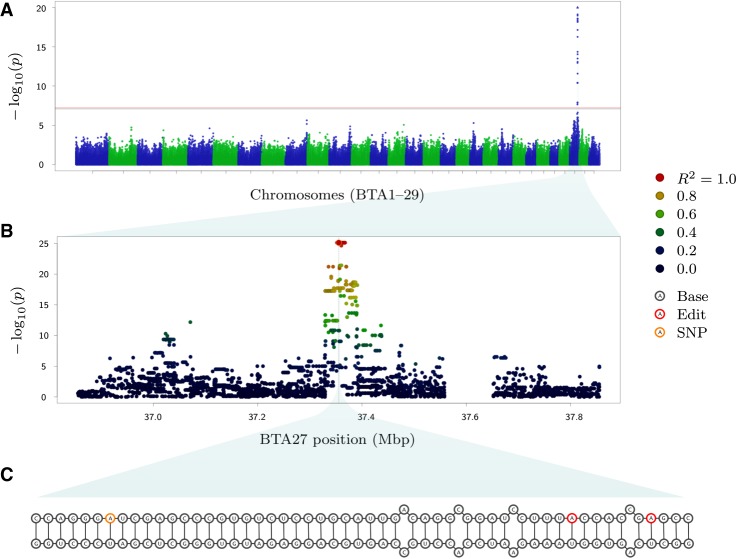
Genetic analysis of RNA editing at the *HOOK3* locus. (*A*) A Manhattan plot showing an edQTL using markers from the BovineHD panel, with the edited site located at BTA27:37,355,505 within *HOOK3*. The *horizontal* black line indicates the genome-wide significance after Bonferroni correction. (*B*) A 1 Mbp window *centered* on the most significant variant in the WGS-resolution data (rs109157662; BTA27:37,355,466). Colors represent the strength of LD (*R*^2^) with that variant. The *vertical* dashed line indicates the position of the edited site. No variants are present around 37.6 Mbp due to the presence of numerous small contigs at this locus in the reference sequence, which have been filtered out of the WGS data set due to errors in phasing. (*C*) Putative structure of the pre-mRNA surrounding the edited sites (red) and candidate causative SNP (orange). Site BTA27:37,355,505 is the *leftmost* edited site. The candidate causative SNP is also shown in *B* with an orange border.

We next aimed to fine-map signals using imputed WGS data. Imputation was conducted using methods analogous to those previously described (Lopdell et al. 2017; Materials and Methods). The 134 sites with *cis*-edQTL were remapped at WGS resolution for 1 Mbp windows in the 355 quantification set animals, with intervals centered on the most significant marker identified on the BovineHD panel for each site. Associations were conducted as per the analysis using the BovineHD panel. Sixty-two of 134 sites had at least one strongly associated WGS marker (exceeding the Bonferroni threshold) that mapped within the double-stranded region containing that site. Figure 4 shows example plots for the *HOOK3* gene.

### Examination of RNA phase and complementarity relationships between edit sites and candidate modulatory variants

Since base substitutions within double-stranded RNA transcripts could be assumed to modify the structure and stability of such molecules, we reasoned that colocated, RNA editing-associated WGS variants would make strong candidate causal variants for the observed edQTL. To investigate these relationships, edit sites that exhibited significant *cis*-edQTL were further analyzed in the following two ways. First, read pair information was used to derive individual transcript haplotypes between edited bases and candidate causative alleles, with consistency of these phase relationships then assessed for heterozygous animals. Although we assumed such relationships would be due to impacts on base complementarity in double-stranded RNA molecules, phase analysis was not restricted to regions predicted to form these structures, since the distance between edited bases and WGS variants was relatively short, and necessarily limited to the read fragment length (median unspliced length = 150 bp). Variant pairs were also filtered to exclude those that had fewer than five reads encompassing both sites. This yielded 70 pairs of edited bases and WGS variants, representing 49 distinct edit sites in 37 genes (where the reduced number of edits compared to pairs reflected sites that were paired with multiple variants). Association analysis revealed strong phase enrichment for the majority of pairs, with 58 of 70 significant at the Bonferroni threshold of *P* < 7.14 × 10^−4^ (see Materials and Methods).

**FIGURE 5.**
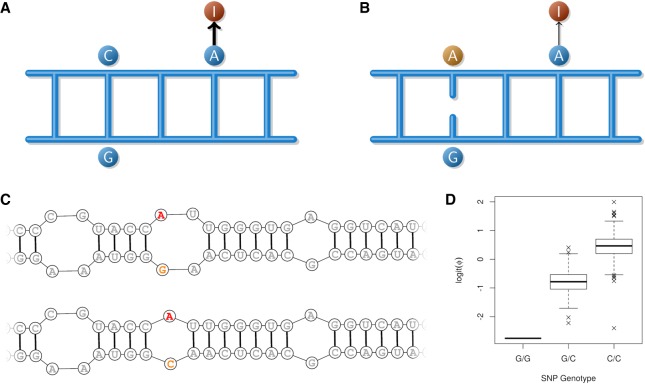
Graphical illustration of the mechanistic hypothesis that reducing sequence complementarity will decrease rates of editing. (*A*) A short double-stranded section of an mRNA secondary structure, containing a SNP (C) with complementary base (G), and an adjacent editing site (A), with high levels of editing. (*B*) The alternative SNP allele (A) reduces complementarity, destabilizing the secondary structure with a consequent reduction in editing rates. Panel *C* shows a site (Chr19.9446387.AG.LPO) in the lactoperoxidase gene exemplifying this scenario. In this case, the edit site (red) is directly opposite the candidate modulatory *G/C* SNP (rs133364759; orange), being highly associated with editing frequency (Φ). The “*G*” allele of this SNP is predicted to reduce the stability of the double-stranded helix adjacent to the edit site, with a predicted altered secondary structure and consequent reduction in the rate of editing (*D*).

The second analysis focused only on pairs of edited bases and edit-associated WGS variants colocating to double-stranded structures (*N* = 144 pairs; where double-stranded regions were predicted as previously described). We hypothesized that WGS alleles that were complementary to the base on the opposite, paired strand would increase the substrate affinity for ADAR enzymes, thus leading to increased editing for these sites (Fig. 5). To test this, we removed all editing-associated variants for which neither allele paired with the opposite base on the complementary strand (*N* = 125 pairs remaining). Using a one-sided *t*-test to assess whether the anticipated sign of effect between edits and complementary and noncomplementary alleles was different than zero, a modest, but significant effect was observed (*P* = 0.012). This observation, and the allele-specific editing results described above, supports the hypothesis that the mechanism of genetic modulation of editing at least partly derives from the impact of these variants on RNA secondary structures.

### Correlations with expression and lactation QTL

RNA editing has previously been reported (Goldstein et al. 2017) to regulate levels of gene expression in *C. elegans*, so we hypothesized that edQTL may also influence expression, where these relationships should manifest as cosegregating edQTL and eQTL. To test this, we first analyzed the 89 genes with *cis*-edQTL for the presence of *cis*-eQTL at WGS resolution. The methods used were analogous to those applied for detection of *cis*-edQTL, with gene expression phenotypes calculated from exonic read counts and normalized using the variance stabilizing transformation (VST) in the DESeq R package (Anders and Huber 2010, see Materials and Methods for further details). This analysis revealed that 41 of the 89 genes had significant *cis*-eQTL with significance defined as having at least one variant with *P* < 1 × 10^−8^. This list included genes with protein products known to be secreted in milk (*CSN3*: κ-casein; *LPO*: lactoperoxidase), along with several genes for which genetic impacts on milk composition or production have previously been published, including *MARC1* (Lopdell et al. 2017) and *PICALM* (Lopdell et al. 2017).

To test for shared regulatory architecture between the eQTL and colocated edQTL, Spearman correlations were calculated using the association *χ*^2^ statistics of each pair of QTL, in an approach similar to that previously described (Littlejohn et al. 2014b, 2016; Lopdell et al. 2017). This method highlights cosegregation patterns for QTL, where, assuming a common causal variant and haplotype structure between signals, the rank-order of associated markers should be similar for effects that are genetically coregulated. Supplemental Table S2 shows results for the 160 edits with *cis*-edQTL, alongside eQTL results and Spearman correlations between QTL pairs. At 15 A-to-I edited sites (Table 5 and examples in Supplemental Fig. S6), correlations of greater than 0.707 were observed between edQTL and eQTL, potentially suggesting a gene expression consequence of the observed edQTL effects. These 15 sites mapped to nine discrete genes.

**TABLE 5.**
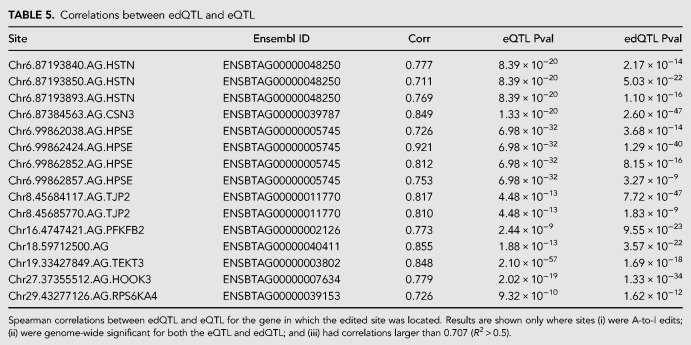
Correlations between edQTL and eQTL

Given the bias toward negatively correlated gene expression and editing per se (see “Relationship between RNA editing and transcript abundance” section above), we also wondered whether *cis*-edQTL/eQTL pairs would reflect this relationship, where we could anticipate allelic effects to show antagonistic signs of effect between edQTL and eQTL. To test this, pairs of eQTL and edQTL that were significantly correlated were identified following Bonferroni adjustment (*N* = 59; *P* < 3.13 × 10^−4^), and subsequently classified as to whether the effect of the top edQTL variant had the same or opposite sign of effect to the eQTL. Given our prior hypothesis that the sign of effects would be reversed, we conducted a one-sided *t*-test that the mean sign of these effects was negative. This yielded a marginally significant *P*-value of 0.0250. This observation suggests that, at least for the loci for which eQTL/edQTL pairs are most strongly correlated (and thus most likely to represent a common genetic signal), increased levels of editing lead to decreases in mRNA expression.

**FIGURE 6.**
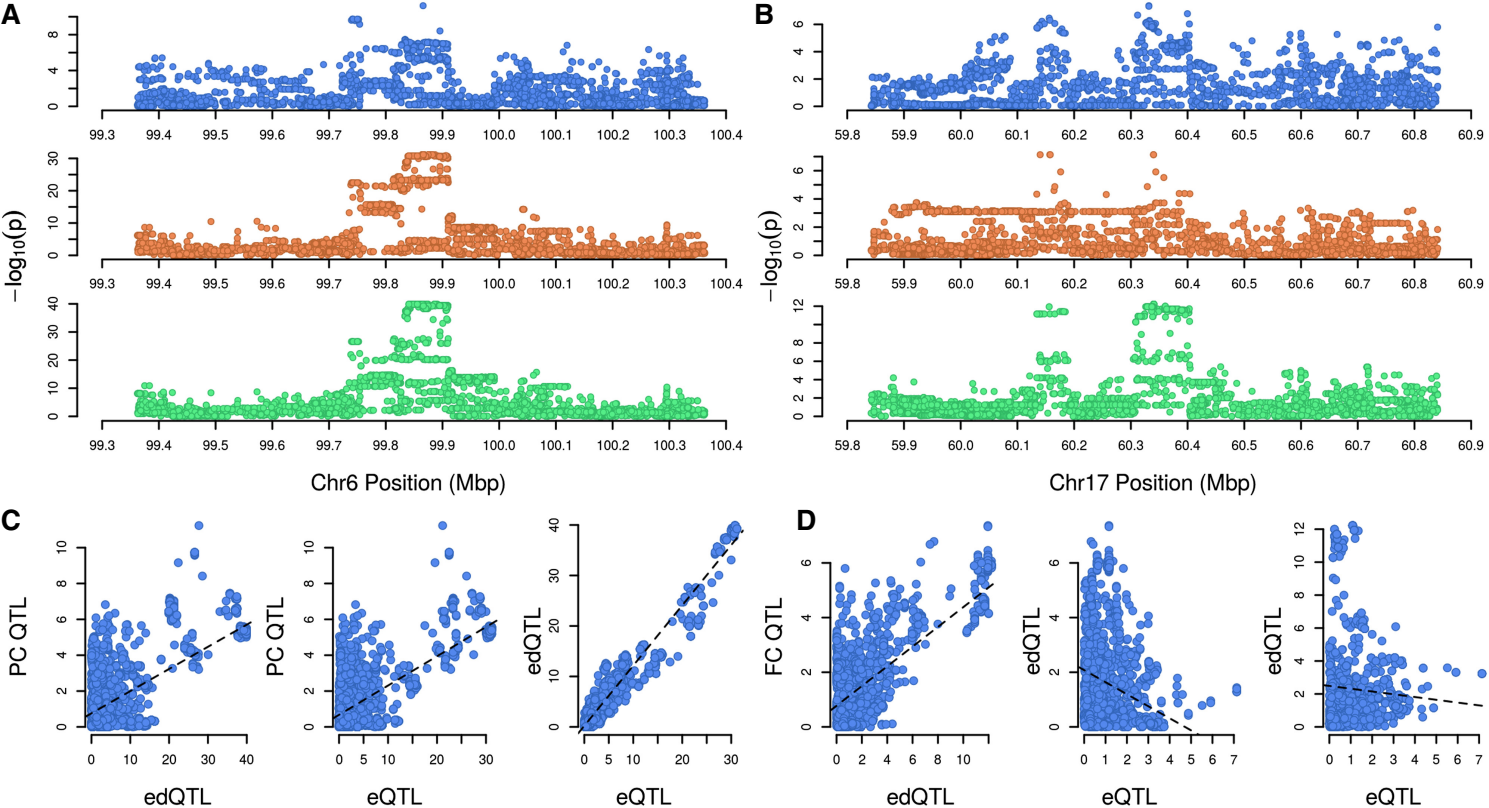
Production QTL and correlations with colocated edQTL and eQTL. (*A* and *B*) 1 Mbp windows for two production QTL (blue), eQTL (orange), and edQTL (green). Panel *A* shows the protein concentration (PC) QTL at Chr6:99.9Mbp, with the cosegregating *HPSE* eQTL and Chr6.99862424.AG.HPSE site edQTL. Panel *B* shows the fat concentration (FC) QTL at Chr17:60.3 Mbp, and cosegregating Chr17.60341051.AG.FBXW8 edQTL, along with an independently segregating *FBXW8* eQTL (panel *D*). *C* and *D*) Plots of QTL *P*-values (−log_10_ scale) against one another. Panel *C* illustrates the positive correlations between all three QTL in panel *A*. Panel *D* shows a case where, while the FC QTL and Chr17.60341051.AG.FBXW8 edQTL are correlated, and therefore may share a similar genetic underpinning, the *FBXW8* eQTL is not correlated with either.

Since edQTL might have further effects on lactation traits such as milk yield and milk component concentration phenotypes, we conducted association analysis on these phenotypes using imputed WGS genotypes and the GLS models described above. To perform association analysis of lactation traits, herd test data for 9989 cows was used to test for the presence of fat, protein, lactose, and milk yield QTL that were colocated to each of the 134 *cis*-edQTL intervals. Examining cosegregation signals using the same methods applied to analysis of eQTL data, 10 edited sites exhibited edQTL that were strongly correlated (*r* > 0.7) with at least one production QTL (Table 6 and examples in Fig. 6). These effects were distributed across five genes, with one site, at Chr6:99862424 in the *HPSE* gene also showing correlations with eQTL (compare Fig. 6C and Fig. 6D). Additional sites with correlations greater than 0.5 were observed in a number of genes (see Supplemental Table S3).

**TABLE 6.**
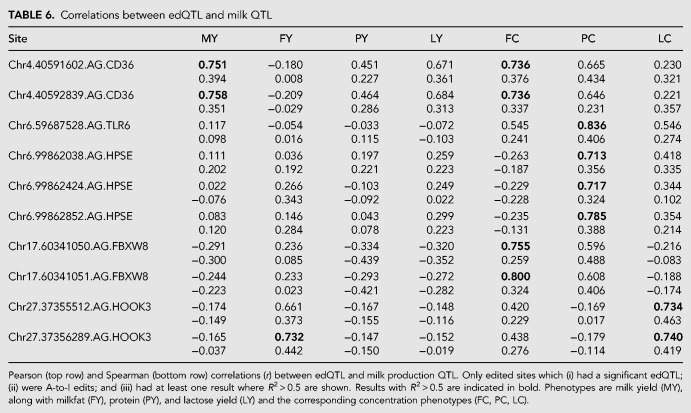
Correlations between edQTL and milk QTL

## DISCUSSION

We report the discovery of 2413 RNA editing sites in the bovine mammary transcriptome, and subsequently explore the genomic context and properties of these sites. We note strong correlations between the extent of RNA editing and the overall abundance of these transcripts, and we further report genome-wide association analyses of editing to identify genetic modulators of these effects. Association analysis of gene expression and lactation phenotypes for variants mapping to edQTL intervals reveals a number of overlapping signals at these locations, providing a potential mechanistic linkage between the editing of transcripts and mammary and lactation physiology. We discuss some of these findings in more detail, below.

### Editing site frequencies

The majority of editing sites discovered were A-to-I edits (2380 of 2413). This percentage (98.6%) is higher than the 80%–88% reported previously for the cattle transcriptome (Chen et al. 2016; Bakhtiarizadeh et al. 2018), but is similar to results reported in human transcriptomes (97.25%, Porath et al. 2014; 93.8%–99.2%, Chen 2013). In the cattle study referenced above (Chen et al. 2016), several tissues were examined, identifying between 180 and 404 edits per tissue. Another recent study in cattle by Porath et al. (2017) identified over 200,000 unique editing sites in bovine brain tissue. The disparity in the number of sites discovered between the Porath et al. (2017) paper, and that reported here, likely derives from the latter using a pipeline specifically designed to identify transcripts with hyper editing (A-to-I edit clusters), and the fact that brain tissue appears to be more highly edited across species (Rosenthal and Seeburg 2012). Studies conducted on human samples have generally reported far higher numbers of edit sites, where the numbers have increased over time due to the growing availability of large data sets: 14,500 in 2004 (Athanasiadis et al. 2004), 22,700 in 2012 (Peng et al. 2012), to over 100 million in 2013 (Bazak et al. 2014). The bulk of these edits occur in *Alu* repeat elements, where almost all adenosines are edited (Bazak et al. 2014). However, because these elements are primate-specific (Bazak et al. 2014), the observation of fewer edits can be anticipated for cattle.

Previous work in mice (Gu et al. 2016) has reported the majority of edited sites falling in UTR regions. Here, in contrast, we found that the majority of sites (2036 of 2413) were intronic. This contrast may be partially attributable to differences between tissues and bovine and murine transcriptomes, though one important distinction between our study and that of Gu et al. (2016) is that we targeted much higher read depths (>200 M reads per sample, versus 10 M). The extreme read depth targeted in this study reflects a strategy to overcome expression biases that are a feature of secretory organs such as the lactating mammary gland (that present a limited diversity of highly expressed transcripts). It is fair to assume, however, that despite these biases, the increased read depth better represents intronic sequences in the current study. The transcriptional status of secretory mammary tissue may also help explain the seemingly low expression values of the ADAR genes. A recent publication in cattle reported TPM values from <5 to >45 across a range of tissues (Bakhtiarizadeh et al. 2018), though given that TPM values are ratios and thus also impacted by the expression levels of other RNA molecules present in the samples (Robinson and Oshlack 2010), highly expressed secretory transcripts could be expected to bias the estimates of other genes downward.

The fact that edited sites, while resembling heterozygous SNPs, will in general not approximate a 50% allelic balance, will also have impacted our ability to discover sites, given that most variant callers assume a diploid state. We chose a variant caller (FreeBayes) that should be less sensitive to allelic imbalances than other commonly applied callers such as HaplotypeCaller (GATK RNA-seq Best Practices), though it can be assumed that the results reported here likely underrepresent sites with low levels of editing.

### Non A-to-I edits

In total, 33 (1.4%) of the 2413 edited sites reported in this study were not canonical A-to-I edits. The three most common noncanonical edit types previously reported for cattle are C-to-U, G-to-A, and U-to-C (Chen et al. 2016), while G-to-A is also reported as the most common in humans (Peng et al. 2012). In the data reported here, the most common noncanonical edit type is U-to-C, with 13 sites of this type. Although edits of this type have been reported in plant chloroplasts and mitochondria (Knie et al. 2016), as well as in transcripts from at least one human gene (Sharma et al. 1994), the most likely case is that these sites represent A-to-I edits from transcripts which have been annotated on the incorrect strand.

Functional edit sites of the second most common type, G-to-A, have been reported for humans in the *hnRNPK* (Klimek-Tomczak et al. 2006), *TPH2* (Grohmann et al. 2010), and *WT1* (Niavarani et al. 2015) genes. These sites are hypothesized to be edited by the APOBEC3A enzyme in humans (Niavarani et al. 2015); however, the homologous cattle gene showed little or no expression in the mammary gland in the current study. Four noncanonical edit sites exhibited C-to-U edits. This type of edit has been attributed in humans to the actions of the APOBEC1 enzyme (Siriwardena et al. 2016). The homologous bovine gene also shows minimal levels of transcription in the current study. Previous work has reported an over-representation of A and U nucleotides in the immediate vicinity of C-to-U edit sites in mice (Rosenberg et al. 2011), however, this was not observed for the sites detected in this study. Considering the minimal expression levels of APOBEC1, these observations suggest this class of edits may be enriched for false-positive sites, and interpretation of these results should be considered accordingly in our analysis.

### Relationships between editing, double-strandedness, and transcript abundance

The majority of edited sites were located within regions for which double-stranded secondary structures were predicted. Of these sites, 58.4% were adenosine nucleotides base-paired to uridine nucleotides, in accordance with Watson–Crick base-pairing rules. When these sites are edited, the stability of the resulting structure is likely to be reduced, though it is noteworthy that I-U base pairs are valid under wobble base-pairing rules (Murphy and Ramakrishnan 2004). Conversely, 37.1% of sites were predicted A-C pairs, which we expect to be unstable until edited into the I-C wobble base pair. This observation is consistent with the work of Wong et al. (2001) and Martin et al. (1985), who showed that I:C edits are more stable than I:A or I:G, and with the work of Levanon et al. (2004), who reported that A:C mismatches are preferred over A:A or A:G mismatches at RNA editing sites in humans. Therefore, we hypothesize that RNA editing is contributing to modulation of the stability of folded pre-mRNA secondary structures. Lower editing frequencies (Φ) were observed in double-stranded regions where the predicted base pair of the edit site was a guanosine. This observation can be explained by wobble base-pairing (Murphy and Ramakrishnan 2004), as guanosine is the only standard RNA base which does not pair with inosine, resulting in lower stability in the double-stranded region after editing at these sites compared to sites paired with other nucleotides. Another explanation, proposed by Wong et al. (2001), is that steric interference by the larger purine bases in A:A and A:G mismatches interfere with the function of the ADAR enzymes, reducing editing efficiency.

We also noted that for many genes, the proportion of editing was significantly correlated with transcript abundance. These correlations were largely negative, where increased editing was associated with decreased mRNA expression. A biological cause and effect relationship is difficult to establish here, given that RNA editing may be a consequence (as opposed to cause) of reduced mRNA production, and other biases relating to library preparation or sequencing (Harcourt et al. 2017) could conceivably lead to spurious, negative correlation. However, these findings are similar to those reported in other analyses of global RNA editing profiles (Hwang et al. 2016). A broadly negative relationship between editing and transcript abundance also fits with a mechanism by which mRNA expression is controlled through preferential retention of edited transcripts in the nucleus (Zhang and Carmichael 2001; Prasanth et al. 2005), or a mechanism whereby mature mRNA abundance is reduced due to altered splicing mediated by RNA editing (Fei et al. 2016). We note, however, that these results are different to those reported recently by Sharpnack et al. (2018), who observed a positive association between RNA editing and gene expression, acknowledging that this was in the context of lung adenocarcinoma.

### RNA edited genes and QTL

A number of the editing sites we detected mapped to genes that are involved in lactation. These genes include the major milk protein components (caseins) encoded by the *CSN1S1*, *CSN2*, and *CSN3* genes, as well as the antimicrobial LPO and LTF proteins. A number of other edited genes are important mediators of milk fat synthesis (*LPL*, *ACACA*, *GPAM* [Bionaz and Loor 2008]) and secretion (*XDH*, *PLIN3* [Bionaz and Loor 2008]), or are involved in the transport of small molecules in milk: *SLC37A1* (Pan et al. 2011), *ABCG2* (Otero et al. 2016). Together, these genes represent some of the most prominent and well-published genes in lactation biology, and include many of the largest effect loci implicated in genetic regulation of these traits (Olsen et al. 2007; Bionaz and Loor 2008; Khatib et al. 2008; He et al. 2011; Kemper et al. 2016). This suggests, at a minimum, that RNA editing may functionally moderate aspects of mammary and lactation physiology, and further presents RNA editing as one of the mechanisms that may underpin milk and lactation QTL.

To investigate the latter hypothesis, GWAS was conducted for all 2413 edited sites, treating the RNA editing proportion (Φ) as a phenotype. This analysis yielded significant *cis*-edQTL at 134 sites. Further analysis of these edQTL suggested that highly associated variants tend to be in phase with the corresponding edit sites, implying a consistency of phase within individual pre-mRNA molecules. We also found evidence that, when edit sites are predicted to be located within the same double-stranded secondary structure as a significant variant, alleles which increase the stability of the structure tend to increase editing, and alleles which destabilize the structure tend to decrease editing. These results are concordant with a mechanism whereby *cis*-edQTL causal variants act within each pre-mRNA transcript to stabilize or destabilize secondary structures, potentially modifying the substrate affinity of these molecules to RNA editing enzymes. These findings broadly support an analysis of the genetic impacts of RNA editing in humans, where the authors similarly looked at aspects of allele-specific editing (Park et al. 2017). This human study (Park et al. 2017), and two other studies in mouse (Gu et al. 2016) and *Drosophila* (Ramaswami et al. 2015), similarly propose allelic effects on folded RNA structures as the likely mechanism driving edQTL.

To look for potential impacts of edQTL on gene expression and lactation phenotypes, we conducted association mapping using intervals of WGS-resolution variants highlighted from RNA editing analyses. Of the 89 genes highlighted with *cis*-edQTL, we identified 41 with significant *cis*-eQTL, 15 of which also showed strong correlation of association statistics (*r* > 0.707). We also assessed correlations between edQTL and colocated lactation QTL, determined in a separate population of 9989 outbred cows. Correlations greater than 0.707 were observed for A-to-I sites in the *CD36*, *FBXW8*, *HOOK3*, *HPSE*, and *TLR6* genes. Two sites in the *CD36* gene were correlated with colocated fat concentration and milk yield QTL, with lower correlations also observed for the lactose yield and protein concentration phenotypes. This gene encodes a component of the milk fat globule membrane, comprising a glycoprotein that is implicated in fatty acid transport (Ibrahimi and Abumrad 2002), and in mammary gland cell proliferation and involution (Spitsberg et al. 1995). The *HOOK3* gene contained three sites with edQTL that were strongly correlated with QTL for lactose concentration or fat yield. *HOOK3* encodes a microtubule tethering protein involved in intracellular vesicle trafficking (Xu et al. 2008), and is broadly analogous in function to the gene *PICALM* that has previously been associated with lactose concentration in milk (Lopdell et al. 2017). It should be noted, however, that *HOOK3* maps near the *GPAT4* gene, which is itself an excellent candidate gene, and for which associations with these phenotypes have been reported previously (Littlejohn et al. 2014b). Additional work would therefore be required to differentiate between potential linkage disequilibrium effects from *GPAT4*, and a genuinely discrete signal driven through a possible *HOOK3* edQTL.

Of the five genes with strong correlations with lactation QTL, *CD36*, and *FBXW8* had relatively modest edQTL/eQTL correlations, whereas *HPSE* and *HOOK3* showed correspondingly strong correlations with eQTL. For these latter genes, our findings present a potential chain of causality from variants modulating the editing of pre-mRNA transcripts, to consequent mRNA expression and lactation effects. Although the mechanism linking mRNA expression to physiological impacts is straightforward, the understanding of the impacts of RNA editing on gene expression is comparatively poor, though can be expected to advance in accordance with the rapidly growing body of literature regarding RNA editing biology. Together, these results improve our understanding of RNA editing in mammals, and our understanding of the link between genotypes and phenotypes in lactation.

## MATERIALS AND METHODS

### DNA and RNA sequencing

Potential RNA editing sites were detected by comparing variant calls from mammary RNA-seq to whole-genome DNA sequence calls. A total of 364 cows of mixed Holstein-Friesian, Jersey, and cross-bred ancestry were divided into two nonoverlapping sets. A discovery set composed of nine F2 Holstein-Friesian/Jersey cross-bred animals was sequenced using both RNA-seq and whole-genome approaches, to enable discovery of edited sites within the RNA. The second set of 355 animals (the quantification set) was sequenced using RNA-seq only, and used to quantify the level of editing at the sites first identified in the discovery set. The quantification animals were used to generate editing proportion phenotypes (Φ) for use in edQTL mapping.

RNA sequencing was performed on mammary biopsies from all 364 animals, as reported previously (Littlejohn et al. 2016). Briefly, high-depth mammary RNA-seq was conducted on tissue obtained via mammary biopsy, sampled at several points in time. Following library preparation, samples were sequenced using the Illumina HiSeq 2000 instrument to produce 100 bp paired-end reads. RNA-seq reads were mapped to the UMD 3.1 reference genome using TopHat2 (version 2.0.12; Kim et al. 2013), mapping an average of 207.9 million reads per sample. Duplicate reads were marked using the MarkDuplicates command in the Picard software package (version 1.89; Broad Institute).

WGS was performed for the animals in the discovery set using methods we have described previously (Littlejohn et al. 2014b, 2016). All animals were sequenced using 100 bp paired-end reads on the Illumina HiSeq 2000 instrument, followed by mapping to the UMD 3.1 bovine reference, using BWA MEM 0.7.8 (Li and Durbin 2009). This yielded mean and median mapped read depths of 22.1× and 22.2×, respectively.

### Identifying edited sites in the RNA

Variant calling was performed on the nine discovery set animals, for both DNA and RNA alignments, using Freebayes version 1.2.0 (Garrison and Marth 2012). Reads that had been marked as duplicates, or with mapping quality scores below 20, were excluded. Variant calling on RNA alignments was performed using the Freebayes options -C 3 -F 0.1 -m 20 -q 25 –min-coverage 5 -U 8, and on DNA alignments with less stringent options -C 2 -F 0.2 -m 10 –q 20 –min-coverage 2 -U 8. Additional filters were subsequently applied to the RNA-seq variant calls, excluding variants with quality scores less than 30, variants with missing genotypes in more than a single animal, and any variants with five or fewer observed alternative alleles. Following the recommendations of Ramaswami et al. (2012), variants were removed from the RNA-seq call list where these were located adjacent to RNA splice sites or homopolymer sequences (≥5 bp), or which were observed only within the first or last six bases of the reads, as well as any variants mapping to regions identified as simple repeats or low-complexity regions using RepeatMasker (version 4.0.5) on the UCSC BosTau8 bovine genome (UMD 3.1.1) with RepBase library release 20140131. Due to the difficulty in accurately calling indel variants, these were excluded from both the RNA and DNA variant sets.

Variants present in the RNA-called set but absent from the DNA-called set formed the initial set of potential RNA edits, yielding a set of 9520 candidate RNA editing sites. These sites were further subjected to manual evaluation to remove sites where, for example, no read coverage was available in the DNA sequence, or where alignments in the RNA sequence appeared anomalous. This resulted in a conservative subset of 4522 sites. Bases were counted at each site in the discovery set animals, and sites were excluded where fewer than two thirds of the animals had an edited read, or fewer than two thirds of animals had fewer than five reads mapping to the site. Using these criteria yielded a final set of 2413 manually appraised sites. An example region comparing DNA and RNA sequencing data for three animals is illustrated in Supplemental Figure S7.

Editing proportions for each of the 2413 verified sites were calculated for each cow in the quantification set by reporting the base composition of reads in the RNA alignments. Edit sites were allocated to genes using the Ensembl Variant Effect Predictor (McLaren et al. 2016), requiring genes to map on the correct strand. Because UTR regions in the released annotations often appeared to be considerably shorter than those evident in the RNA sequence data, variants labeled as upstream or downstream were considered to sit in 5′- and 3′-UTRs, respectively, given that they were discovered in RNA (i.e., expressed) data.

### Predicting two-dimensional mRNA structure

Local mRNA secondary folding structure was predicted for each editing site. Within each gene, sequence was extracted for an interval that included 1.5 kbp of sequence upstream and downstream from the most 5′ and 3′ edited sites. In cases where the total sequence extracted for a gene exceeded 15 kbp, multiple, shorter sub-sequences were used.

Each sequence was then plotted against its complement to generate dot-plots (Supplemental Fig. S2). Dots were placed where at least 11 of the 15 nucleotides, centered on each pair of positions, were complementary. Diagonal lines appearing in the plots are indicative of long strands of complementary sequence, highlighting potential double-stranded regions for examination, by manual observation, for the presence of edited sites. These regions were also processed using the bifold-smp program from the RNAstructure software package (Reuter and Mathews 2010) to generate candidate secondary folded structures.

### Genotyping and RNA QTL discovery

To enable the discovery of edQTL via GWAS, all 355 animals in the quantification data set were genotyped using the BovineHD SNP-chip (Illumina). Variants with minor allele frequencies <1% were removed. As a filter for erroneous SNP assays, tests were conducted for Hardy–Weinberg equilibrium (Graffelman and Moreno 2013) using PLINK software (Purcell et al. 2007; Chang et al. 2015) (version 1.9b3i), with variants yielding *P*-values less than 1.0 × 10^−30^ excluded. The final variant set, containing 630,774 variants, was used for edQTL and eQTL.

As described above, the base composition of RNA editing sites was determined in the quantification set of animals to determine the proportions of edited reads for each animal and site (Φ; Park et al. 2017). To satisfy the normality requirement for phenotypes used in the GLS model, the proportions of edited reads were transformed using the logit function. For each edited site, logit-transformed Φ values (y) were fitted to a GLS model to identify edQTL. The numerator relationship (A) matrix was used to account for any covariances between animals that were due to shared ancestry.

Each genotyped variant was fitted individually using the GLS model described in Lopdell et al. (2017). Briefly, the model used was **y** = **X***β* + *ɛ*, where the error term (*ɛ*) has the conditional mean E (*ɛ*|**X**) = 0 and covariance Var (*ɛ*|**X**) = **W**, where $W=\sigma_{P}^{2}\times (0.3\cdot A+0.7\cdot I$), **I** is the identity matrix and $\sigma_{P}^{2}$ is the phenotypic variance.

To confirm that shared ancestry was not inflating the GLS results, the statistic $\chi^{2}={\left(\frac{\hat{\beta }}{\mathrm{se}(\hat{\beta })}\right)}^{2}$ was calculated for each variant using the estimate of the slope $\left(\hat{\beta }\right)$. For each edited site, the median of the *χ*^2^ statistic was calculated, with the ratio of the observed median and the expected median (0.4549) yielding the inflation statistic for that edited site. Inflation is indicated when the value of this statistic exceeds unity.

As part of a previous study (Littlejohn et al. 2016), gene expression phenotypes were derived for a larger set of 375 animals, of which the 355 animals in the quantification set formed a subset. Gene expression, measured in fragments per kilobase of transcript per million mapped reads (FPKM), and in transcripts per million (TPM) (Wagner et al. 2012), was quantified for genes containing RNA editing sites using Stringtie software (version 1.2.4) (Pertea et al. 2015), with annotations from Ensembl genebuild release 81. Additional gene expression phenotypes were also derived by applying the “VST” function from DESeq (Anders and Huber 2010) to read counts for each gene, resulting in phenotypes with a distribution closer to the normal distribution, and therefore more suitable for analysis with linear models. The read counts used here consisted of only those reads that either (i) mapped entirely within a single exon; or (ii) spliced across one or more annotated exon junctions, according to the exon boundaries defined by the Ensembl annotations (release 81). Reads that spliced at a site not recorded in the Ensembl annotations were excluded.

### WGS imputation and fine mapping

To map variants at a higher resolution around identified edQTL, WGS variants were imputed into the quantification animal set using a previously described reference population of 565 animals (Littlejohn et al. 2014a, 2016), comprising Holstein-Friesians, Jerseys, and cross-bred cattle. Briefly, these cattle were sequenced using the Illumina HiSeq 2000 instrument, yielding 100 bp paired-end reads. Mapping was conducted using BWA MEM 0.7.8 (Li and Durbin 2009), resulting in mean and median mapped read depths of 15× and 8×, respectively. Variant calling was conducted using GATK HaplotypeCaller (version 3.2) (DePristo et al. 2011) with base quality score recalibration, followed by phasing using Beagle 4 (Browning and Browning 2009). Variants with phasing allelic *R*^2^< 0.95 were excluded for quality filtering purposes.

1 Mbp intervals, centered on the top *cis*-edQTL markers, were imputed to WGS resolution using Beagle 4 (Browning and Browning 2009) with the reference population described above, excluding variants with minor allele frequencies below 0.05. Across all 131 intervals, this process resulted in a total of 659,199 variants (mean 5032; min. 2102; max. 10,870 per interval). Although we have no truth set with which to directly determine the imputation accuracy for these animals, previous work we have performed (Littlejohn et al. 2016) indicates accuracies of around 98%–99% when imputing BovineHD-resolution genotypes to WGS. Association analysis was conducted using the same GLS model described for SNP chip-based GWAS. Within the same intervals, gene expression phenotypes (described above) were used analogously to discover eQTL.

### Phase and complementarity relationships

To investigate phase relationships between edited sites and variants on the same transcript, WGS variants for each site with a significant edQTL were extracted.

These analyses were restricted to animals heterozygous for the implicated site, and only variants that were correlated *R*^2^ > 0.95 with the most significant variant, and were within 150 bases of the edit site, were included in these analyses. These criteria yielded 70 pairs of edit sites and WGS variants. Read pairs were then extracted from RNA-seq BAM files where the read pairs contained both the edit and variant positions. Reads meeting these criteria were subsequently counted to yield 2 × 2-contingency tables for the number edited/not edited for the edit site, and the number reference/alternative for the variant allele. Contingency tables of expected counts under independence were generated, and any pairs where at least one cell in either the observed or expected contingency table was less than six were excluded. This yielded 70 pairs that were then tested for independence using a *χ*^2^ test. Results where *P* < 7.14 × 10^−4^ were considered significant, applying a multiple testing threshold of *P* = 0.05/70.

Complementarity relationships were investigated by extracting pairs of edit sites that exhibited significant edQTL, and WGS variants that colocated to the same double-stranded secondary structure. Structures were determined using dot-plots and the RNAstructure software package (Reuter and Mathews 2010) as described above. Only WGS variants within 8 kbp of the edited site were considered. Additive allelic substitution effects $\left(\hat{\beta }\right)$ for the noncomplementary allele were extracted from the WGS-resolution edQTL analysis for each edit site/variant pair. As we hypothesized that decreased complementarity would decrease editing, a one-sample *t*-test was performed for the one-sided alternative hypothesis that mean $\hat{\beta }<0$.

To explore the relationship between RNA editing and transcript abundance, transcripts were quantified as described above, using only reads which mapped within or spliced across exons defined by the Ensembl annotations (release 81), then normalized using the variance-stabilizing transformation (VST). RNA editing was quantified using the logit function on the proportion of edited reads (Φ), with Pearson correlations and significance levels for transcript abundances calculated using R (R Core Team 2017). For the analysis that examined the potential impact of *ADAR* expression on these relationships, transcript abundances were fitted against *ADAR* expression using ordinary least squares, and the residuals extracted to generate adjusted abundance levels.

### Milk phenotypes and QTL

To examine the effect of RNA editing on milk production traits, milk fat, protein, and lactose phenotypes were derived for 9989 cows from measurements taken as part of standard herd-testing procedures. Milk samples were processed by LIC Testlink (Newstead) using Fourier transform infrared spectroscopy on Milkoscan FT6000 (FOSS) and Bentley FTS (Bentley) instruments. Individual phenotypic measurements for each animal were estimated using repeated measures models in ASReml-R as described in Lopdell et al. (2017).

These 9989 cows had previously been genotyped on several different SNP platforms: Illumina Bovine SNP50 (*N* = 6481), BovineHD (*N* = 62), and GeneSeek Genomic Profiler BeadChip (*N* = 3950; GeneSeek/Illumina). Five hundred and one cows had been genotyped on multiple panels. All cows were imputed to WGS resolution for the 134 edQTL intervals using Beagle 4 as described above. These genotypes were subsequently used with the milk-sample-derived phenotypes to explore QTL at each of these intervals, using the GLS model described above.

### Primer design and Sanger sequencing

To validate a subset of eight edit sites using Sanger sequencing, a randomly chosen subset of candidate sites was generated by targeting A-to-I sites with values above what could be anticipated to be detected via sequencing of diploid PCR products (nominal mean Φ of 0.21, median Φ > 0.165). Candidate sites were also restricted to those appearing in at least 305 animals (i.e., maximum of 50 animals where Φ = 0). Primers were then designed to amplify each candidate edit in an ∼300–400 bp amplicon.

Reverse transcriptase PCR (RT-PCR) and Sanger sequencing were performed to attempt to validate the subset of candidate variants. Synthesis of cDNA was performed using total RNA template in conjunction with random hexamer primers and the standard synthesis conditions of the SuperScript III First-Strand Synthesis System (Thermofisher). Four samples were targeted for cDNA synthesis, representing four of the nine animals used for the edit-site discovery phase of the broader study.

Primer sequences can be found in Supplemental Table S4. Generation of transcript amplicons was performed using KAPA2G Robust DNA polymerase (5 U/µL, KAPA Biosystems) with the following thermocycler conditions: 3 min initial denaturation at 95°C, followed by 40 cycles of 15 sec denaturation at 95°C, 15 sec annealing at 60°C, and 30 sec extension at 72°C, followed by a final extension at 72°C for 1 min.

Amplicons were visualized by electrophoresis on a 1.2% agarose gel prior to purification using NucleoSpin Gel and PCR Clean-up kit (Macherey-Nagel) and direct sequencing using Applied Biosystems BigDye version 3.1 terminator chemistry (Life Technologies/Applied Biosystems) on the Applied Biosystems 3130xL instrument (Life Technologies/Applied Biosystems). Sequencing was performed at the University of Auckland DNA Sequencing Facility (Auckland, New Zealand). Traces for five sites are presented in Supplemental Figure S1.

### Ethics approval

All animal experiments in this study were conducted in accordance with all rules and guidelines in the New Zealand Animal Welfare Act 1999. As the majority of data were generated as part of routine commercial activities, formal committee assessment and ethical approval (as defined by the above guidelines) were not required. Samples were obtained for the mammary tissue biopsy experiment in accordance with protocols approved by the Ruakura Animal Ethics Committee, Hamilton, New Zealand (approval number AEC 12845). No animals were killed for this study.

## DATA DEPOSITION

Sequence (BAM) files containing the WGS and RNA-seq reads sequenced from the nine discovery set cows, have been uploaded to the NCBI sequence read archive (SRA). BioProject accession number: PRJNA446068 (SRP136662); BioSample accession numbers: SAMN08810150 to SAMN08810167.

## SUPPLEMENTAL MATERIAL

Supplemental material is available for this article.

## COMPETING INTEREST STATEMENT

T.J.L., C.C., K.T., S.R.D., B.L.H., and M.D.L. are employees of Livestock Improvement Corporation, a commercial provider of bovine germplasm. The remaining authors declare that they have no competing interests.

## Supplementary Material

Supplemental Material
