# Supplementary material for: Widespread cis-regulation of RNA editing in a large mammal
Source: RNA. 2019 Mar;25(3):319–35. doi: 10.1261/rna.066902.118 (PMC6380278; doi:10.1261/rna.066902.118)
Supplement: Supplemental Material [file supp_25_3_319__index.html]

Widespread cis-regulation of RNA editing in a large mammal — Supplemental Material 

# Widespread *cis*-regulation of RNA editing in a large mammal

## Supplemental Material

- Supplemental\_Figure\_S1.pdf
- Supplemental\_Figure\_S2.pdf
- Supplemental\_Figure\_S3.pdf
- Supplemental\_Figure\_S4.tiff
- Supplemental\_Figure\_S5.pdf
- Supplemental\_Figure\_S6.tiff
- Supplemental\_Figure\_S7.tiff
- Supplemental\_Legends.docx
- Supplemental\_Table\_S1.xlsx
- Supplemental\_Table\_S2.xlsx
- Supplemental\_Table\_S3.xlsx
- Supplemental\_Table\_S4.xlsx
