# Supplementary material for: Widespread cis-regulation of RNA editing in a large mammal
Source: RNA. 2019 Mar;25(3):319–35. doi: 10.1261/rna.066902.118 (PMC6380278; doi:10.1261/rna.066902.118)

Site 6 - Chr15:65834689

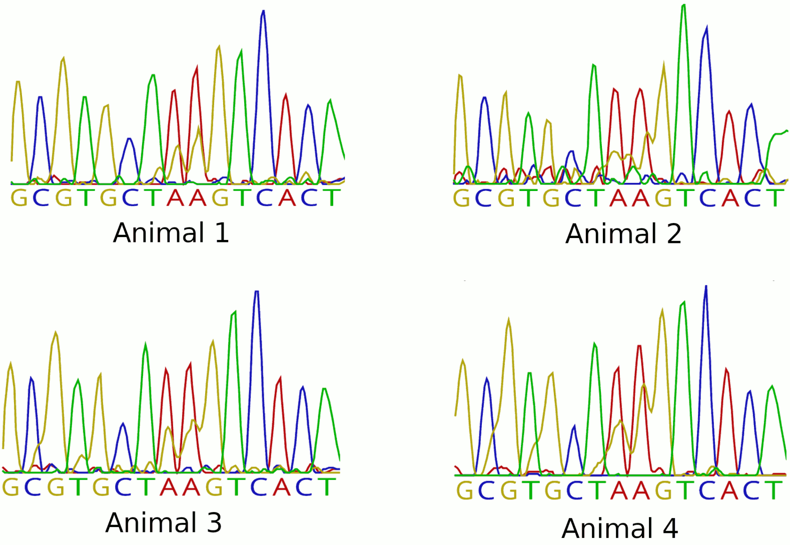

Site 7 - Chr16:29640359

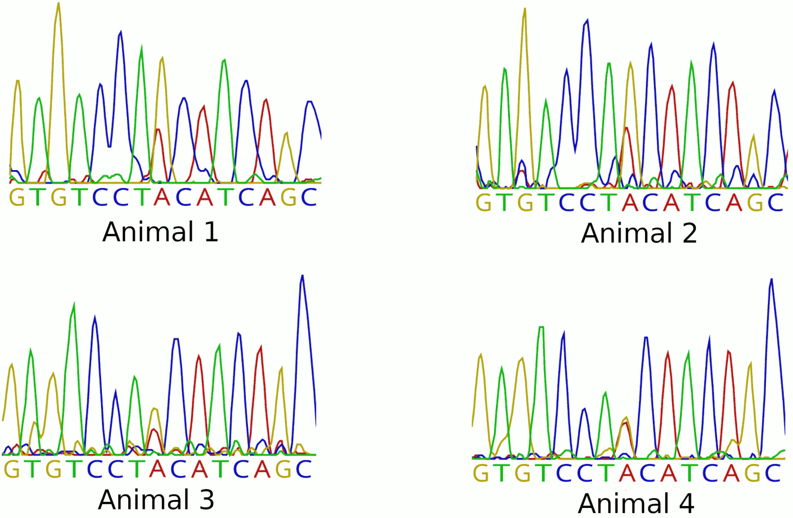

Site 14 - Chr13:51750150

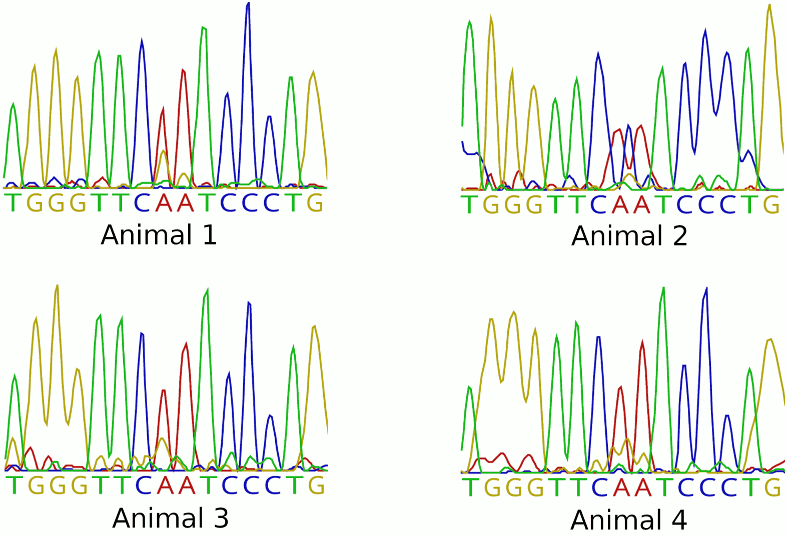

Site 18 - Chr6:59687528

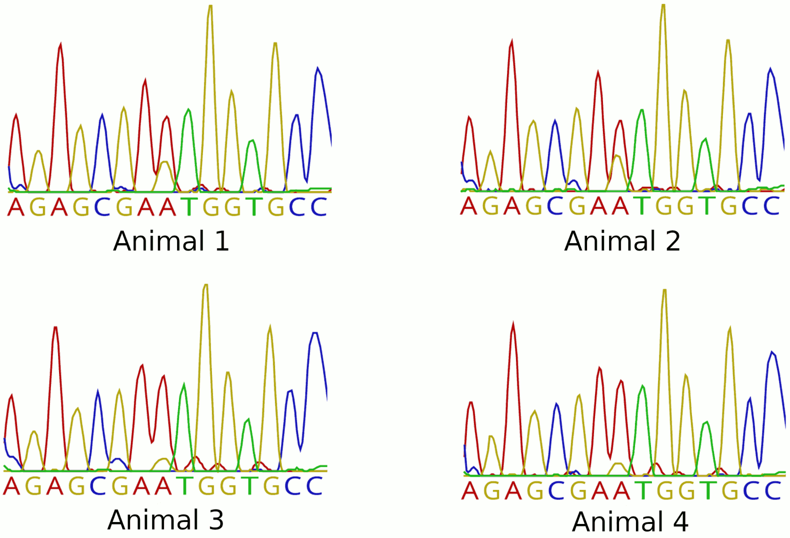

Site 20 - Chr17:60341051

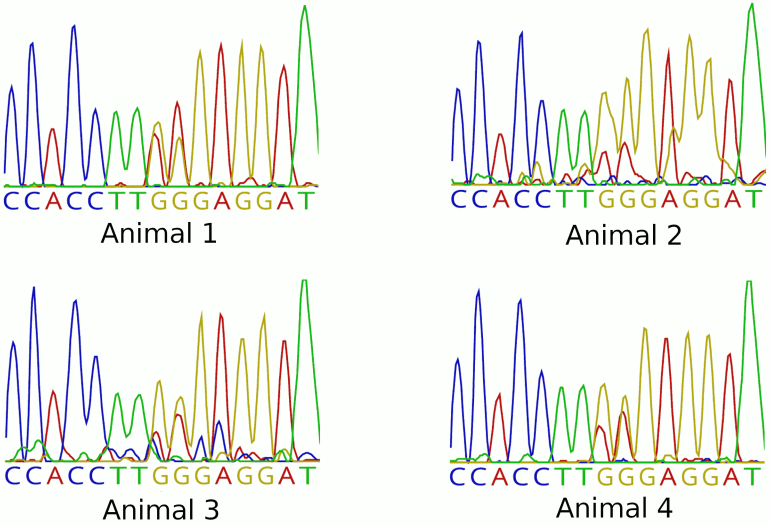

Supplement: Supplemental Material [file supp_066902.118_Supplemental_Figure_S1.pdf]
