# Supplementary material for: Widespread cis-regulation of RNA editing in a large mammal
Source: RNA. 2019 Mar;25(3):319–35. doi: 10.1261/rna.066902.118 (PMC6380278; doi:10.1261/rna.066902.118)
Supplement: Supplemental Material [file supp_066902.118_Supplemental_Figure_S2.pdf]

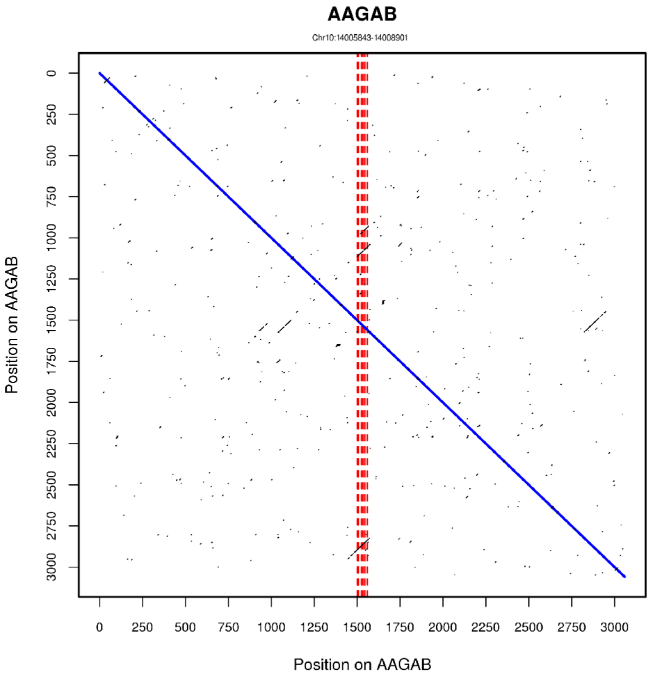
AAGAB
Chr10:14005843-14008901
Position on AAGAB
Position on AAGAB

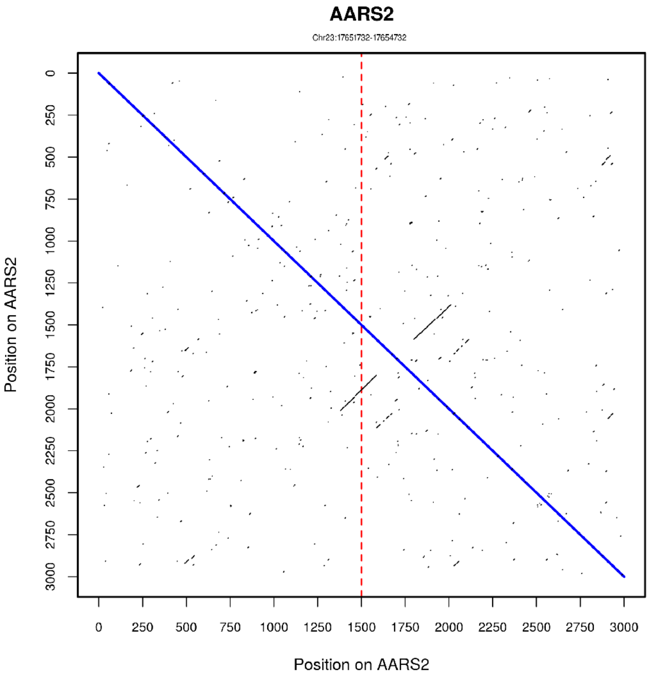
AARS2
Chr23:17651732-17654732
Position on AARS2
Position on AARS2

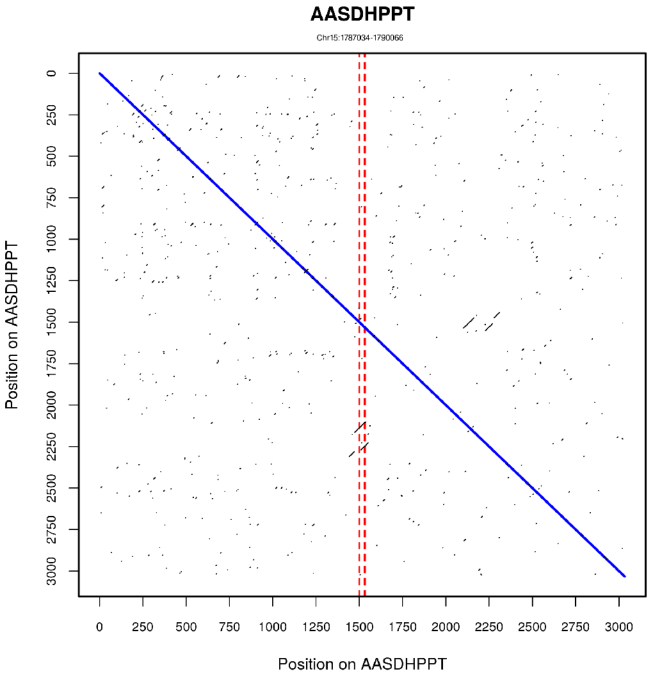
AASDHPPT
Chr15:1787034-1790066
Position on AASDHPPT
Position on AASDHPPT

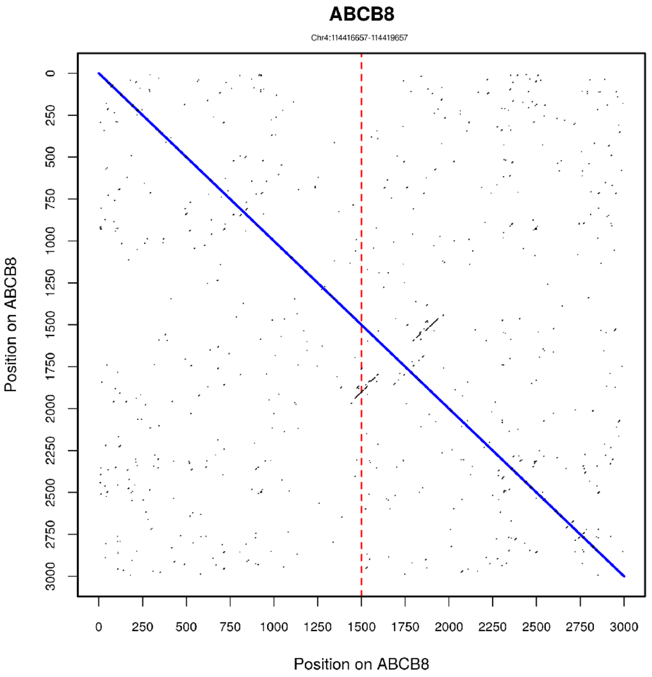
ABCB8
Chr4:114416657-114419657
Position on ABCB8
Position on ABCB8

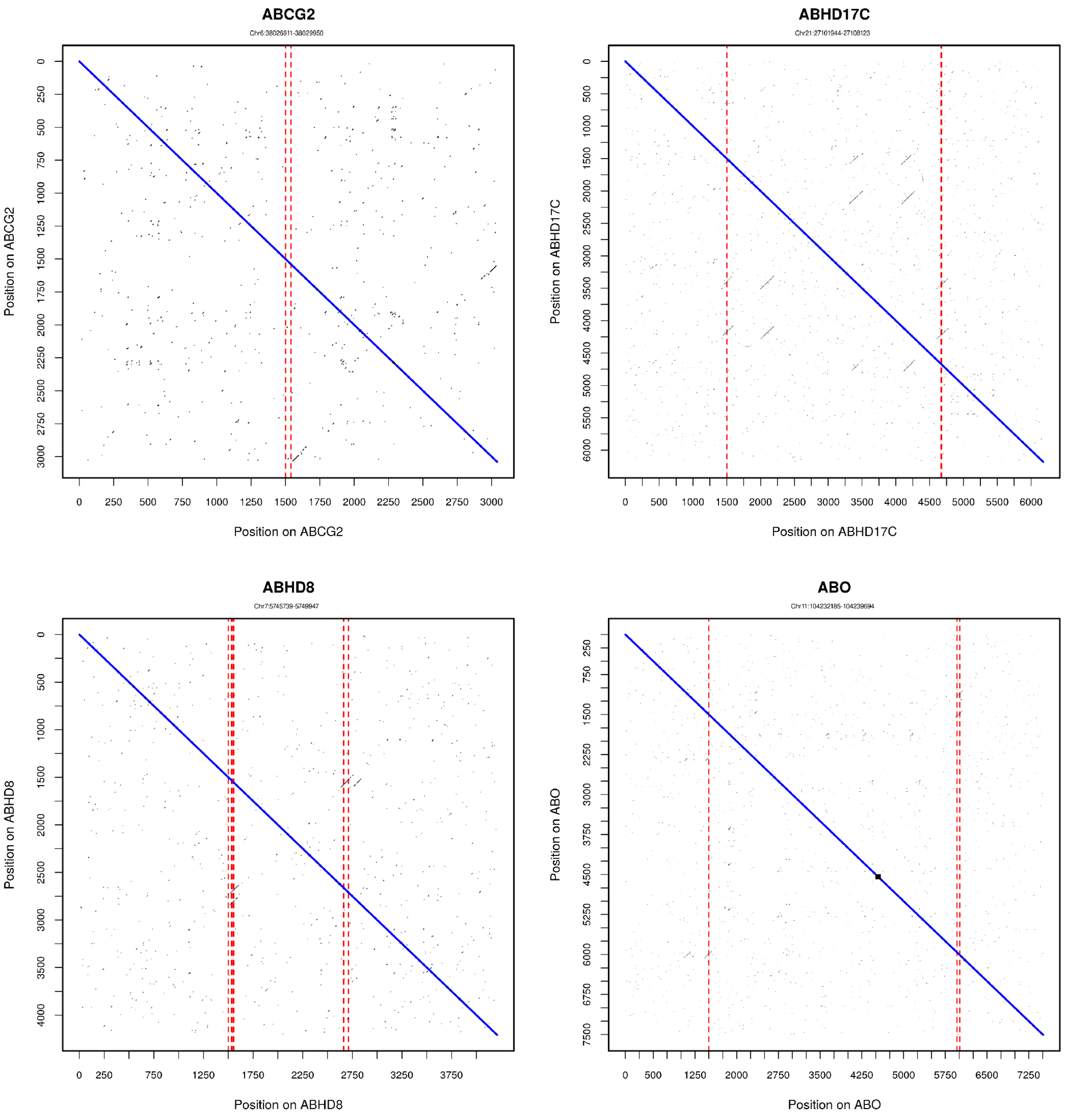
ABCG2
Chr6:38026911-38029950
Position on ABCG2
Position on ABCG2
ABHD17C
Chr21:27101944-27108123
Position on ABHD17C
Position on ABHD17C
ABHD8
Chr7:5745739-5749947
Position on ABHD8
Position on ABHD8
ABO
Chr11:104232185-104239694
Position on ABO
Position on ABO

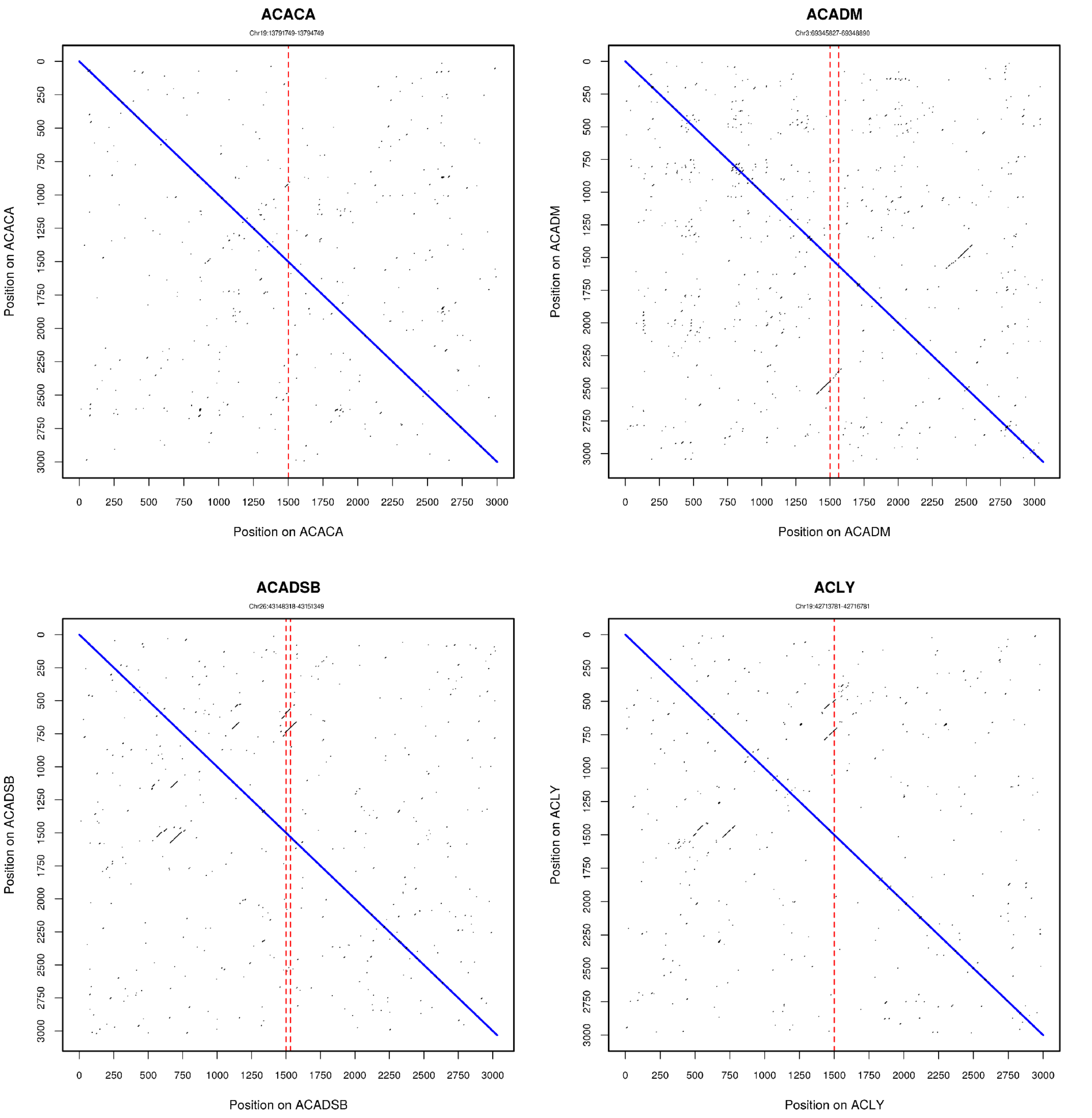

ACACA
Chr19:13791749-13794749
Position on ACACA
Position on ACACA
ACADM
Chr3:69345827-69348890
Position on ACADM
Position on ACADM
ACADSB
Chr26:43148318-43151349
Position on ACADSB
Position on ACADSB
ACLY
Chr19:42713781-42716781
Position on ACLY
Position on ACLY

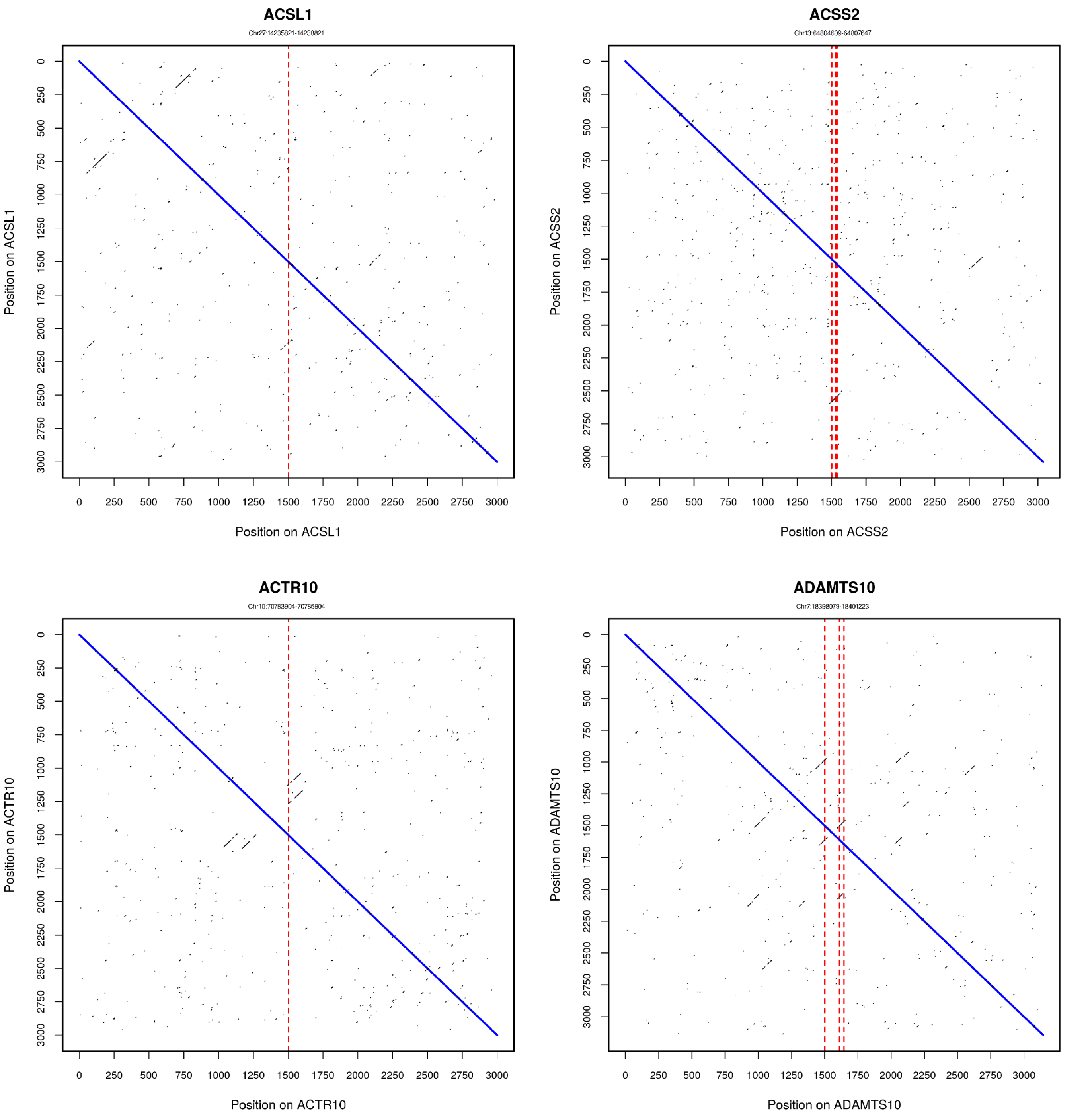
ACSL1
Chr27:14235821-14238821
Position on ACSL1
Position on ACSL1
ACSS2
Chr13:64804609-64807647
Position on ACSS2
Position on ACSS2
ACTR10
Chr10:70783904-70786904
Position on ACTR10
Position on ACTR10
ADAMTS10
Chr7:18398079-18401223
Position on ADAMTS10
Position on ADAMTS10

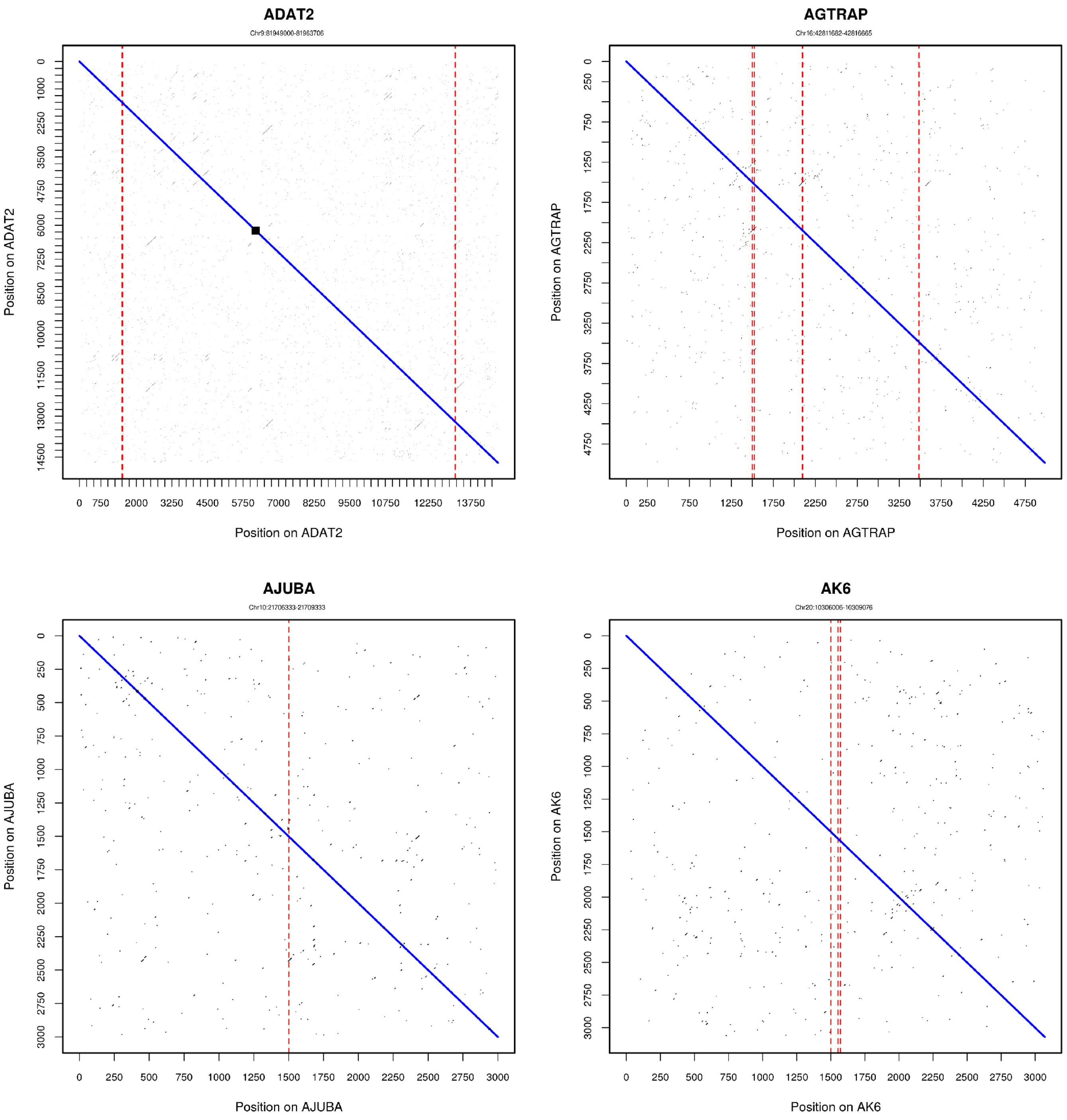
ADAT2
Chr9:81949000-81963706
Position on ADAT2
Position on ADAT2
0
750
2000
3250
4500
5750
7000
8250
9500
10750
12250
13750
1000
2250
3500
4750
6000
7250
8500
10000
11500
13000
14500
AGTRAP
Chr16:42811682-42816665
Position on AGTRAP
Position on AGTRAP
0
250
750
1250
1750
2250
2750
3250
3750
4250
4750
AJUBA
Chr10:21706333-21709333
Position on AJUBA
Position on AJUBA
0
250
500
750
1000
1250
1500
1750
2000
2250
2500
2750
3000
AK6
Chr20:10306006-10309076
Position on AK6
Position on AK6
0
250
500
750
1000
1250
1500
1750
2000
2250
2500
2750
3000

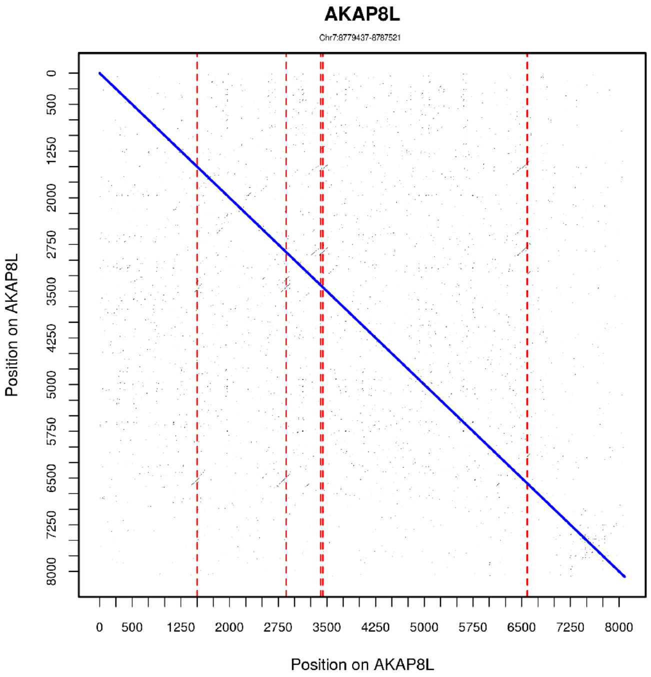
AKAP8L
Chr7:8779437-8787521
Position on AKAP8L
Position on AKAP8L

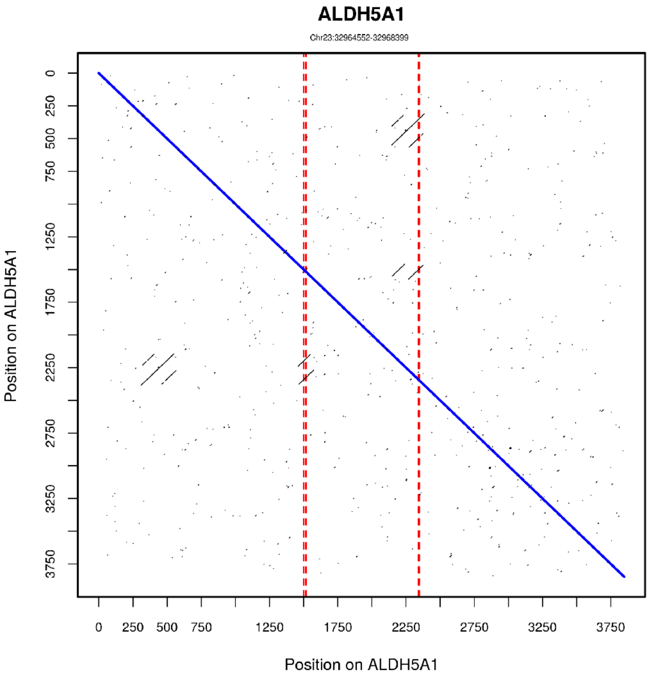
ALDH5A1
Chr23:32964552-32968399
Position on ALDH5A1
Position on ALDH5A1

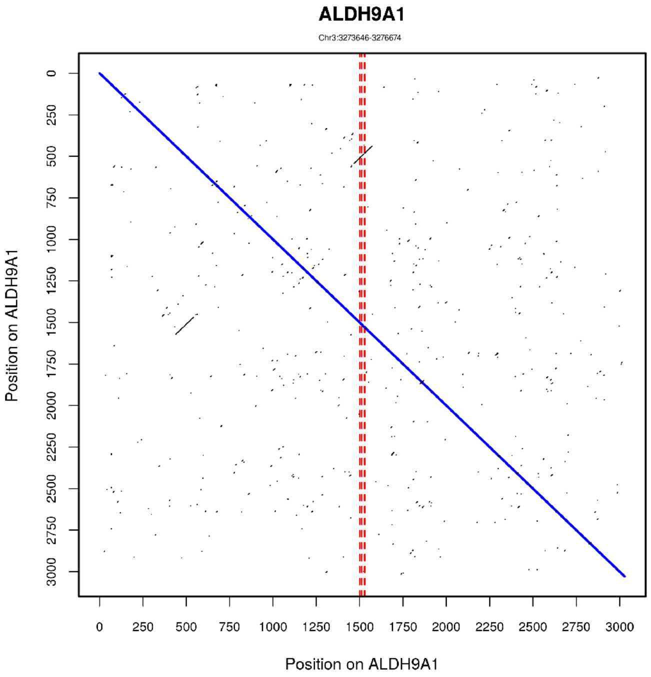
ALDH9A1
Chr3:3273646-3276674
Position on ALDH9A1
Position on ALDH9A1

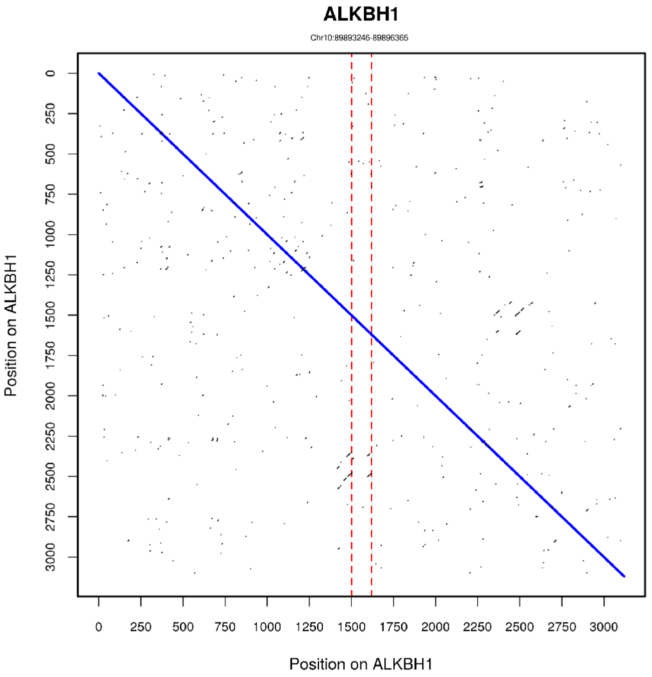
ALKBH1
Chr10:89893246-89896365
Position on ALKBH1
Position on ALKBH1

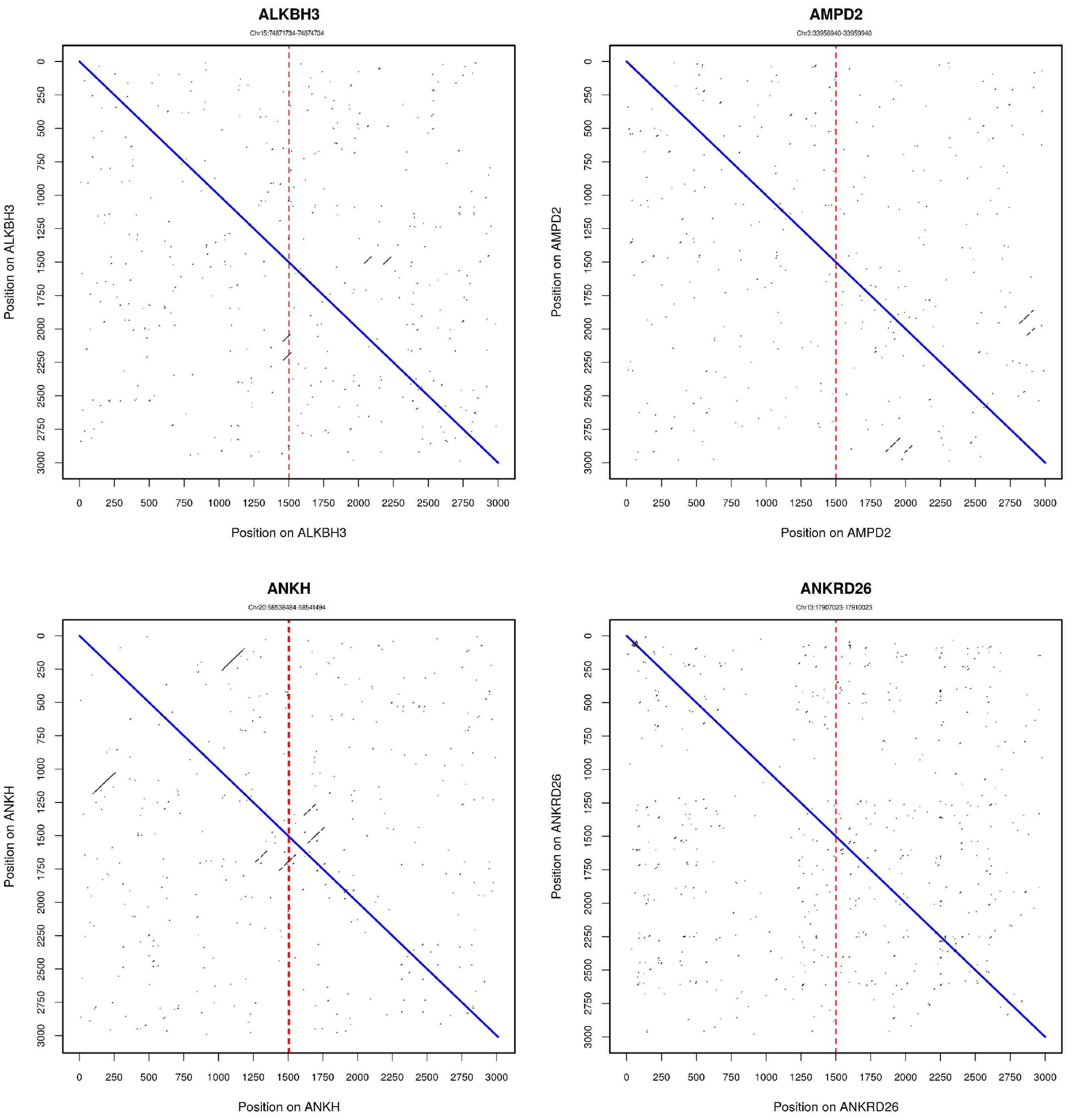
ALKBH3
Chr15:74871734-74874734
Position on ALKBH3
Position on ALKBH3
AMPD2
Chr3:33956940-33959940
Position on AMPD2
Position on AMPD2
ANKH
Chr20:58538484-58541494
Position on ANKH
Position on ANKH
ANKRD26
Chr13:17907023-17910023
Position on ANKRD26
Position on ANKRD26

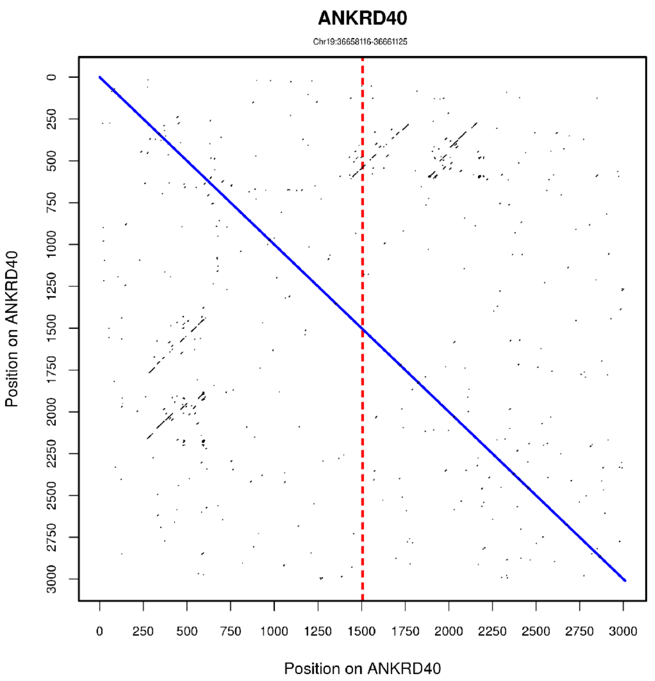
ANKRD40
Chr19:36658116-36661125
Position on ANKRD40
Position on ANKRD40

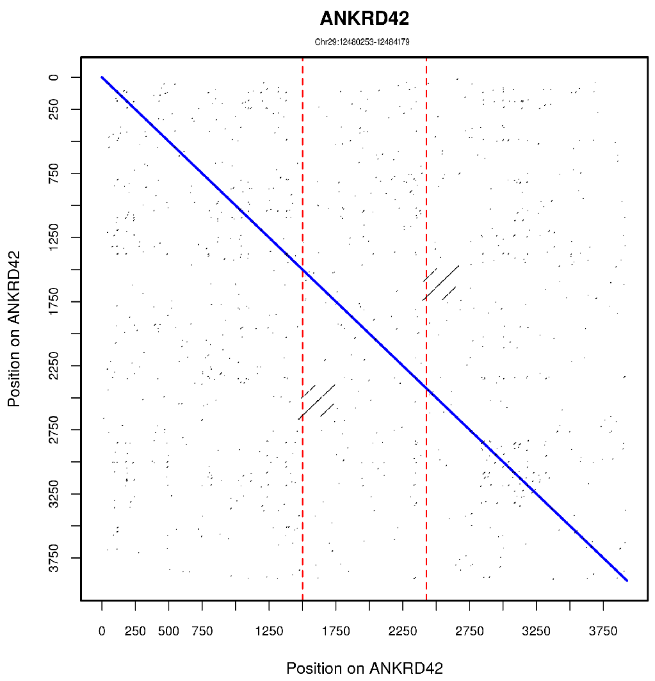
ANKRD42
Chr29:12480253-12484179
Position on ANKRD42
Position on ANKRD42

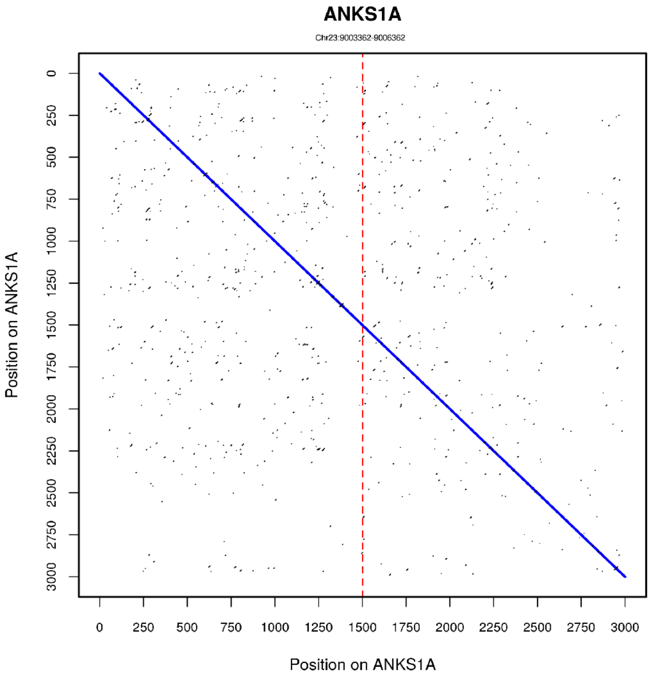
ANKS1A
Chr23:9003362-9006362
Position on ANKS1A
Position on ANKS1A

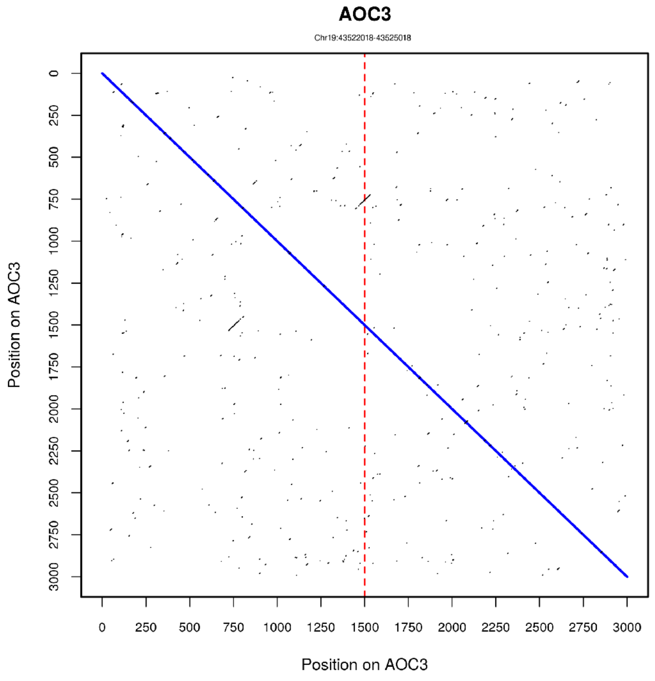
AOC3
Chr19:43522018-43525018
Position on AOC3
Position on AOC3

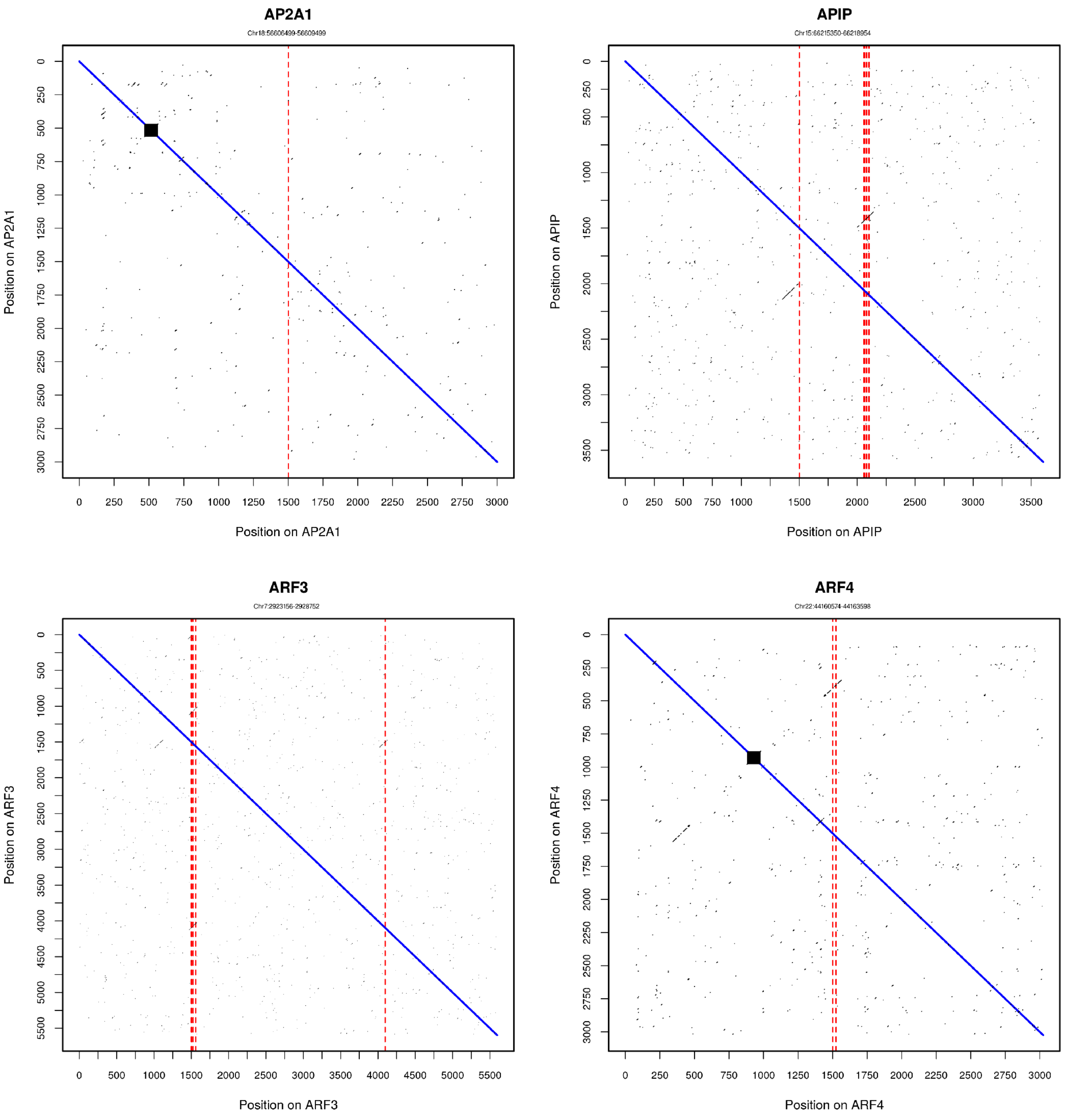
AP2A1
Chr18:56606499-56609499
Position on AP2A1
Position on AP2A1
APIP
Chr15:66215350-66218954
Position on APIP
Position on APIP
ARF3
Chr7:2923156-2928752
Position on ARF3
Position on ARF3
ARF4
Chr22:44160574-44163598
Position on ARF4
Position on ARF4

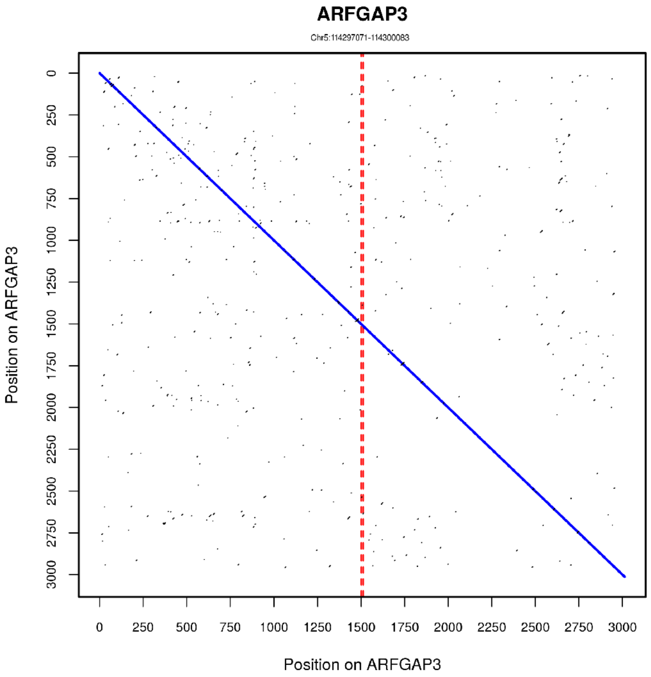
ARFGAP3
Chr5:114297071-114300083
Position on ARFGAP3
Position on ARFGAP3

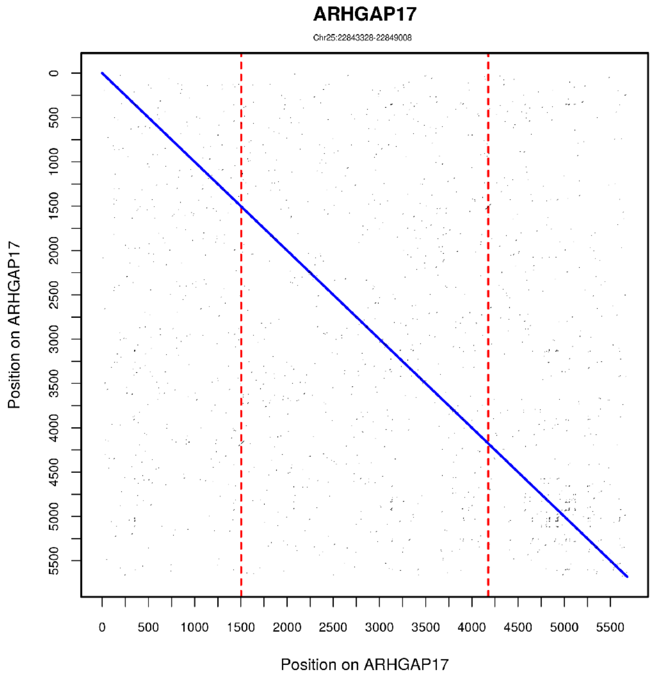
ARHGAP17
Chr25:22843328-22849008
Position on ARHGAP17
Position on ARHGAP17

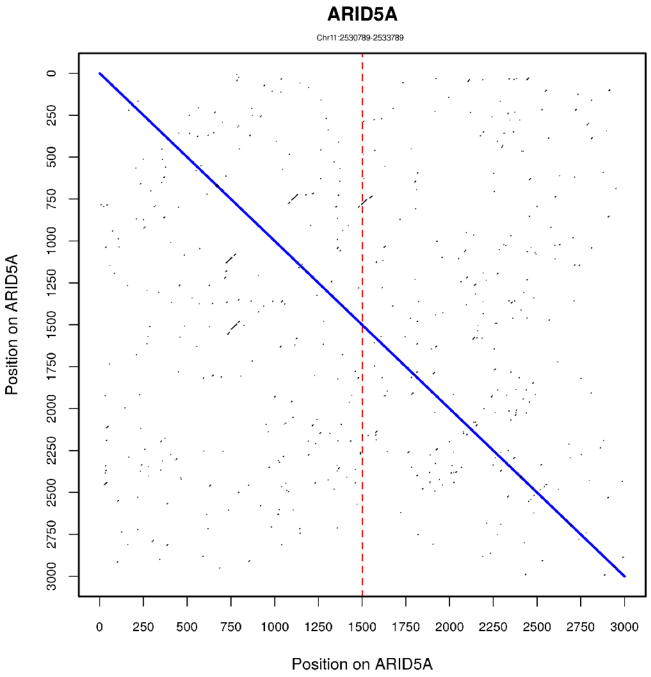
ARID5A
Chr11:2530789-2533789
Position on ARID5A
Position on ARID5A

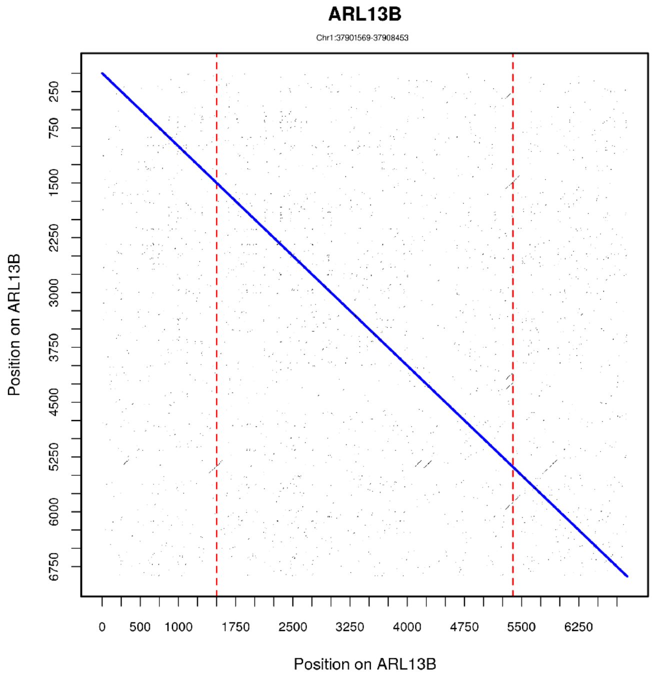
ARL13B
Chr1:37901569-37908453
Position on ARL13B
Position on ARL13B

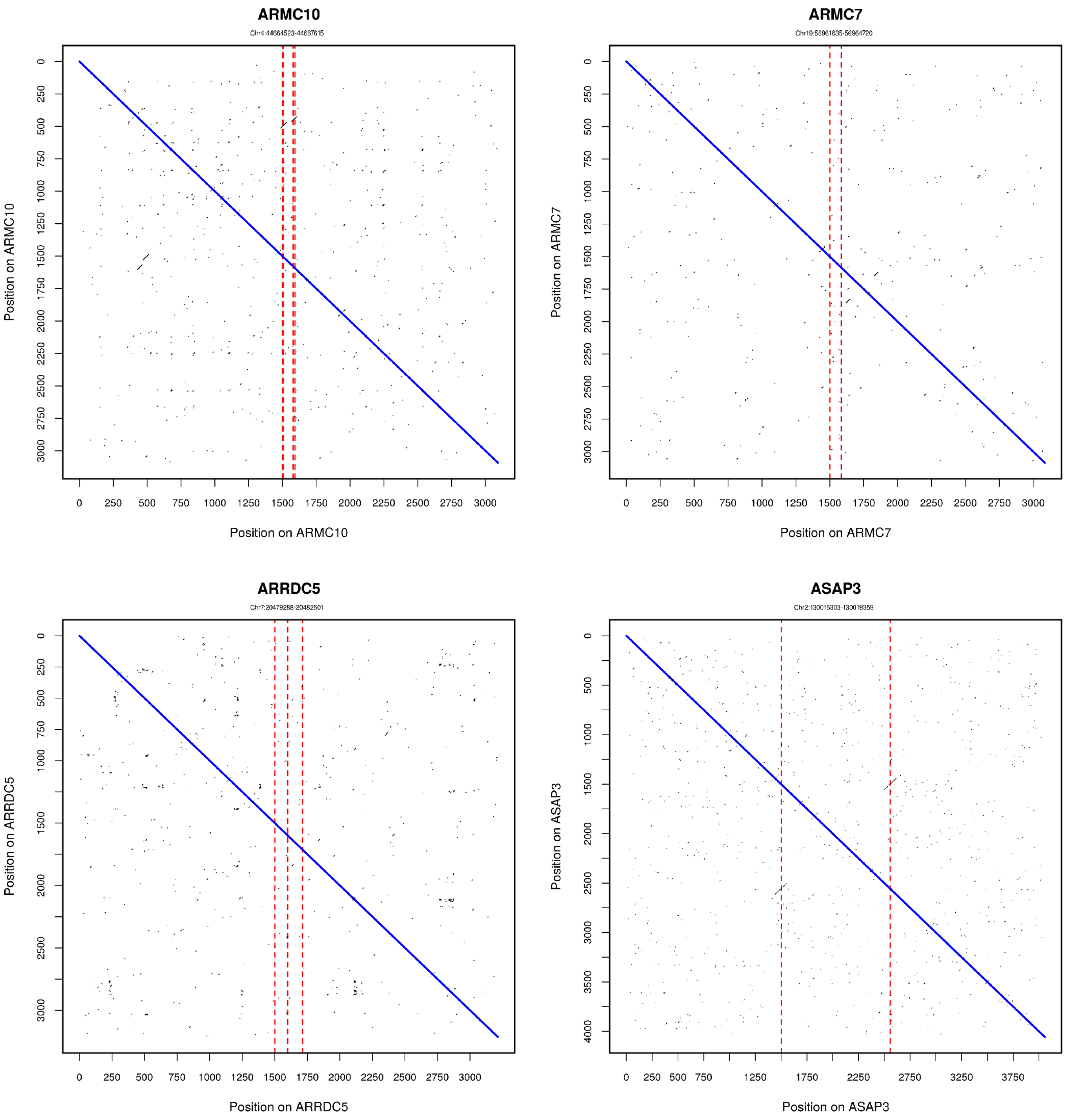
ARMC10
Chr4:44664523-44667615
Position on ARMC10
Position on ARMC10
ARMC7
Chr19:56961635-56964720
Position on ARMC7
Position on ARMC7
ARRDC5
Chr7:20479288-20482501
Position on ARRDC5
Position on ARRDC5
ASAP3
Chr2:130015303-130019359
Position on ASAP3
Position on ASAP3

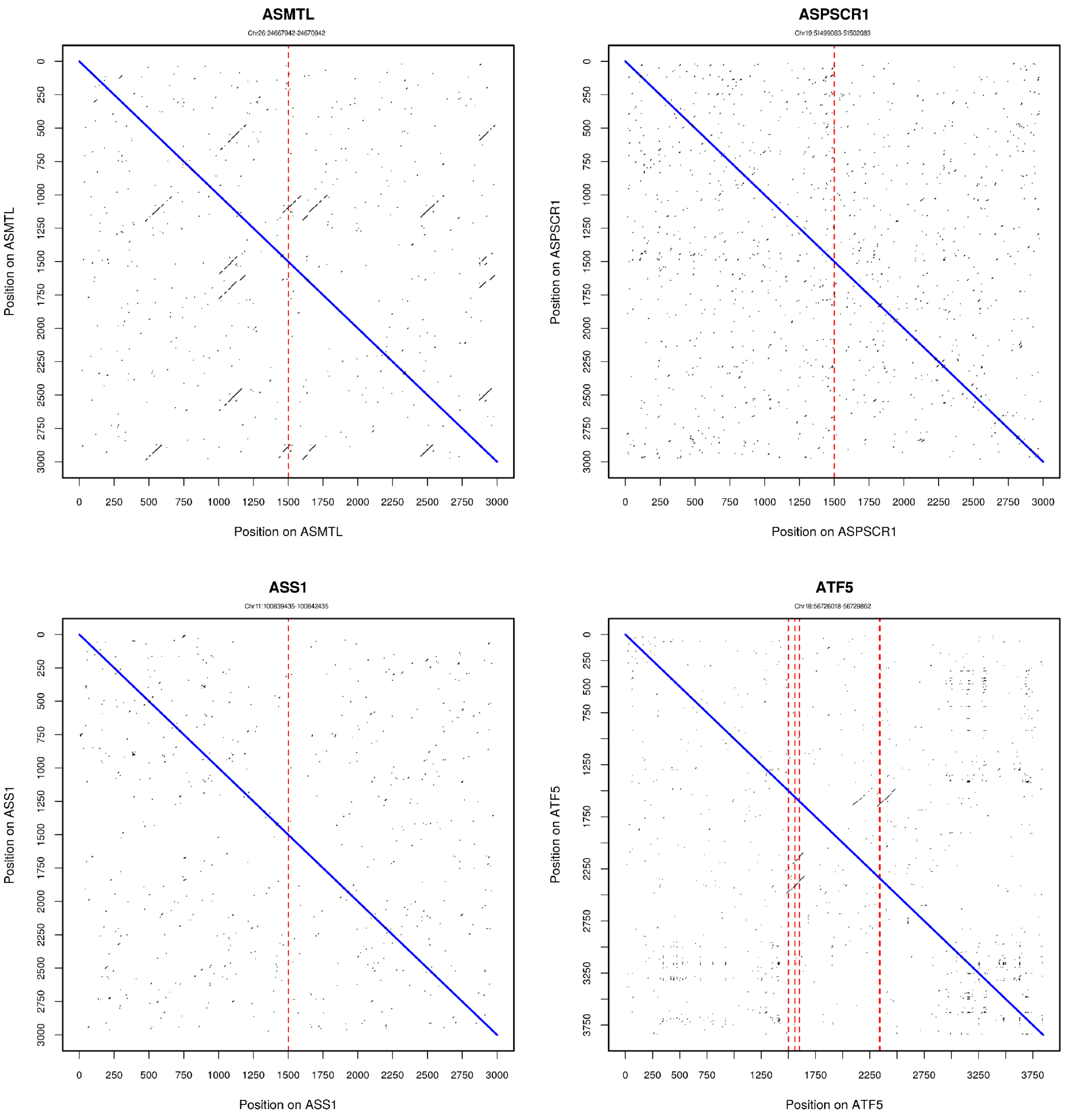
ASMTL
Chr26:24667942-24670942
Position on ASMTL
Position on ASMTL
ASPSCR1
Chr19:51499083-51502083
Position on ASPSCR1
Position on ASPSCR1
ASS1
Chr11:100839435-100842435
Position on ASS1
Position on ASS1
ATF5
Chr18:56726018-56729862
Position on ATF5
Position on ATF5

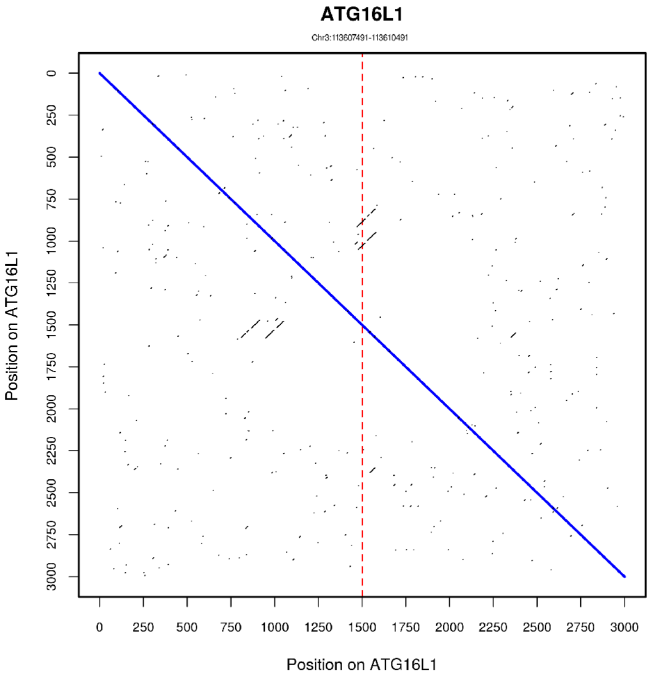
ATG16L1
Chr3:113607491-113610491
Position on ATG16L1
Position on ATG16L1

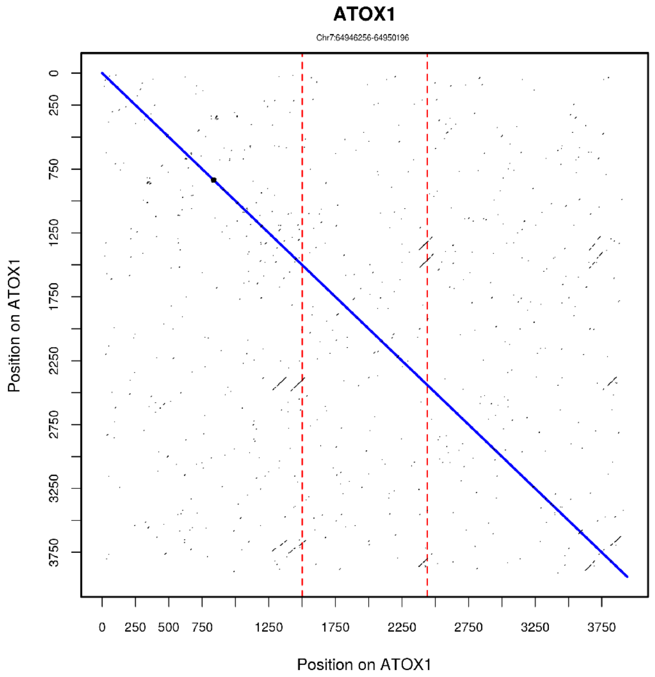
ATOX1
Chr7:64946256-64950196
Position on ATOX1
Position on ATOX1

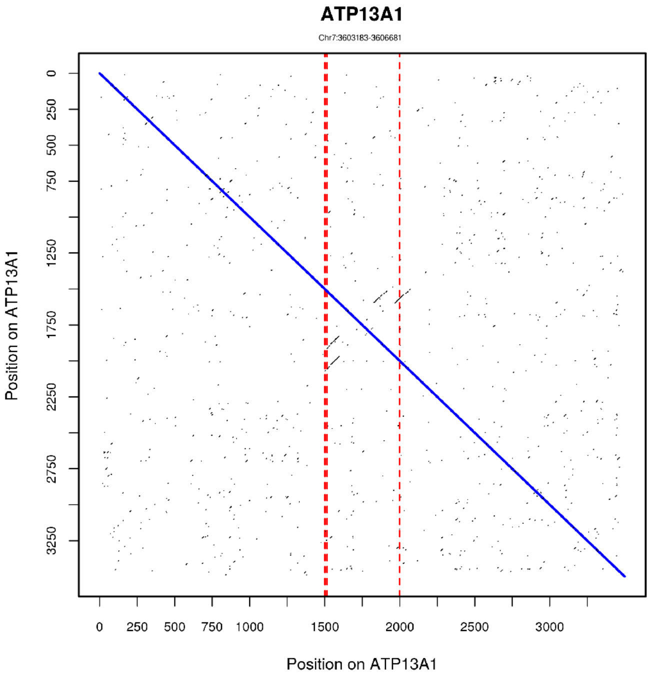
ATP13A1
Chr7:3603183-3606681
Position on ATP13A1
Position on ATP13A1

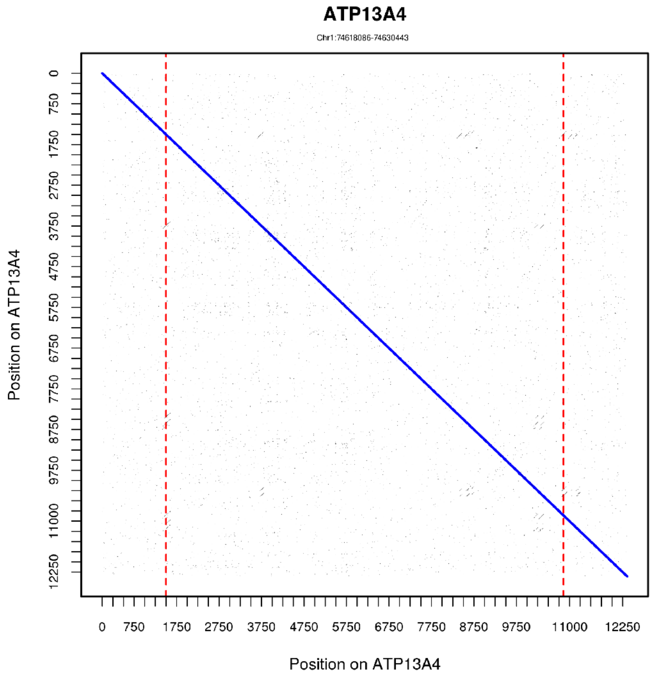
ATP13A4
Chr1:74618086-74630443
Position on ATP13A4
Position on ATP13A4

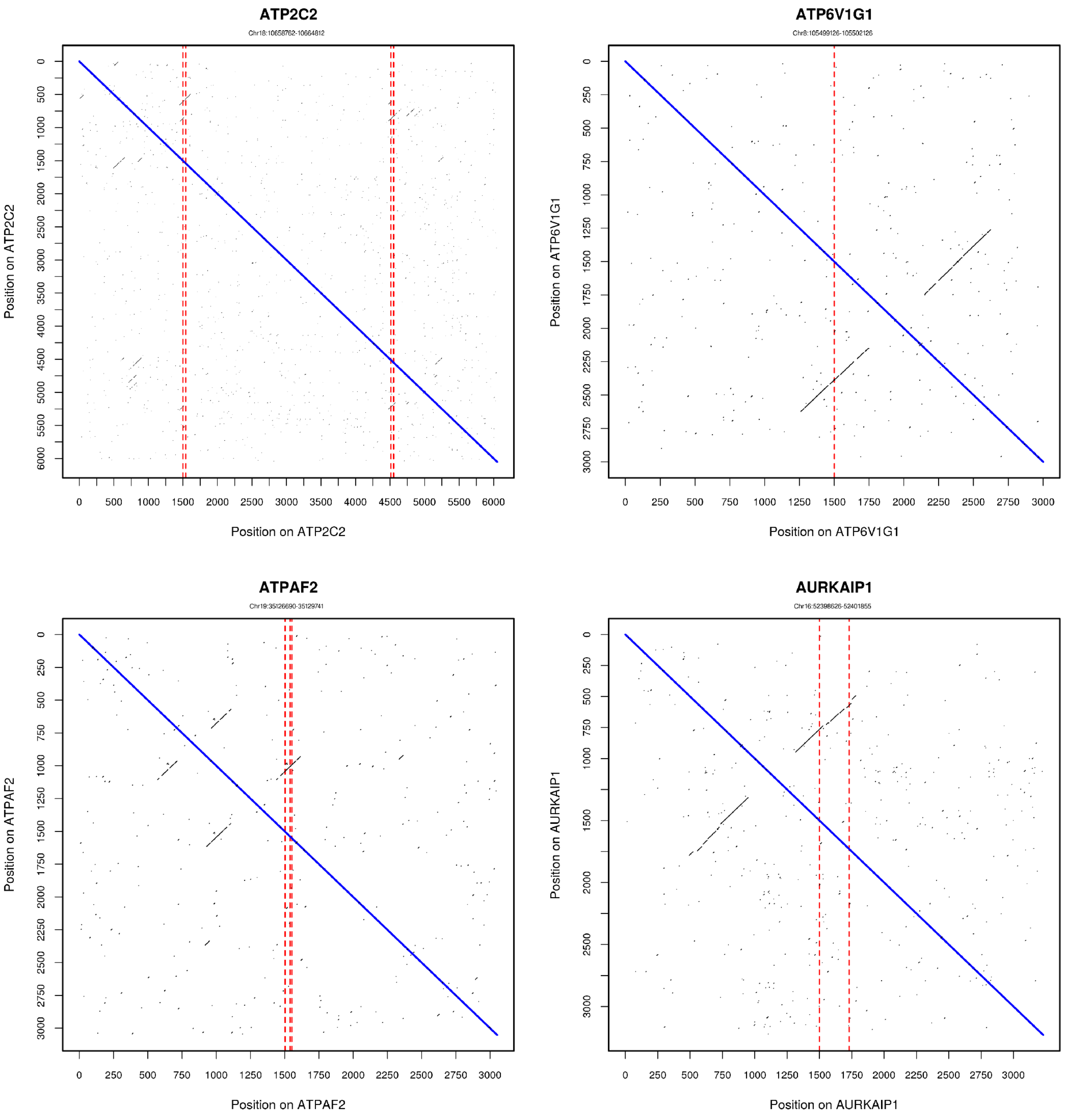

ATP2C2
Chr18:10658762-10664812
Position on ATP2C2
Position on ATP2C2
ATP6V1G1
Chr8:105499126-105502126
Position on ATP6V1G1
Position on ATP6V1G1
ATPAF2
Chr19:35126690-35129741
Position on ATPAF2
Position on ATPAF2
AURKAIP1
Chr16:52398626-52401855
Position on AURKAIP1
Position on AURKAIP1

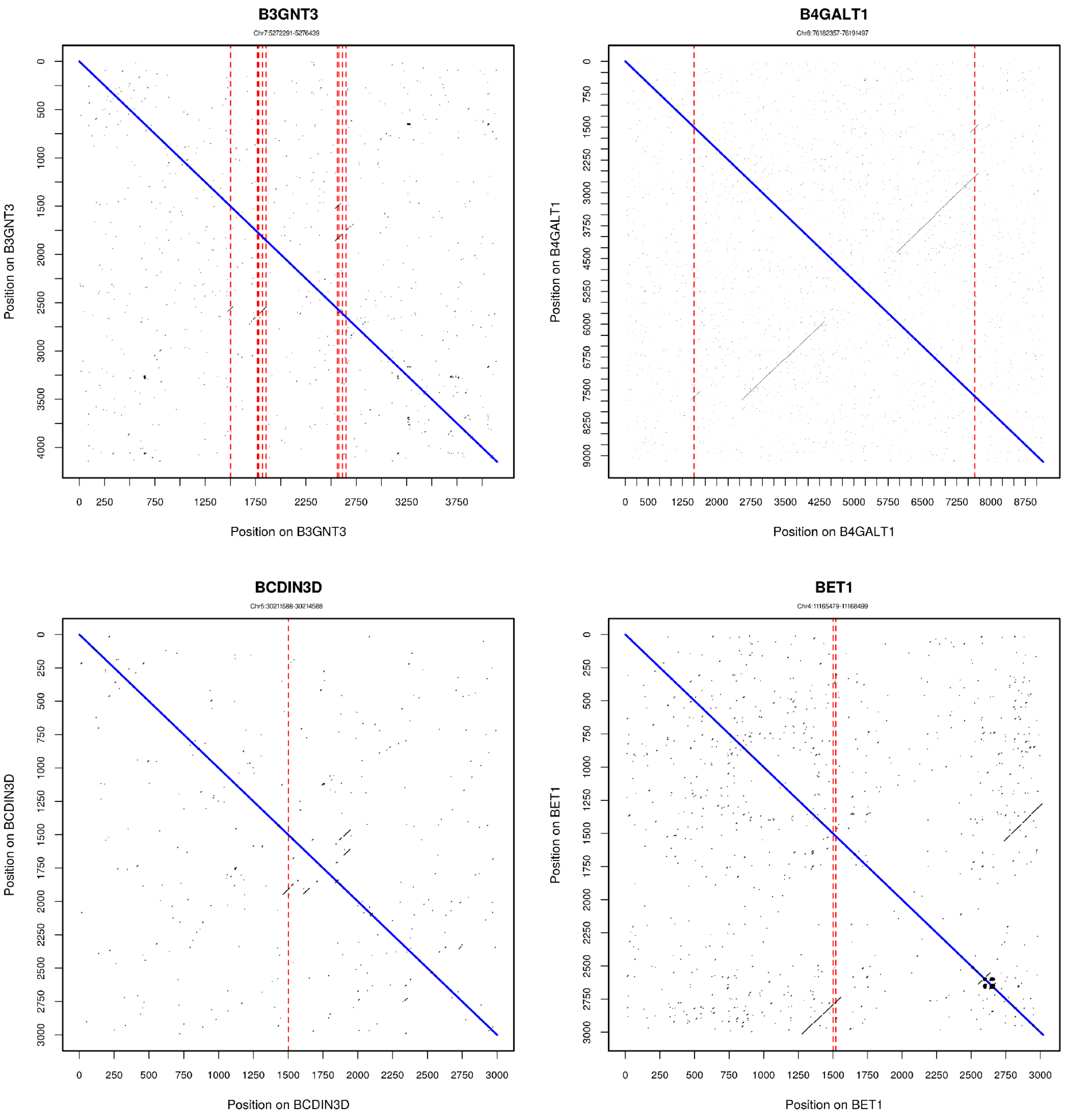
B3GNT3
Chr7:5272291-5276439
Position on B3GNT3
Position on B3GNT3
B4GALT1
Chr8:76182357-76191497
Position on B4GALT1
Position on B4GALT1
BCDIN3D
Chr5:30211588-30214588
Position on BCDIN3D
Position on BCDIN3D
BET1
Chr4:11165479-11168499
Position on BET1
Position on BET1

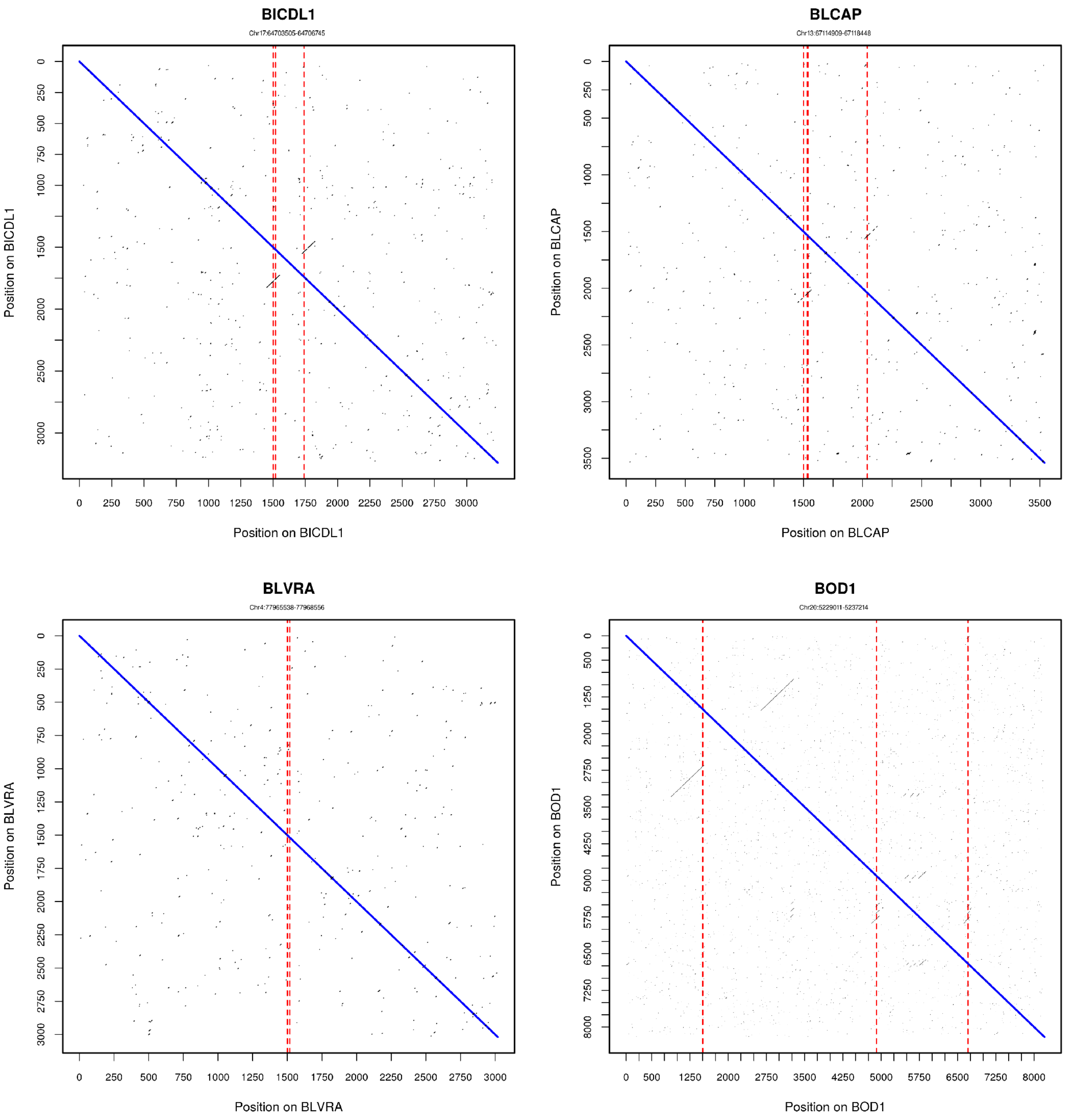

BICDL1
Chr17:64703505-64706745
Position on BICDL1
Position on BICDL1
BLCAP
Chr13:67114909-67118448
Position on BLCAP
Position on BLCAP
BLVRA
Chr4:77965538-77968556
Position on BLVRA
Position on BLVRA
BOD1
Chr20:5229011-5237214
Position on BOD1
Position on BOD1

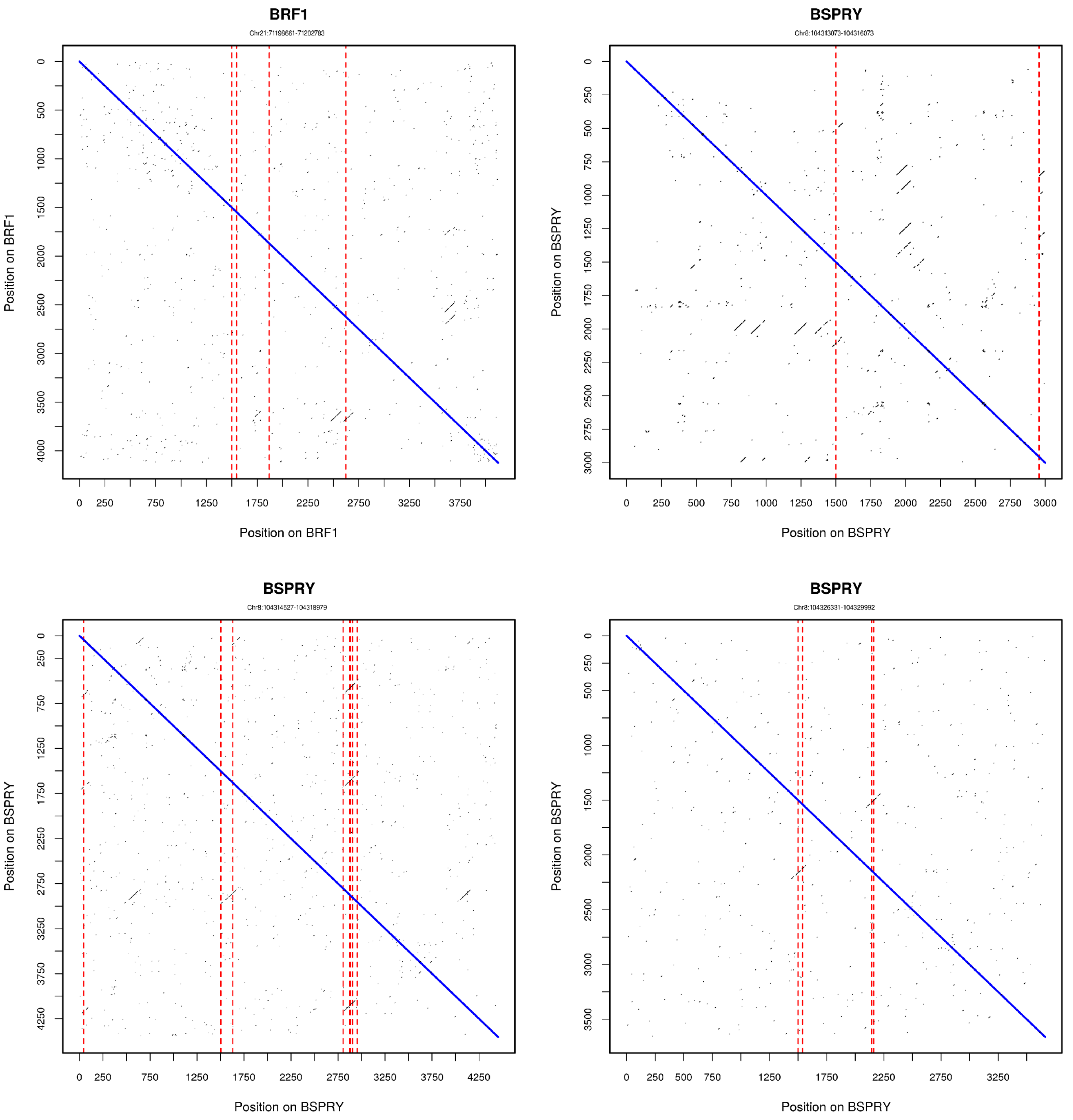

BRF1
Chr21:71198661-71202783
Position on BRF1
Position on BRF1
BSPRY
Chr8:104313073-104316073
Position on BSPRY
Position on BSPRY
BSPRY
Chr8:104314527-104318979
Position on BSPRY
Position on BSPRY
BSPRY
Chr8:104326331-104329992
Position on BSPRY
Position on BSPRY

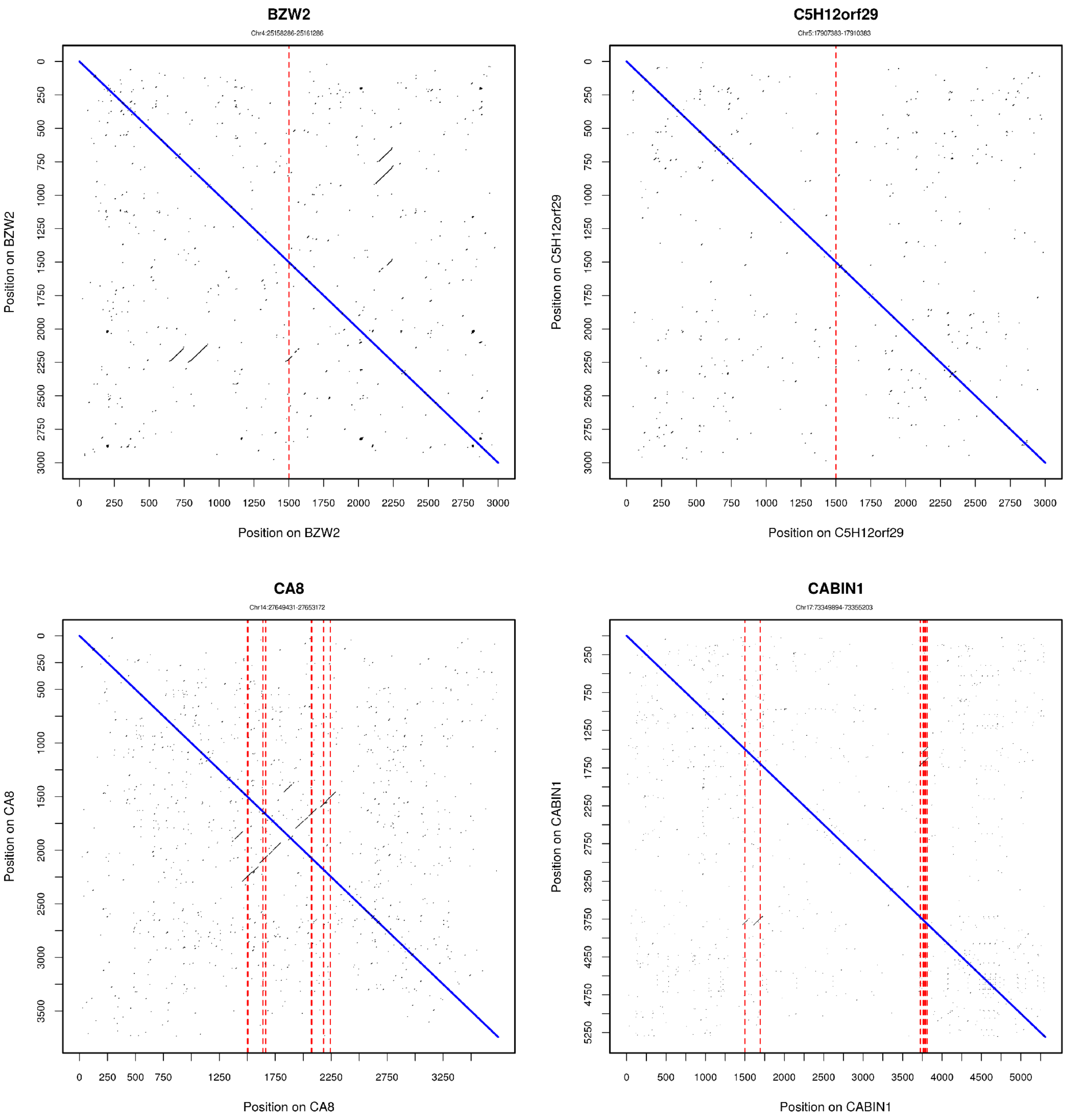
BZW2
Chr4:25158286-25161286
Position on BZW2
Position on BZW2
C5H12orf29
Chr5:17907383-17910383
Position on C5H12orf29
Position on C5H12orf29
CA8
Chr14:27649431-27653172
Position on CA8
Position on CA8
CABIN1
Chr17:73349894-73355203
Position on CABIN1
Position on CABIN1

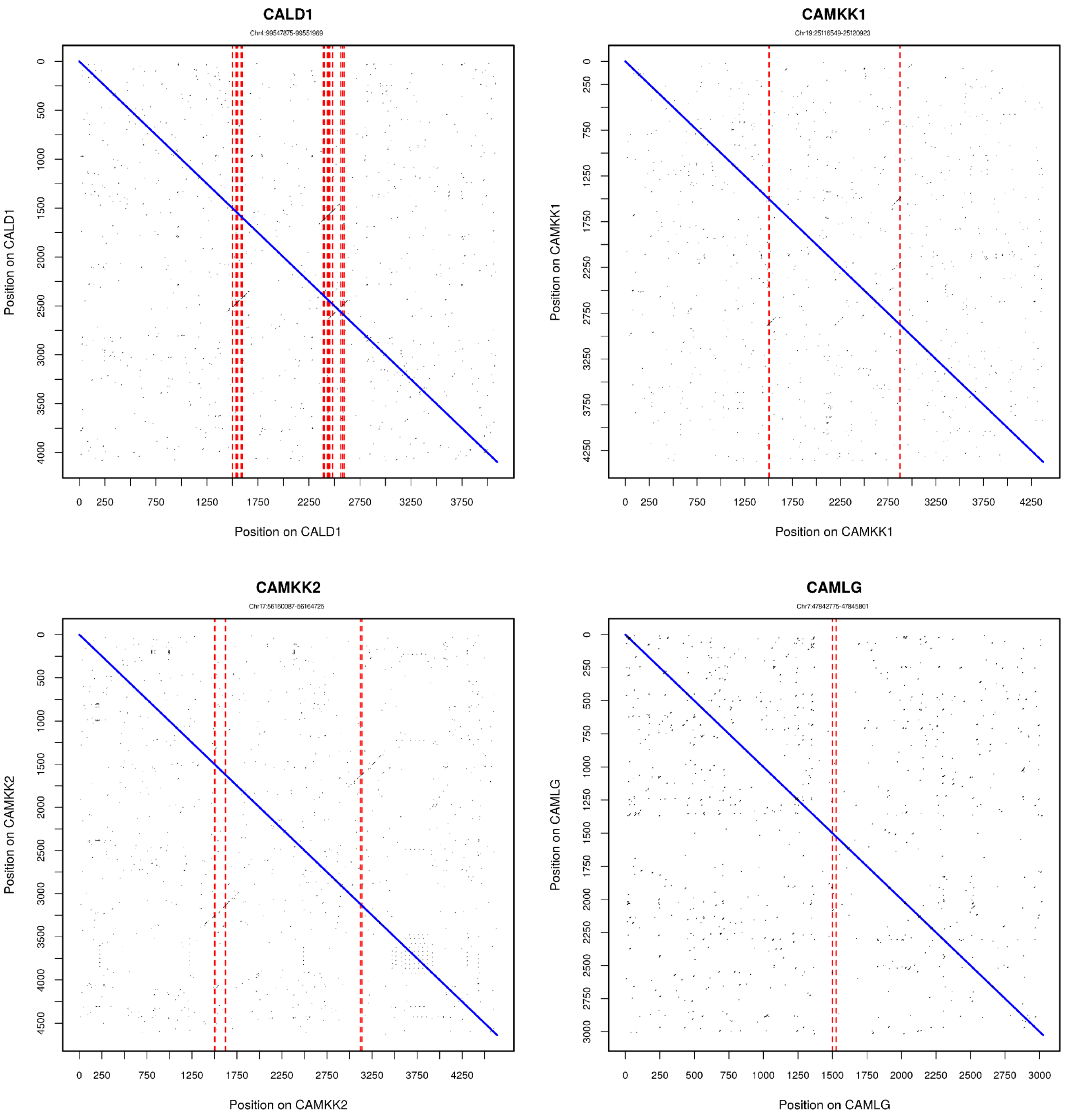
CALD1
Chr4:99547875-99551969
Position on CALD1
Position on CALD1
CAMKK1
Chr19:25116549-25120923
Position on CAMKK1
Position on CAMKK1
CAMKK2
Chr17:56160087-56164725
Position on CAMKK2
Position on CAMKK2
CAMLG
Chr7:47842775-47845801
Position on CAMLG
Position on CAMLG

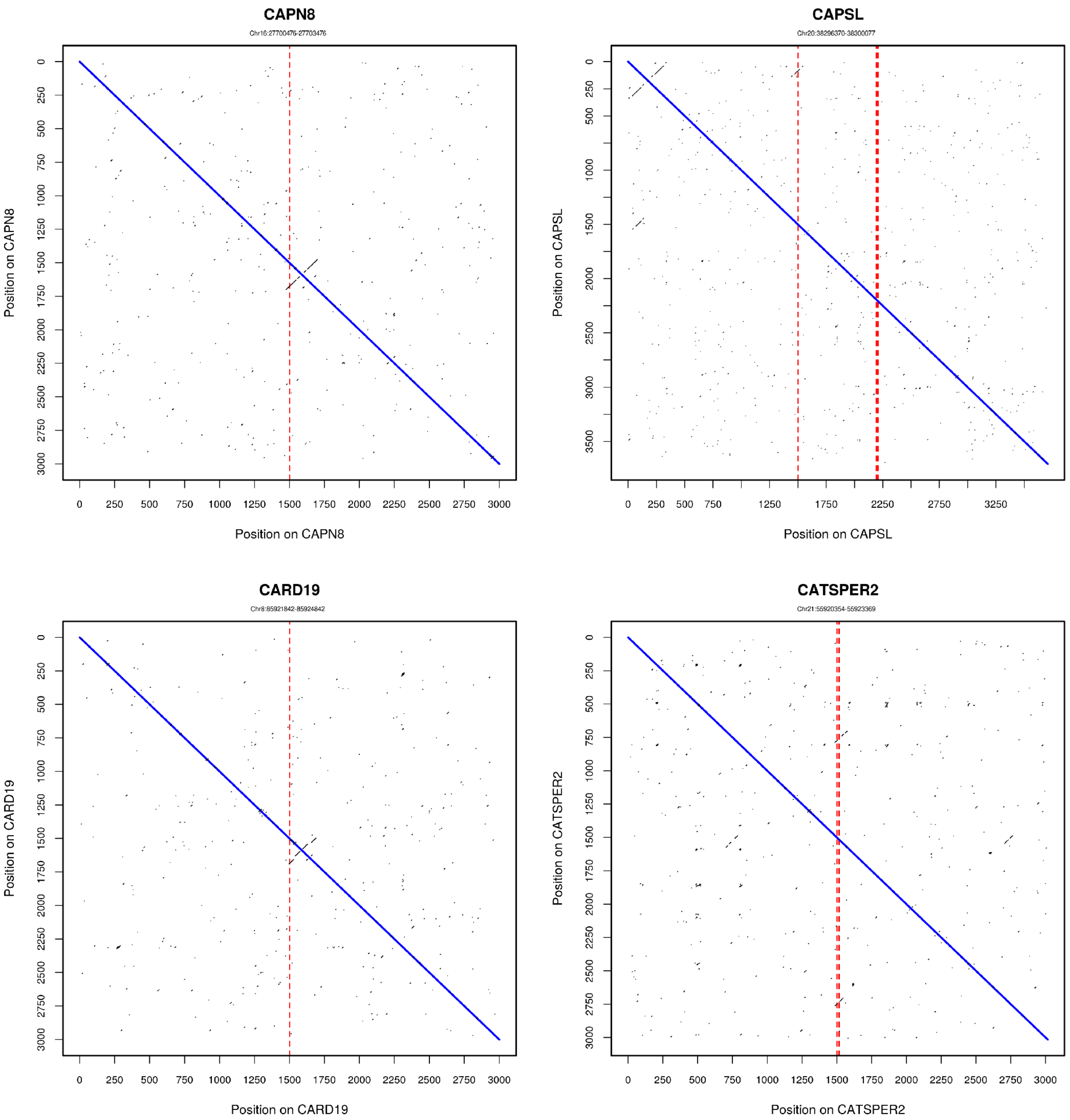

CAPN8
Chr16:27700476-27703476
Position on CAPN8
Position on CAPN8
CAPSL
Chr20:38296370-38300077
Position on CAPSL
Position on CAPSL
CARD19
Chr8:85921842-85924842
Position on CARD19
Position on CARD19
CATSPER2
Chr21:55920354-55923369
Position on CATSPER2
Position on CATSPER2

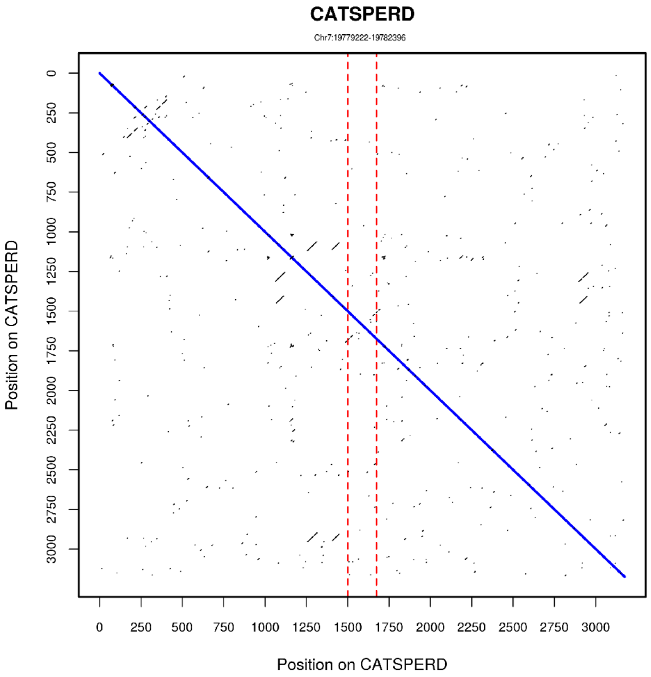
CATSPERD
Chr7:19779222-19782396
Position on CATSPERD
Position on CATSPERD

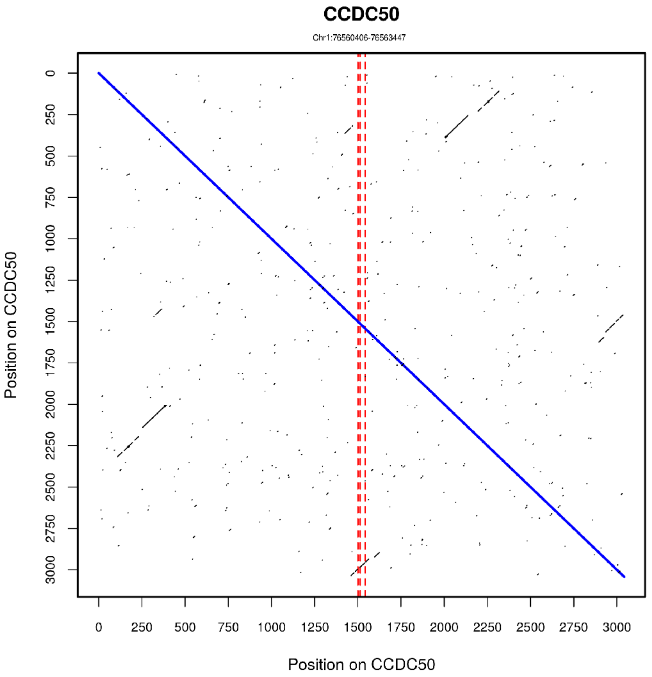
CCDC50
Chr1:76560406-76563447
Position on CCDC50
Position on CCDC50

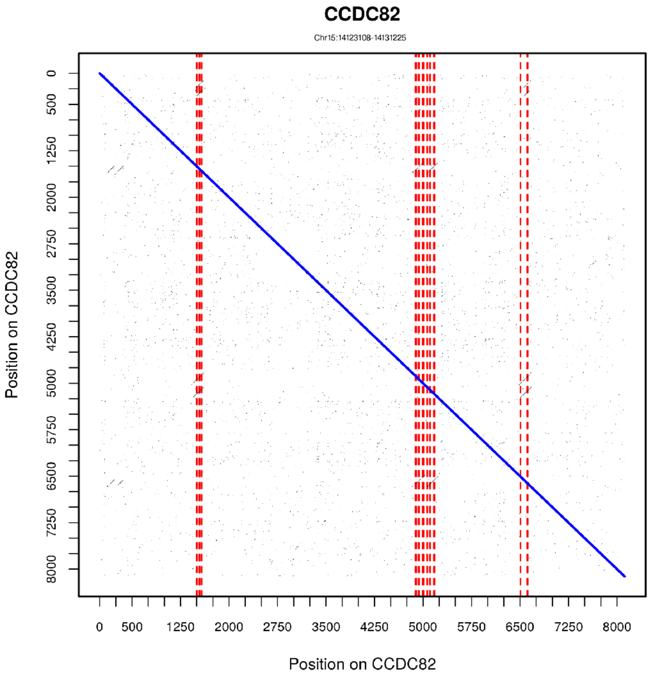
CCDC82
Chr15:14123108-14131225
Position on CCDC82
Position on CCDC82

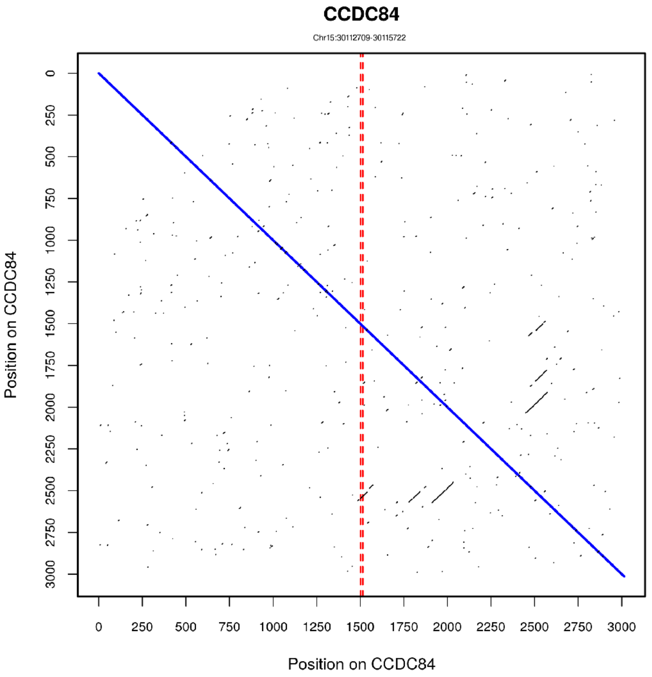
CCDC84
Chr15:30112709-30115722
Position on CCDC84
Position on CCDC84

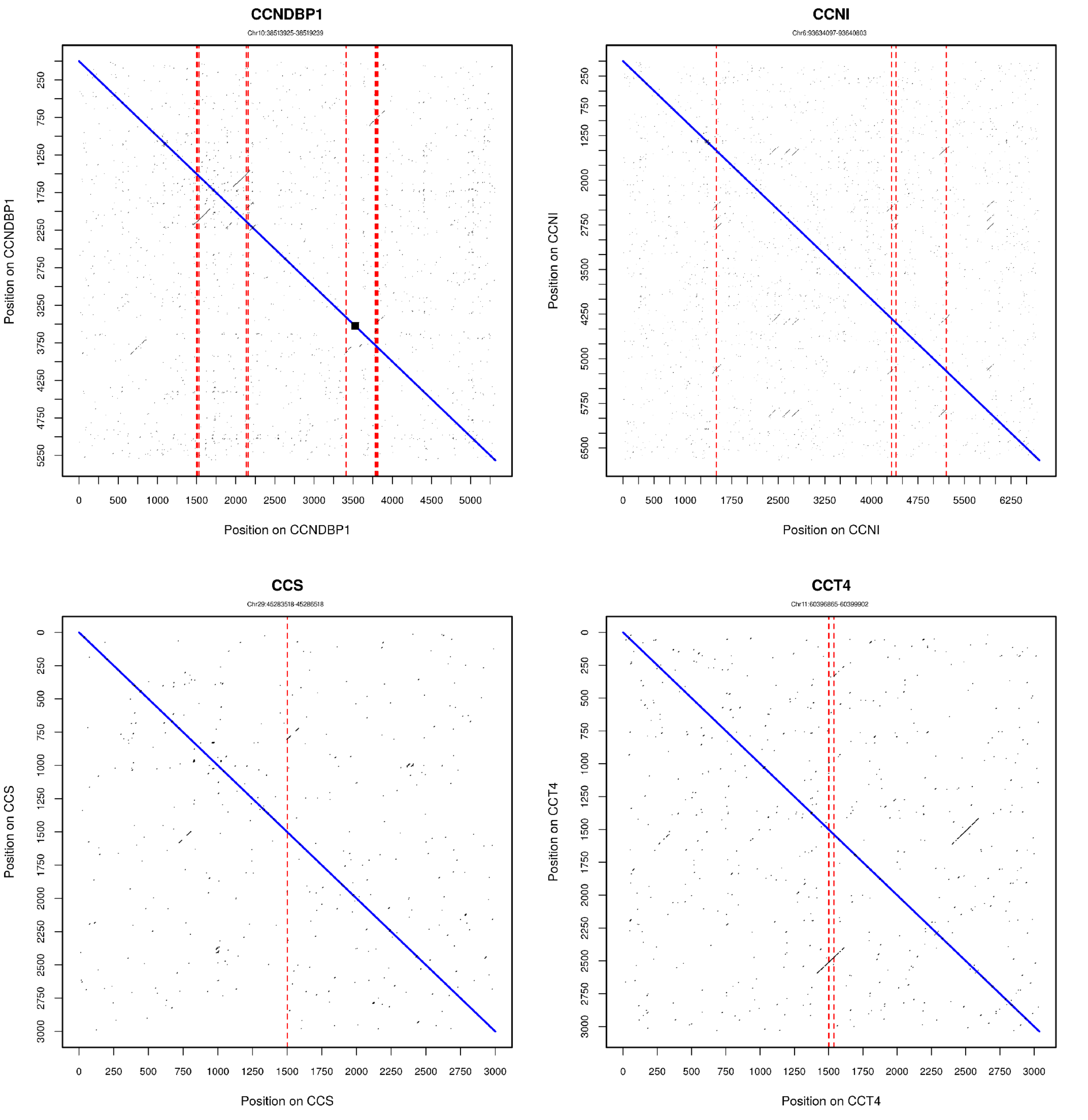
CCNDBP1
Chr10:38513925-38519239
Position on CCNDBP1
Position on CCNDBP1
CCNI
Chr6:93634097-93640803
Position on CCNI
Position on CCNI
CCS
Chr29:45283518-45286518
Position on CCS
Position on CCS
CCT4
Chr11:60396865-60399902
Position on CCT4
Position on CCT4

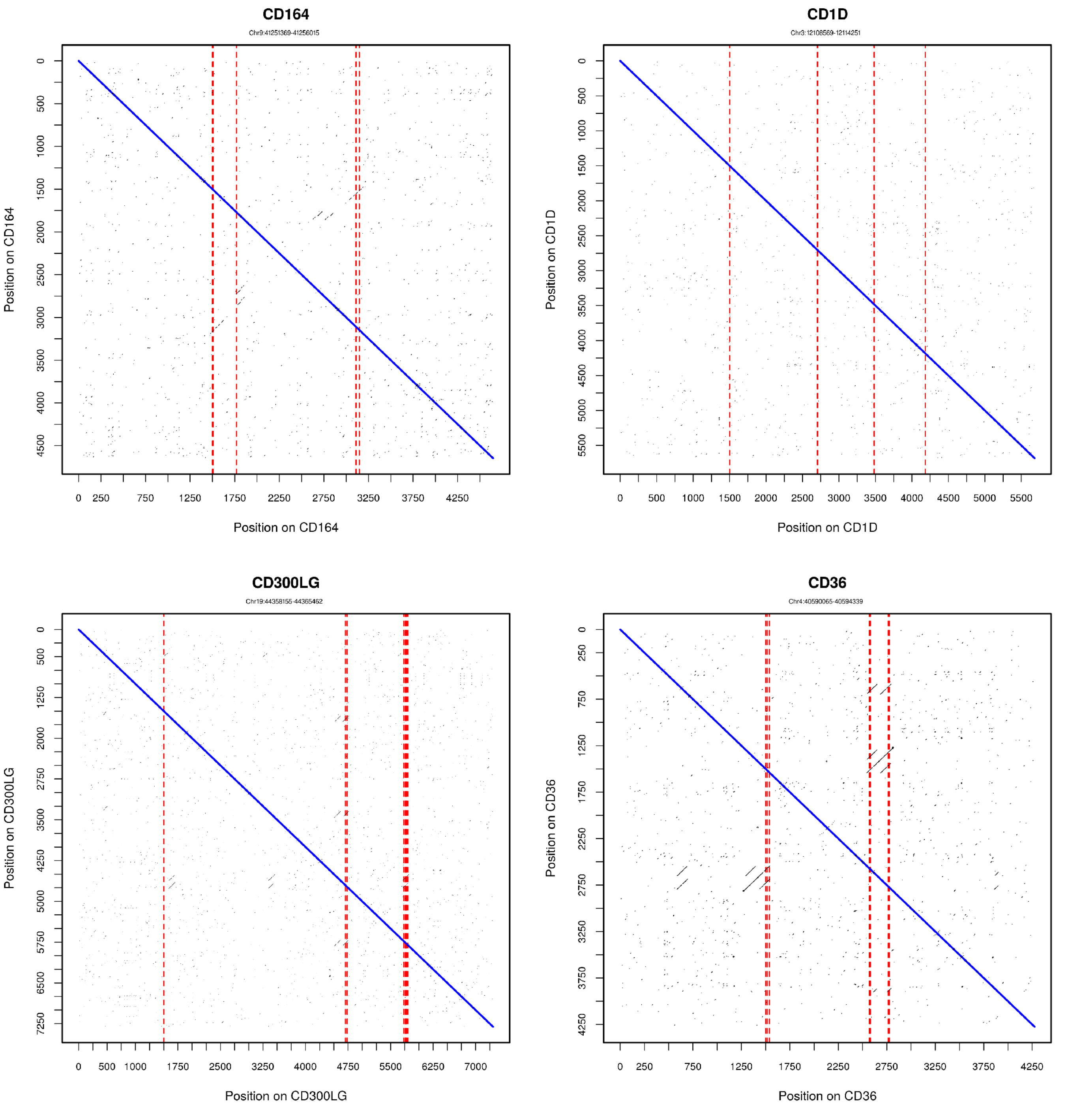
CD164
Chr9:41251369-41256015
Position on CD164
Position on CD164
CD1D
Chr3:12108569-12114251
Position on CD1D
Position on CD1D
CD300LG
Chr19:44358155-44365462
Position on CD300LG
Position on CD300LG
CD36
Chr4:40590065-40594339
Position on CD36
Position on CD36

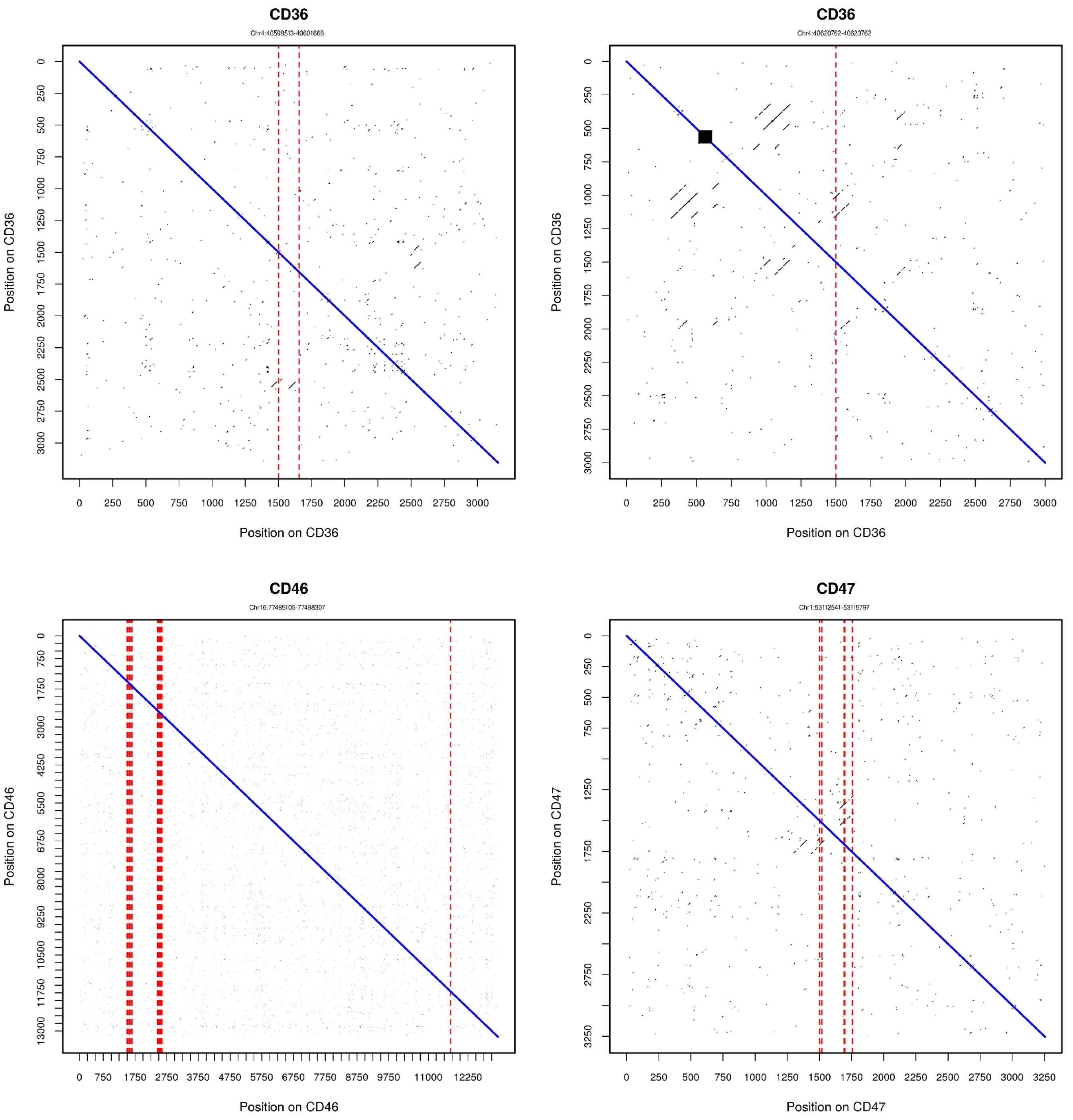
CD36
Chr4:40598513-40601668
Position on CD36
Position on CD36
CD36
Chr4:40620762-40623762
Position on CD36
Position on CD36
CD46
Chr16:77485105-77498307
Position on CD46
Position on CD46
CD47
Chr1:53112541-53115797
Position on CD47
Position on CD47

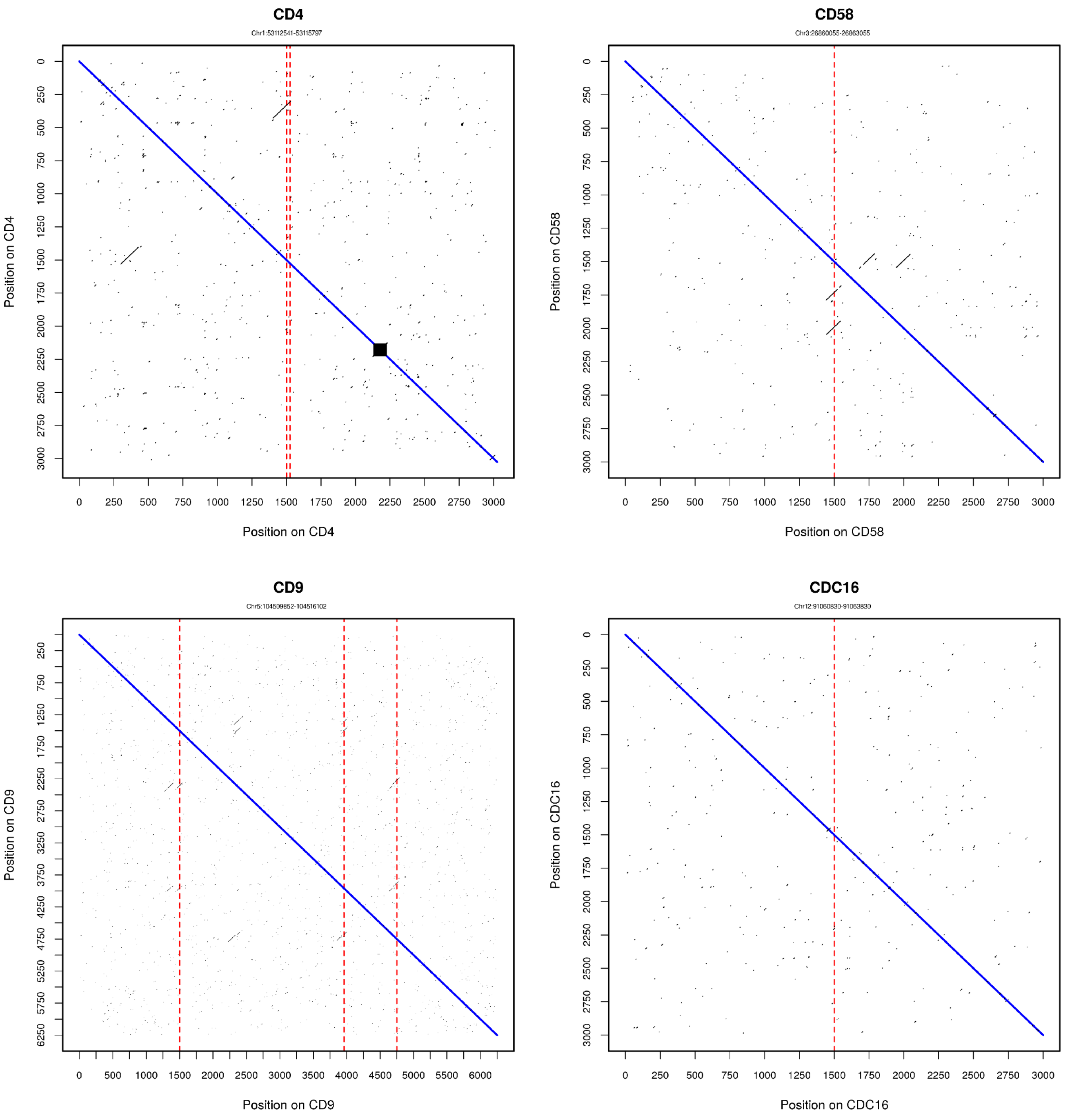
CD4
Chr1:53112541-53115797
Position on CD4
Position on CD4
CD58
Chr3:26860055-26863055
Position on CD58
Position on CD58
CD9
Chr5:104509852-104516102
Position on CD9
Position on CD9
CDC16
Chr12:91060830-91063830
Position on CDC16
Position on CDC16

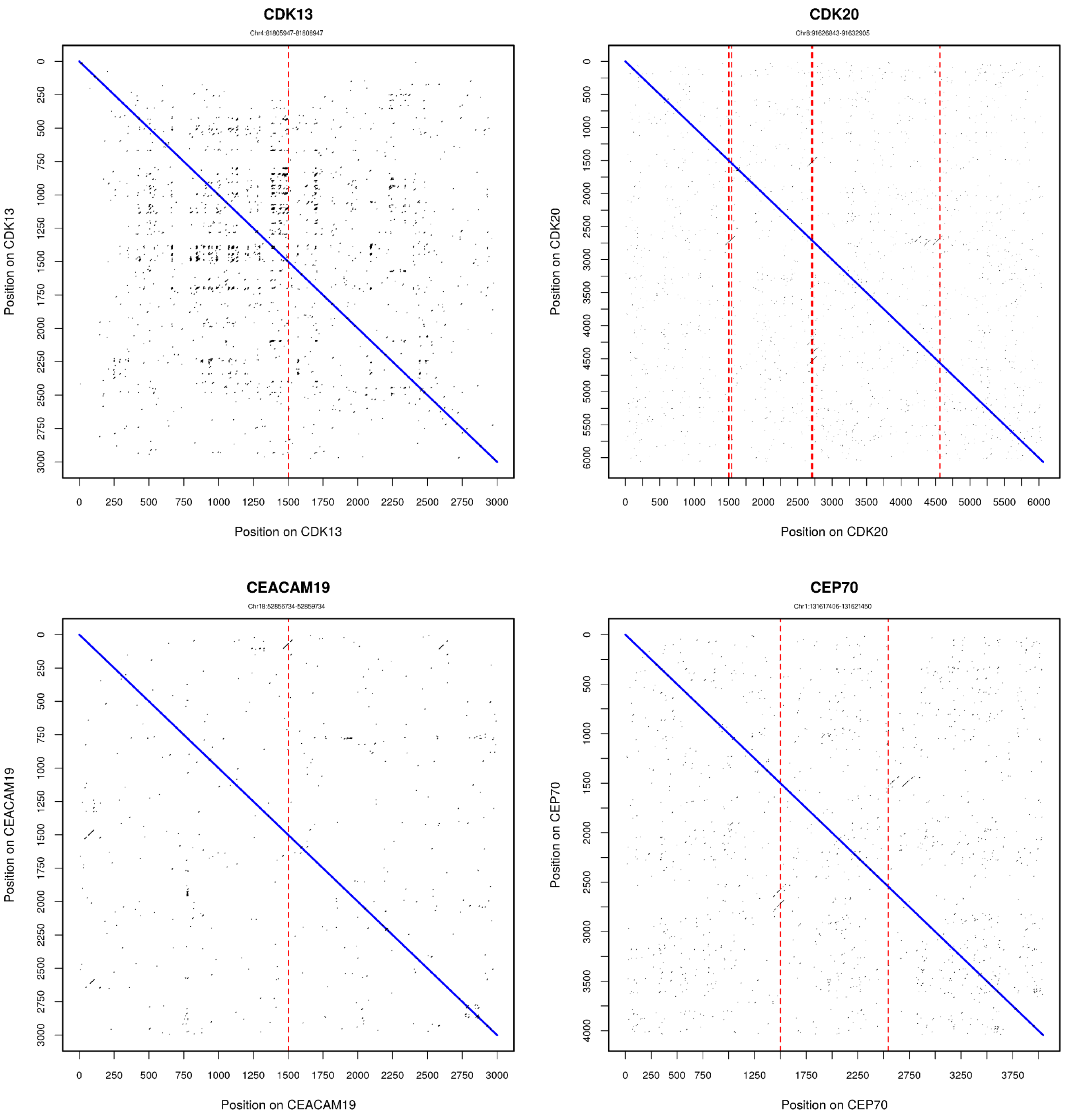

CDK13
Chr4:81805947-81808947
Position on CDK13
Position on CDK13
CDK20
Chr8:91626843-91632905
Position on CDK20
Position on CDK20
CEACAM19
Chr18:52856734-52859734
Position on CEACAM19
Position on CEACAM19
CEP70
Chr1:131617406-131621450
Position on CEP70
Position on CEP70

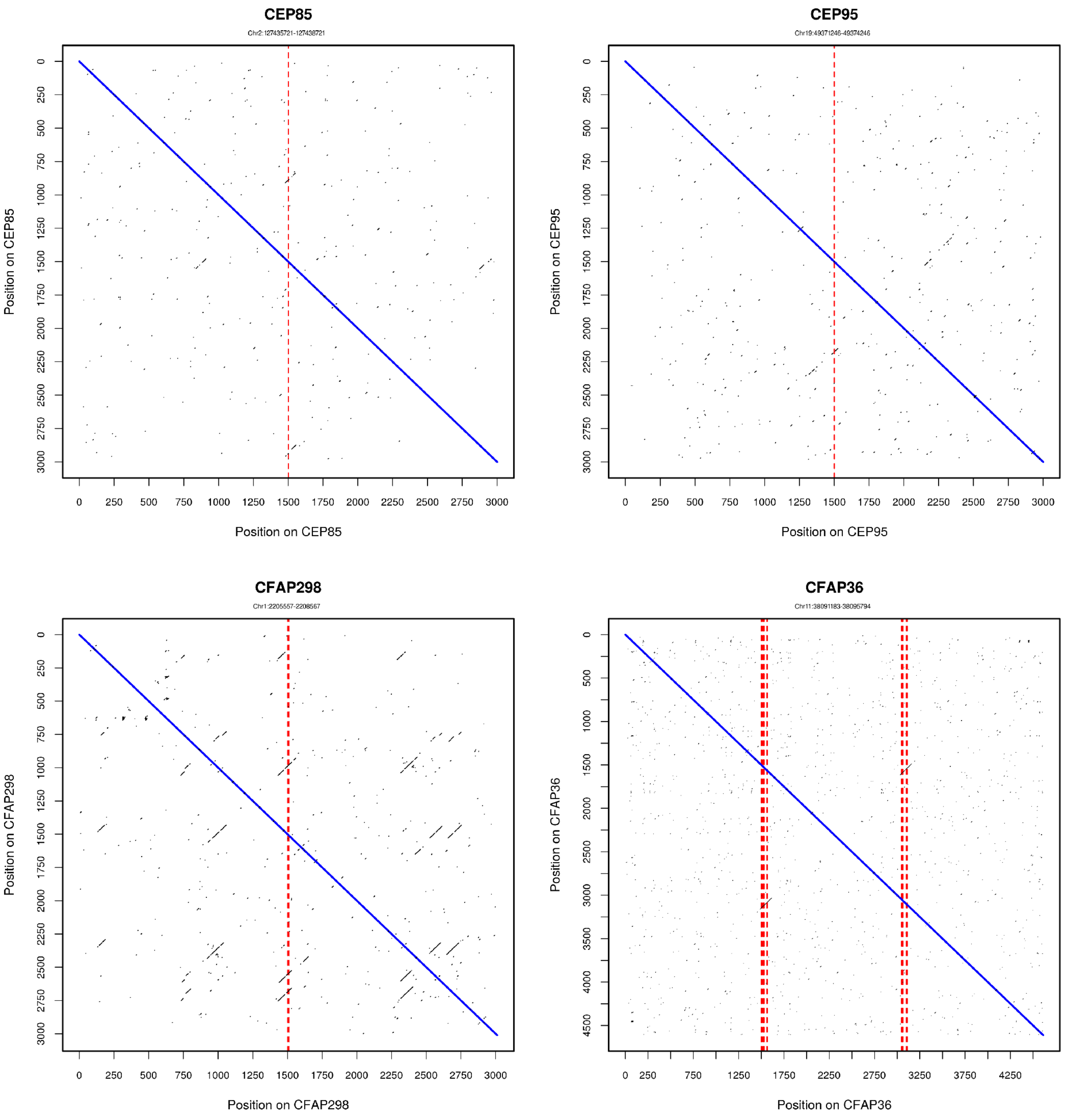
CEP85
Chr2:127435721-127438721
Position on CEP85
Position on CEP85
CEP95
Chr19:49371246-49374246
Position on CEP95
Position on CEP95
CFAP298
Chr1:2205557-2208567
Position on CFAP298
Position on CFAP298
CFAP36
Chr11:38091183-38095794
Position on CFAP36
Position on CFAP36

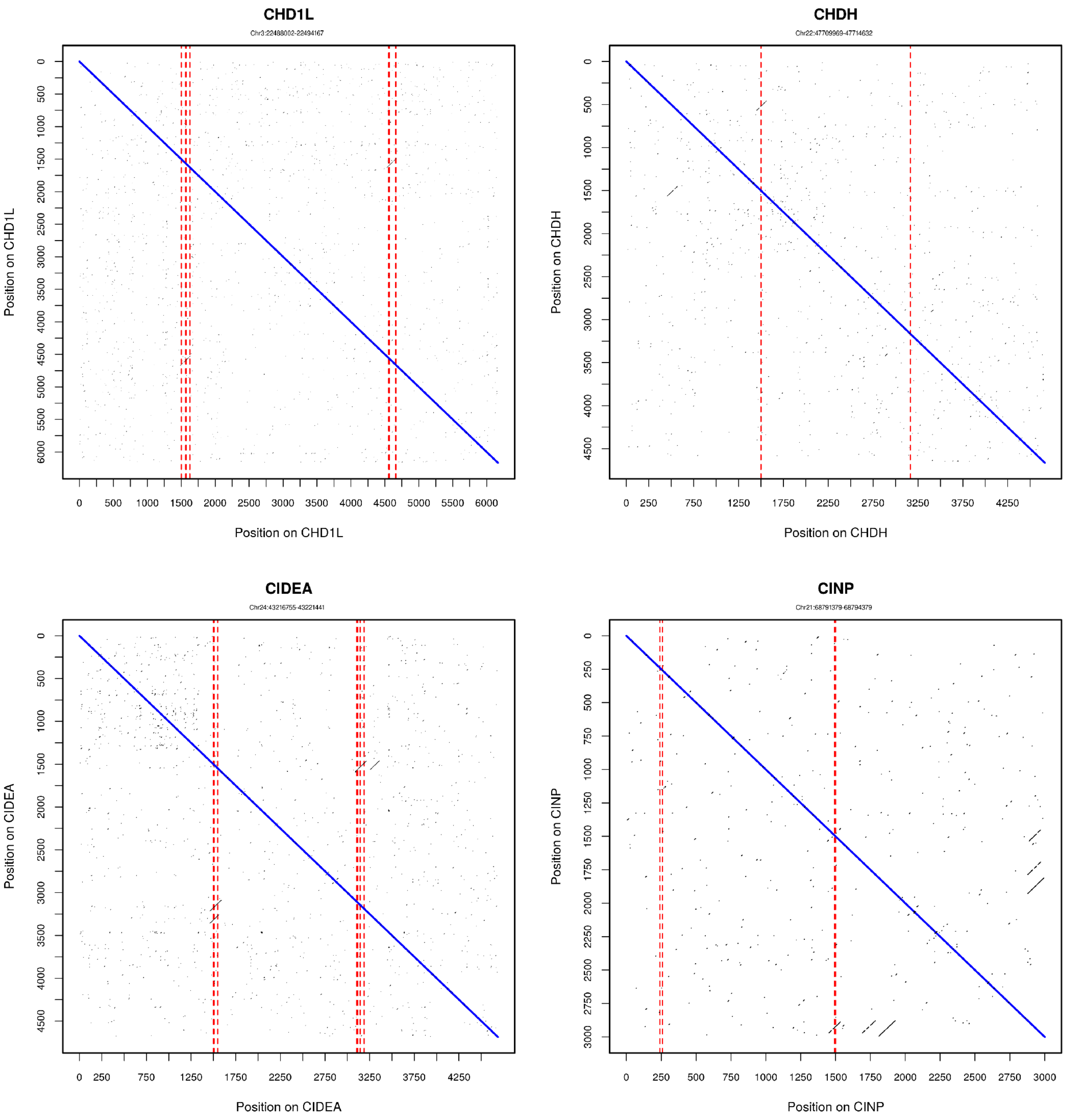
CHD1L
Chr3:22488002-22494167
Position on CHD1L
Position on CHD1L
CHDH
Chr22:47709969-47714632
Position on CHDH
Position on CHDH
CIDEA
Chr24:43216755-43221441
Position on CIDEA
Position on CIDEA
CINP
Chr21:68791379-68794379
Position on CINP
Position on CINP

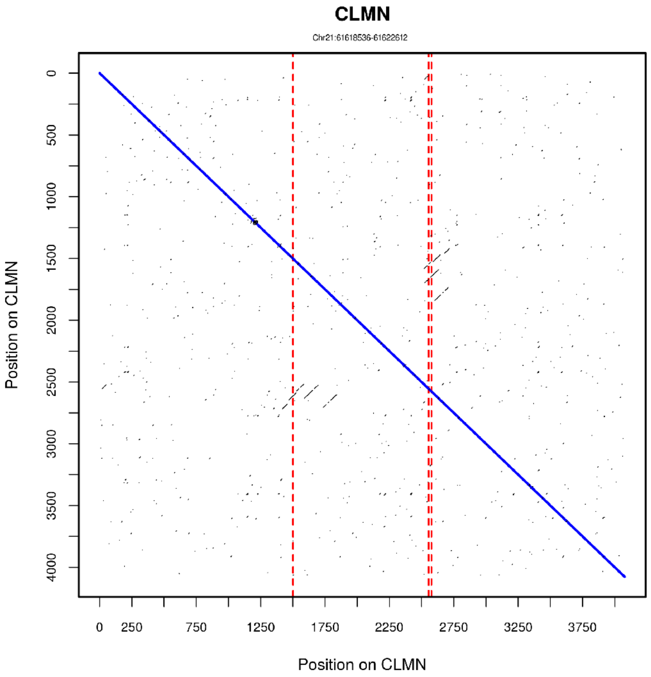
CLMN
Chr21:61618536-61622612
Position on CLMN
Position on CLMN
0
250
750
1250
1750
2250
2750
3250
3750
0
500
1000
1500
2000
2500
3000
3500
4000

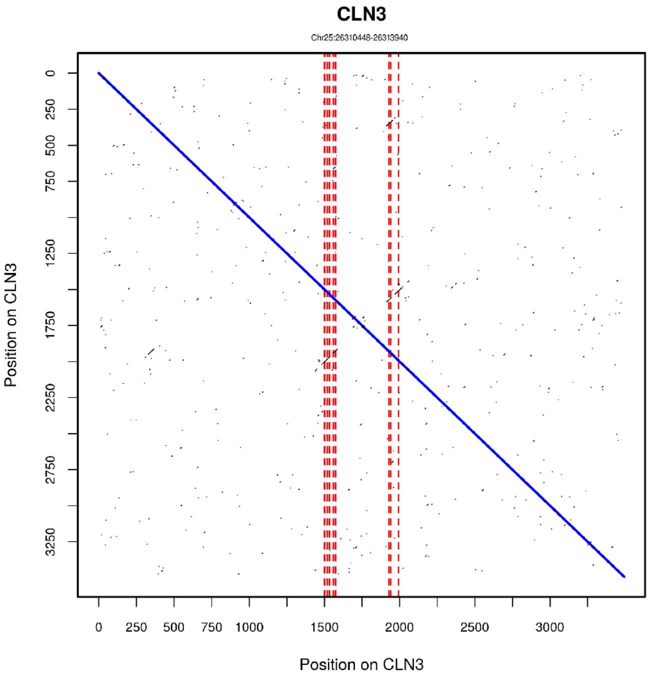
CLN3
Chr25:26310448-26313940
Position on CLN3
Position on CLN3
0
250
500
750
1000
1500
2000
2500
3000
0
250
500
750
1250
1750
2250
2750
3250

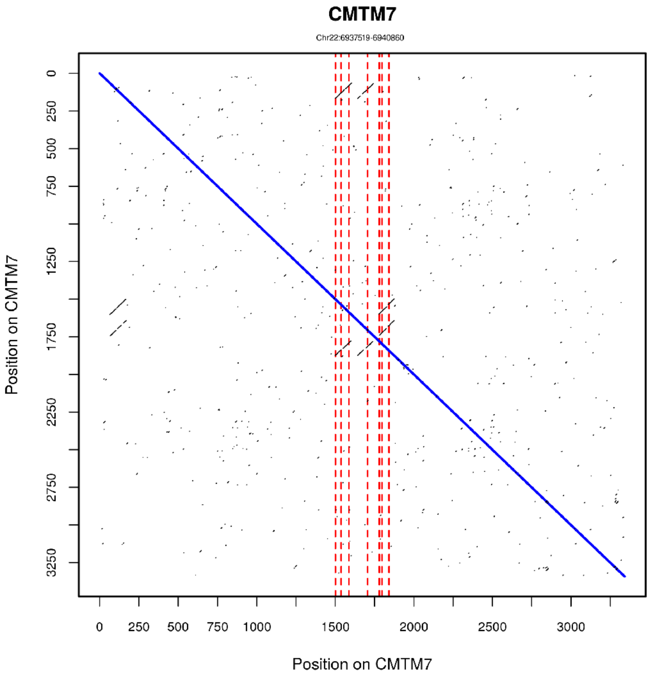
CMTM7
Chr22:6937519-6940860
Position on CMTM7
Position on CMTM7
0
250
500
750
1000
1500
2000
2500
3000
0
250
500
750
1250
1750
2250
2750
3250

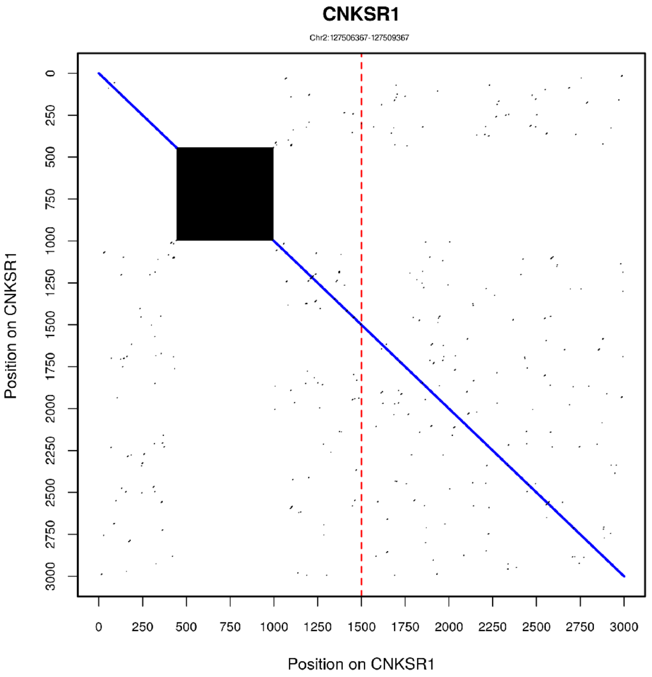
CNKSR1
Chr2:127506367-127509367
Position on CNKSR1
Position on CNKSR1
0
250
500
750
1000
1250
1500
1750
2000
2250
2500
2750
3000
0
250
500
750
1000
1250
1500
1750
2000
2250
2500
2750
3000

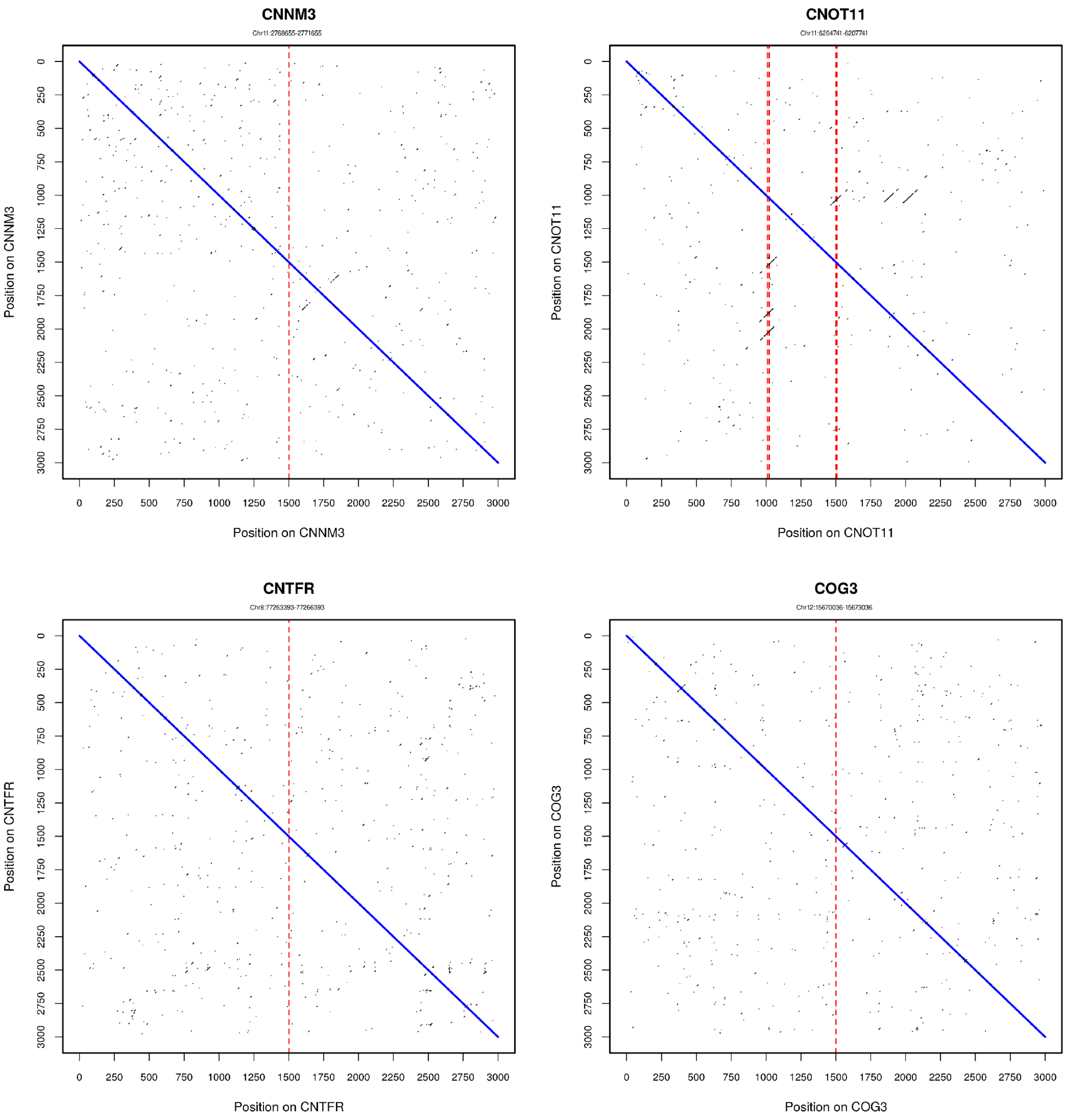
CNNM3
Chr11:2768655-2771655
Position on CNNM3
Position on CNNM3
CNOT11
Chr11:6204741-6207741
Position on CNOT11
Position on CNOT11
CNTFR
Chr8:77263393-77266393
Position on CNTFR
Position on CNTFR
COG3
Chr12:15670036-15673036
Position on COG3
Position on COG3
0
250
500
750
1000
1250
1500
1750
2000
2250
2500
2750
3000

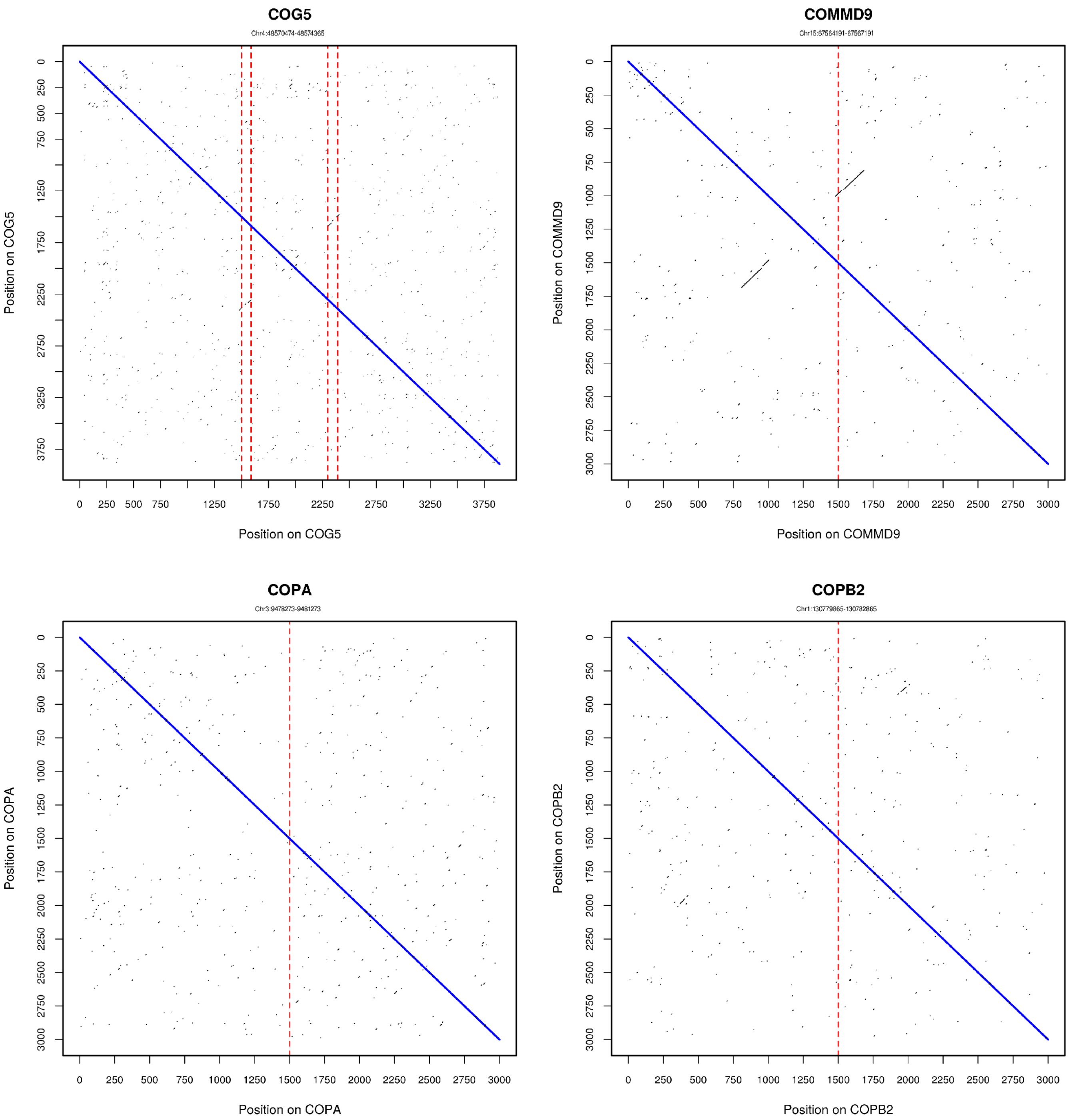
COG5
Chr4:48570474-48574365
Position on COG5
Position on COG5
COMMD9
Chr15:67564191-67567191
Position on COMMD9
Position on COMMD9
COPA
Chr3:9478273-9481273
Position on COPA
Position on COPA
COPB2
Chr1:130779865-130782865
Position on COPB2
Position on COPB2

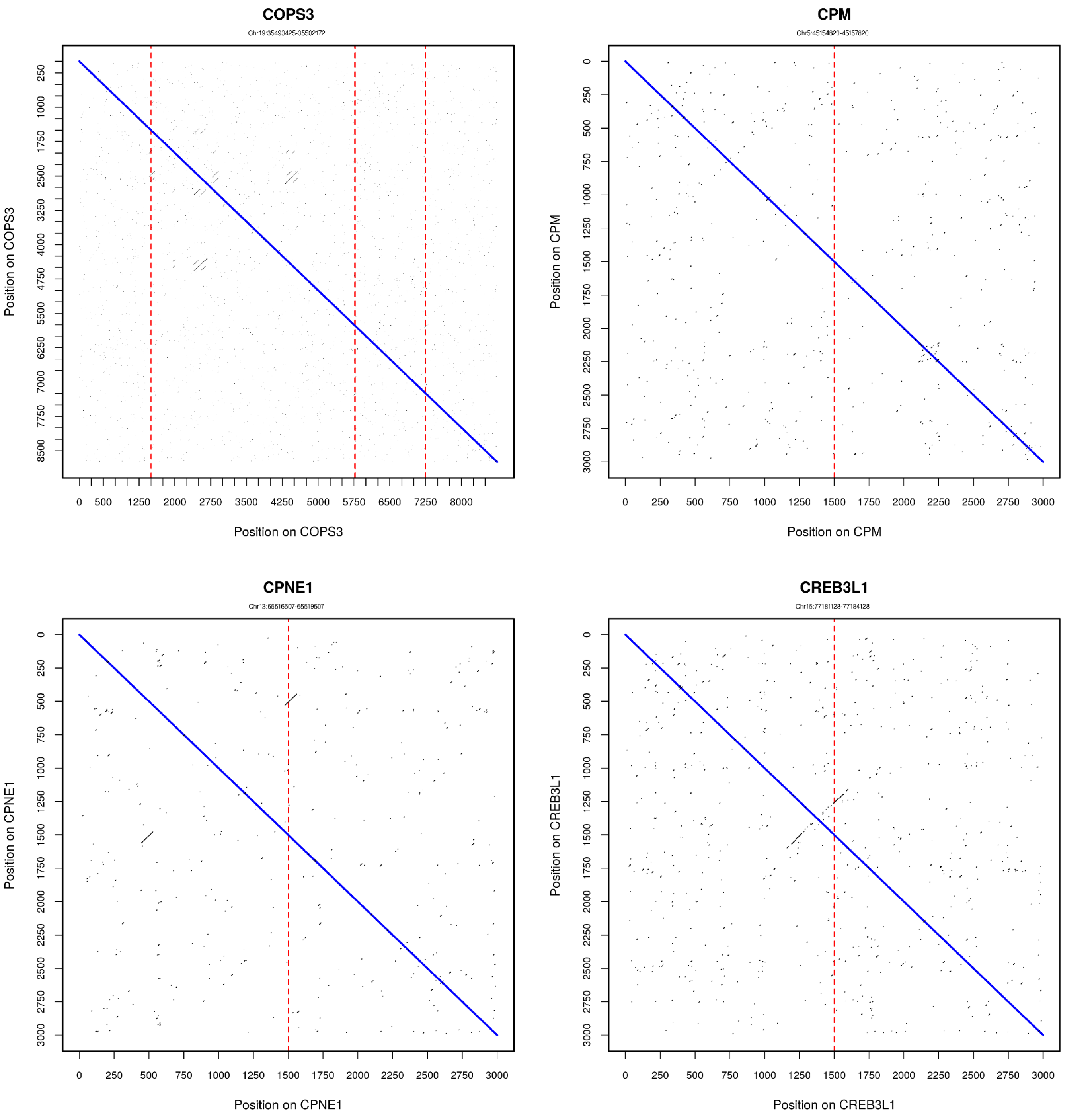

COPS3
Chr19:35493425-35502172
Position on COPS3
Position on COPS3
CPM
Chr5:45154820-45157820
Position on CPM
Position on CPM
CPNE1
Chr13:65516507-65519507
Position on CPNE1
Position on CPNE1
CREB3L1
Chr15:77181128-77184128
Position on CREB3L1
Position on CREB3L1

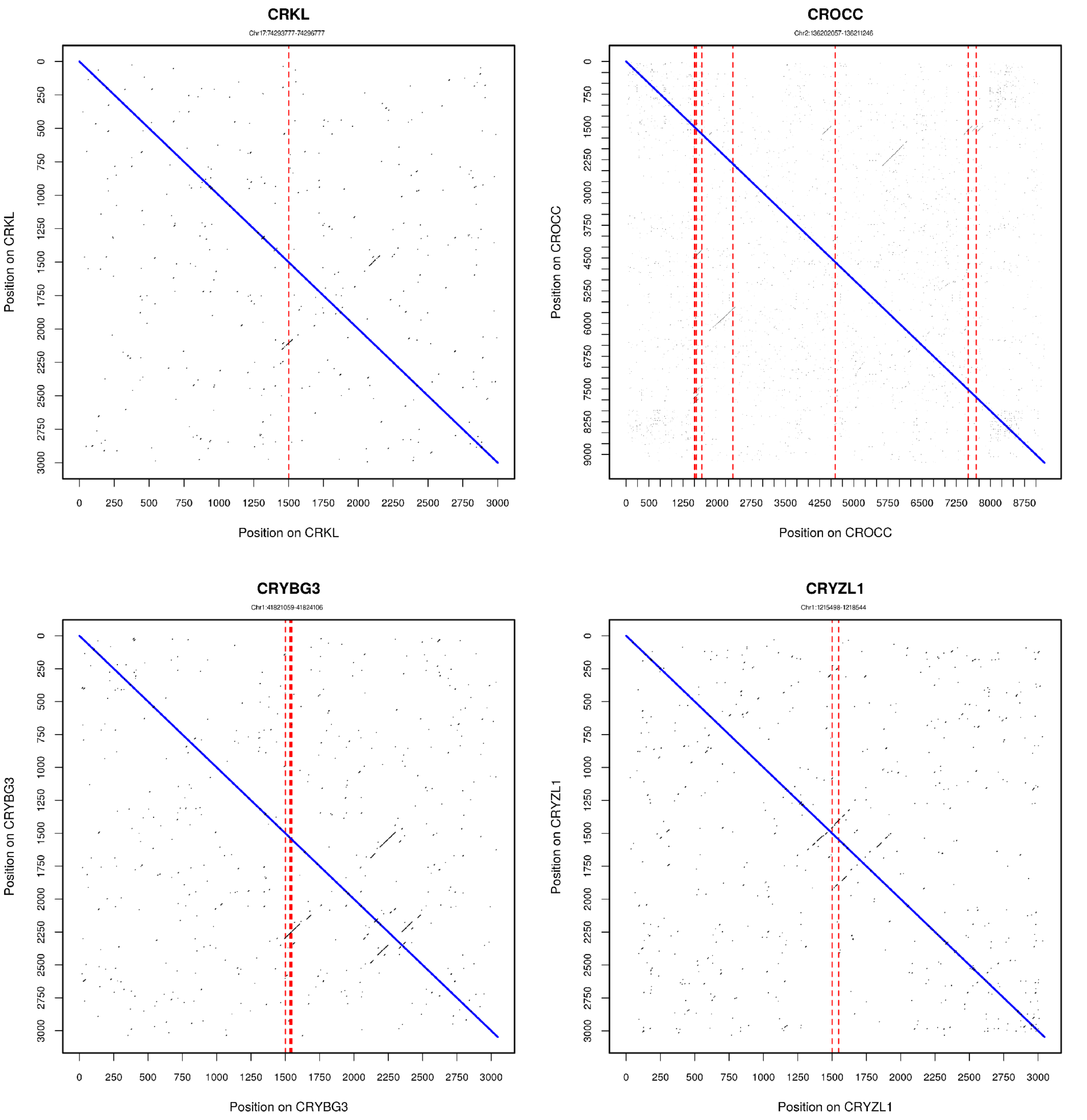
CRKL
Chr17:74293777-74296777
Position on CRKL
Position on CRKL
CROCC
Chr2:136202057-136211246
Position on CROCC
Position on CROCC
CRYBG3
Chr1:41821059-41824106
Position on CRYBG3
Position on CRYBG3
CRYZL1
Chr1:1215498-1218544
Position on CRYZL1
Position on CRYZL1

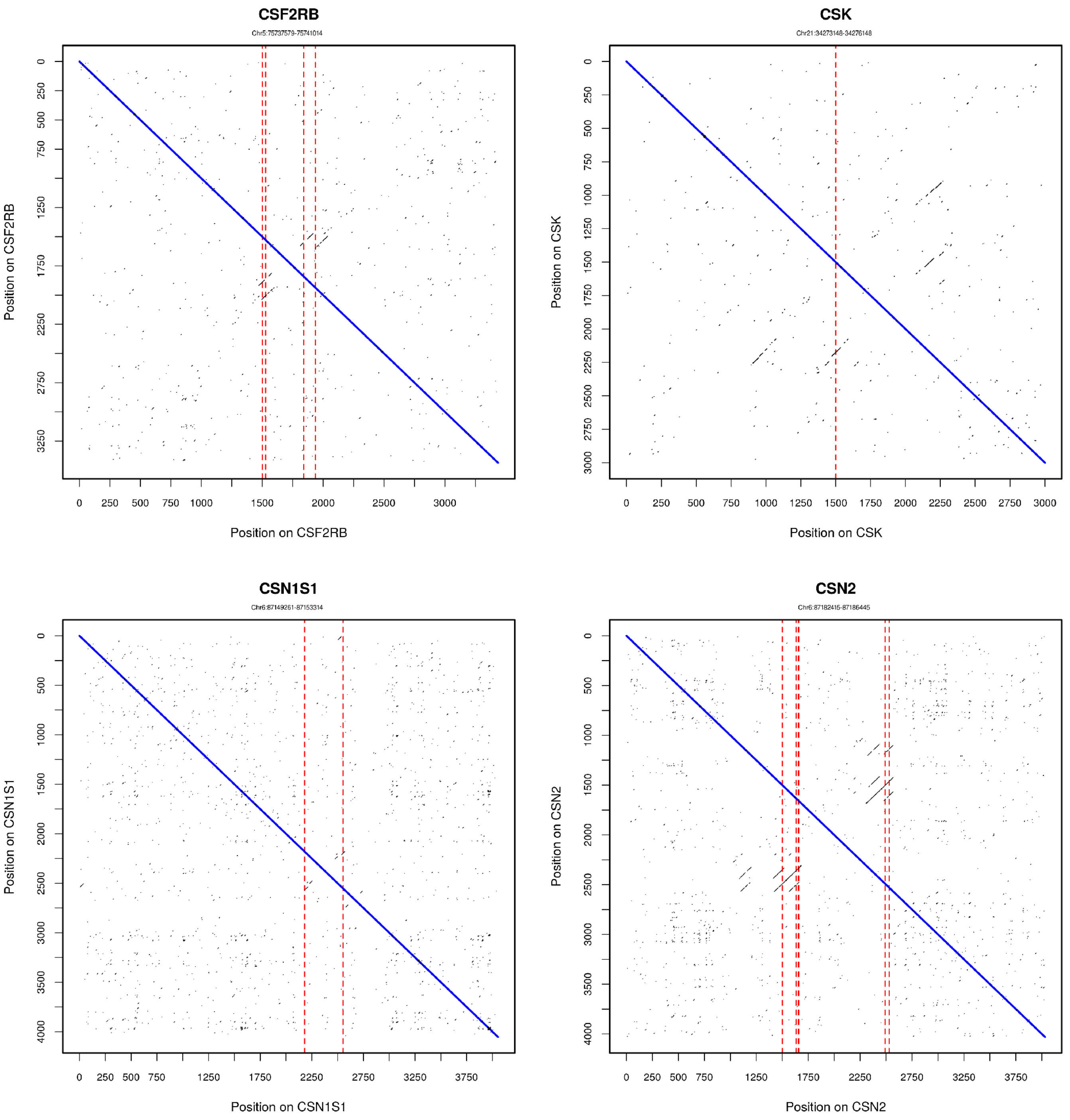
CSF2RB
Chr5:75737579-75741014
Position on CSF2RB
Position on CSF2RB
CSK
Chr21:34273148-34276148
Position on CSK
Position on CSK
CSN1S1
Chr6:87149261-87153314
Position on CSN1S1
Position on CSN1S1
CSN2
Chr6:87182415-87186445
Position on CSN2
Position on CSN2

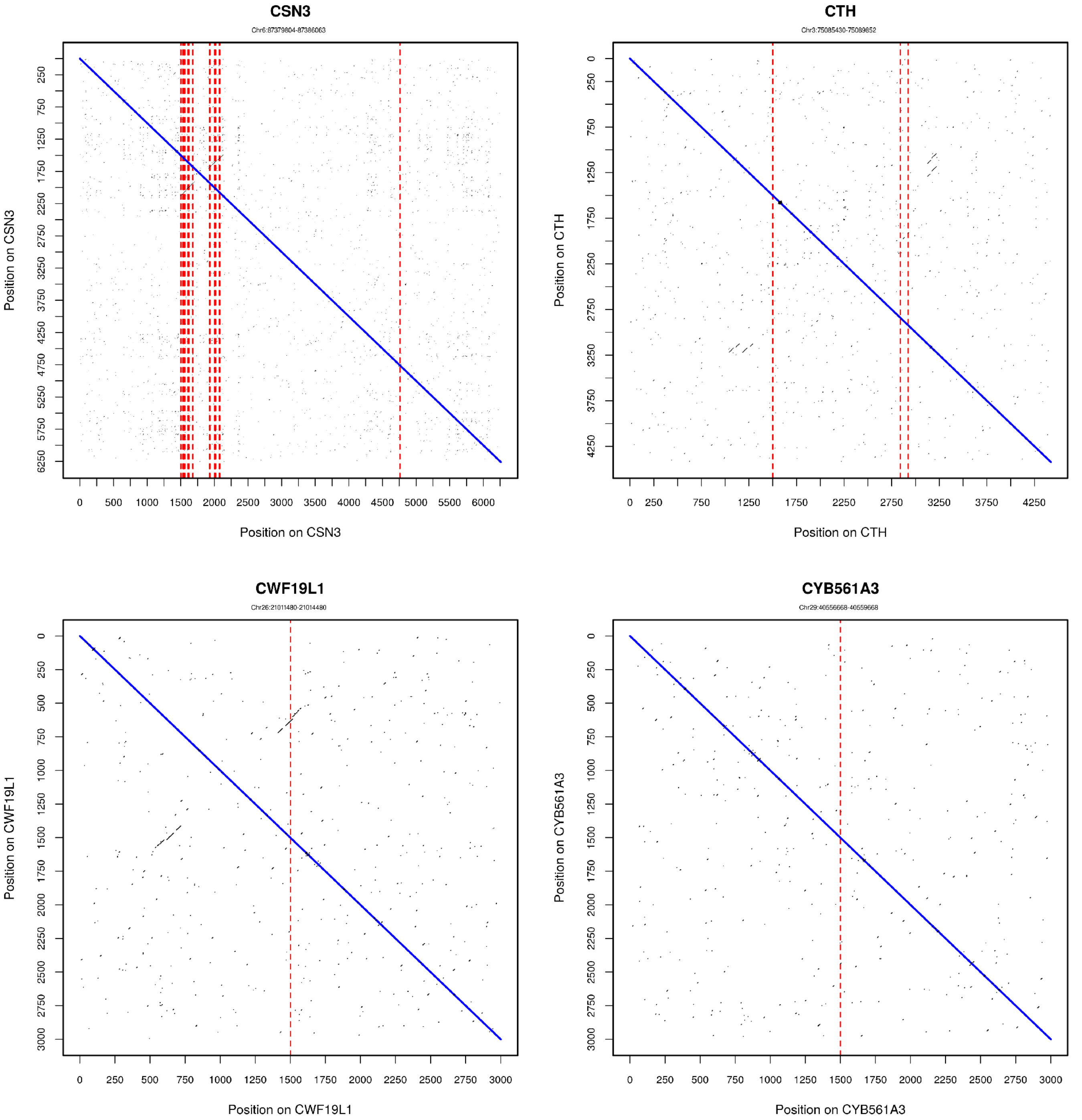

CSN3
Chr6:87379804-87386063
Position on CSN3
Position on CSN3
CTH
Chr3:75085430-75089852
Position on CTH
Position on CTH
CWF19L1
Chr26:21011480-21014480
Position on CWF19L1
Position on CWF19L1
CYB561A3
Chr29:40556668-40559668
Position on CYB561A3
Position on CYB561A3

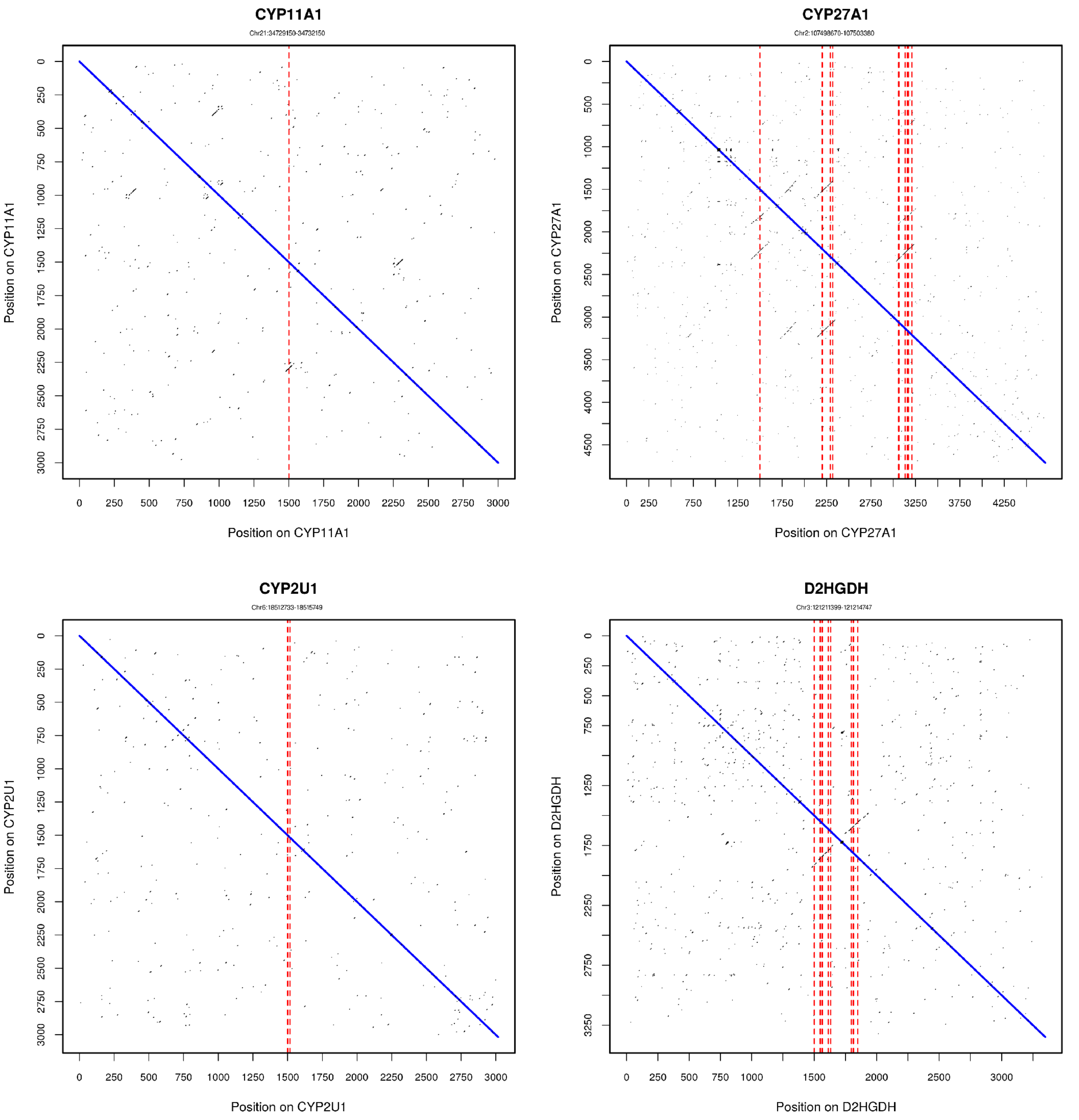

CYP11A1
Chr21:34729150-34732150
Position on CYP11A1
Position on CYP11A1
CYP27A1
Chr2:107498670-107503380
Position on CYP27A1
Position on CYP27A1
CYP2U1
Chr6:18512733-18515749
Position on CYP2U1
Position on CYP2U1
D2HGDH
Chr3:121211399-121214747
Position on D2HGDH
Position on D2HGDH

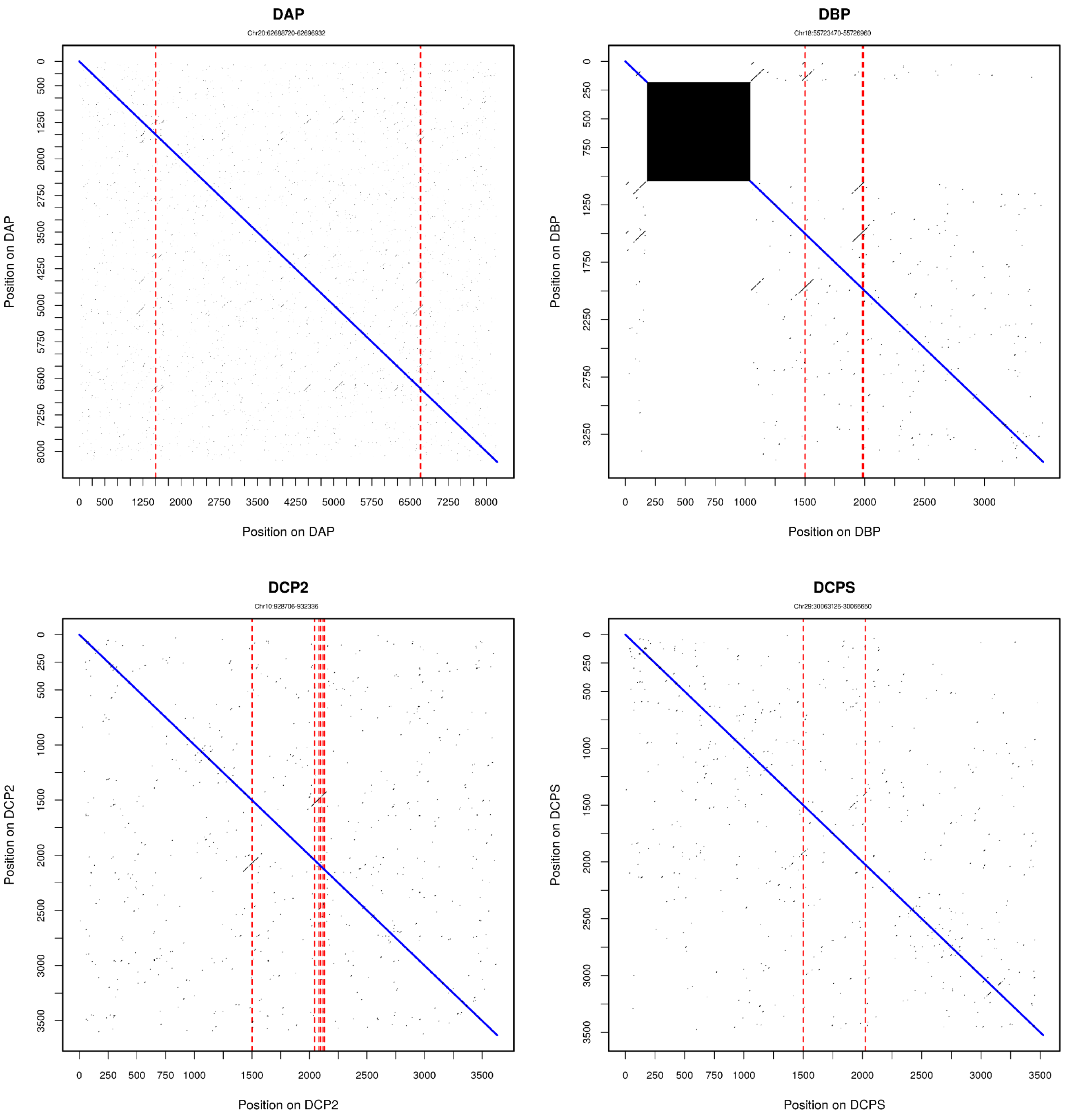
DAP
Chr20:62688720-62696932
Position on DAP
Position on DAP
DBP
Chr18:55723470-55726960
Position on DBP
Position on DBP
DCP2
Chr10:928706-932336
Position on DCP2
Position on DCP2
DCPS
Chr29:30063126-30066650
Position on DCPS
Position on DCPS

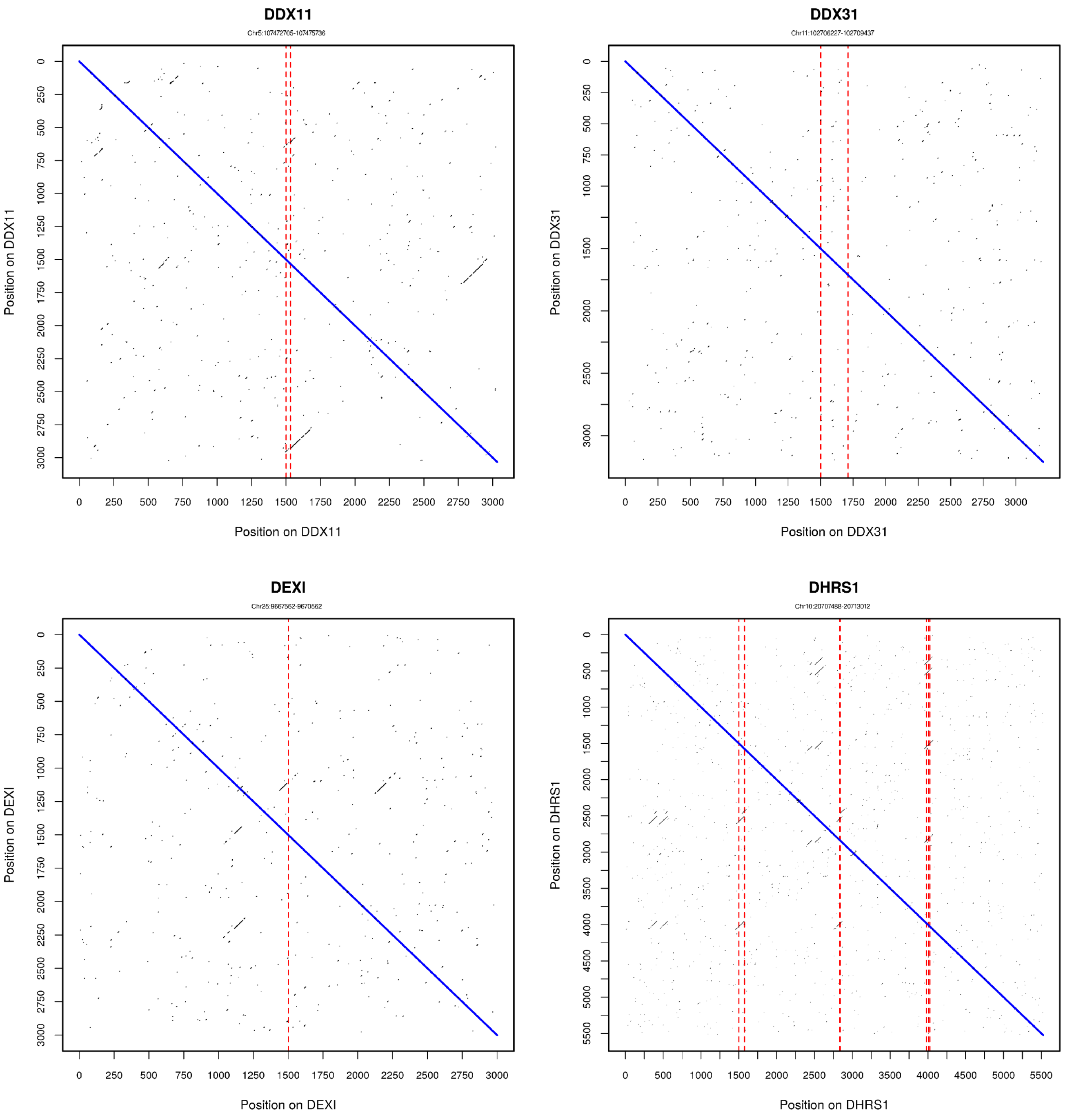
DDX11
Chr5:107472705-107475736
Position on DDX11
Position on DDX11
DDX31
Chr11:102706227-102709437
Position on DDX31
Position on DDX31
DEXI
Chr25:9667562-9670562
Position on DEXI
Position on DEXI
DHRS1
Chr10:20707488-20713012
Position on DHRS1
Position on DHRS1

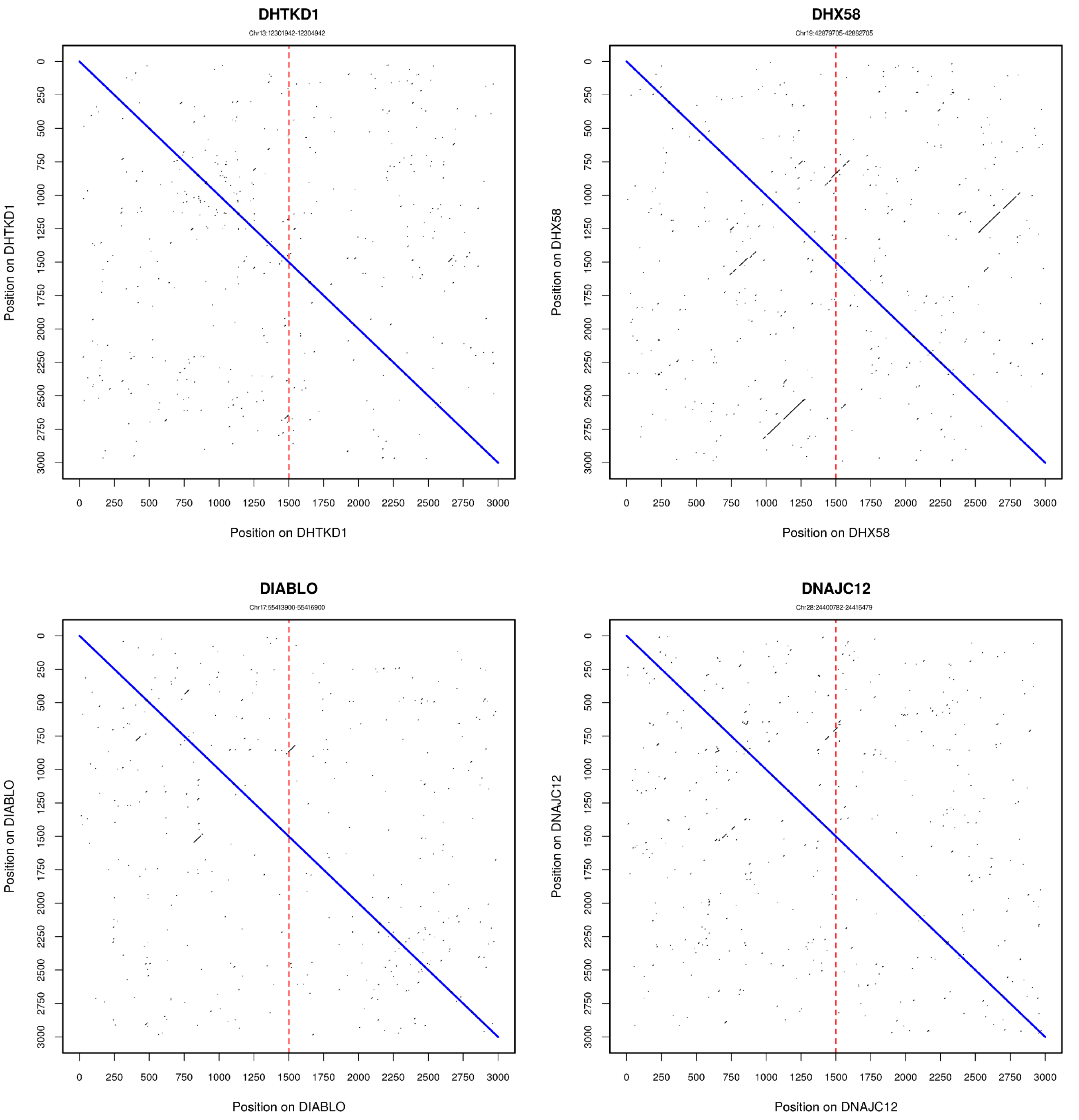
DHTKD1
Chr13:12301942-12304942
Position on DHTKD1
Position on DHTKD1
DHX58
Chr19:42879705-42882705
Position on DHX58
Position on DHX58
DIABLO
Chr17:55413900-55416900
Position on DIABLO
Position on DIABLO
DNAJC12
Chr28:24400782-24416479
Position on DNAJC12
Position on DNAJC12

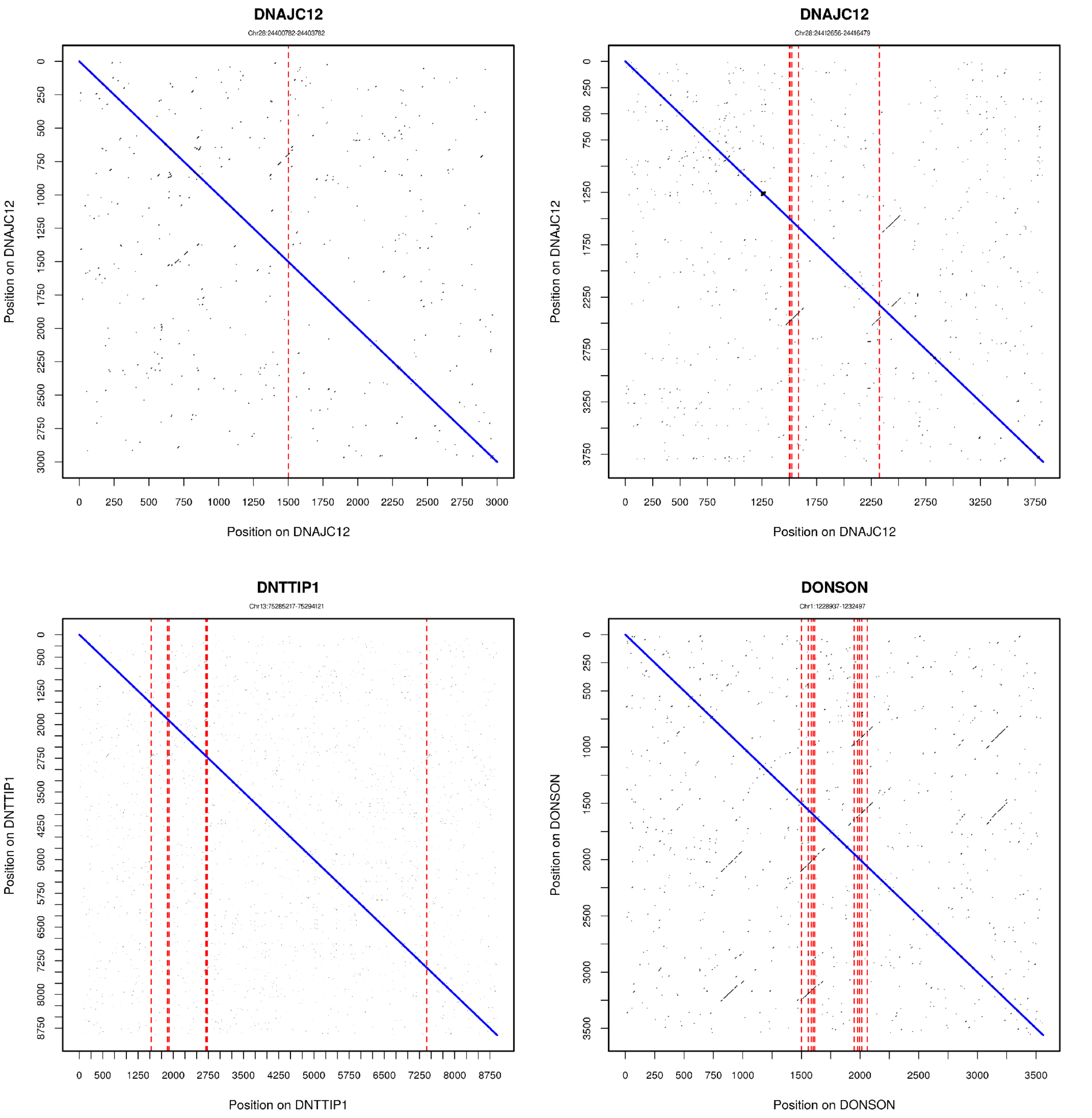

DNAJC12
Chr28:24400782-24403782
Position on DNAJC12
Position on DNAJC12
DNAJC12
Chr28:24412656-24416479
Position on DNAJC12
Position on DNAJC12
DNTTIP1
Chr13:75285217-75294121
Position on DNTTIP1
Position on DNTTIP1
DONSON
Chr1:1228937-1232497
Position on DONSON
Position on DONSON

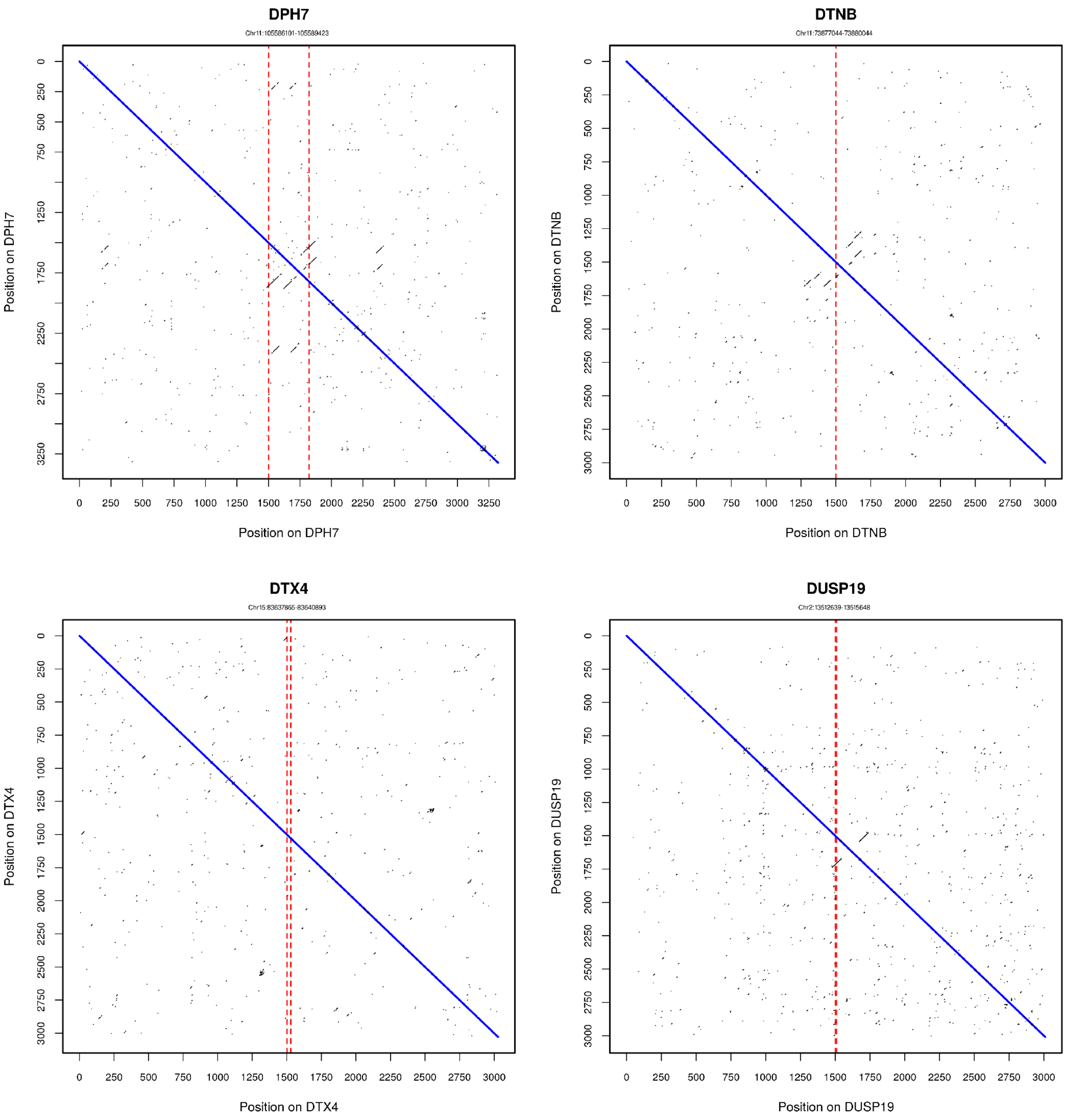
DPH7
Chr11:105586101-105589423
Position on DPH7
Position on DPH7
DTNB
Chr11:73877044-73880044
Position on DTNB
Position on DTNB
DTX4
Chr15:83637865-83640893
Position on DTX4
Position on DTX4
DUSP19
Chr2:13512639-13515648
Position on DUSP19
Position on DUSP19

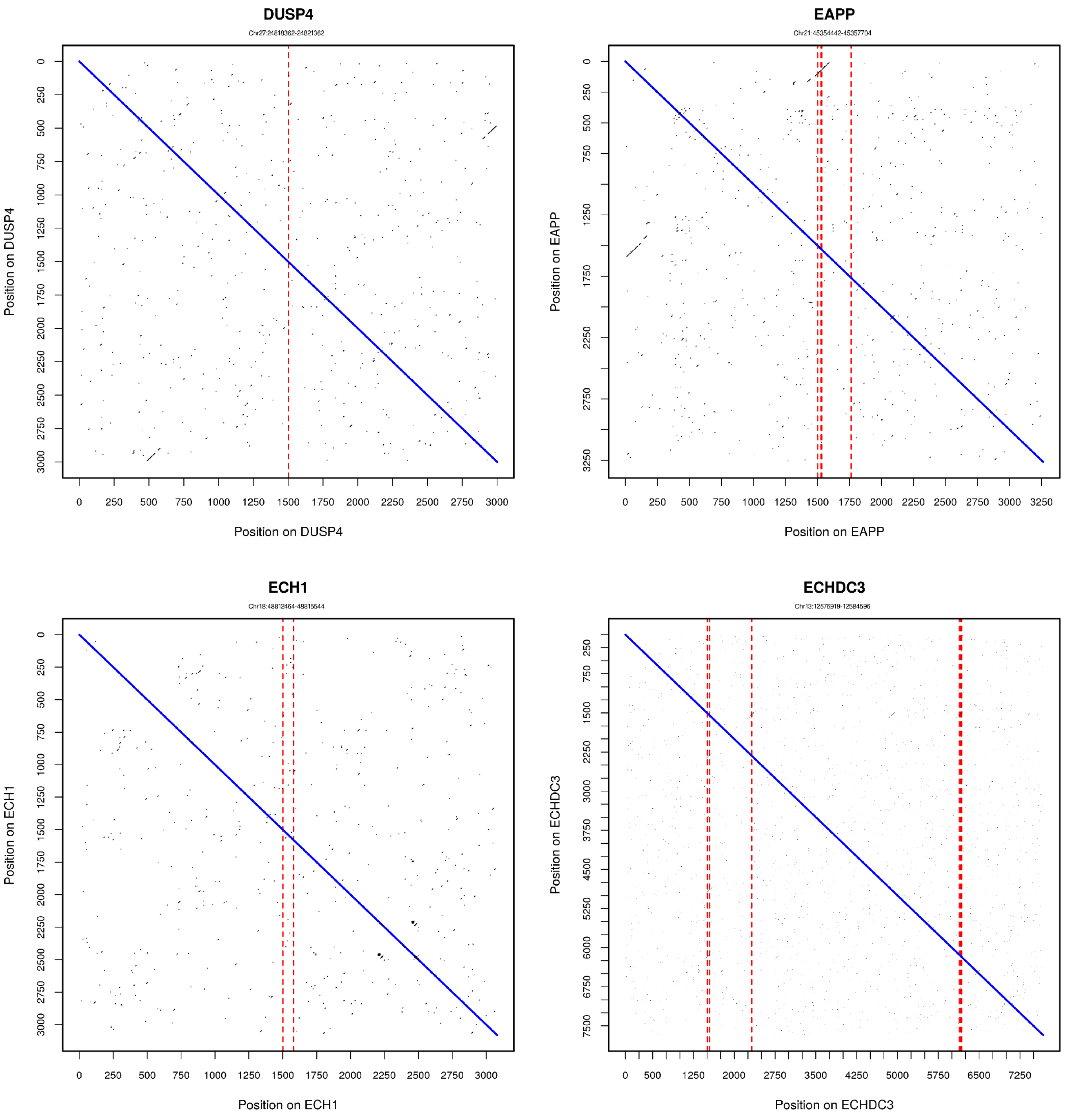
DUSP4
Chr27:24818362-24821362
Position on DUSP4
Position on DUSP4
EAPP
Chr21:45354442-45357704
Position on EAPP
Position on EAPP
ECH1
Chr18:48812464-48815544
Position on ECH1
Position on ECH1
ECHDC3
Chr13:12576919-12584596
Position on ECHDC3
Position on ECHDC3

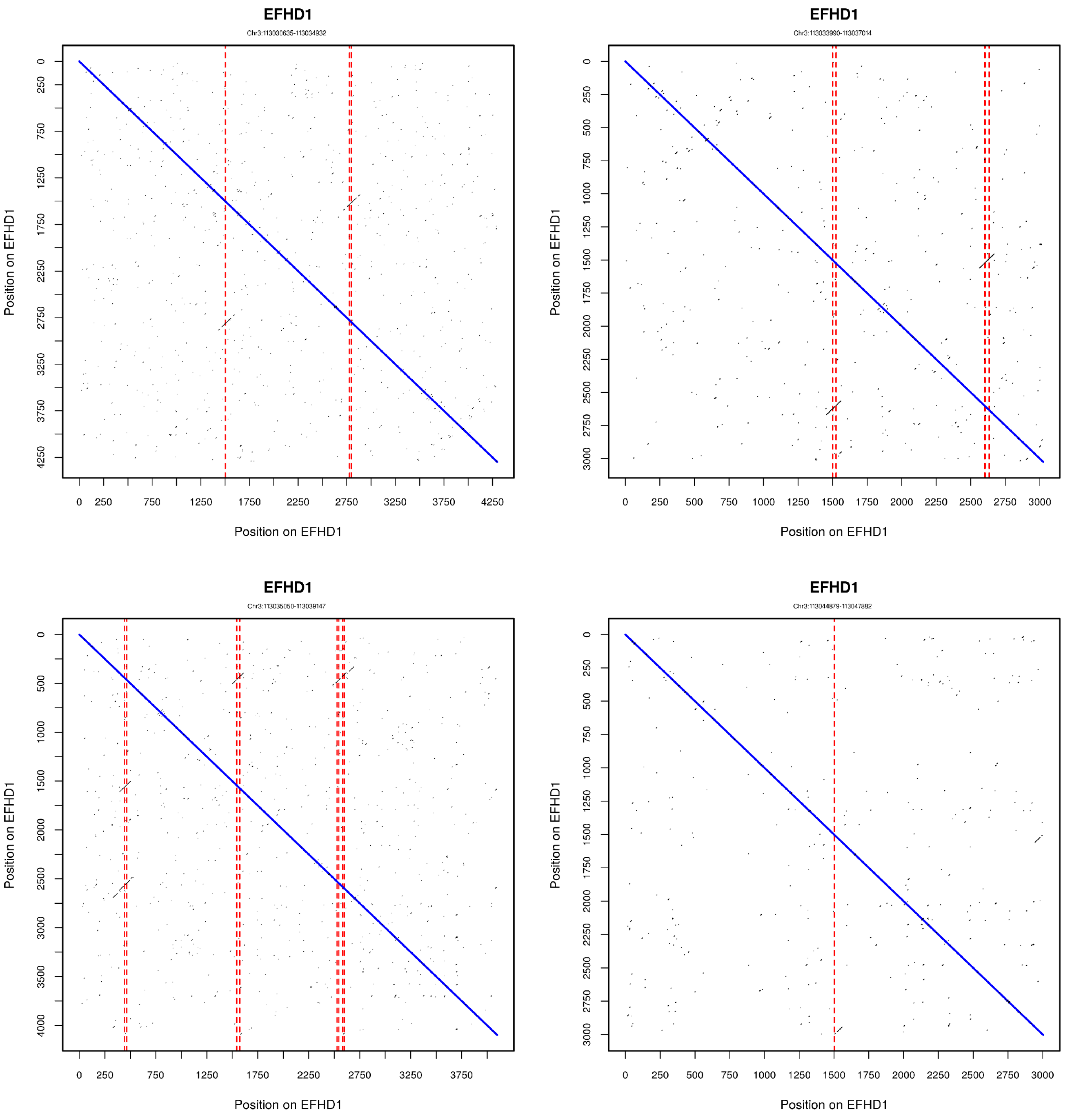

EFHD1
Chr3:113030635-113034932
Position on EFHD1
Position on EFHD1
EFHD1
Chr3:113033990-113037014
Position on EFHD1
Position on EFHD1
EFHD1
Chr3:113035050-113039147
Position on EFHD1
Position on EFHD1
EFHD1
Chr3:113044879-113047882
Position on EFHD1
Position on EFHD1

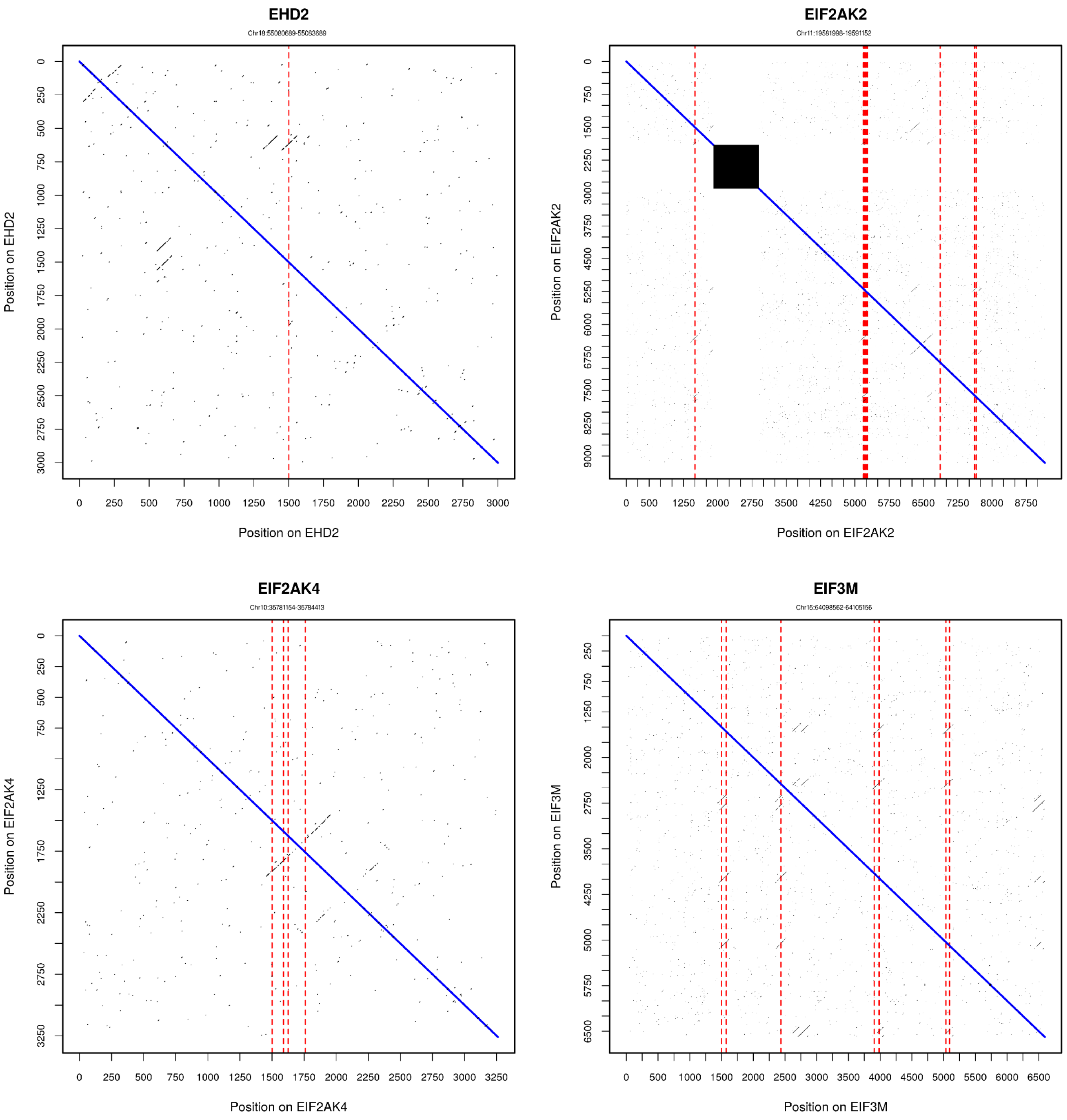
EHD2
Chr18:55080689-55083689
Position on EHD2
Position on EHD2
EIF2AK2
Chr11:19581998-19591152
Position on EIF2AK2
Position on EIF2AK2
EIF2AK4
Chr10:35781154-35784413
Position on EIF2AK4
Position on EIF2AK4
EIF3M
Chr15:64098562-64105156
Position on EIF3M
Position on EIF3M

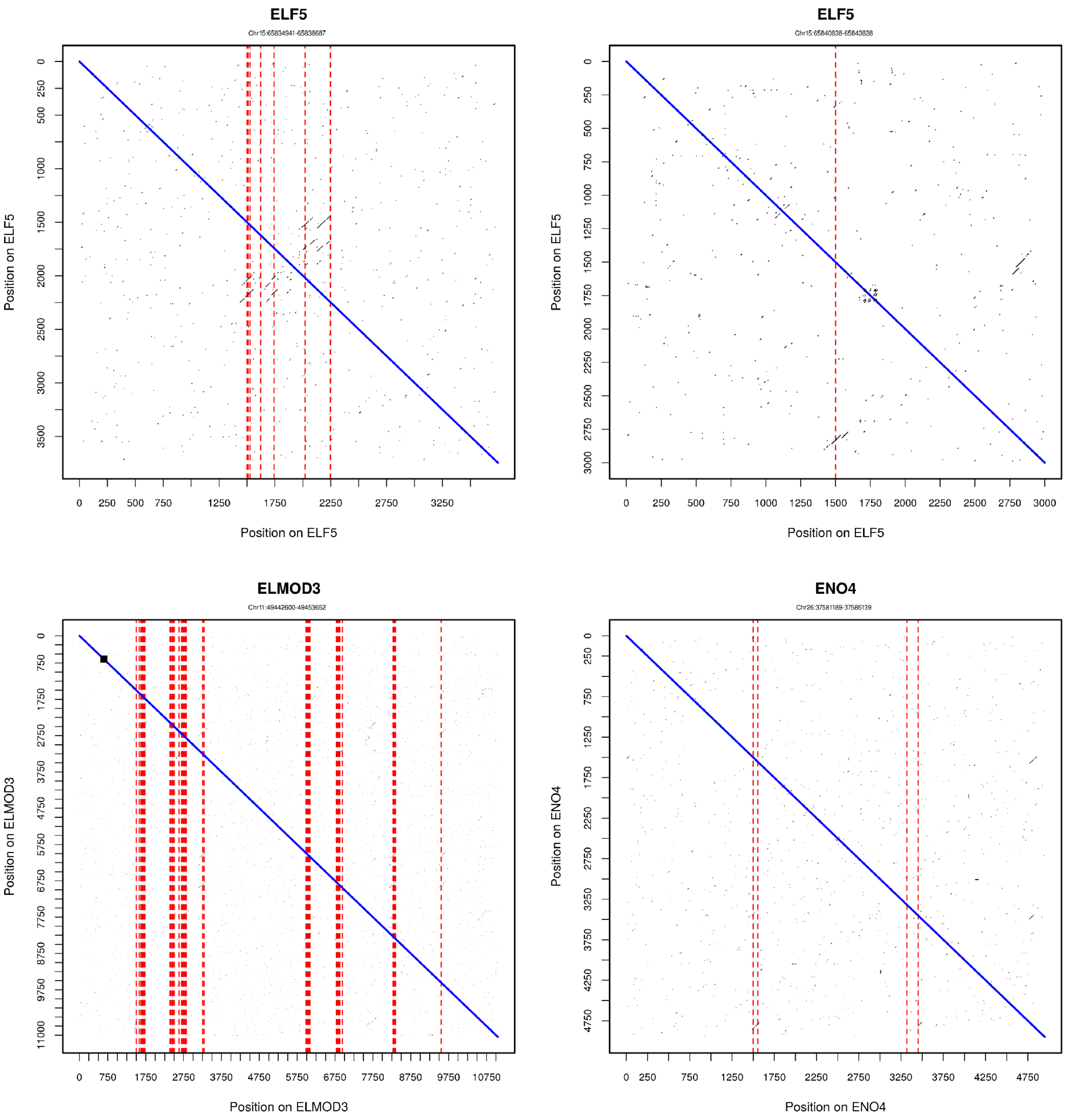
ELF5
Chr15:65834941-65838687
Position on ELF5
Position on ELF5
ELF5
Chr15:65840838-65843838
Position on ELF5
Position on ELF5
ELMOD3
Chr11:49442600-49453652
Position on ELMOD3
Position on ELMOD3
ENO4
Chr26:37581189-37586139
Position on ENO4
Position on ENO4

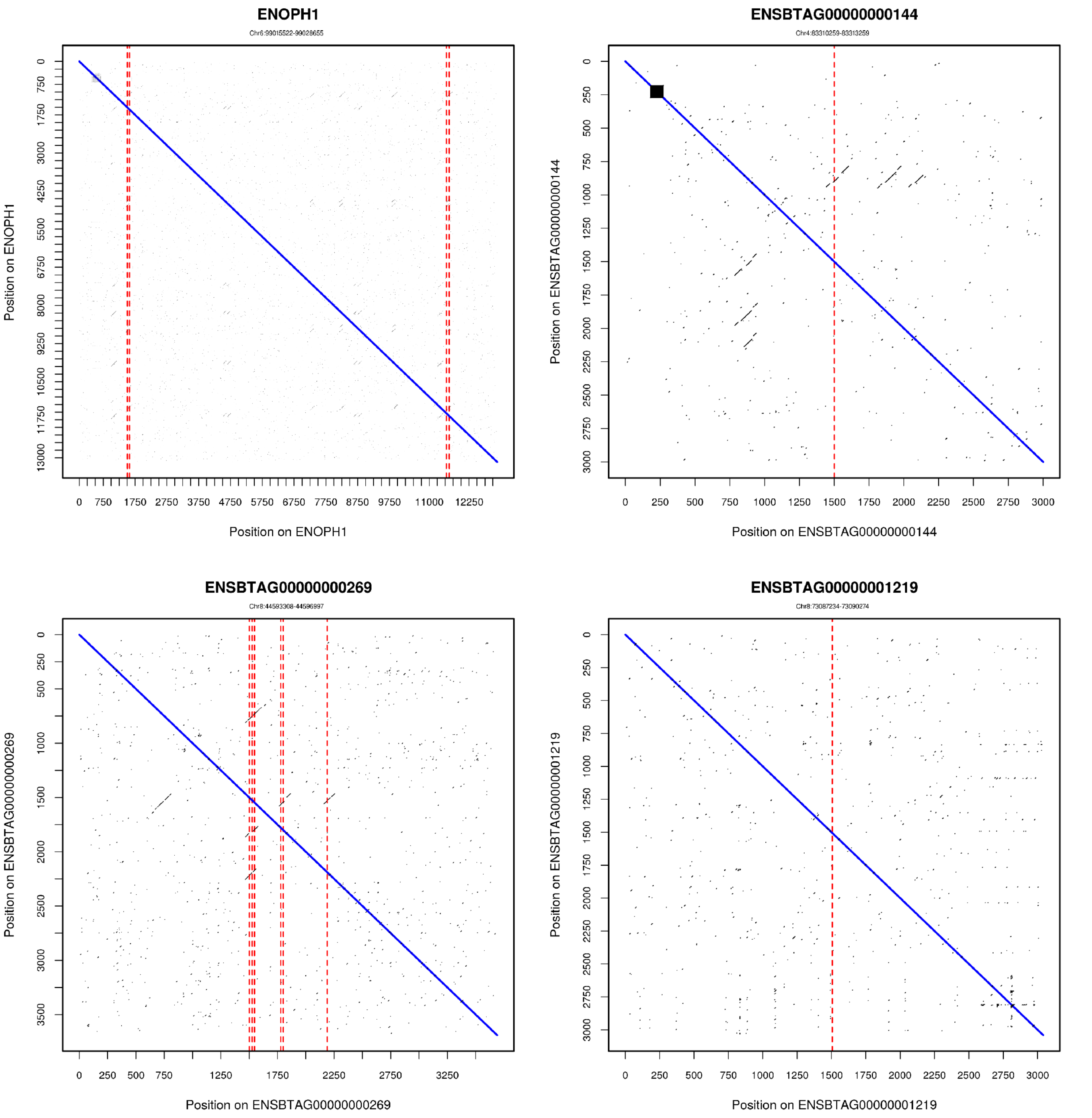
ENOPH1
Chr6:99015522-99028655
Position on ENOPH1
Position on ENOPH1
ENSBTAG00000000144
Chr4:83310259-83313259
Position on ENSBTAG00000000144
Position on ENSBTAG00000000144
ENSBTAG00000000269
Chr8:44593308-44596997
Position on ENSBTAG00000000269
Position on ENSBTAG00000000269
ENSBTAG00000001219
Chr8:73087234-73090274
Position on ENSBTAG00000001219
Position on ENSBTAG00000001219

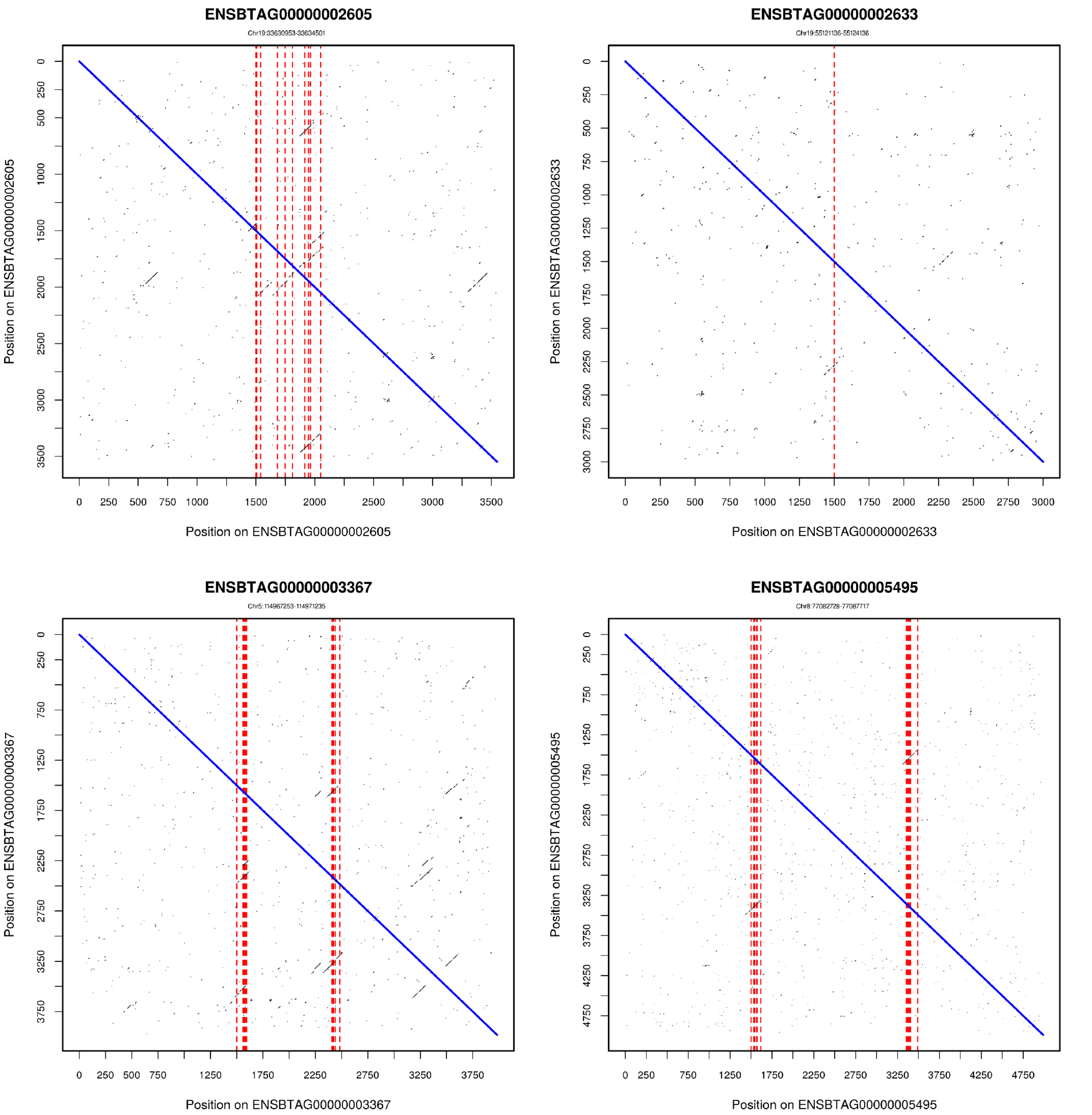

ENSBTAG00000002605
Chr19:33630953-33634501
Position on ENSBTAG00000002605
Position on ENSBTAG00000002605
ENSBTAG00000002633
Chr19:55121136-55124136
Position on ENSBTAG00000002633
Position on ENSBTAG00000002633
ENSBTAG00000003367
Chr5:114967253-114971235
Position on ENSBTAG00000003367
Position on ENSBTAG00000003367
ENSBTAG00000005495
Chr8:77082728-77087717
Position on ENSBTAG00000005495
Position on ENSBTAG00000005495

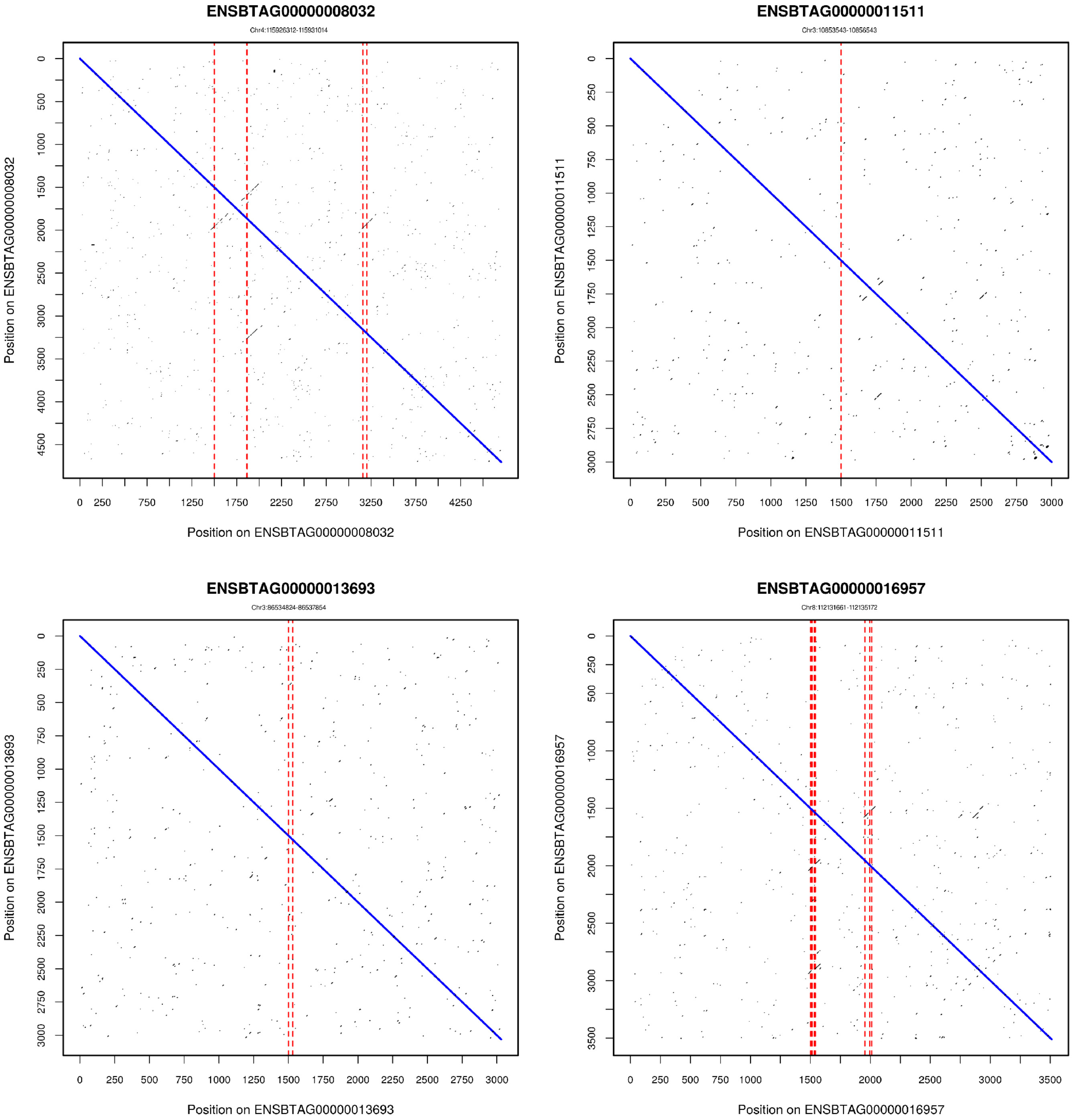

ENSBTAG00000008032
Chr4:115926312-115931014
Position on ENSBTAG00000008032
Position on ENSBTAG00000008032
ENSBTAG00000011511
Chr3:10853543-10856543
Position on ENSBTAG00000011511
Position on ENSBTAG00000011511
ENSBTAG00000013693
Chr3:86534824-86537854
Position on ENSBTAG00000013693
Position on ENSBTAG00000013693
ENSBTAG00000016957
Chr8:112131661-112135172
Position on ENSBTAG00000016957
Position on ENSBTAG00000016957

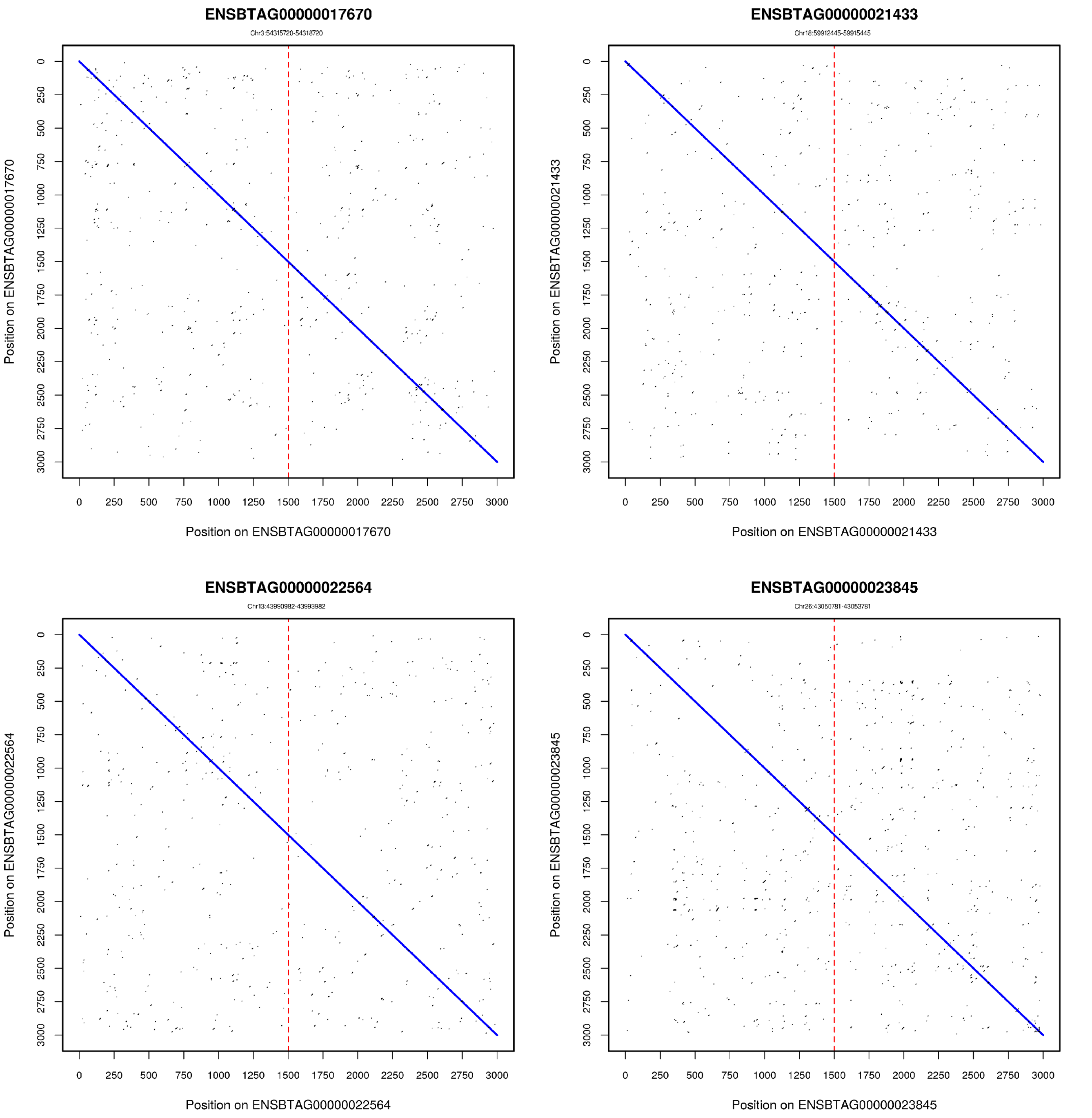
ENSBTAG00000017670
Chr3:54315720-54318720
Position on ENSBTAG00000017670
Position on ENSBTAG00000017670
0
250
500
750
1000
1250
1500
1750
2000
2250
2500
2750
3000
ENSBTAG00000021433
Chr18:59912445-59915445
Position on ENSBTAG00000021433
Position on ENSBTAG00000021433
ENSBTAG00000022564
Chr13:43990982-43993982
Position on ENSBTAG00000022564
Position on ENSBTAG00000022564
ENSBTAG00000023845
Chr26:43050781-43053781
Position on ENSBTAG00000023845
Position on ENSBTAG00000023845

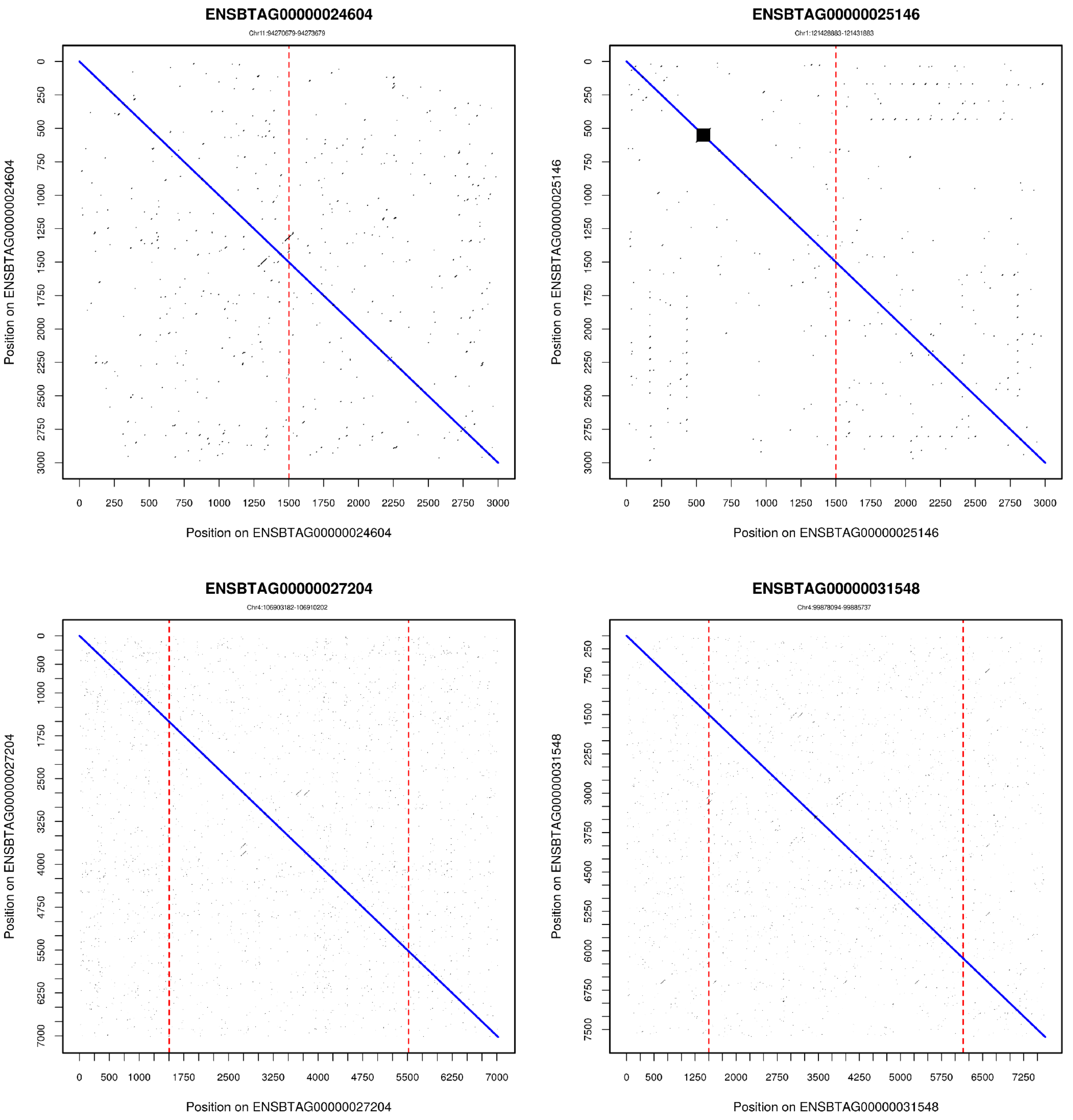
ENSBTAG00000024604
Chr11:94270679-94273679
Position on ENSBTAG00000024604
Position on ENSBTAG00000024604
ENSBTAG00000025146
Chr1:121428883-121431883
Position on ENSBTAG00000025146
Position on ENSBTAG00000025146
ENSBTAG00000027204
Chr4:106903182-106910202
Position on ENSBTAG00000027204
Position on ENSBTAG00000027204
ENSBTAG00000031548
Chr4:99878094-99885737
Position on ENSBTAG00000031548
Position on ENSBTAG00000031548

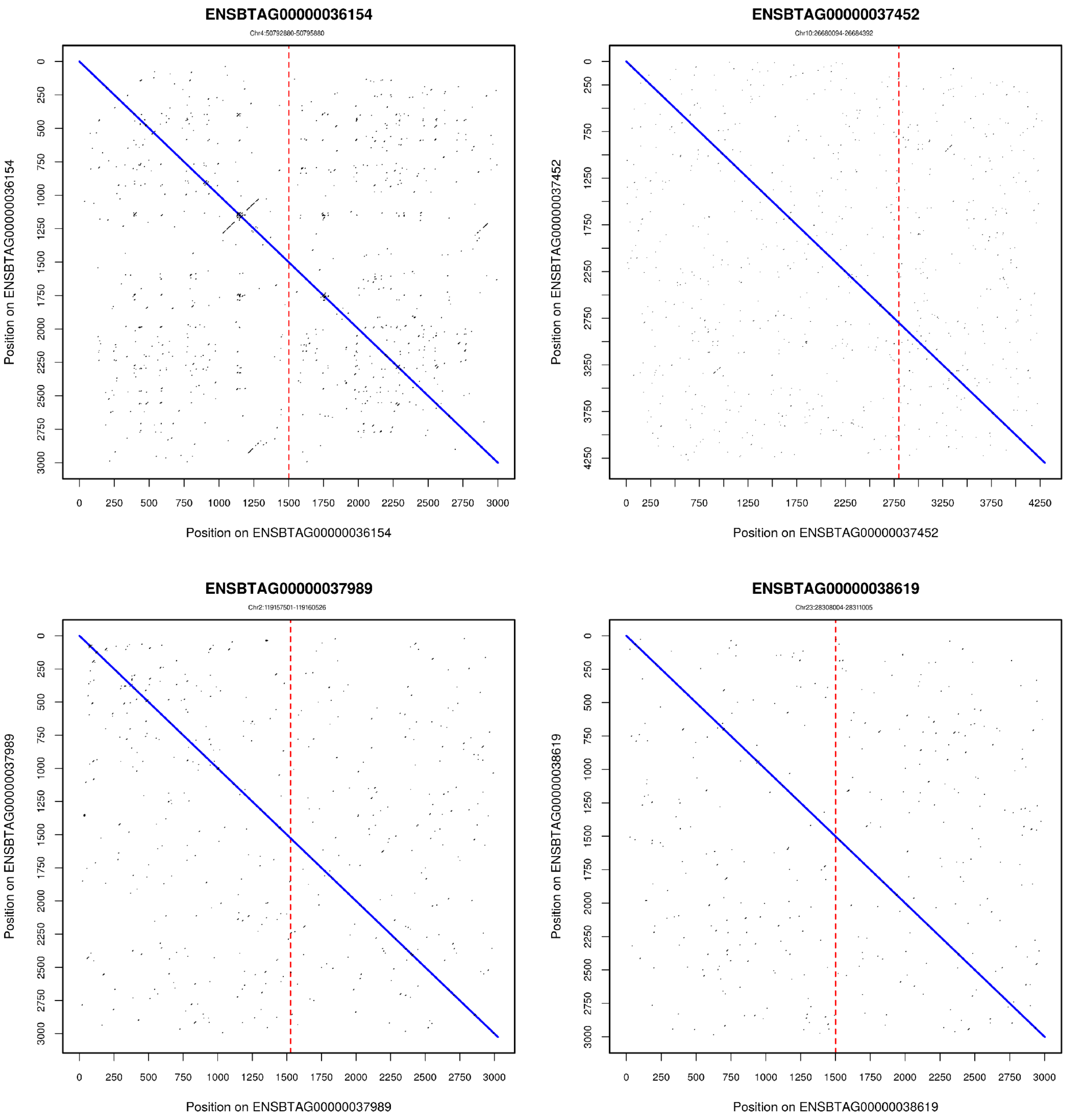
ENSBTAG00000036154
Chr4:50792880-50795880
Position on ENSBTAG00000036154
Position on ENSBTAG00000036154
0 250 500 750 1000 1250 1500 1750 2000 2250 2500 2750 3000
ENSBTAG00000037452
Chr10:26680094-26684392
Position on ENSBTAG00000037452
Position on ENSBTAG00000037452
0 250 750 1250 1750 2250 2750 3250 3750 4250
ENSBTAG00000037989
Chr2:119157501-119160526
Position on ENSBTAG00000037989
Position on ENSBTAG00000037989
0 250 500 750 1000 1250 1500 1750 2000 2250 2500 2750 3000
ENSBTAG00000038619
Chr23:28308004-28311005
Position on ENSBTAG00000038619
Position on ENSBTAG00000038619
0 250 500 750 1000 1250 1500 1750 2000 2250 2500 2750 3000

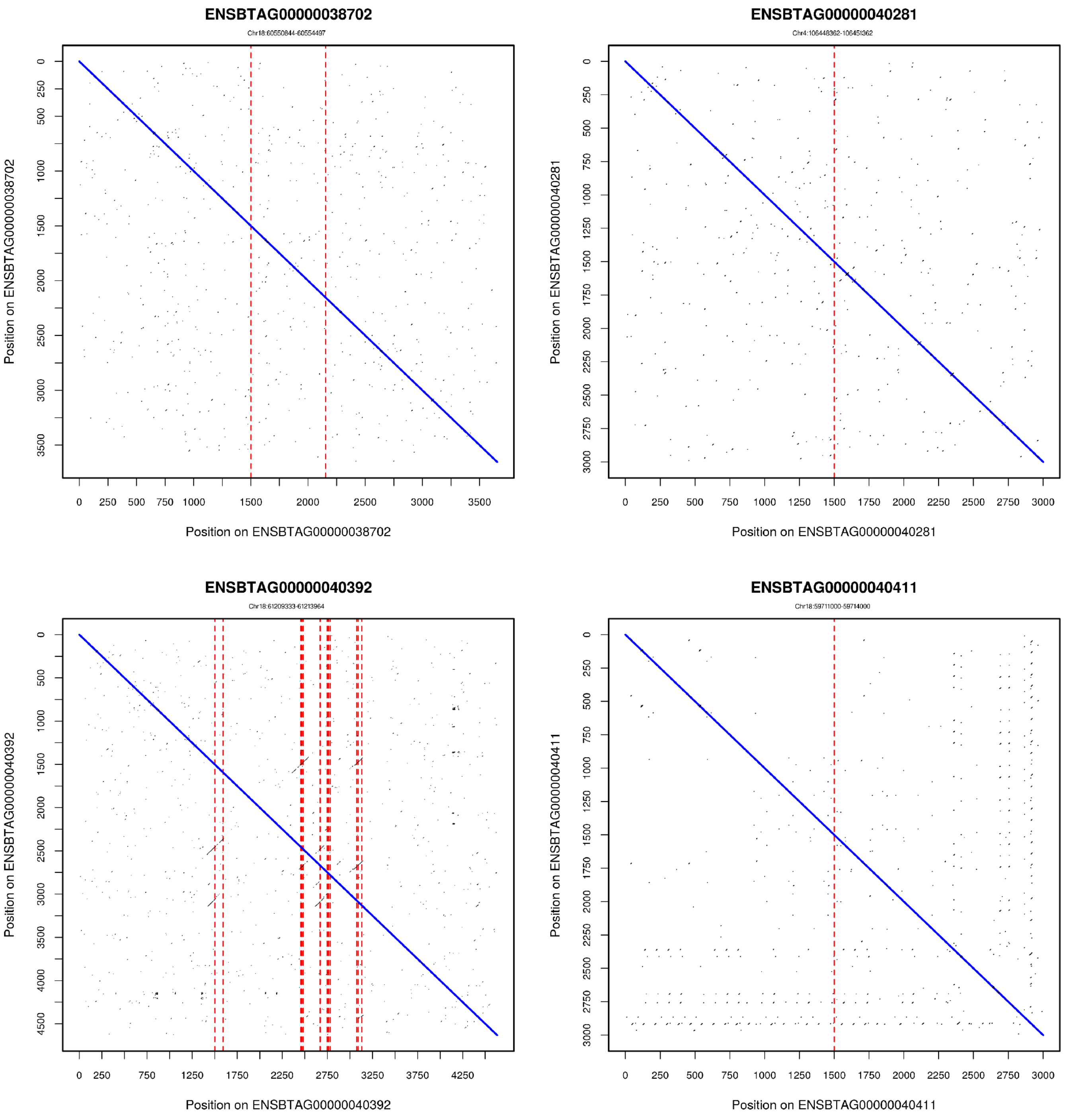
ENSBTAG00000038702
Chr18:60550844-60554497
Position on ENSBTAG00000038702
Position on ENSBTAG00000038702
ENSBTAG00000040281
Chr4:106448362-106451362
Position on ENSBTAG00000040281
Position on ENSBTAG00000040281
ENSBTAG00000040392
Chr18:61209333-61213964
Position on ENSBTAG00000040392
Position on ENSBTAG00000040392
ENSBTAG00000040411
Chr18:59711000-59714000
Position on ENSBTAG00000040411
Position on ENSBTAG00000040411

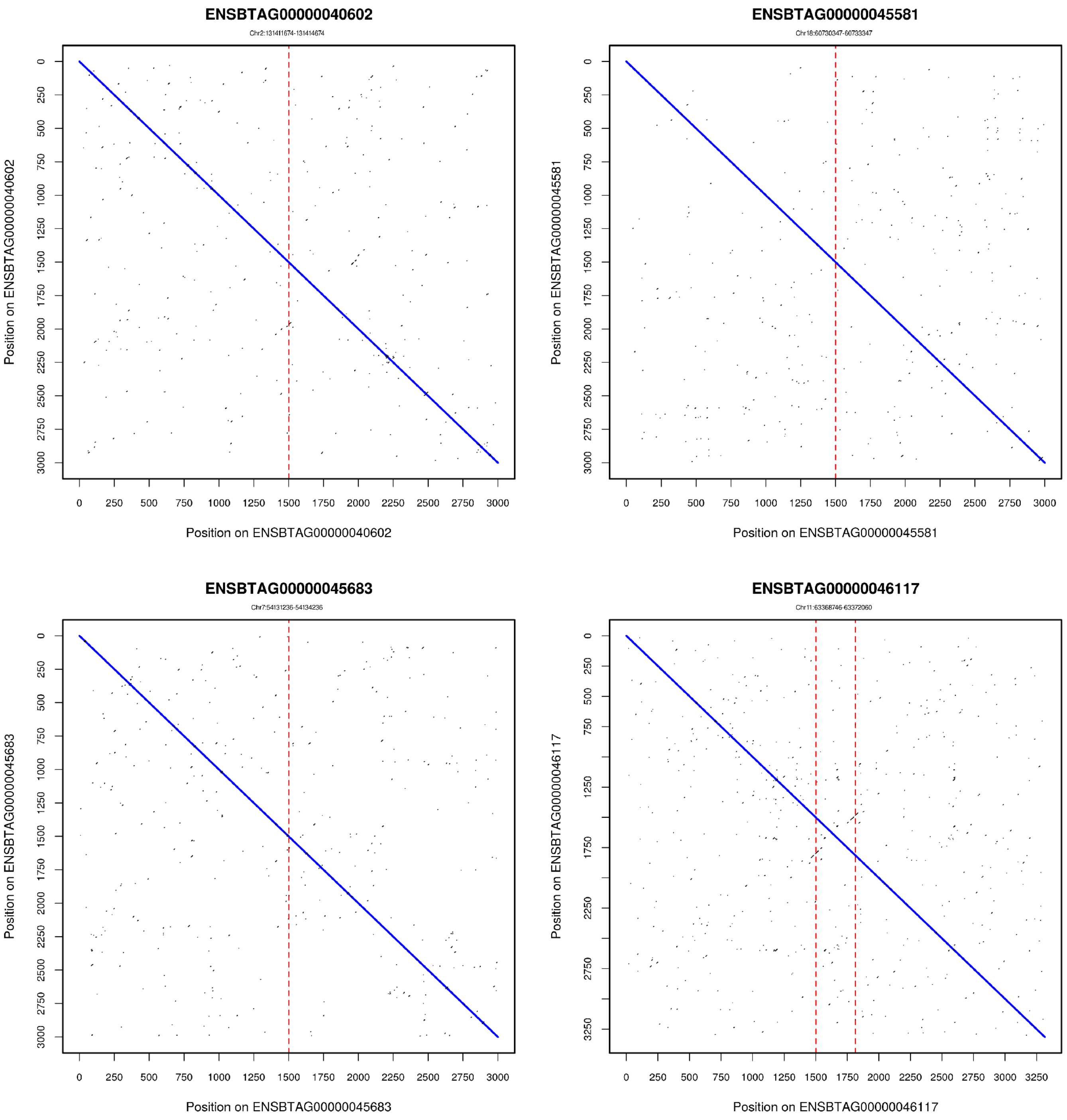
ENSBTAG00000040602
Chr2:131411674-131414674
Position on ENSBTAG00000040602
Position on ENSBTAG00000040602
ENSBTAG00000045581
Chr18:60730347-60733347
Position on ENSBTAG00000045581
Position on ENSBTAG00000045581
ENSBTAG00000045683
Chr7:54131236-54134236
Position on ENSBTAG00000045683
Position on ENSBTAG00000045683
ENSBTAG00000046117
Chr11:63368746-63372060
Position on ENSBTAG00000046117
Position on ENSBTAG00000046117

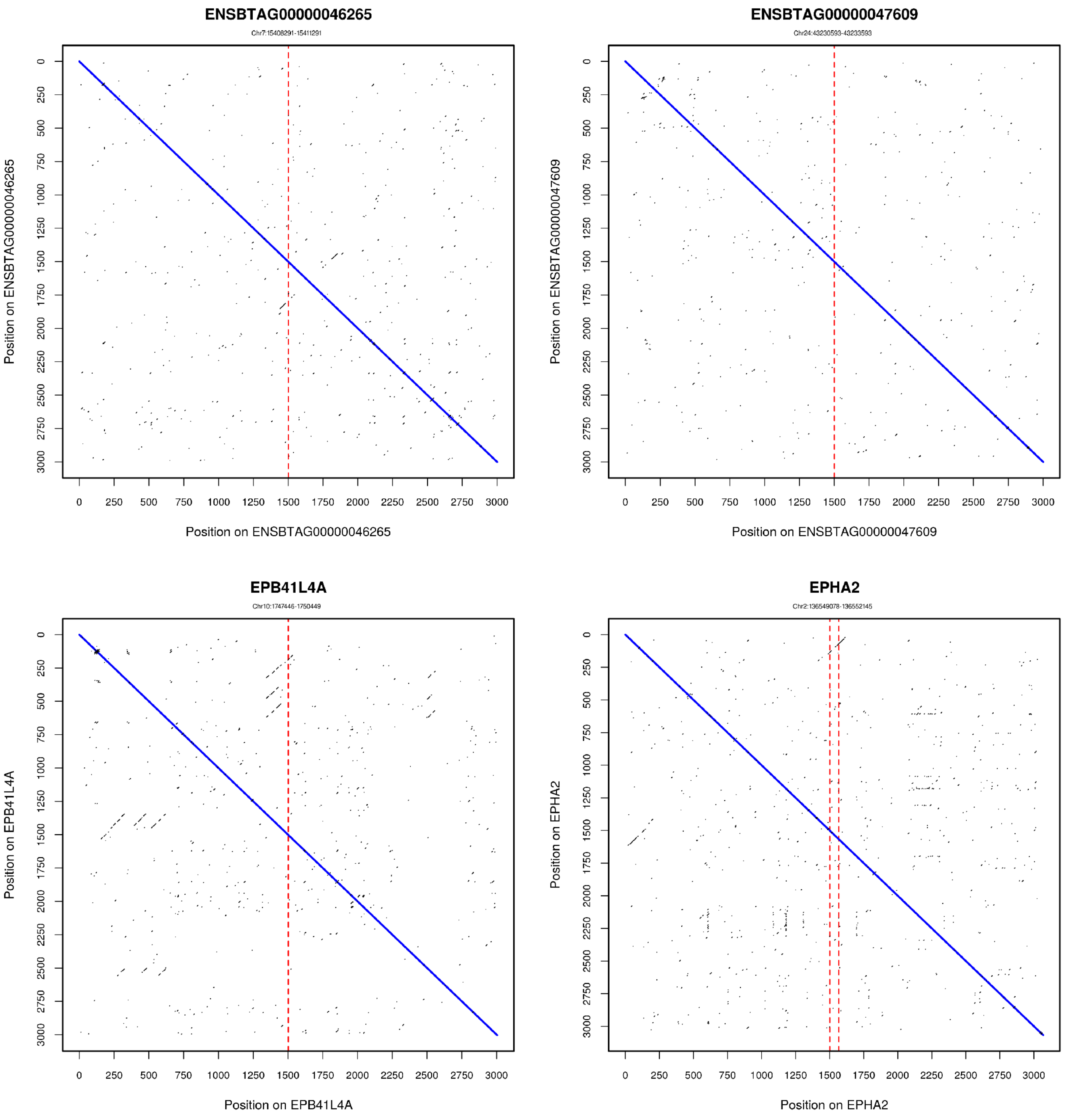

ENSBTAG00000046265
Chr7:15408291-15411291
Position on ENSBTAG00000046265
Position on ENSBTAG00000046265
ENSBTAG00000047609
Chr24:43230593-43233593
Position on ENSBTAG00000047609
Position on ENSBTAG00000047609
EPB41L4A
Chr10:1747446-1750449
Position on EPB41L4A
Position on EPB41L4A
EPHA2
Chr2:136549078-136552145
Position on EPHA2
Position on EPHA2

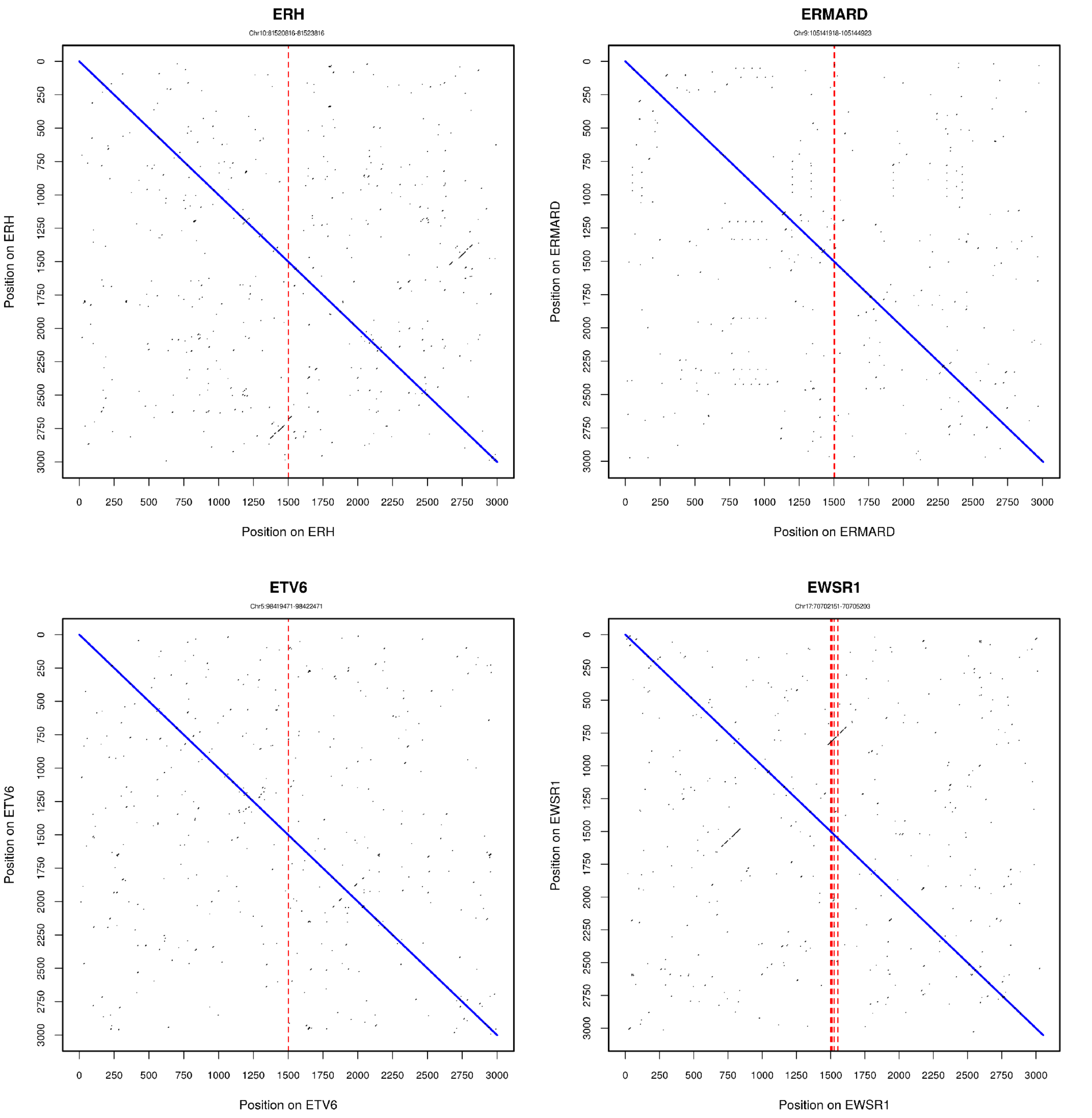
ERH
Chr10:81520816-81523816
Position on ERH
Position on ERH
ERMARD
Chr9:105141918-105144923
Position on ERMARD
Position on ERMARD
ETV6
Chr5:98419471-98422471
Position on ETV6
Position on ETV6
EWSR1
Chr17:70702151-70705203
Position on EWSR1
Position on EWSR1

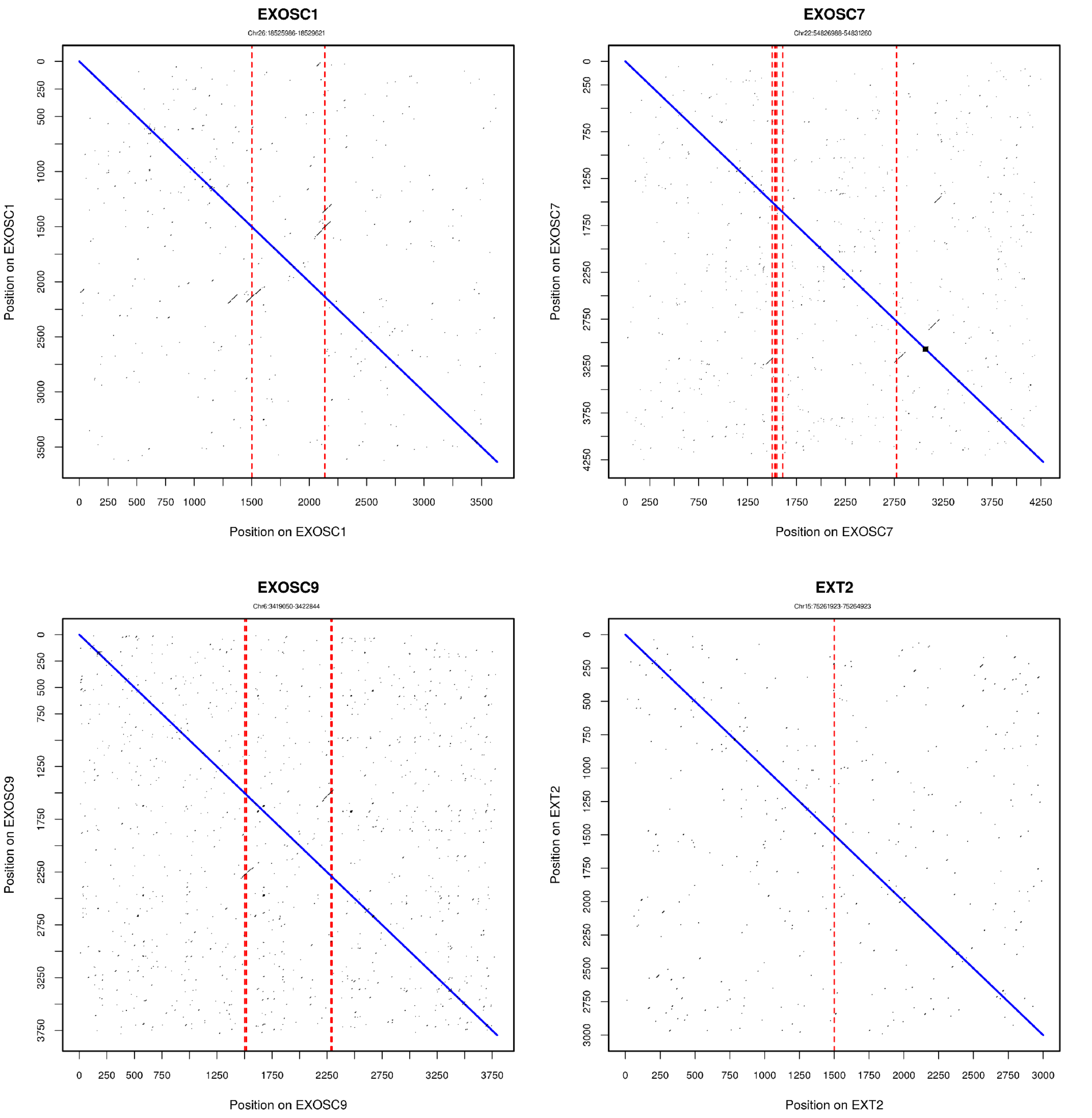
EXOSC1
Chr26:18525986-18529621
Position on EXOSC1
Position on EXOSC1
EXOSC7
Chr22:54826988-54831260
Position on EXOSC7
Position on EXOSC7
EXOSC9
Chr6:3419050-3422844
Position on EXOSC9
Position on EXOSC9
EXT2
Chr15:75261923-75264923
Position on EXT2
Position on EXT2

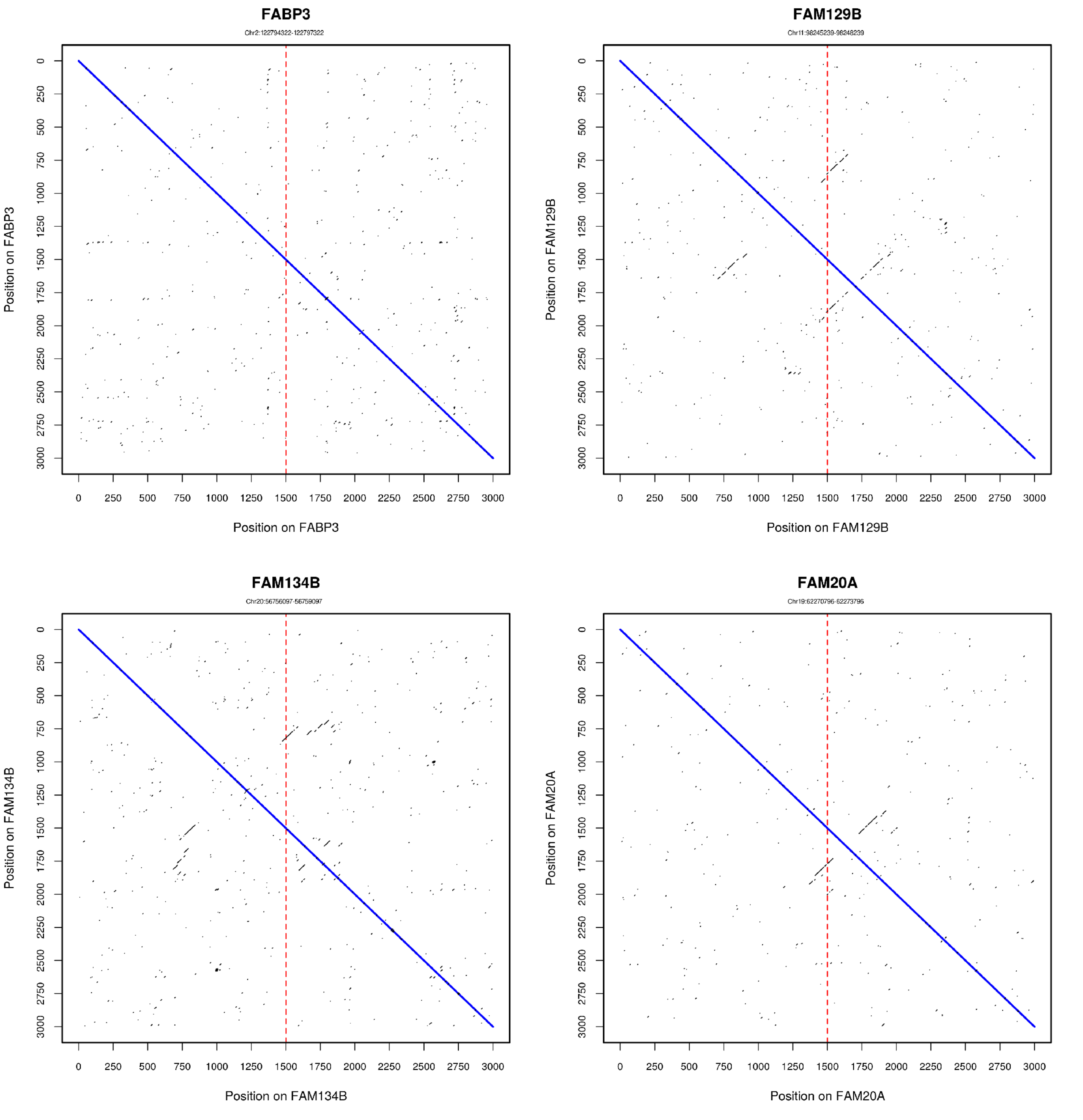
FABP3
Chr2:122794322-122797322
Position on FABP3
Position on FABP3
FAM129B
Chr11:98245239-98248239
Position on FAM129B
Position on FAM129B
FAM134B
Chr20:56756097-56759097
Position on FAM134B
Position on FAM134B
FAM20A
Chr19:62270796-62273796
Position on FAM20A
Position on FAM20A

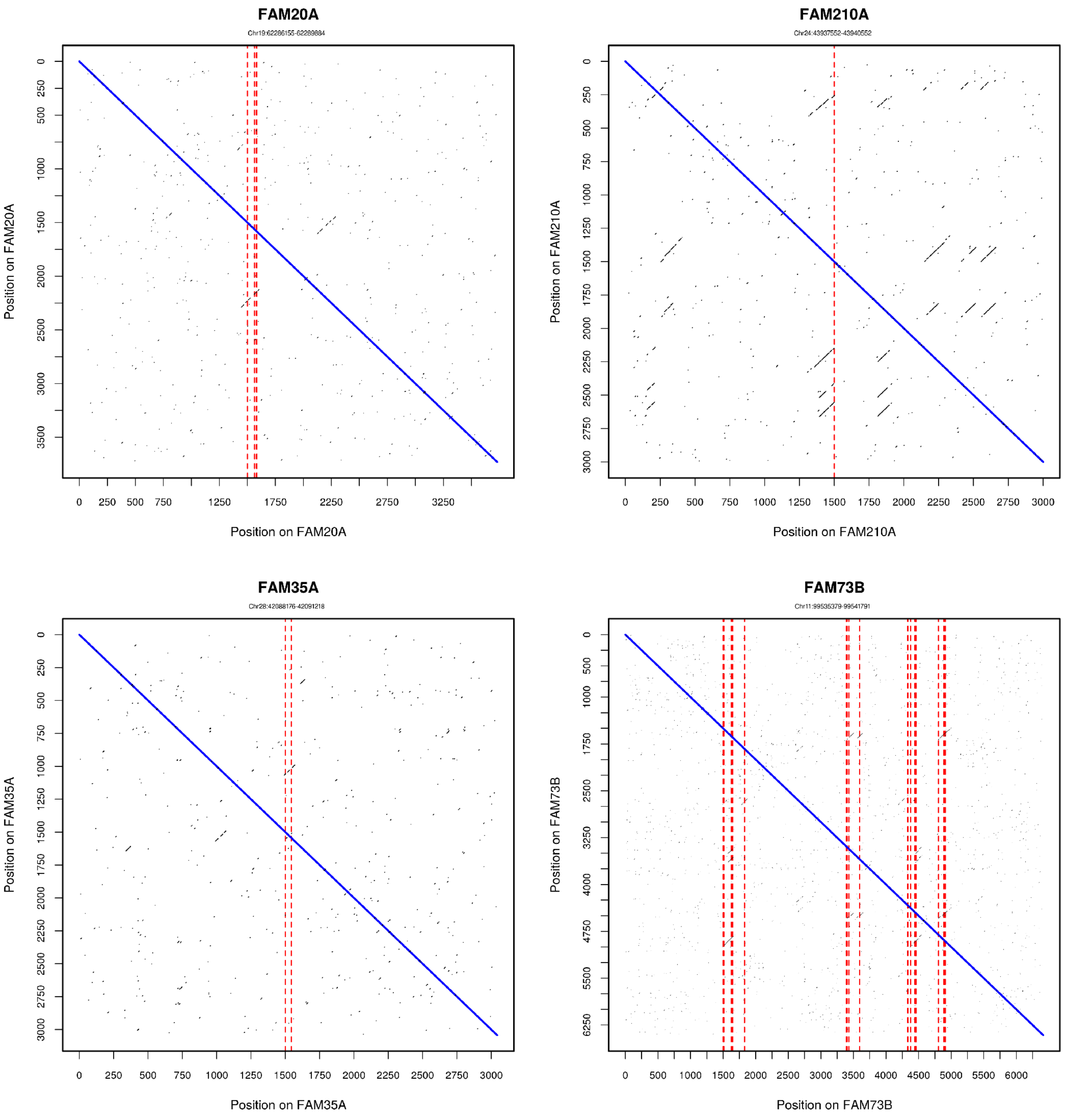
FAM20A
Chr19:62286155-62289884
Position on FAM20A
Position on FAM20A
FAM210A
Chr24:43937552-43940552
Position on FAM210A
Position on FAM210A
FAM35A
Chr28:42088176-42091218
Position on FAM35A
Position on FAM35A
FAM73B
Chr11:99535379-99541791
Position on FAM73B
Position on FAM73B

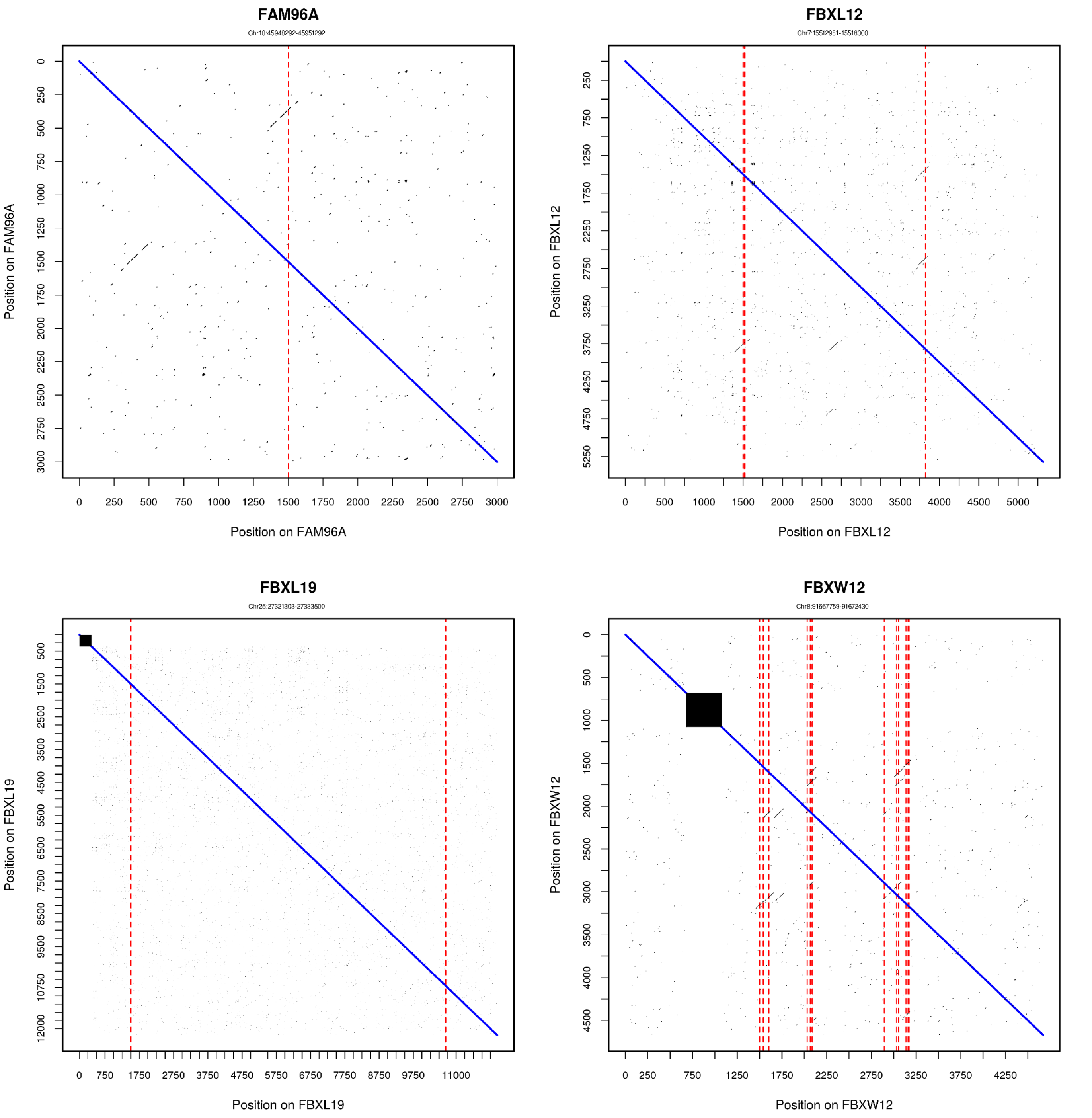
FAM96A
Chr10:45948292-45951292
Position on FAM96A
Position on FAM96A
FBXL12
Chr7:15512981-15518300
Position on FBXL12
Position on FBXL12
FBXL19
Chr25:27321303-27333500
Position on FBXL19
Position on FBXL19
FBXW12
Chr8:91667759-91672430
Position on FBXW12
Position on FBXW12

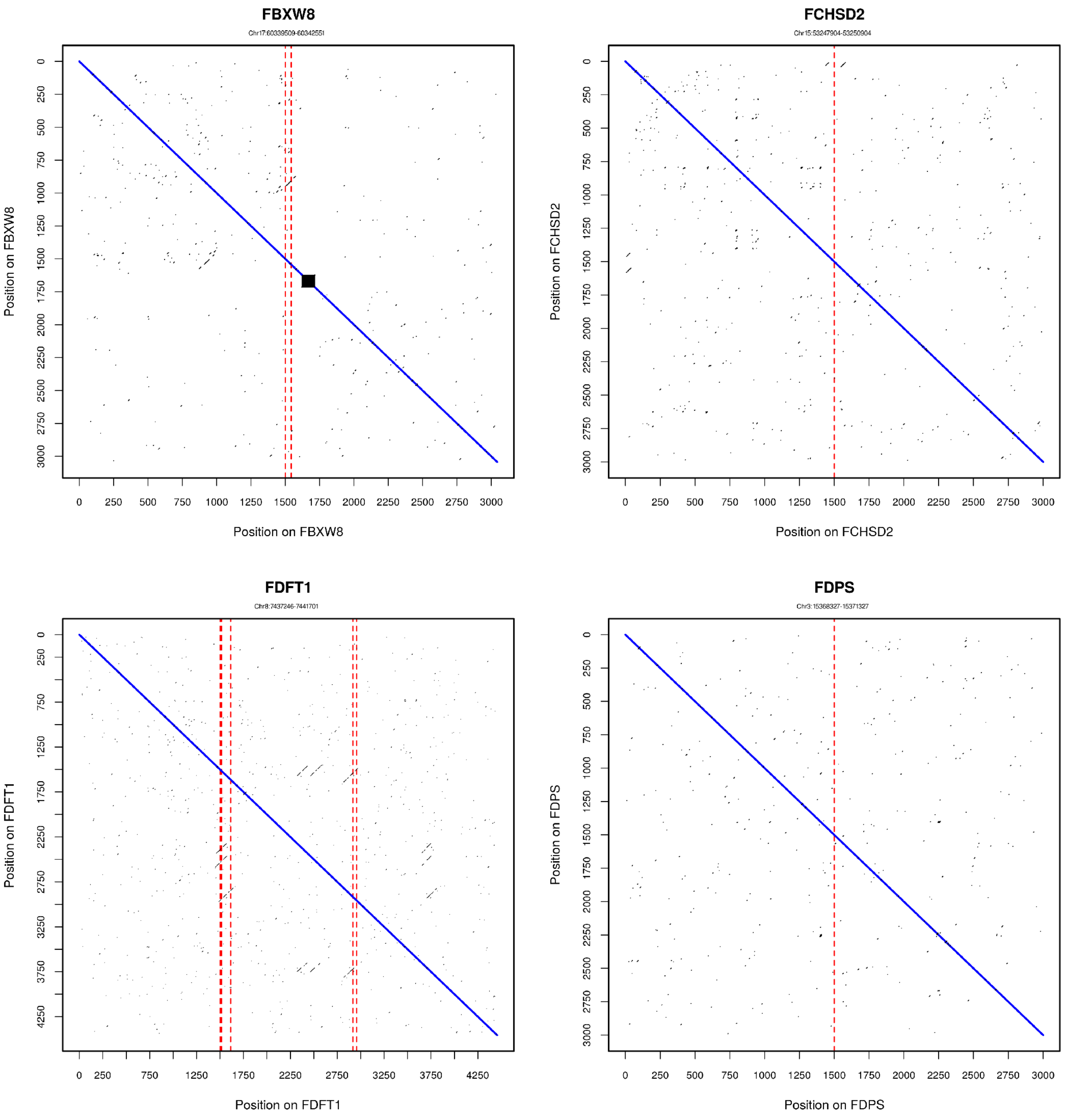
FBXW8
Chr17:60339509-60342551
Position on FBXW8
Position on FBXW8
FCHSD2
Chr15:53247904-53250904
Position on FCHSD2
Position on FCHSD2
FDFT1
Chr8:7437246-7441701
Position on FDFT1
Position on FDFT1
FDPS
Chr3:15368327-15371327
Position on FDPS
Position on FDPS

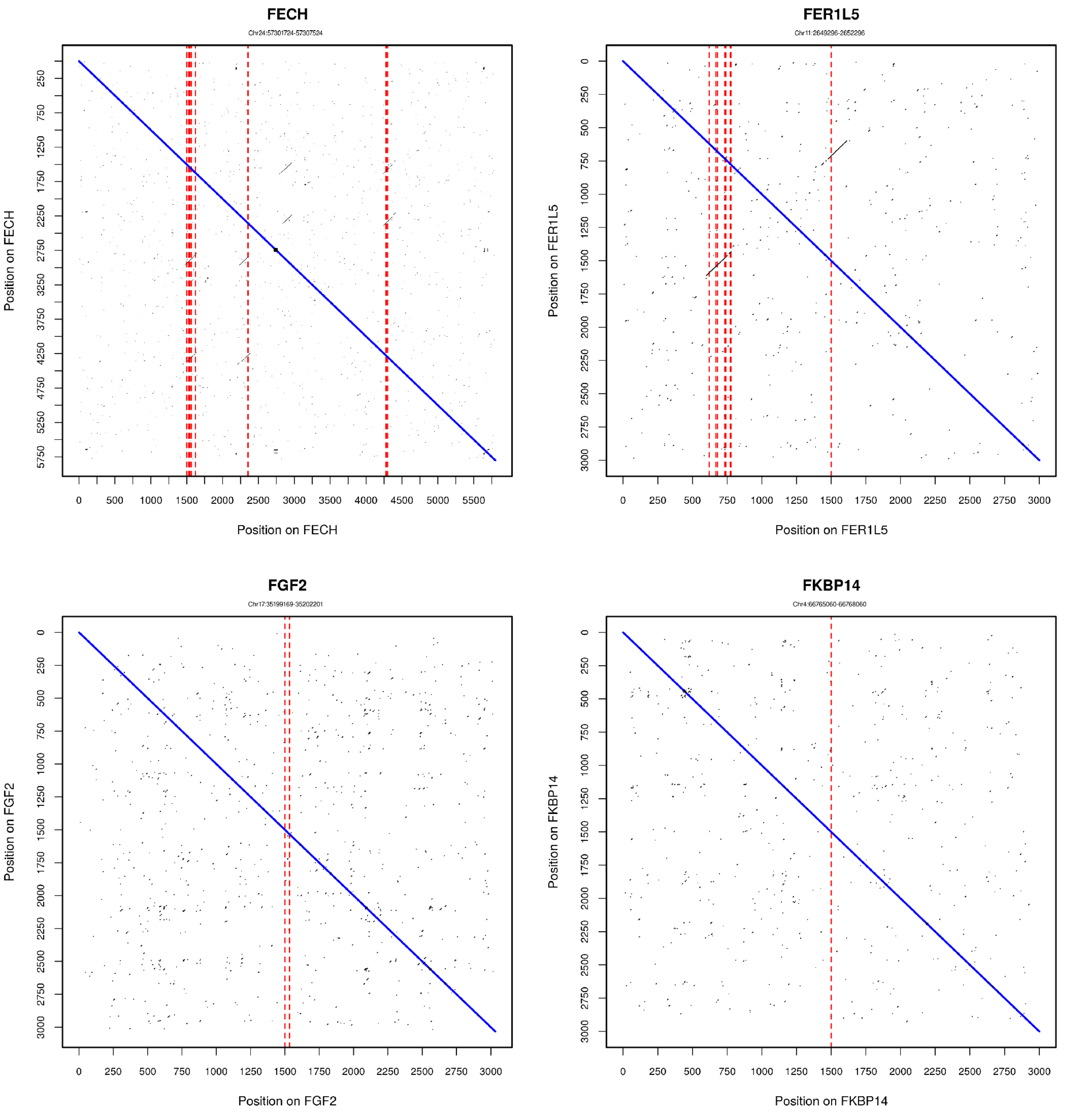
FECH
Chr24:57301724-57307524
Position on FECH
Position on FECH
FER1L5
Chr11:2649296-2652296
Position on FER1L5
Position on FER1L5
FGF2
Chr17:35199169-35202201
Position on FGF2
Position on FGF2
FKBP14
Chr4:66765060-66768060
Position on FKBP14
Position on FKBP14

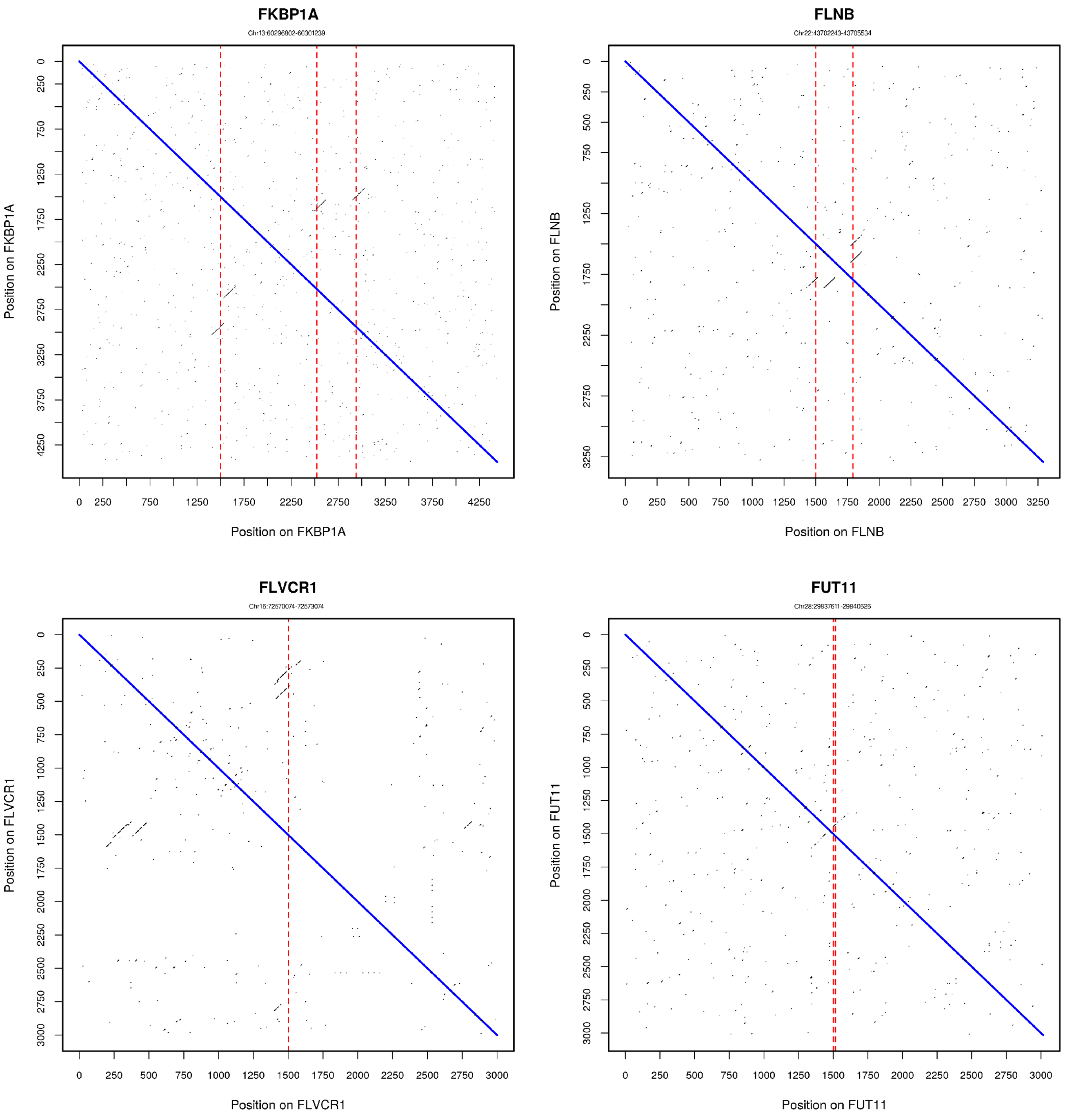
FKBP1A
Chr13:60296802-60301239
Position on FKBP1A
Position on FKBP1A
FLNB
Chr22:43702243-43705534
Position on FLNB
Position on FLNB
FLVCR1
Chr16:72570074-72573074
Position on FLVCR1
Position on FLVCR1
FUT11
Chr28:29837611-29840626
Position on FUT11
Position on FUT11

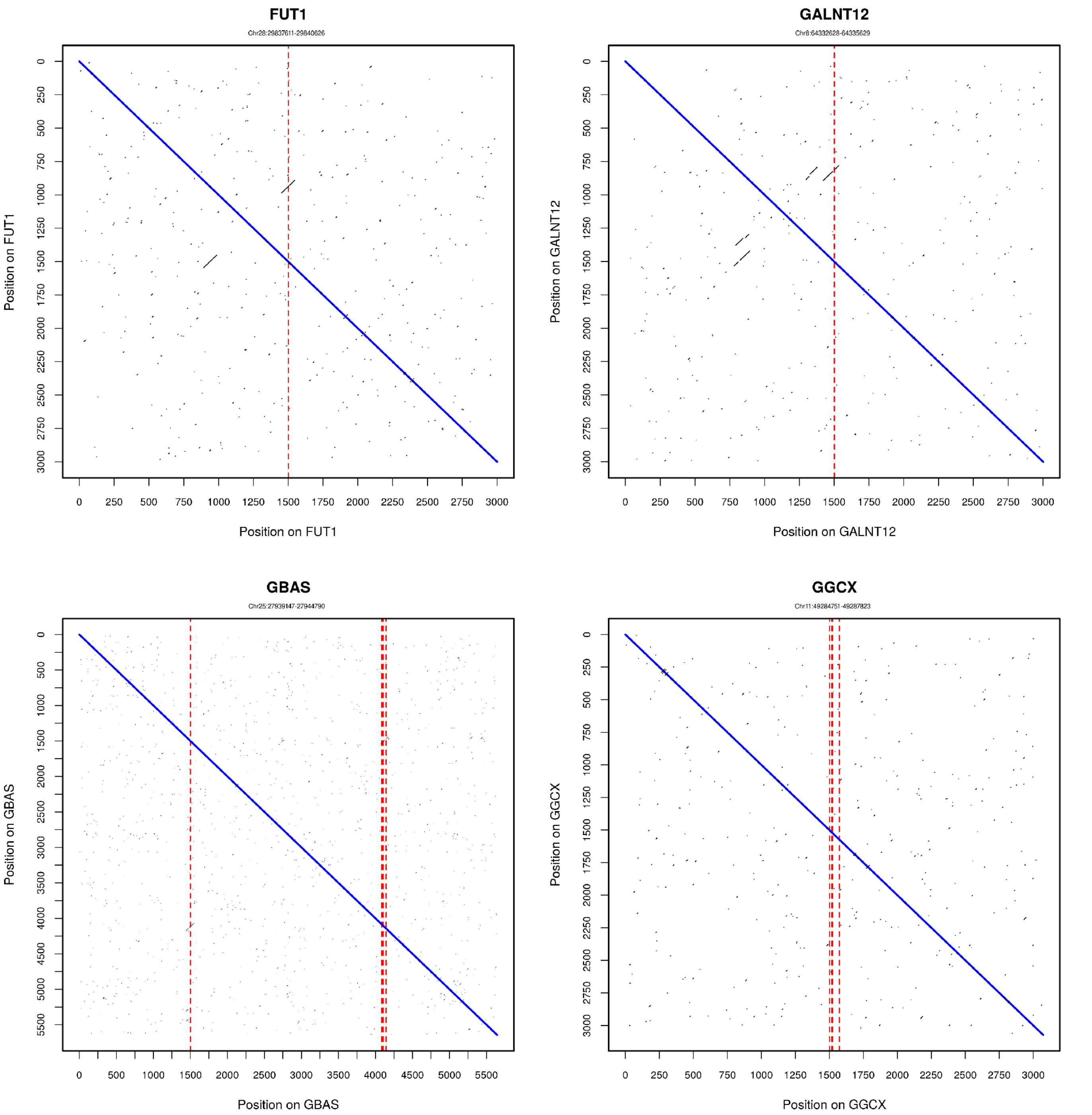
FUT1
Chr28:29837611-29840626
Position on FUT1
Position on FUT1
GALNT12
Chr8:64332628-64335629
Position on GALNT12
Position on GALNT12
GBAS
Chr25:27939147-27944790
Position on GBAS
Position on GBAS
GGCX
Chr11:49284751-49287823
Position on GGCX
Position on GGCX

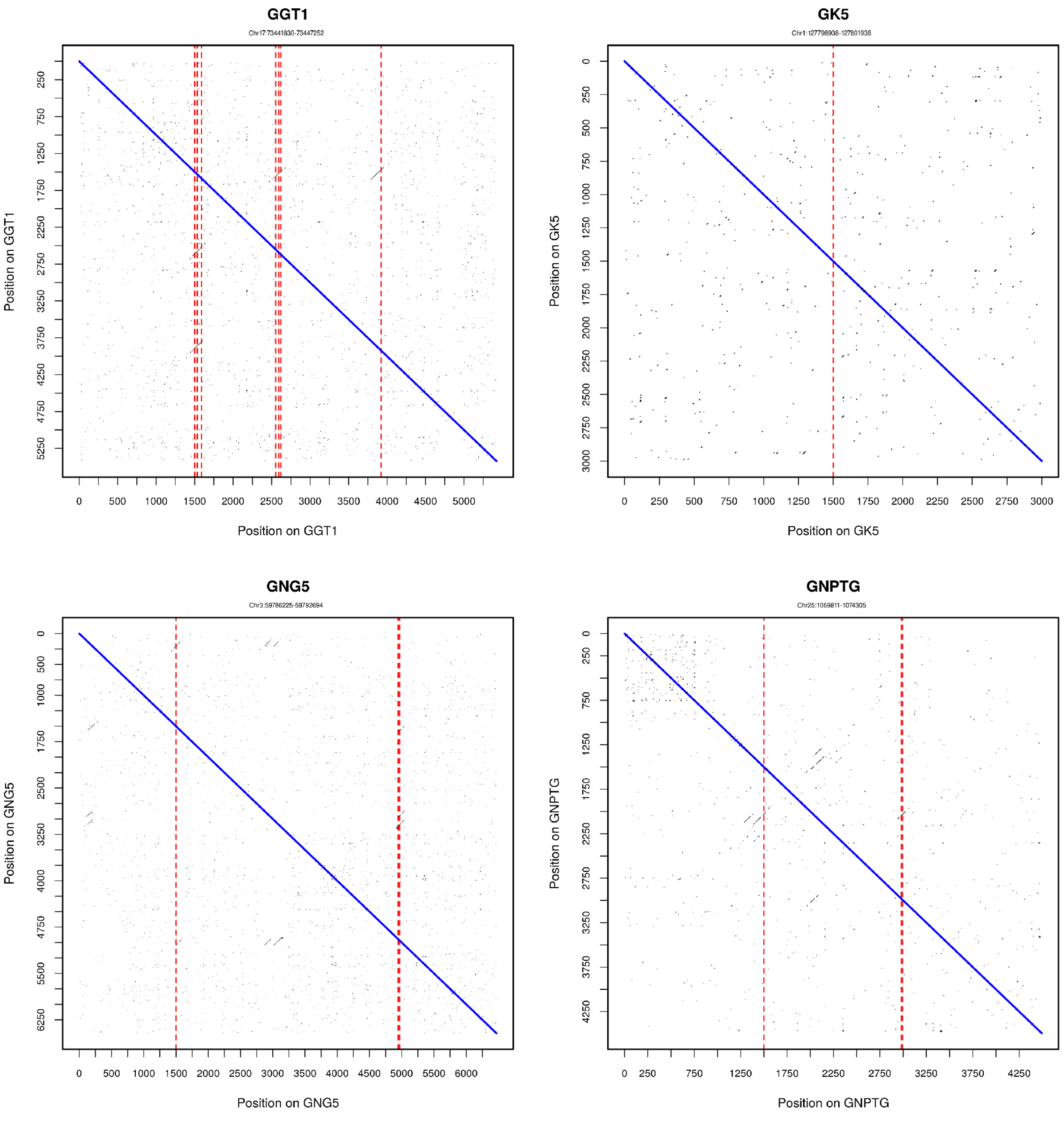
GGT1
Chr17:73441830-73447252
Position on GGT1
Position on GGT1
GK5
Chr1:127798938-127801938
Position on GK5
Position on GK5
GNG5
Chr3:59786225-59792694
Position on GNG5
Position on GNG5
GNPTG
Chr25:1069811-1074305
Position on GNPTG
Position on GNPTG

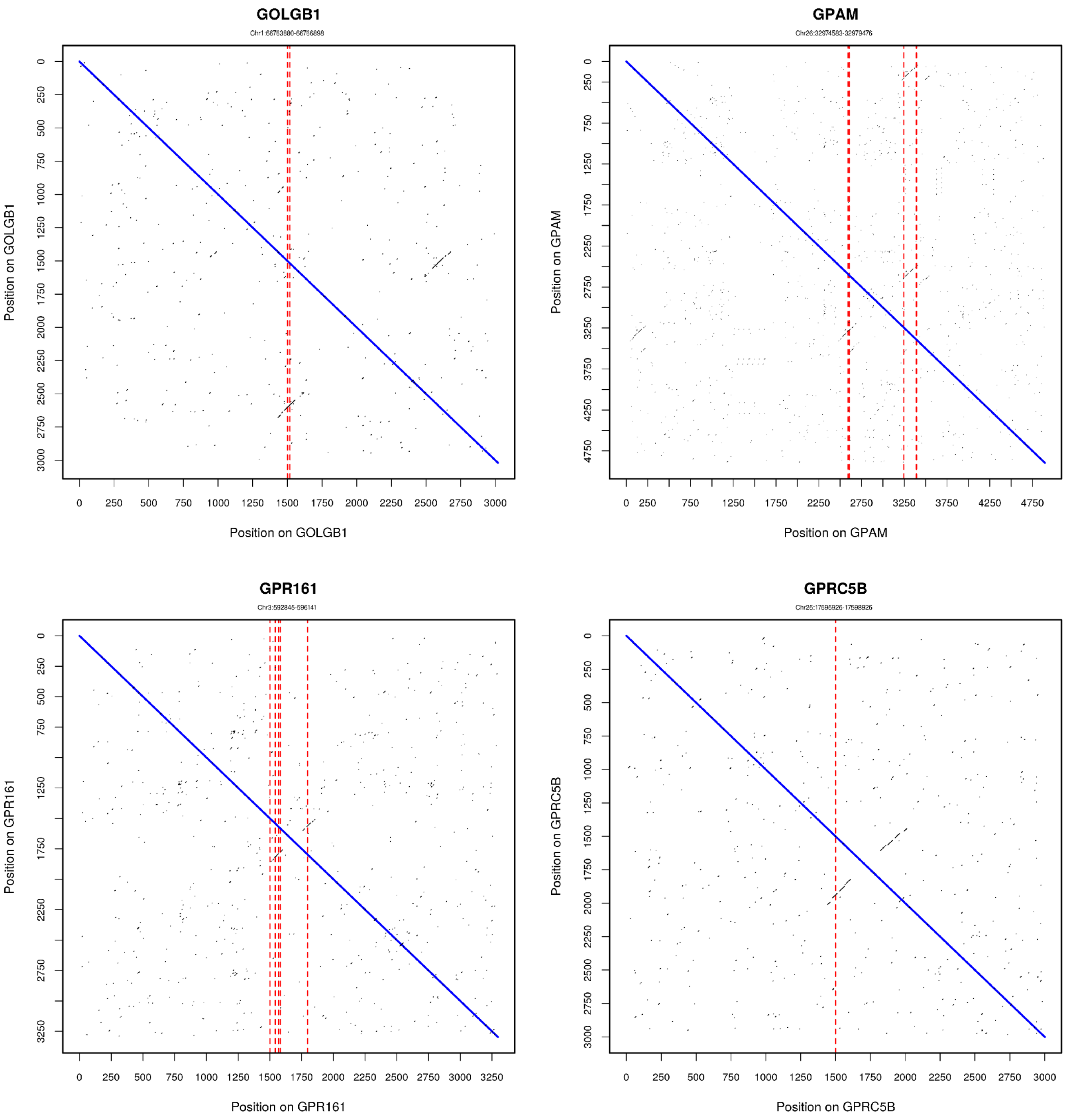

GOLGB1
Chr1:66763880-66766898
Position on GOLGB1
Position on GOLGB1
GPAM
Chr26:32974583-32979476
Position on GPAM
Position on GPAM
GPR161
Chr3:592845-596141
Position on GPR161
Position on GPR161
GPRC5B
Chr25:17595926-17598926
Position on GPRC5B
Position on GPRC5B

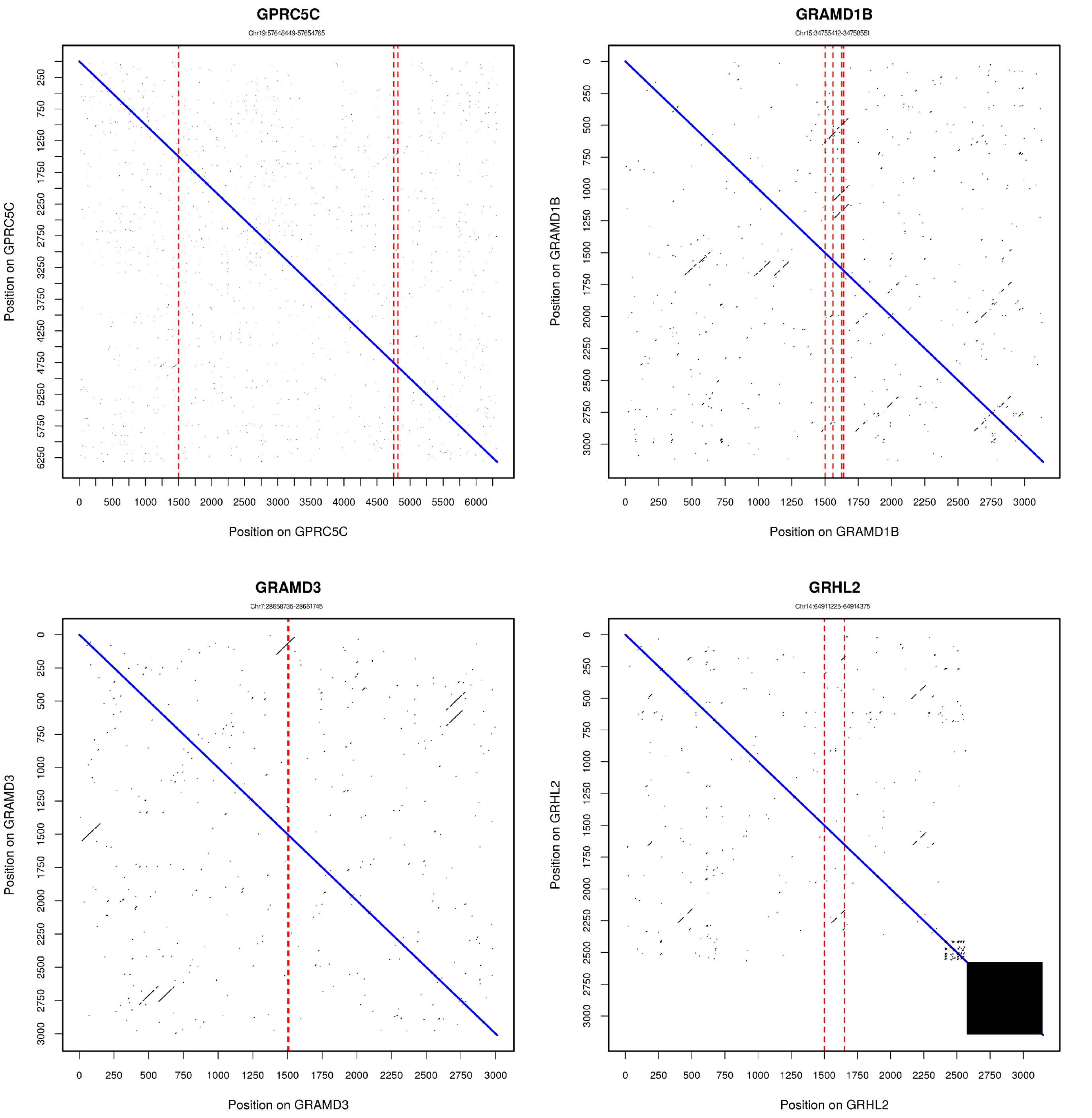
GPRC5C
Chr19:57648449-57654765
Position on GPRC5C
Position on GPRC5C
GRAMD1B
Chr15:34755412-34758551
Position on GRAMD1B
Position on GRAMD1B
GRAMD3
Chr7:28658735-28661745
Position on GRAMD3
Position on GRAMD3
GRHL2
Chr14:64911225-64914375
Position on GRHL2
Position on GRHL2

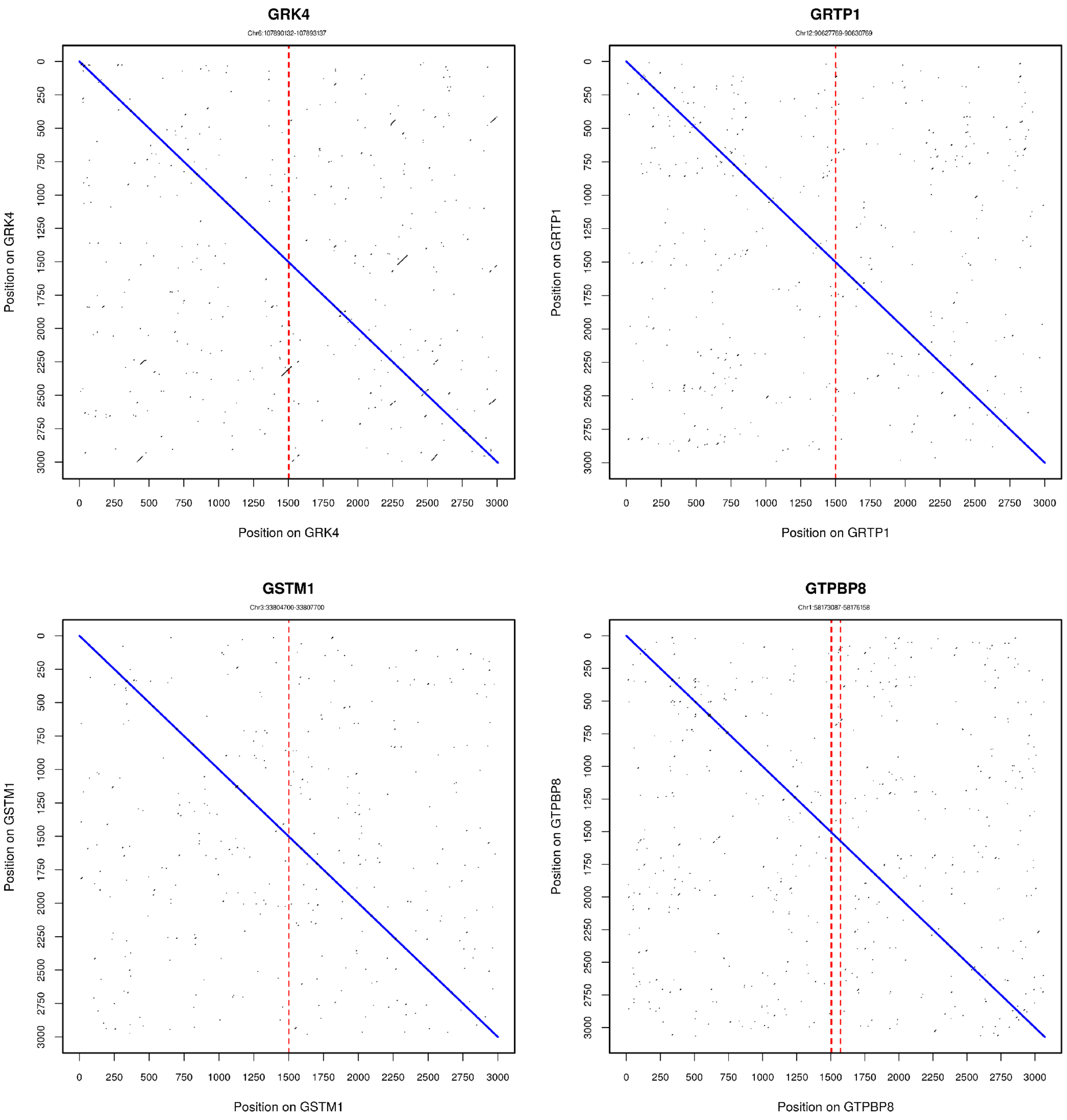
GRK4
Chr6:107890132-107893137
Position on GRK4
Position on GRK4
GRTP1
Chr12:90627769-90630769
Position on GRTP1
Position on GRTP1
GSTM1
Chr3:33804700-33807700
Position on GSTM1
Position on GSTM1
GTPBP8
Chr1:58173087-58176158
Position on GTPBP8
Position on GTPBP8

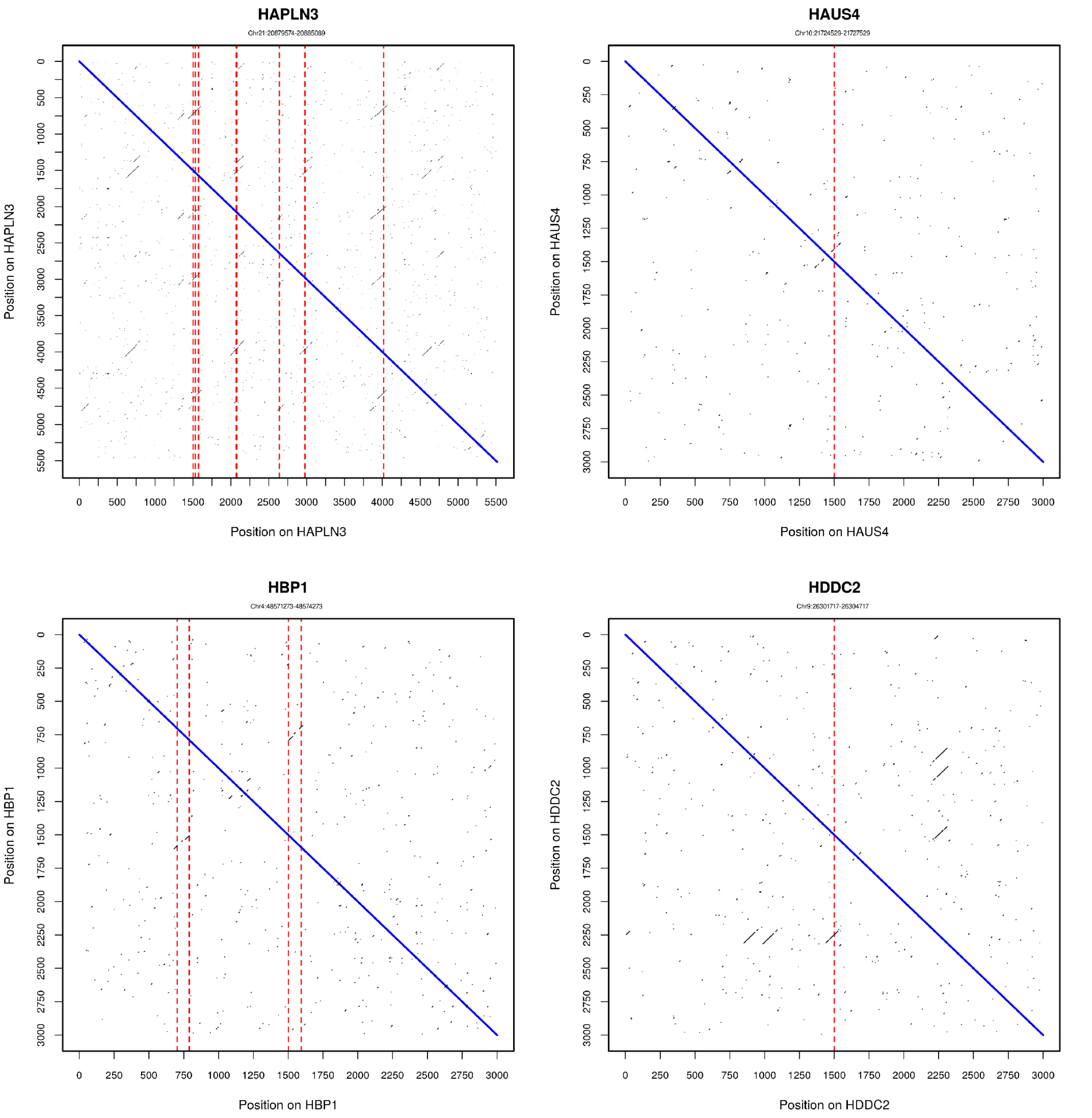
HAPLN3
Chr21:20879574-20885089
Position on HAPLN3
Position on HAPLN3
HAUS4
Chr10:21724529-21727529
Position on HAUS4
Position on HAUS4
HBP1
Chr4:48571273-48574273
Position on HBP1
Position on HBP1
HDDC2
Chr9:26301717-26304717
Position on HDDC2
Position on HDDC2

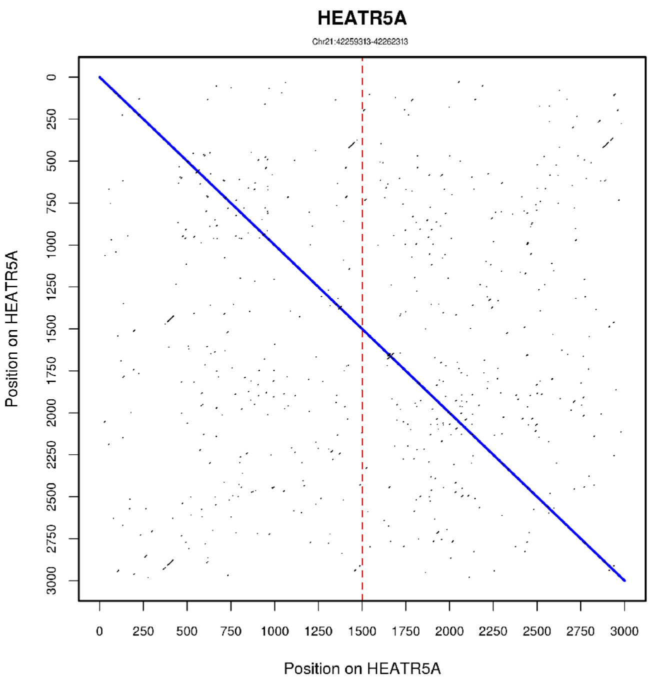
HEATR5A
Chr21:42259313-42262313
Position on HEATR5A
Position on HEATR5A

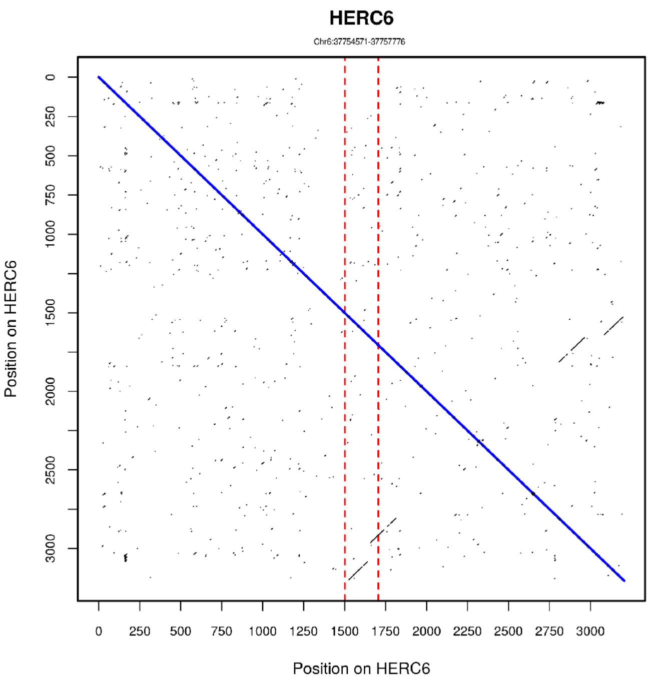
HERC6
Chr6:37754571-37757776
Position on HERC6
Position on HERC6

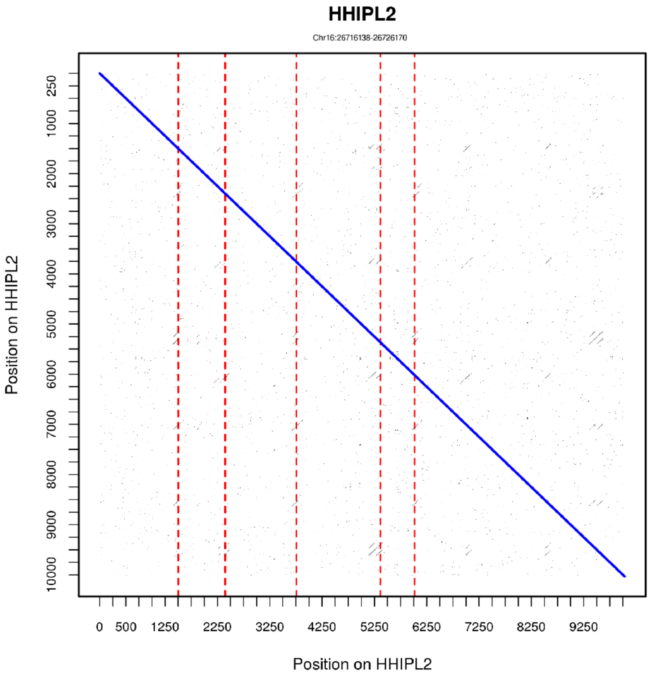
HHIPL2
Chr16:26716138-26726170
Position on HHIPL2
Position on HHIPL2

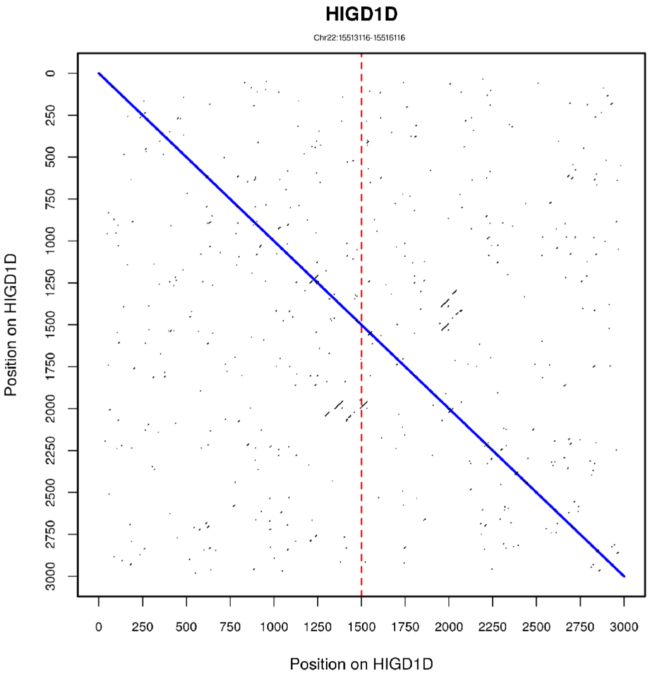
HIGD1D
Chr22:15513116-15516116
Position on HIGD1D
Position on HIGD1D

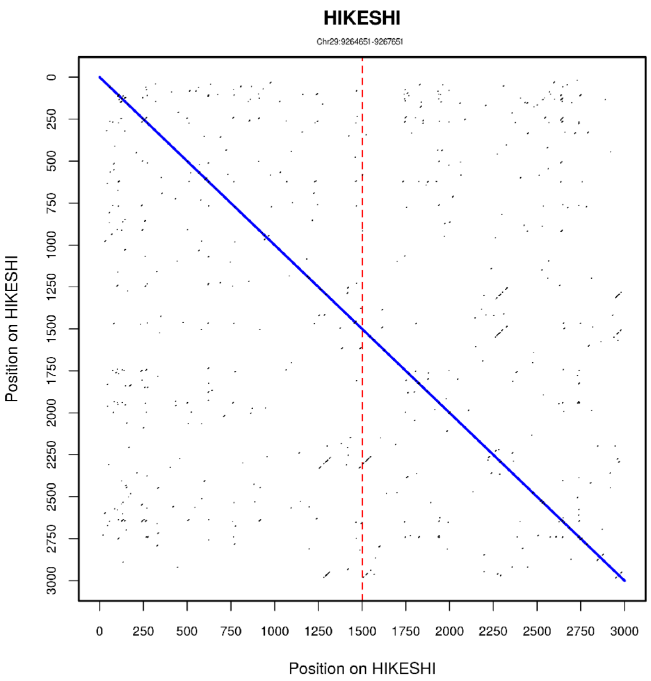
HIKESHI
Chr29:9264651-9267651
Position on HIKESHI
Position on HIKESHI

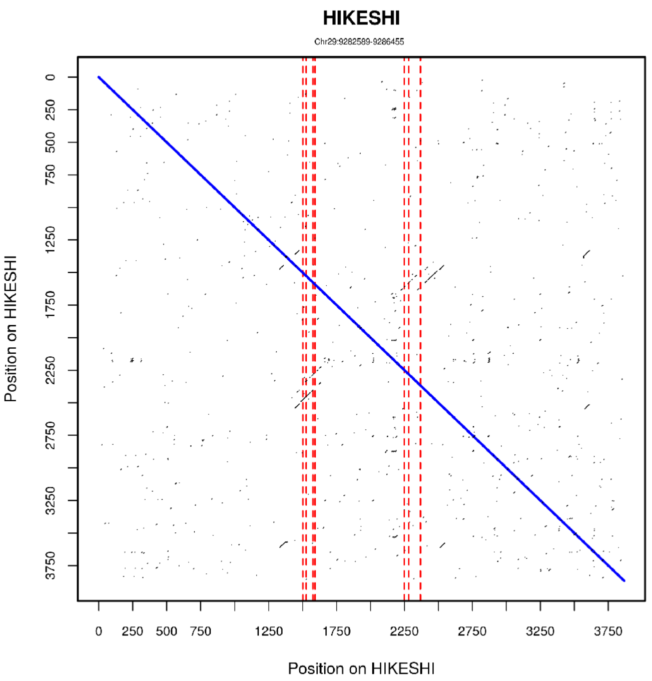
HIKESHI
Chr29:9282589-9286455
Position on HIKESHI
Position on HIKESHI

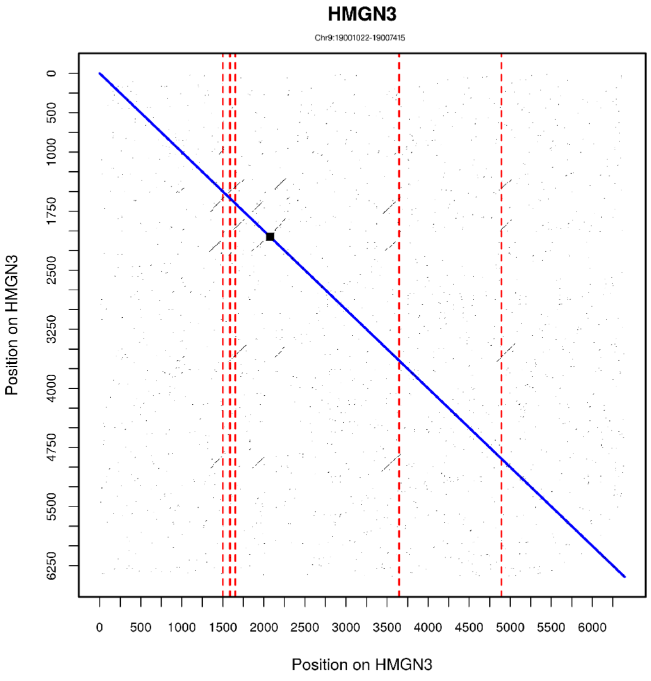
HMGN3
Chr9:19001022-19007415
Position on HMGN3
Position on HMGN3

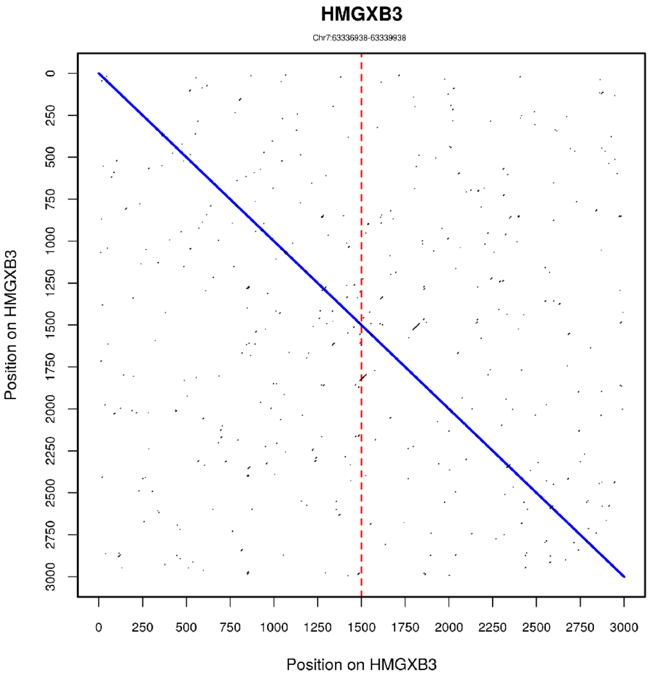
HMGXB3
Chr7:63336938-63339938
Position on HMGXB3
Position on HMGXB3

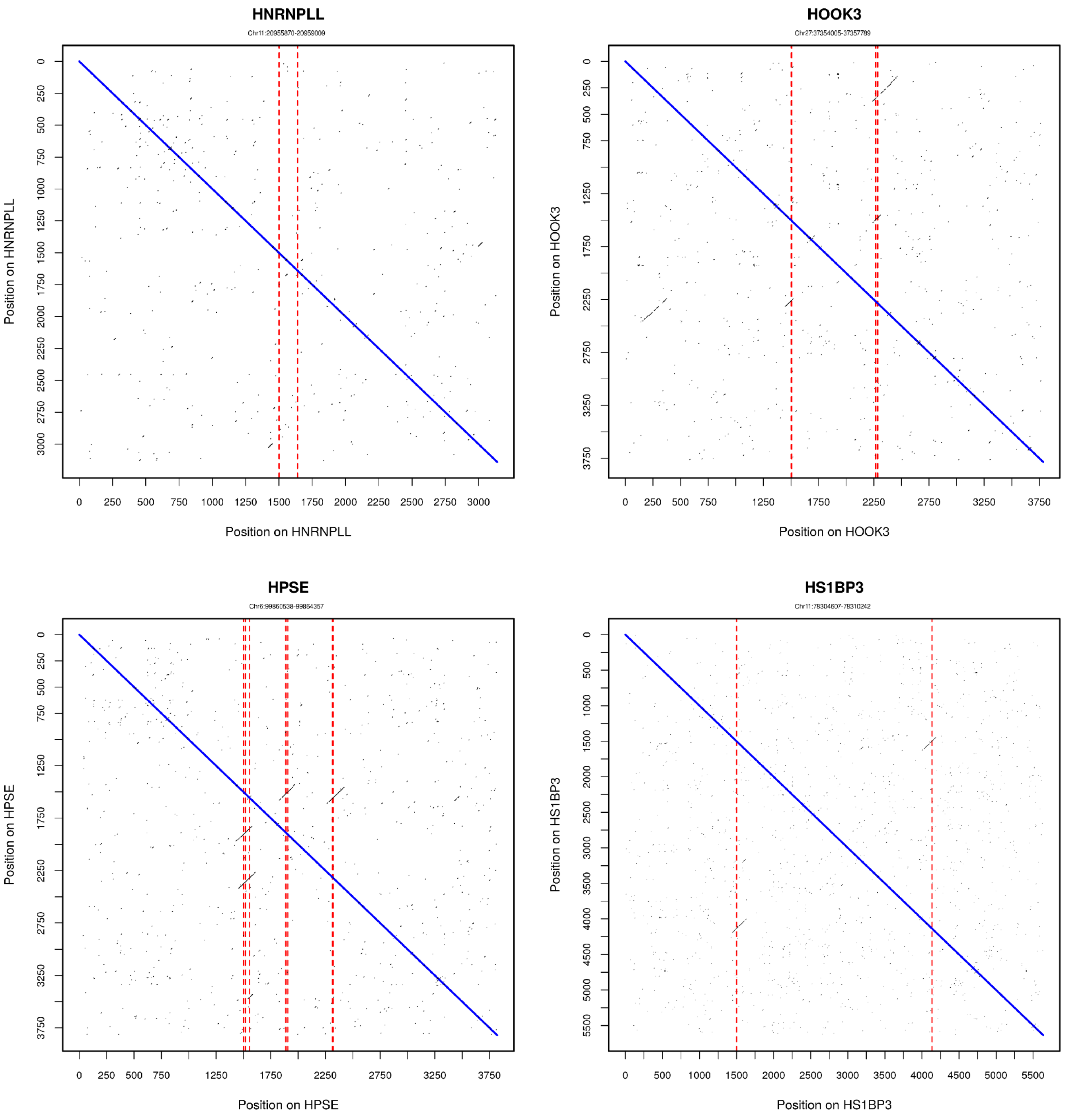
HNRNPLL
Chr11:20955870-20959009
Position on HNRNPLL
Position on HNRNPLL
HOOK3
Chr27:37354005-37357789
Position on HOOK3
Position on HOOK3
HPSE
Chr6:99860538-99864357
Position on HPSE
Position on HPSE
HS1BP3
Chr11:78304607-78310242
Position on HS1BP3
Position on HS1BP3

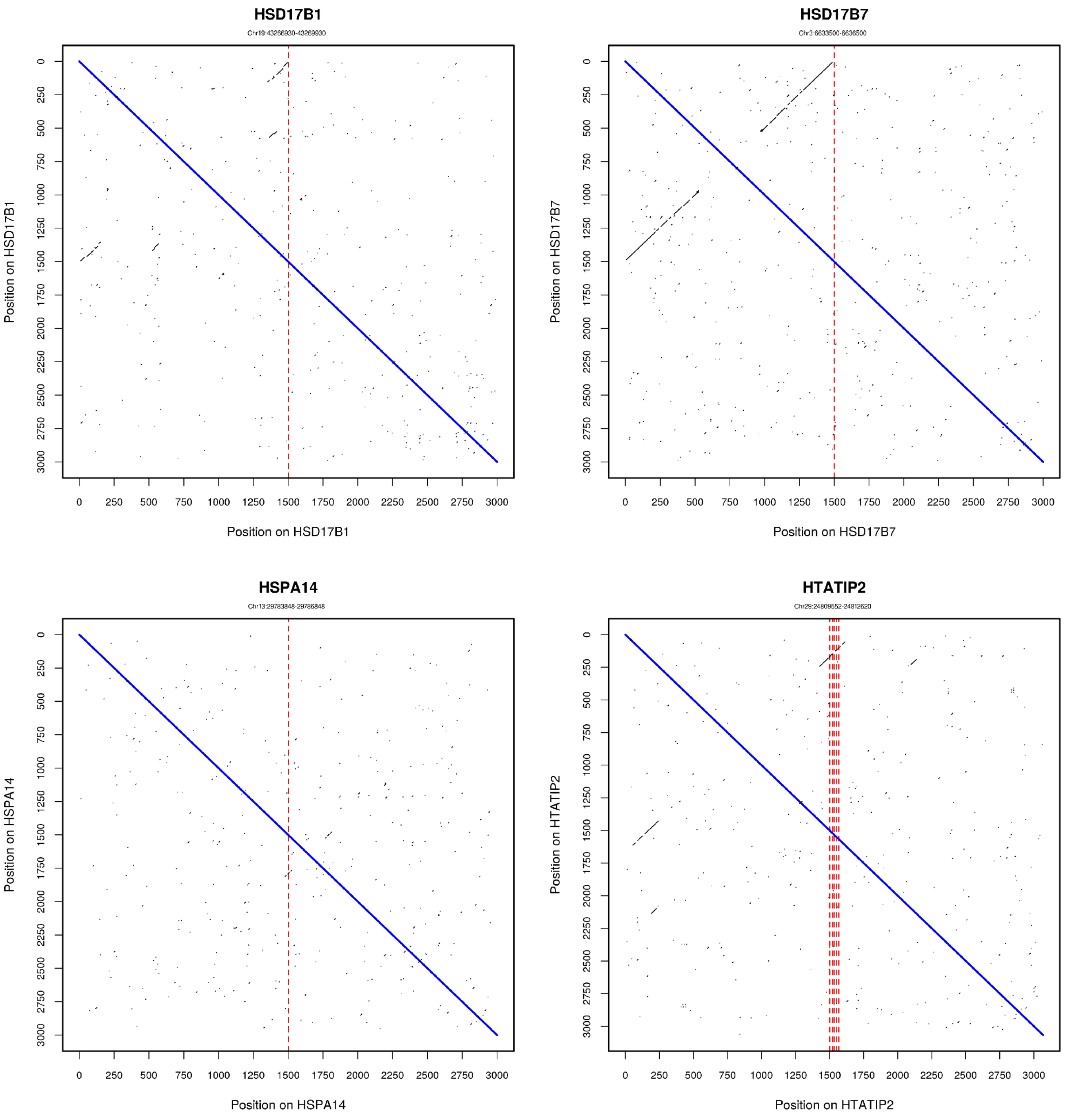
HSD17B1
Chr19:43266930-43269930
Position on HSD17B1
Position on HSD17B1
HSD17B7
Chr3:6633500-6636500
Position on HSD17B7
Position on HSD17B7
HSPA14
Chr13:29783848-29786848
Position on HSPA14
Position on HSPA14
HTATIP2
Chr29:24809552-24812620
Position on HTATIP2
Position on HTATIP2

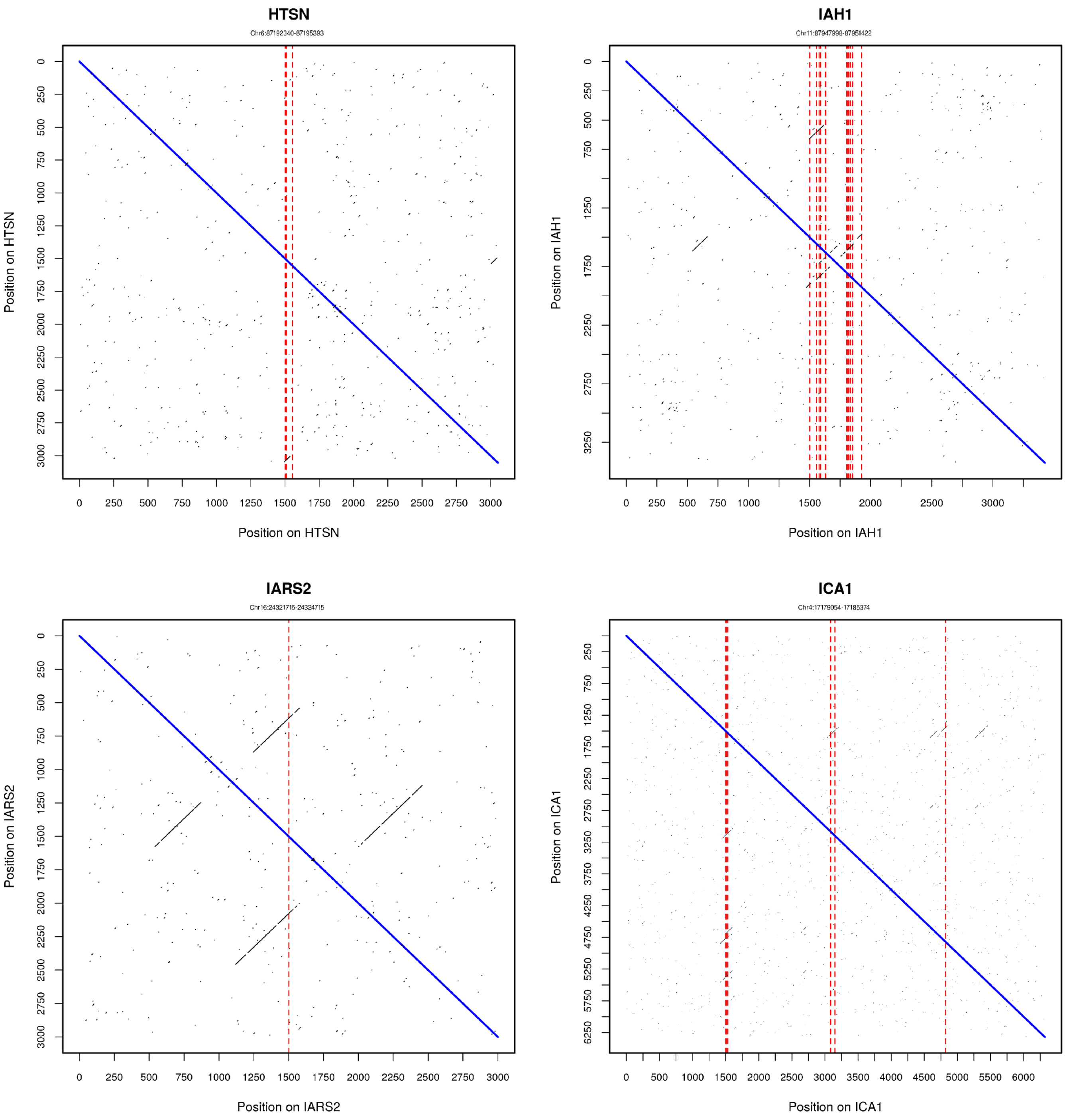
HTSN
Chr6:87192340-87195393
Position on HTSN
Position on HTSN
IAH1
Chr11:87947998-87951422
Position on IAH1
Position on IAH1
IARS2
Chr16:24321715-24324715
Position on IARS2
Position on IARS2
ICA1
Chr4:17179054-17185374
Position on ICA1
Position on ICA1

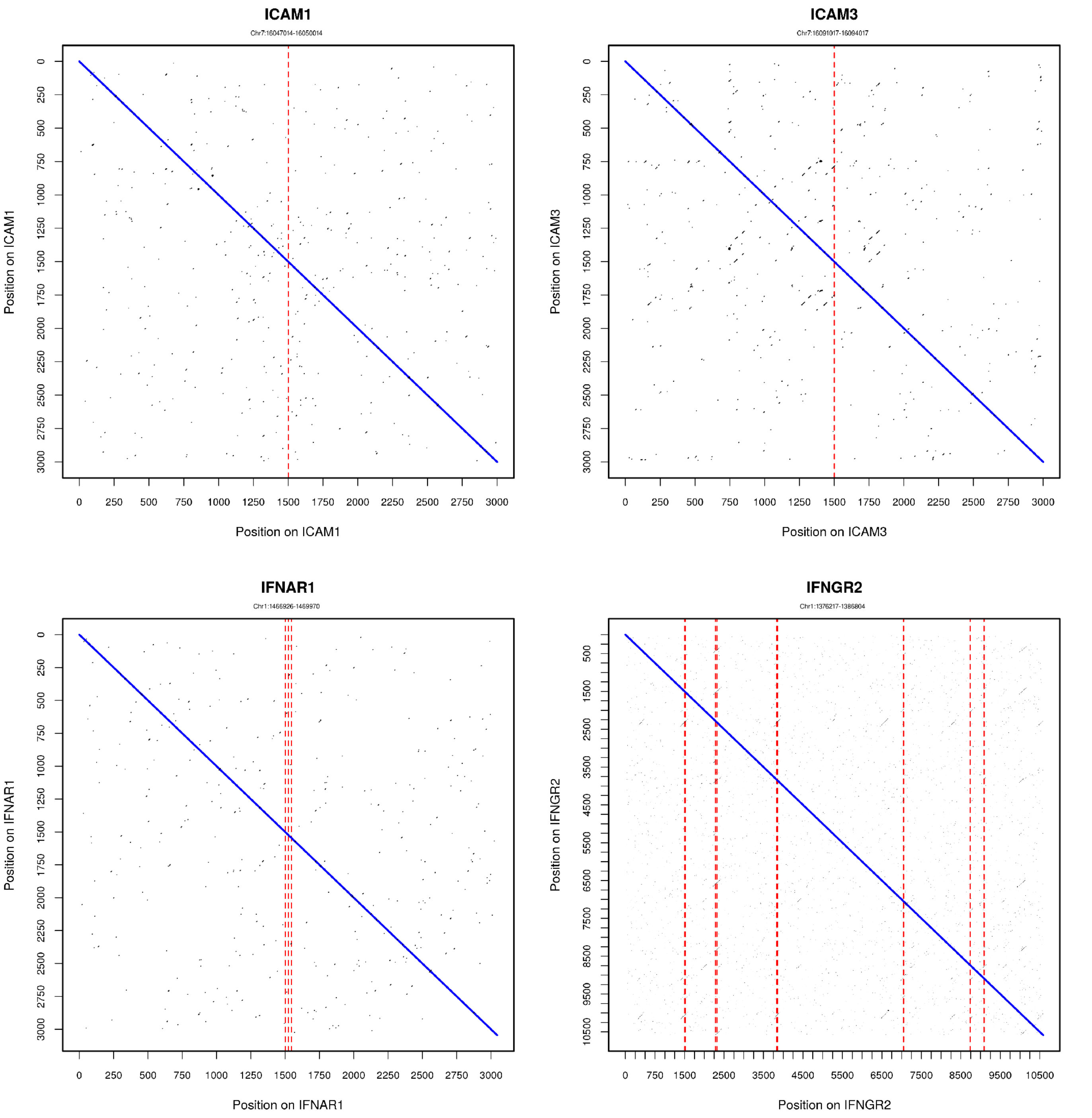
ICAM1
Chr7:16047014-16050014
Position on ICAM1
Position on ICAM1
ICAM3
Chr7:16091017-16094017
Position on ICAM3
Position on ICAM3
IFNAR1
Chr1:1466926-1469970
Position on IFNAR1
Position on IFNAR1
IFNGR2
Chr1:1376217-1386804
Position on IFNGR2
Position on IFNGR2

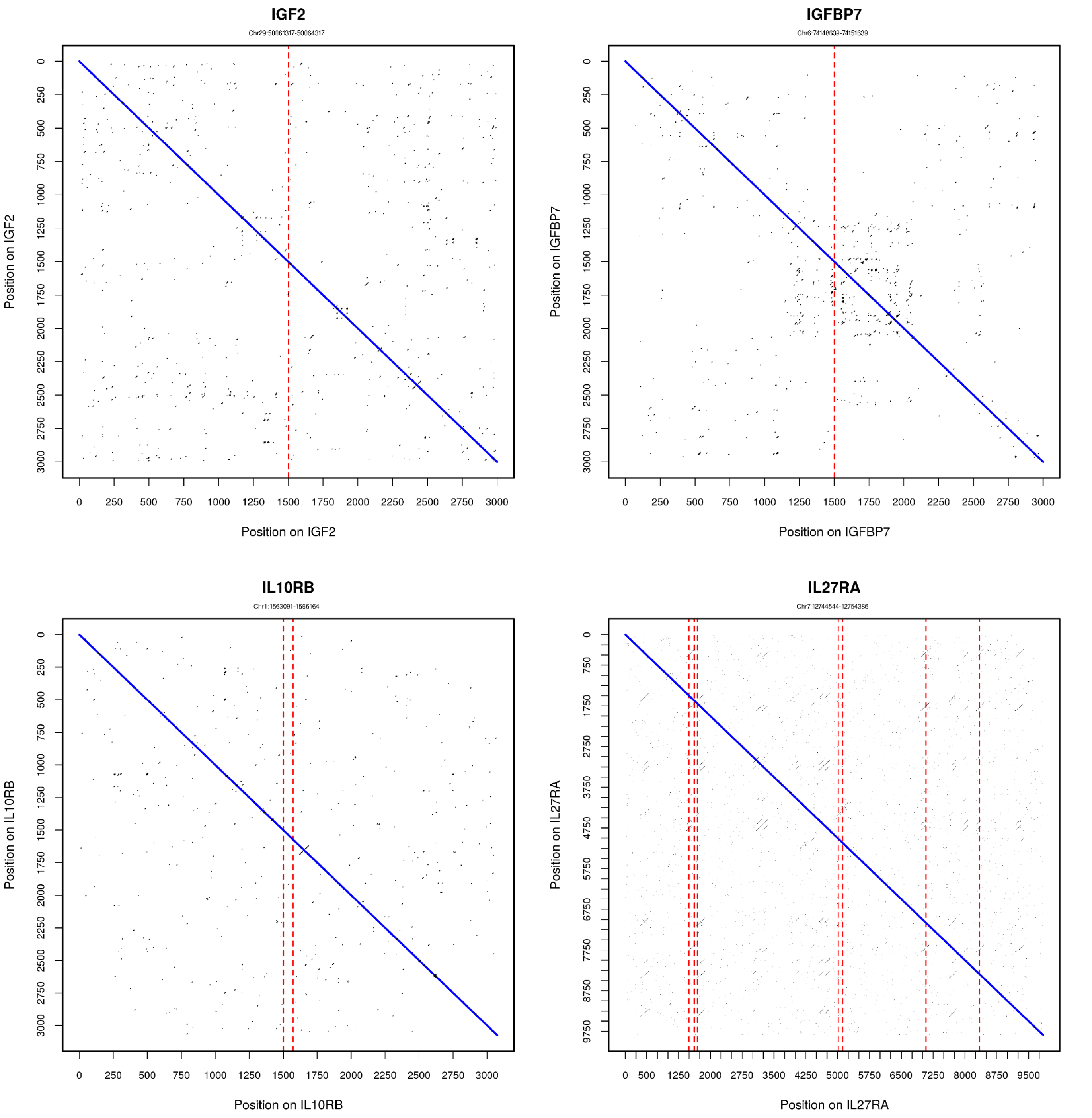

IGF2
Chr29:50061317-50064317
Position on IGF2
Position on IGF2
IGFBP7
Chr6:74148639-74151639
Position on IGFBP7
Position on IGFBP7
IL10RB
Chr1:1563091-1566164
Position on IL10RB
Position on IL10RB
IL27RA
Chr7:12744544-12754386
Position on IL27RA
Position on IL27RA

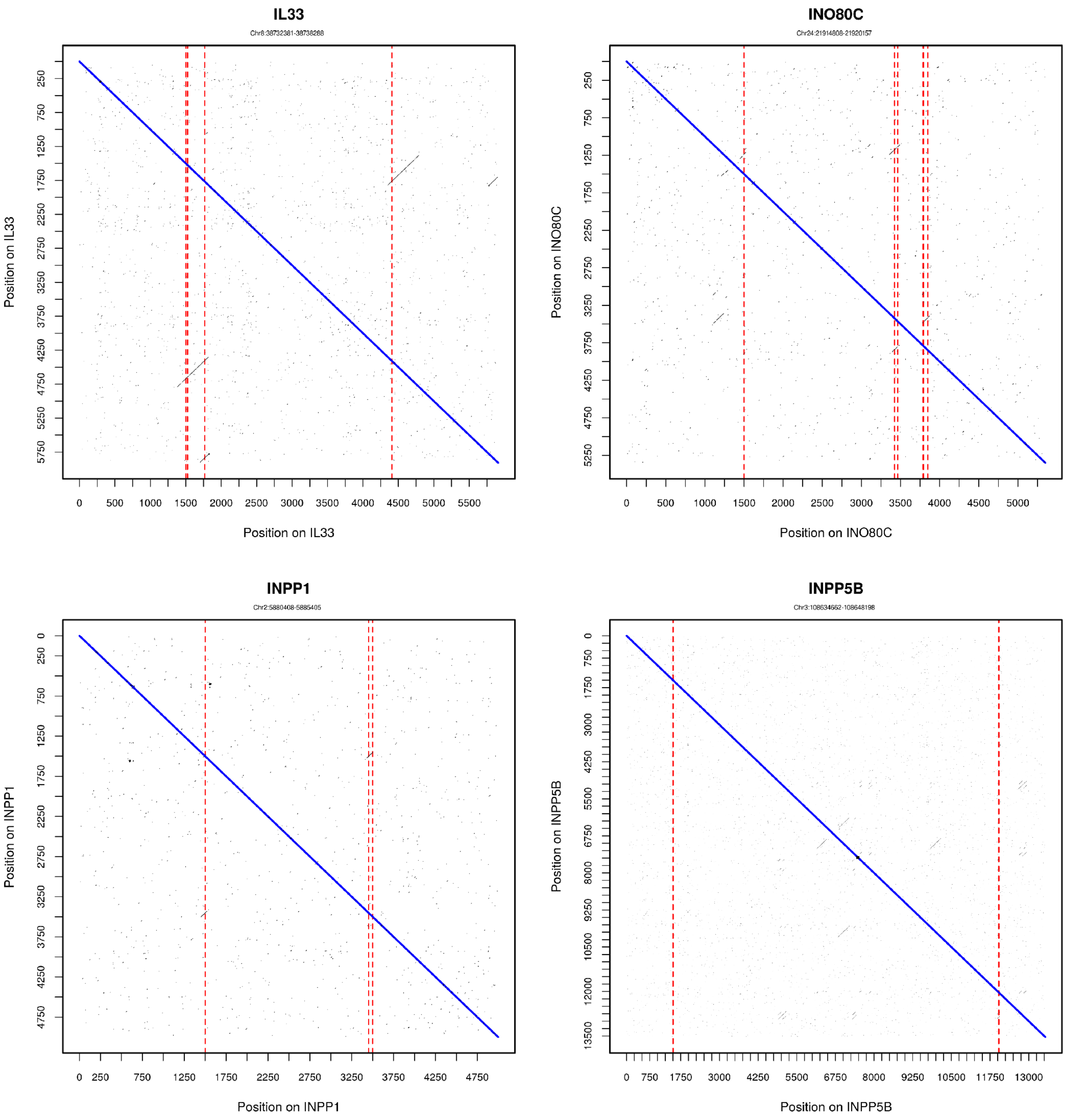
IL33
Chr8:38732381-38738288
Position on IL33
Position on IL33
INO80C
Chr24:21914808-21920157
Position on INO80C
Position on INO80C
INPP1
Chr2:5880408-5885405
Position on INPP1
Position on INPP1
INPP5B
Chr3:108634662-108648198
Position on INPP5B
Position on INPP5B

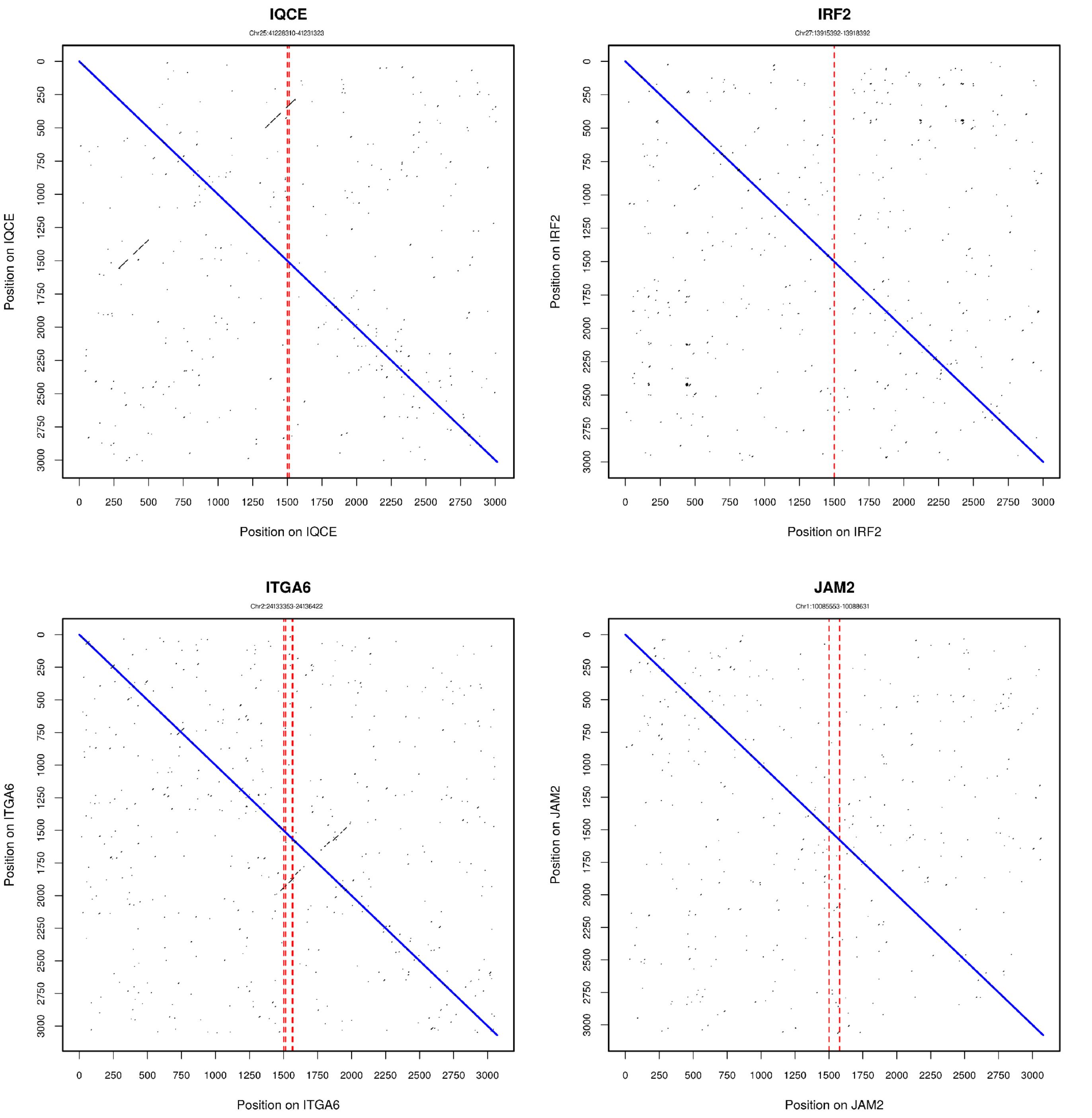

IQCE
Chr25:41228310-41231323
Position on IQCE
Position on IQCE
IRF2
Chr27:13915392-13918392
Position on IRF2
Position on IRF2
ITGA6
Chr2:24133353-24136422
Position on ITGA6
Position on ITGA6
JAM2
Chr1:10085553-10088631
Position on JAM2
Position on JAM2

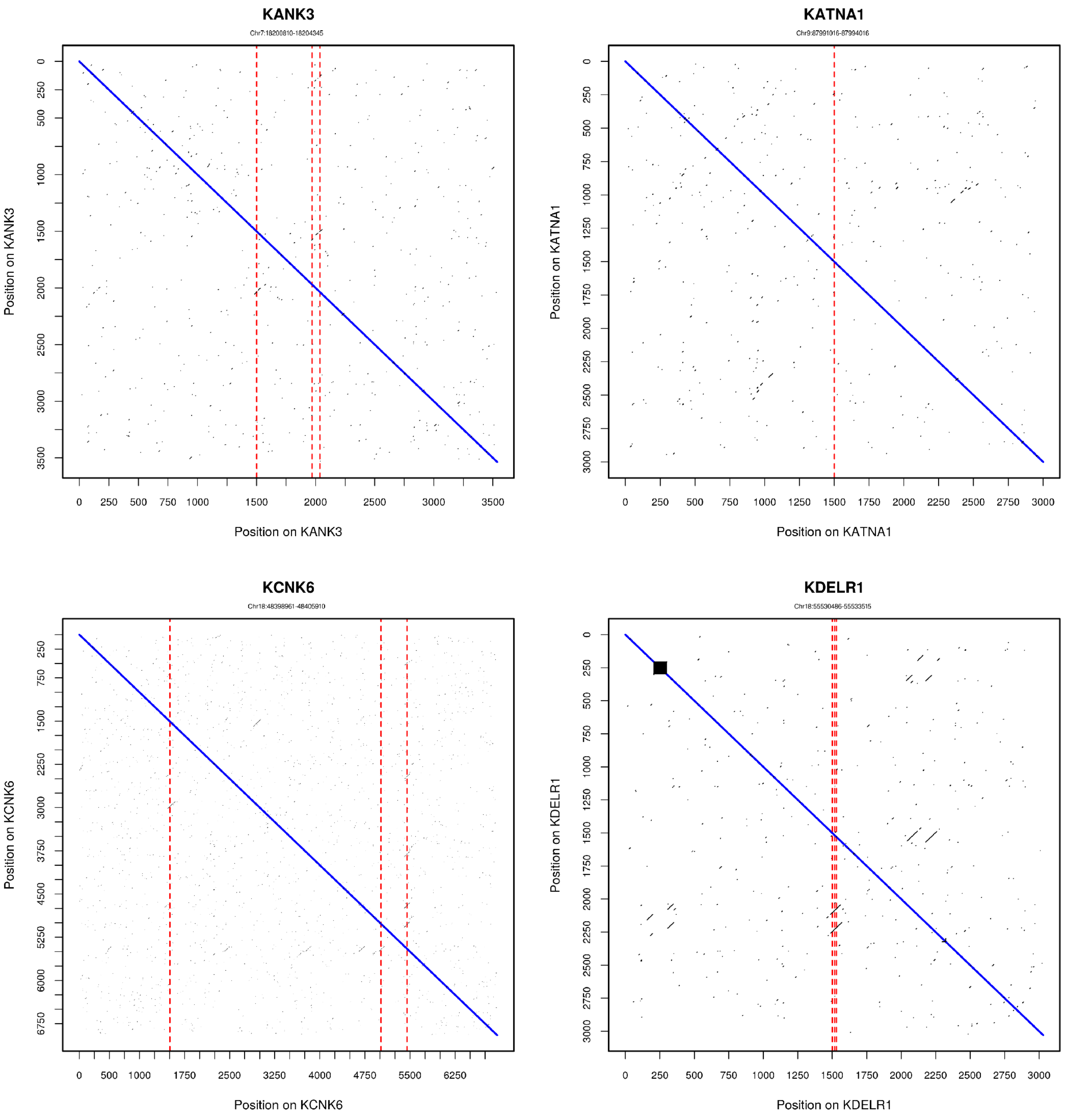
KANK3
Chr7:18200810-18204345
Position on KANK3
Position on KANK3
KATNA1
Chr9:87991016-87994016
Position on KATNA1
Position on KATNA1
KCNK6
Chr18:48398961-48405910
Position on KCNK6
Position on KCNK6
KDELR1
Chr18:55530486-55533515
Position on KDELR1
Position on KDELR1

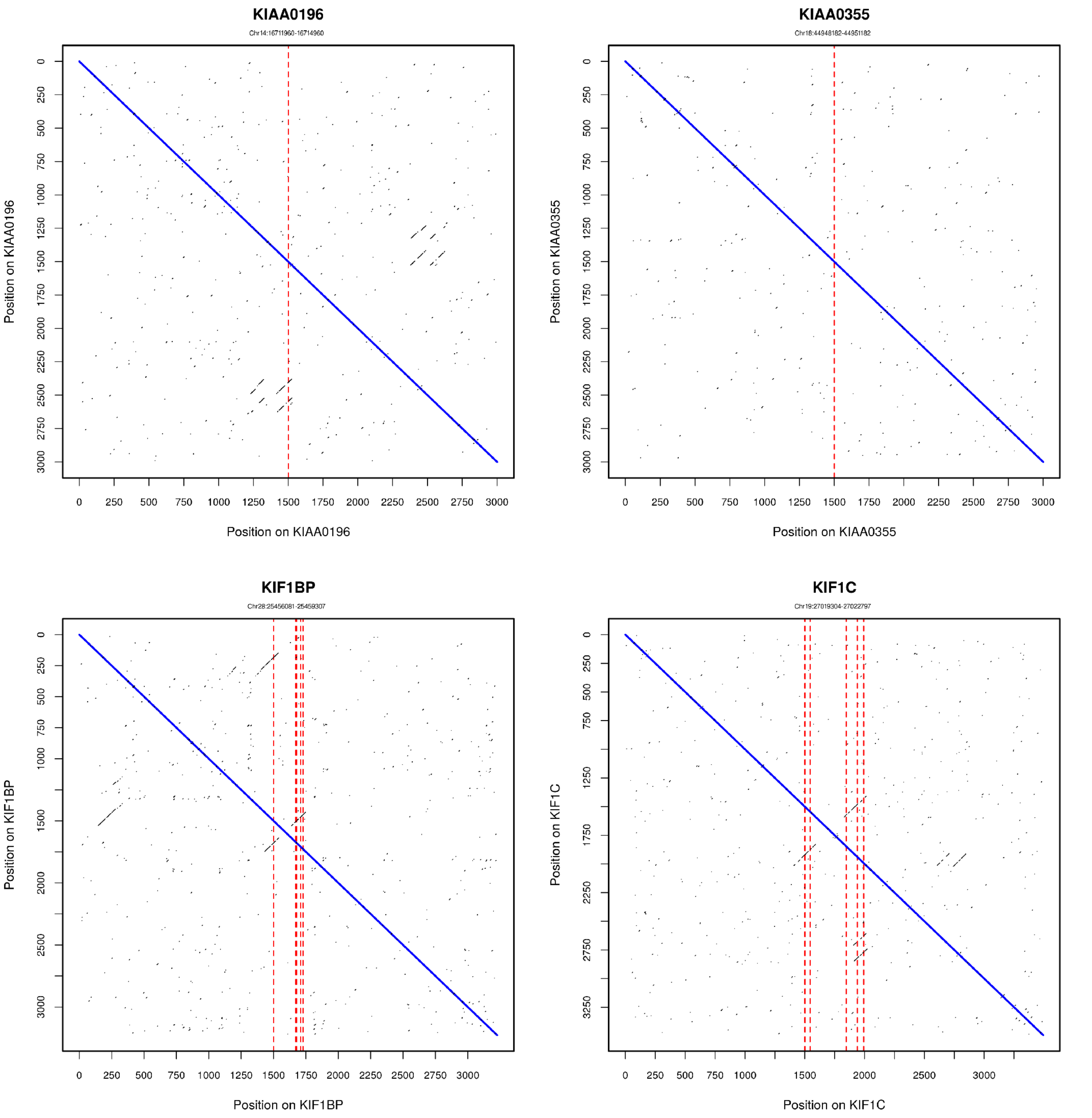
KIAA0196
Chr14:16711960-16714960
Position on KIAA0196
Position on KIAA0196
KIAA0355
Chr18:44948182-44951182
Position on KIAA0355
Position on KIAA0355
KIF1BP
Chr28:25456081-25459307
Position on KIF1BP
Position on KIF1BP
KIF1C
Chr19:27019304-27022797
Position on KIF1C
Position on KIF1C

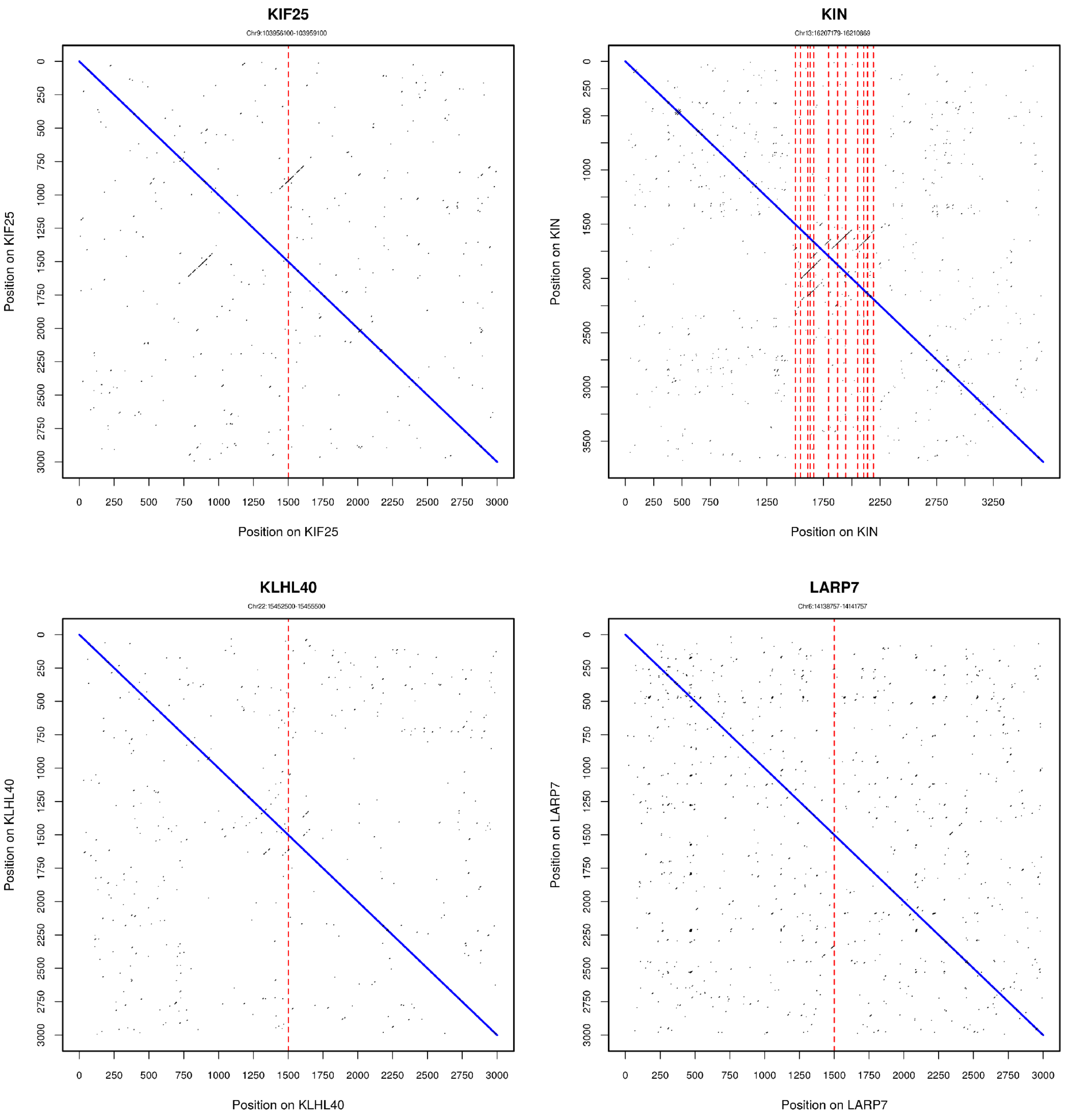
KIF25
Chr9:103956100-103959100
Position on KIF25
Position on KIF25
KIN
Chr13:16207179-16210869
Position on KIN
Position on KIN
KLHL40
Chr22:15452500-15455500
Position on KLHL40
Position on KLHL40
LARP7
Chr6:14138757-14141757
Position on LARP7
Position on LARP7

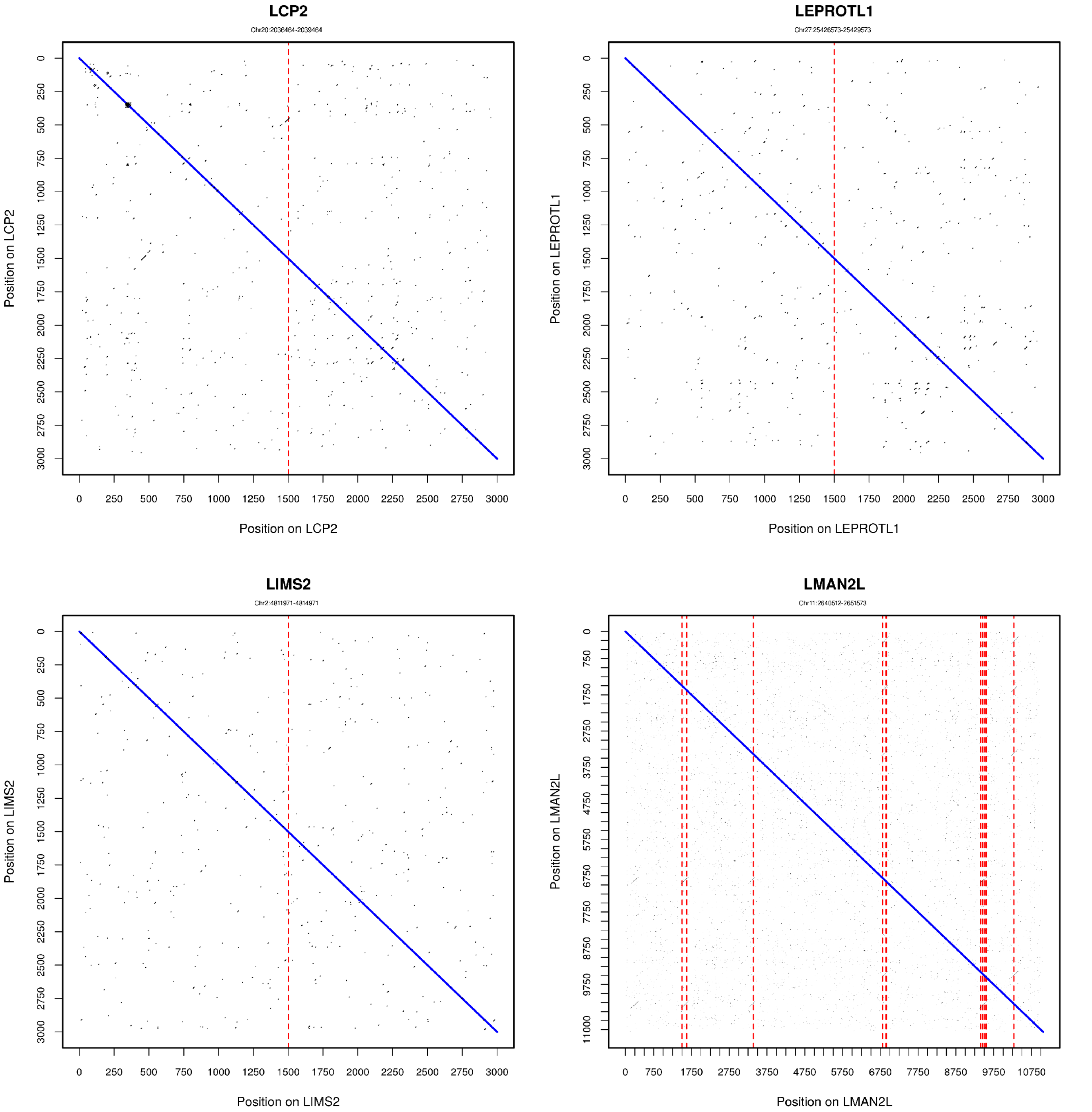

LCP2
Chr20:2036464-2039464
Position on LCP2
Position on LCP2
LEPROTL1
Chr27:25426573-25429573
Position on LEPROTL1
Position on LEPROTL1
LIMS2
Chr2:4811971-4814971
Position on LIMS2
Position on LIMS2
LMAN2L
Chr11:2640512-2651573
Position on LMAN2L
Position on LMAN2L

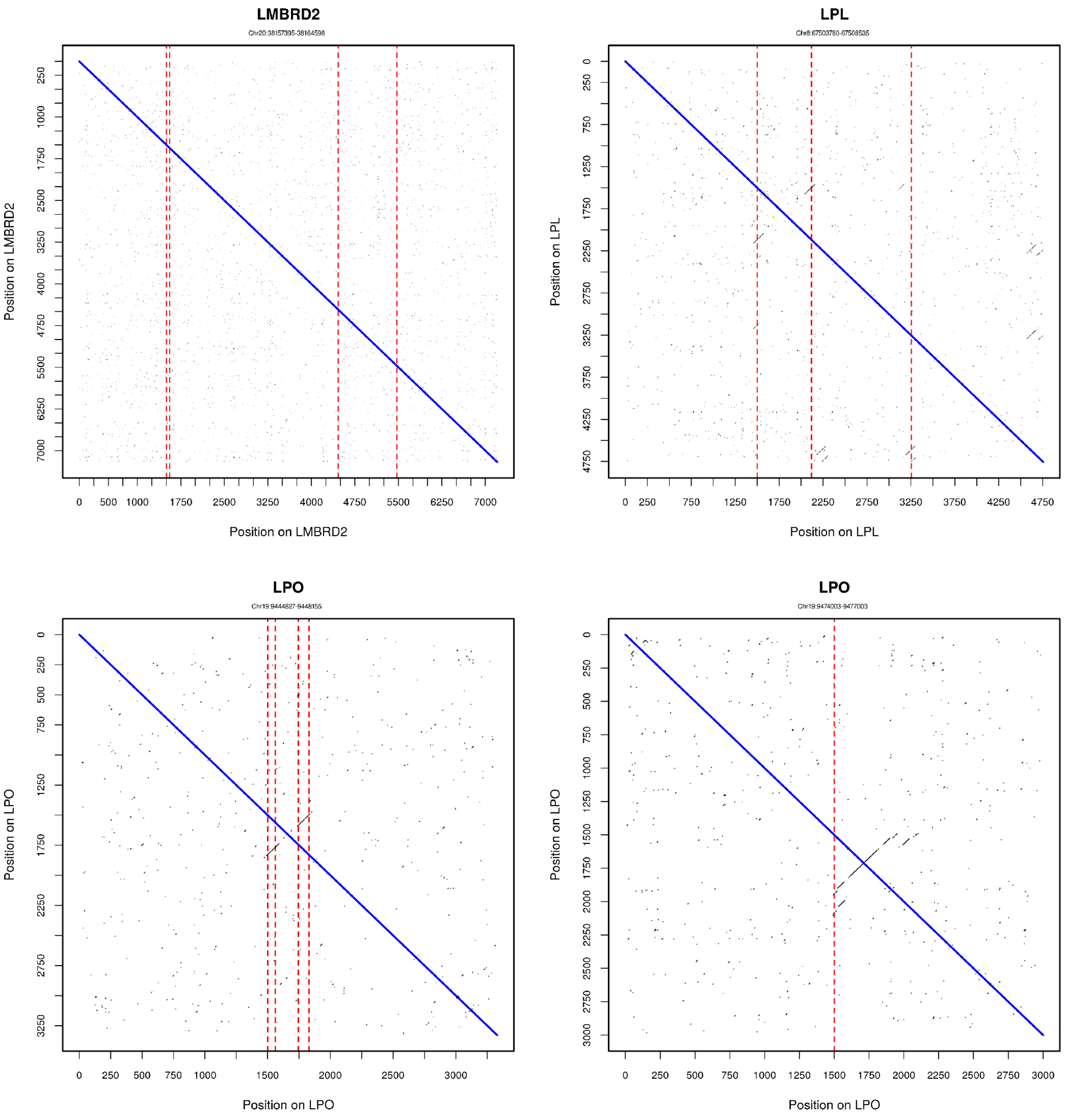

LMBRD2
Chr20:38157395-38164598
Position on LMBRD2
Position on LMBRD2
LPL
Chr8:67503780-67508535
Position on LPL
Position on LPL
LPO
Chr19:9444827-9448155
Position on LPO
Position on LPO
LPO
Chr19:9474003-9477003
Position on LPO
Position on LPO

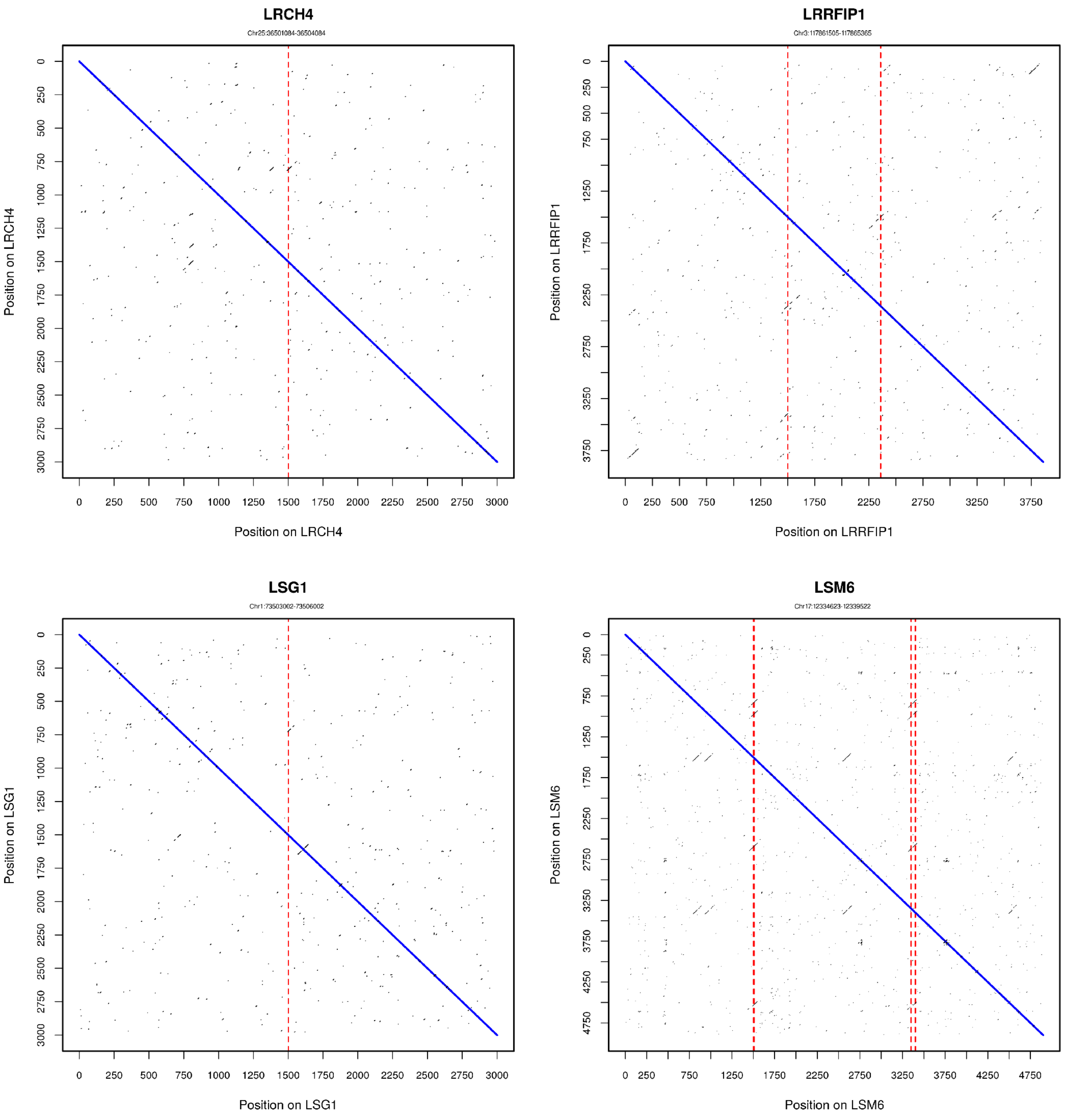

LRCH4
Chr25:36501084-36504084
Position on LRCH4
Position on LRCH4
LRRFIP1
Chr3:117861505-117865365
Position on LRRFIP1
Position on LRRFIP1
LSG1
Chr1:73503002-73506002
Position on LSG1
Position on LSG1
LSM6
Chr17:12334623-12339522
Position on LSM6
Position on LSM6

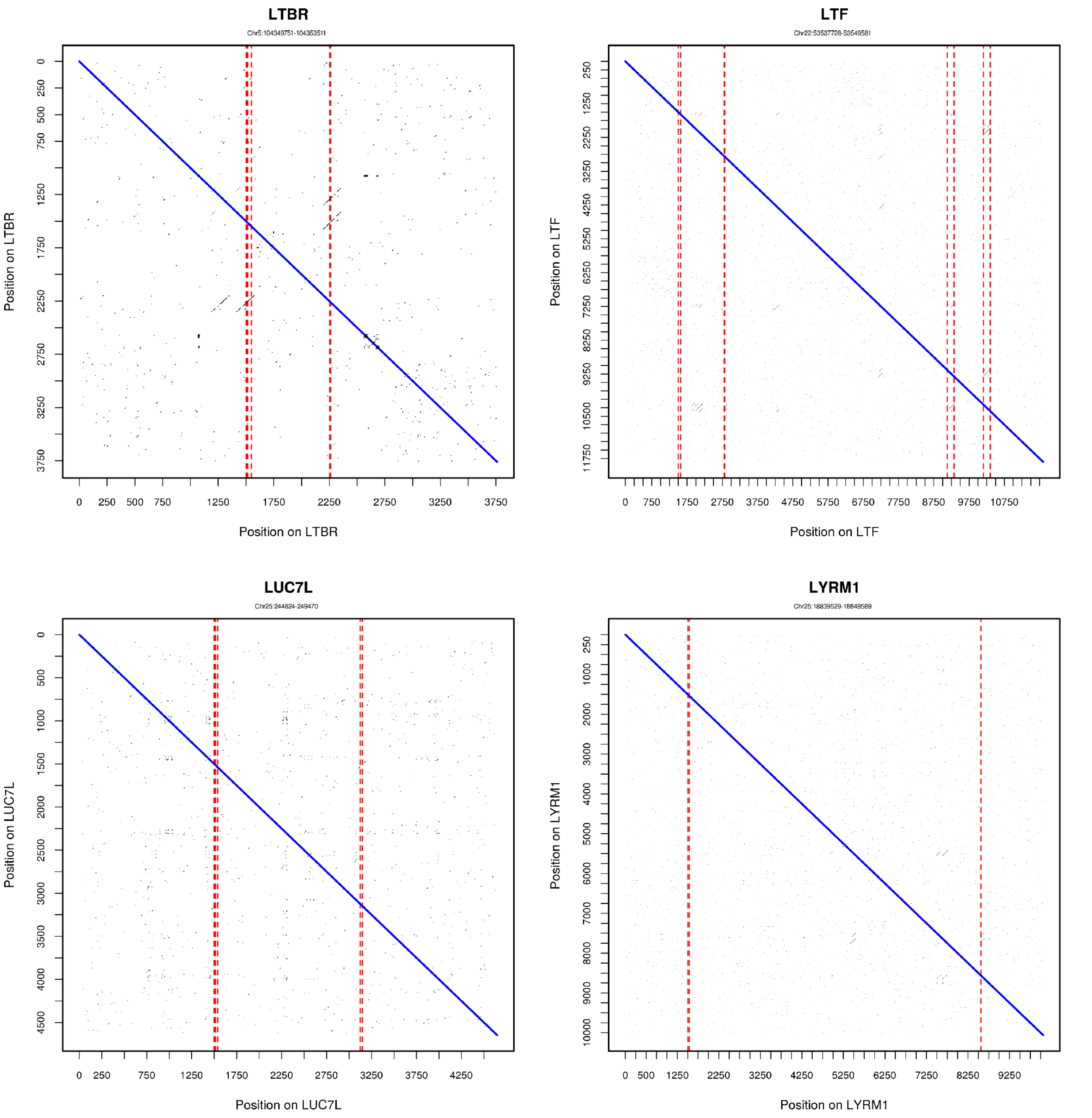

LTBR
Chr5:104349751-104353511
Position on LTBR
Position on LTBR
LTF
Chr22:53537728-53549581
Position on LTF
Position on LTF
LUC7L
Chr25:244824-249470
Position on LUC7L
Position on LUC7L
LYRM1
Chr25:18839529-18849589
Position on LYRM1
Position on LYRM1

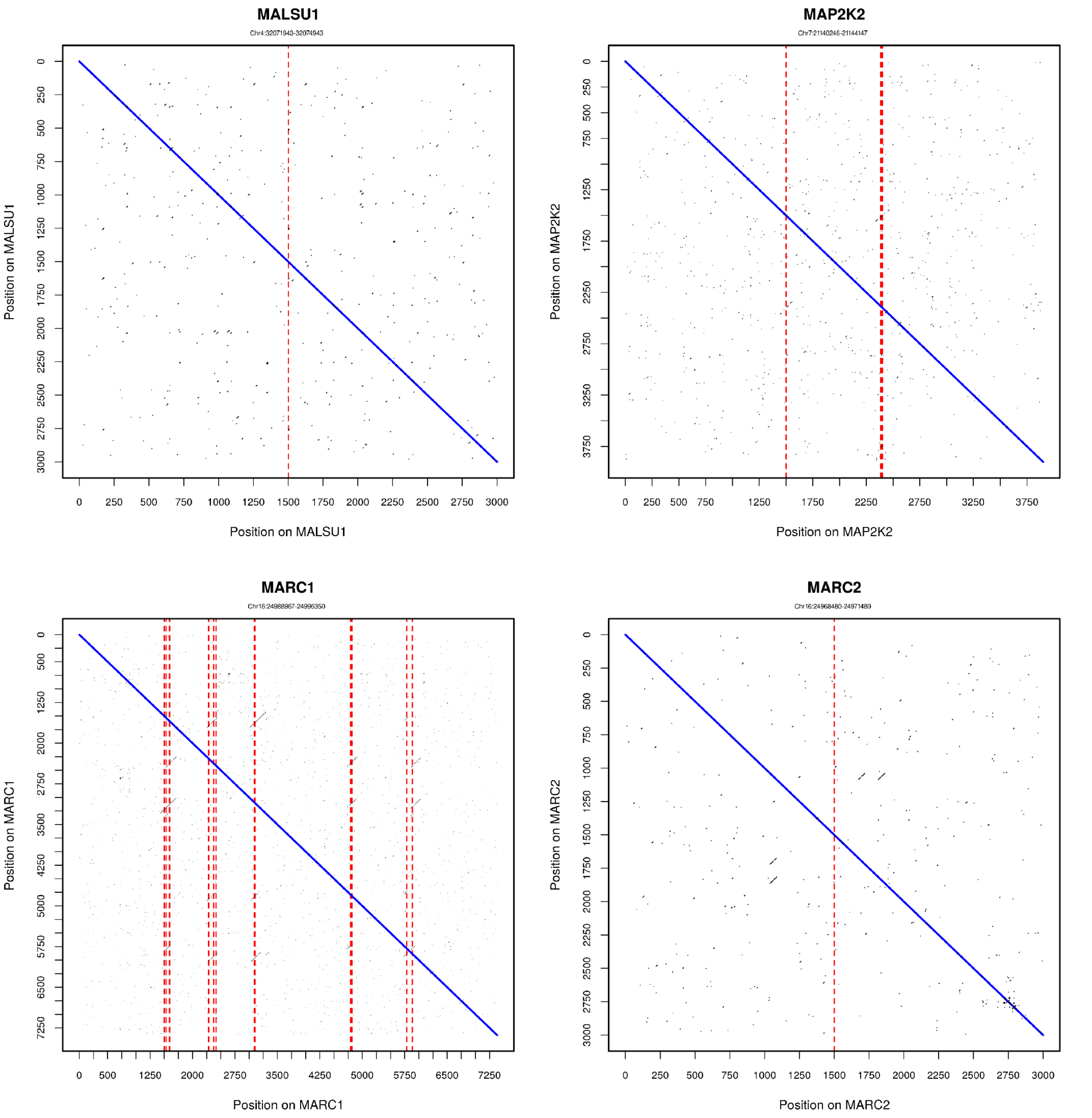
MALSU1
Chr4:32071943-32074943
Position on MALSU1
Position on MALSU1
MAP2K2
Chr7:21140246-21144147
Position on MAP2K2
Position on MAP2K2
MARC1
Chr16:24988967-24996350
Position on MARC1
Position on MARC1
MARC2
Chr16:24968480-24971480
Position on MARC2
Position on MARC2

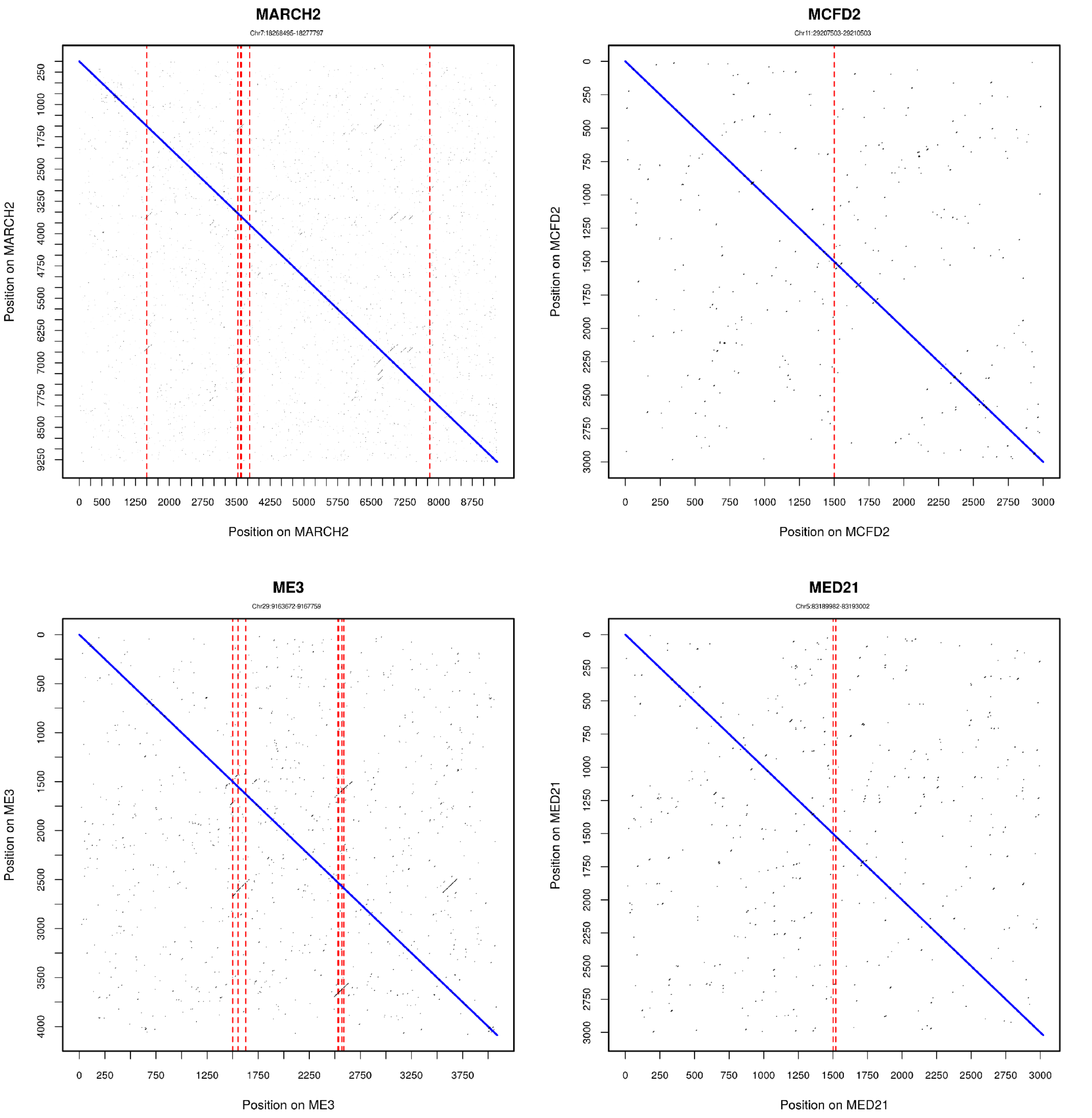
MARCH2
Chr7:18268495-18277797
Position on MARCH2
Position on MARCH2
MCFD2
Chr11:29207503-29210503
Position on MCFD2
Position on MCFD2
ME3
Chr29:9163672-9167759
Position on ME3
Position on ME3
MED21
Chr5:83189982-83193002
Position on MED21
Position on MED21

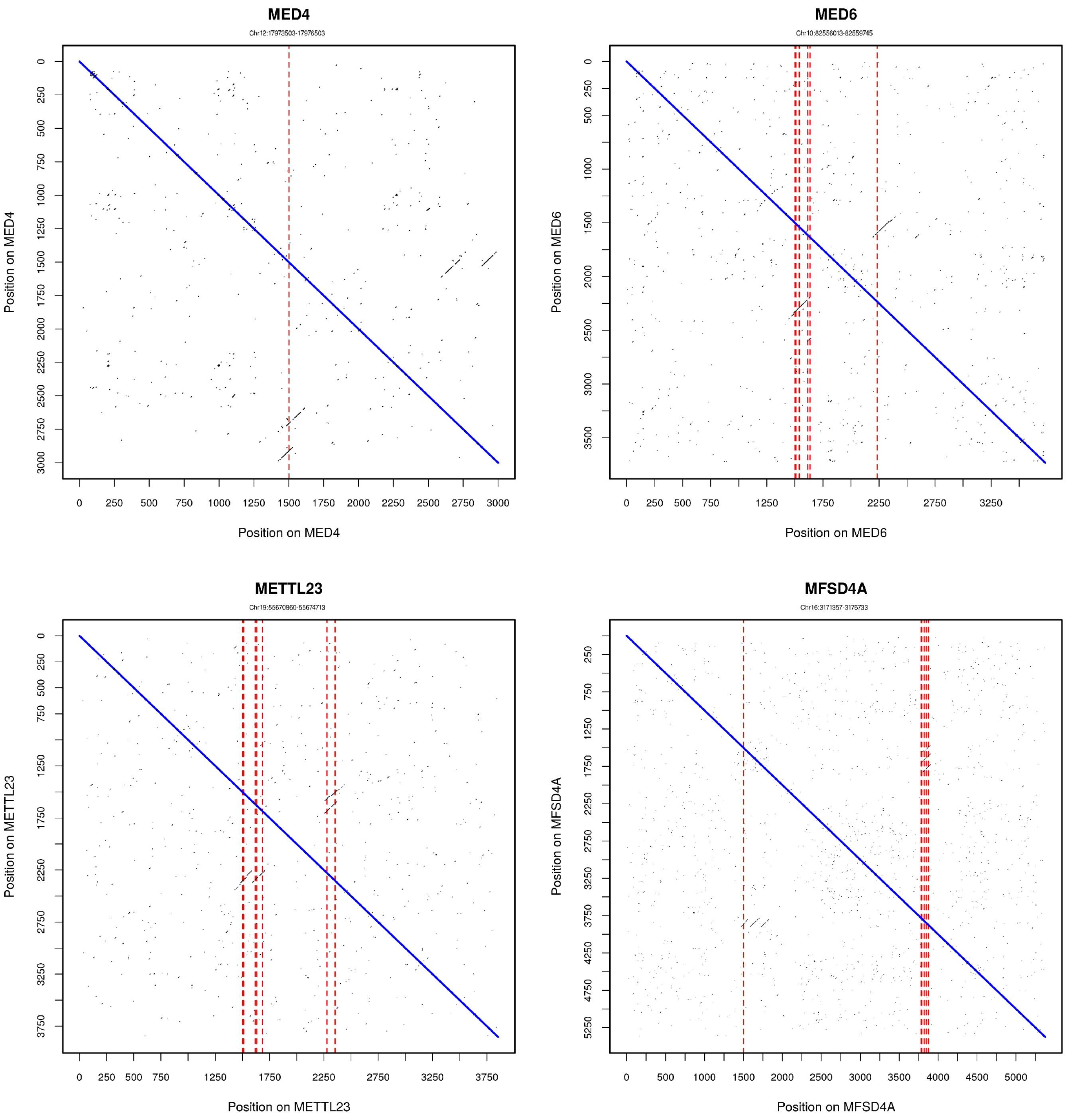
MED4
Chr12:17973503-17976503
Position on MED4
Position on MED4
MED6
Chr10:82556013-82559745
Position on MED6
Position on MED6
METTL23
Chr19:55670860-55674713
Position on METTL23
Position on METTL23
MFSD4A
Chr16:3171357-3176733
Position on MFSD4A
Position on MFSD4A

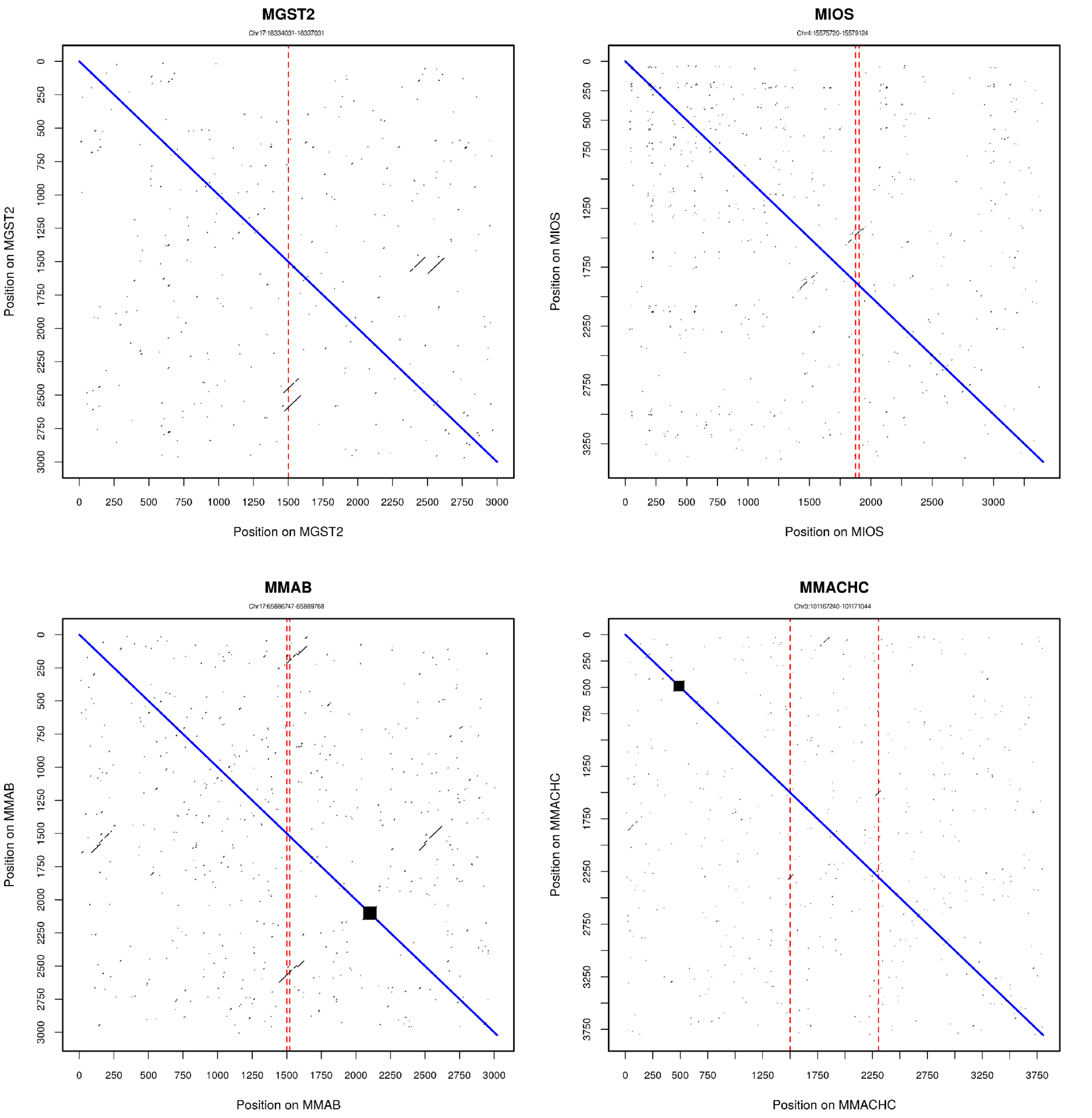
MGST2
Chr17:18334031-18337031
Position on MGST2
Position on MGST2
MIOS
Chr4:15575720-15579124
Position on MIOS
Position on MIOS
MMAB
Chr17:65886747-65889768
Position on MMAB
Position on MMAB
MMACHC
Chr3:101167240-101171044
Position on MMACHC
Position on MMACHC

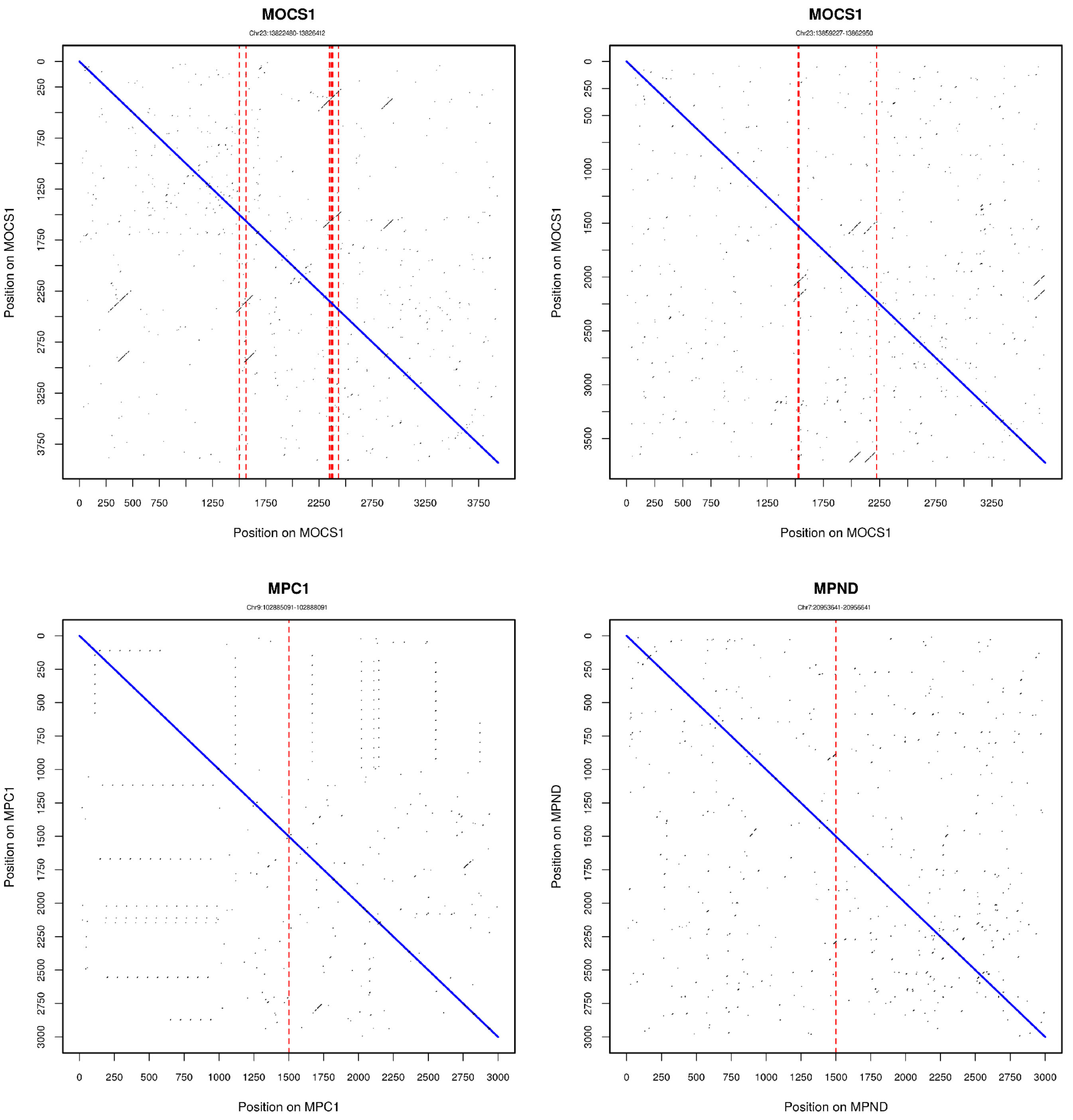
MOCS1
Chr23:13822480-13826412
Position on MOCS1
Position on MOCS1
MOCS1
Chr23:13859227-13862950
Position on MOCS1
Position on MOCS1
MPC1
Chr9:102885091-102888091
Position on MPC1
Position on MPC1
MPND
Chr7:20953641-20956641
Position on MPND
Position on MPND

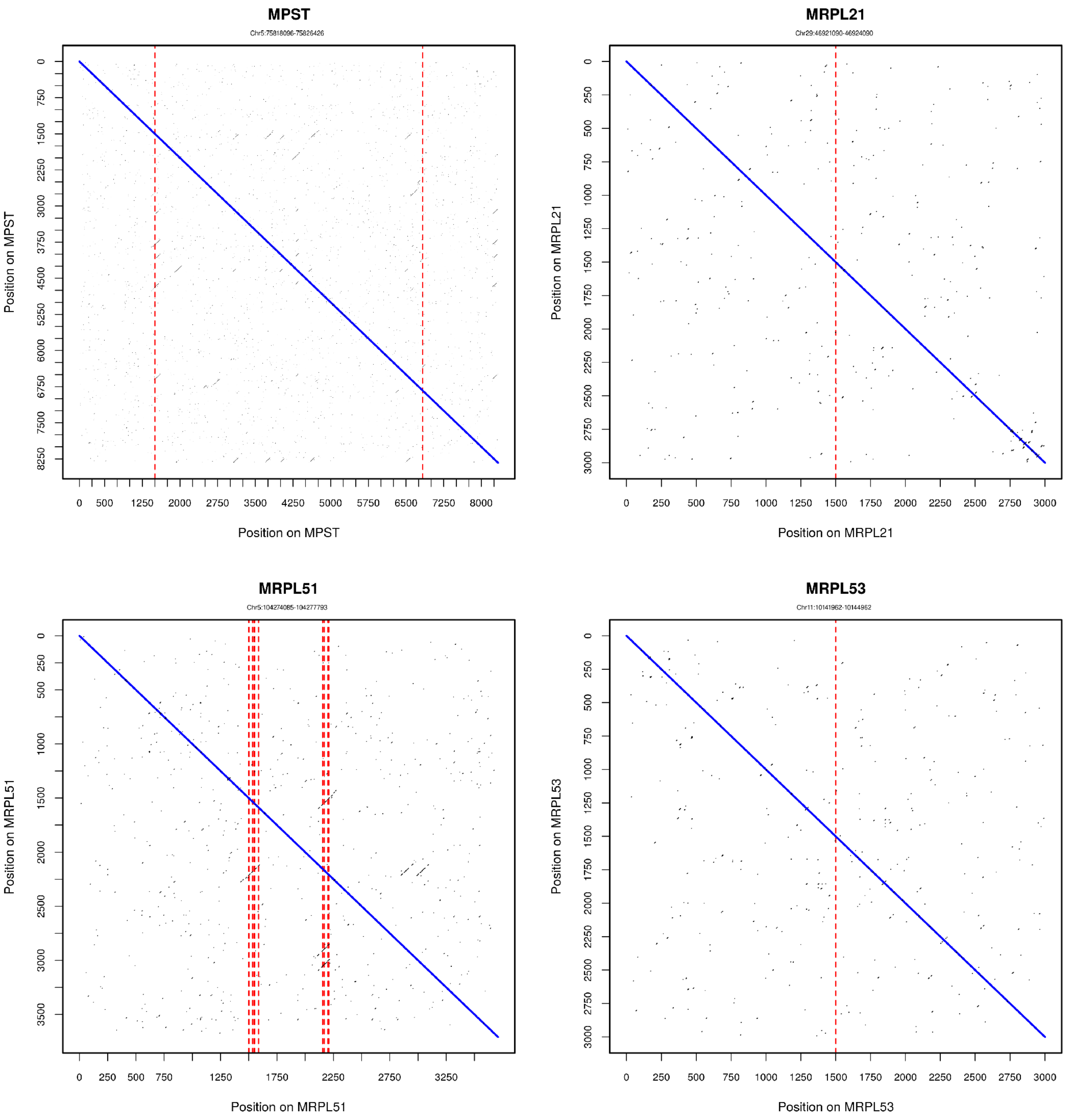
MPST
Chr5:75818096-75826426
Position on MPST
Position on MPST
MRPL21
Chr29:46921090-46924090
Position on MRPL21
Position on MRPL21
MRPL51
Chr5:104274085-104277793
Position on MRPL51
Position on MRPL51
MRPL53
Chr11:10141962-10144962
Position on MRPL53
Position on MRPL53

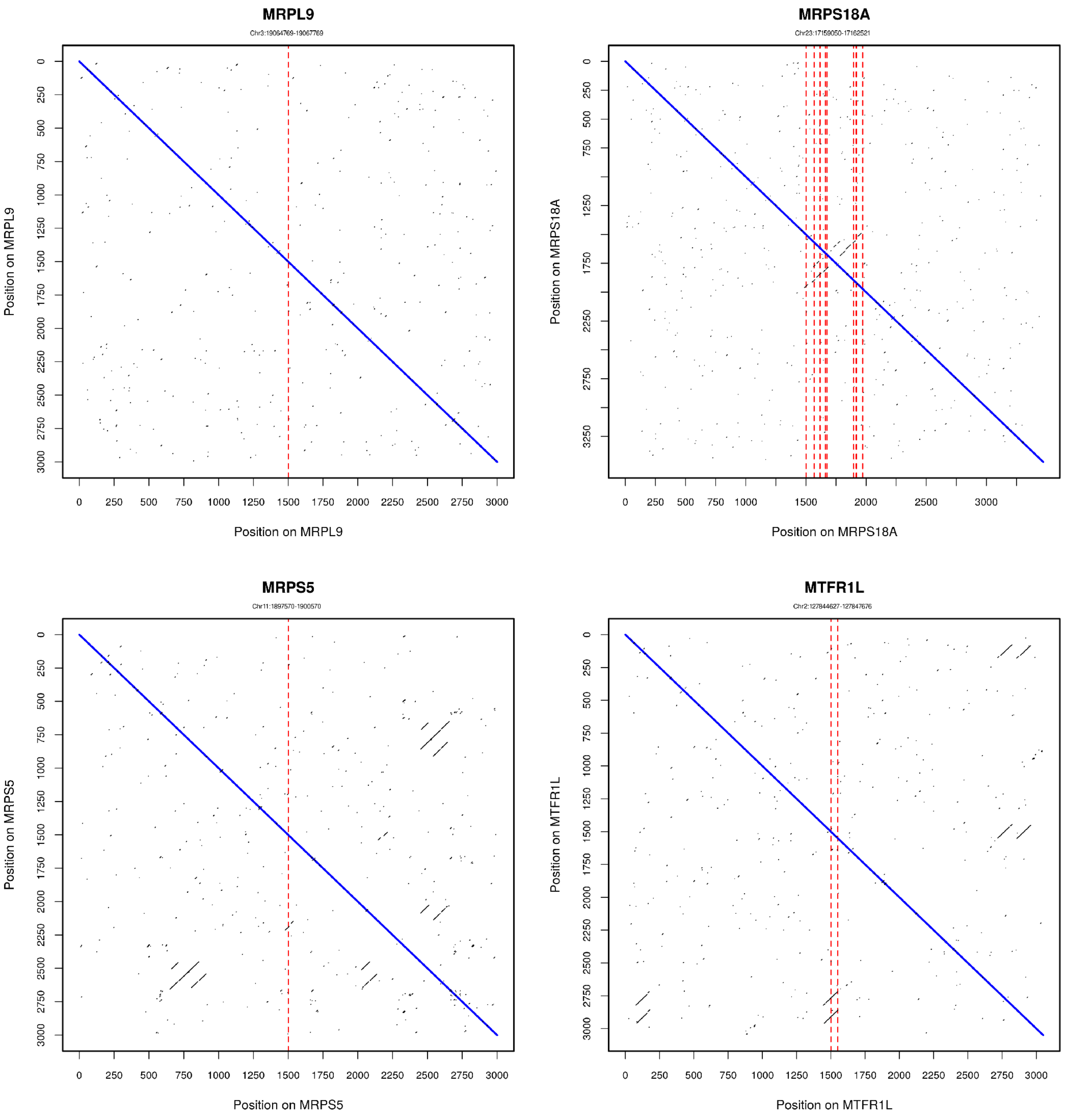

MRPL9
Chr3:19064769-19067769
Position on MRPL9
Position on MRPL9
MRPS18A
Chr23:17159050-17162521
Position on MRPS18A
Position on MRPS18A
MRPS5
Chr11:1897570-1900570
Position on MRPS5
Position on MRPS5
MTFR1L
Chr2:127844627-127847676
Position on MTFR1L
Position on MTFR1L

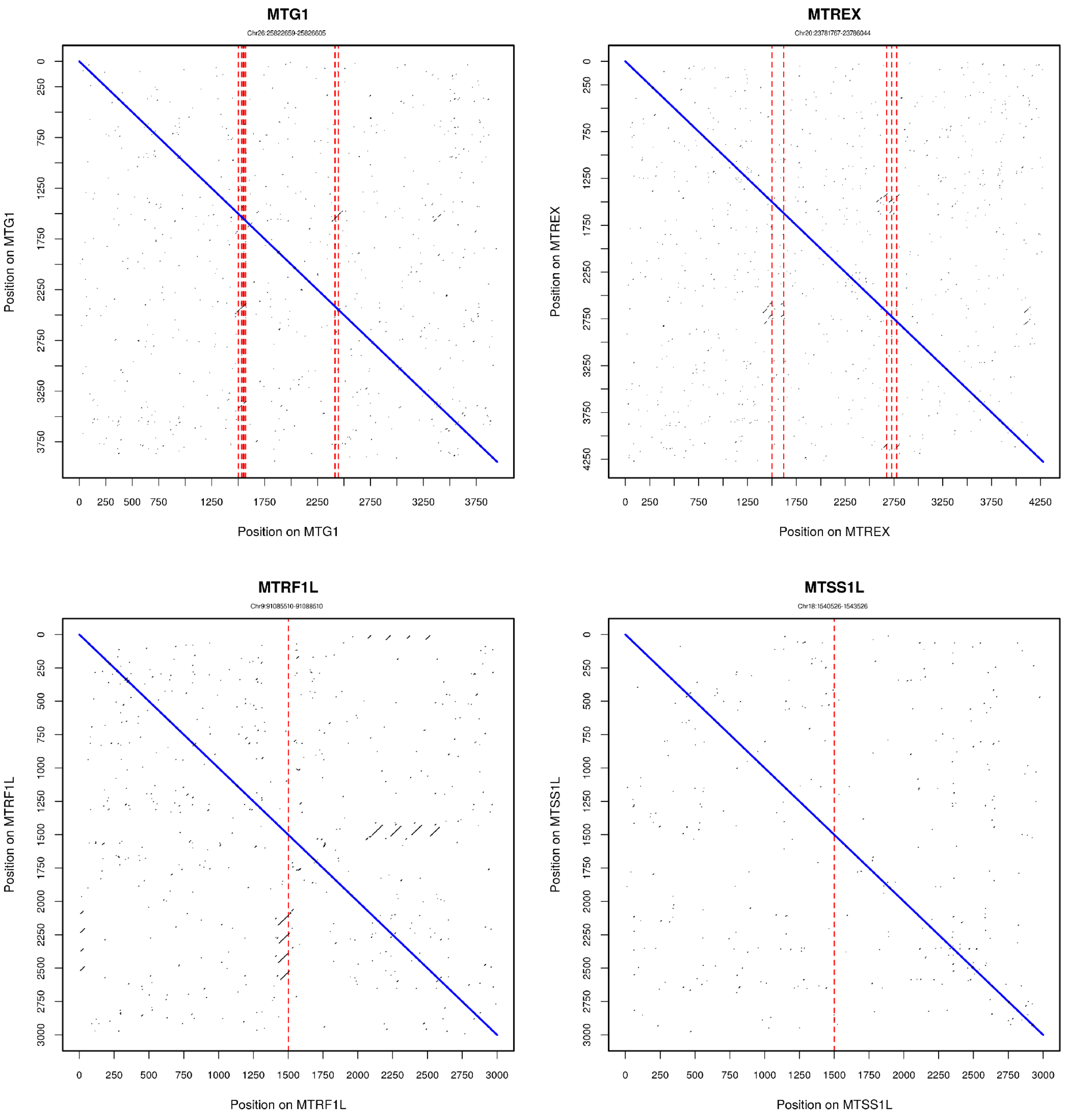

MTG1
Chr26:25822659-25826605
Position on MTG1
Position on MTG1
MTREX
Chr20:23781767-23786044
Position on MTREX
Position on MTREX
MTRF1L
Chr9:91085510-91088510
Position on MTRF1L
Position on MTRF1L
MTSS1L
Chr18:1540526-1543526
Position on MTSS1L
Position on MTSS1L

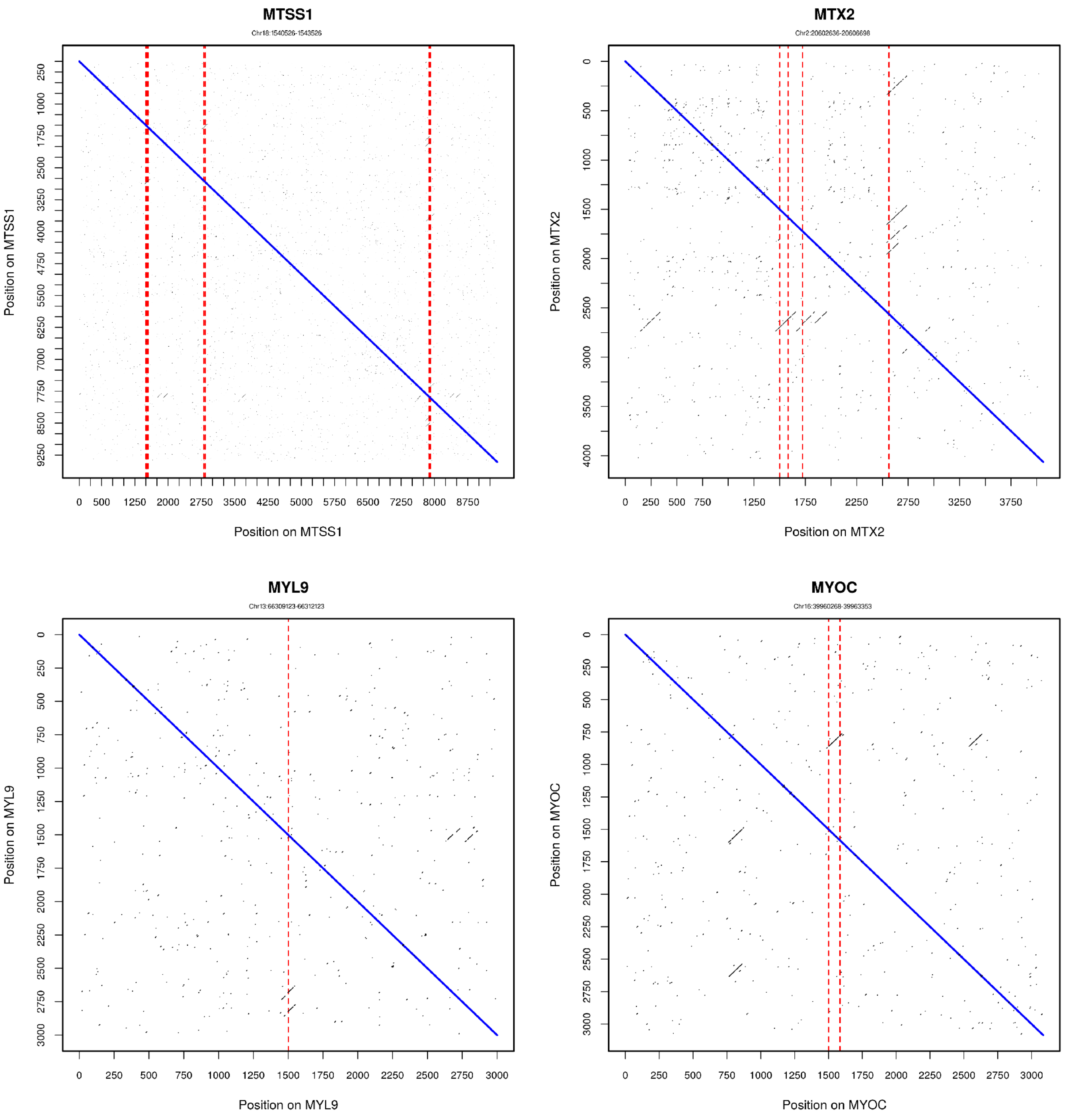

MTSS1
Chr18:1540526-1543526
Position on MTSS1
Position on MTSS1
MTX2
Chr2:20602636-20606698
Position on MTX2
Position on MTX2
MYL9
Chr13:66309123-66312123
Position on MYL9
Position on MYL9
MYOC
Chr16:39960268-39963353
Position on MYOC
Position on MYOC

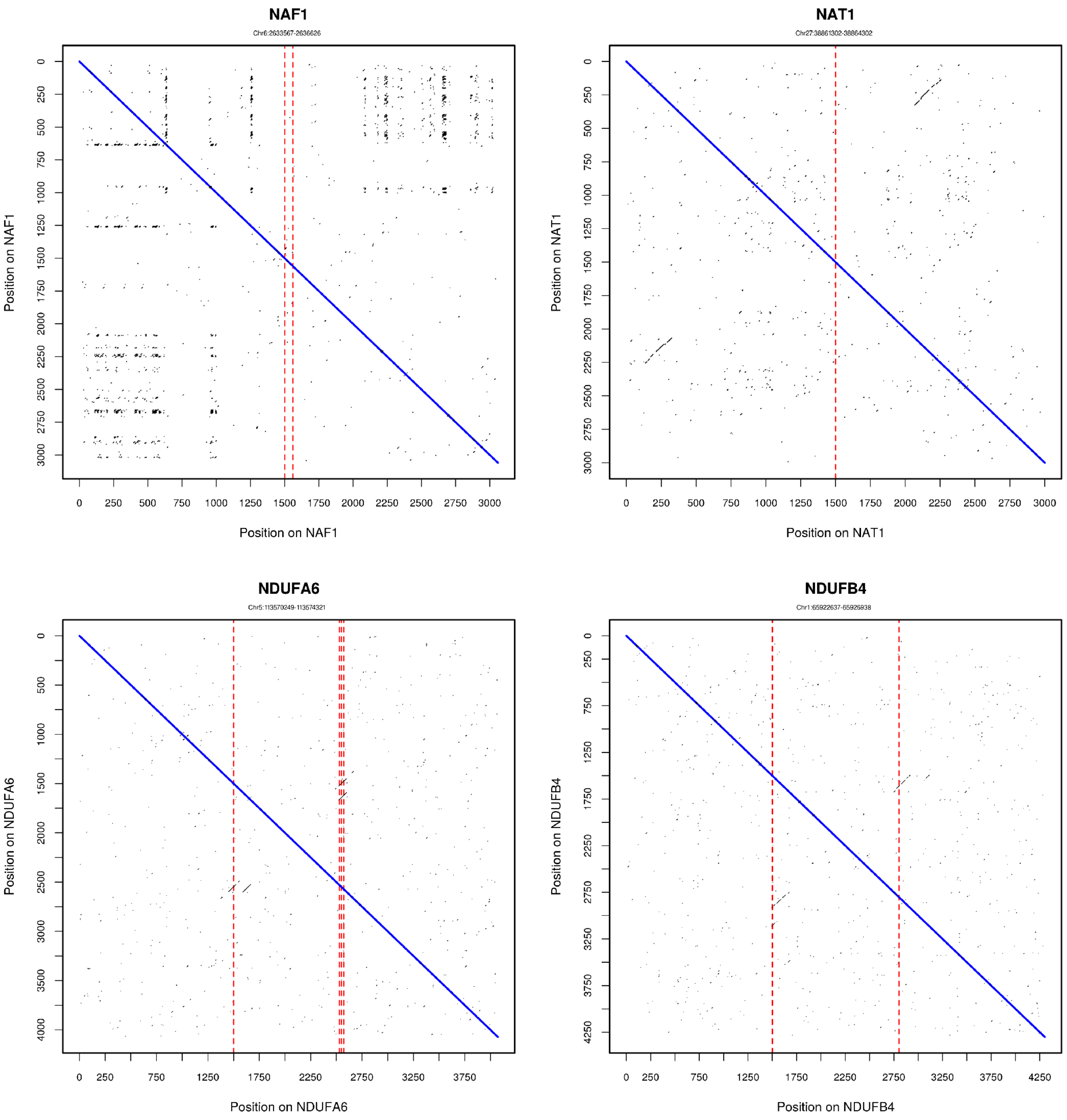
NAF1
Chr6:2633567-2636626
Position on NAF1
Position on NAF1
0
250
500
750
1000
1250
1500
1750
2000
2250
2500
2750
3000
NAT1
Chr27:38861302-38864302
Position on NAT1
Position on NAT1
0
250
500
750
1000
1250
1500
1750
2000
2250
2500
2750
3000
NDUFA6
Chr5:113570249-113574321
Position on NDUFA6
Position on NDUFA6
0
250
500
750
1000
1250
1500
1750
2000
2250
2500
2750
3000
3250
3500
3750
4000
NDUFB4
Chr1:65922637-65926938
Position on NDUFB4
Position on NDUFB4
0
250
750
1250
1750
2250
2750
3250
3750
4250

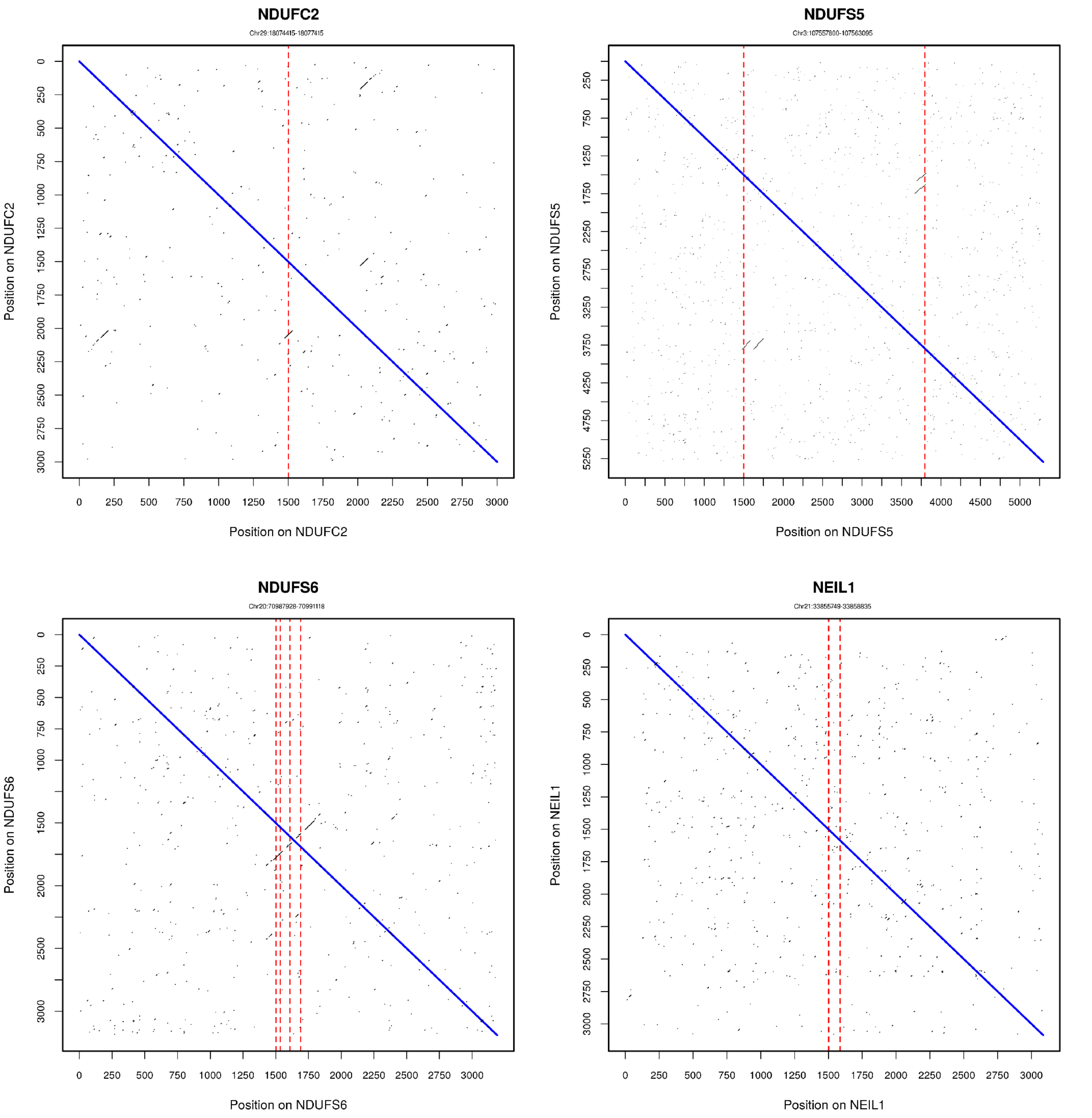
NDUFC2
Chr29:18074415-18077415
Position on NDUFC2
Position on NDUFC2
NDUFS5
Chr3:107557800-107563095
Position on NDUFS5
Position on NDUFS5
NDUFS6
Chr20:70987928-70991118
Position on NDUFS6
Position on NDUFS6
NEIL1
Chr21:33855749-33858835
Position on NEIL1
Position on NEIL1

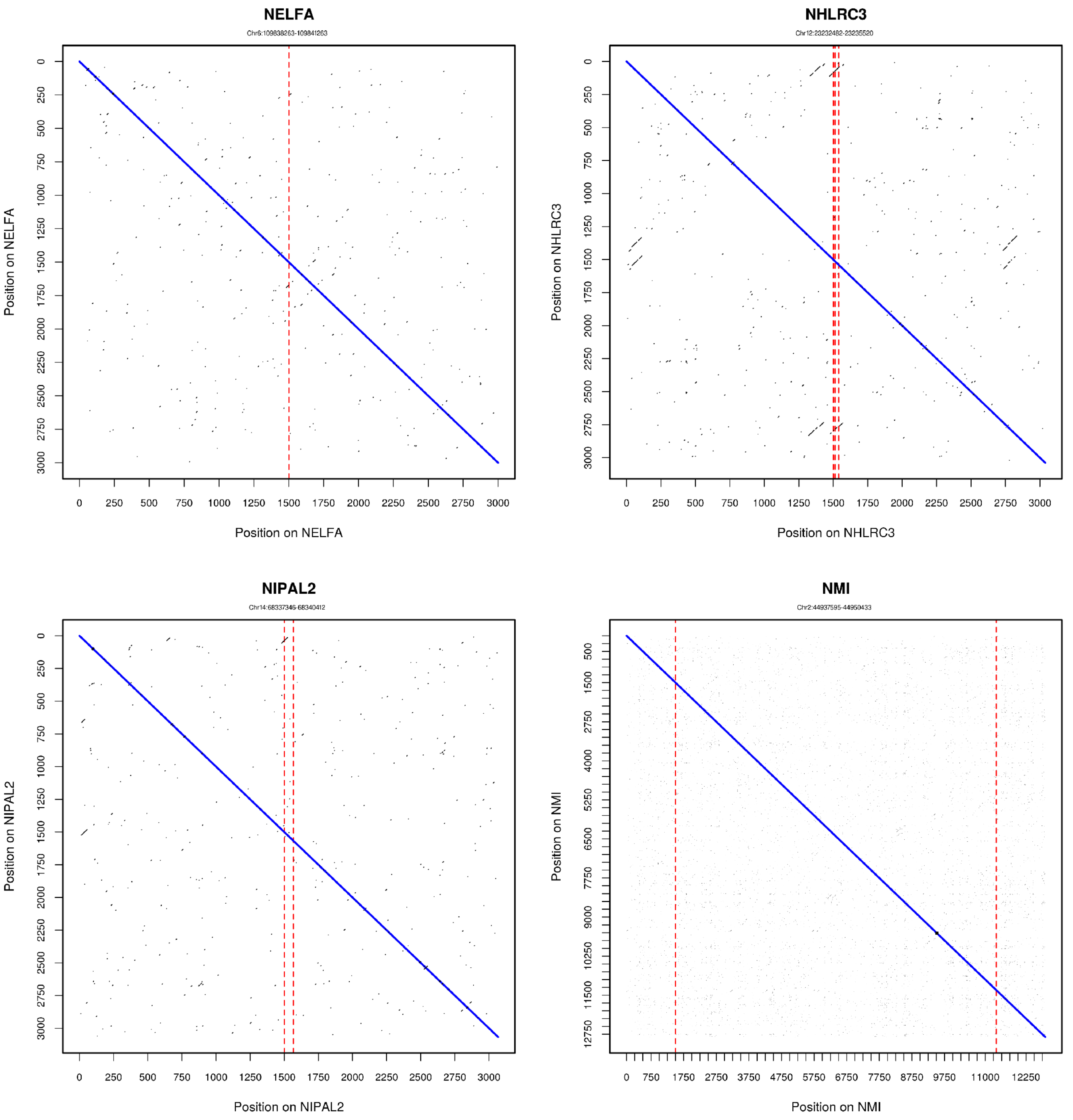
NELFA
Chr6:109838263-109841263
Position on NELFA
Position on NELFA
NHLRC3
Chr12:23232482-23235520
Position on NHLRC3
Position on NHLRC3
NIPAL2
Chr14:68337346-68340412
Position on NIPAL2
Position on NIPAL2
NMI
Chr2:44937595-44950433
Position on NMI
Position on NMI

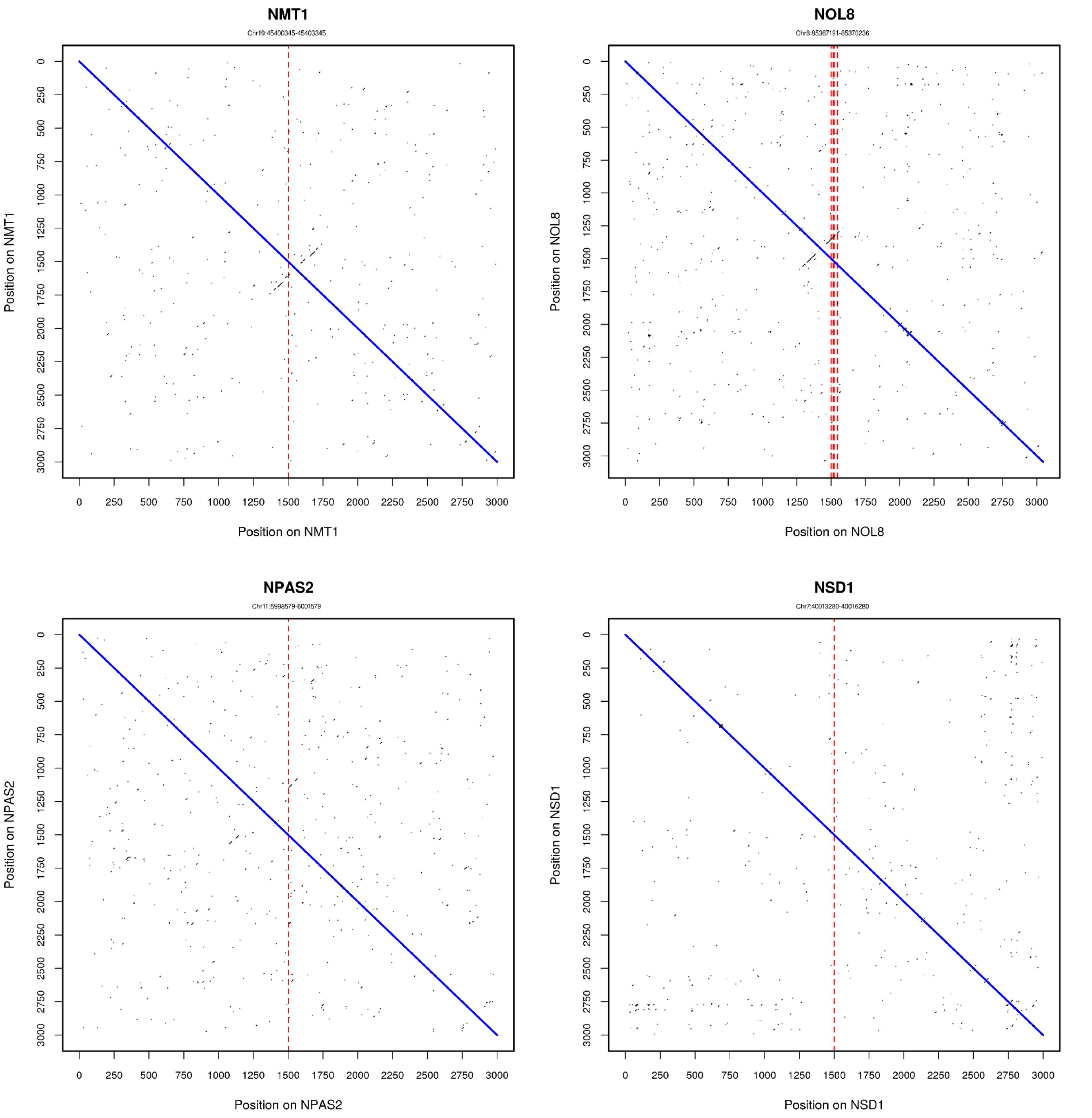

NMT1
Chr19:45400345-45403345
Position on NMT1
Position on NMT1
NOL8
Chr8:85367191-85370236
Position on NOL8
Position on NOL8
NPAS2
Chr11:5998579-6001579
Position on NPAS2
Position on NPAS2
NSD1
Chr7:40013280-40016280
Position on NSD1
Position on NSD1

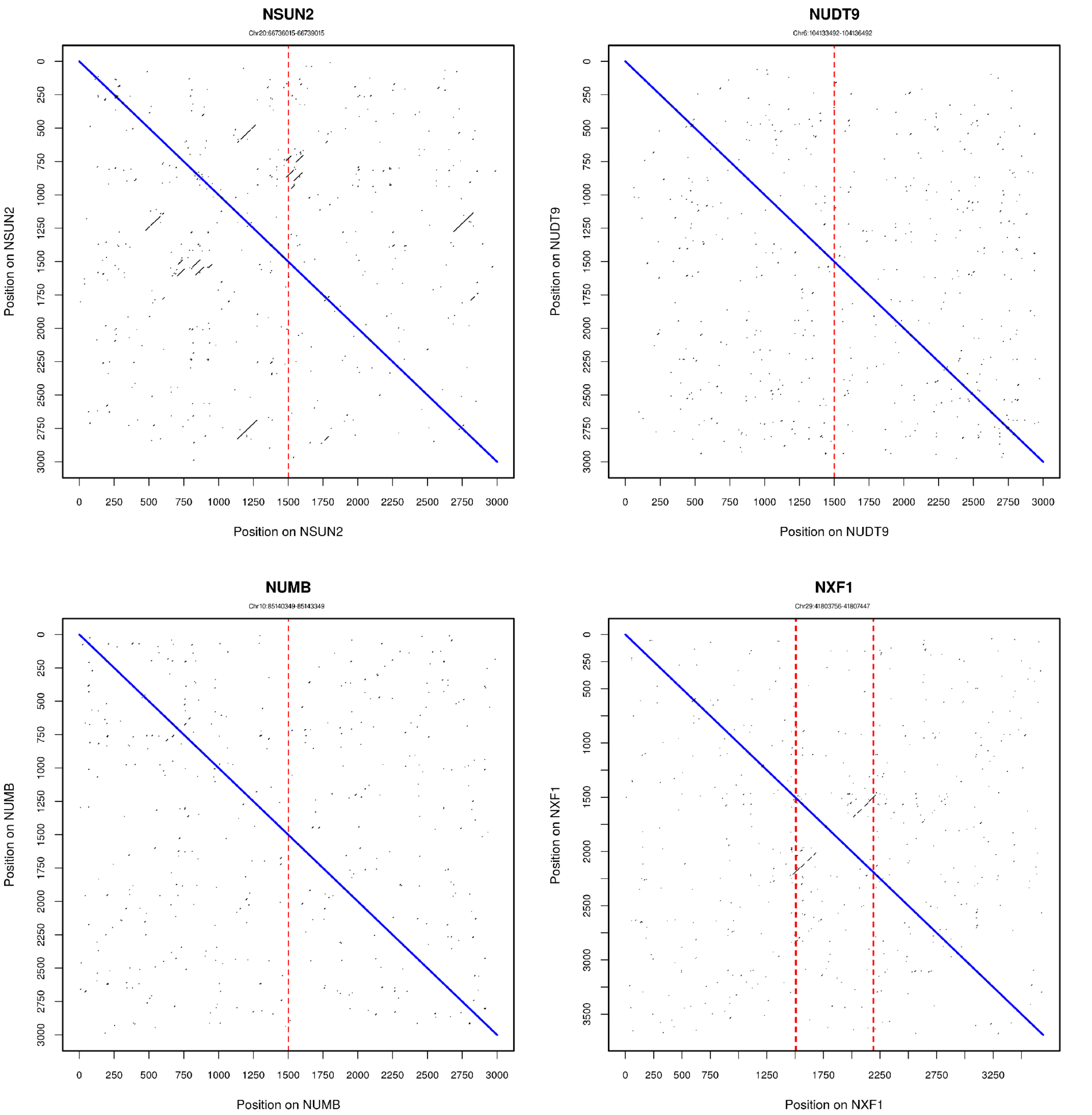

NSUN2
Chr20:66736015-66739015
Position on NSUN2
Position on NSUN2
NUDT9
Chr6:104133492-104136492
Position on NUDT9
Position on NUDT9
NUMB
Chr10:85140349-85143349
Position on NUMB
Position on NUMB
NXF1
Chr29:41803756-41807447
Position on NXF1
Position on NXF1

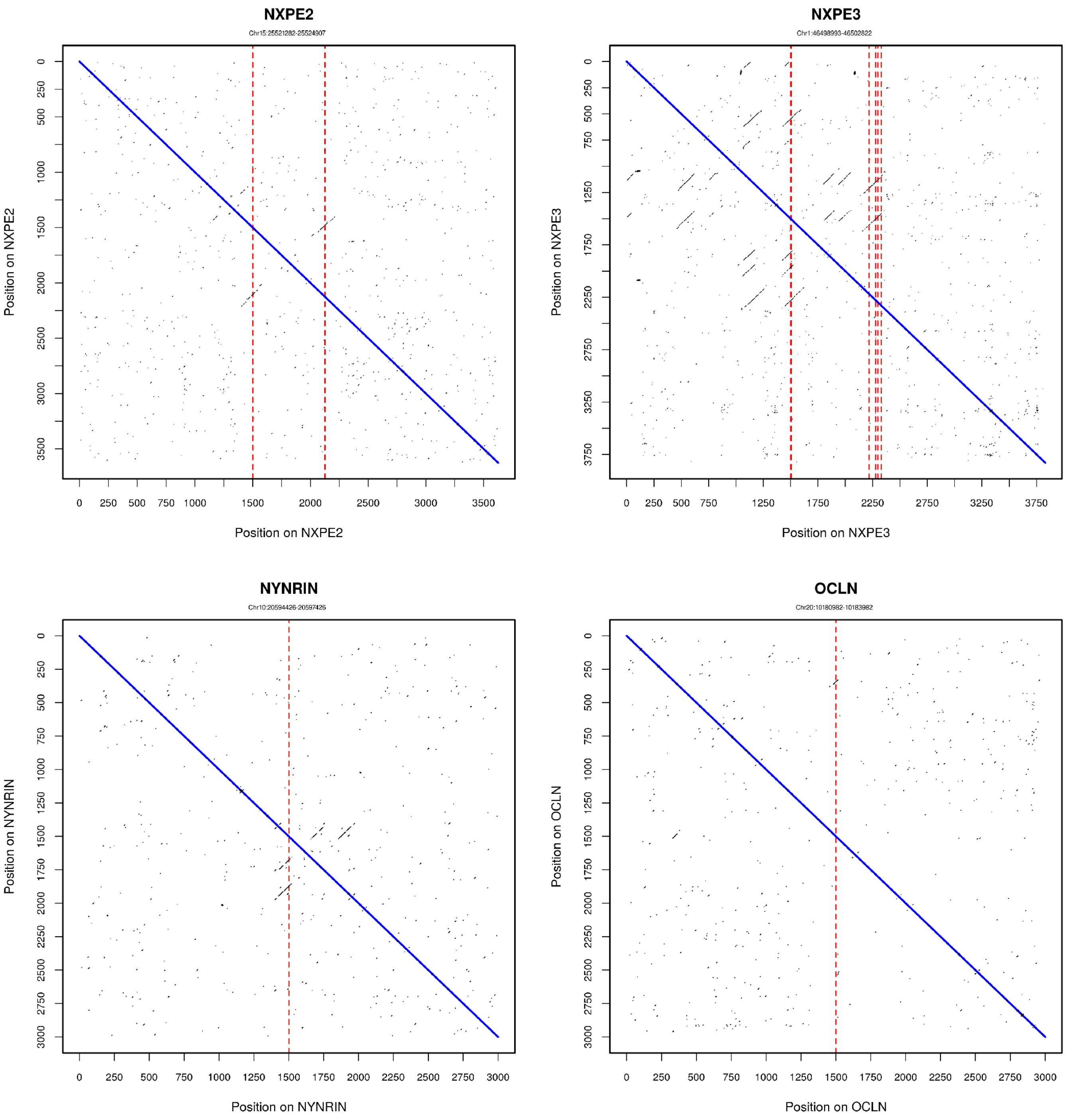
NXPE2
Chr15:25521282-25524907
Position on NXPE2
Position on NXPE2
NXPE3
Chr1:46498993-46502822
Position on NXPE3
Position on NXPE3
NYNRIN
Chr10:20594426-20597426
Position on NYNRIN
Position on NYNRIN
OCLN
Chr20:10180982-10183982
Position on OCLN
Position on OCLN

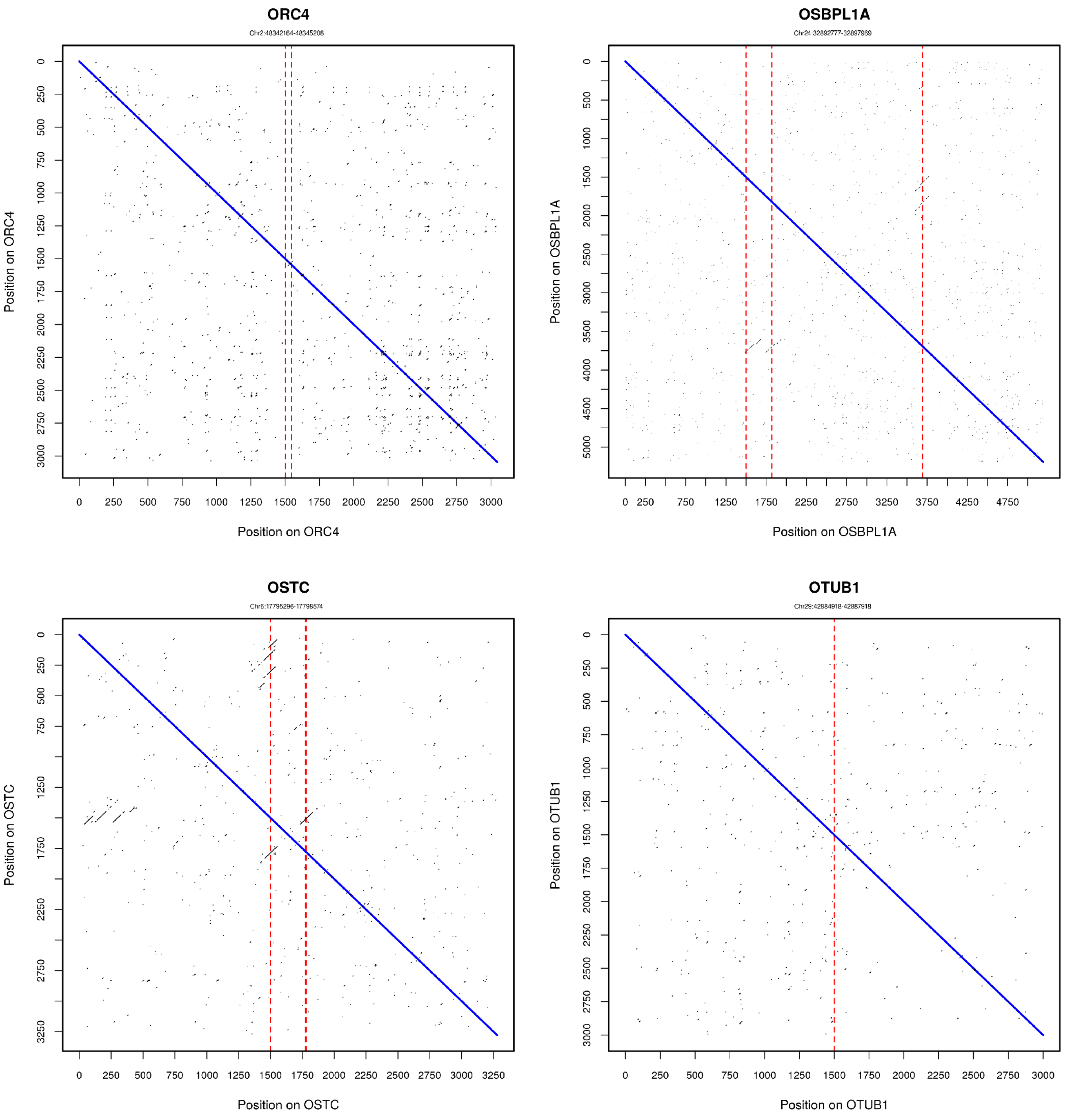

ORC4
Chr2:48342164-48345208
Position on ORC4
Position on ORC4
OSBPL1A
Chr24:32892777-32897969
Position on OSBPL1A
Position on OSBPL1A
OSTC
Chr6:17795296-17798574
Position on OSTC
Position on OSTC
OTUB1
Chr29:42884918-42887918
Position on OTUB1
Position on OTUB1

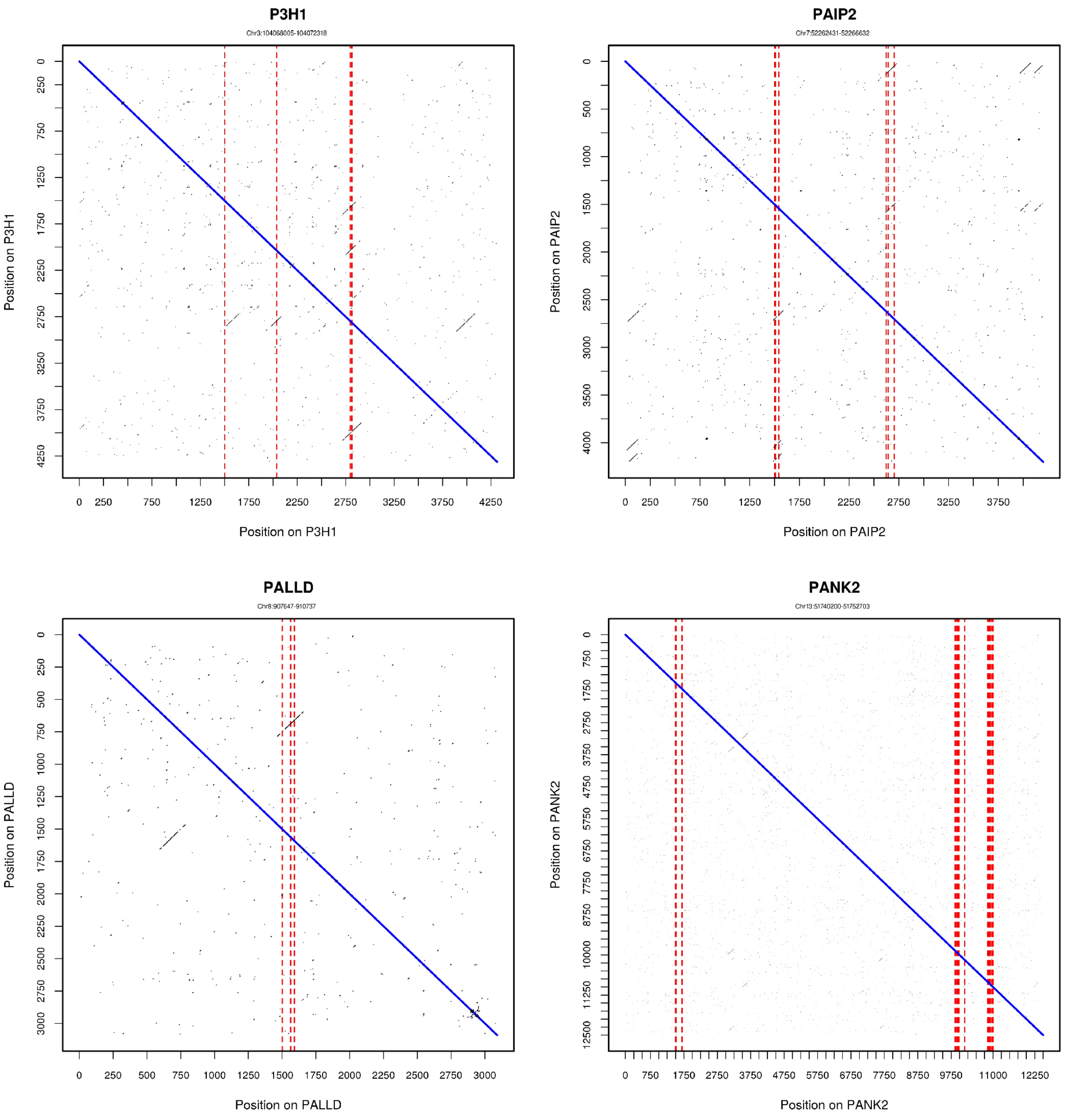

P3H1
Chr3:104068005-104072318
Position on P3H1
Position on P3H1
PAIP2
Chr7:52262431-52266632
Position on PAIP2
Position on PAIP2
PALLD
Chr8:907647-910737
Position on PALLD
Position on PALLD
PANK2
Chr13:51740200-51752703
Position on PANK2
Position on PANK2

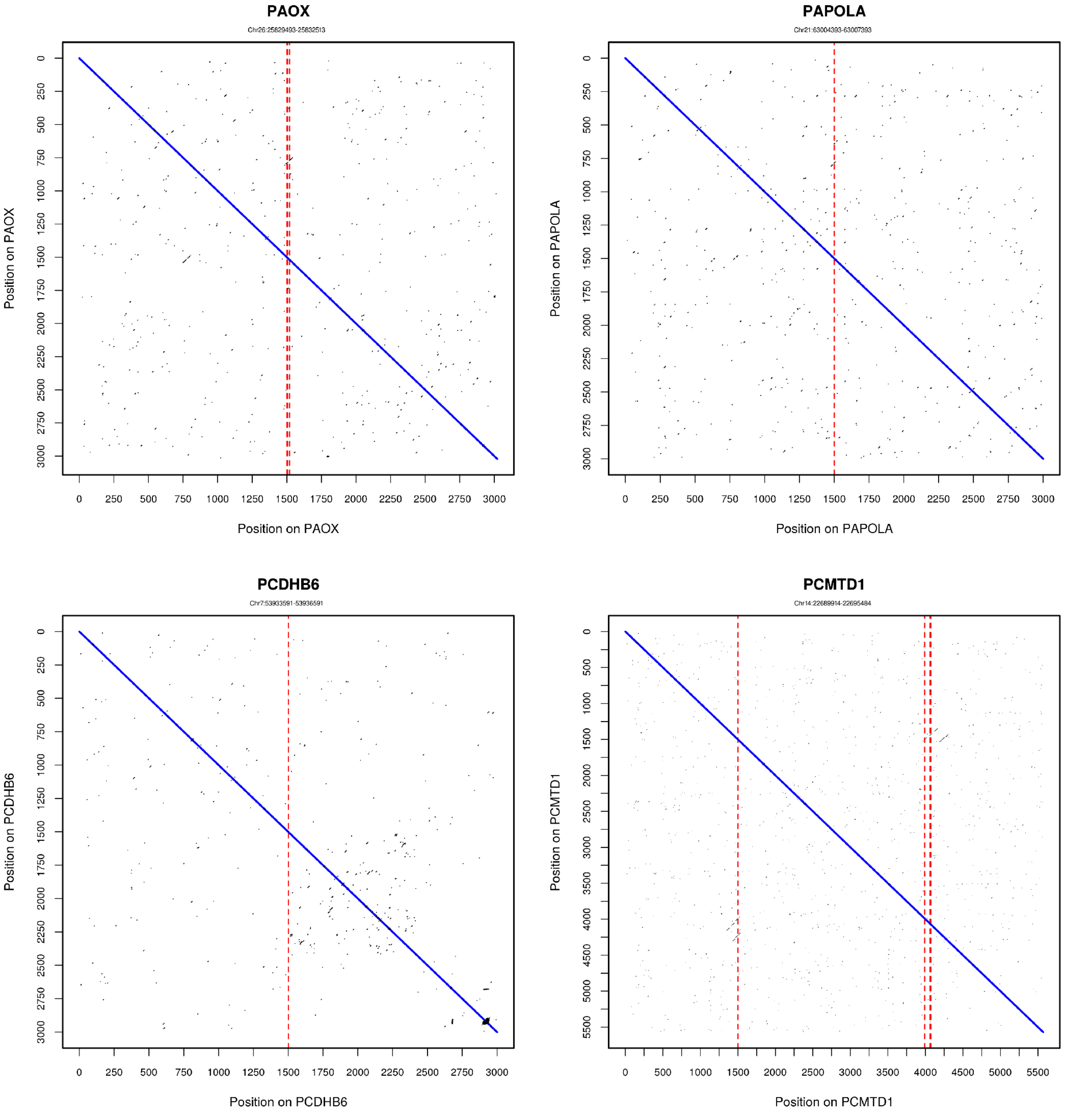

PAOX
Chr26:25829493-25832513
Position on PAOX
Position on PAOX
0
250
500
750
1000
1250
1500
1750
2000
2250
2500
2750
3000
PAPOLA
Chr21:63004393-63007393
Position on PAPOLA
Position on PAPOLA
PCDHB6
Chr7:53933591-53936591
Position on PCDHB6
Position on PCDHB6
PCMTD1
Chr14:22689914-22695484
Position on PCMTD1
Position on PCMTD1
3500
4000
4500
5000
5500

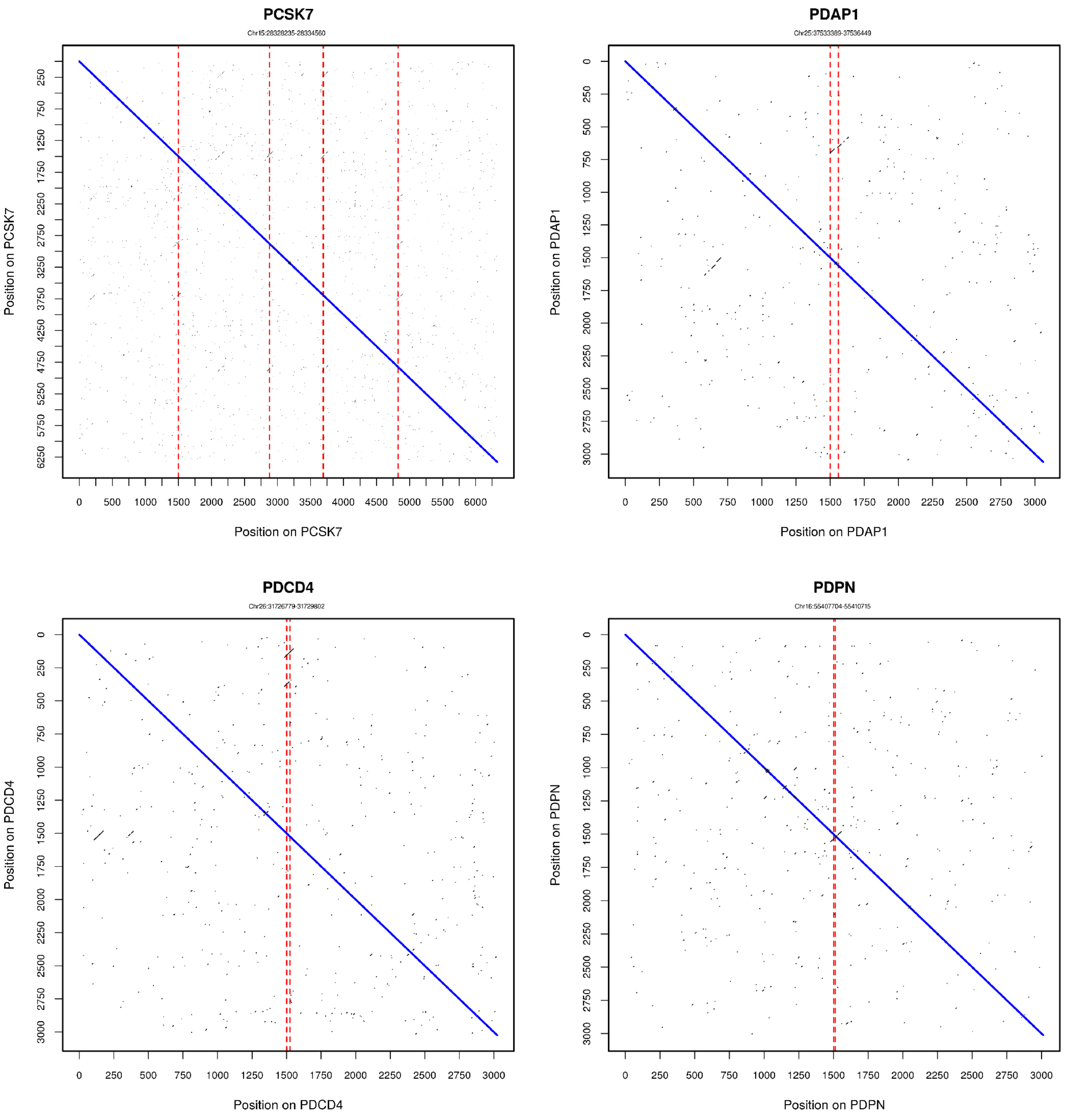
PCSK7
Chr15:28328235-28334560
Position on PCSK7
Position on PCSK7
PDAP1
Chr25:37533389-37536449
Position on PDAP1
Position on PDAP1
PDCD4
Chr26:31726779-31729802
Position on PDCD4
Position on PDCD4
PDPN
Chr16:55407704-55410715
Position on PDPN
Position on PDPN

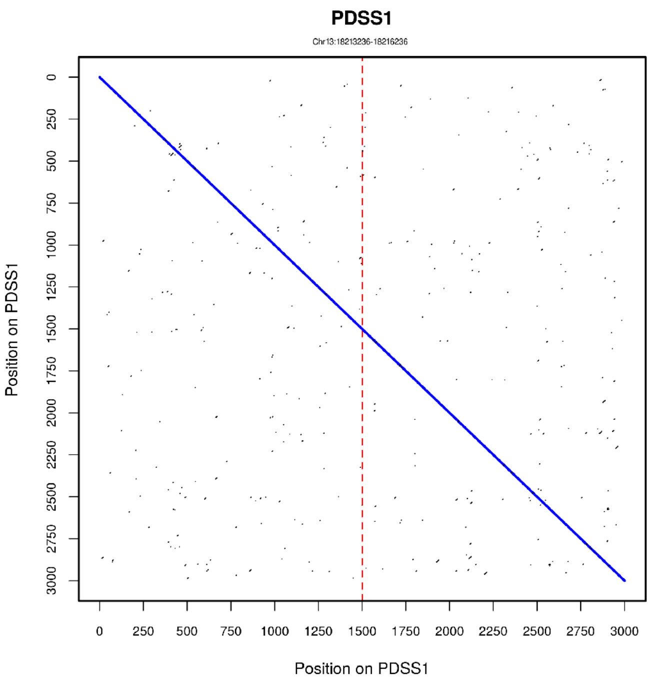
PDSS1
Chr13:18213236-18216236
Position on PDSS1
Position on PDSS1
0
250
500
750
1000
1250
1500
1750
2000
2250
2500
2750
3000

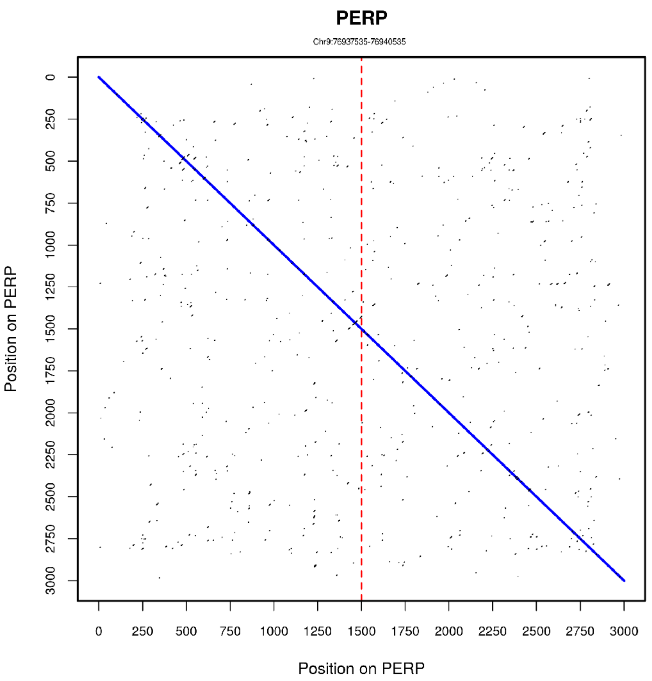
PERP
Chr9:76937535-76940535
Position on PERP
Position on PERP
0
250
500
750
1000
1250
1500
1750
2000
2250
2500
2750
3000

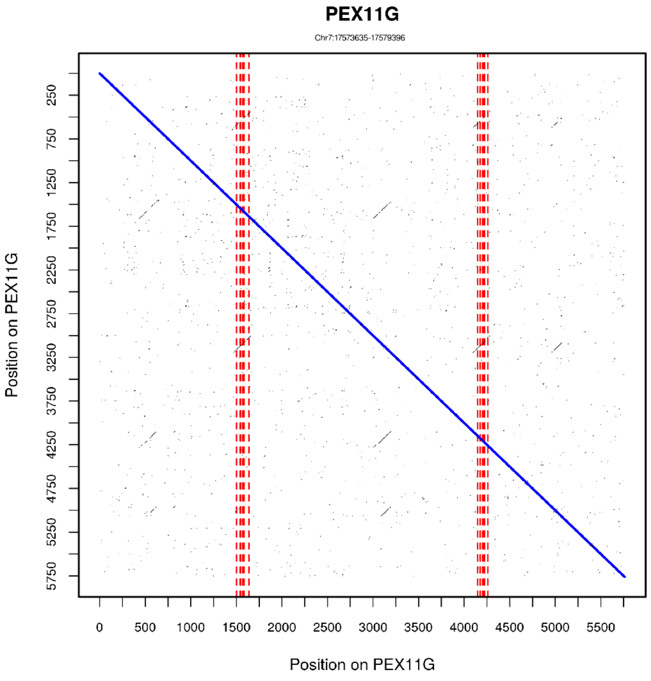
PEX11G
Chr7:17573635-17579396
Position on PEX11G
Position on PEX11G
0
500
1000
1500
2000
2500
3000
3500
4000
4500
5000
5500
250
750
1250
1750
2250
2750
3250
3750
4250
4750
5250
5750

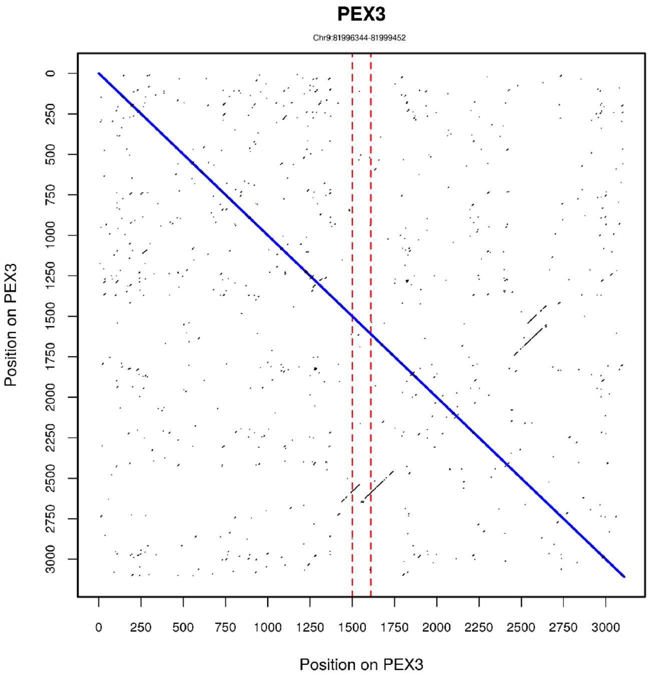
PEX3
Chr9:81996344-81999452
Position on PEX3
Position on PEX3
0
250
500
750
1000
1250
1500
1750
2000
2250
2500
2750
3000

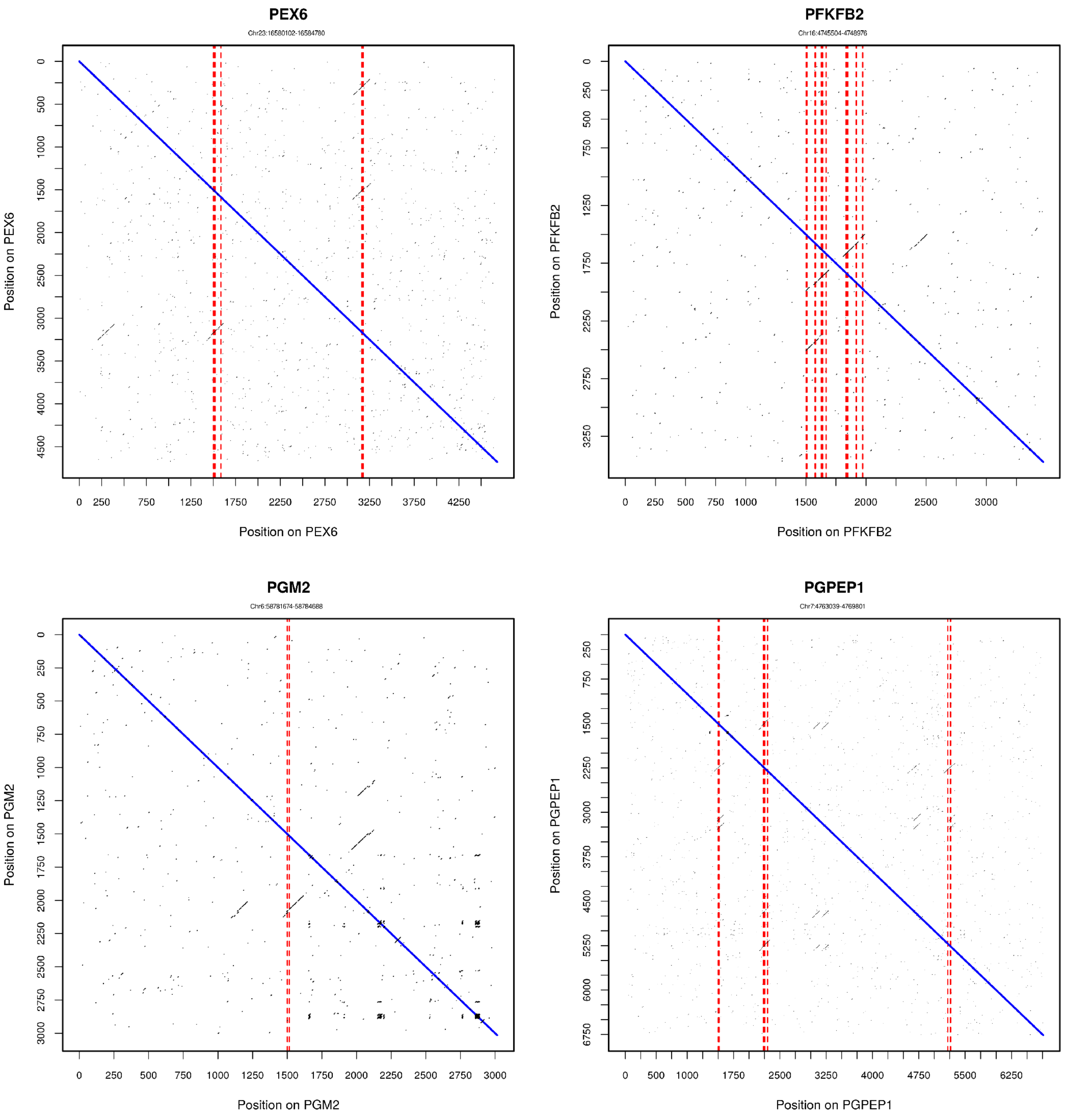
PEX6
Chr23:16580102-16584780
Position on PEX6
Position on PEX6
PFKFB2
Chr16:4745504-4748976
Position on PFKFB2
Position on PFKFB2
PGM2
Chr6:58781674-58784688
Position on PGM2
Position on PGM2
PGPEP1
Chr7:4763039-4769801
Position on PGPEP1
Position on PGPEP1

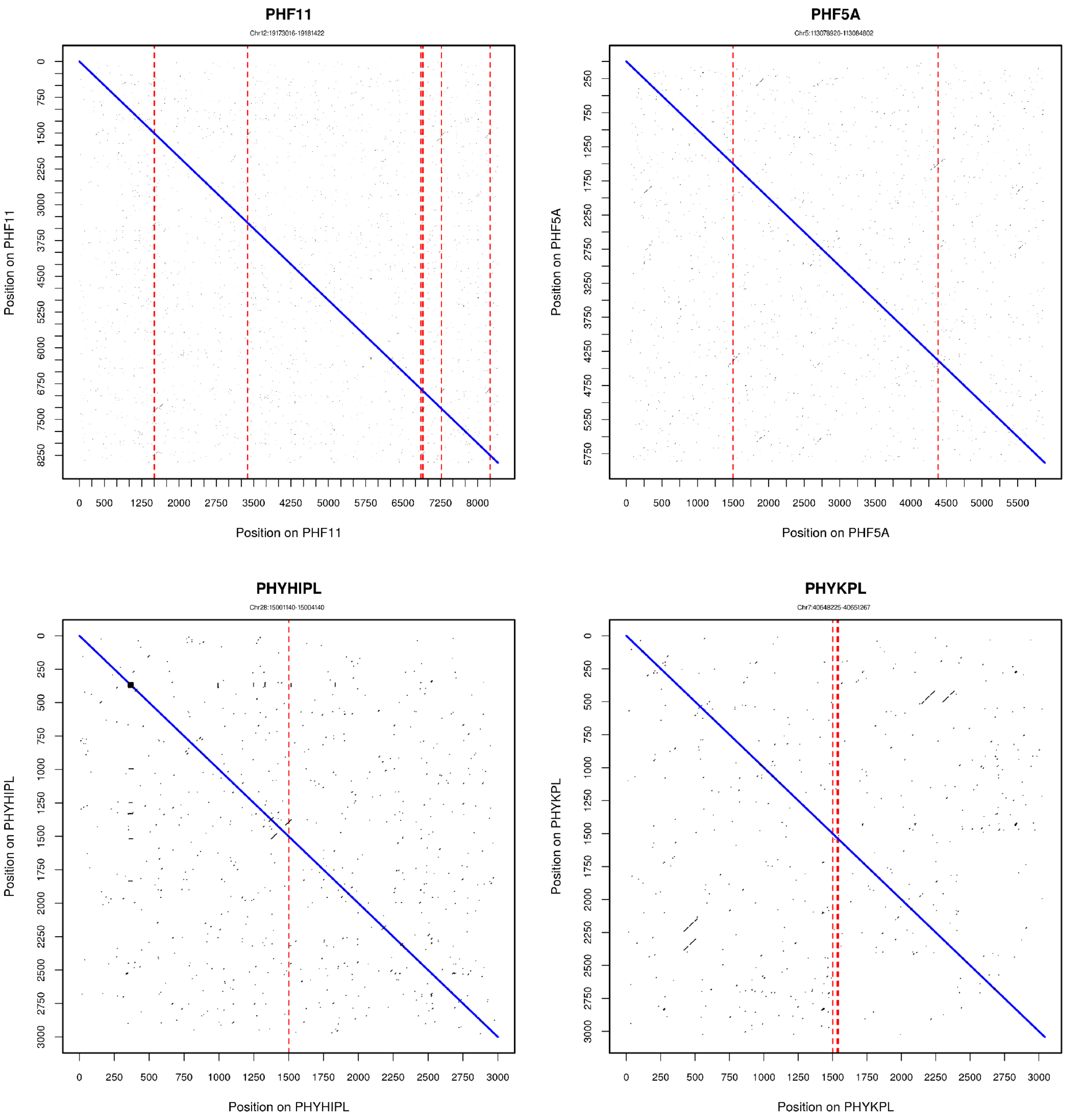

PHF11
Chr12:19173016-19181422
Position on PHF11
Position on PHF11
PHF5A
Chr5:113078920-113084802
Position on PHF5A
Position on PHF5A
PHYHIPL
Chr28:15001140-15004140
Position on PHYHIPL
Position on PHYHIPL
PHYKPL
Chr7:40648225-40651267
Position on PHYKPL
Position on PHYKPL

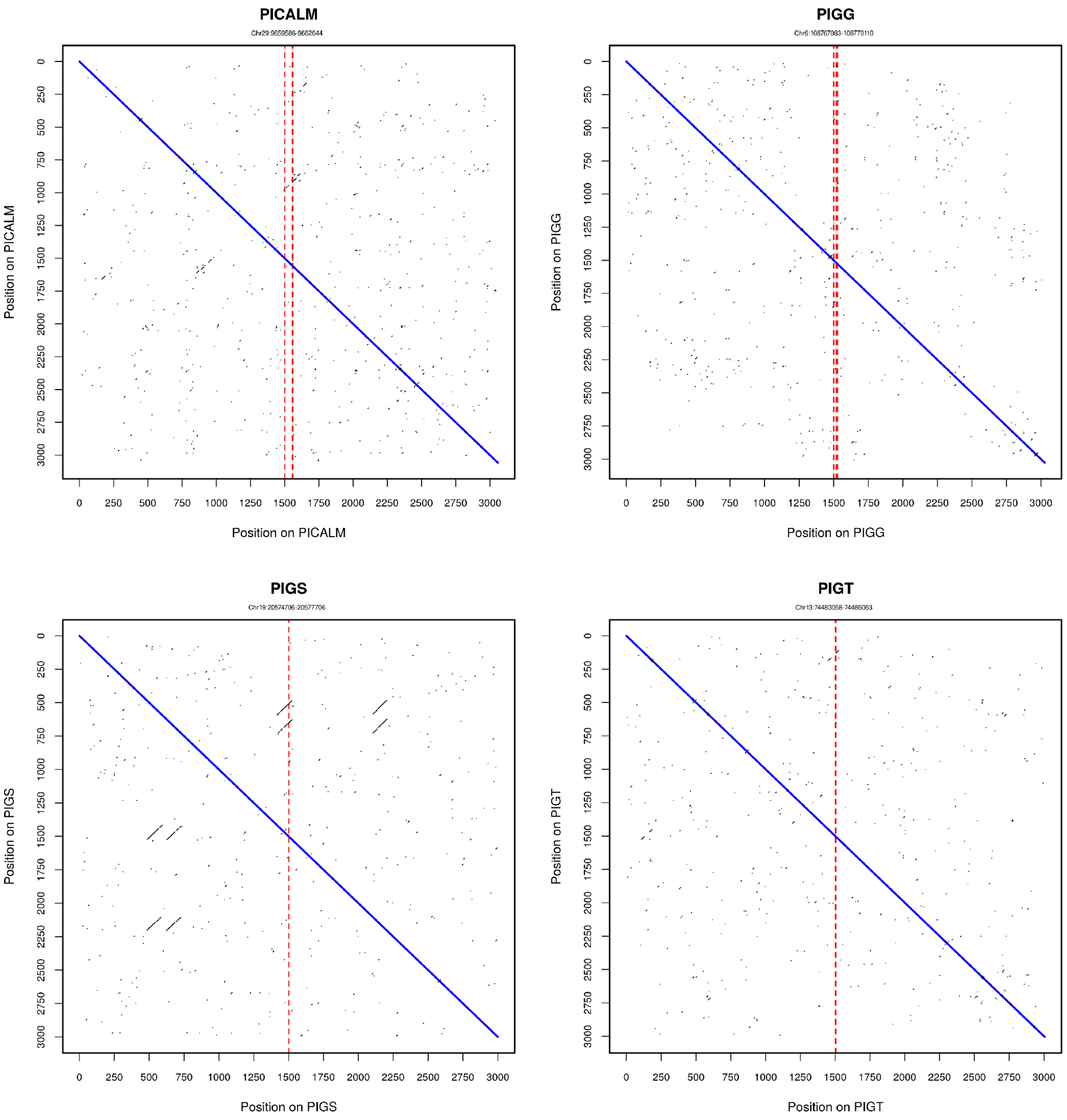

PICALM
Chr29:9659586-9662644
Position on PICALM
Position on PICALM
PIGG
Chr6:108767083-108770110
Position on PIGG
Position on PIGG
PIGS
Chr19:20574706-20577706
Position on PIGS
Position on PIGS
PIGT
Chr13:74483058-74486063
Position on PIGT
Position on PIGT

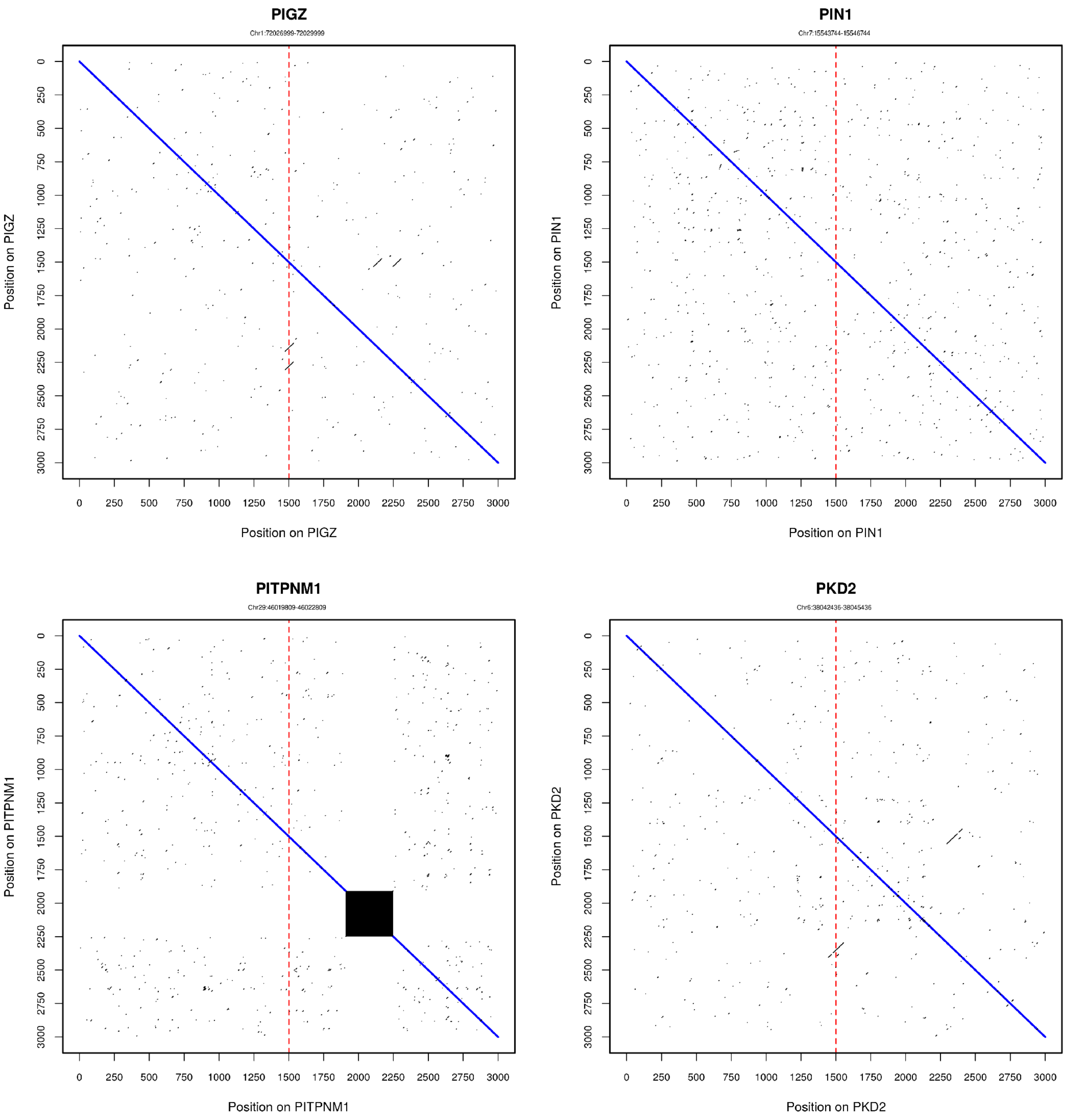
PIGZ
Chr1:72026999-72029999
Position on PIGZ
Position on PIGZ
PIN1
Chr7:15543744-15546744
Position on PIN1
Position on PIN1
PITPNM1
Chr29:46019809-46022809
Position on PITPNM1
Position on PITPNM1
PKD2
Chr6:38042436-38045436
Position on PKD2
Position on PKD2

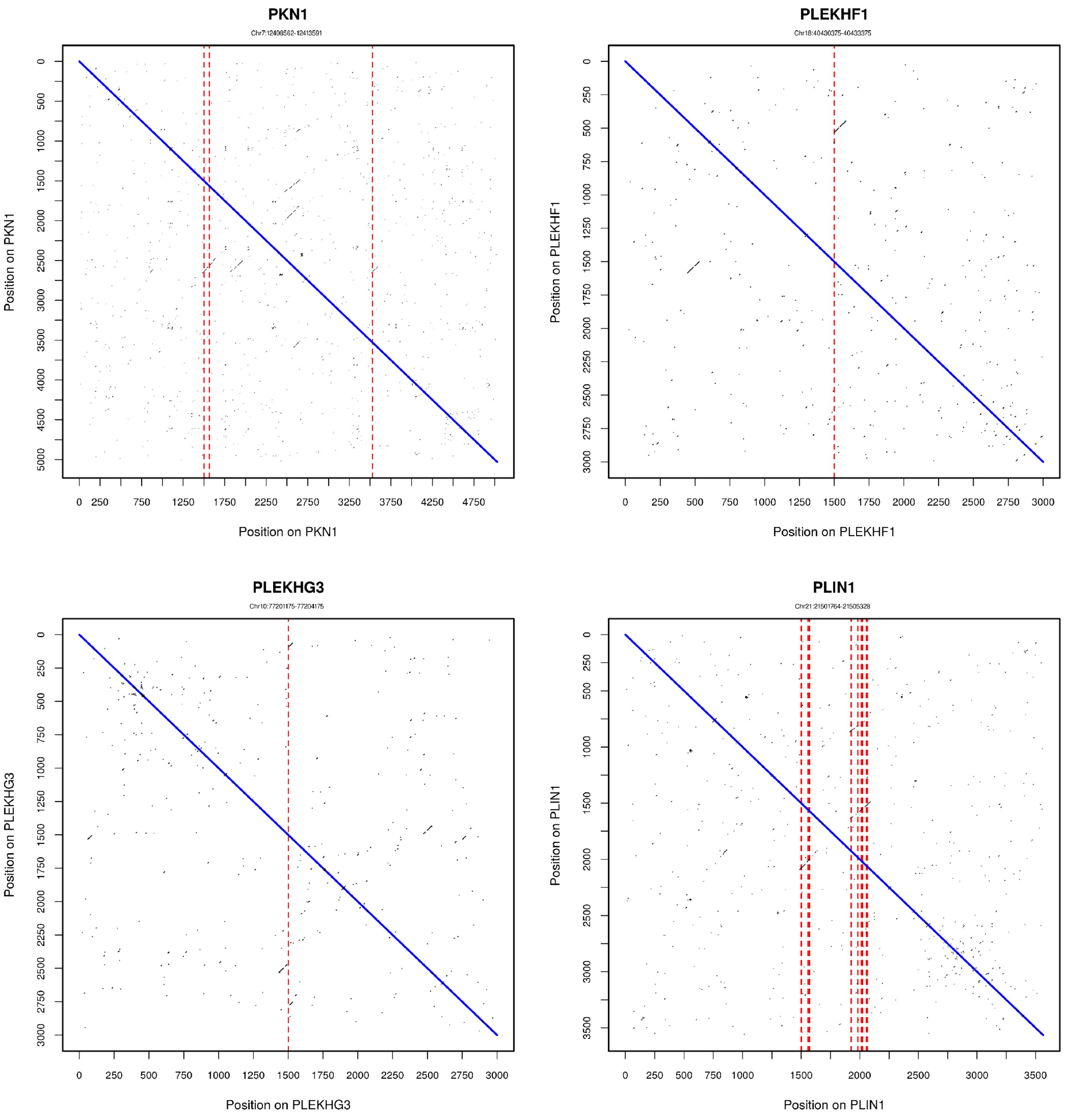
PKN1
Chr7:12408562-12413591
Position on PKN1
Position on PKN1
PLEKHF1
Chr18:40430375-40433375
Position on PLEKHF1
Position on PLEKHF1
PLEKHG3
Chr10:77201175-77204175
Position on PLEKHG3
Position on PLEKHG3
PLIN1
Chr21:21501764-21505328
Position on PLIN1
Position on PLIN1

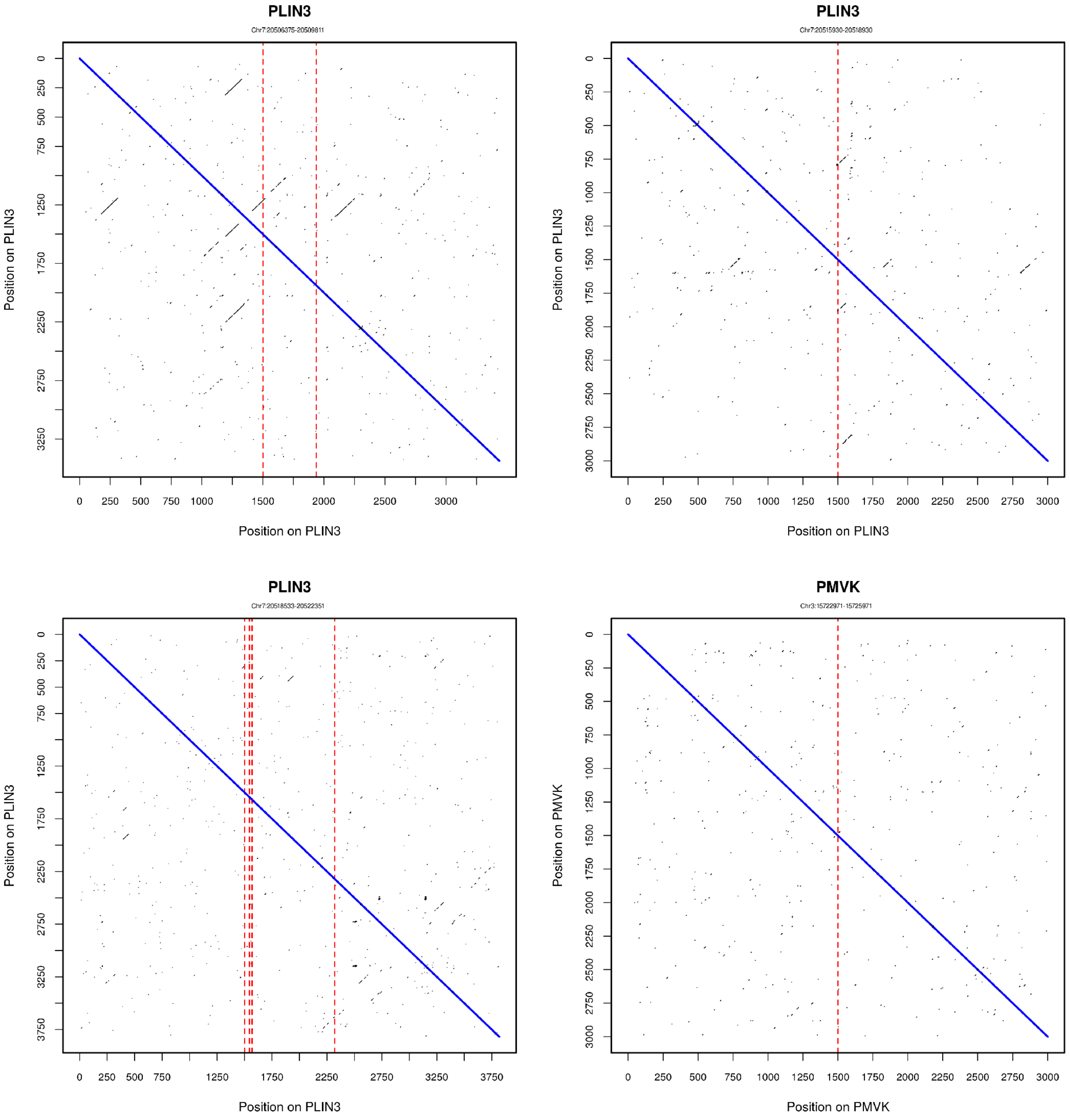
PLIN3
Chr7:20506375-20509811
Position on PLIN3
Position on PLIN3
PLIN3
Chr7:20515930-20518930
Position on PLIN3
Position on PLIN3
PLIN3
Chr7:20518533-20522351
Position on PLIN3
Position on PLIN3
PMVK
Chr3:15722971-15725971
Position on PMVK
Position on PMVK

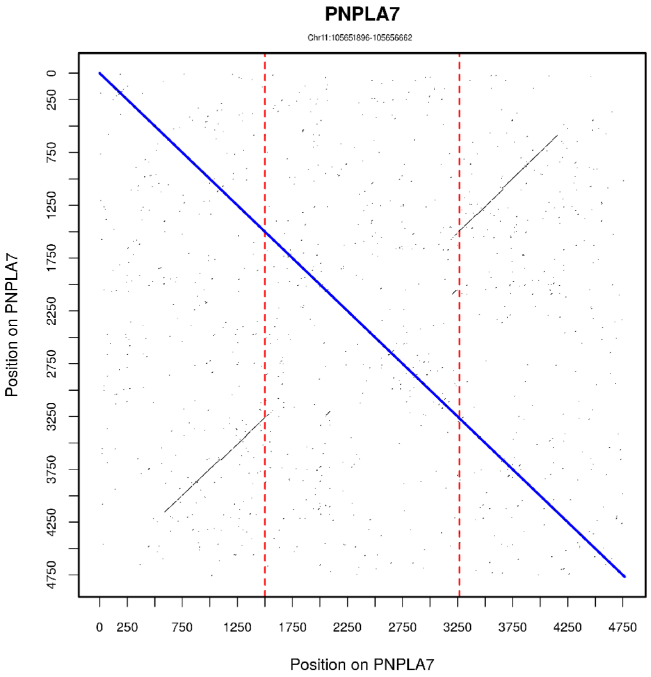
PNPLA7
Chr11:105651896-105656662
Position on PNPLA7
Position on PNPLA7

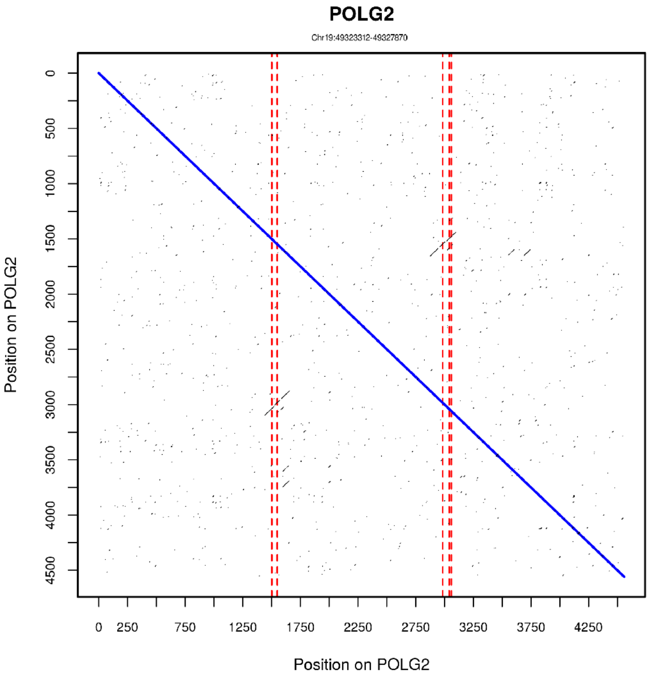
POLG2
Chr19:49323312-49327870
Position on POLG2
Position on POLG2

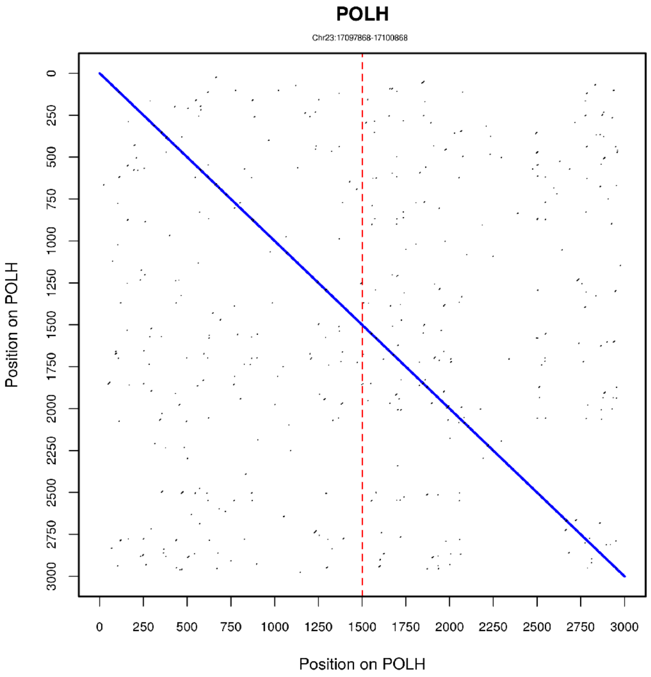
POLH
Chr23:17097868-17100868
Position on POLH
Position on POLH

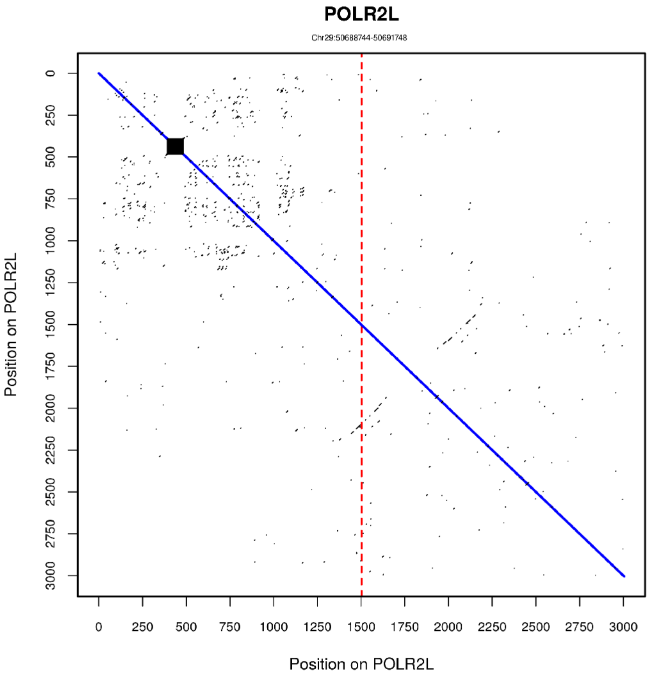
POLR2L
Chr29:50688744-50691748
Position on POLR2L
Position on POLR2L

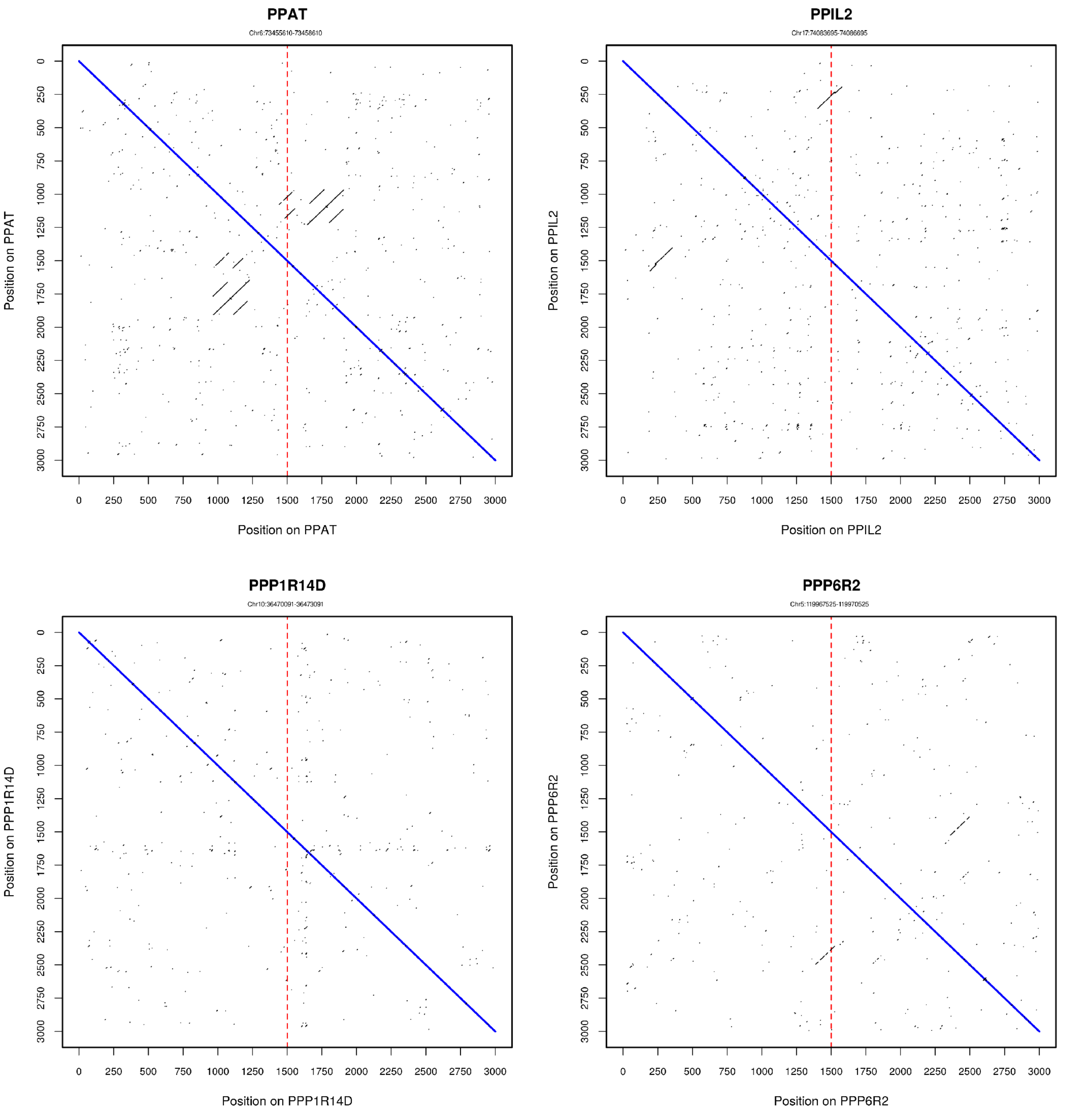
PPAT
Chr6:73455610-73458610
Position on PPAT
Position on PPAT
PPIL2
Chr17:74083695-74086695
Position on PPIL2
Position on PPIL2
PPP1R14D
Chr10:36470091-36473091
Position on PPP1R14D
Position on PPP1R14D
PPP6R2
Chr5:119967525-119970525
Position on PPP6R2
Position on PPP6R2
0
250
500
750
1000
1250
1500
1750
2000
2250
2500
2750
3000

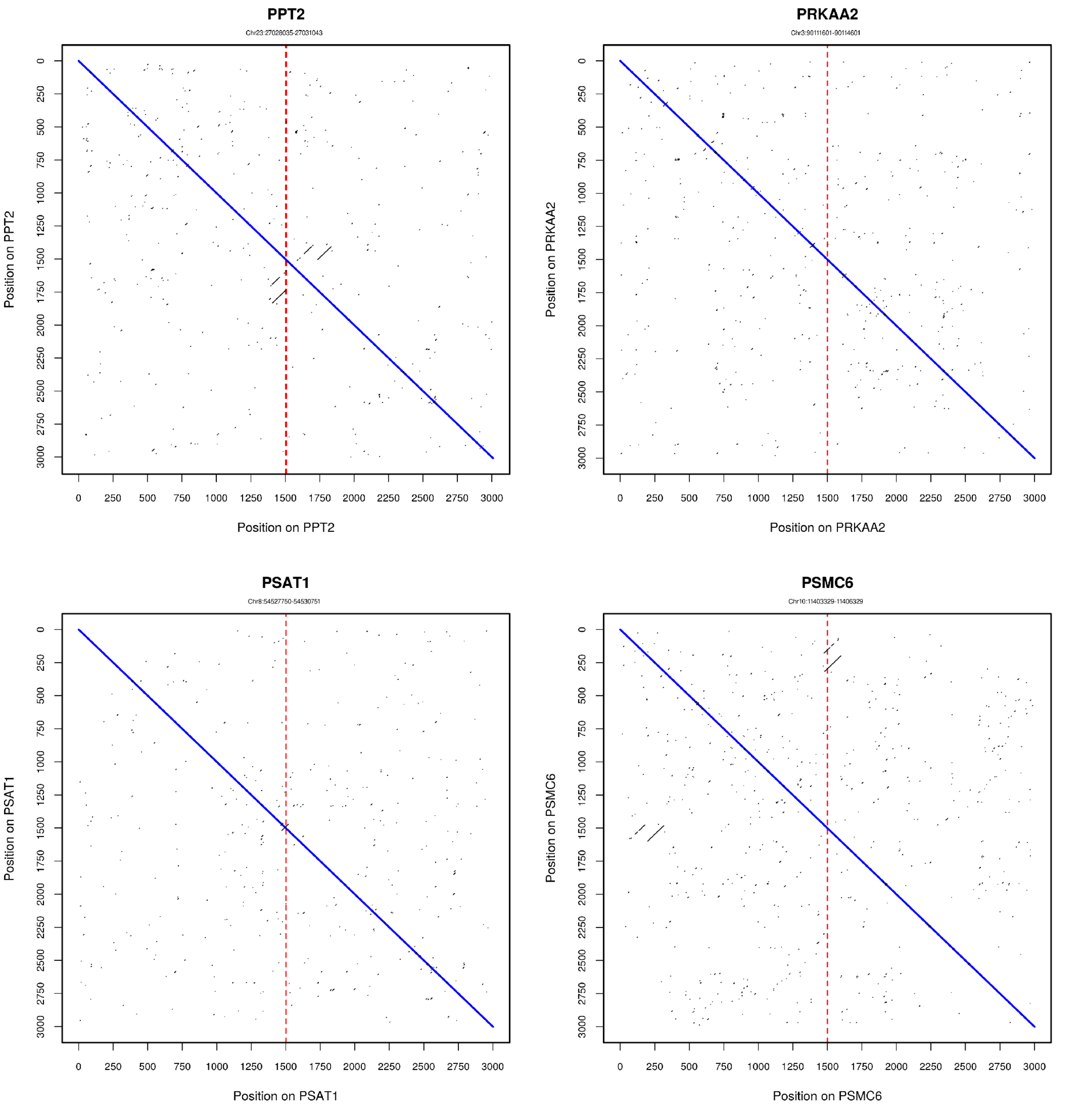

PPT2
Chr23:27028035-27031043
Position on PPT2
Position on PPT2
PRKAA2
Chr3:90111601-90114601
Position on PRKAA2
Position on PRKAA2
PSAT1
Chr8:54527750-54530751
Position on PSAT1
Position on PSAT1
PSMC6
Chr10:11403329-11406329
Position on PSMC6
Position on PSMC6

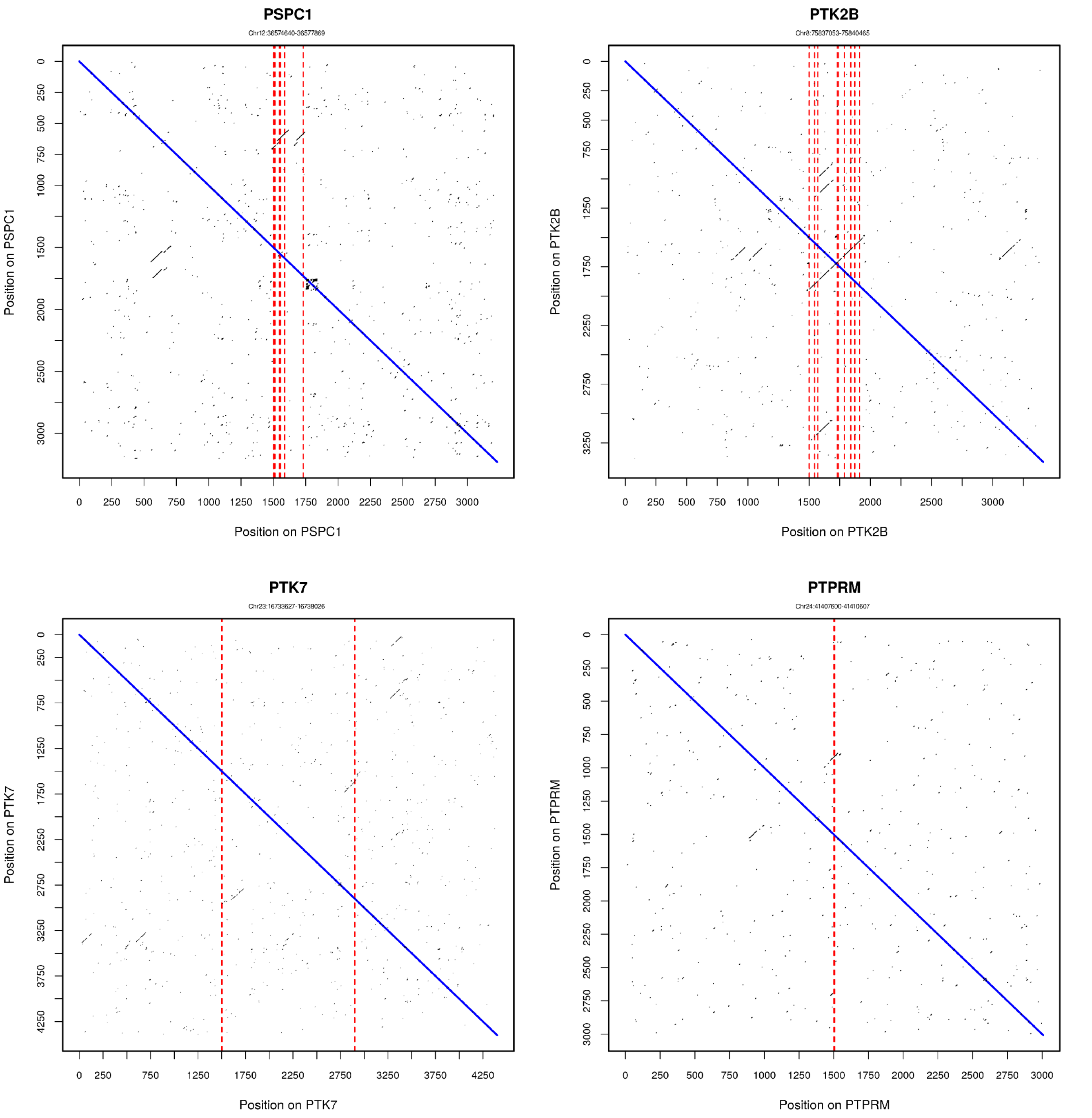
PSPC1
Chr12:36574640-36577869
Position on PSPC1
Position on PSPC1
PTK2B
Chr8:75837053-75840465
Position on PTK2B
Position on PTK2B
PTK7
Chr23:16733627-16738026
Position on PTK7
Position on PTK7
PTPRM
Chr24:41407600-41410607
Position on PTPRM
Position on PTPRM

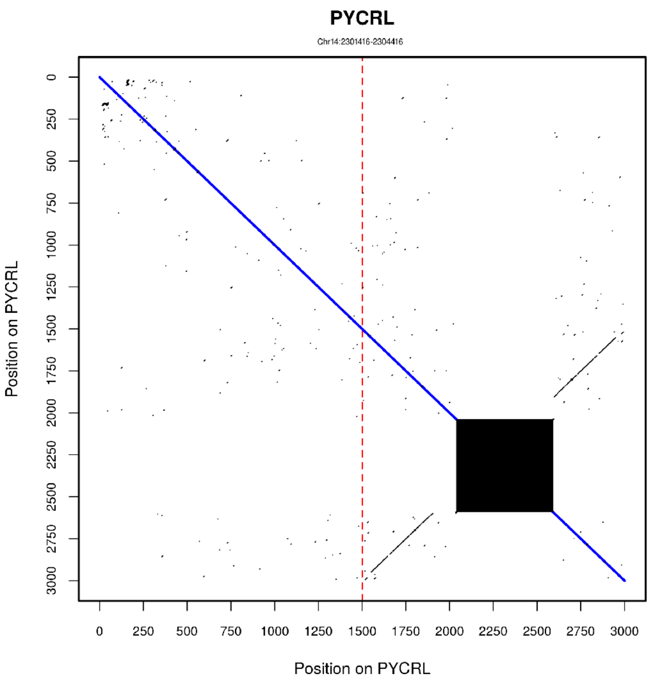
PYCRL
Chr14:2301416-2304416
Position on PYCRL
Position on PYCRL

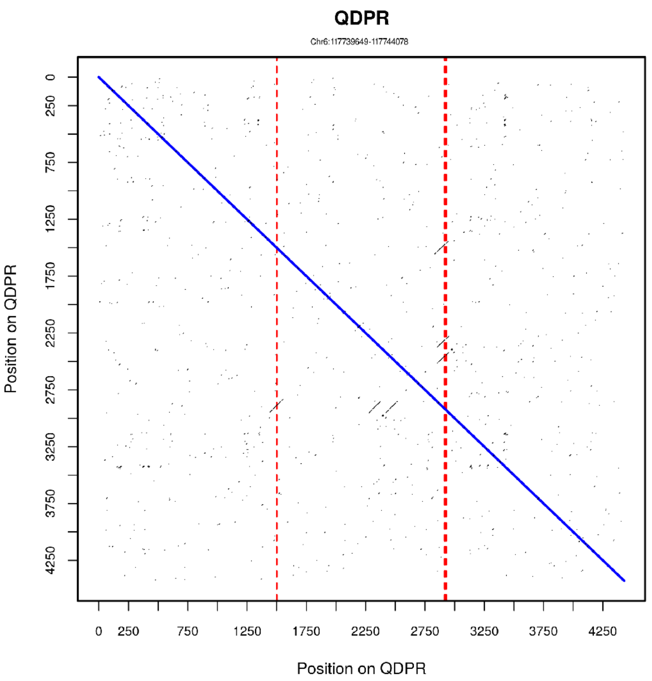
QDPR
Chr6:117739649-117744078
Position on QDPR
Position on QDPR

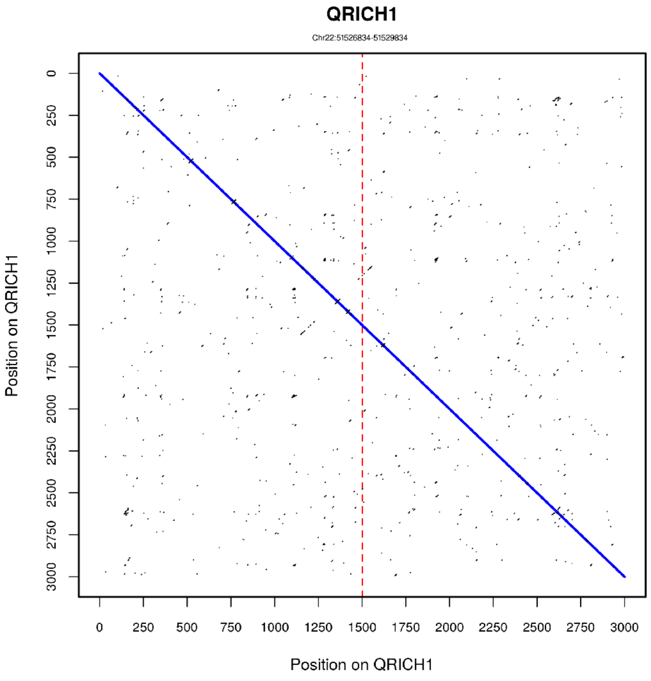
QRICH1
Chr22:51526834-51529834
Position on QRICH1
Position on QRICH1

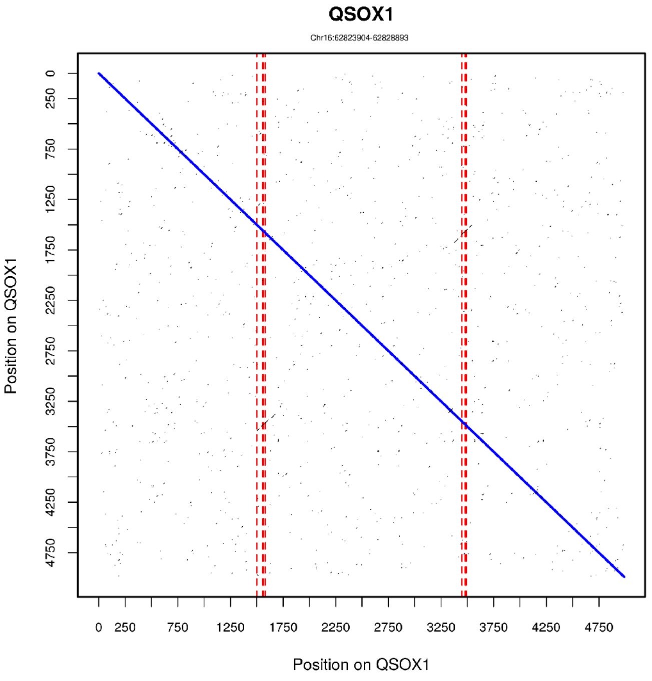
QSOX1
Chr16:62823904-62828893
Position on QSOX1
Position on QSOX1

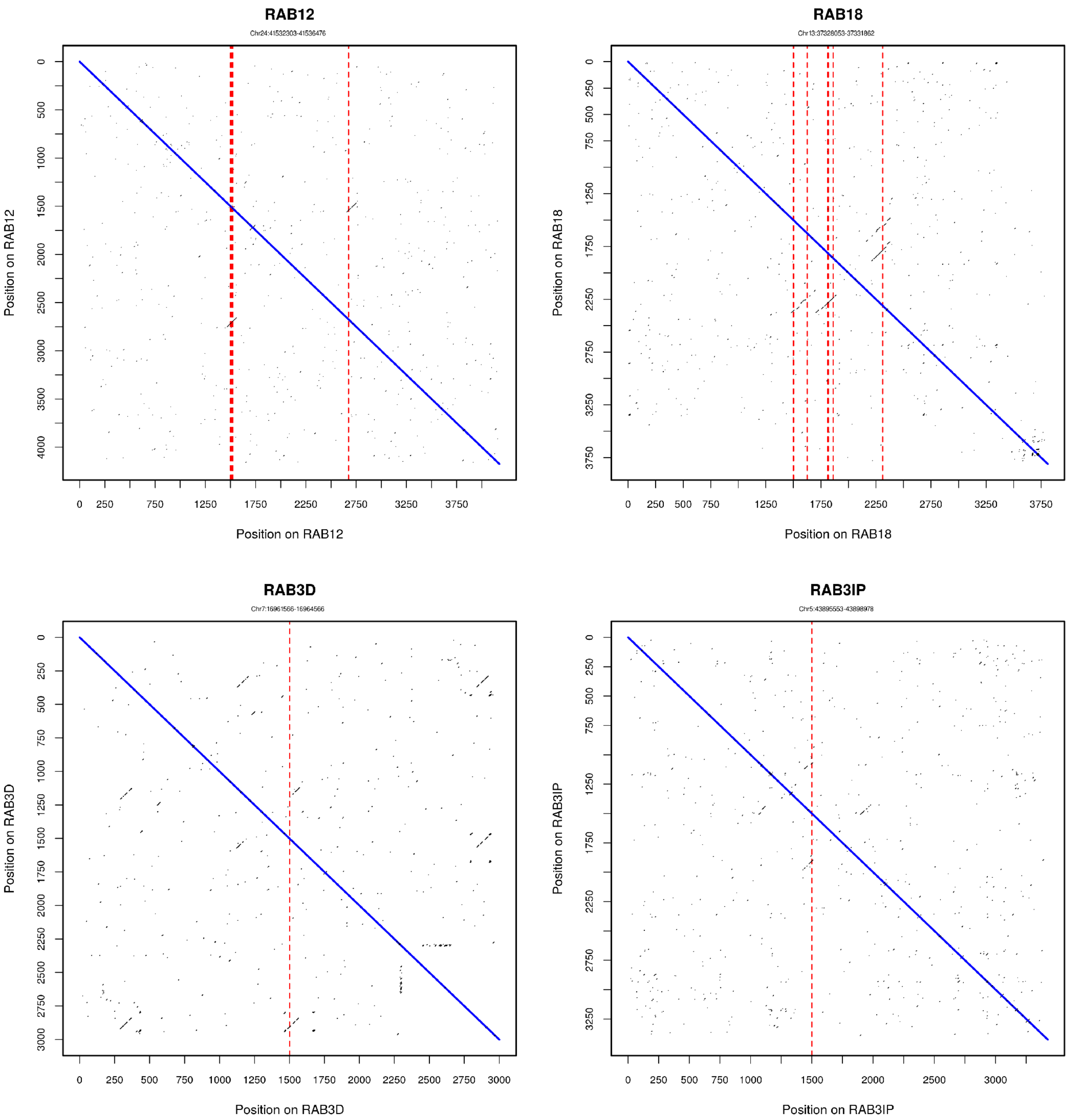
RAB12
Chr24:41532303-41536476
Position on RAB12
Position on RAB12
RAB18
Chr13:37328053-37331862
Position on RAB18
Position on RAB18
RAB3D
Chr7:16961566-16964566
Position on RAB3D
Position on RAB3D
RAB3IP
Chr5:43895553-43898978
Position on RAB3IP
Position on RAB3IP

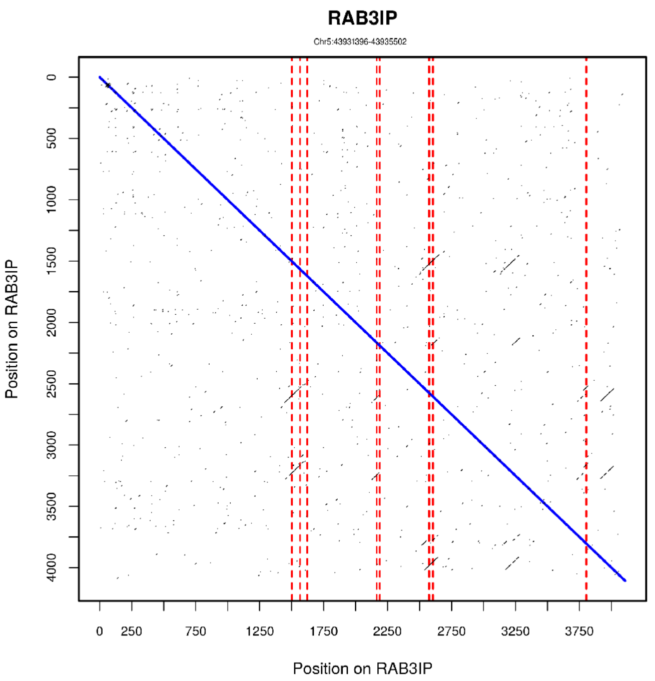
RAB3IP
Chr5:43931396-43935502
Position on RAB3IP
Position on RAB3IP

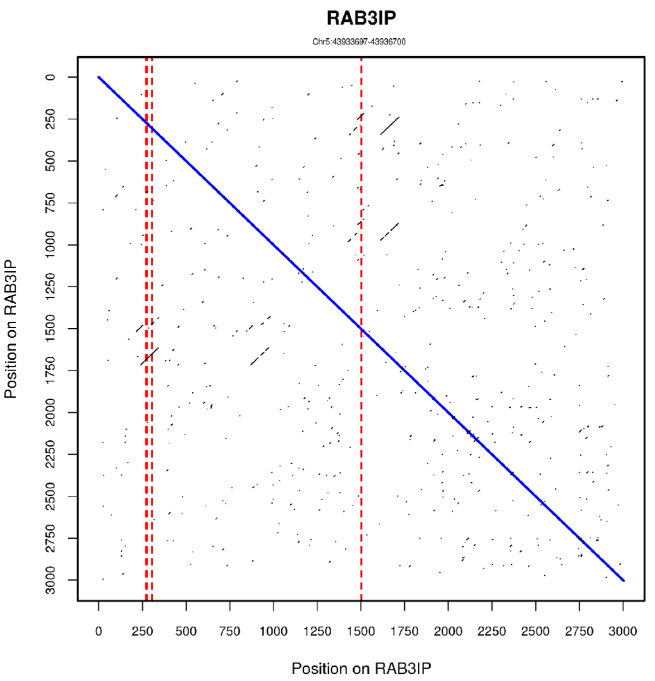
RAB3IP
Chr5:43933697-43936700
Position on RAB3IP
Position on RAB3IP

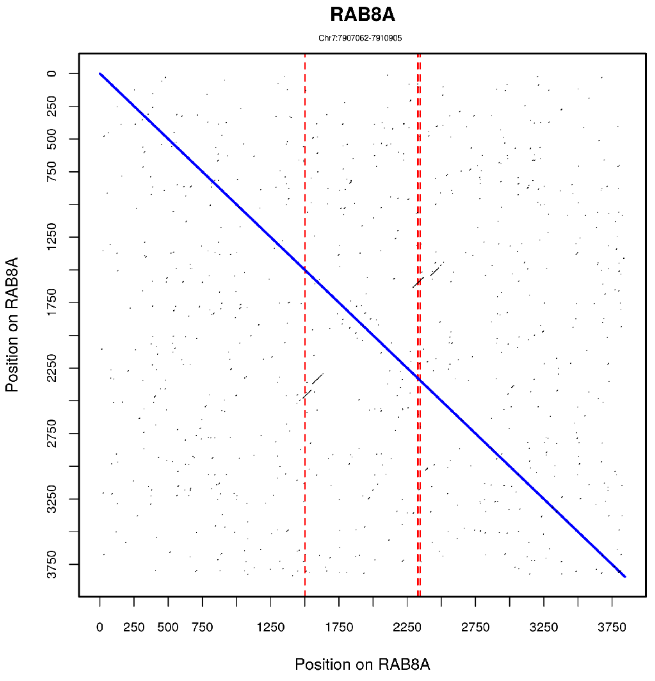
RAB8A
Chr7:7907062-7910905
Position on RAB8A
Position on RAB8A

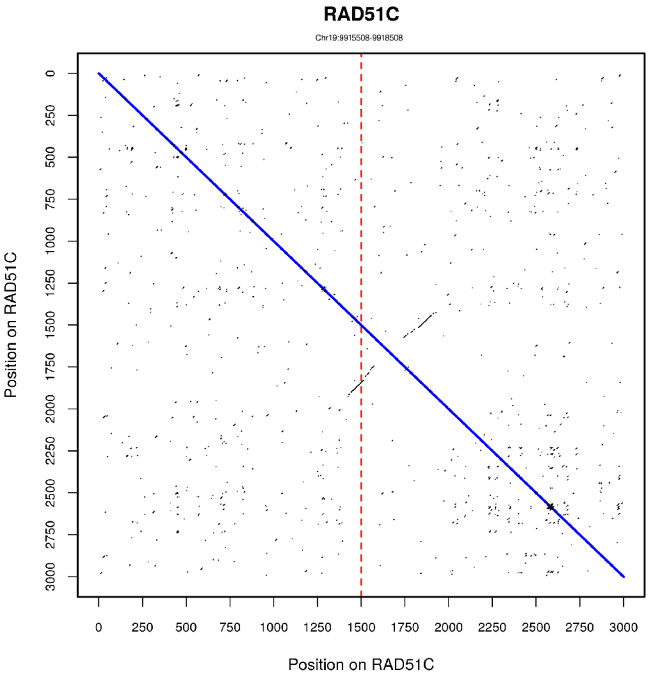
RAD51C
Chr19:9915508-9918508
Position on RAD51C
Position on RAD51C

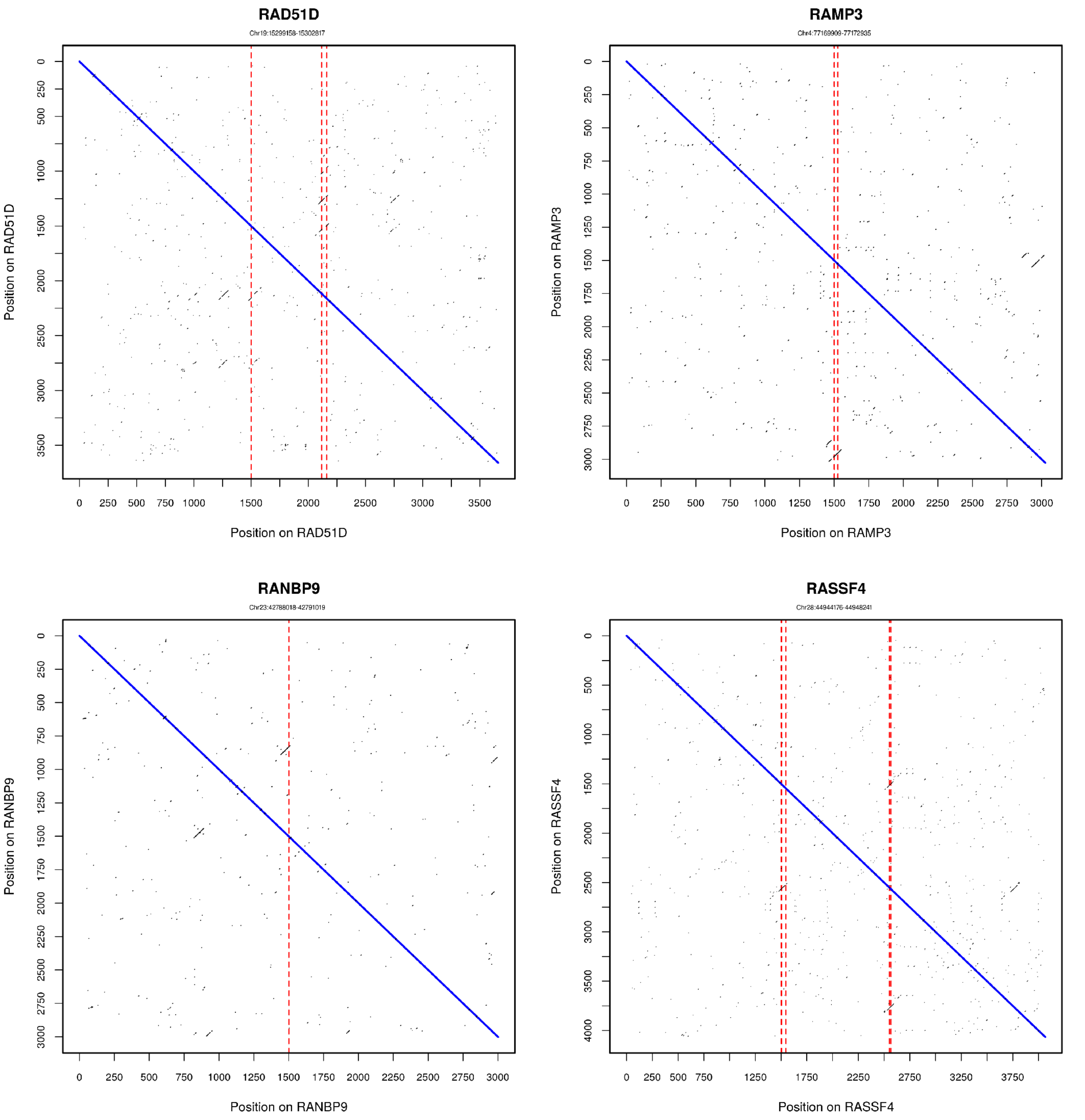
RAD51D
Chr19:15299158-15302817
Position on RAD51D
Position on RAD51D
RAMP3
Chr4:77169909-77172935
Position on RAMP3
Position on RAMP3
RANBP9
Chr23:42788018-42791019
Position on RANBP9
Position on RANBP9
RASSF4
Chr28:44944176-44948241
Position on RASSF4
Position on RASSF4

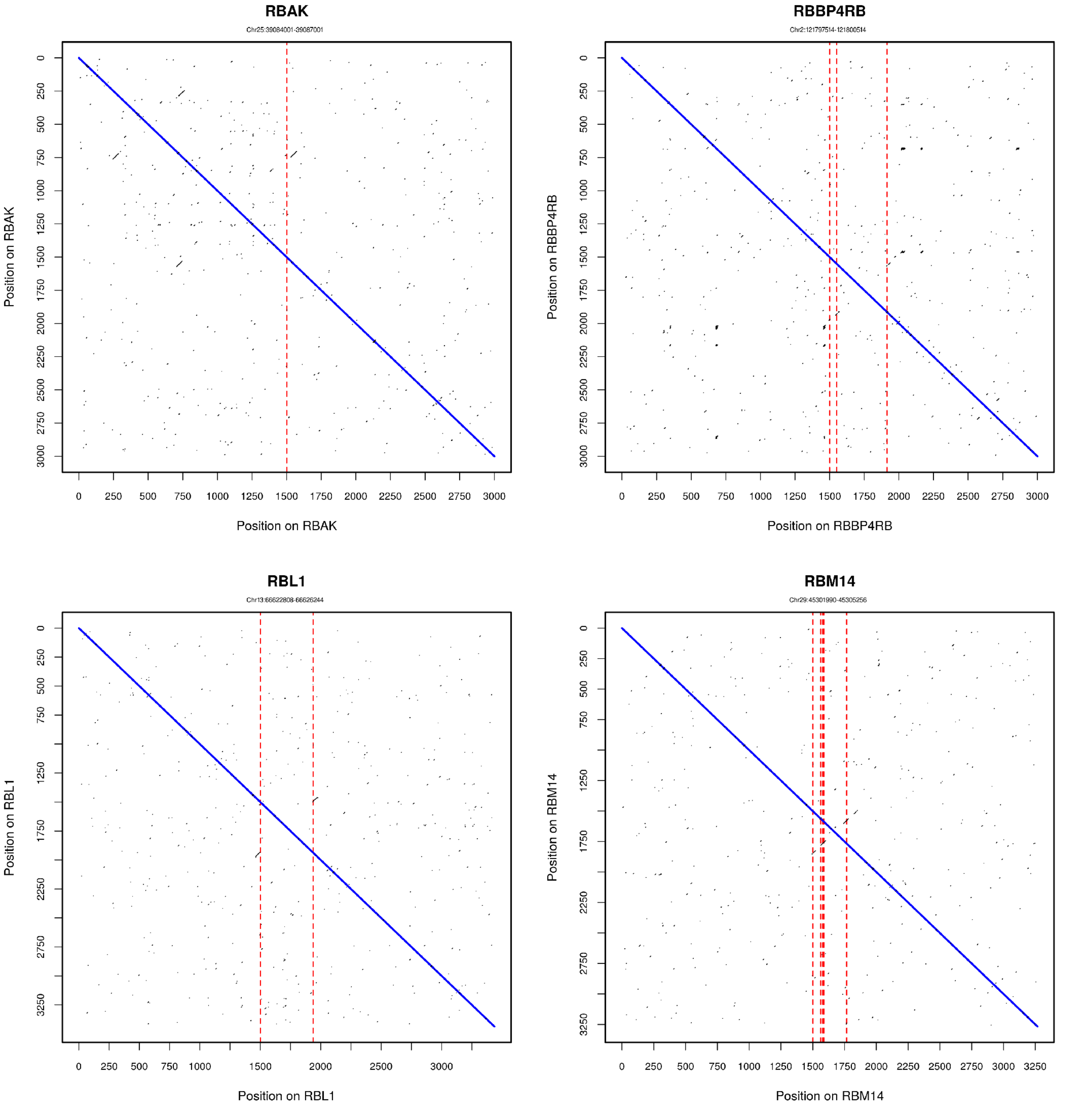

RBAK
Chr25:39084001-39087001
Position on RBAK
Position on RBAK
RBBP4RB
Chr2:121797514-121800514
Position on RBBP4RB
Position on RBBP4RB
RBL1
Chr13:66622808-66626244
Position on RBL1
Position on RBL1
RBM14
Chr29:45301990-45305256
Position on RBM14
Position on RBM14

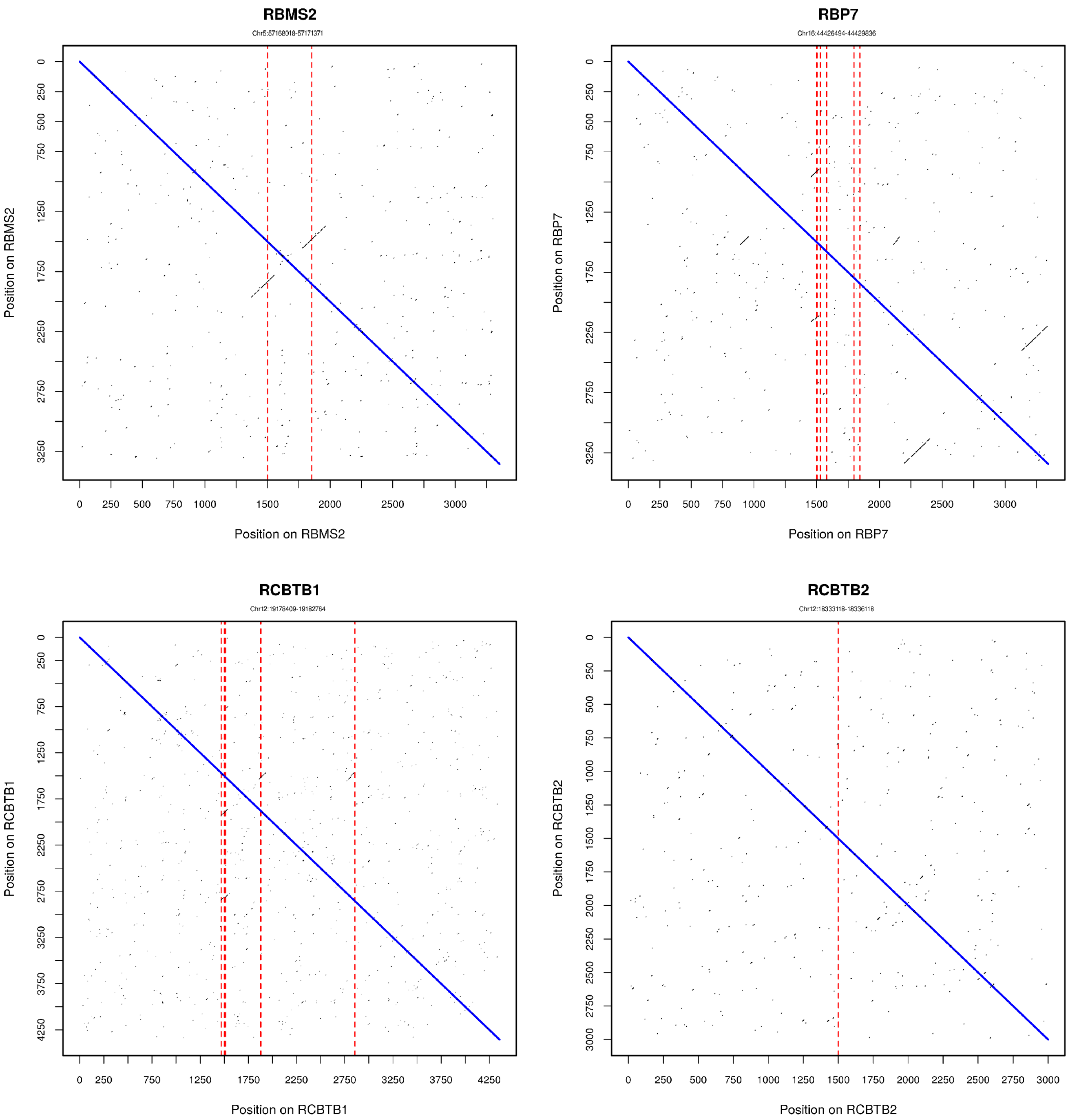
RBMS2
Chr5:57168018-57171371
Position on RBMS2
Position on RBMS2
RBP7
Chr16:44426494-44429836
Position on RBP7
Position on RBP7
RCBTB1
Chr12:19178409-19182764
Position on RCBTB1
Position on RCBTB1
RCBTB2
Chr12:18333118-18336118
Position on RCBTB2
Position on RCBTB2

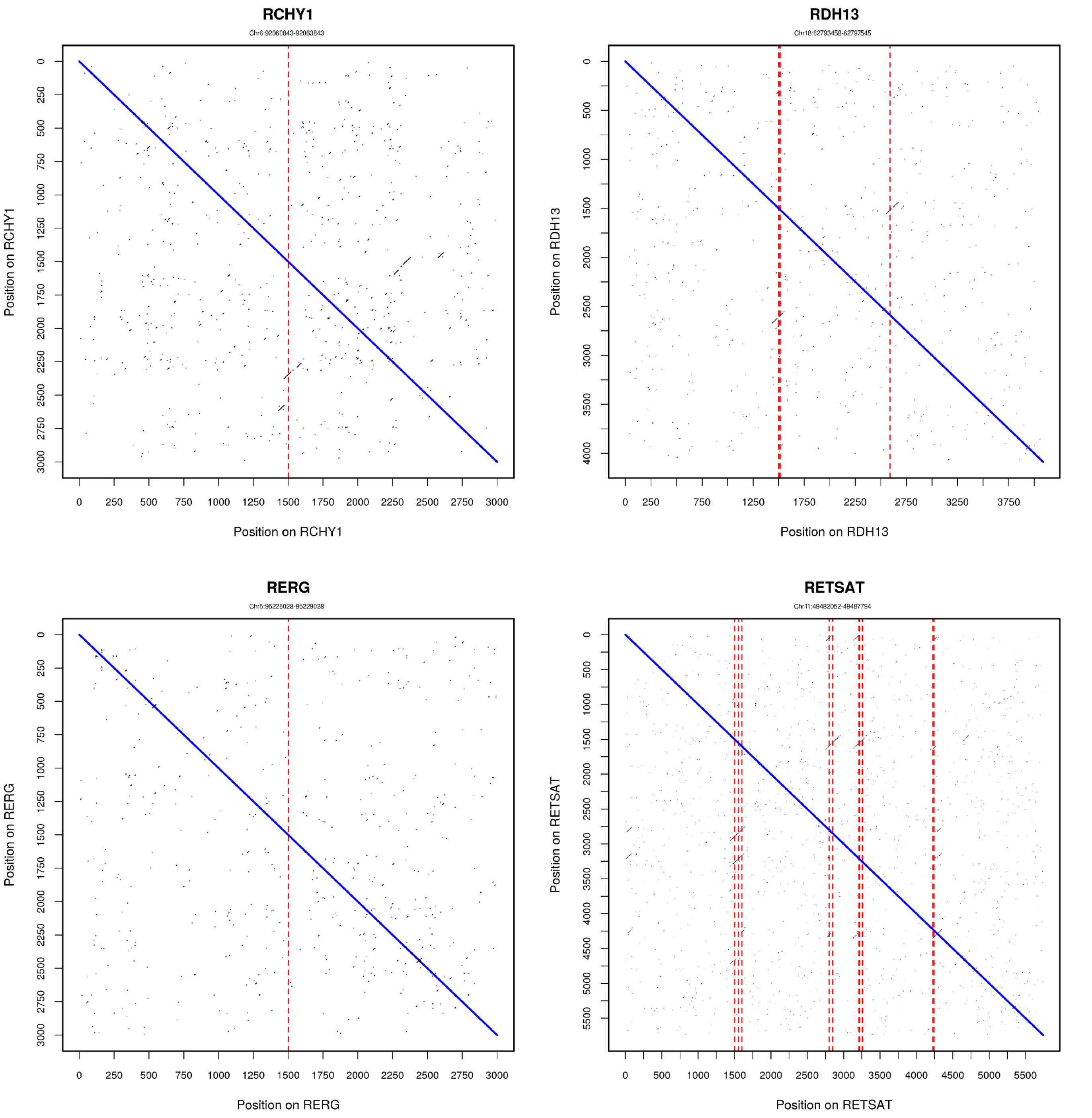
RCHY1
Chr6:92060843-92063843
Position on RCHY1
Position on RCHY1
RDH13
Chr18:62793458-62797545
Position on RDH13
Position on RDH13
RERG
Chr5:95226028-95229028
Position on RERG
Position on RERG
RETSAT
Chr11:49482052-49487794
Position on RETSAT
Position on RETSAT

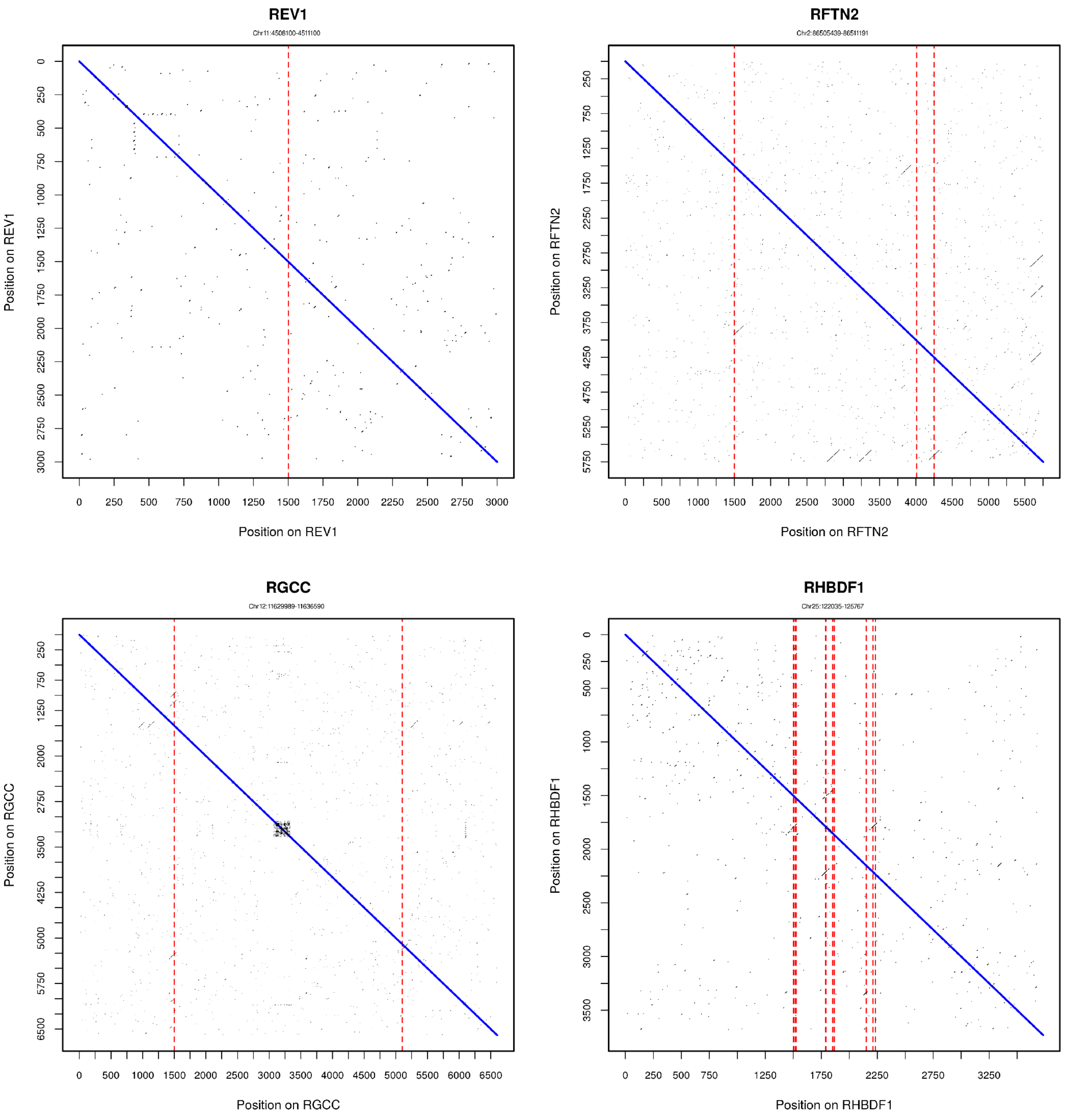
REV1
Chr11:4508100-4511100
Position on REV1
Position on REV1
RFTN2
Chr2:86505439-86511191
Position on RFTN2
Position on RFTN2
RGCC
Chr12:11629989-11636590
Position on RGCC
Position on RGCC
RHBDF1
Chr25:122035-125767
Position on RHBDF1
Position on RHBDF1

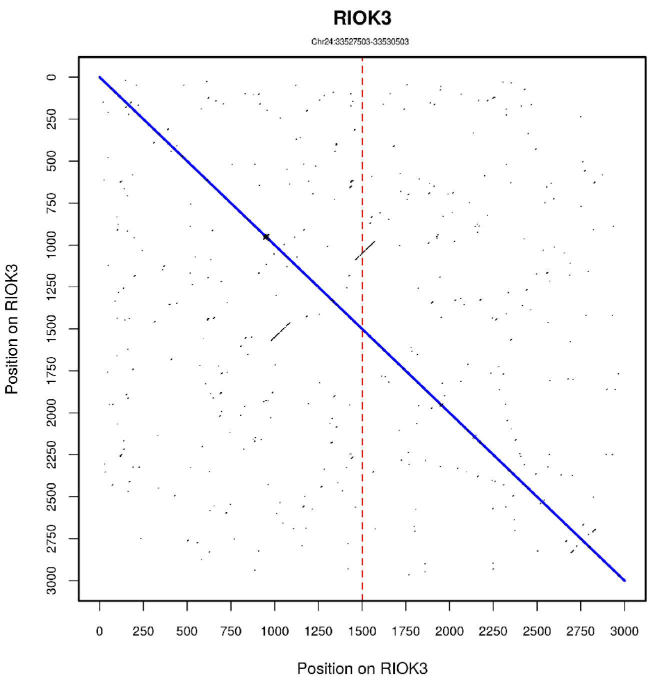
RIOK3
Chr24:33527503-33530503
Position on RIOK3
Position on RIOK3

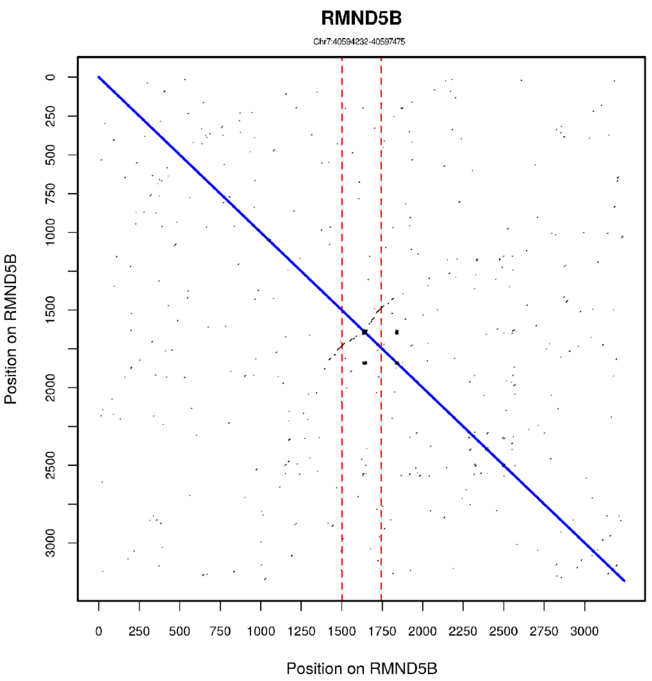
RMND5B
Chr7:40594232-40597475
Position on RMND5B
Position on RMND5B

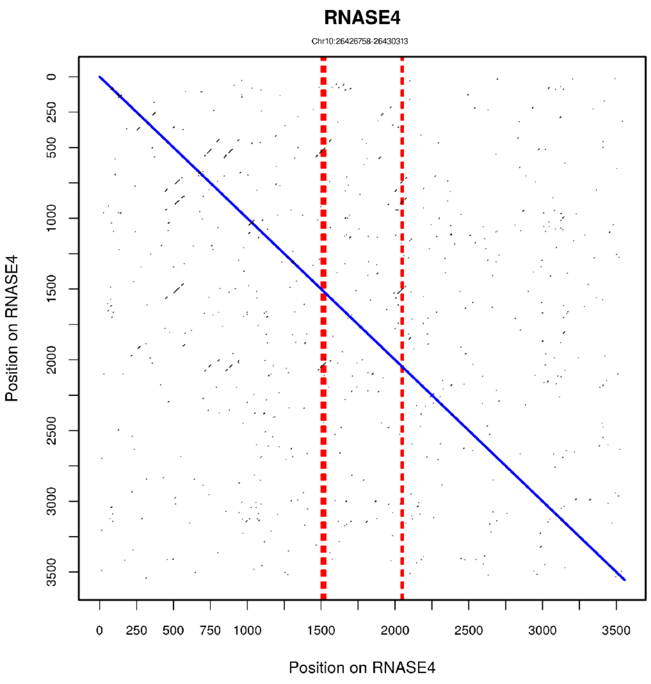
RNASE4
Chr10:26426758-26430313
Position on RNASE4
Position on RNASE4

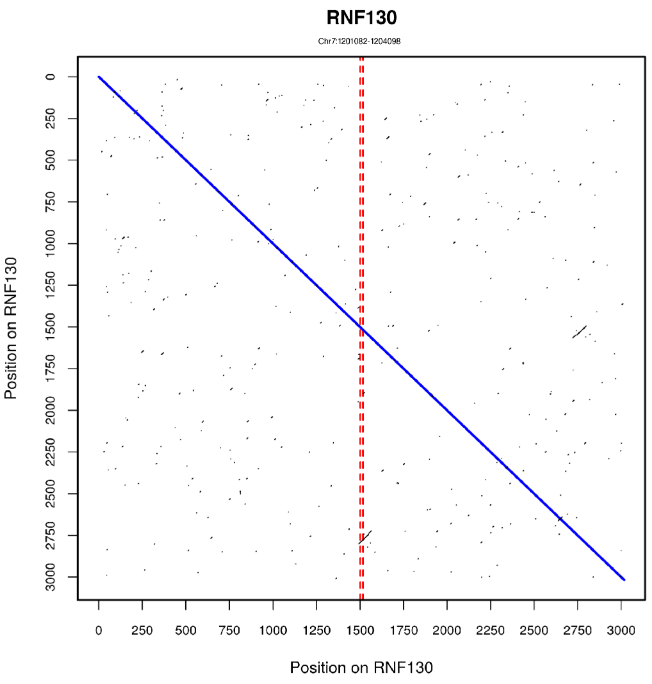
RNF130
Chr7:1201082-1204098
Position on RNF130
Position on RNF130

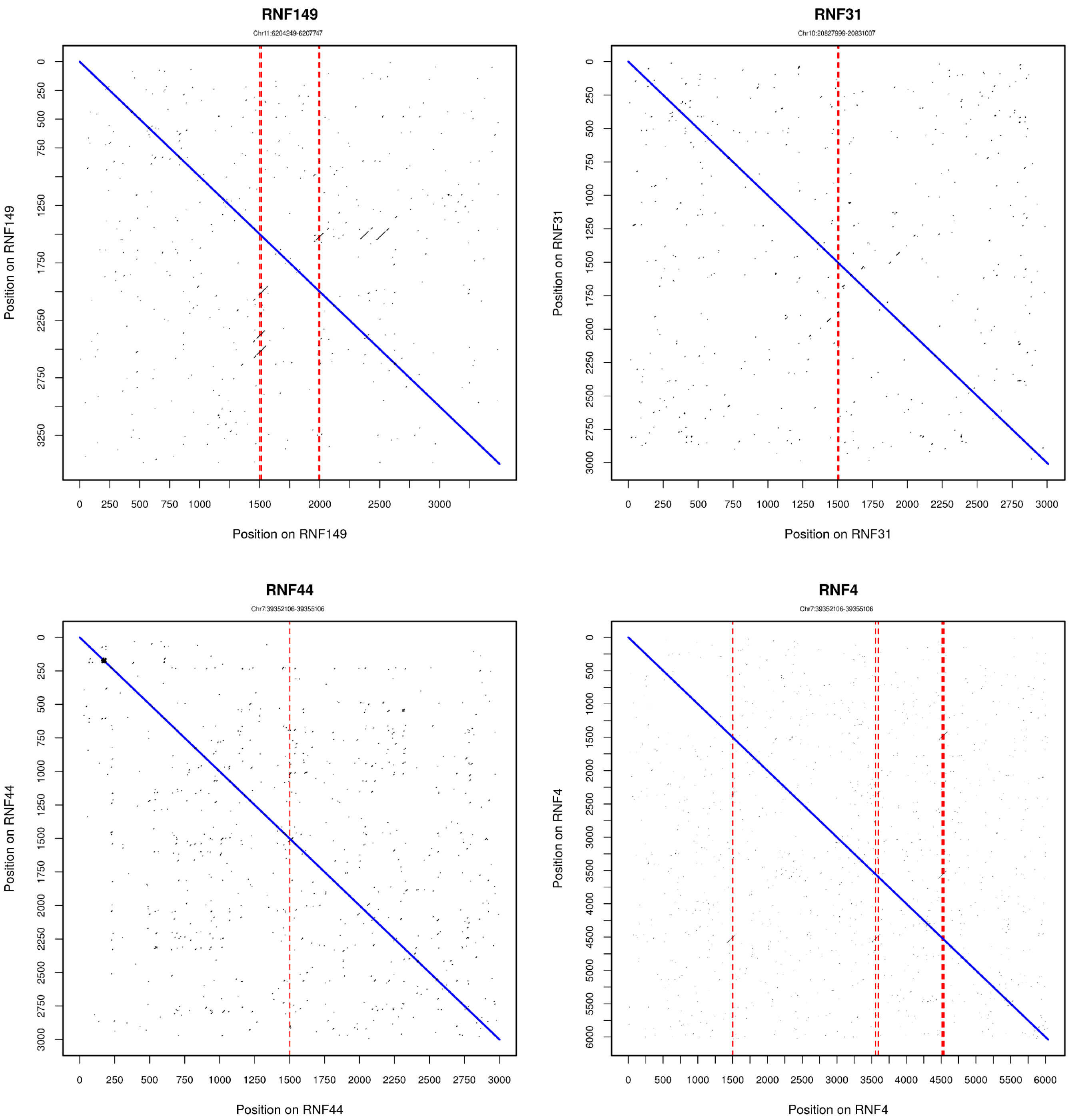
RNF149
Chr11:6204249-6207747
Position on RNF149
Position on RNF149
RNF31
Chr10:20827999-20831007
Position on RNF31
Position on RNF31
RNF44
Chr7:39352106-39355106
Position on RNF44
Position on RNF44
RNF4
Chr7:39352106-39355106
Position on RNF4
Position on RNF4

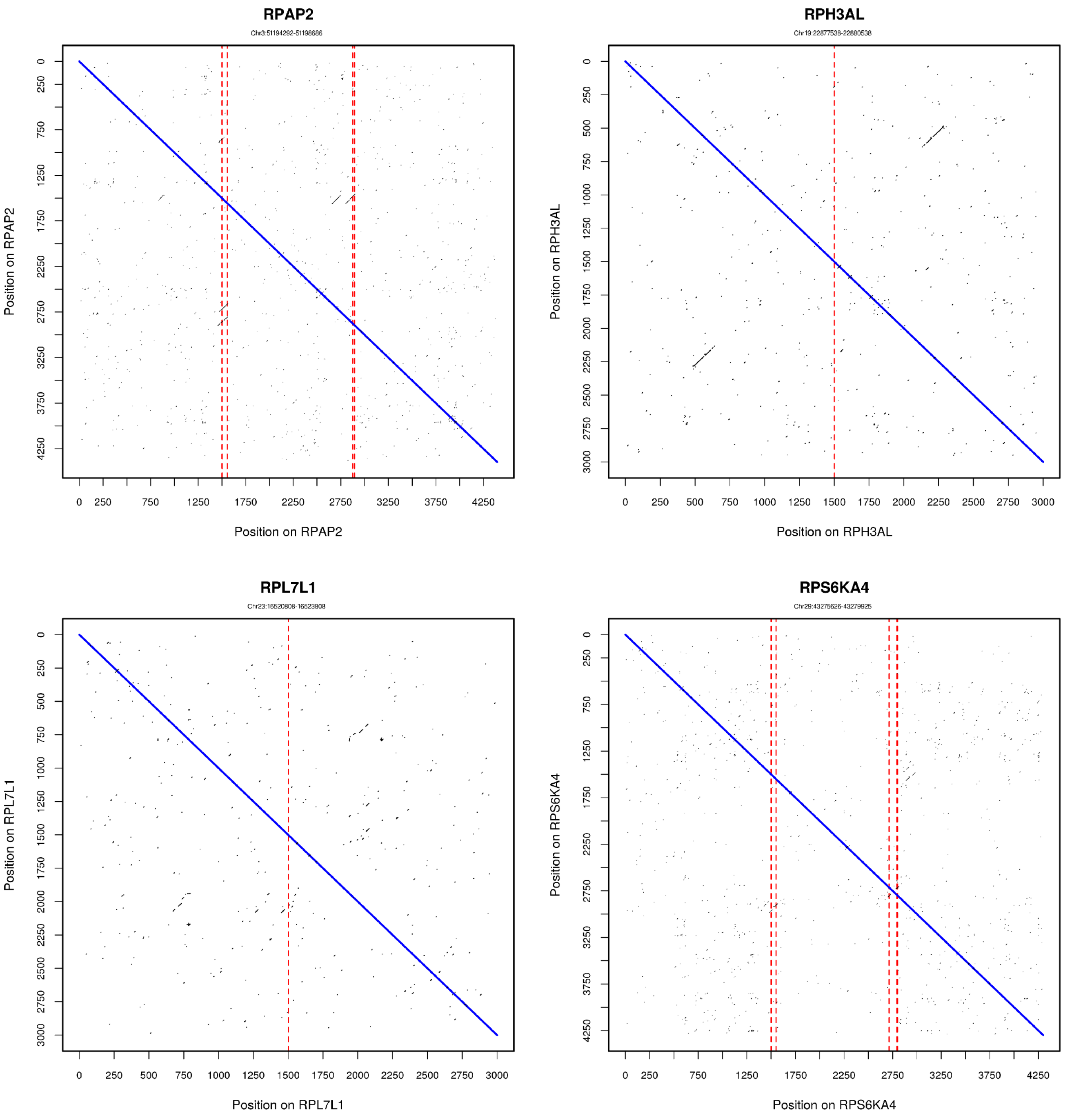

RPAP2
Chr3:51194292-51198686
Position on RPAP2
Position on RPAP2
RPH3AL
Chr19:22877538-22880538
Position on RPH3AL
Position on RPH3AL
RPL7L1
Chr23:16520808-16523808
Position on RPL7L1
Position on RPL7L1
RPS6KA4
Chr29:43275626-43279925
Position on RPS6KA4
Position on RPS6KA4

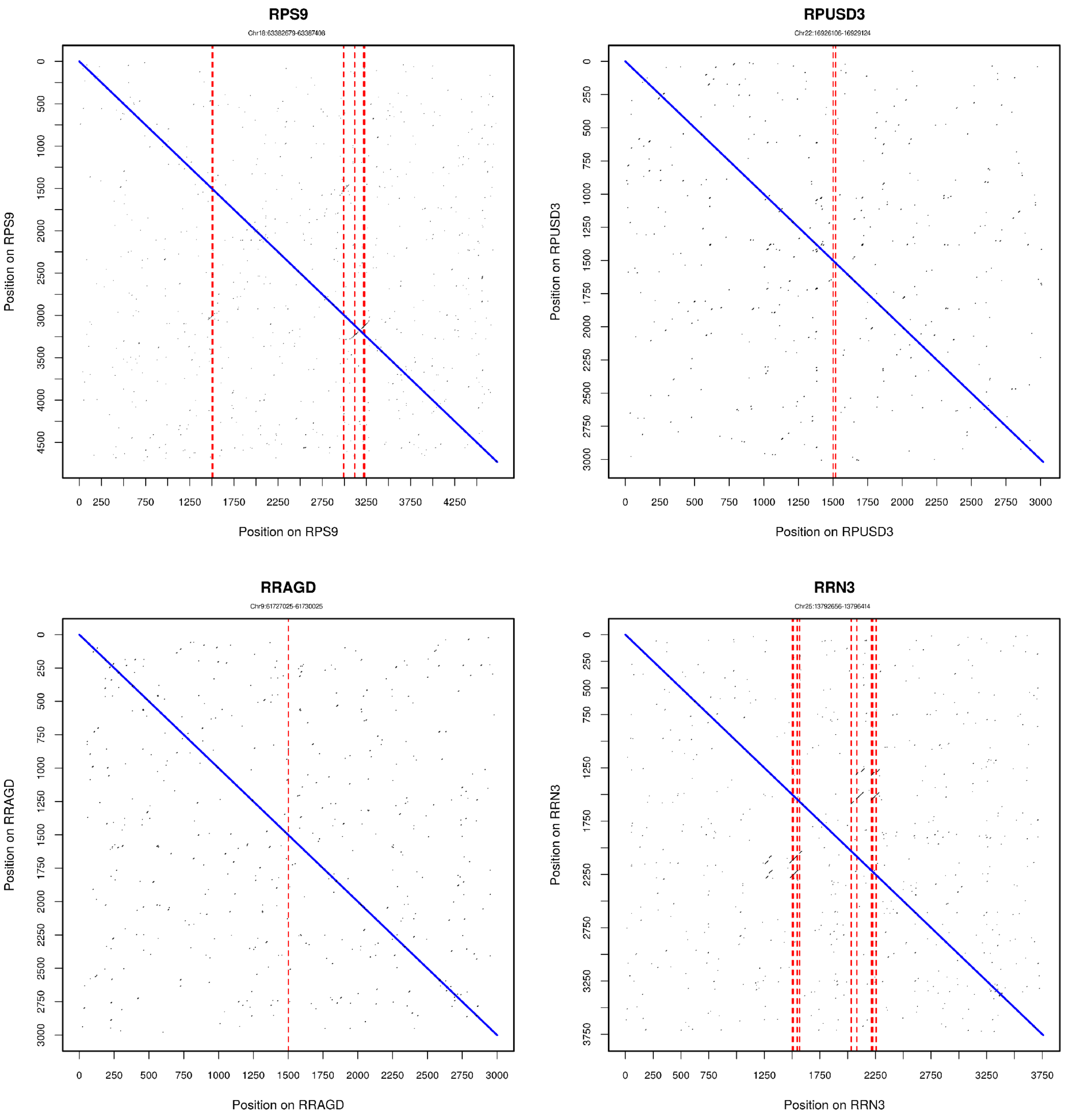
RPS9
Chr18:63382679-63387408
Position on RPS9
Position on RPS9
RPUSD3
Chr22:16926106-16929124
Position on RPUSD3
Position on RPUSD3
RRAGD
Chr9:61727025-61730025
Position on RRAGD
Position on RRAGD
RRN3
Chr25:13792656-13796414
Position on RRN3
Position on RRN3

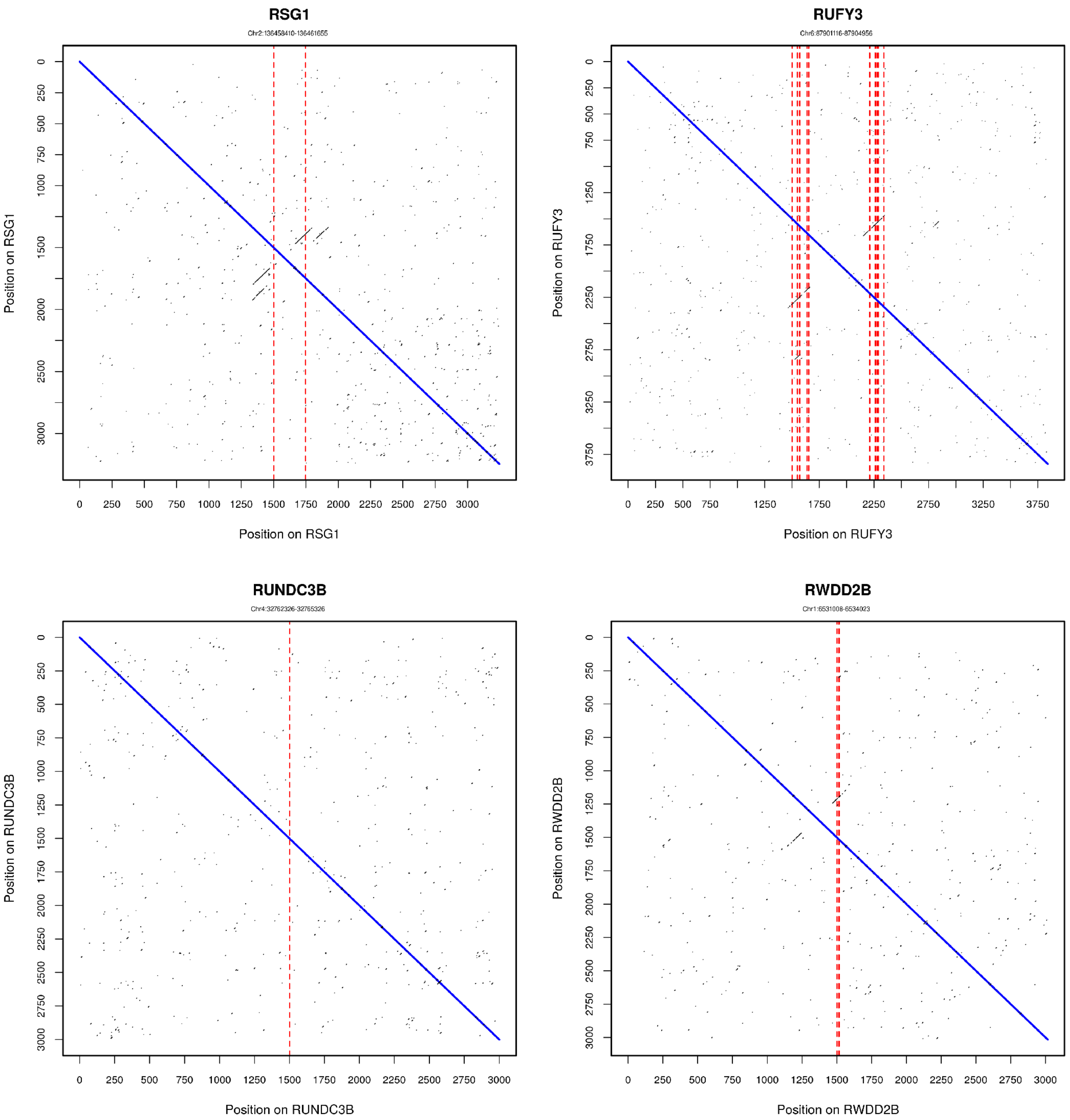

RSG1
Chr2:136458410-136461655
Position on RSG1
Position on RSG1
RUFY3
Chr6:87901116-87904956
Position on RUFY3
Position on RUFY3
RUNDC3B
Chr4:32762326-32765326
Position on RUNDC3B
Position on RUNDC3B
RWDD2B
Chr1:6531008-6534023
Position on RWDD2B
Position on RWDD2B

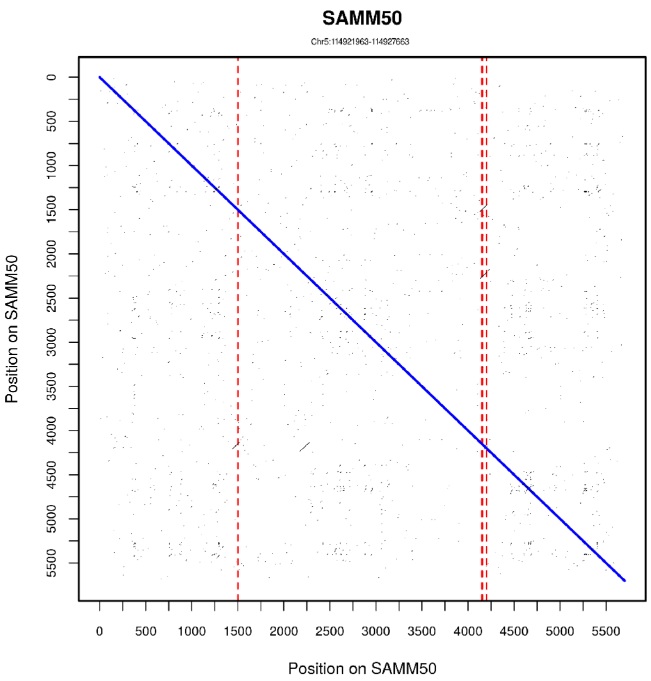
SAMM50
Chr5:114921963-114927663
Position on SAMM50
Position on SAMM50

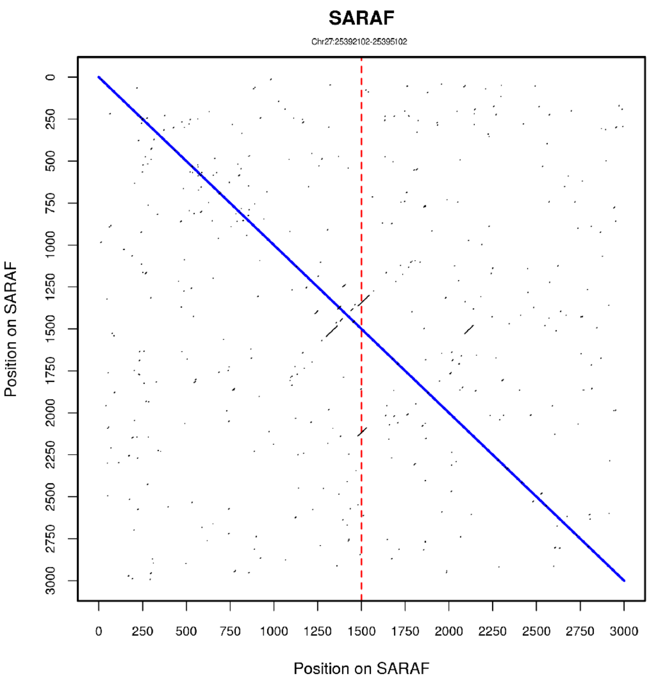
SARAF
Chr27:25392102-25395102
Position on SARAF
Position on SARAF

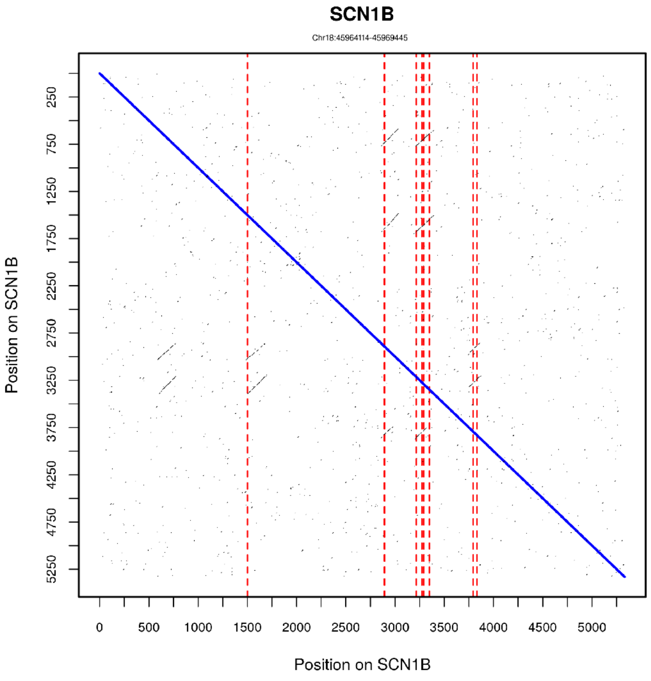
SCN1B
Chr18:45964114-45969445
Position on SCN1B
Position on SCN1B

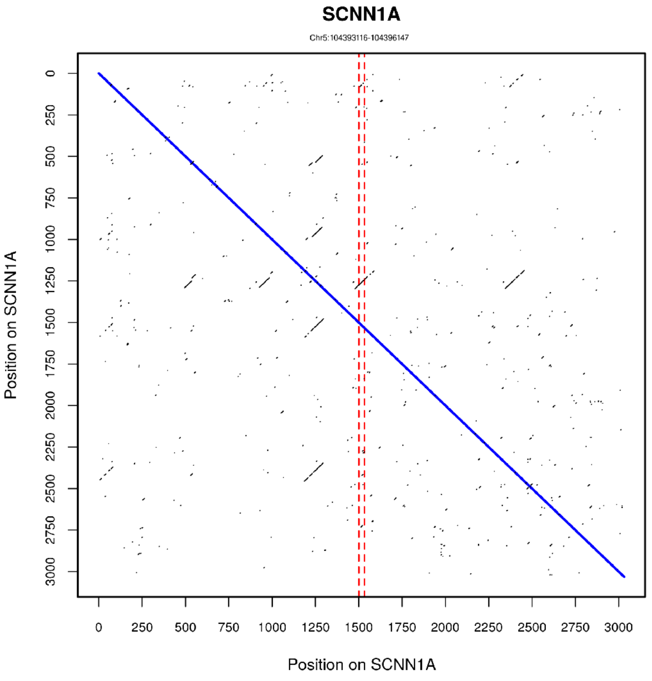
SCNN1A
Chr5:104393116-104396147
Position on SCNN1A
Position on SCNN1A

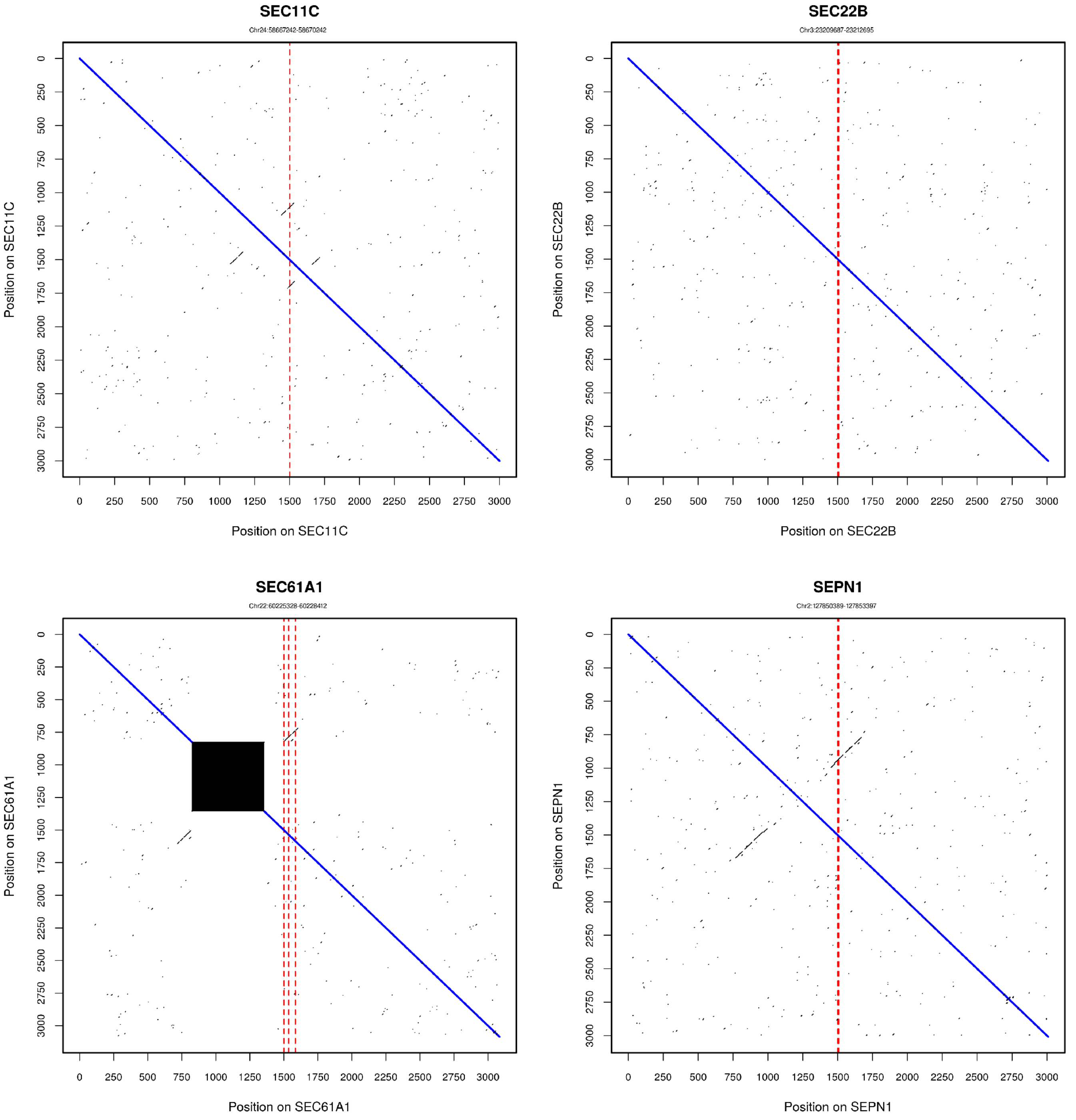
SEC11C
Chr24:58667242-58670242
Position on SEC11C
Position on SEC11C
SEC22B
Chr3:23209687-23212695
Position on SEC22B
Position on SEC22B
SEC61A1
Chr22:60225328-60228412
Position on SEC61A1
Position on SEC61A1
SEPN1
Chr2:127850389-127853397
Position on SEPN1
Position on SEPN1

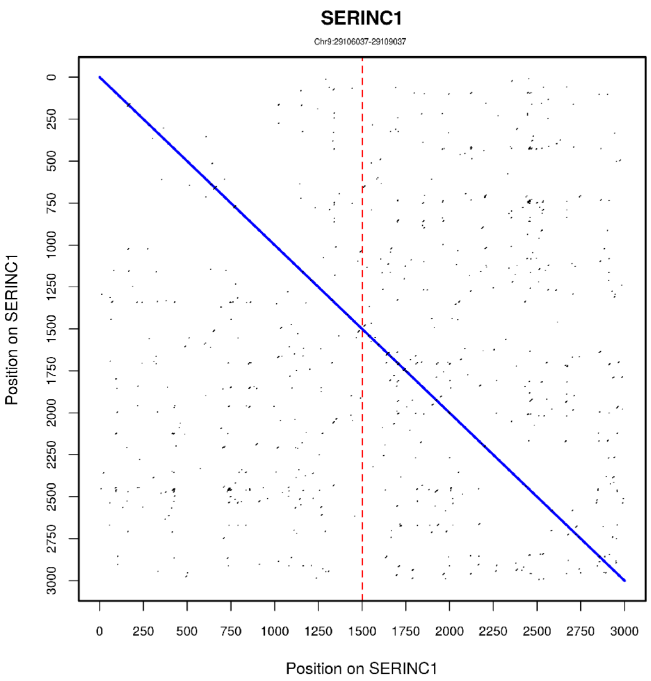
SERINC1
Chr9:29106037-29109037
Position on SERINC1
Position on SERINC1

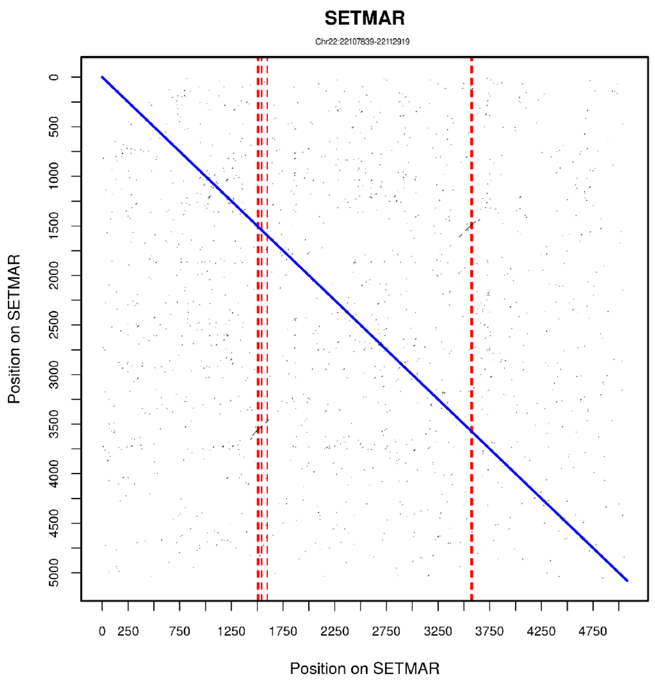
SETMAR
Chr22:22107839-22112919
Position on SETMAR
Position on SETMAR

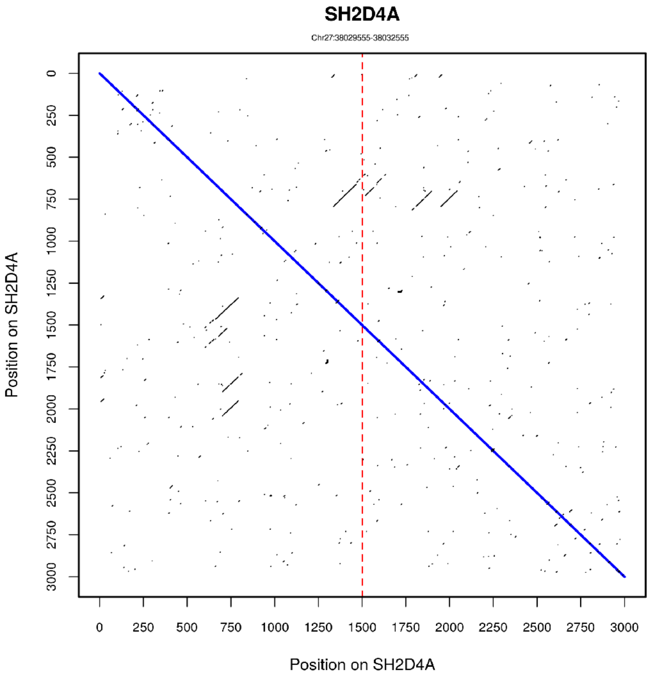
SH2D4A
Chr27:38029555-38032555
Position on SH2D4A
Position on SH2D4A

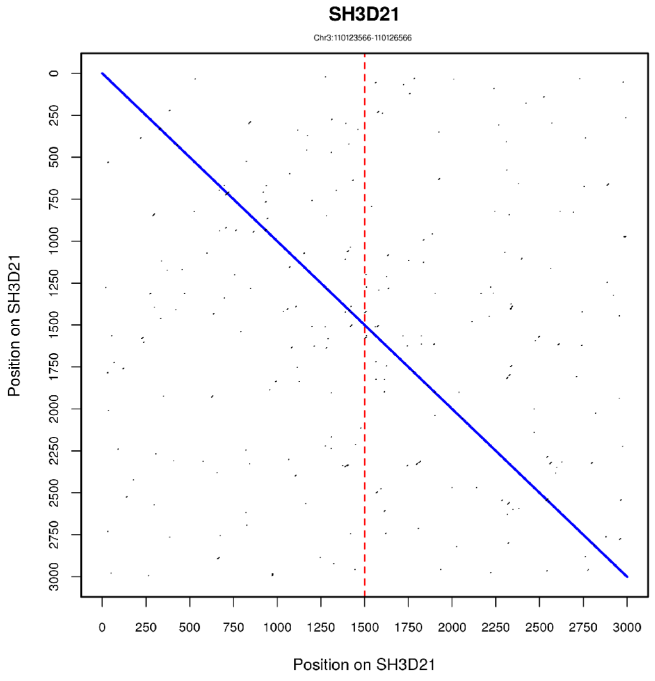
SH3D21
Chr3:110123566-110126566
Position on SH3D21
Position on SH3D21

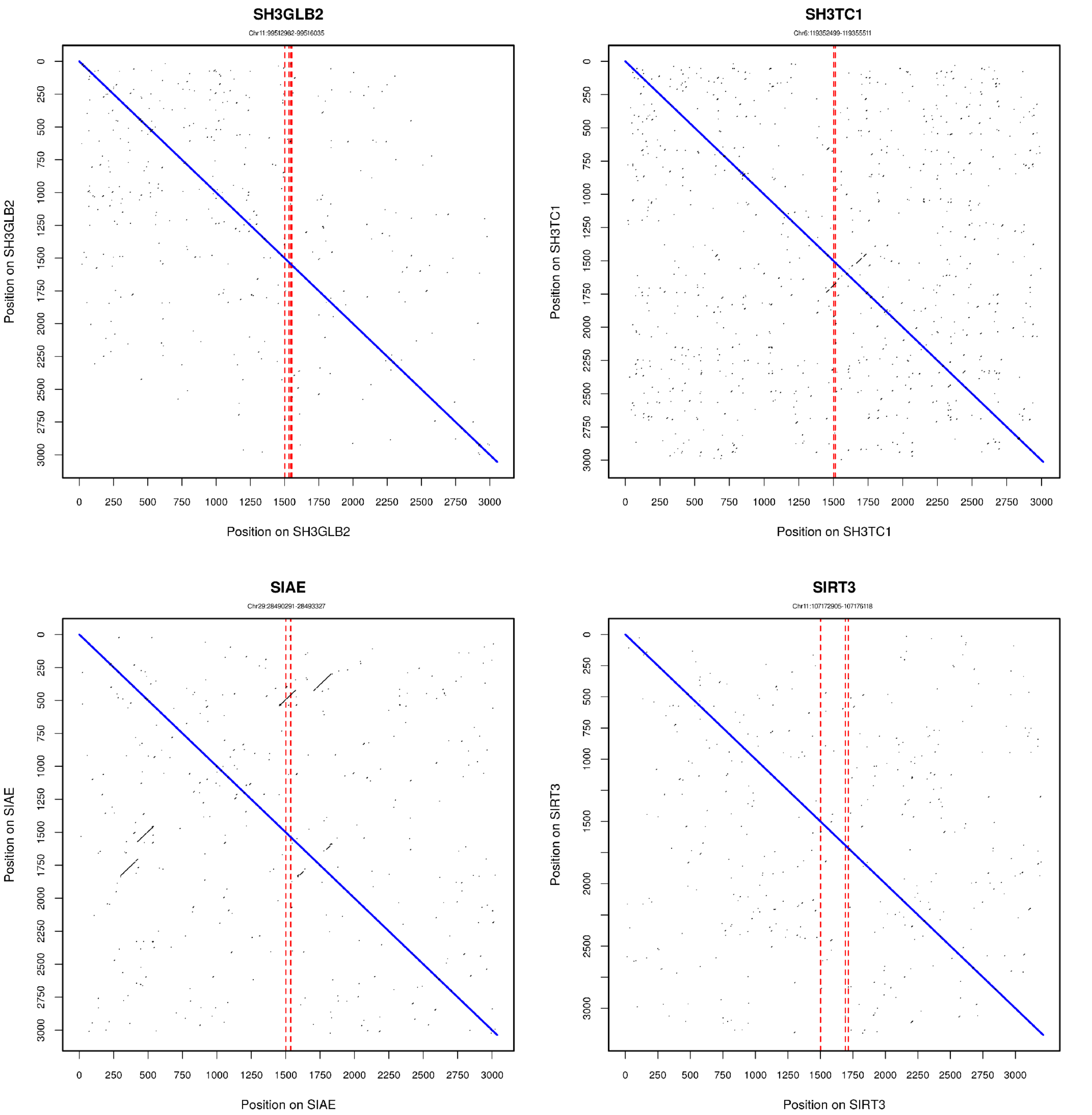
SH3GLB2
Chr11:99512982-99516035
Position on SH3GLB2
Position on SH3GLB2
SH3TC1
Chr6:119352499-119355511
Position on SH3TC1
Position on SH3TC1
SIAE
Chr29:28490291-28493327
Position on SIAE
Position on SIAE
SIRT3
Chr11:107172905-107176118
Position on SIRT3
Position on SIRT3

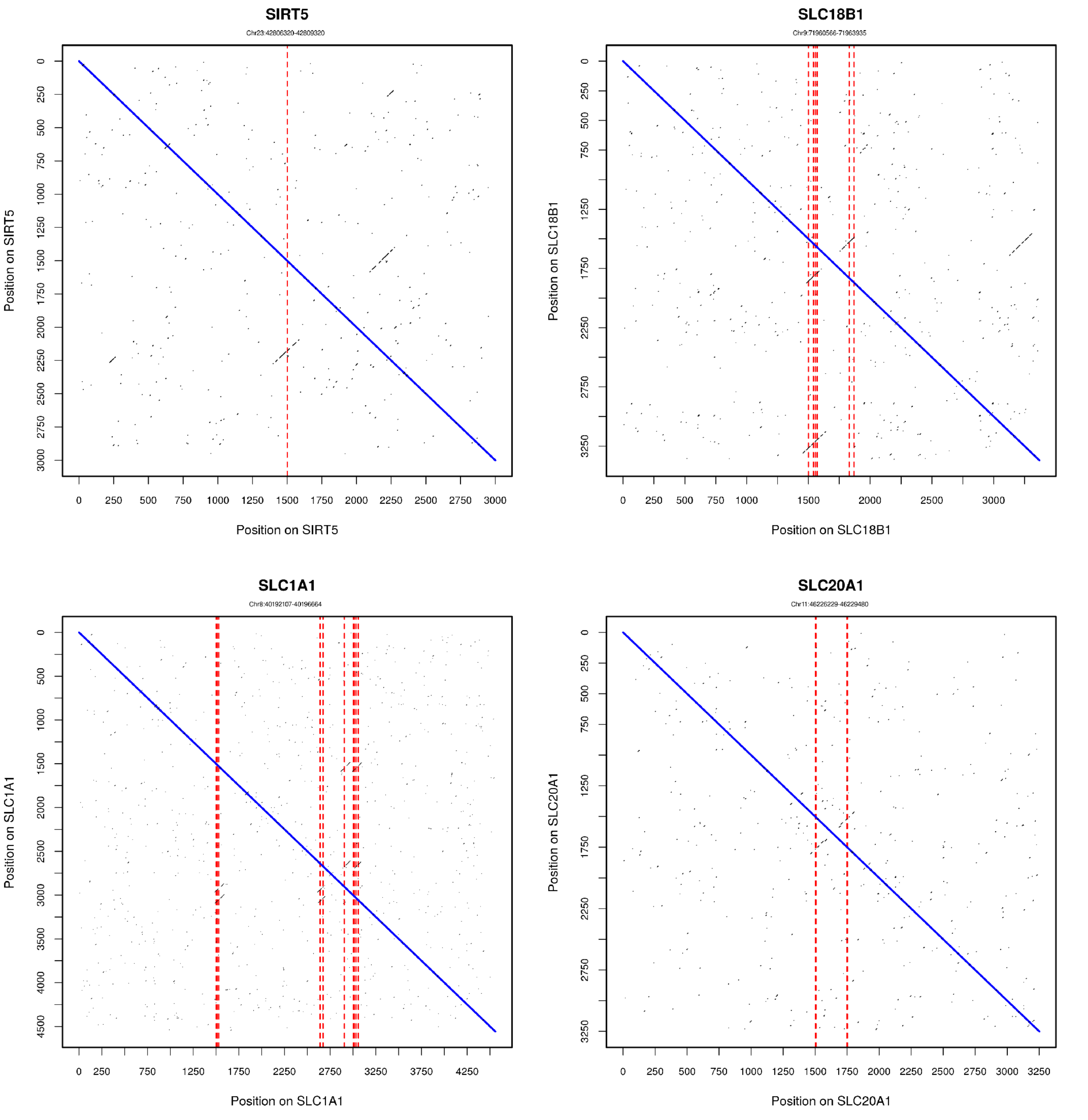

SIRT5
Chr23:42806320-42809320
Position on SIRT5
Position on SIRT5
SLC18B1
Chr9:71960566-71963935
Position on SLC18B1
Position on SLC18B1
SLC1A1
Chr8:40192107-40196664
Position on SLC1A1
Position on SLC1A1
SLC20A1
Chr11:46226229-46229480
Position on SLC20A1
Position on SLC20A1

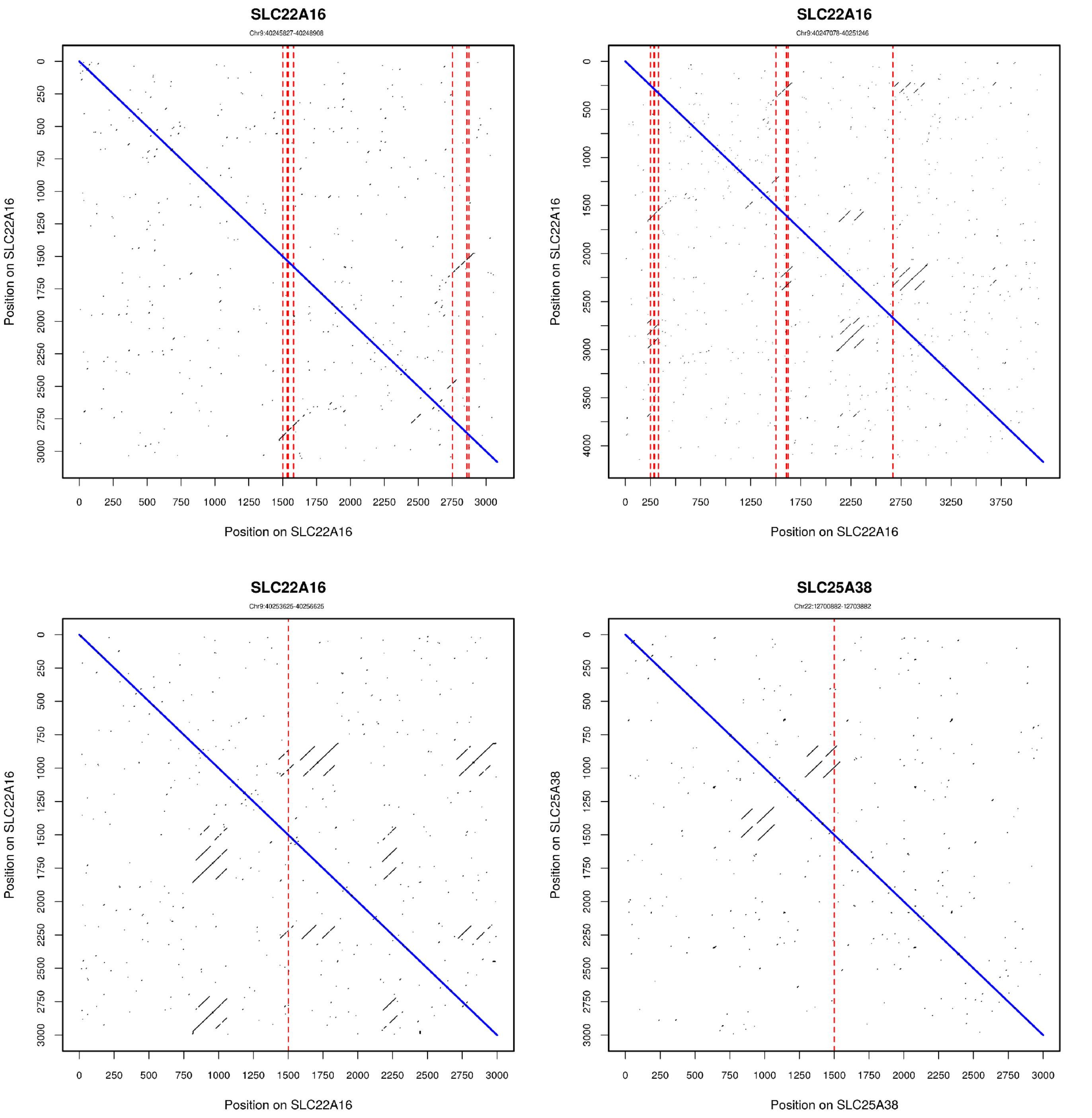
SLC22A16
Chr9:40245827-40248908
Position on SLC22A16
Position on SLC22A16
SLC22A16
Chr9:40247078-40251246
Position on SLC22A16
Position on SLC22A16
SLC22A16
Chr9:40253625-40256625
Position on SLC22A16
Position on SLC22A16
SLC25A38
Chr22:12700882-12703882
Position on SLC25A38
Position on SLC25A38

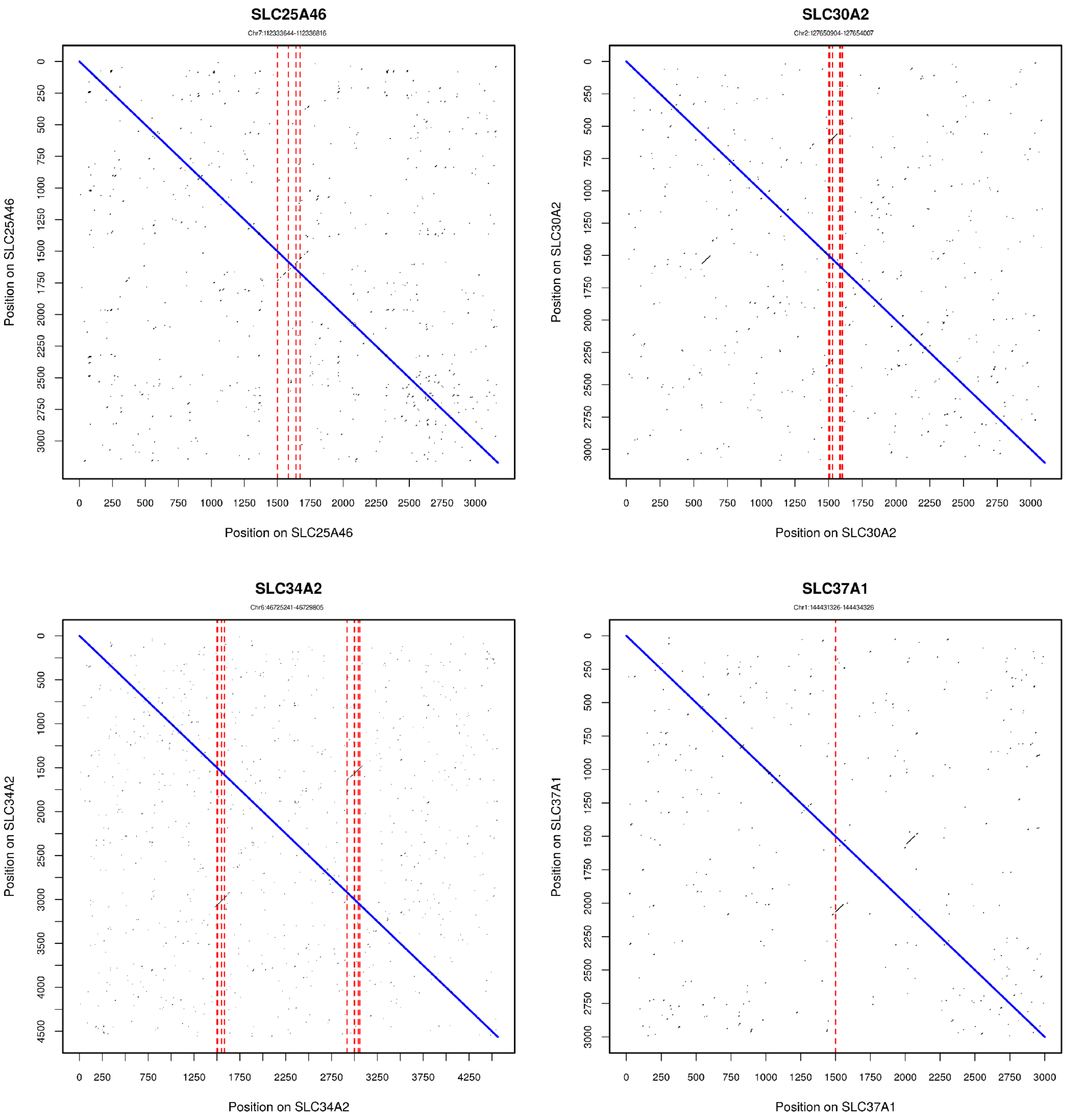
SLC25A46
Chr7:112333644-112336816
Position on SLC25A46
Position on SLC25A46
SLC30A2
Chr2:127650904-127654007
Position on SLC30A2
Position on SLC30A2
SLC34A2
Chr6:46725241-46729805
Position on SLC34A2
Position on SLC34A2
SLC37A1
Chr1:144431326-144434326
Position on SLC37A1
Position on SLC37A1

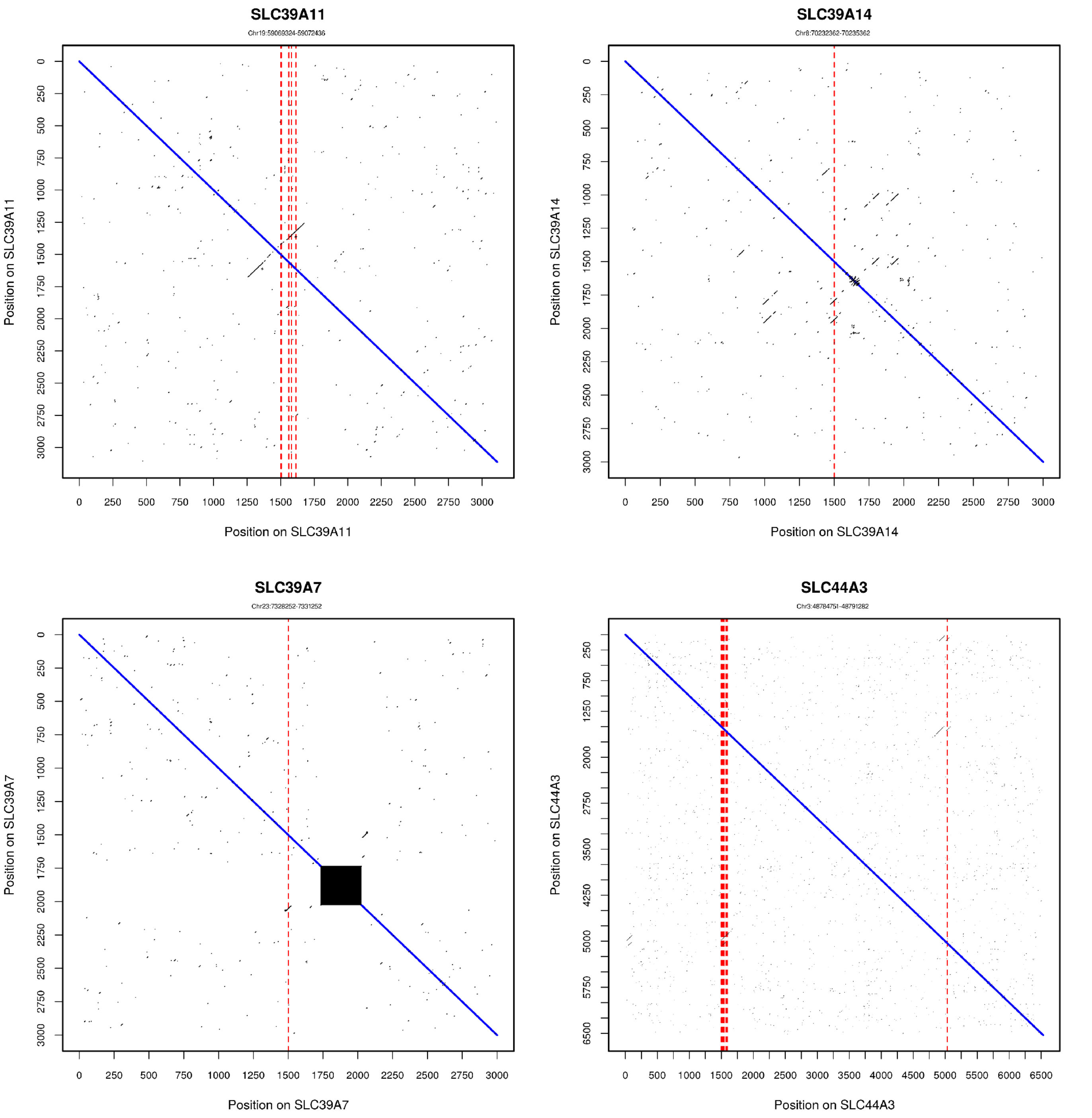

SLC39A11
Chr19:59069324-59072436
Position on SLC39A11
Position on SLC39A11
SLC39A14
Chr8:70232362-70235362
Position on SLC39A14
Position on SLC39A14
SLC39A7
Chr23:7328252-7331252
Position on SLC39A7
Position on SLC39A7
SLC44A3
Chr3:48784751-48791282
Position on SLC44A3
Position on SLC44A3

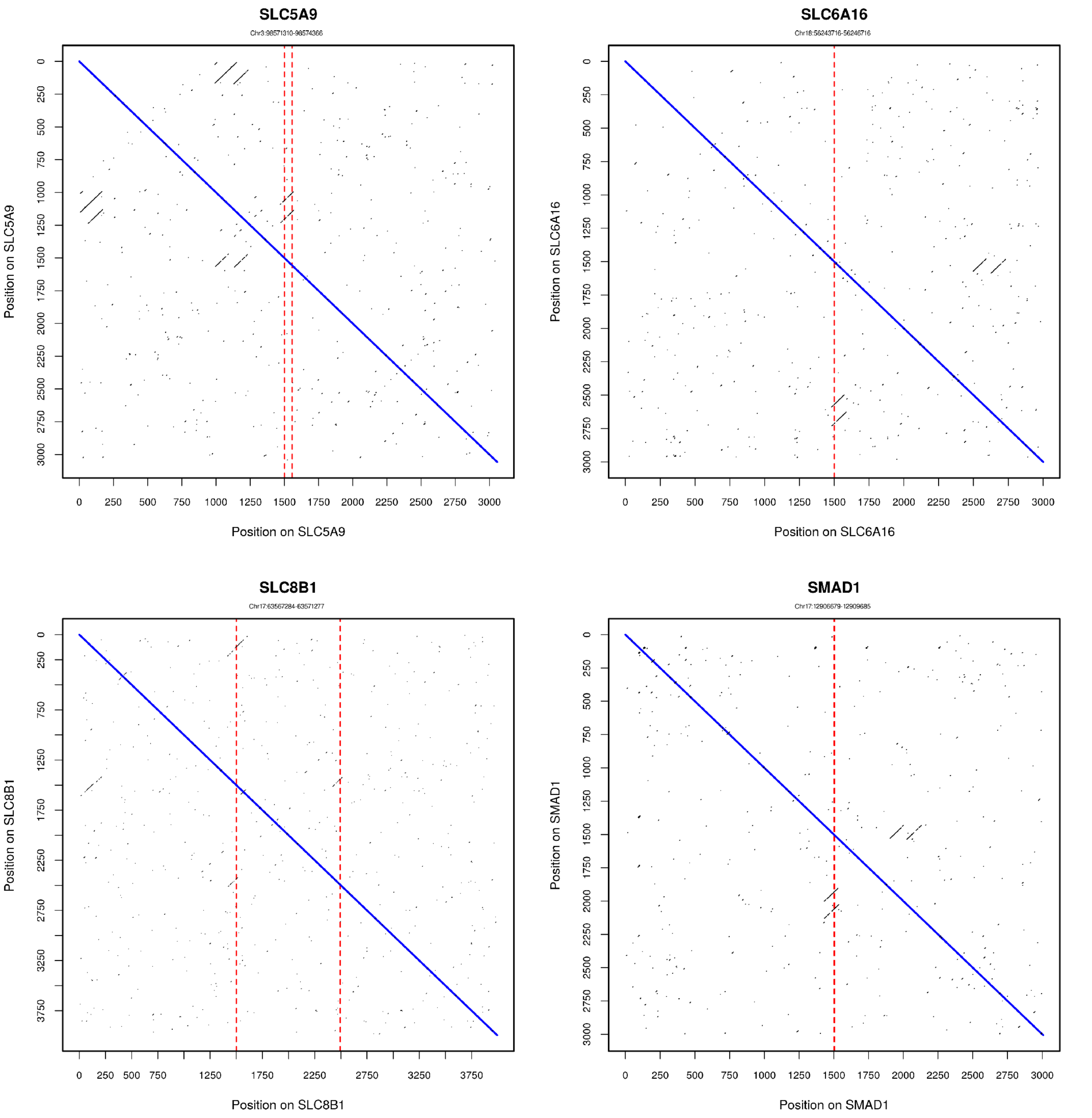
SLC5A9
Chr3:98571310-98574366
Position on SLC5A9
Position on SLC5A9
SLC6A16
Chr18:56243716-56246716
Position on SLC6A16
Position on SLC6A16
SLC8B1
Chr17:63567284-63571277
Position on SLC8B1
Position on SLC8B1
SMAD1
Chr17:12906679-12909685
Position on SMAD1
Position on SMAD1

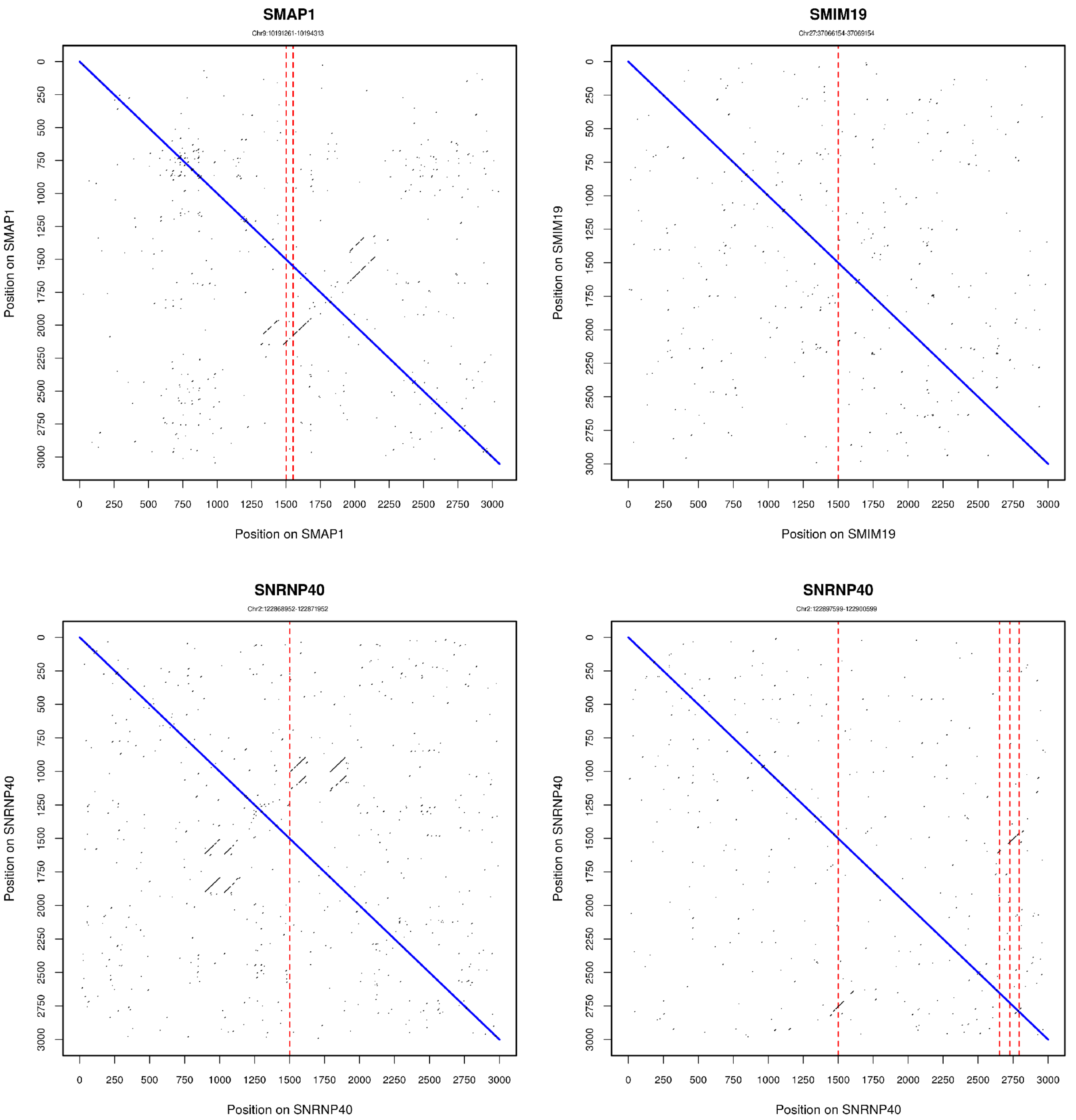
SMAP1
Chr9:10191261-10194313
Position on SMAP1
Position on SMAP1
SMIM19
Chr27:37066154-37069154
Position on SMIM19
Position on SMIM19
SNRNP40
Chr2:122868952-122871952
Position on SNRNP40
Position on SNRNP40
SNRNP40
Chr2:122897599-122900599
Position on SNRNP40
Position on SNRNP40

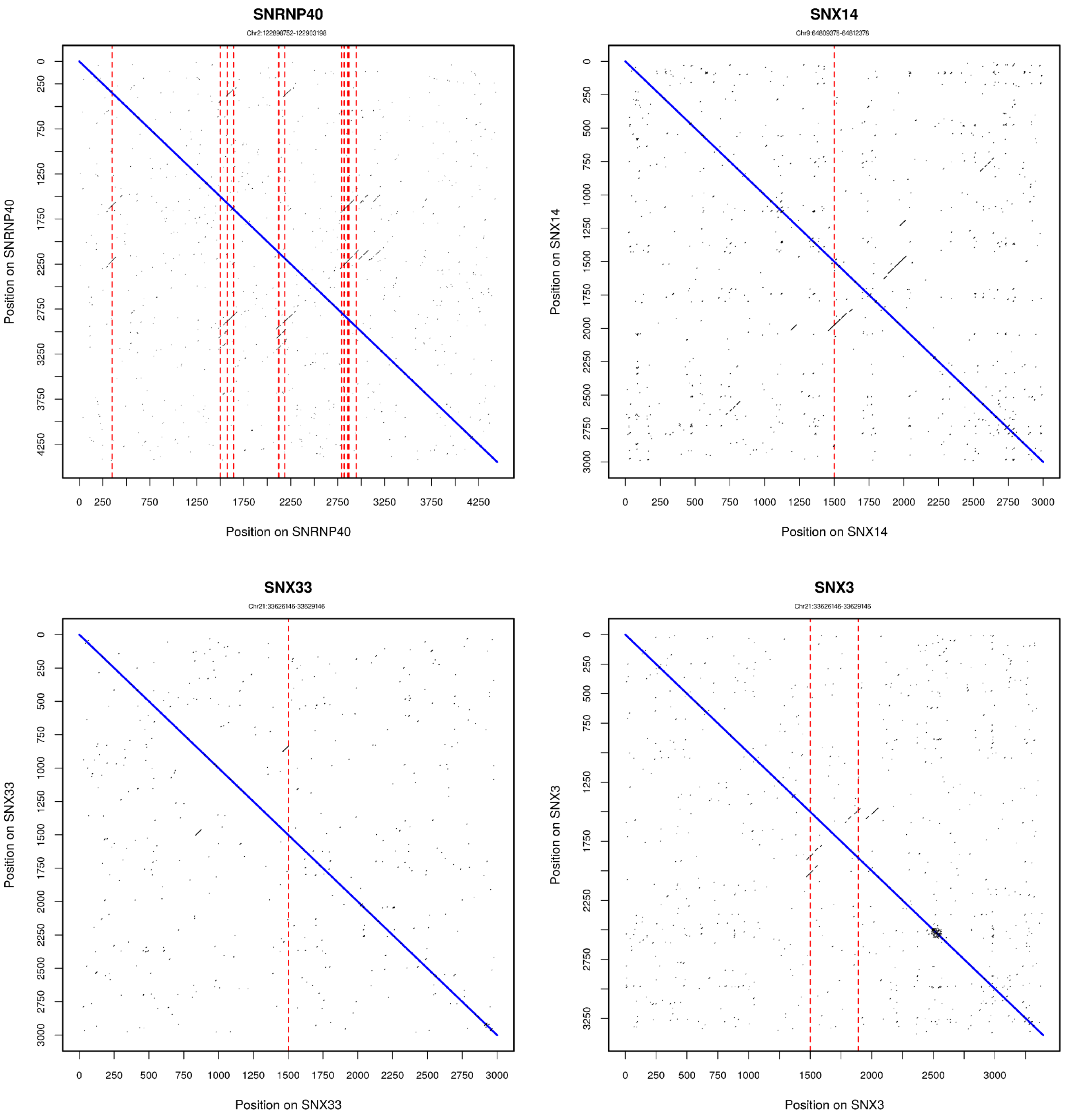
SNRNP40
Chr2:122898752-122903198
0 250 750 1250 1750 2250 2750 3250 3750 4250
Position on SNRNP40
Position on SNRNP40
SNX14
Chr9:64809378-64812378
0 250 500 750 1000 1250 1500 1750 2000 2250 2500 2750 3000
Position on SNX14
Position on SNX14
SNX33
Chr21:33626146-33629146
0 250 500 750 1000 1250 1500 1750 2000 2250 2500 2750 3000
Position on SNX33
Position on SNX33
SNX3
Chr21:33626146-33629146
0 250 500 750 1000 1500 2000 2500 3000
0 250 500 750 1250 1750 2250 2750 3250
Position on SNX3
Position on SNX3

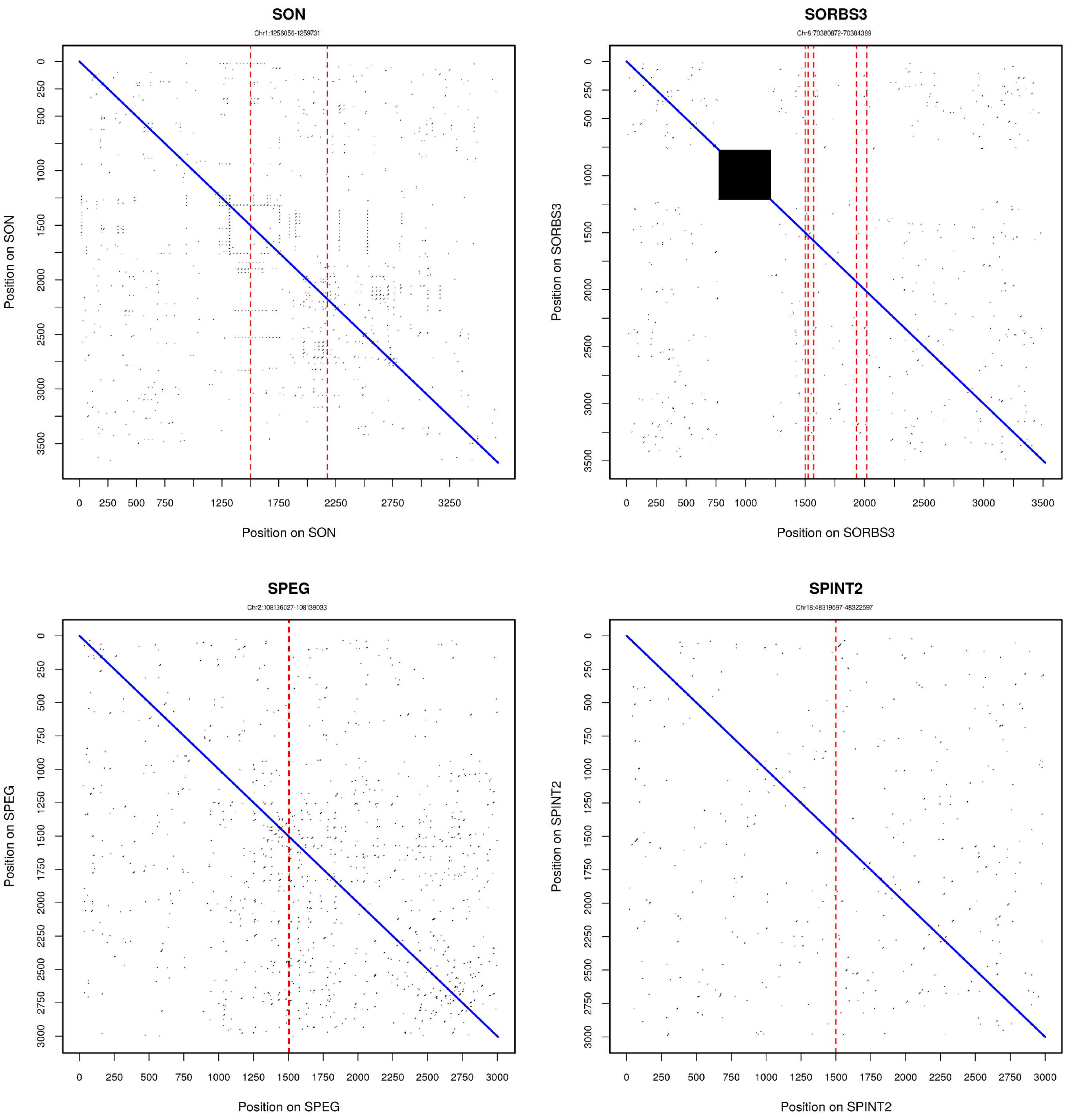
SON
Chr1:1256056-1259731
Position on SON
Position on SON
SORBS3
Chr8:70380872-70384389
Position on SORBS3
Position on SORBS3
SPEG
Chr2:108136027-108139033
Position on SPEG
Position on SPEG
SPINT2
Chr18:48319597-48322597
Position on SPINT2
Position on SPINT2

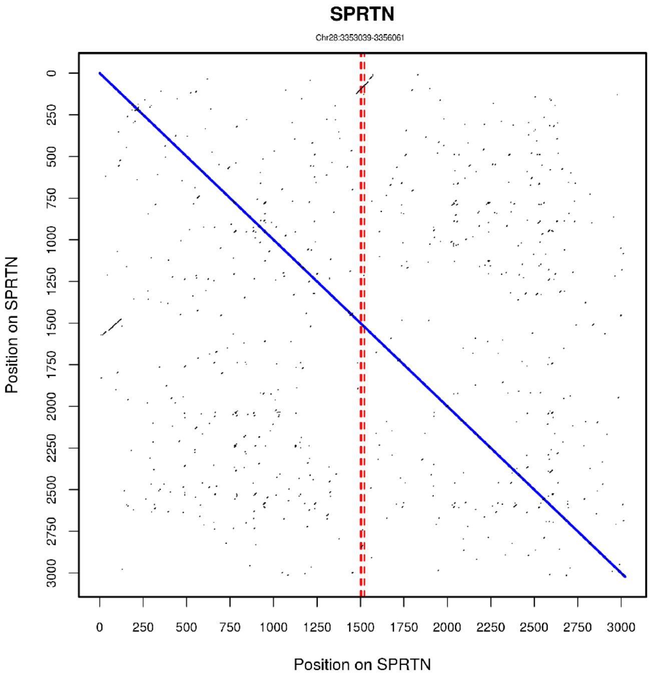
SPRTN
Chr28:3353039-3356061
Position on SPRTN
Position on SPRTN

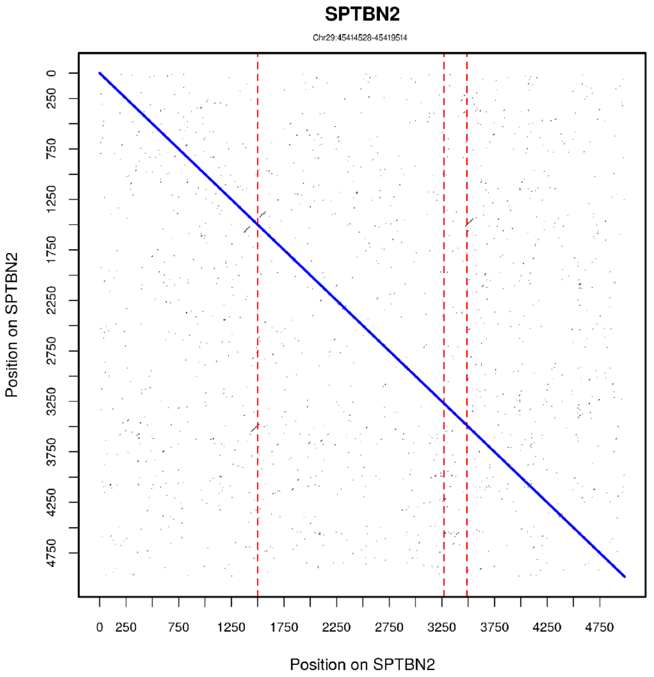
SPTBN2
Chr29:45414528-45419514
Position on SPTBN2
Position on SPTBN2

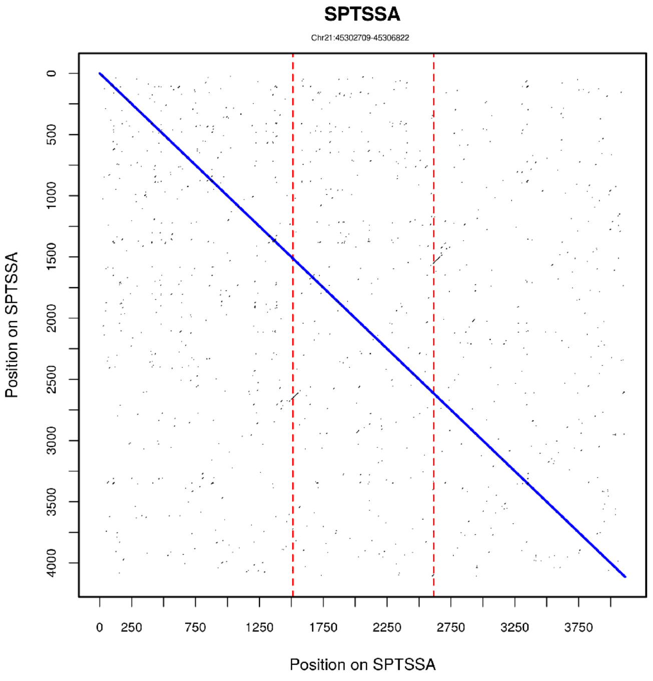
SPTSSA
Chr21:45302709-45306822
Position on SPTSSA
Position on SPTSSA

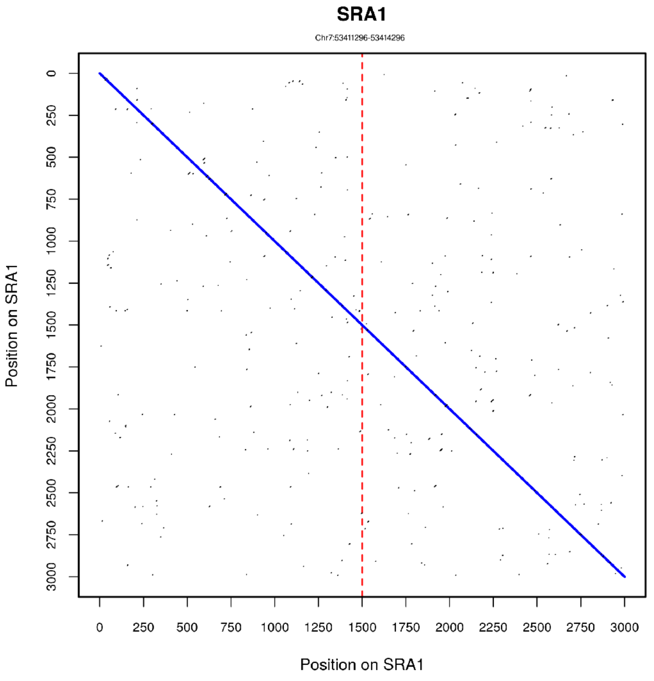
SRA1
Chr7:53411296-53414296
Position on SRA1
Position on SRA1

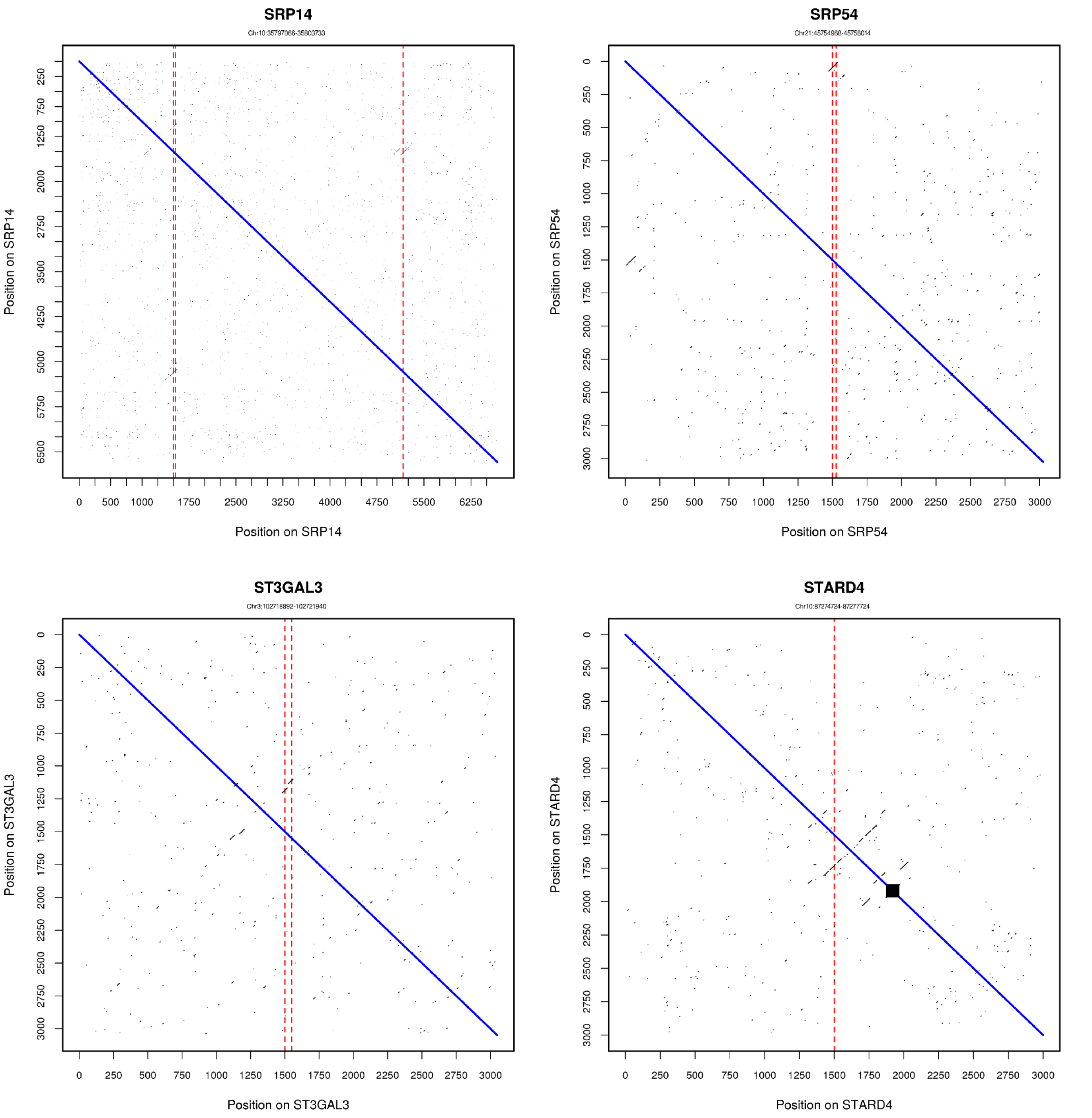

SRP14
Chr10:35797066-35803733
Position on SRP14
0 500 1000 1750 2500 3250 4000 4750 5500 6250
250 750 1250 2000 2750 3500 4250 5000 5750 6500
Position on SRP14
SRP54
Chr21:45754988-45758014
Position on SRP54
0 250 500 750 1000 1250 1500 1750 2000 2250 2500 2750 3000
Position on SRP54
ST3GAL3
Chr3:102718892-102721940
Position on ST3GAL3
0 250 500 750 1000 1250 1500 1750 2000 2250 2500 2750 3000
Position on ST3GAL3
STARD4
Chr10:87274724-87277724
Position on STARD4
0 250 500 750 1000 1250 1500 1750 2000 2250 2500 2750 3000
Position on STARD4

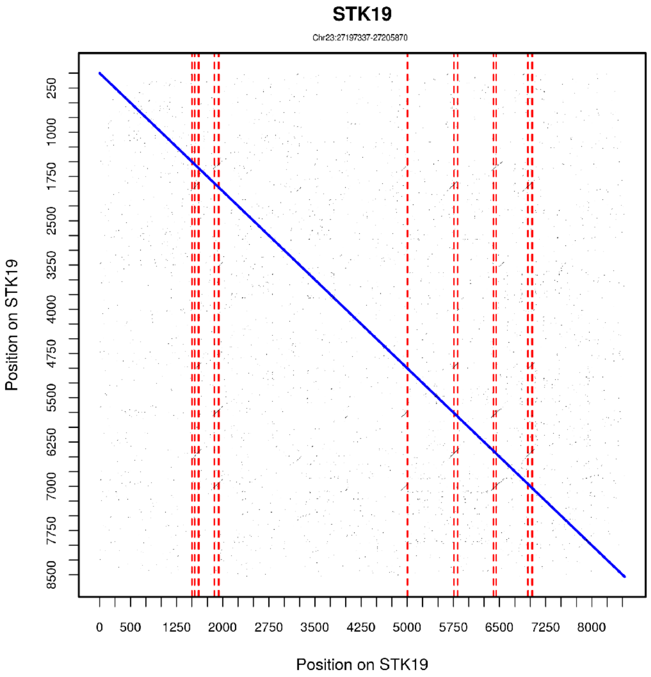
STK19
Chr23:27197337-27205870
Position on STK19
Position on STK19
0
500
1250
2000
2750
3500
4250
5000
5750
6500
7250
8000
250
1000
1750
2500
3250
4000
4750
5500
6250
7000
7750
8500

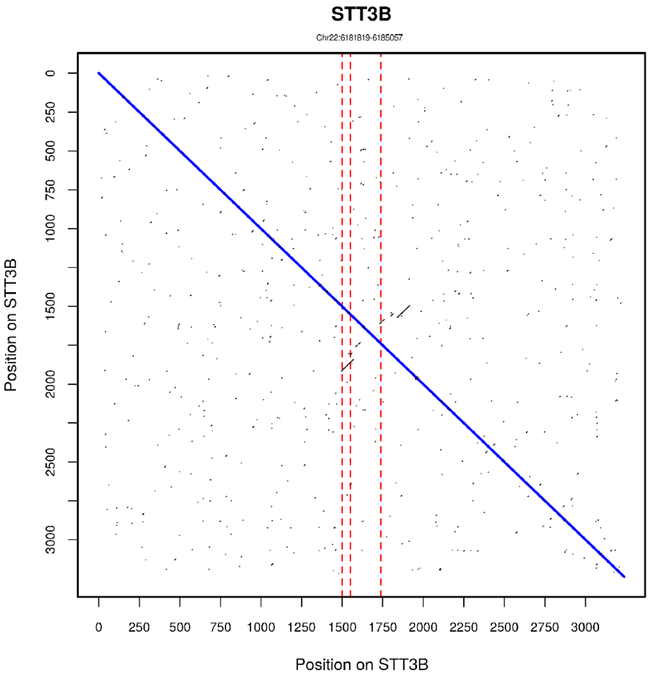
STT3B
Chr22:6181819-6185057
Position on STT3B
Position on STT3B
0
250
500
750
1000
1250
1500
1750
2000
2250
2500
2750
3000

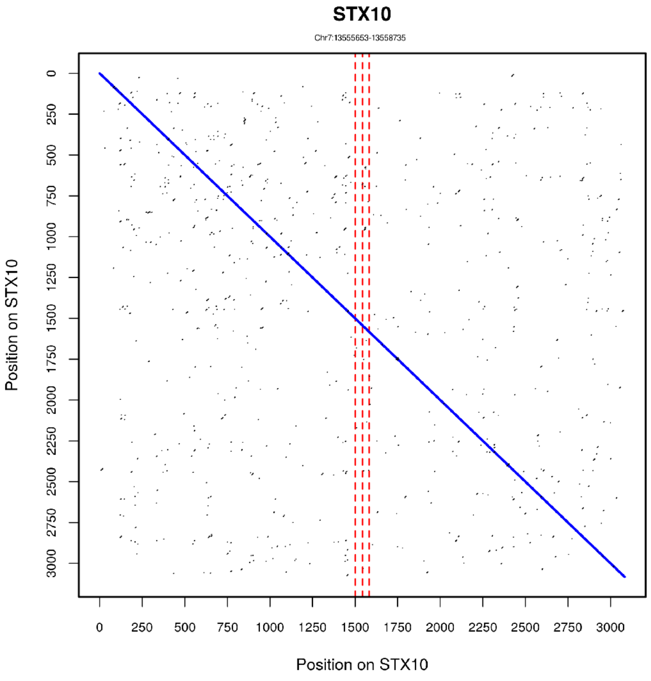
STX10
Chr7:13555653-13558735
Position on STX10
Position on STX10
0
250
500
750
1000
1250
1500
1750
2000
2250
2500
2750
3000

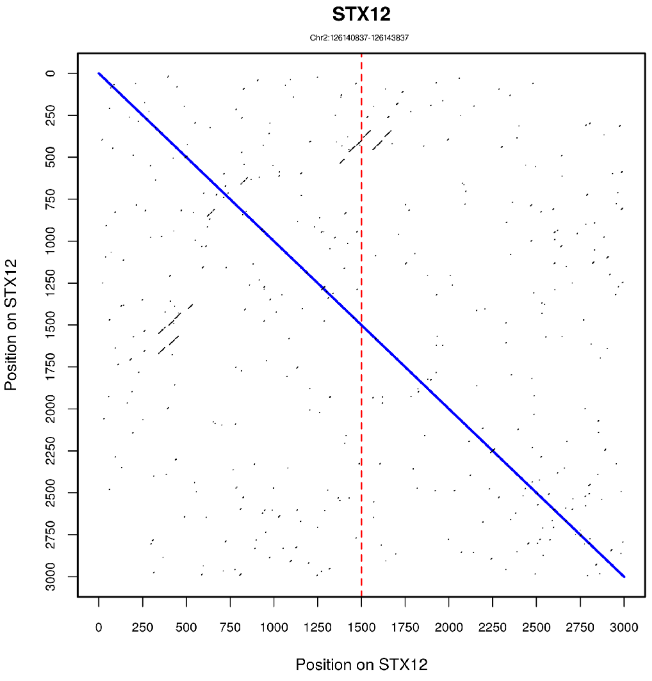
STX12
Chr2:126140837-126143837
Position on STX12
Position on STX12
0
250
500
750
1000
1250
1500
1750
2000
2250
2500
2750
3000

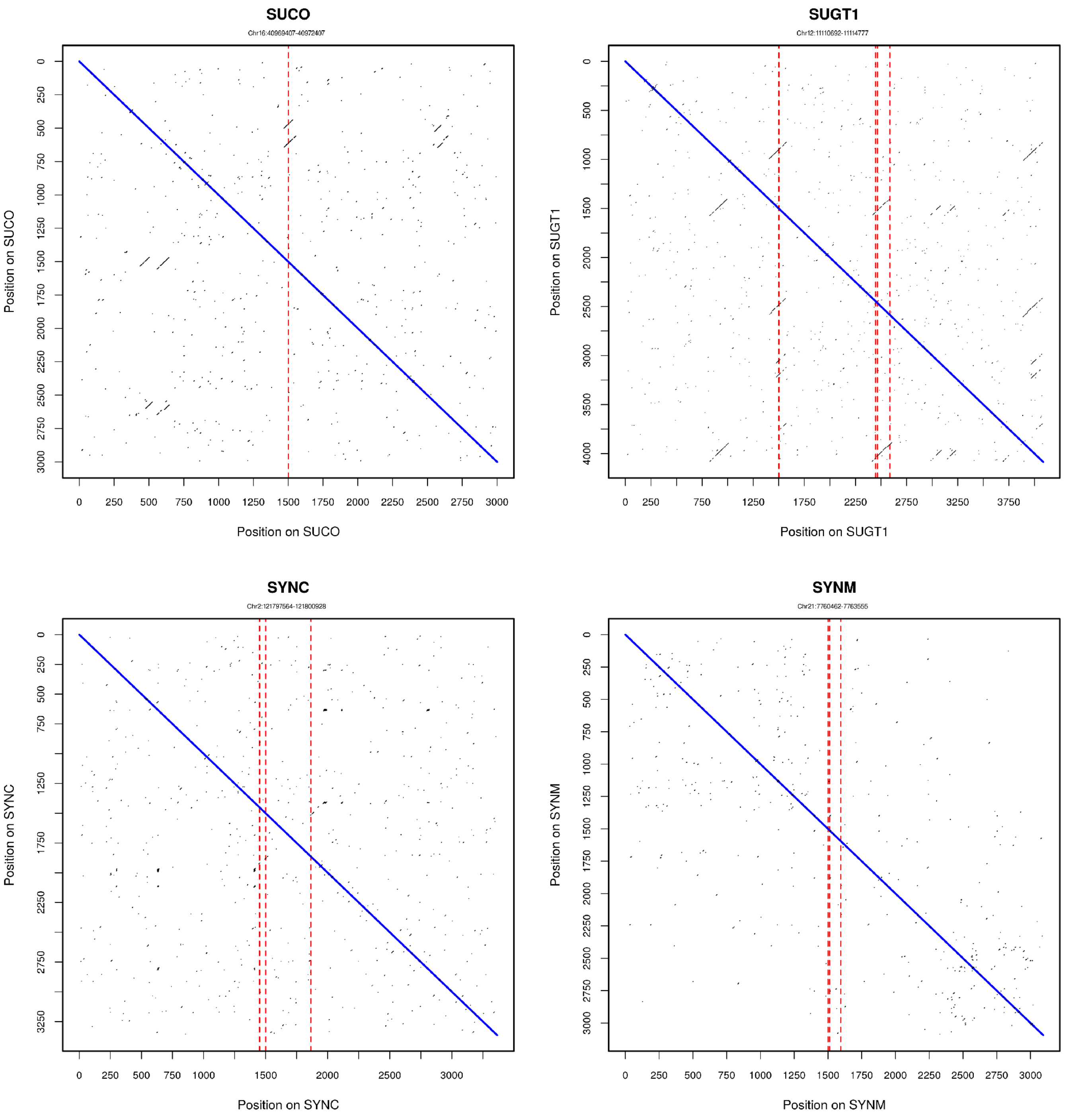

SUCO
Chr16:40969407-40972407
Position on SUCO
Position on SUCO
0
250
500
750
1000
1250
1500
1750
2000
2250
2500
2750
3000
SUGT1
Chr12:11110692-11114777
Position on SUGT1
Position on SUGT1
0
250
750
1250
1750
2250
2750
3250
3750
500
1000
1500
2000
2500
3000
3500
4000
SYNC
Chr2:121797564-121800928
Position on SYNC
Position on SYNC
0
250
500
750
1000
1250
1500
1750
2000
2250
2500
2750
3000
3250
SYNM
Chr21:7760462-7763555
Position on SYNM
Position on SYNM
0
250
500
750
1000
1250
1500
1750
2000
2250
2500
2750
3000

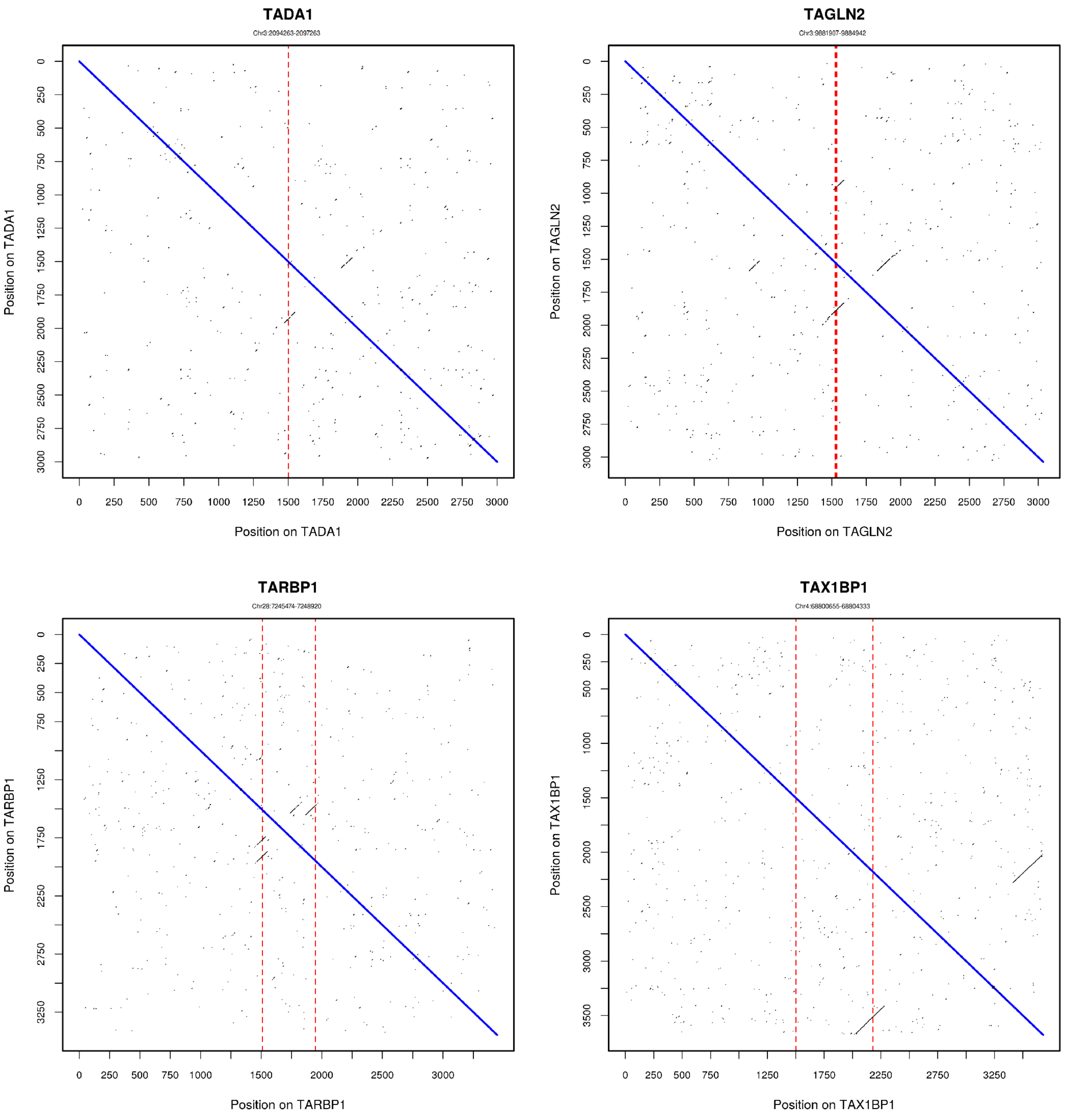
TADA1
Chr3:2094263-2097263
Position on TADA1
Position on TADA1
TAGLN2
Chr3:9881907-9884942
Position on TAGLN2
Position on TAGLN2
TARBP1
Chr28:7245474-7248920
Position on TARBP1
Position on TARBP1
TAX1BP1
Chr4:68800655-68804333
Position on TAX1BP1
Position on TAX1BP1

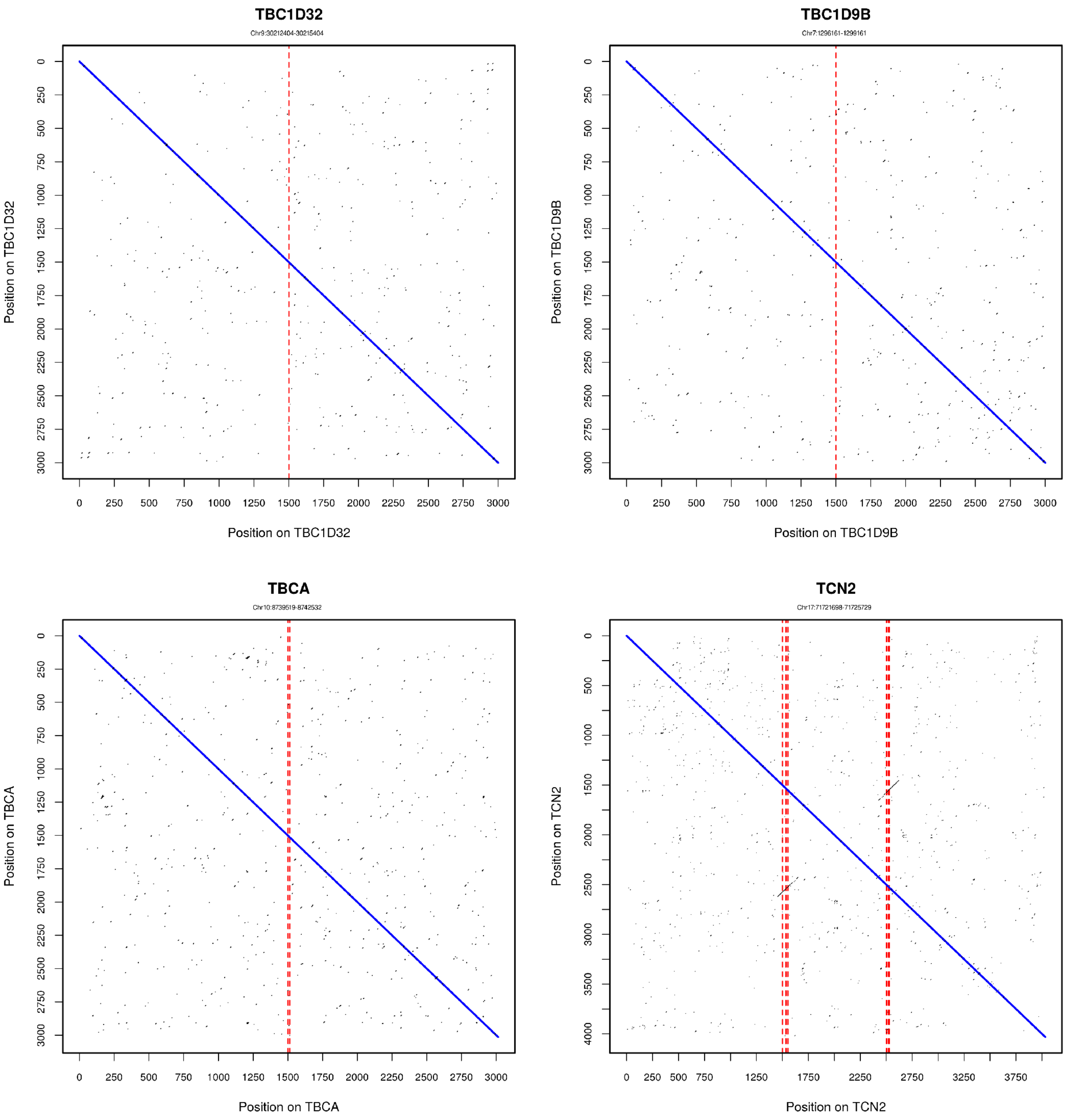

TBC1D32
Chr9:30212404-30215404
Position on TBC1D32
Position on TBC1D32
TBC1D9B
Chr7:1296161-1299161
Position on TBC1D9B
Position on TBC1D9B
TBCA
Chr10:8739519-8742532
Position on TBCA
Position on TBCA
TCN2
Chr17:71721698-71725729
Position on TCN2
Position on TCN2

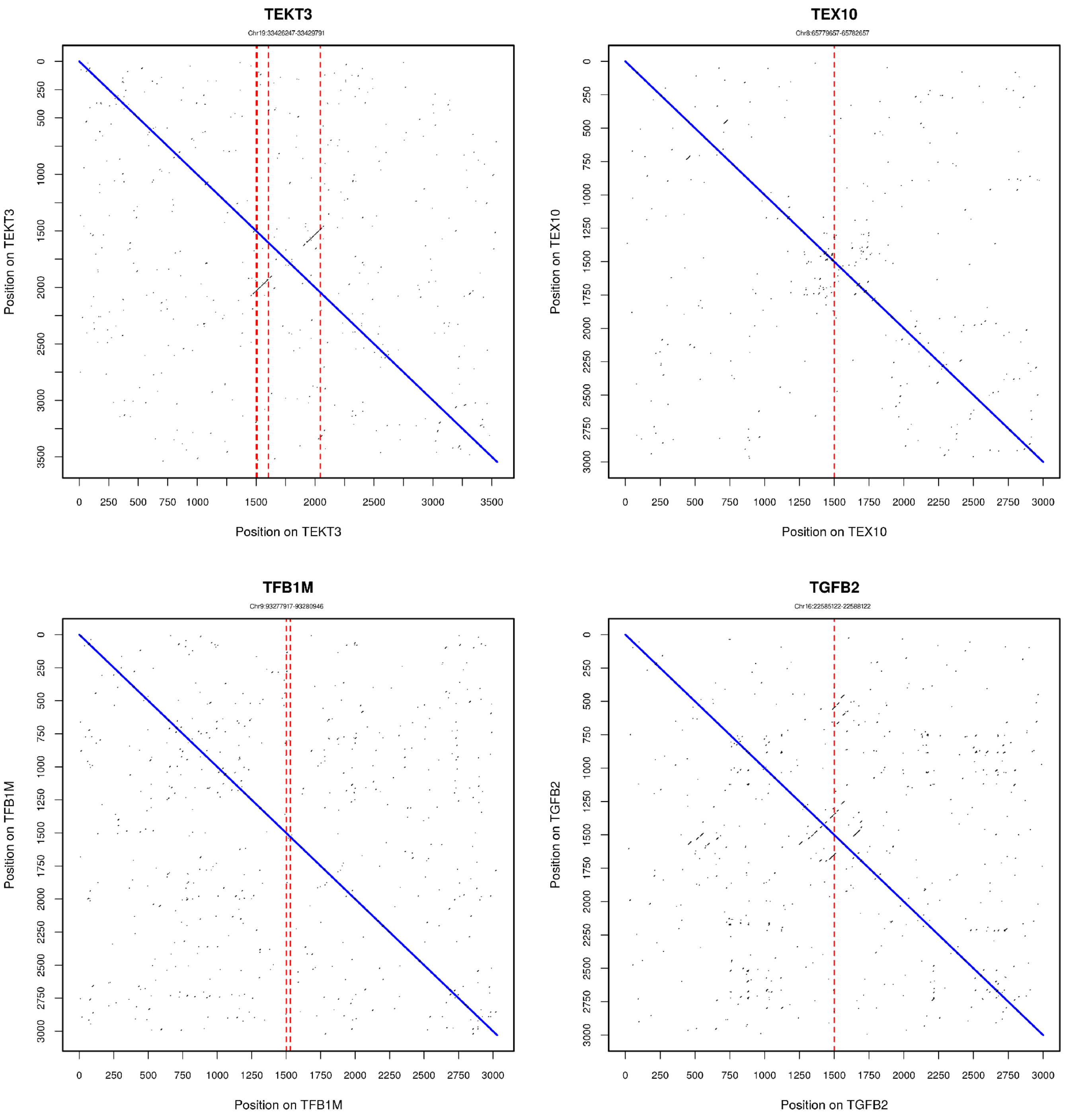

TEKT3
Chr19:33426247-33429791
Position on TEKT3
Position on TEKT3
TEX10
Chr8:65779657-65782657
Position on TEX10
Position on TEX10
TFB1M
Chr9:93277917-93280946
Position on TFB1M
Position on TFB1M
TGFB2
Chr16:22585122-22588122
Position on TGFB2
Position on TGFB2

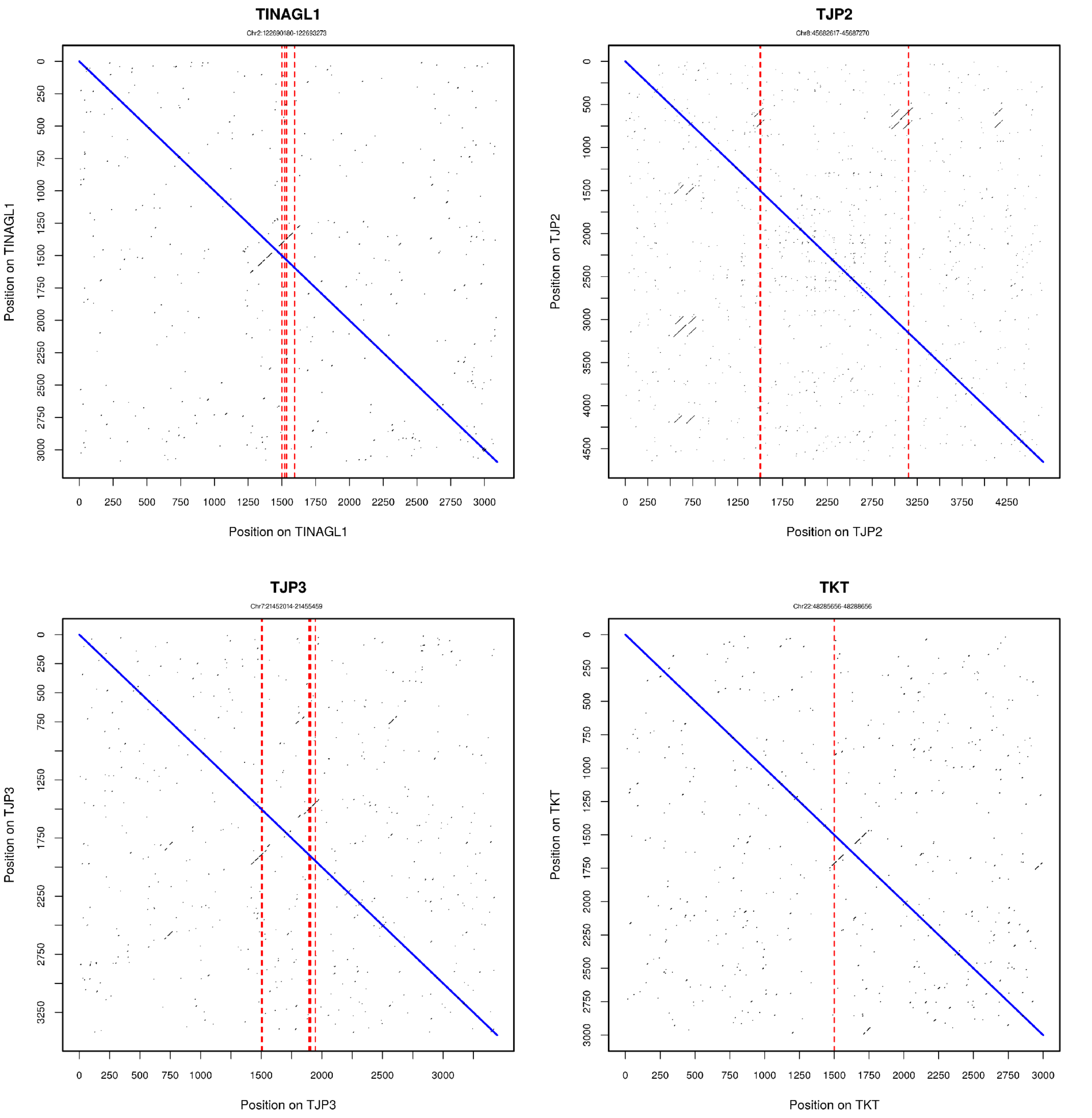
TINAGL1
Chr2:122690180-122693273
Position on TINAGL1
Position on TINAGL1
TJP2
Chr8:45682617-45687270
Position on TJP2
Position on TJP2
TJP3
Chr7:21452014-21455459
Position on TJP3
Position on TJP3
TKT
Chr22:48285656-48288656
Position on TKT
Position on TKT

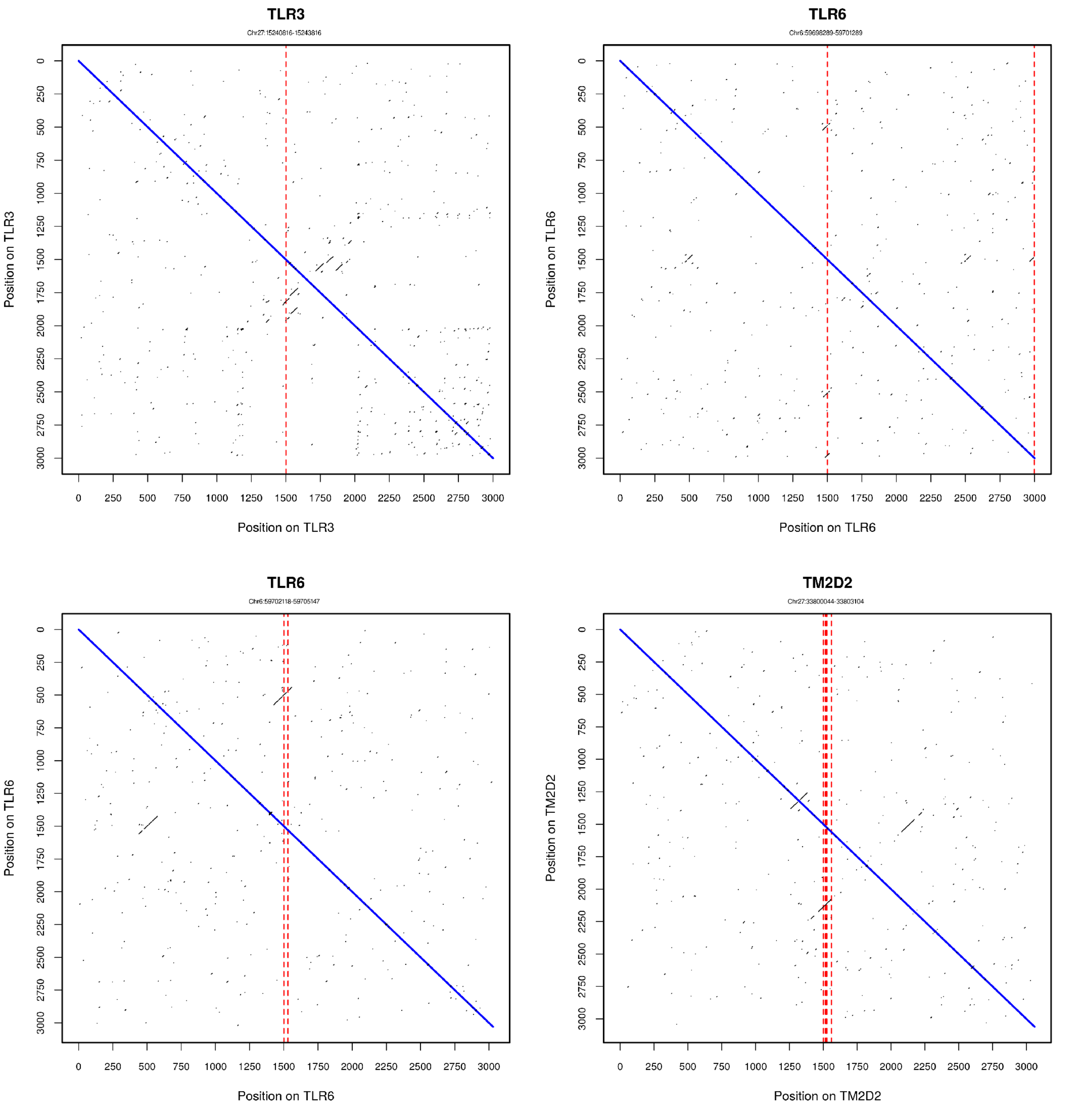
TLR3
Chr27:15240816-15243816
Position on TLR3
Position on TLR3
TLR6
Chr6:59698289-59701289
Position on TLR6
Position on TLR6
TLR6
Chr6:59702118-59705147
Position on TLR6
Position on TLR6
TM2D2
Chr27:33800044-33803104
Position on TM2D2
Position on TM2D2

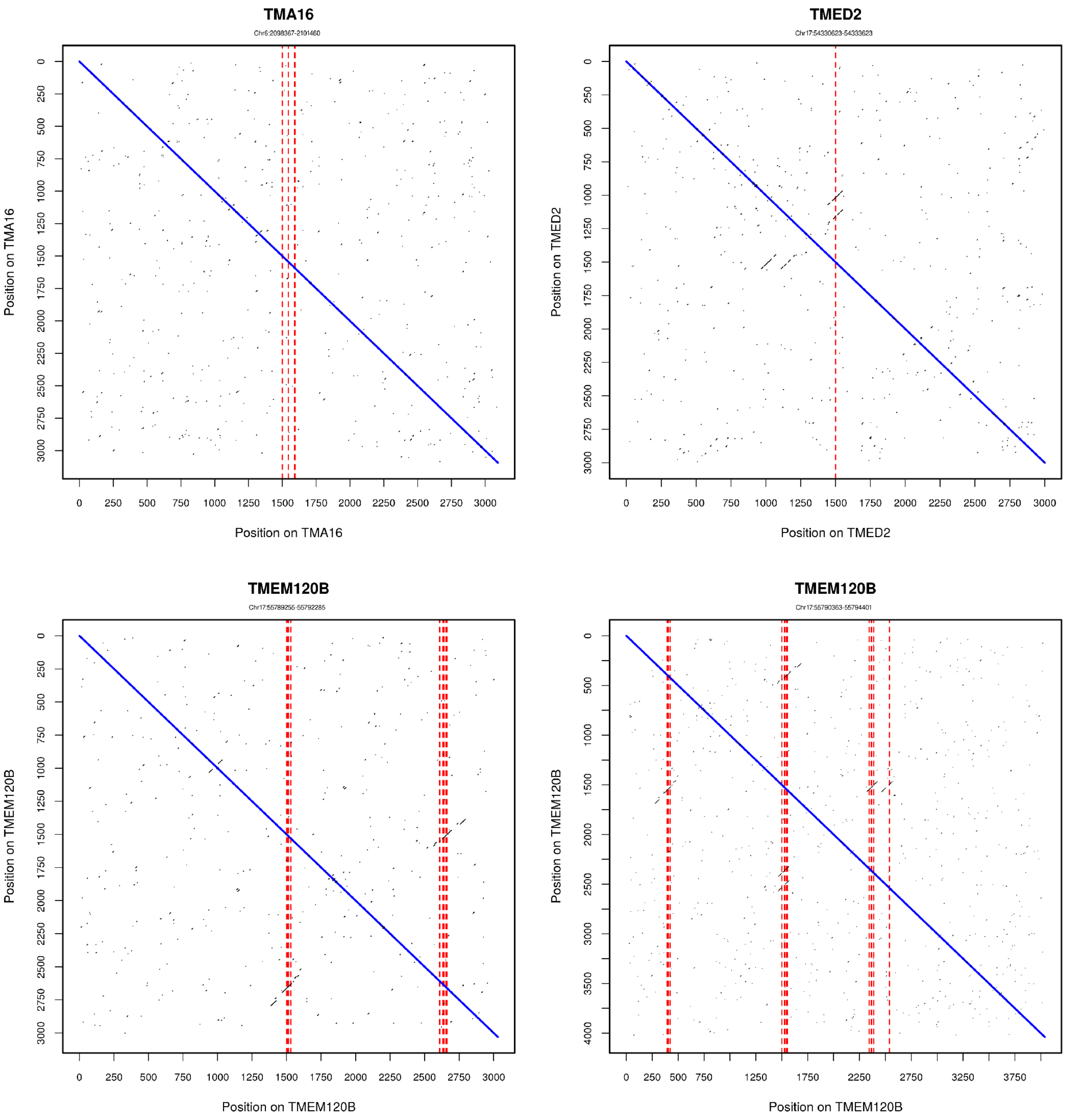

TMA16
Chr6:2098367-2101460
Position on TMA16
Position on TMA16
TMED2
Chr17:54330623-54333623
Position on TMED2
Position on TMED2
TMEM120B
Chr17:55789255-55792285
Position on TMEM120B
Position on TMEM120B
TMEM120B
Chr17:55790363-55794401
Position on TMEM120B
Position on TMEM120B

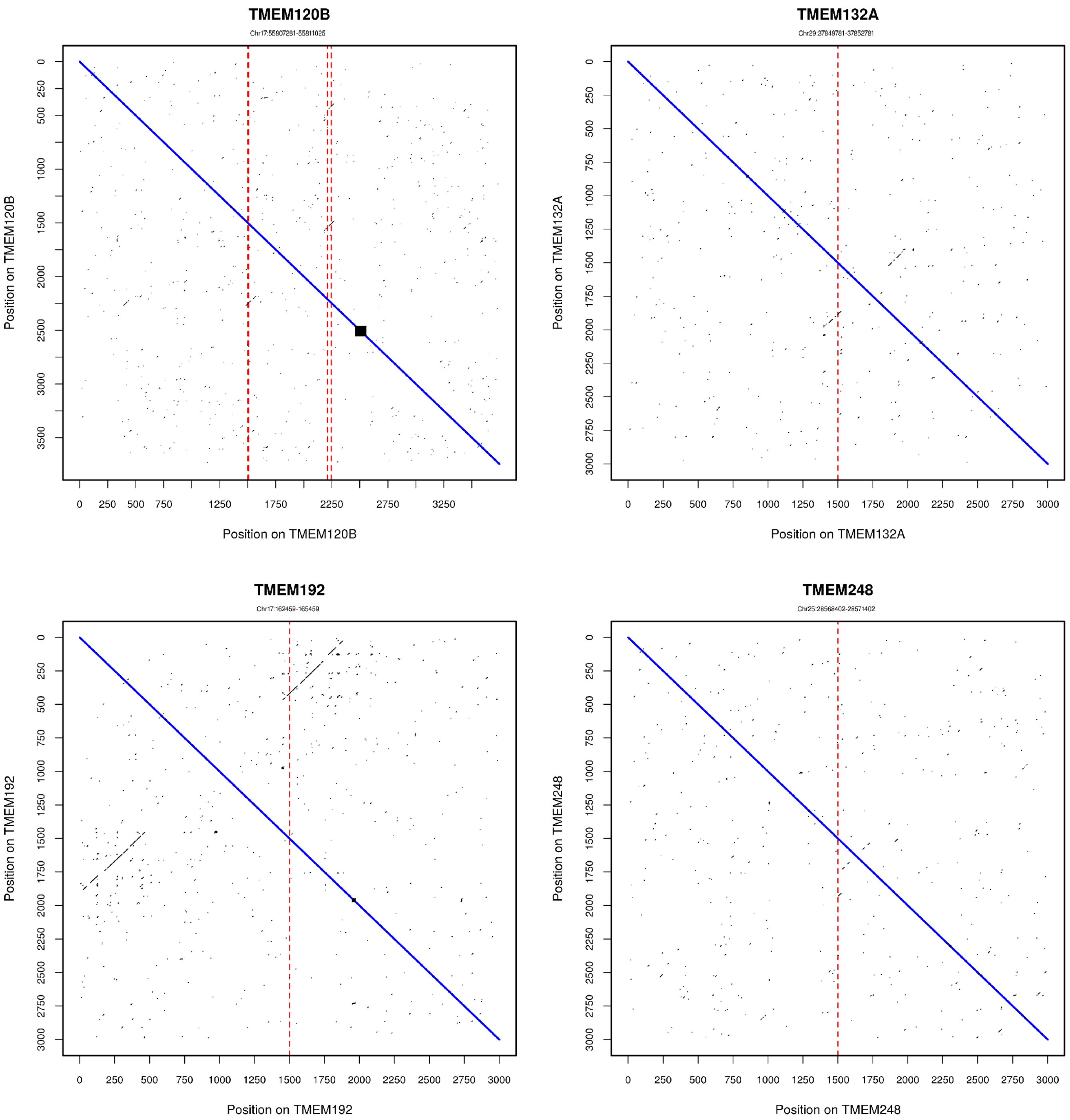

TMEM120B
Chr17:55807281-55811025
Position on TMEM120B
Position on TMEM120B
TMEM132A
Chr29:37849781-37852781
Position on TMEM132A
Position on TMEM132A
TMEM192
Chr17:162459-165459
Position on TMEM192
Position on TMEM192
TMEM248
Chr25:28568402-28571402
Position on TMEM248
Position on TMEM248

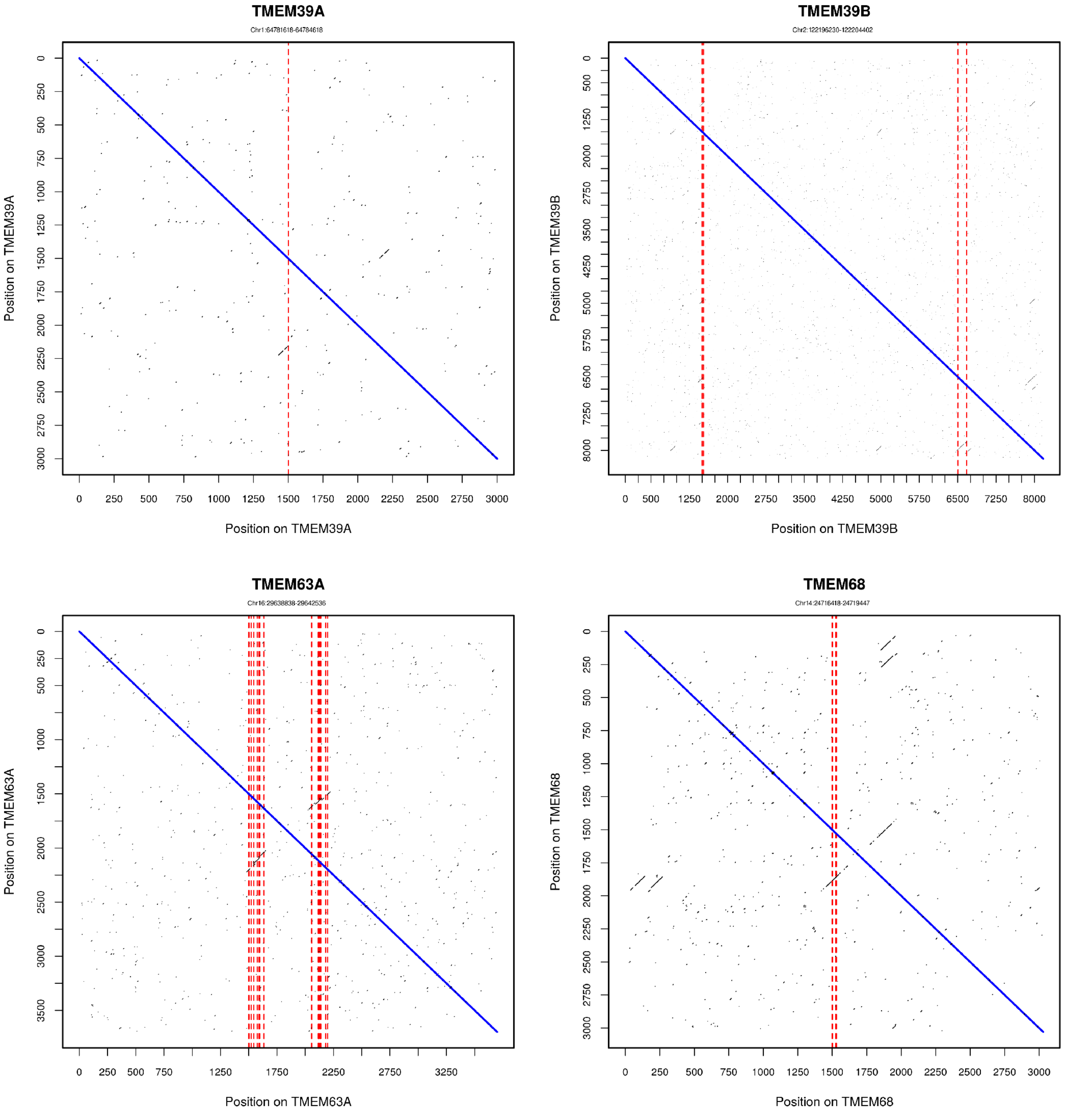

TMEM39A
Chr1:64781618-64784618
Position on TMEM39A
Position on TMEM39A
TMEM39B
Chr2:122196230-122204402
Position on TMEM39B
Position on TMEM39B
TMEM63A
Chr16:29638838-29642536
Position on TMEM63A
Position on TMEM63A
TMEM68
Chr14:24716418-24719447
Position on TMEM68
Position on TMEM68

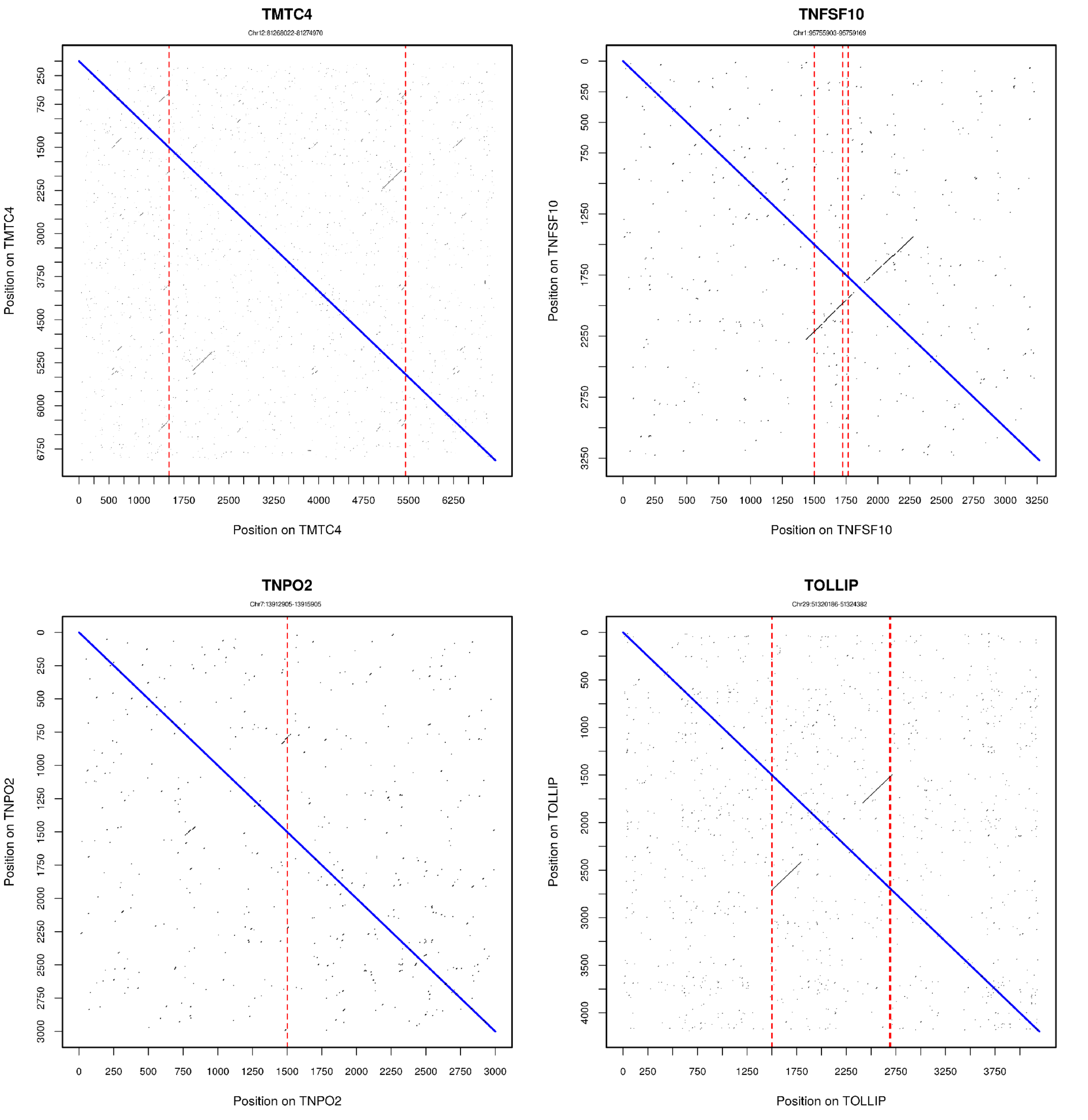
TMTC4
Chr12:81268022-81274970
Position on TMTC4
Position on TMTC4
TNFSF10
Chr1:95755903-95759169
Position on TNFSF10
Position on TNFSF10
TNPO2
Chr7:13912905-13915905
Position on TNPO2
Position on TNPO2
TOLLIP
Chr29:51320186-51324382
Position on TOLLIP
Position on TOLLIP

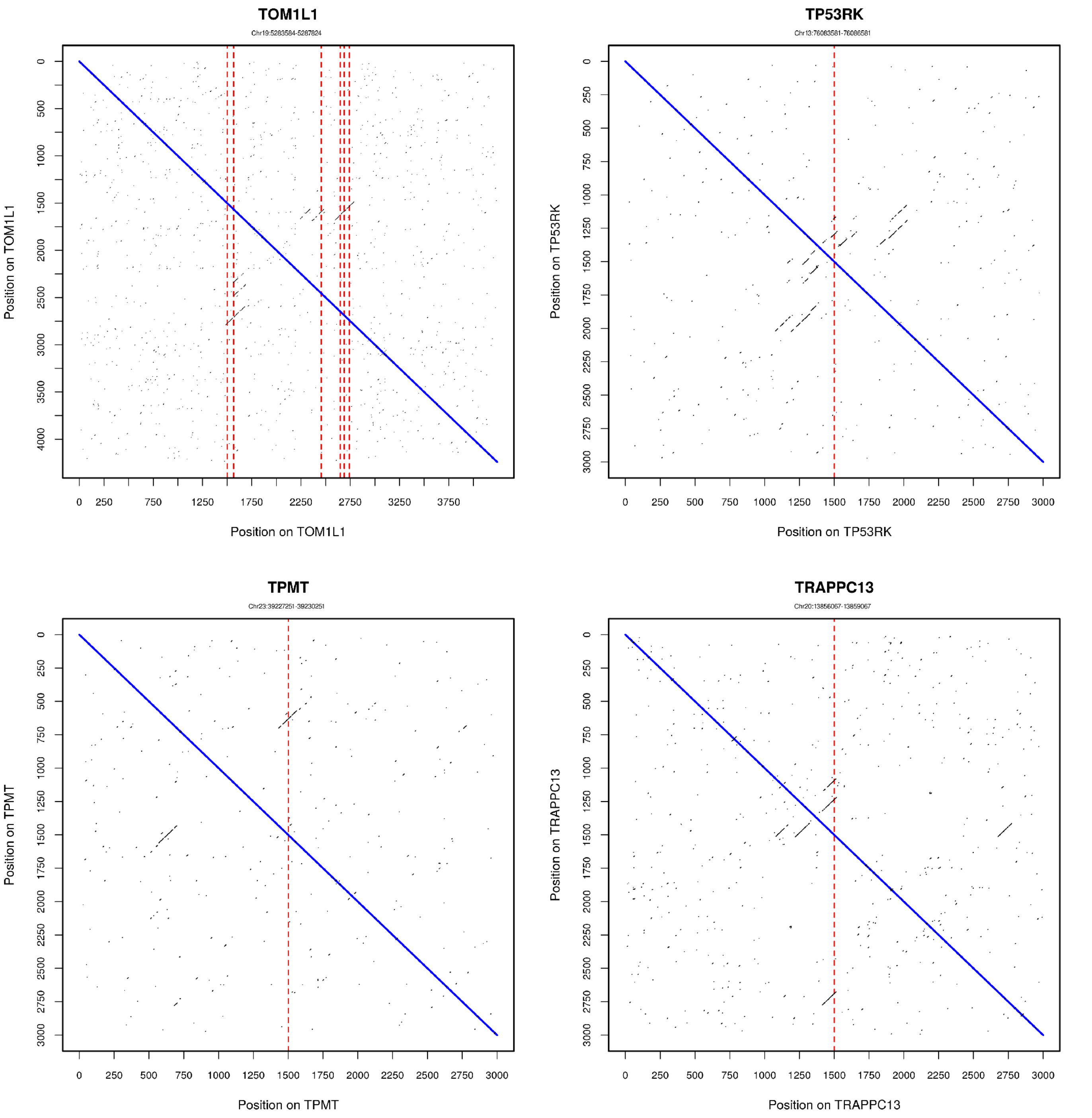

TOM1L1
Chr19:5283584-5287824
Position on TOM1L1
Position on TOM1L1
TP53RK
Chr13:76083581-76086581
Position on TP53RK
Position on TP53RK
TPMT
Chr23:39227251-39230251
Position on TPMT
Position on TPMT
TRAPPC13
Chr20:13856067-13859067
Position on TRAPPC13
Position on TRAPPC13

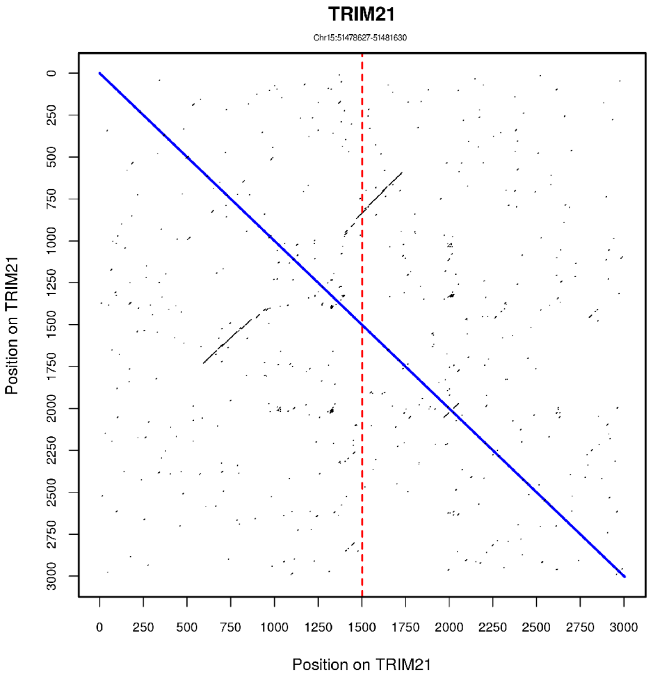
TRIM21
Chr15:51478627-51481630
Position on TRIM21
Position on TRIM21

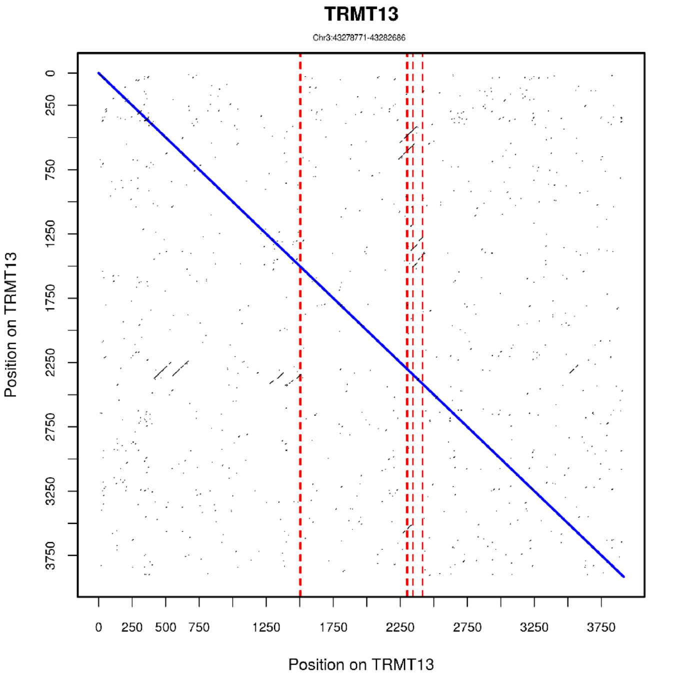
TRMT13
Chr3:43278771-43282686
Position on TRMT13
Position on TRMT13

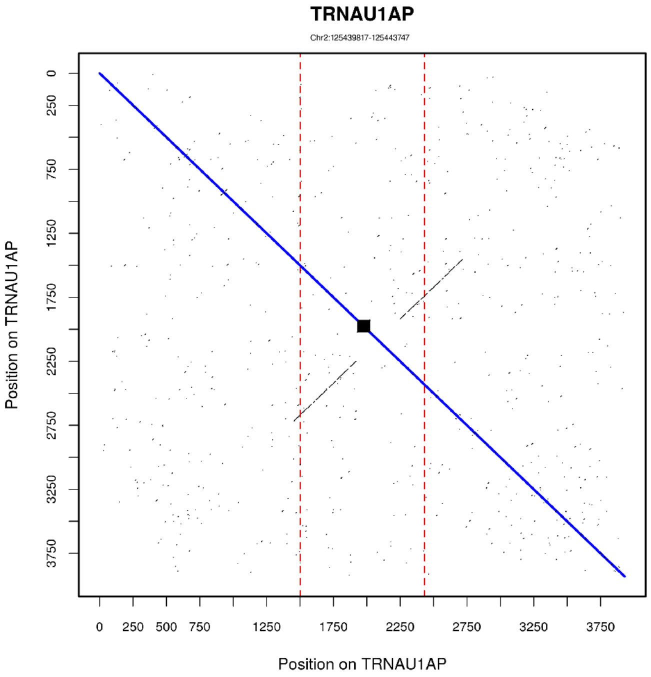
TRNAU1AP
Chr2:125439817-125443747
Position on TRNAU1AP
Position on TRNAU1AP

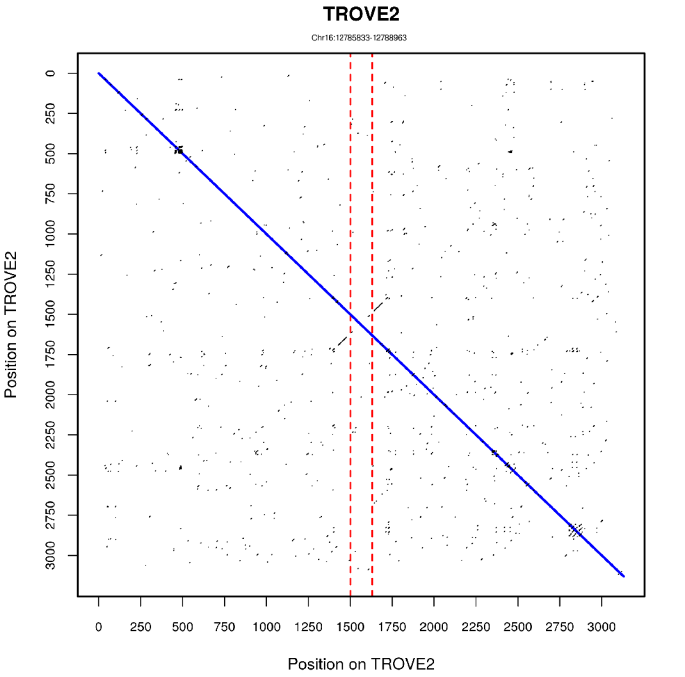
TROVE2
Chr16:12785833-12788963
Position on TROVE2
Position on TROVE2

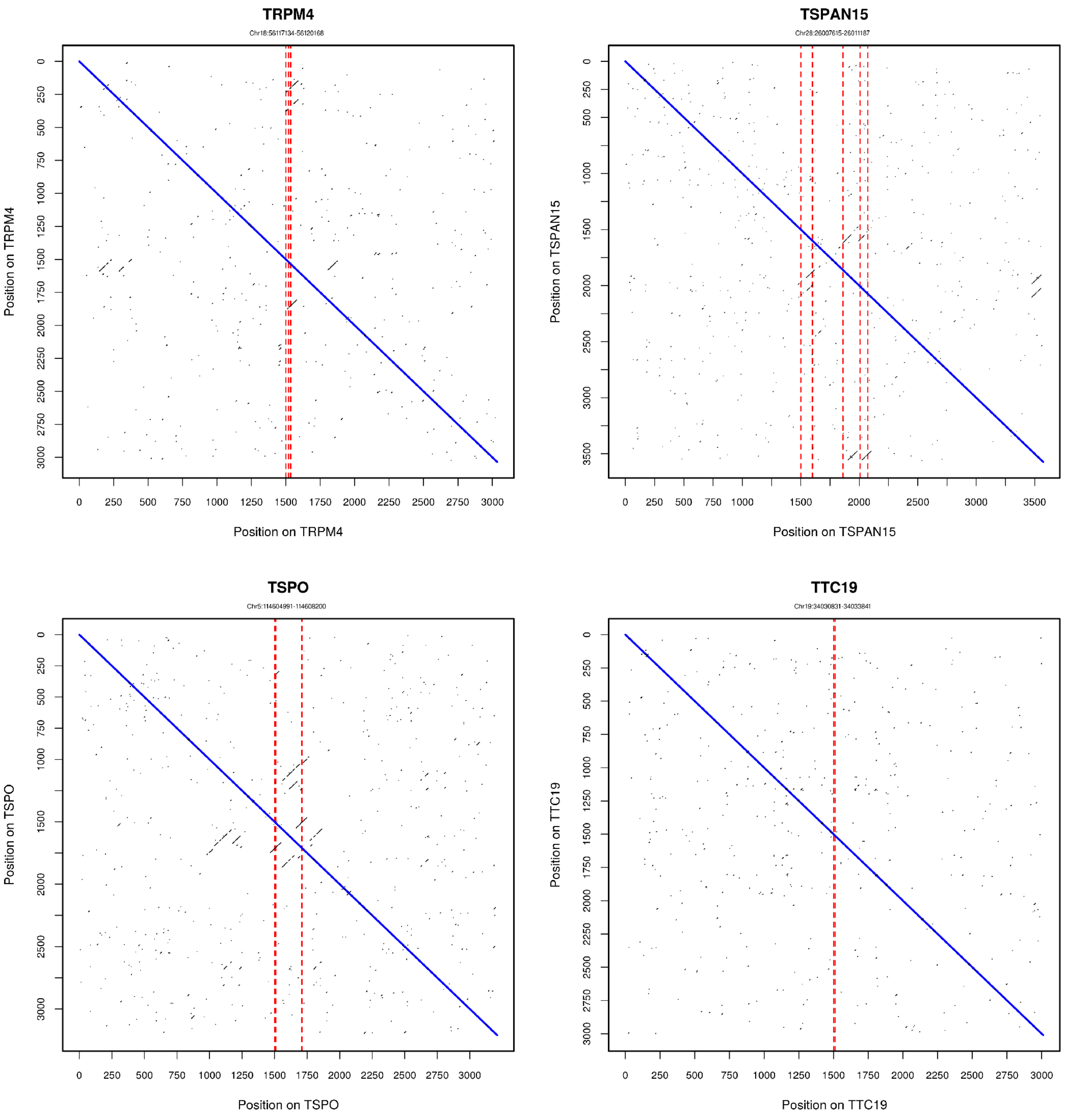

TRPM4
Chr18:56117134-56120168
Position on TRPM4
Position on TRPM4
TSPAN15
Chr28:26007615-26011187
Position on TSPAN15
Position on TSPAN15
TSPO
Chr5:114604991-114608200
Position on TSPO
Position on TSPO
TTC19
Chr19:34030831-34033841
Position on TTC19
Position on TTC19

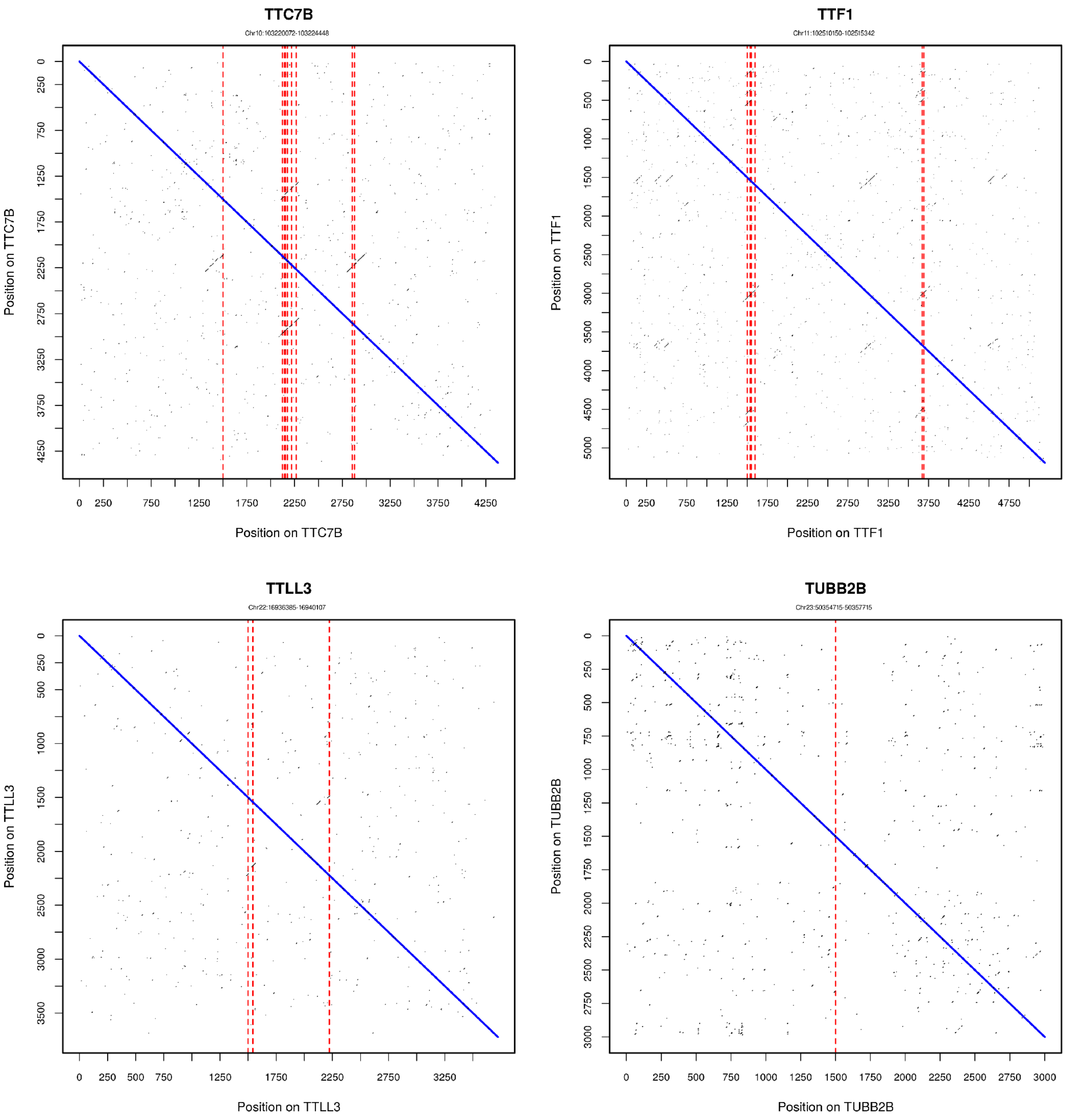

TTC7B
Chr10:103220072-103224448
Position on TTC7B
0 250 750 1250 1750 2250 2750 3250 3750 4250
Position on TTC7B
TTF1
Chr11:102510150-102515342
Position on TTF1
0 500 1000 1500 2000 2500 3000 3500 4000 4500 5000
0 250 750 1250 1750 2250 2750 3250 3750 4250 4750
Position on TTF1
TTLL3
Chr22:16936385-16940107
Position on TTLL3
0 250 500 1000 1500 2000 2500 3000 3500
0 250 500 750 1250 1750 2250 2750 3250
Position on TTLL3
TUBB2B
Chr23:50354715-50357715
Position on TUBB2B
0 250 500 750 1000 1250 1500 1750 2000 2250 2500 2750 3000
Position on TUBB2B

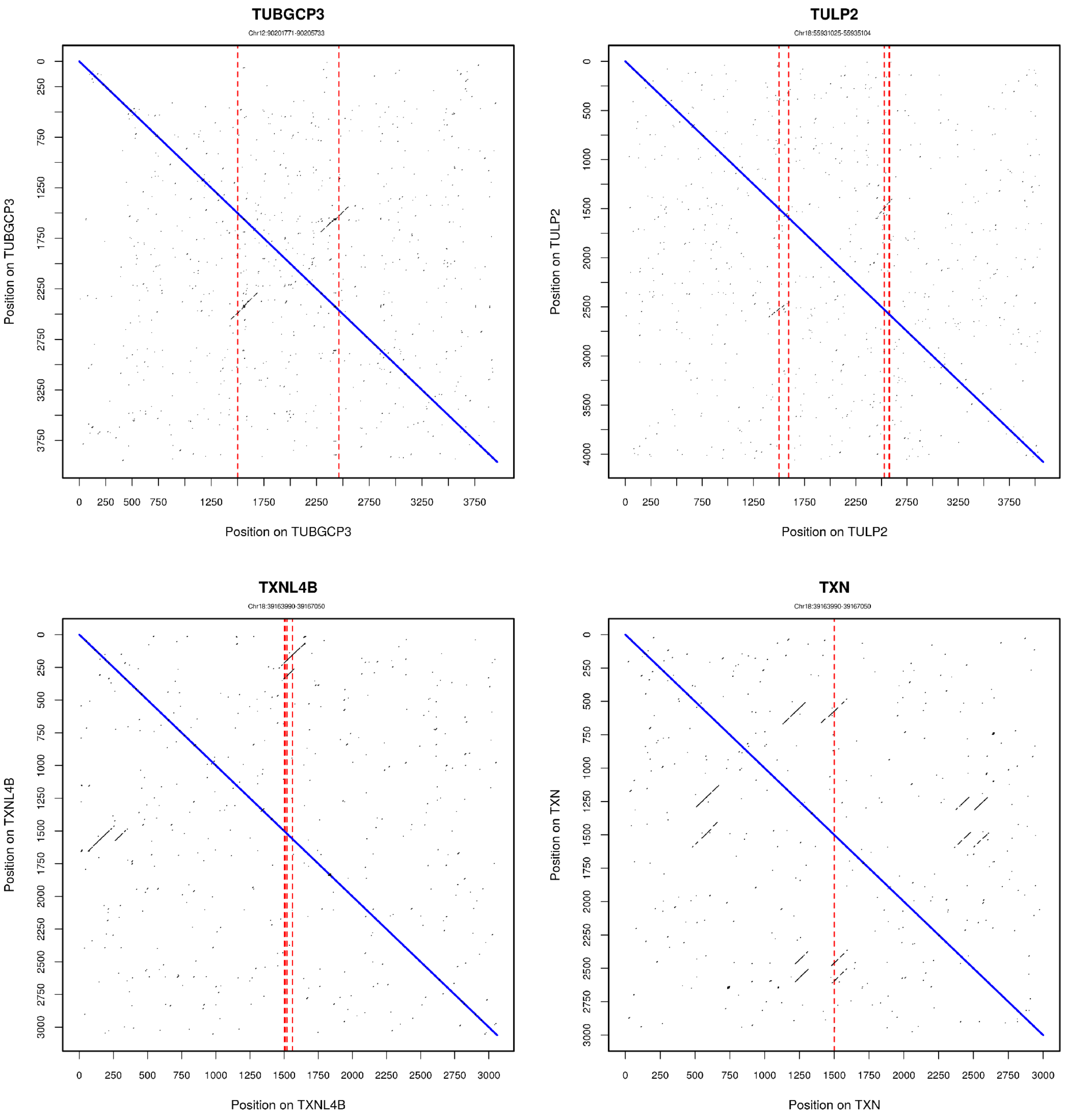

TUBGCP3
Chr12:90201771-90205733
Position on TUBGCP3
Position on TUBGCP3
TULP2
Chr18:55931025-55935104
Position on TULP2
Position on TULP2
TXNL4B
Chr18:39163990-39167050
Position on TXNL4B
Position on TXNL4B
TXN
Chr18:39163990-39167050
Position on TXN
Position on TXN

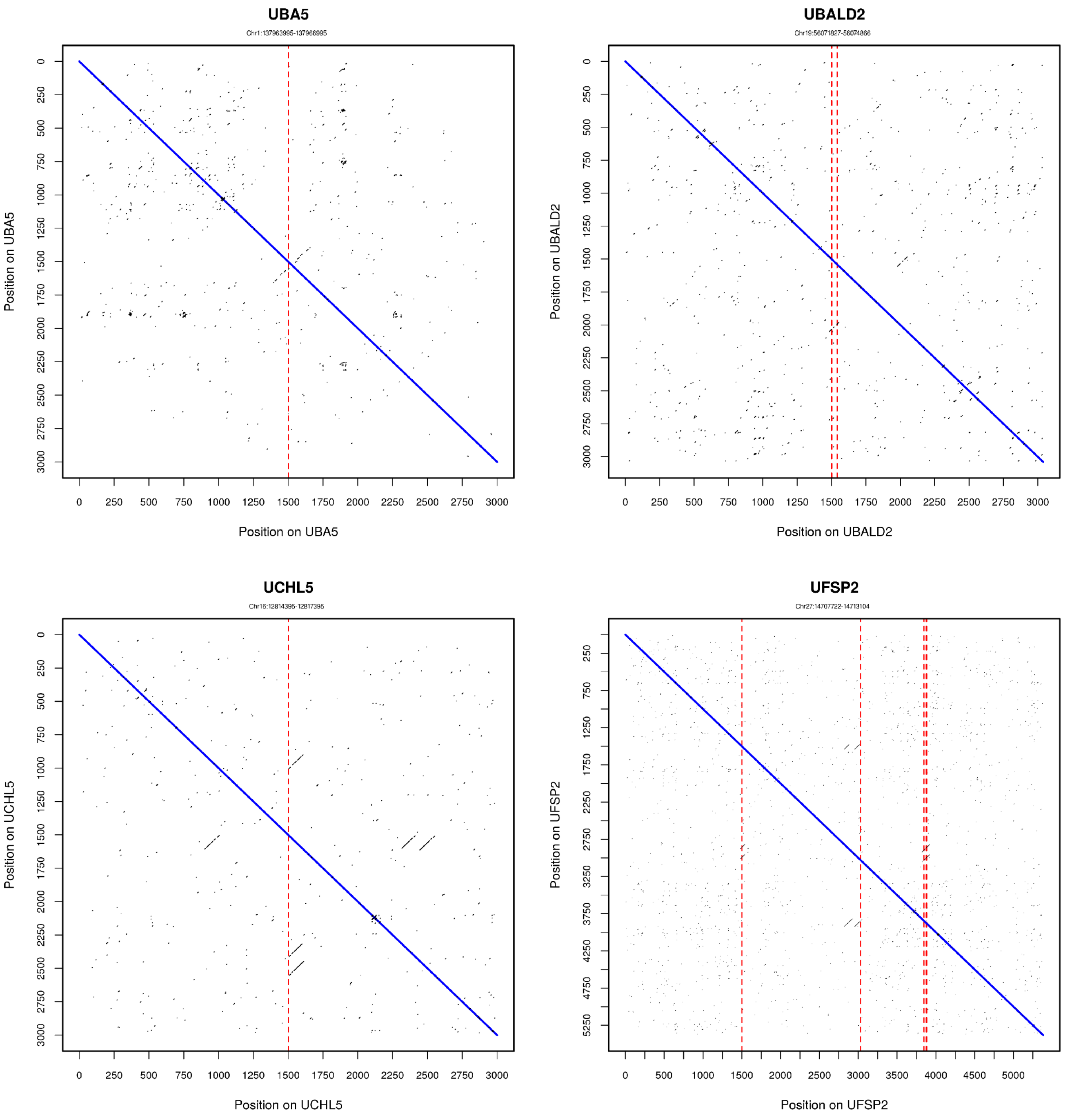
UBA5
Chr1:137963995-137966995
Position on UBA5
Position on UBA5
UBALD2
Chr19:56071827-56074866
Position on UBALD2
Position on UBALD2
UCHL5
Chr16:12814395-12817395
Position on UCHL5
Position on UCHL5
UFSP2
Chr27:14707722-14713104
Position on UFSP2
Position on UFSP2

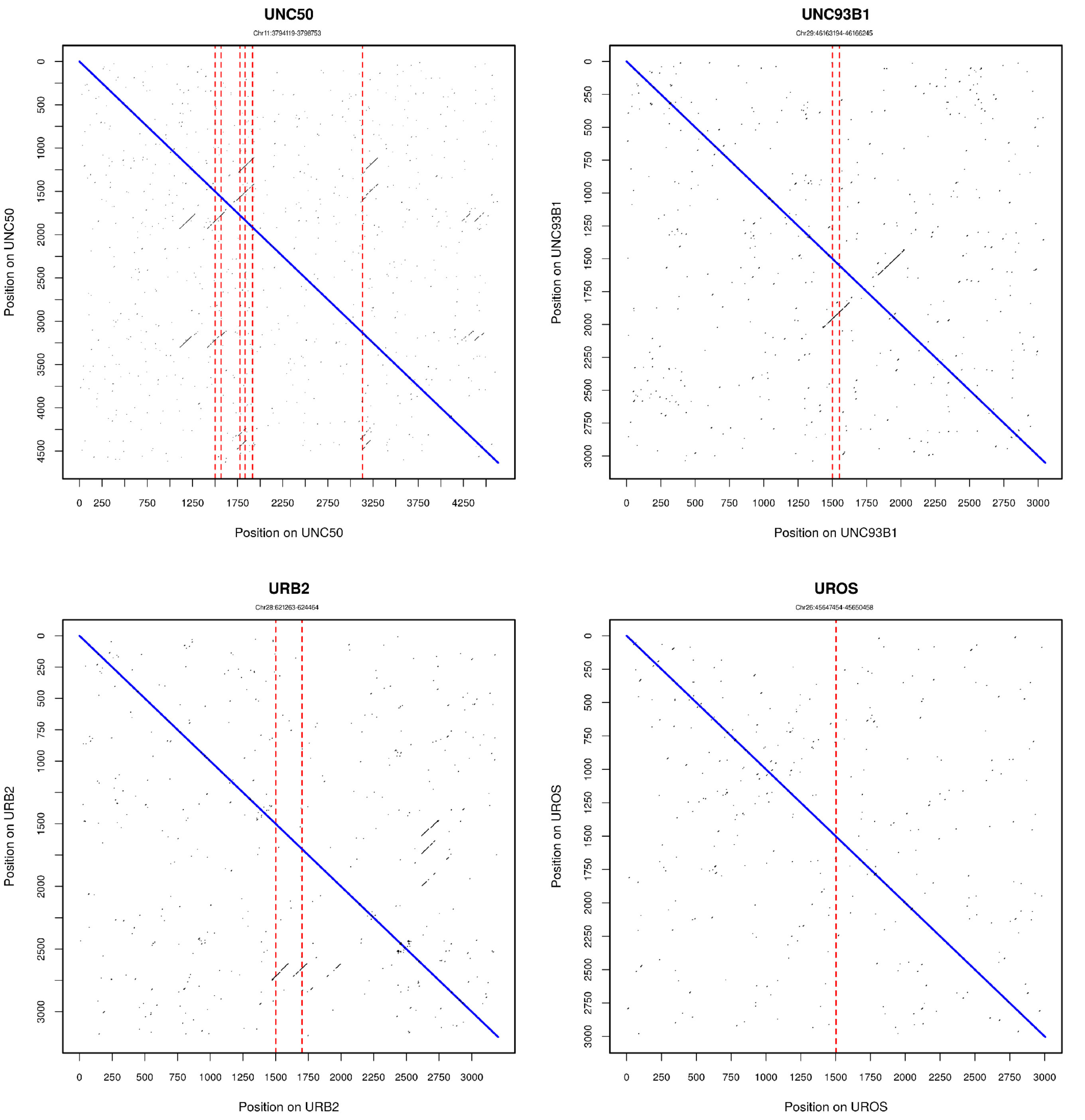
UNC50
Chr11:3794119-3798753
Position on UNC50
Position on UNC50
UNC93B1
Chr29:46163194-46166245
Position on UNC93B1
Position on UNC93B1
URB2
Chr28:621263-624464
Position on URB2
Position on URB2
UROS
Chr26:45647454-45650458
Position on UROS
Position on UROS

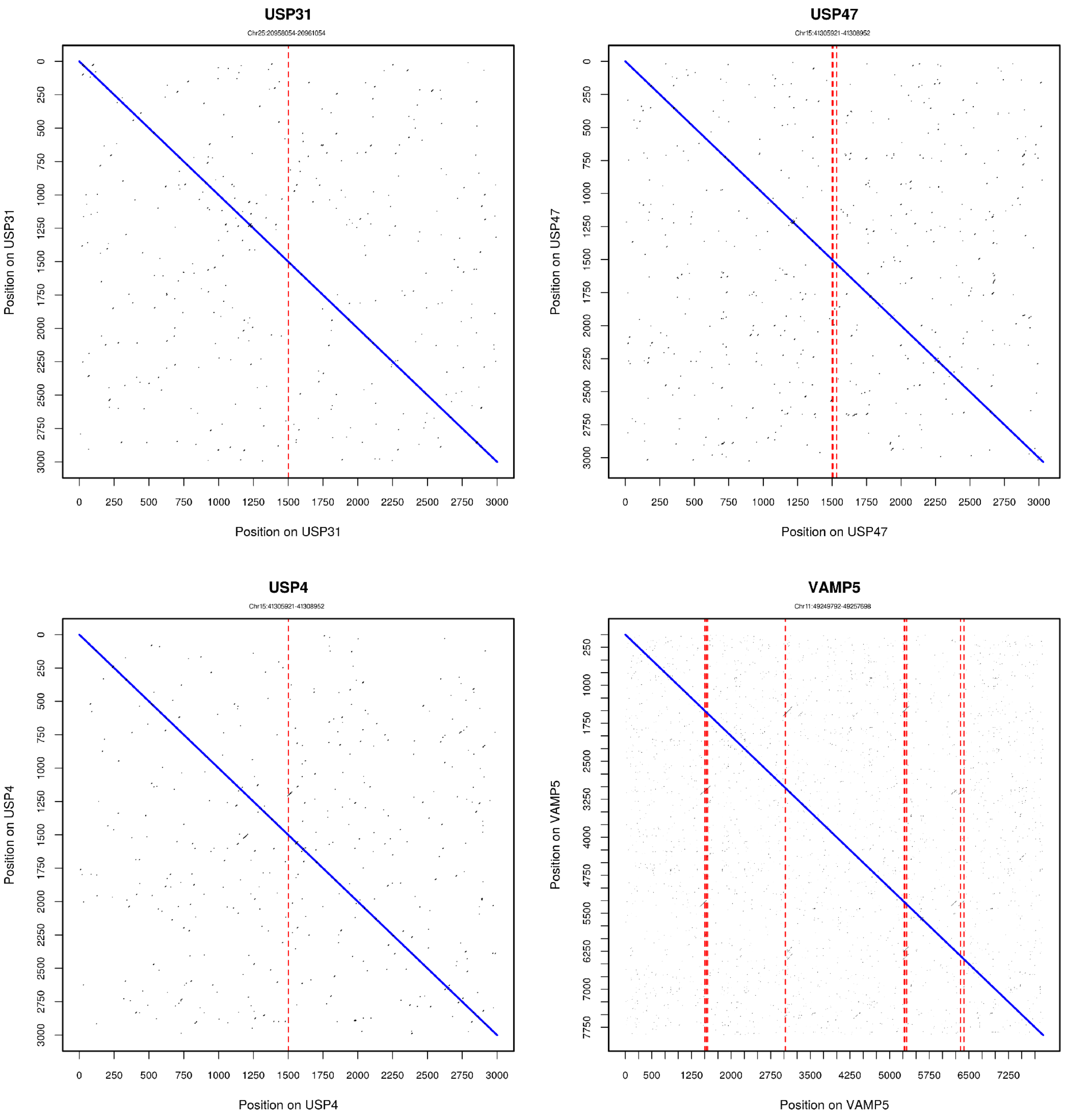
USP31
Chr25:20958054-20961054
Position on USP31
Position on USP31
USP47
Chr15:41305921-41308952
Position on USP47
Position on USP47
USP4
Chr15:41305921-41308952
Position on USP4
Position on USP4
VAMP5
Chr11:49249792-49257698
Position on VAMP5
Position on VAMP5

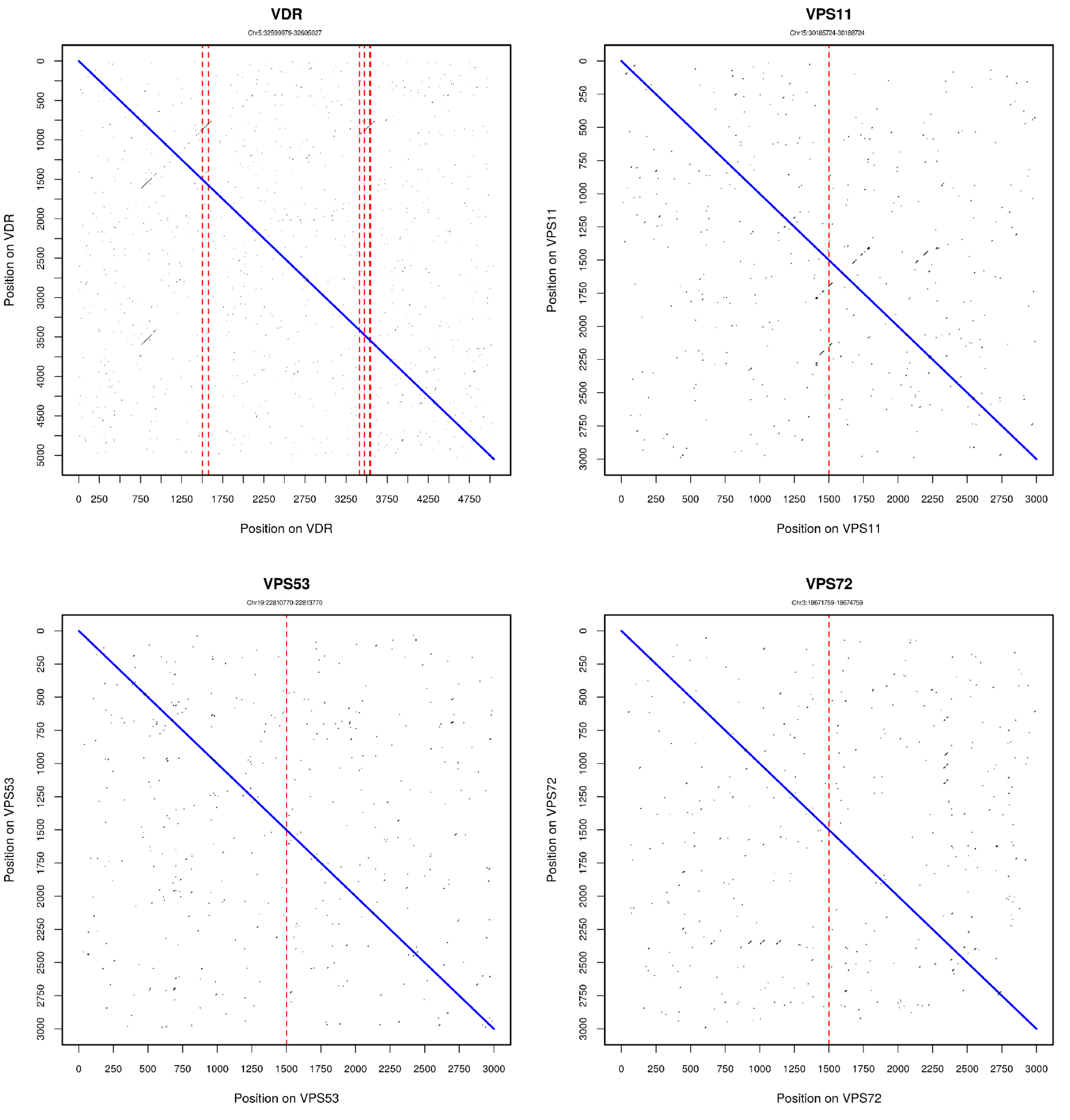
VDR
Chr5:32599979-32605027
Position on VDR
Position on VDR
VPS11
Chr15:30185724-30188724
Position on VPS11
Position on VPS11
VPS53
Chr19:22810770-22813770
Position on VPS53
Position on VPS53
VPS72
Chr3:19671759-19674759
Position on VPS72
Position on VPS72

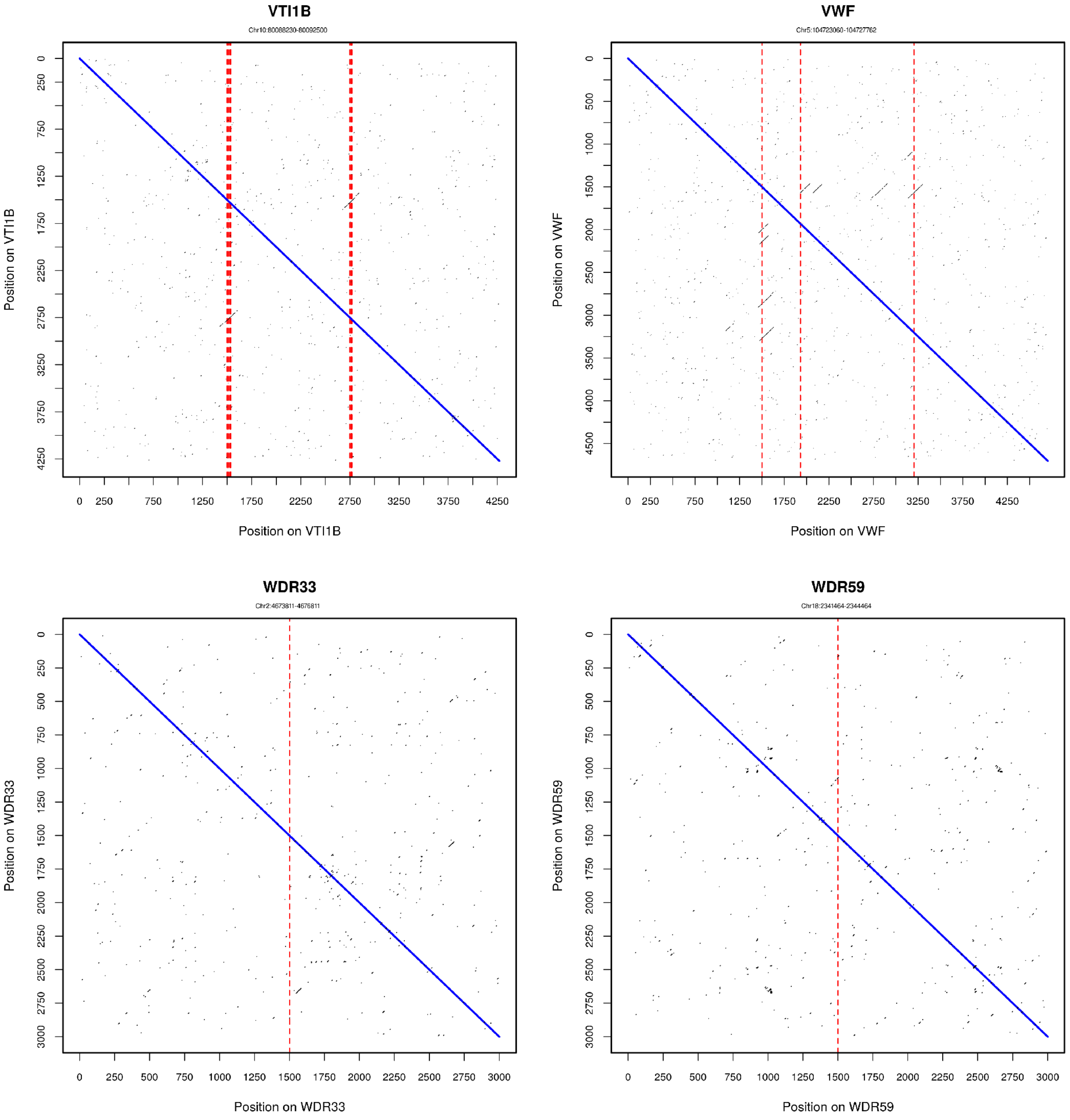

VTI1B
Chr10:80088230-80092500
Position on VTI1B
Position on VTI1B
VWF
Chr5:104723060-104727762
Position on VWF
Position on VWF
WDR33
Chr2:4673811-4676811
Position on WDR33
Position on WDR33
WDR59
Chr18:2341464-2344464
Position on WDR59
Position on WDR59

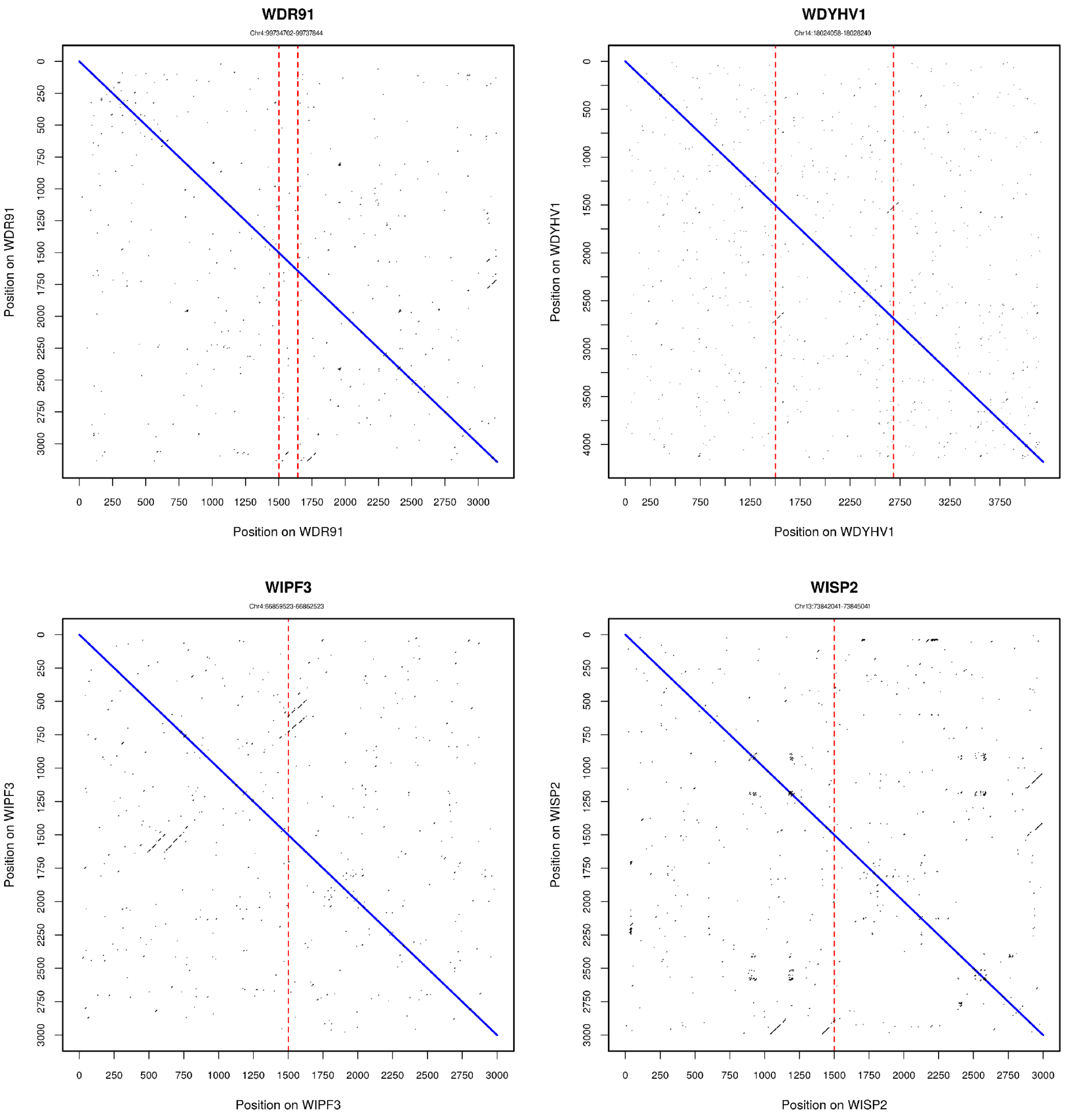
WDR91
Chr4:99734702-99737844
Position on WDR91
Position on WDR91
WDYHV1
Chr14:18024058-18028240
Position on WDYHV1
Position on WDYHV1
WIPF3
Chr4:66859523-66862523
Position on WIPF3
Position on WIPF3
WISP2
Chr13:73842041-73845041
Position on WISP2
Position on WISP2

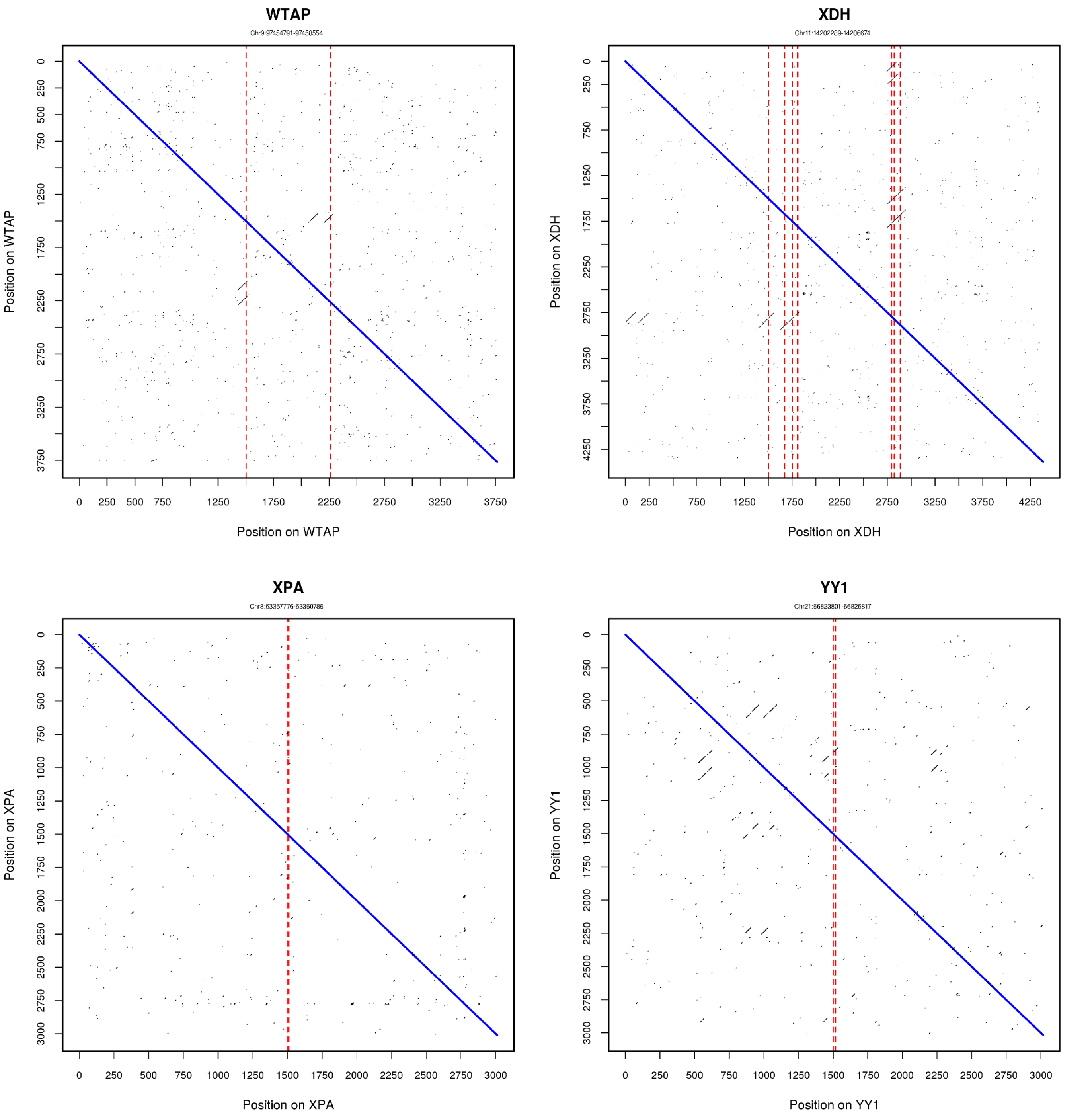
WTAP
Chr9:97454791-97458554
Position on WTAP
Position on WTAP
0
250
500
750
1250
1750
2250
2750
3250
3750
XDH
Chr11:14202289-14206674
Position on XDH
Position on XDH
0
250
750
1250
1750
2250
2750
3250
3750
4250
XPA
Chr8:63357776-63360786
Position on XPA
Position on XPA
0
250
500
750
1000
1250
1500
1750
2000
2250
2500
2750
3000
YY1
Chr21:66823801-66826817
Position on YY1
Position on YY1

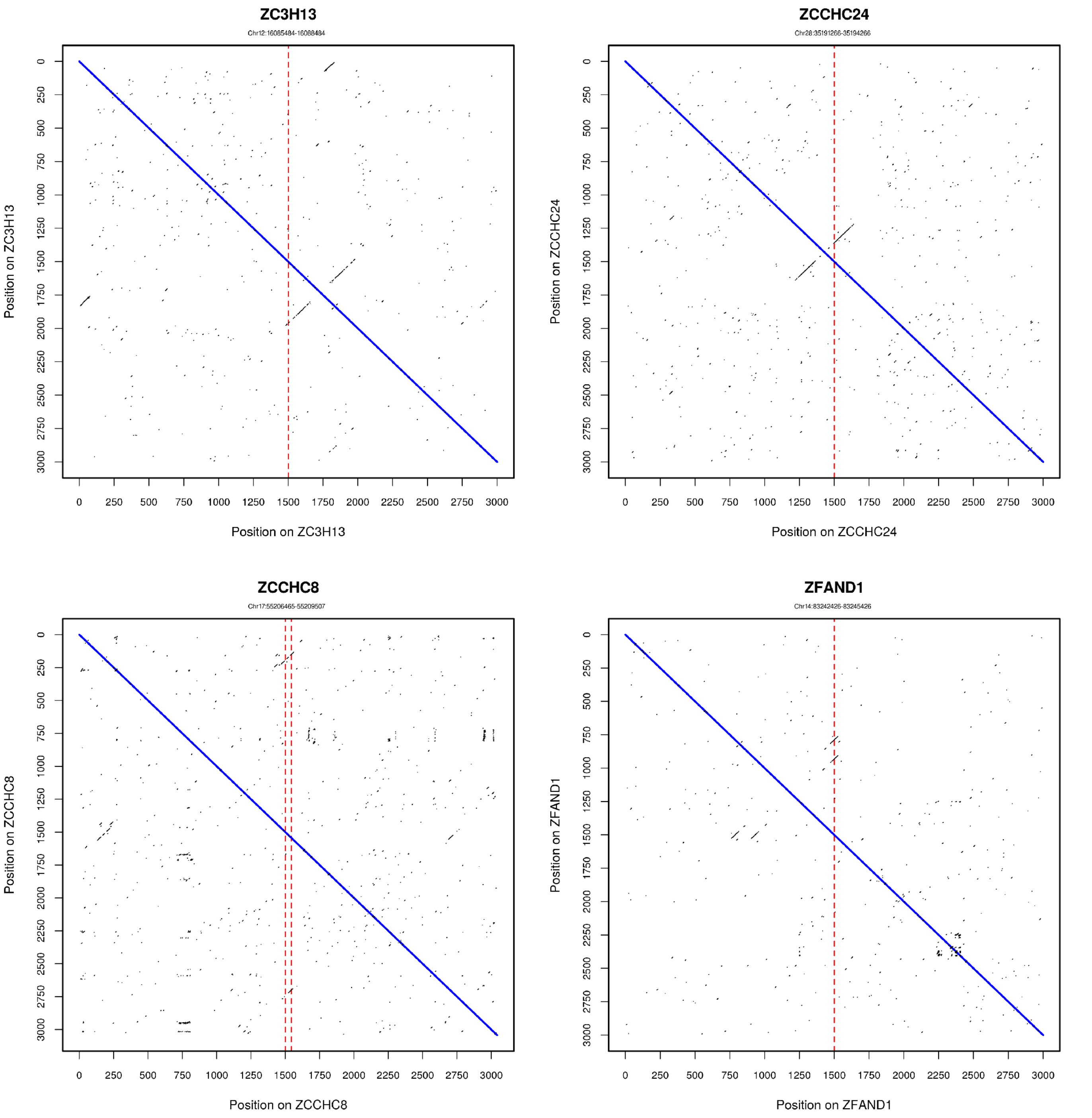
ZC3H13
Chr12:16085484-16088484
Position on ZC3H13
Position on ZC3H13
ZCCHC24
Chr28:35191266-35194266
Position on ZCCHC24
Position on ZCCHC24
ZCCHC8
Chr17:55206465-55209507
Position on ZCCHC8
Position on ZCCHC8
ZFAND1
Chr14:83242426-83245426
Position on ZFAND1
Position on ZFAND1

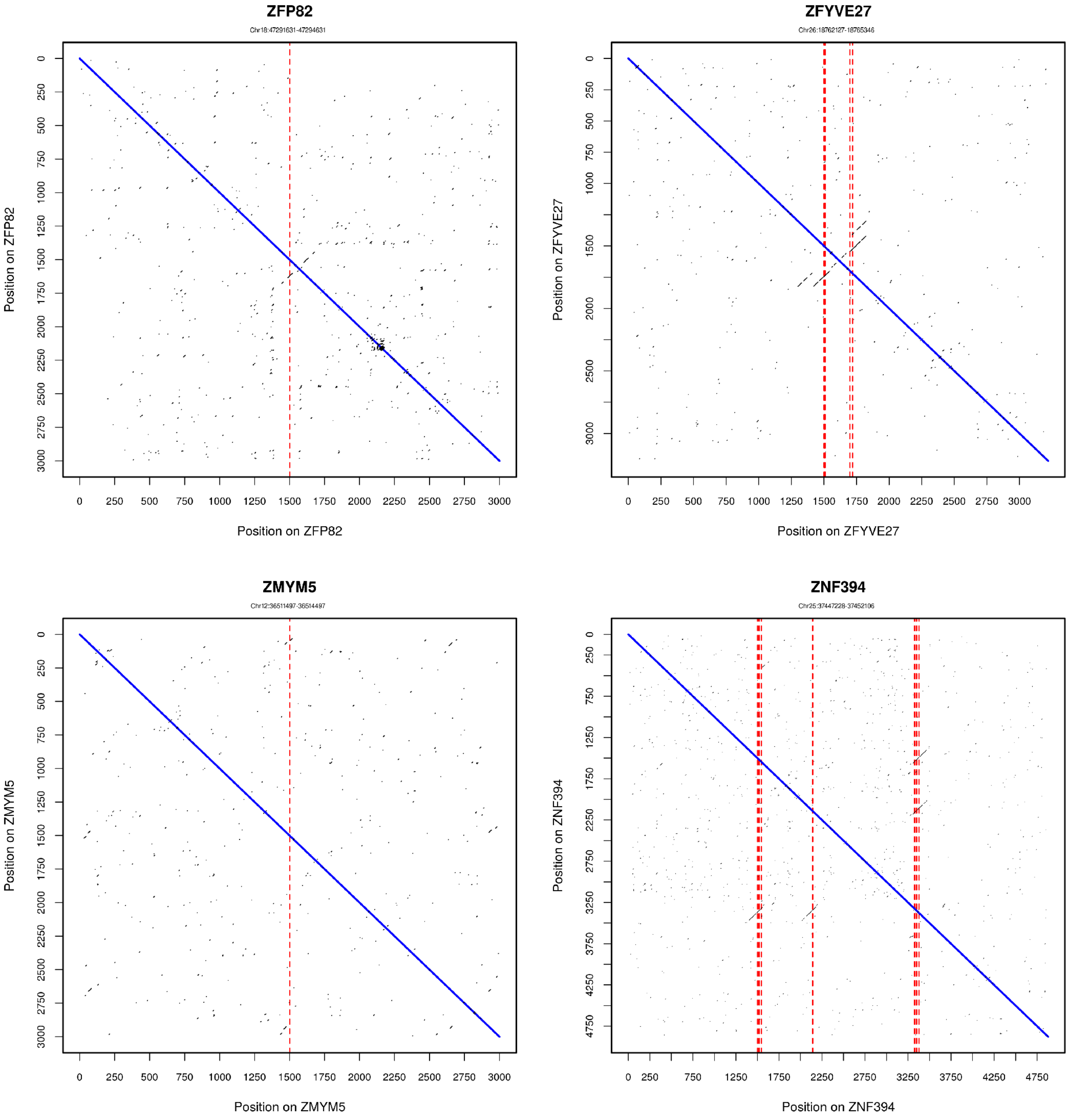

ZFP82
Chr18:47291631-47294631
Position on ZFP82
Position on ZFP82
ZFYVE27
Chr26:18762127-18765346
Position on ZFYVE27
Position on ZFYVE27
ZMYM5
Chr12:36511497-36514497
Position on ZMYM5
Position on ZMYM5
ZNF394
Chr25:37447228-37452106
Position on ZNF394
Position on ZNF394

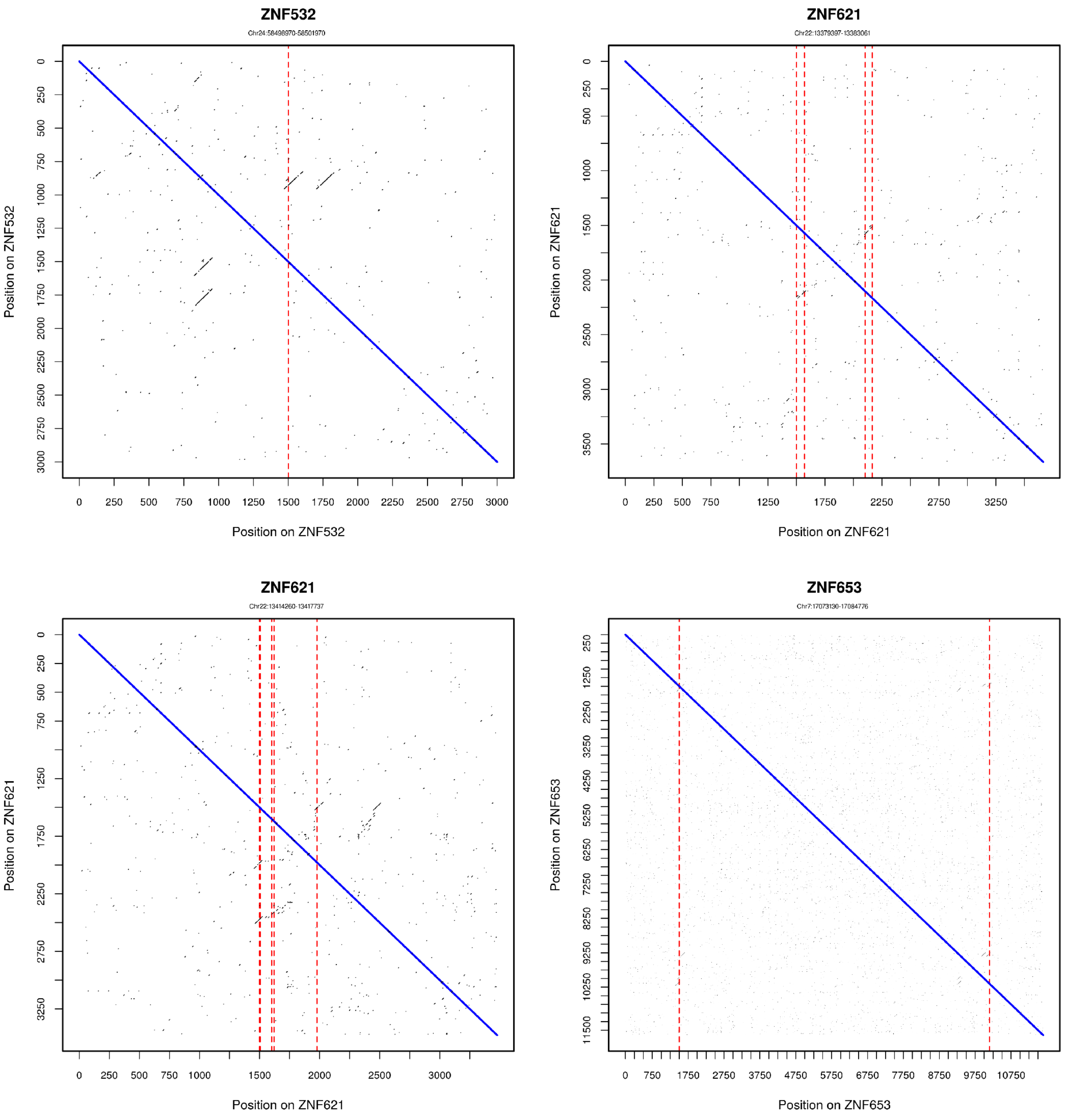

ZNF532
Chr24:58498970-58501970
Position on ZNF532
Position on ZNF532
ZNF621
Chr22:13379397-13383061
Position on ZNF621
Position on ZNF621
ZNF621
Chr22:13414260-13417737
Position on ZNF621
Position on ZNF621
ZNF653
Chr7:17073130-17084776
Position on ZNF653
Position on ZNF653

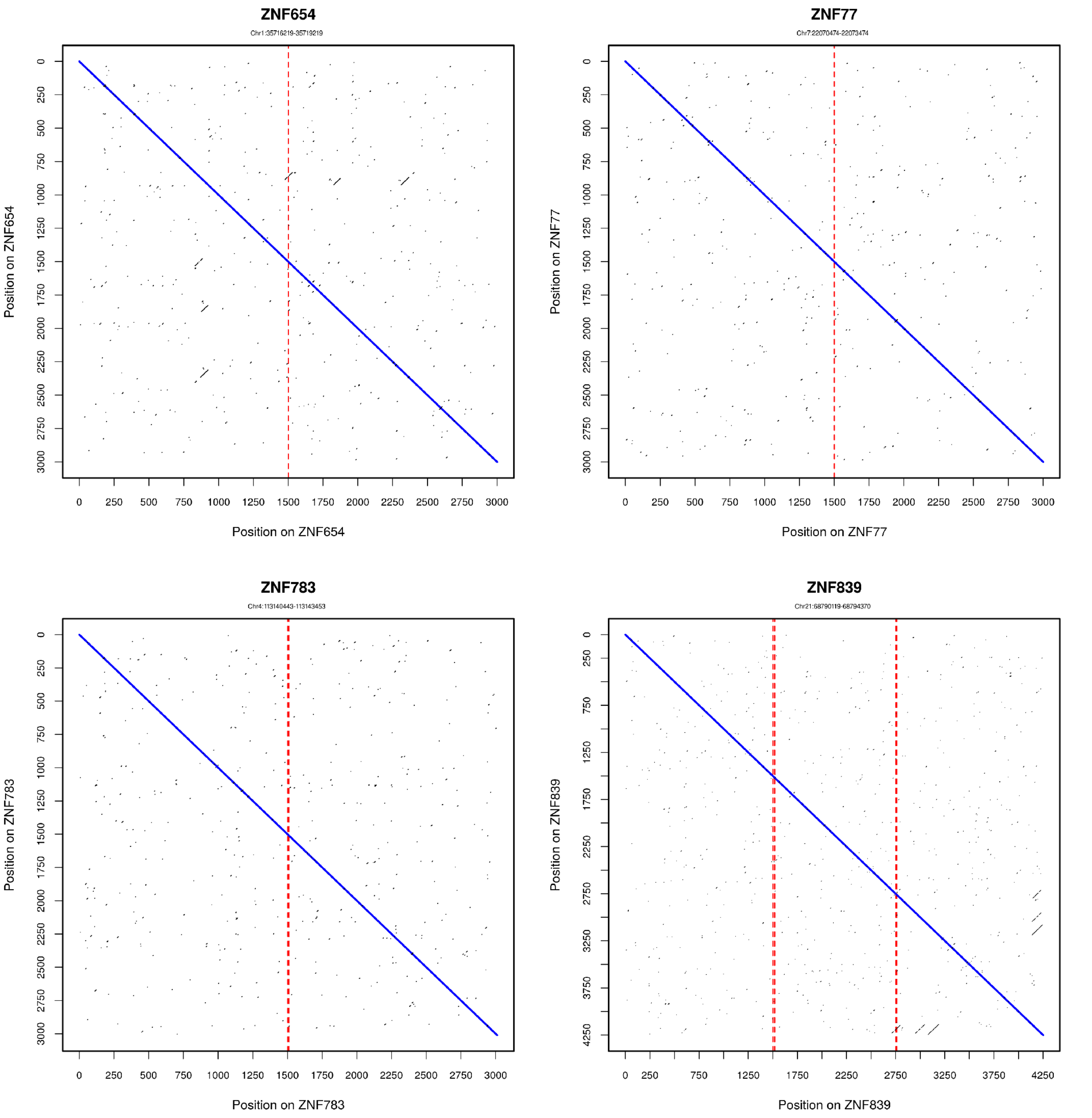

ZNF654
Chr1:35716219-35719219
Position on ZNF654
Position on ZNF654
ZNF77
Chr7:22070474-22073474
Position on ZNF77
Position on ZNF77
ZNF783
Chr4:113140443-113143453
Position on ZNF783
Position on ZNF783
ZNF839
Chr21:68790119-68794370
Position on ZNF839
Position on ZNF839

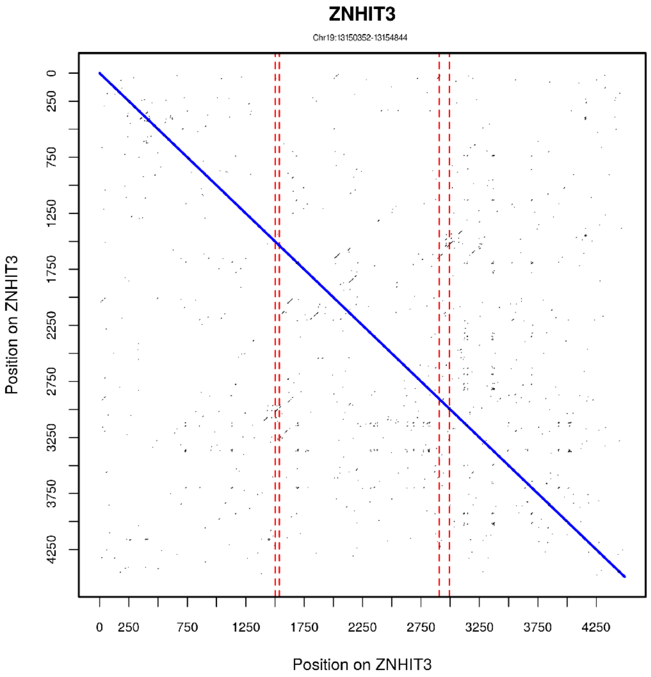

ZNHIT3
Chr19:13150352-13154844
Position on ZNHIT3
Position on ZNHIT3
0
250
750
1250
1750
2250
2750
3250
3750
4250
