# Supplementary material for: Widespread cis-regulation of RNA editing in a large mammal
Source: RNA. 2019 Mar;25(3):319–35. doi: 10.1261/rna.066902.118 (PMC6380278; doi:10.1261/rna.066902.118)

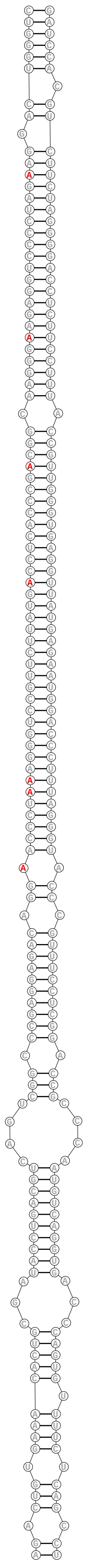

AAGAB

Strands Chr10:14007293-14007412
and Chr10:14008669-14008789

AARS2

Strands Chr23:17653114-17653321
and Chr23:17653527-17653739

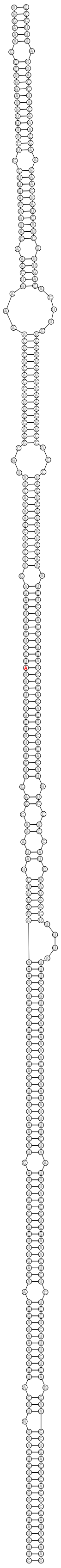

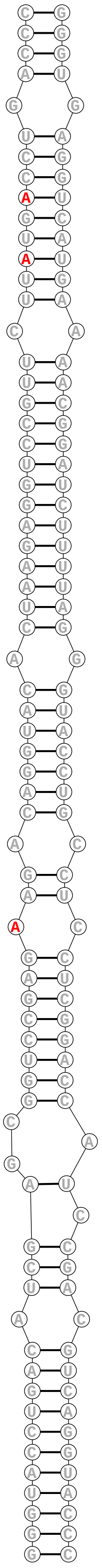

AASDHPPT

Strands Chr15:1788508-1788574
and Chr15:1789130-1789196

ABCB8

Strands Chr4:114418113-114418191
and Chr4:114418523-114418601

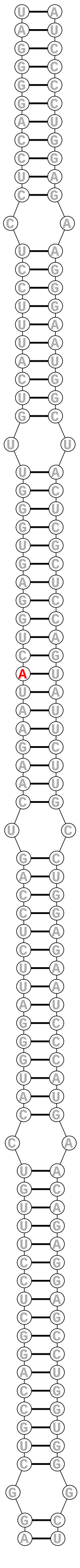

ABHD17C part A

Strands Chr21:27103385-27103541
and Chr21:27106019-27106174

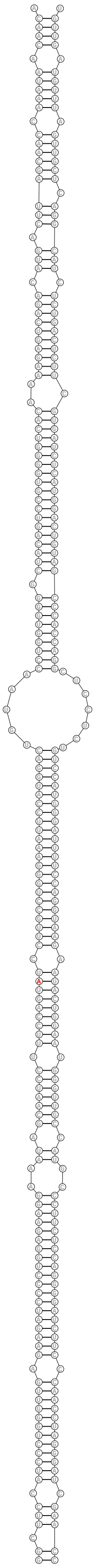

ABHD17C part B

Strands Chr21:27106056-27106229
and Chr21:27106543-27106725

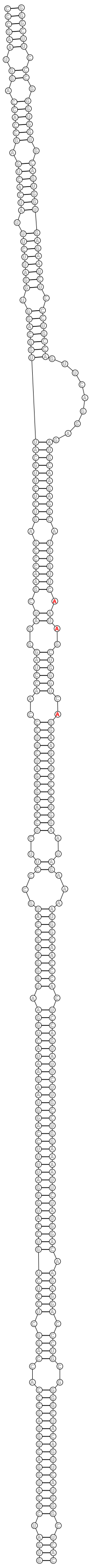

ABHD8

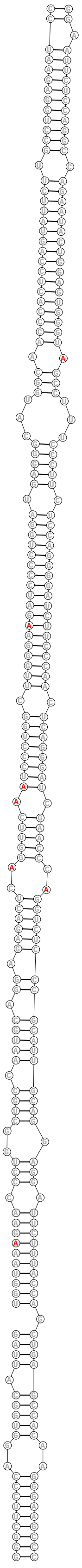

Strands Chr7:5747212-5747347
and Chr7:5748368-5748500

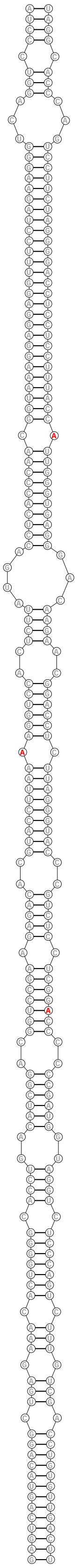

ABO

Strands Chr11:104233617-104233750
and Chr11:104238108-104238240

ACACA

Strands Chr19:13792648-13792693
and Chr19:13793223-13793264

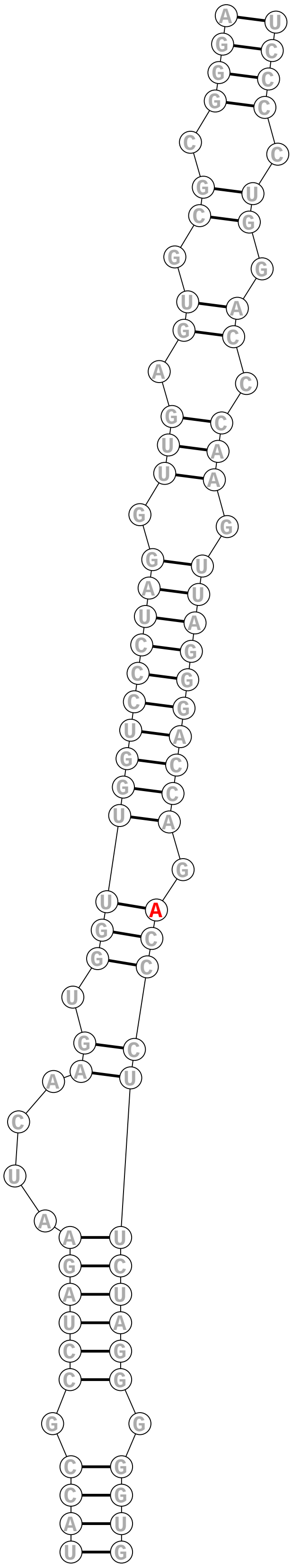

ACADM

Strands Chr3:69347223-69347414
and Chr3:69348174-69348376

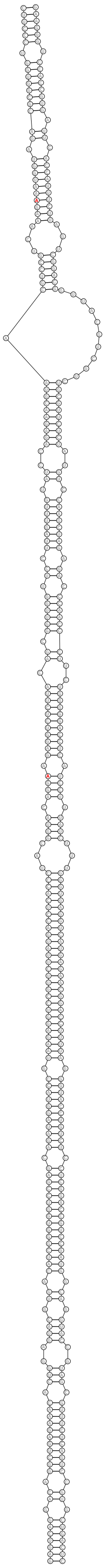

ACADSB

Strands Chr26:43148972-43149065
and Chr26:43149805-43149898

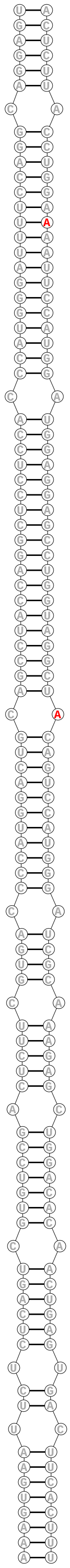

ACLY

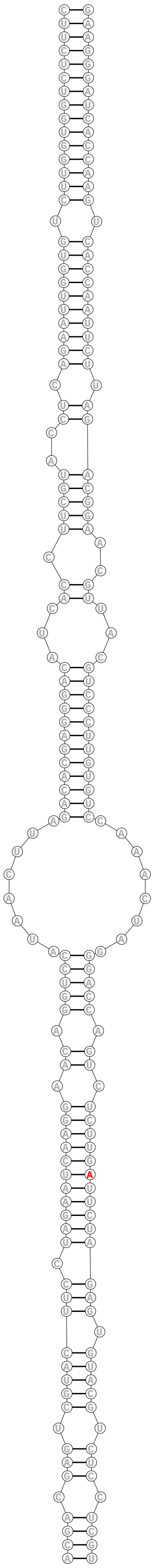

Strands Chr19:42714474-42714573
and Chr19:42715206-42715304

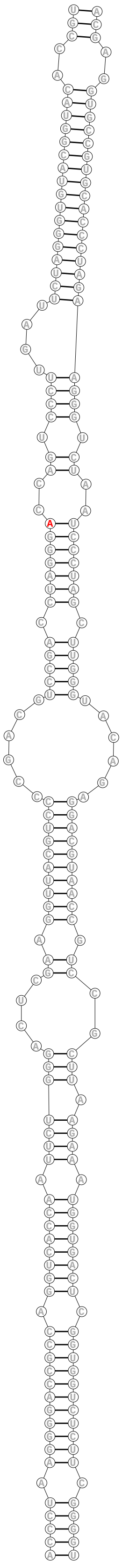

ACSL1

Strands Chr27:14237252-14237356
and Chr27:14237898-14237997

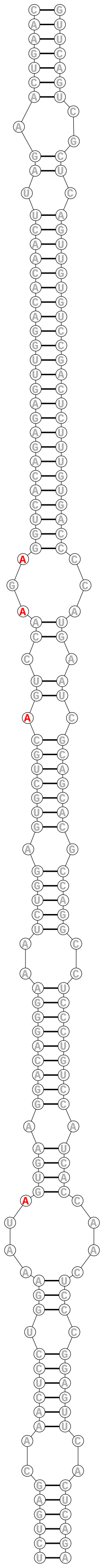

ACSS2

Strands Chr13:64806088-64806182
and Chr13:64807115-64807210

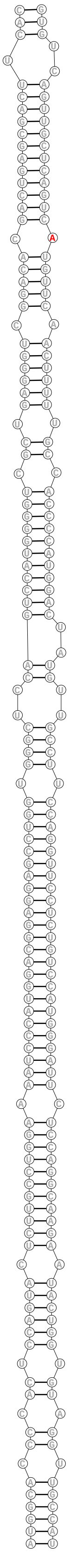

ACTR10

Strands Chr10:70784937-70785045
and Chr10:70785387-70785498

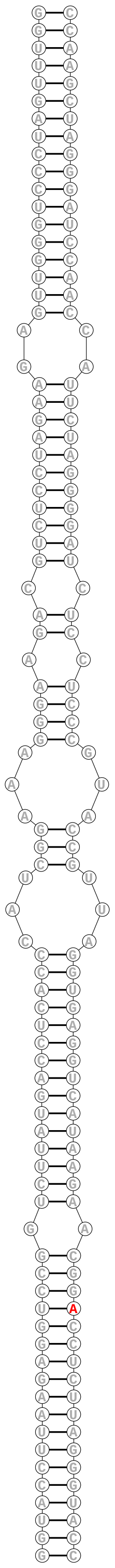

ADAMTS10 part A

Strands Chr7:18399049-18399130
and Chr7:18399512-18399593

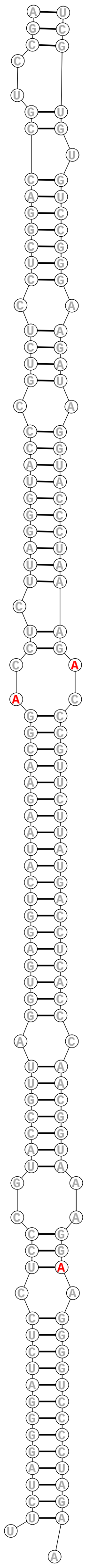

ADAMTS10 part B

Strands Chr7:18399532-18399613
and Chr7:18399659-18399738

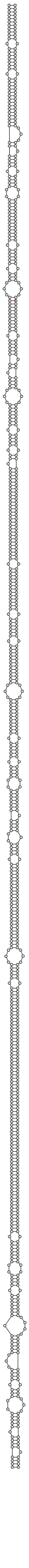

ADAT2 part A

Strands Chr9:81960727-81961115
and Chr9:81962127-81962519

ADAT2 part B

Strands Chr9:81949403-81949526
and Chr9:81950406-81950532

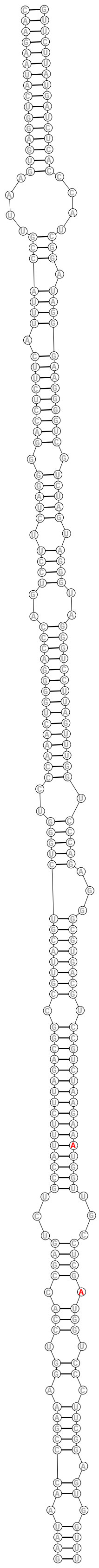

AGTRAP

Strands Chr16:42813122-42813236
and Chr16:42813727-42813836

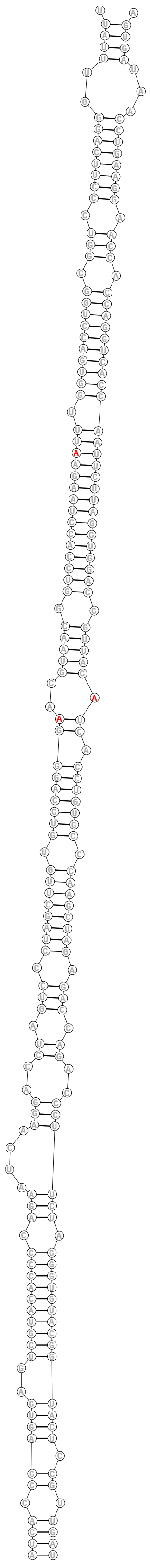

AJUBA

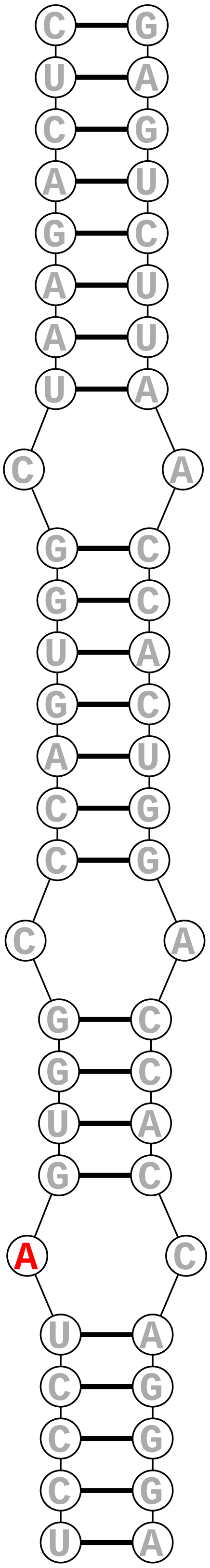

Strands Chr10:21707828-21707854
and Chr10:21708742-21708768

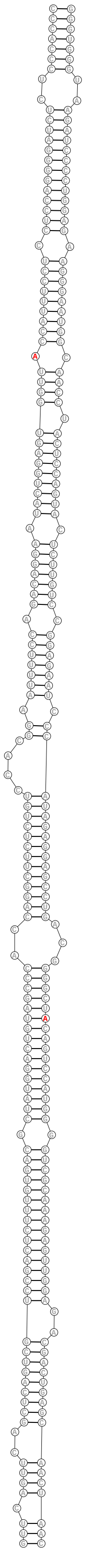

AKAP8L part A

Strands Chr7:8780834-8780969
and Chr7:8785934-8786066

AKAP8L part B

Strands Chr7:8782231-8782370
and Chr7:8782810-8782950

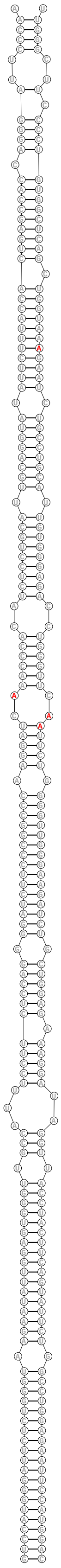

ALDH5A1

Strands Chr23:32966015-32966126
and Chr23:32966819-32966930

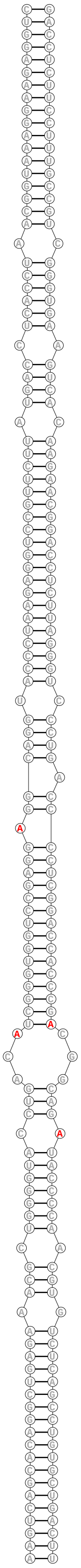

ALDH9A1

Strands Chr3:3274079-3274192
and Chr3:3275109-3275222

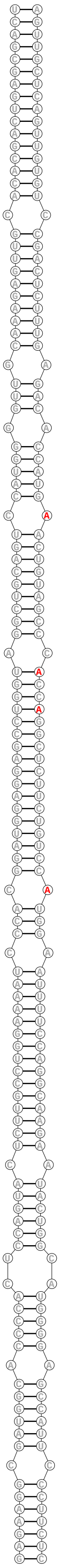

ALKBH1 part A

Strands Chr10:89894716-89894752
and Chr10:89895588-89895625

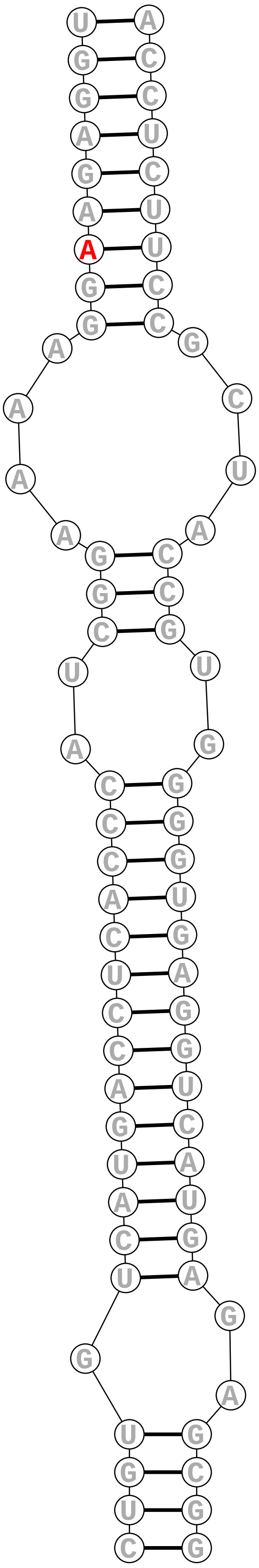

ALKBH1 part B

Strands Chr10:89894843-89894870
and Chr10:89895719-89895746

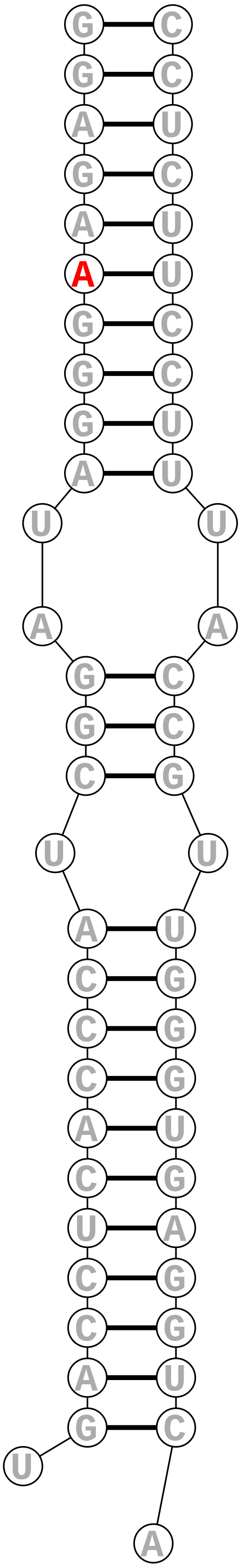

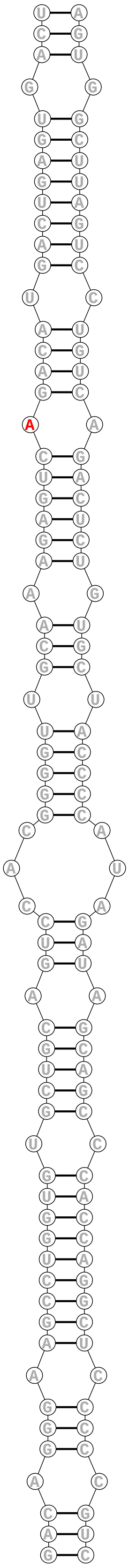

ALKBH3

Strands Chr15:74873188-74873251
and Chr15:74873767-74873830

ANKH

Strands Chr20:58539920-58540041
and Chr20:58540124-58540245

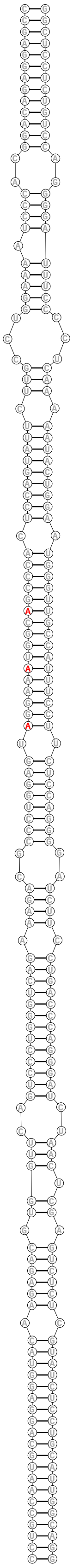

ANKRD26 part A

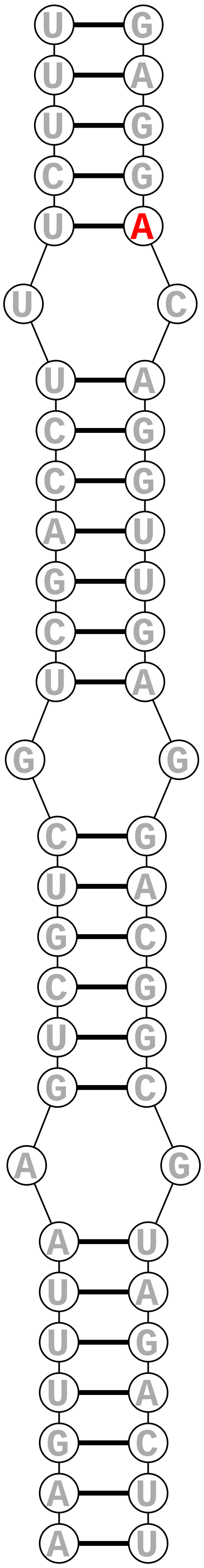

Strands Chr13:17907358-17907385
and Chr13:17908519-17908546

ANKRD40

Strands Chr19:36658629-36658726
and Chr19:36659550-36659646

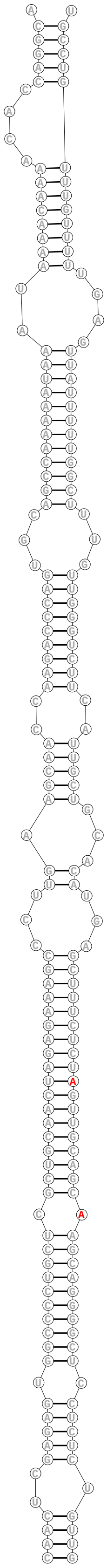

ANKRD42

Strands Chr29:12481718-12481992
and Chr29:12482653-12482925

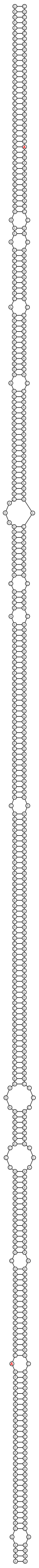

ANKS1A

Strands Chr23:9004833-9004887
and Chr23:9004912-9004969

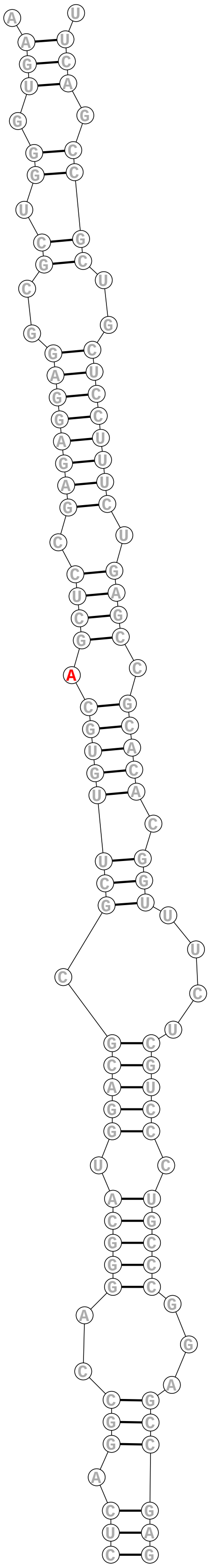

AOC3

Strands Chr19:43522731-43522802
and Chr19:43523488-43523559

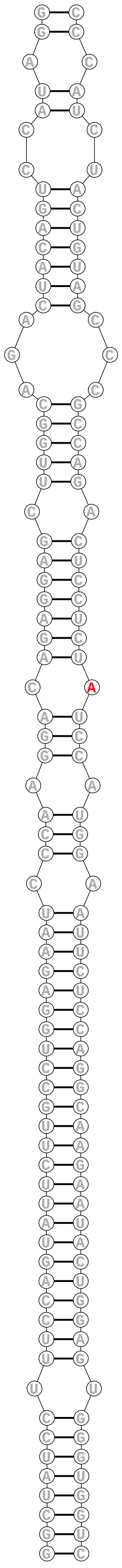

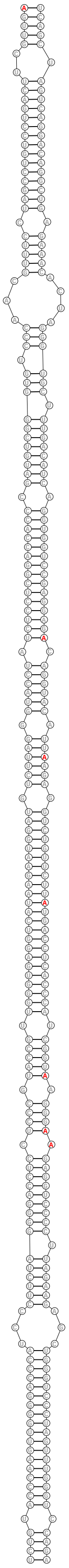

APIP

Strands Chr15:66216698-66216850
and Chr15:66217343-66217496

ARF3

Strands Chr7:2924607-2924740
and Chr7:2927165-2927298

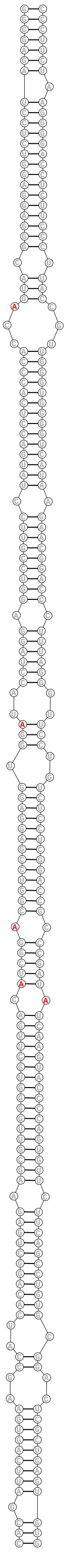

ARF4

Strands Chr22:44160910-44161052
and Chr22:44162004-44162144

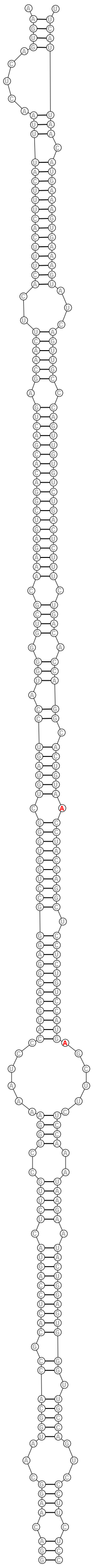

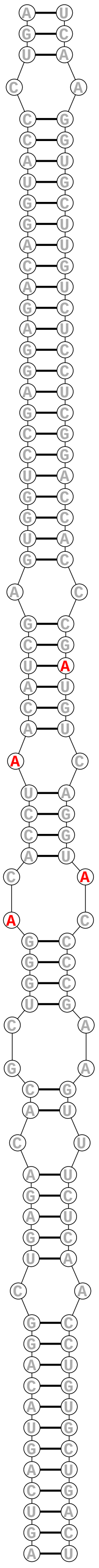

ARHGAP17

Strands Chr25:22844801-22844867
and Chr25:22847470-22847536

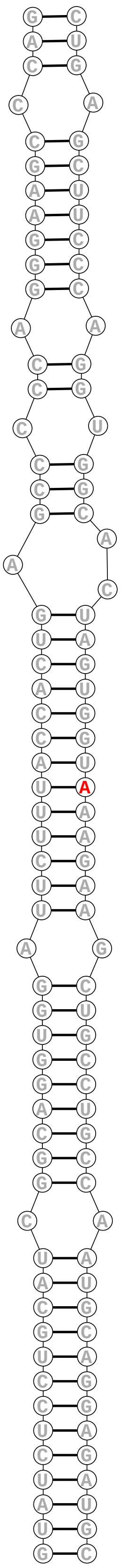

ARID5A

Strands Chr11:2531538-2531593
and Chr11:2532262-2532318

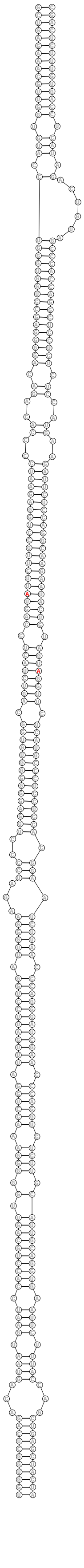

ARL13B

Strands Chr1:37902964-37903133
and Chr1:37906874-37907045

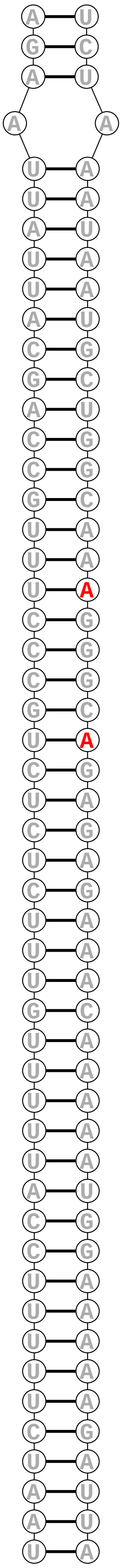

ARMC10 part A

Strands Chr4:44664989-44665039
and Chr4:44666005-44666055

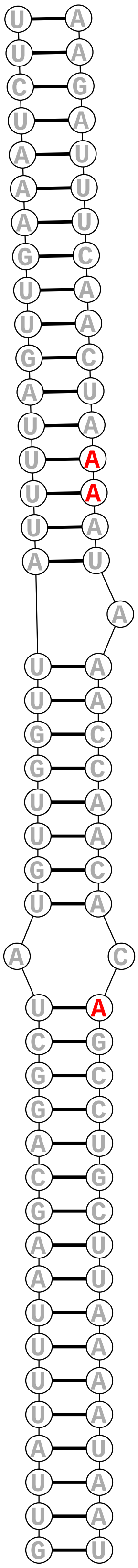

ARMC10 part B

Strands Chr4:44664945-44664987
and Chr4:44666088-44666131

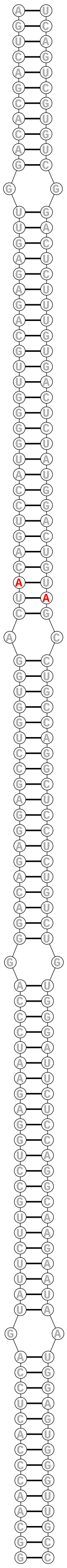

ASAP3

Strands Chr2:130016743-130016839
and Chr2:130017822-130017918

ASMTL

Strands Chr26:24668944-24669077
and Chr26:24669401-24669537

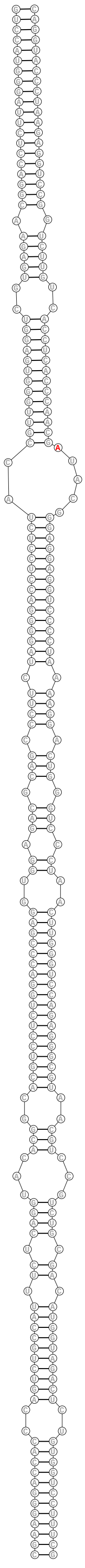

ATF5

Strands Chr18:56727493-56727667
and Chr18:56728335-56728508

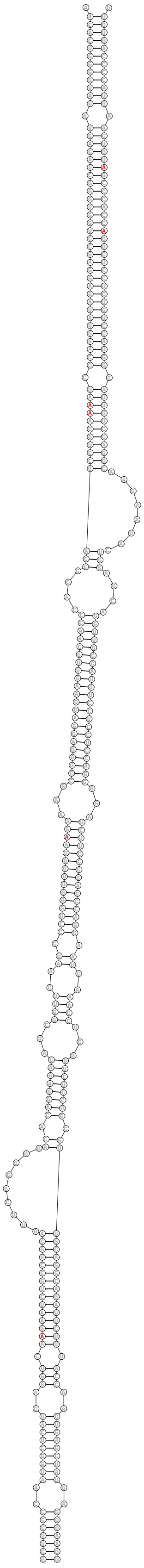

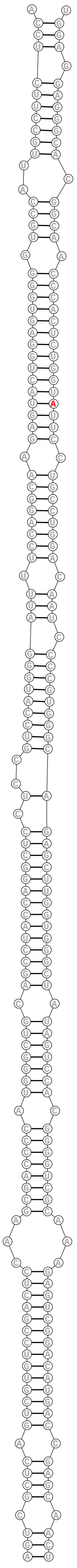

ATG16L1

Strands Chr3:113608433-113608548
and Chr3:113608962-113609075

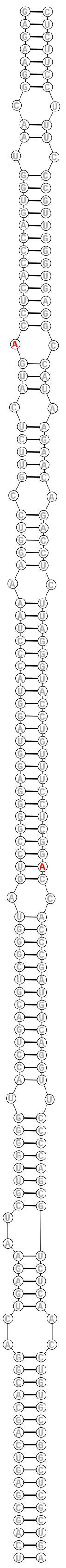

ATOX1

Strands Chr7:64947667-64947780
and Chr7:64948634-64948745

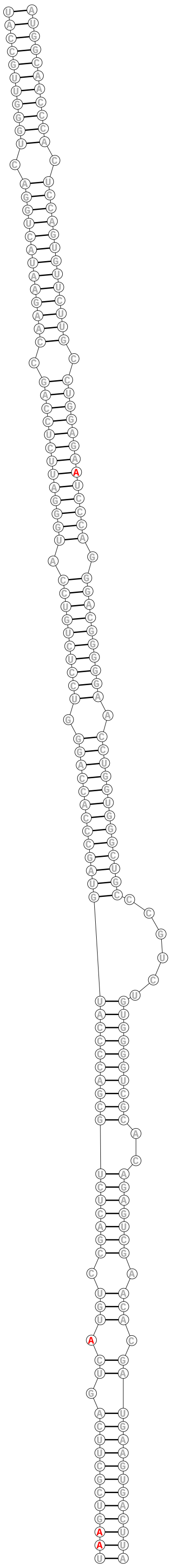

ATP13A1

Strands Chr7:3604682-3604782
and Chr7:3605148-3605255

ATP13A4

Strands Chr1:74619517-74619645
and Chr1:74628874-74629003

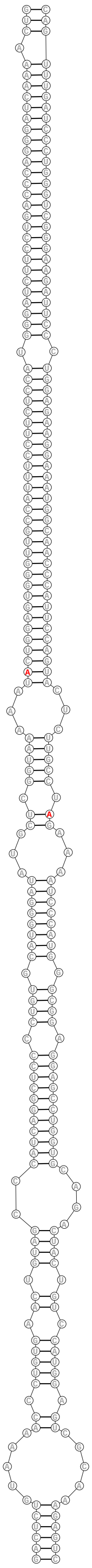

ATP2C2 part A

Strands Chr18:10659232-10659421
and Chr18:10660210-10660396

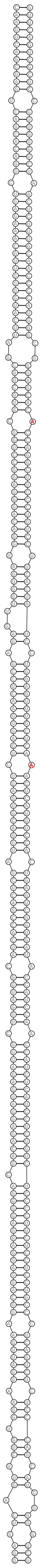

ATP2C2 part B

Strands Chr18:10663241-10663345
and Chr18:10663910-10664013

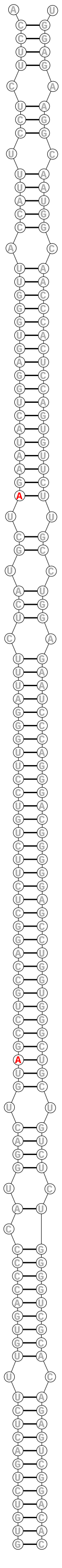

ATP6V1G1

Strands Chr8:105500383-105500873
and Chr8:105501273-105501752

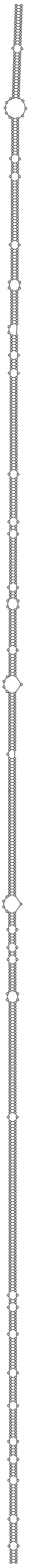

ATPAF2

Strands Chr19:35127619-35127769
and Chr19:35128155-35128305

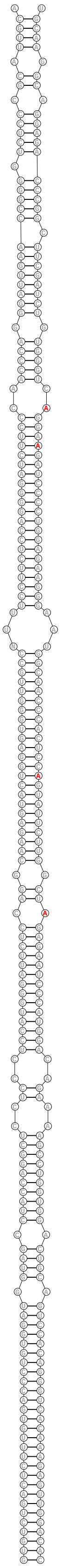

AURKAIP1

Strands Chr16:52399177-52399577
and Chr16:52399938-52400376

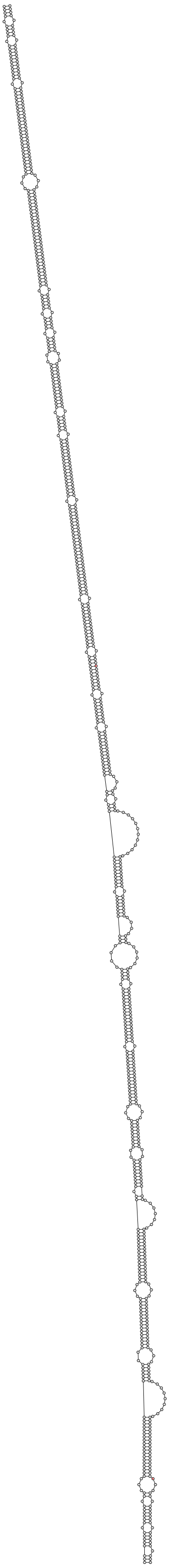

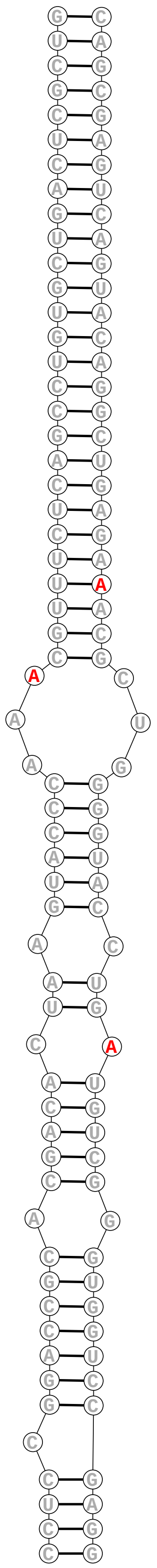

B3GNT3 part A

Strands Chr7:5273761-5273818
and Chr7:5274828-5274884

B3GNT3 part B

Strands Chr7:5273994-5274152
and Chr7:5274828-5274986

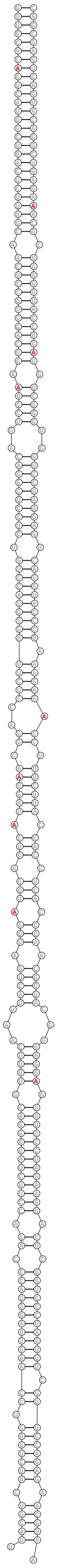

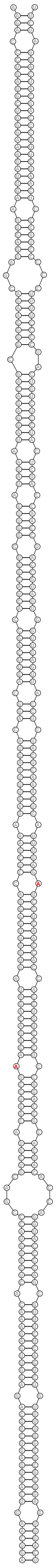

B4GALT1 part A

Strands Chr8:76183795-76183987
and Chr8:76189889-76190082

B4GALT1 part B

Strands Chr8:76184902-76186713
and Chr8:76188293-76190085

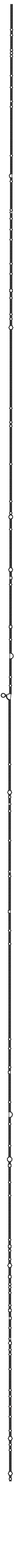

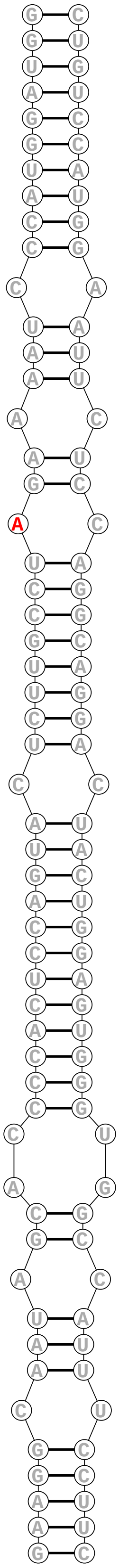

BCDIN3D

Strands Chr5:30213053-30213105
and Chr5:30213480-30213532

BET1

Strands Chr4:11166744-11167046
and Chr4:11168207-11168503

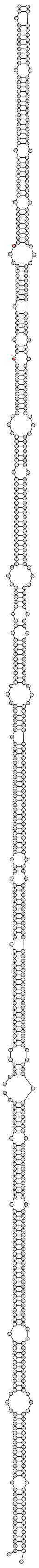

BICDL1

Strands Chr17:64704951-64705053
and Chr17:64705232-64705334

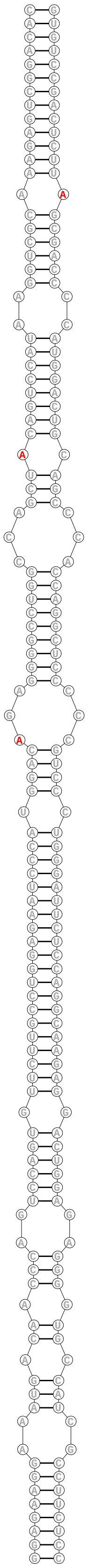

BLCAP

Strands Chr13:67116385-67116472
and Chr13:67116925-67117012

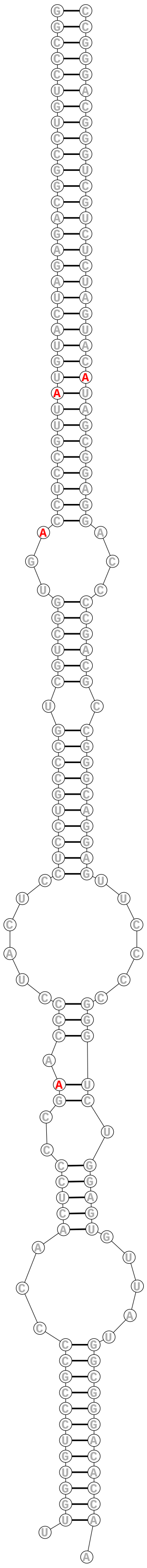

BOD1 part A

Strands Chr20:5229883-5230539
and Chr20:5231653-5232309

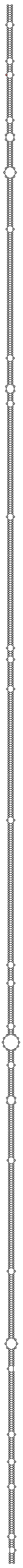

BOD1 part B

Strands Chr20:5233811-5233977
and Chr20:5234736-5234903

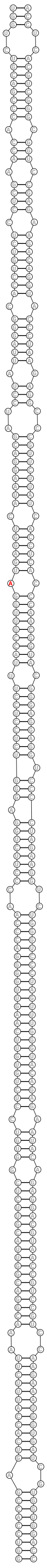

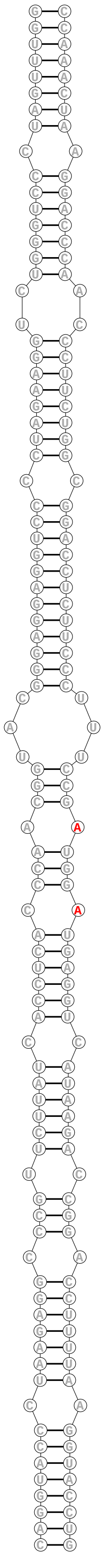

BOD1 part C

Strands Chr20:5234762-5234845
and Chr20:5235665-5235748

BRF1

Strands Chr21:71201261-71201365
and Chr21:71202259-71202360

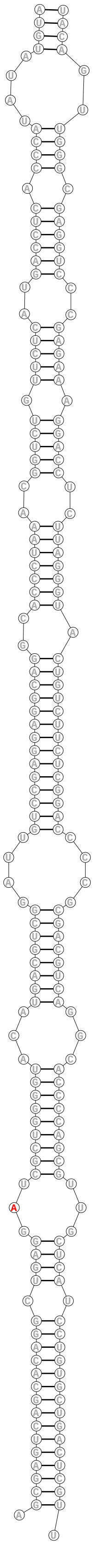

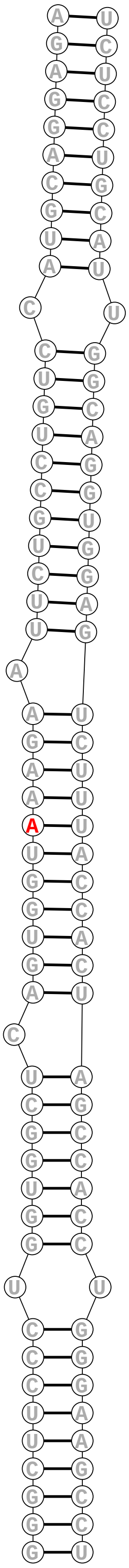

BSPRY part A

Strands Chr8:104314549-104314600
and Chr8:104315155-104315204

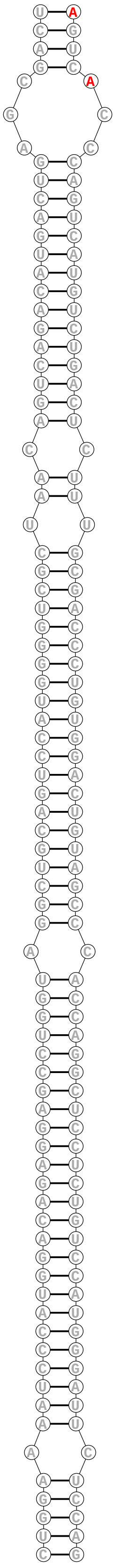

BSPRY part B

Strands Chr8:104313853-104313931
and Chr8:104316027-104316105

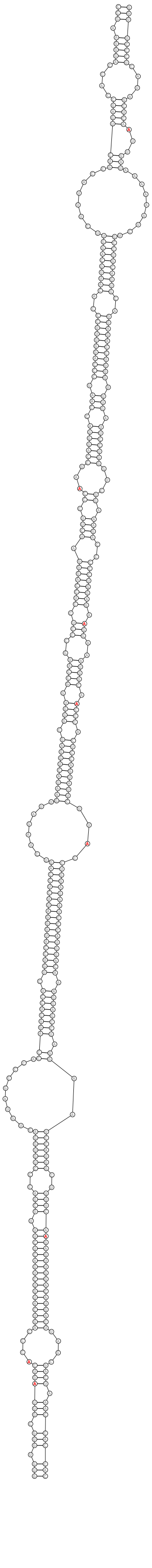

BSPRY part C

Strands Chr8:104316016-104316224
and Chr8:104317313-104317512

BSPRY part D

Strands Chr8:104327763-104327905
and Chr8:104328420-104328559

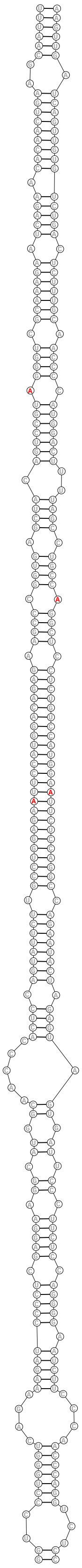

BZW2

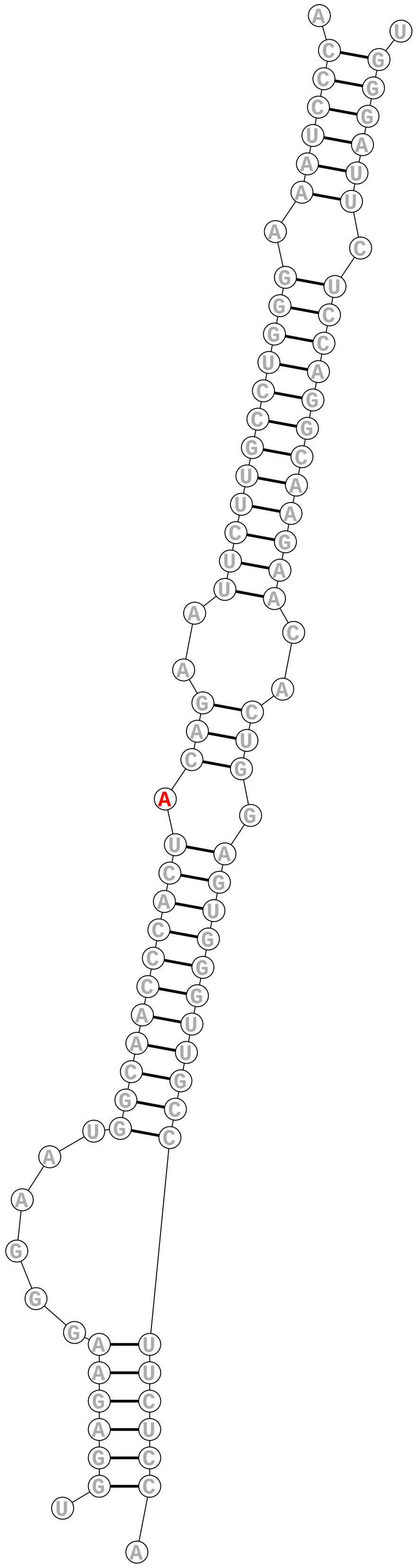

Strands Chr4:25159762-25159811
and Chr4:25160487-25160530

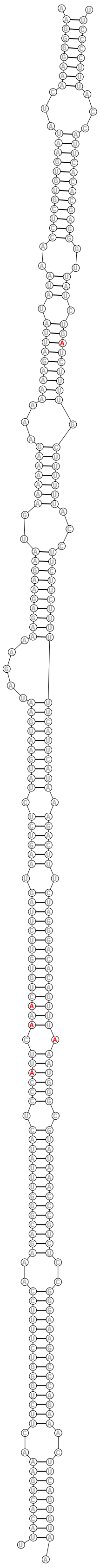

CA8 part A

Strands Chr14:27650884-27651033
and Chr14:27651579-27651722

CA8 part B

Strands Chr14:27651037-27651233
and Chr14:27651356-27651554

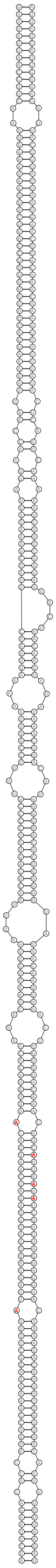

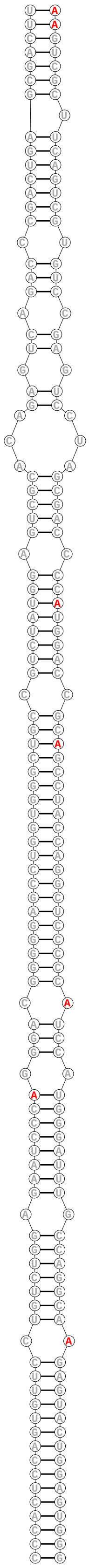

CABIN1 part A

Strands Chr17:73351363-73351461
and Chr17:73353619-73353718

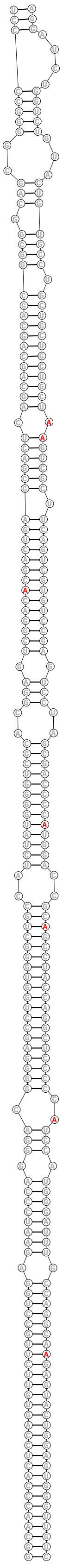

CABIN1 part B

Strands Chr17:73351501-73351633
and Chr17:73353584-73353723

CALD1 part A

Strands Chr4:99549373-99549541
and Chr4:99550220-99550388

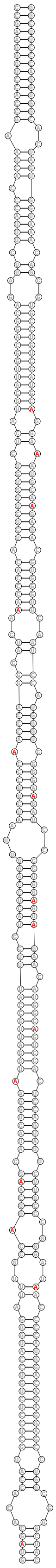

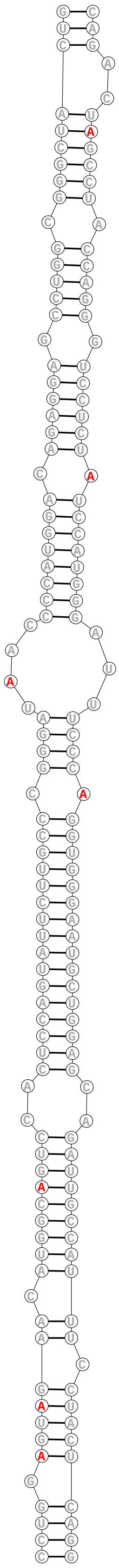

CALD1 part B

Strands Chr4:99550311-99550390
and Chr4:99550429-99550508

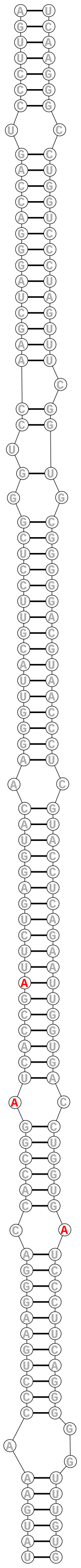

CAMKK1

Strands Chr19:25118024-25118111
and Chr19:25119354-25119442

CAMKK2

Strands Chr17:56161557-56161754
and Chr17:56163172-56163373

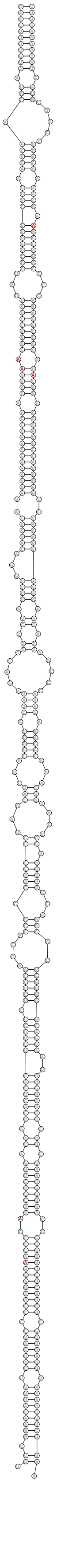

CAPN8

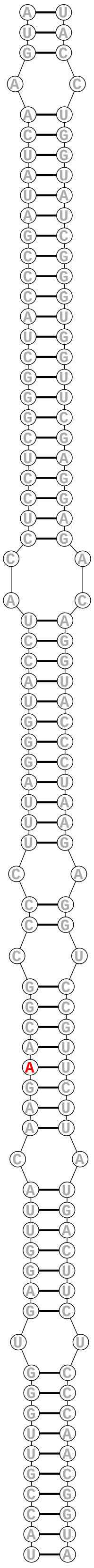

Strands Chr16:27701954-27702024
and Chr16:27702103-27702173

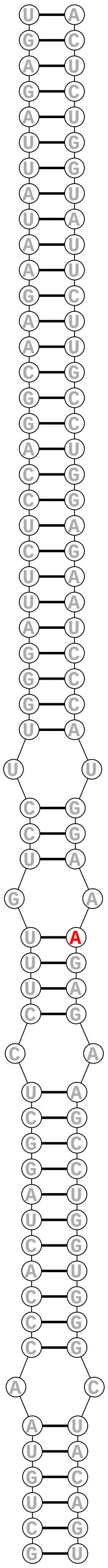

CAPSL

Strands Chr20:38296432-38296488
and Chr20:38297836-38297892

CARD19

Strands Chr8:85923334-85923375
and Chr8:85923495-85923536

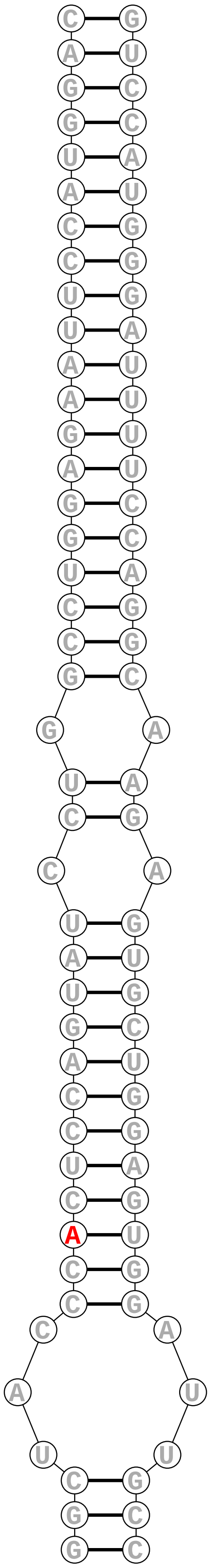

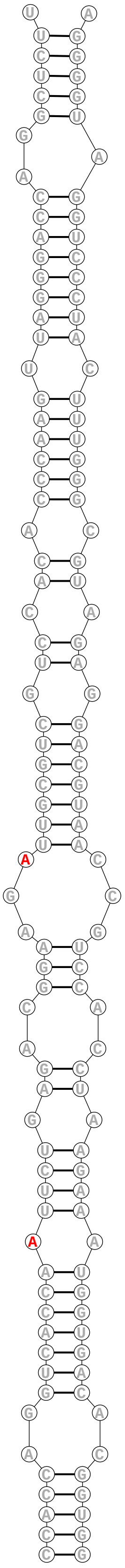

CATSPER2

Strands Chr21:55921840-55921906
and Chr21:55923052-55923117

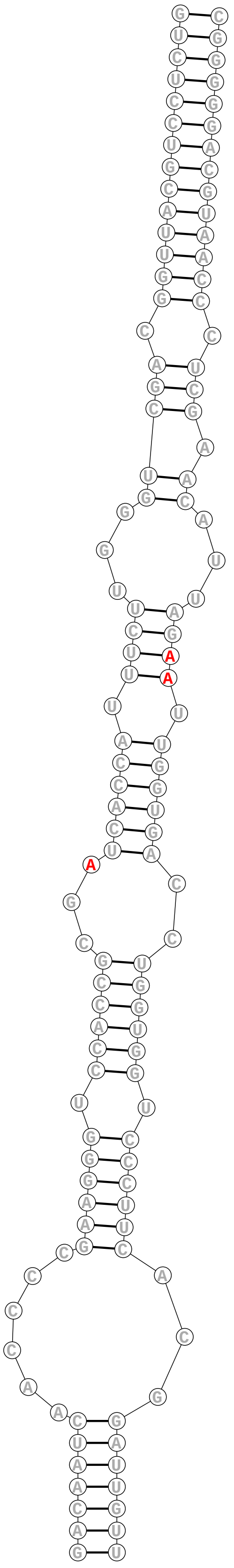

CATSPERD

Strands Chr7:19780694-19780756
and Chr7:19780869-19780928

CATSPERG

Strands Chr18:48418071-48418169
and Chr18:48418603-48418701

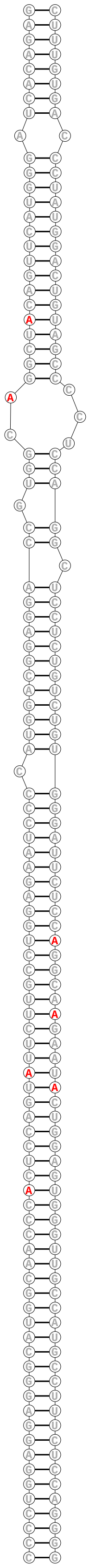

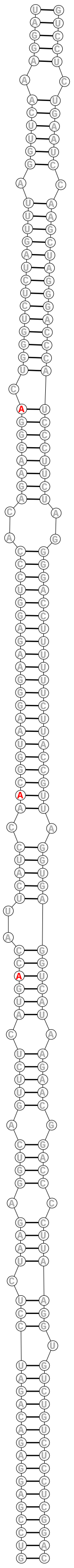

CCDC50

Strands Chr1:76561867-76561975
and Chr1:76563334-76563439

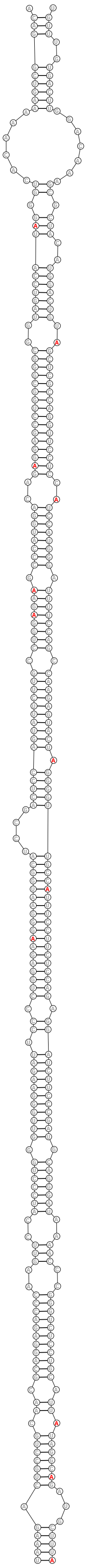

CCDC82 part A

Strands Chr15:14124543-14124704
and Chr15:14127953-14128117

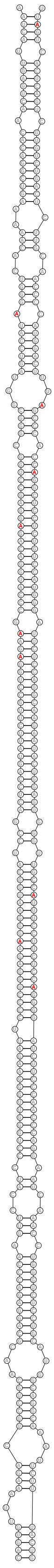

CCDC82 part B

Strands Chr15:14124537-14124719
and Chr15:14128169-14128348

CCDC82 part C

Strands Chr15:14128168-14128365
and Chr15:14129587-14129785

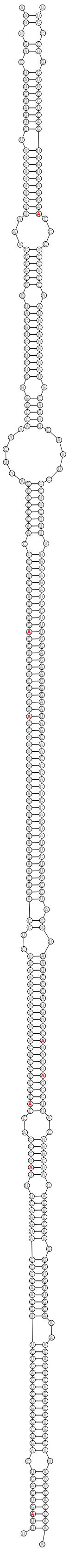

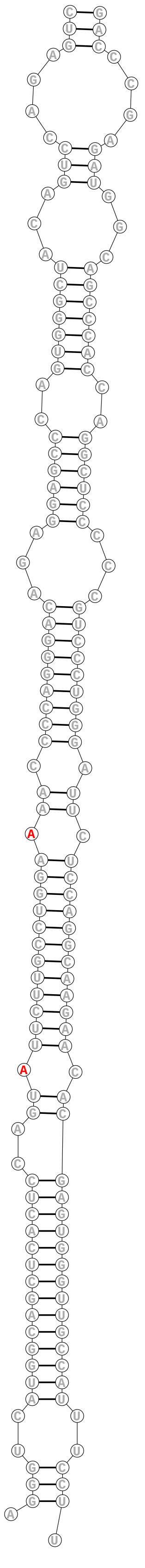

CCDC84

Strands Chr15:30114185-30114265
and Chr15:30115200-30115278

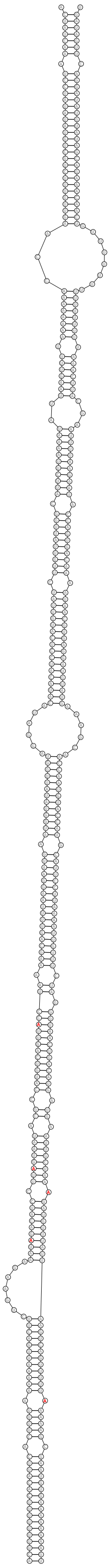

CCNDBP1 part A

Strands Chr10:38515379-38515593
and Chr10:38515893-38516107

CCNDBP1 part B

Strands Chr10:38514578-38514769
and Chr10:38517640-38517832

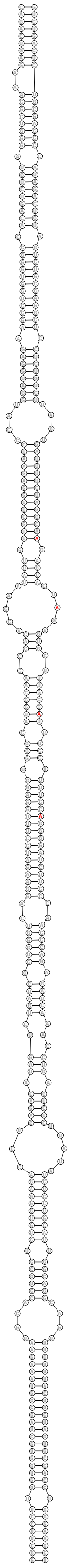

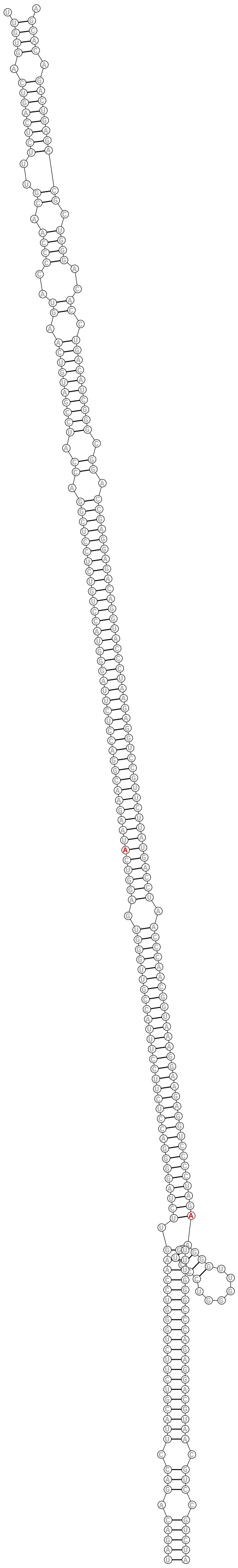

CCNI part A

Strands Chr6:93635530-93635674
and Chr6:93639192-93639349

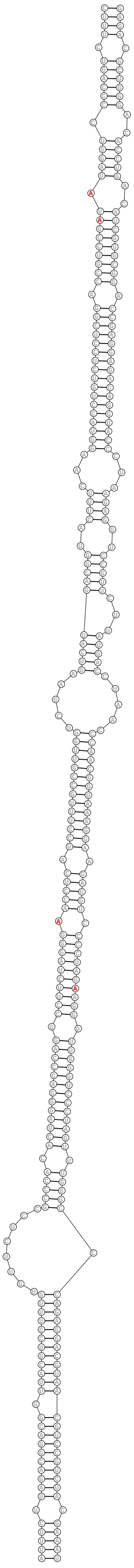

CCNI part B

Strands Chr6:93638355-93638511
and Chr6:93639202-93639355

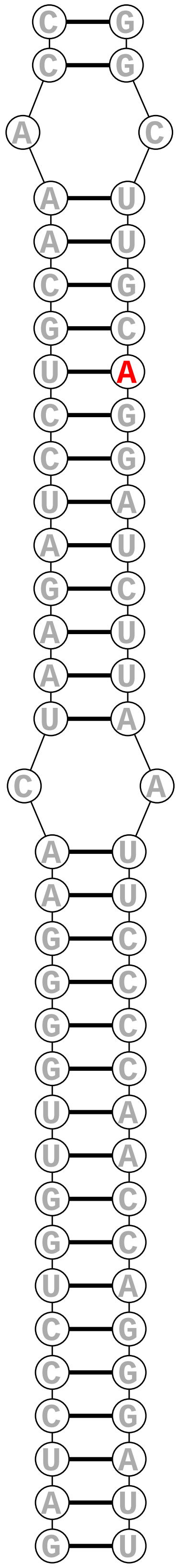

CCS

Strands Chr29:45284294-45284327
and Chr29:45285011-45285044

CCT4

Strands Chr11:60398278-60398484
and Chr11:60399258-60399460

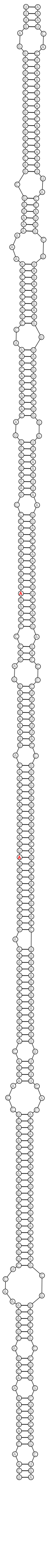

CD164 part A

Strands Chr9:41252861-41253012
and Chr9:41254382-41254543

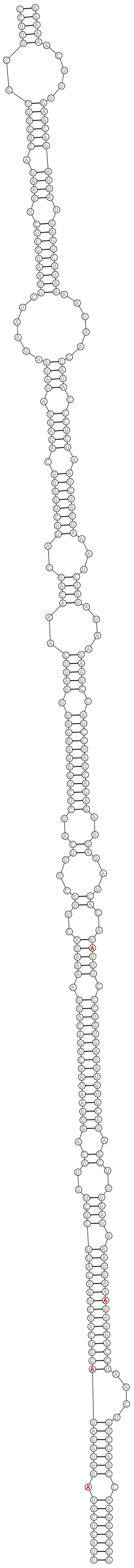

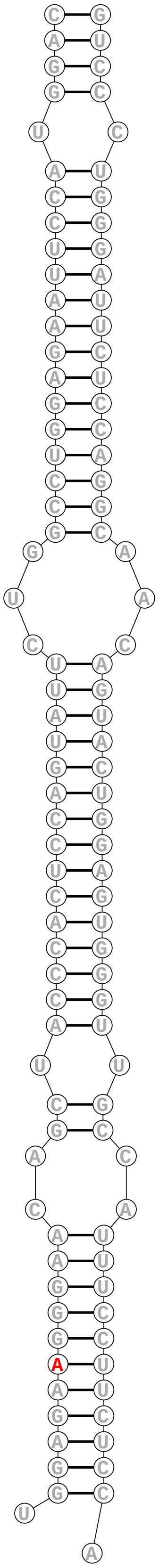

CD164 part B

Strands Chr9:41253130-41253184
and Chr9:41254046-41254100

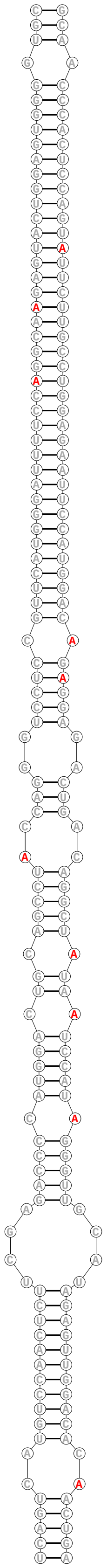

CD300LG

Strands Chr19:44362814-44362909
and Chr19:44363872-44363967

CD36 part A

Strands Chr4:40591332-40591609
and Chr4:40592605-40592884

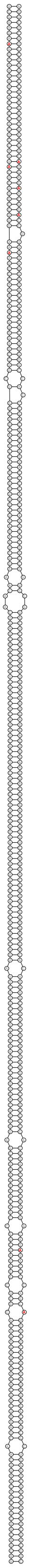

CD36 part B

Strands Chr4:40599959-40600041
and Chr4:40601005-40601072

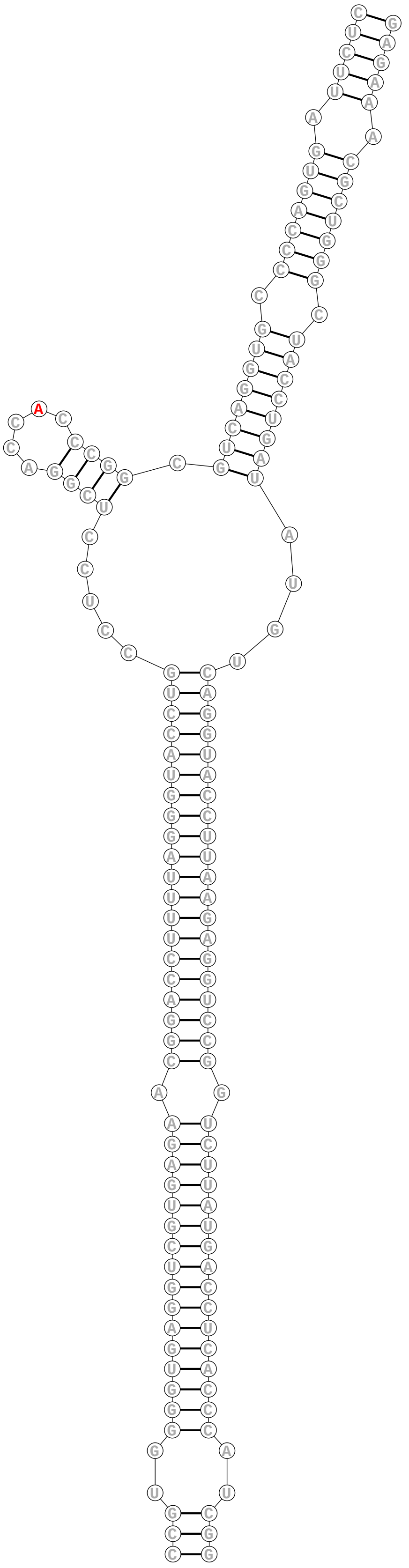

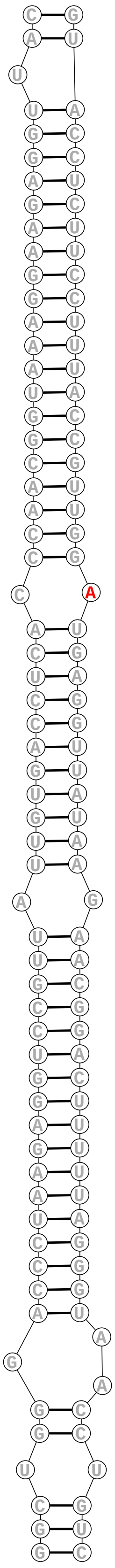

CD36 part C

Strands Chr4:40621735-40621794
and Chr4:40622240-40622299

CD4

Strands Chr5:103994430-103994564
and Chr5:103995536-103995670

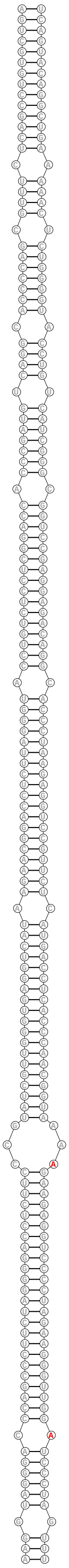

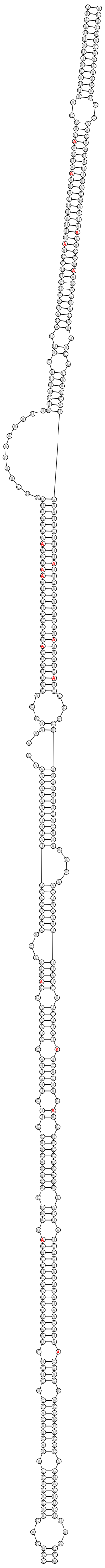

CD46

Strands Chr16:77486559-77486779
and Chr16:77487529-77487734

CD47

Strands Chr1:53113970-53114080
and Chr1:53114199-53114315

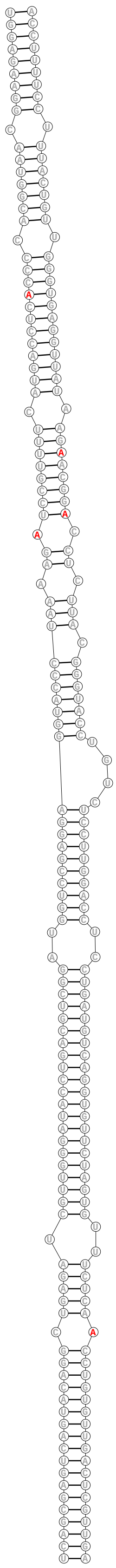

CD58

Strands Chr3:26861490-26861602
and Chr3:26861997-26862108

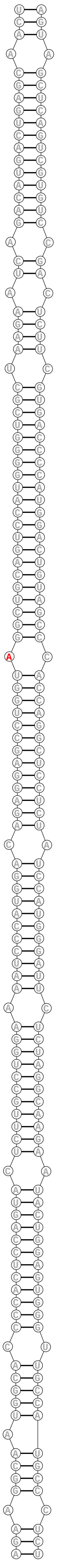

CD9 part A

Strands Chr5:104511279-104511399
and Chr5:104512161-104512281

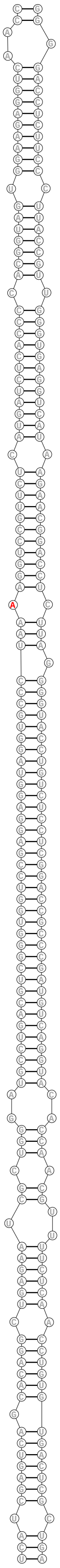

CD9 part B

Strands Chr5:104513700-104513871
and Chr5:104514476-104514629

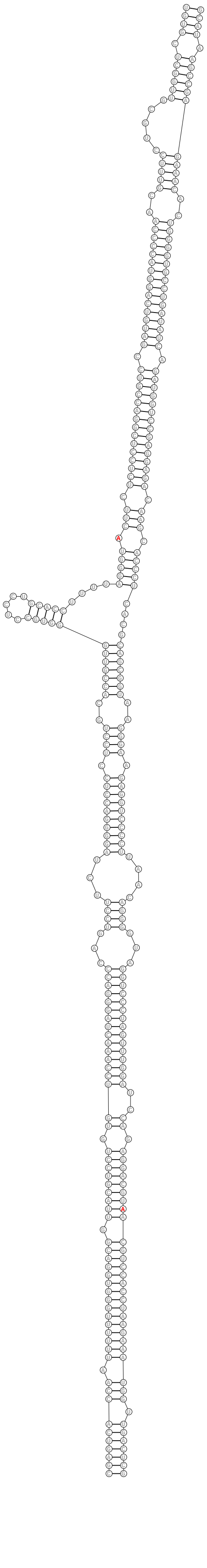

CDC16

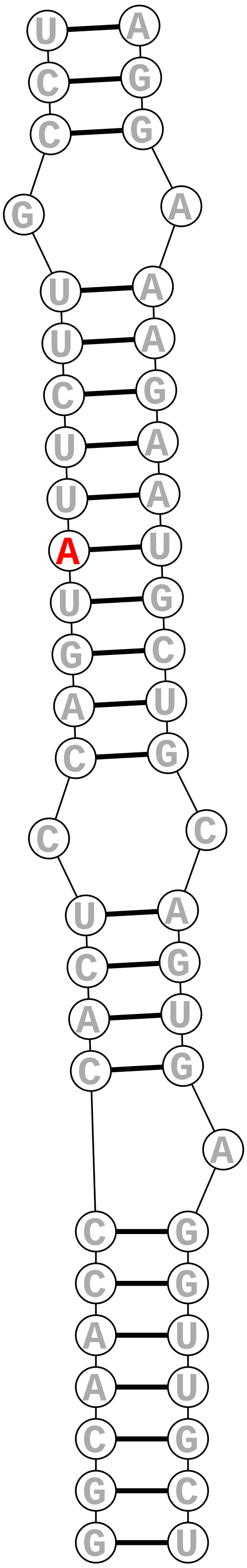

Strands Chr12:91062314-91062339
and Chr12:91063024-91063050

CDK20 part A

Strands Chr8:91628296-91628430
and Chr8:91629488-91629619

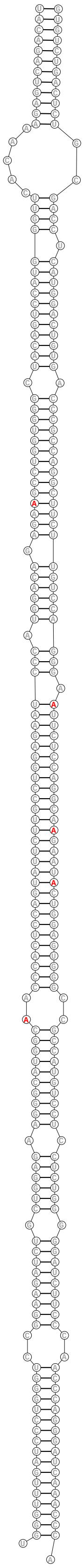

CDK20 part B

Strands Chr8:91629499-91629626
and Chr8:91631299-91631427

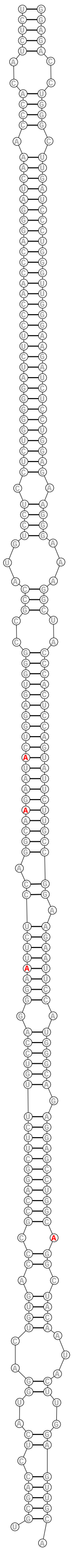

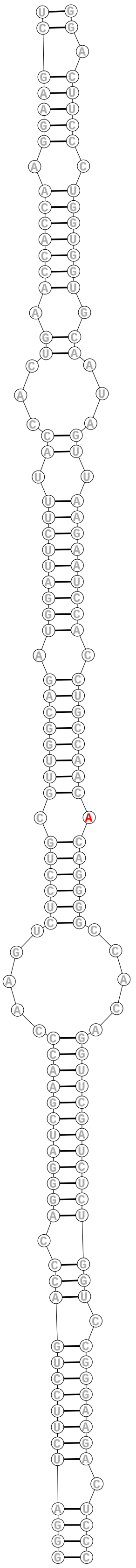

CEACAM19

Strands Chr18:52856762-52856843
and Chr18:52858191-52858274

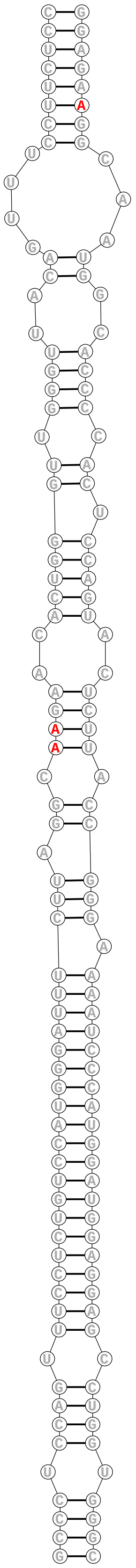

CEP70

Strands Chr1:131618869-131618939
and Chr1:131619945-131620015

CEP85

Strands Chr2:127436599-127436631
and Chr2:127437199-127437231

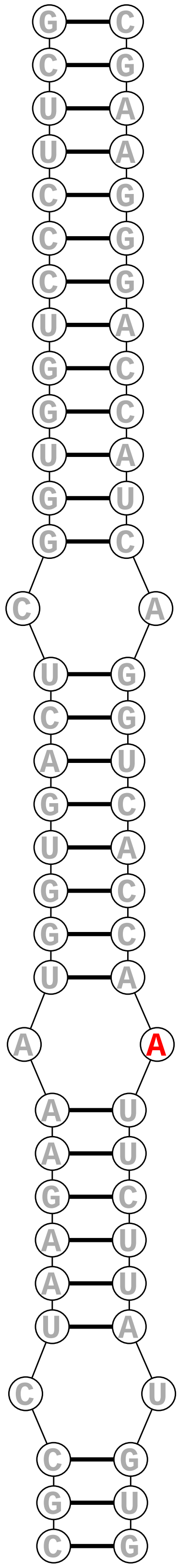

CEP95

Strands Chr19:49372722-49372778
and Chr19:49373389-49373446

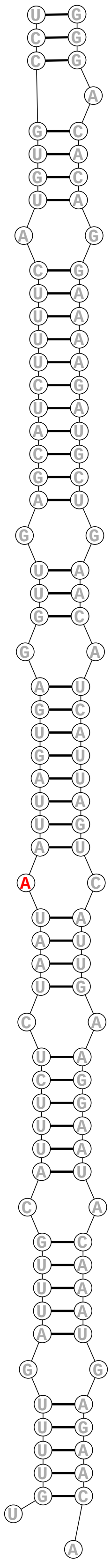

CFAP298

Strands Chr1:2206504-2206624
and Chr1:2206977-2207095

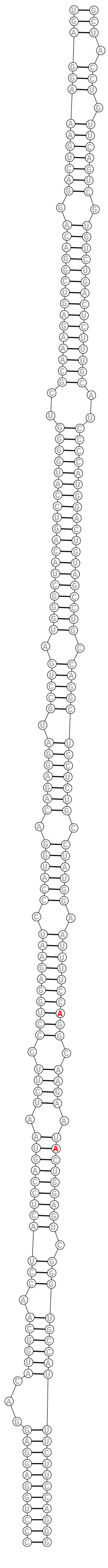

CFAP36

Strands Chr11:38092665-38092806
and Chr11:38094207-38094347

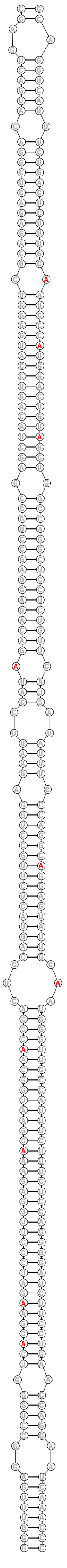

CHD1L

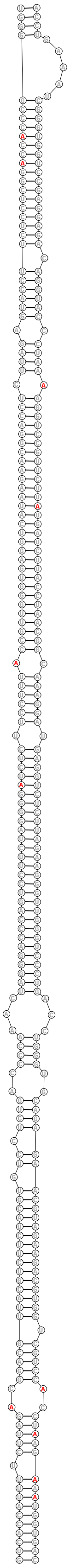

Strands Chr3:22489486-22489638
and Chr3:22492518-22492674

CHDH

Strands Chr22:47710420-47710493
and Chr22:47711464-47711535

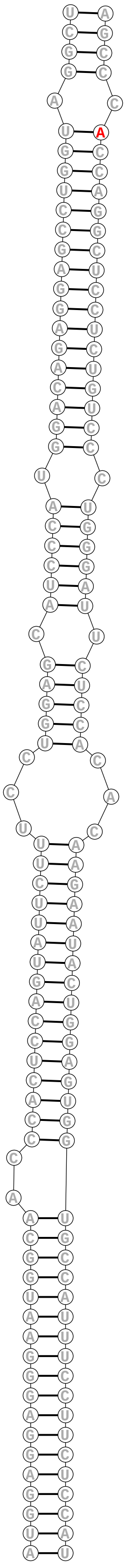

CIDEA

Strands Chr24:43218213-43218341
and Chr24:43219844-43219972

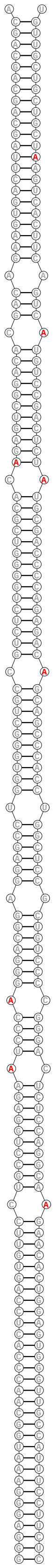

CINP part A

Strands Chr21:68792812-68792916
and Chr21:68794264-68794364

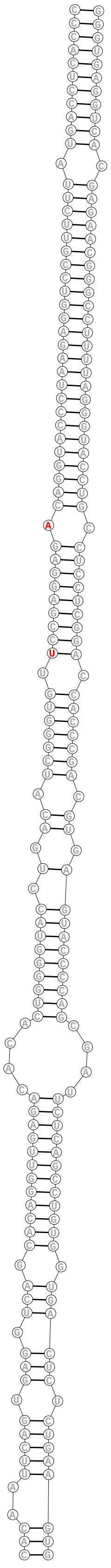

CLMN

Strands Chr21:61619998-61620119
and Chr21:61621054-61621187

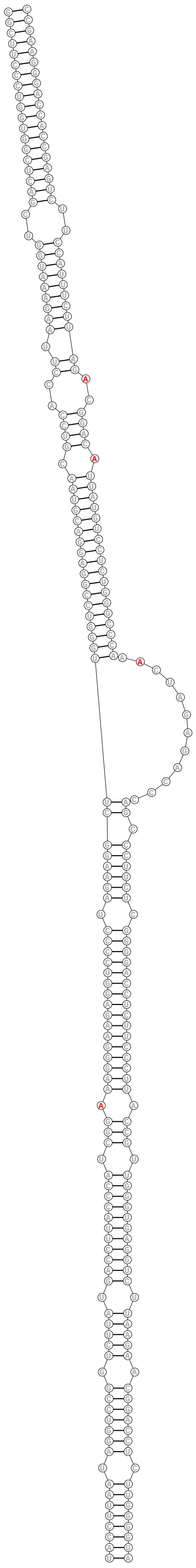

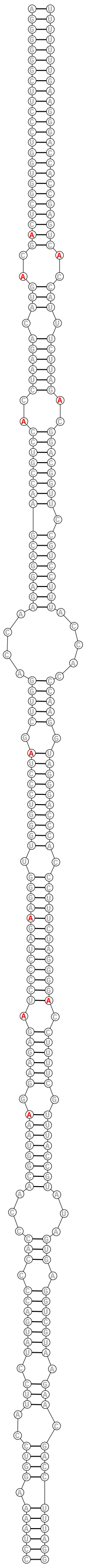

CLN3

Strands Chr25:26311910-26312044
and Chr25:26312351-26312485

CMTM7

Strands Chr22:6939006-6939124
and Chr22:6939290-6939403

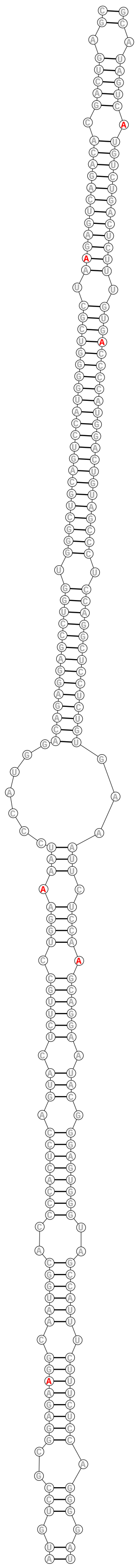

CNOT11

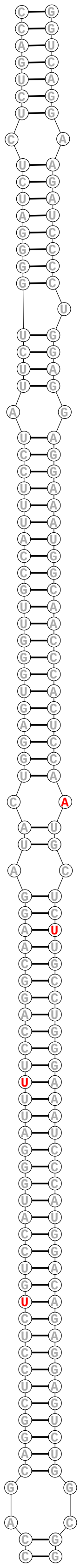

Strands Chr11:6205735-6205819
and Chr11:6206198-6206283

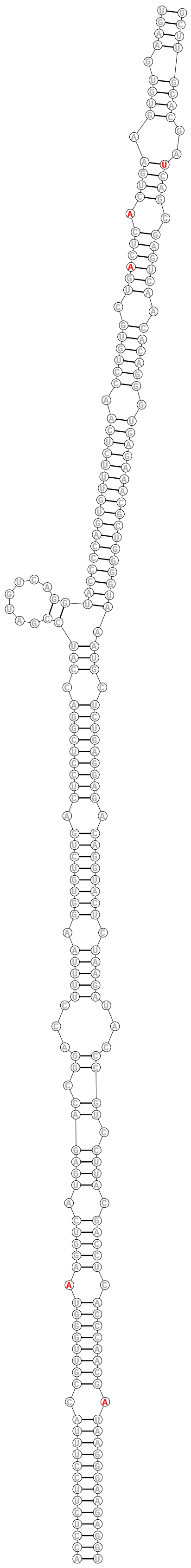

COG5

Strands Chr4:48571952-48572077
and Chr4:48572763-48572878

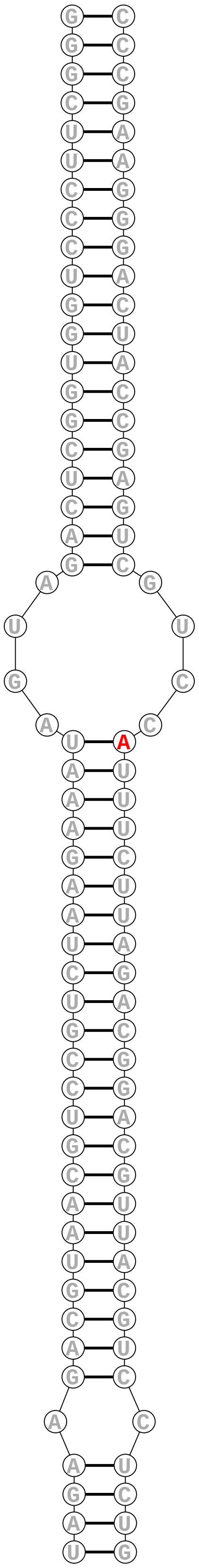

COMMD9

Strands Chr15:67565147-67565198
and Chr15:67565667-67565718

COPS3

Strands Chr19:35494886-35495004
and Chr19:35495817-35495937

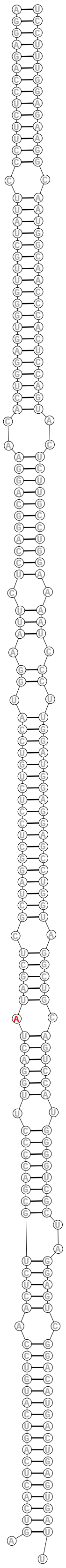

CPNE1

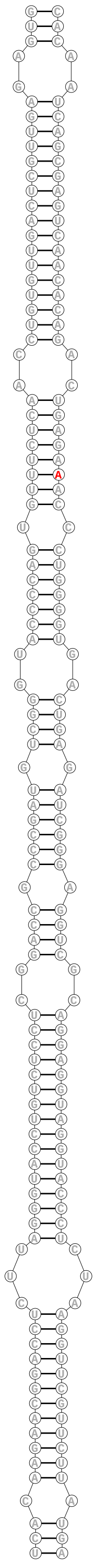

Strands Chr13:65516947-65517041
and Chr13:65517978-65518072

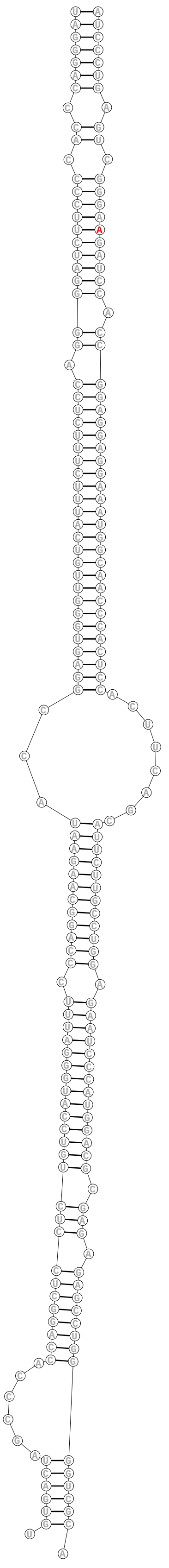

CREB3L1

Strands Chr15:77182297-77182399
and Chr15:77182613-77182716

CRKL

Strands Chr17:74295232-74295310
and Chr17:74295850-74295927

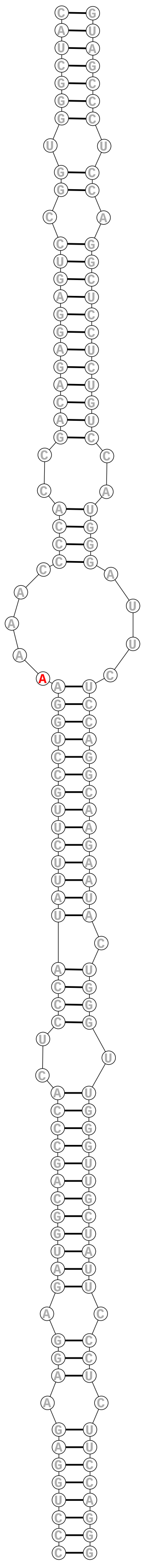

CROCC part A

Strands Chr2:136203532-136203722
and Chr2:136206369-136206561

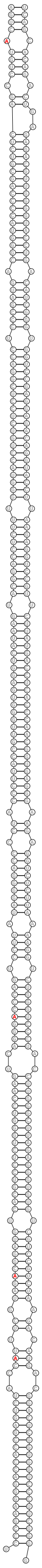

CROCC part B

Strands Chr2:136203973-136204454
and Chr2:136207670-136208134

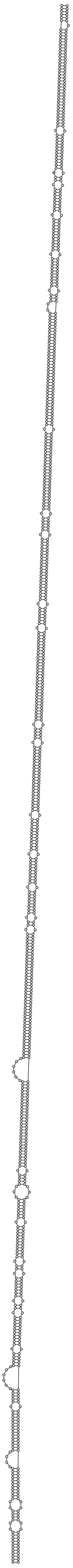

CROCC part C

Strands Chr2:136203513-136203670
and Chr2:136209504-136209670

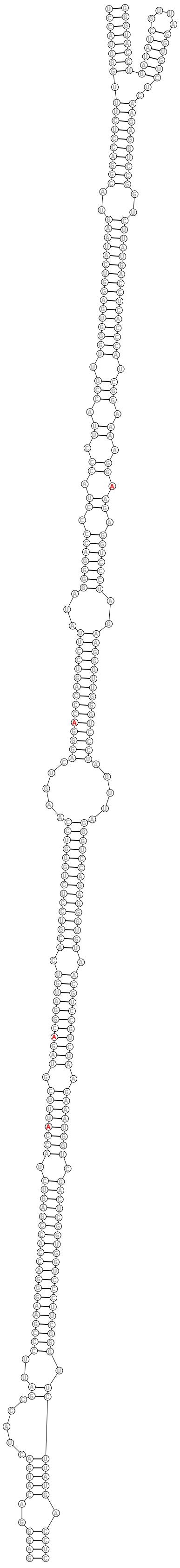

CROCC part D

Strands Chr2:136203533-136203652
and Chr2:136209657-136209776

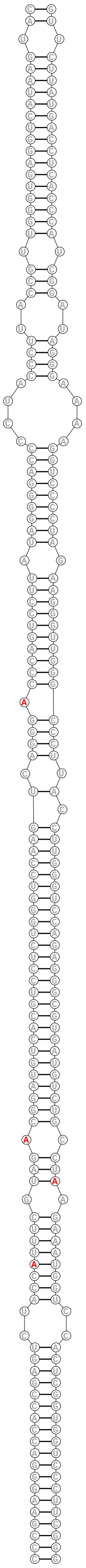

CRYBG3

Strands Chr1:41822546-41822667
and Chr1:41823244-41823365

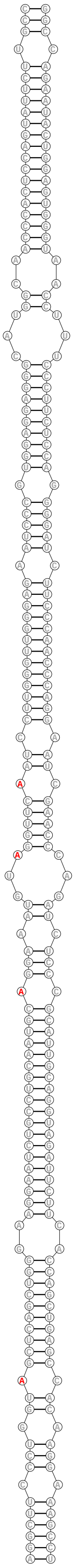

CRYZL1

Strands Chr1:1216883-1216972
and Chr1:1216978-1217061

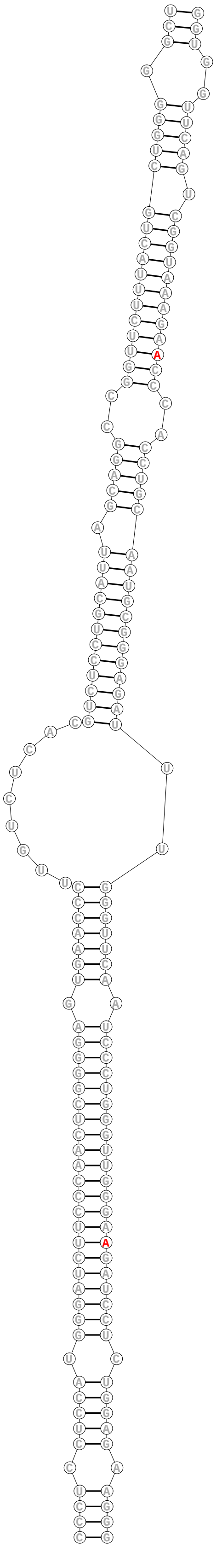

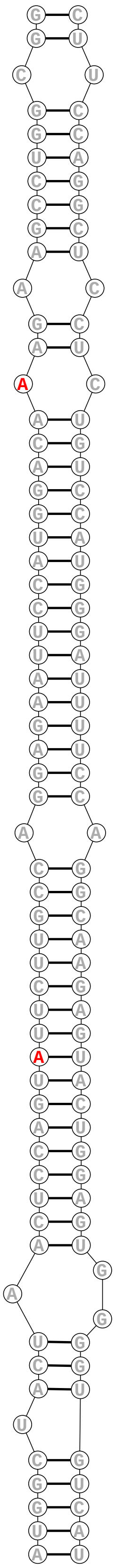

CSF2RB part A

Strands Chr5:75739061-75739119
and Chr5:75739571-75739629

CSF2RB part B

Strands Chr5:75739108-75739165
and Chr5:75739381-75739438

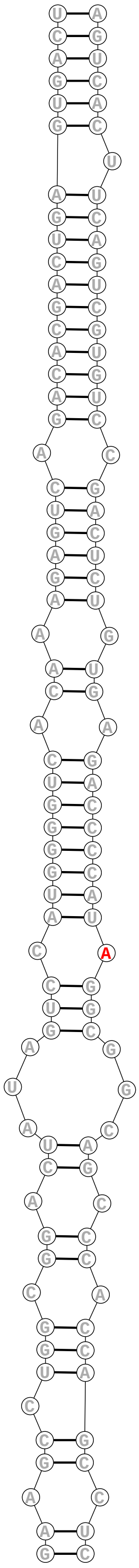

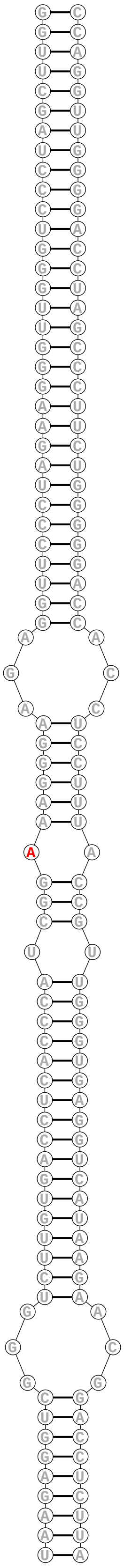

CSK

Strands Chr21:34274615-34274689
and Chr21:34275281-34275355

CSN1S1

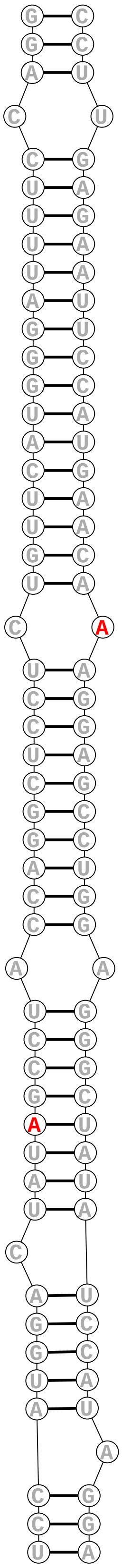

Strands Chr6:87151428-87151476
and Chr6:87151794-87151842

CSN2

Strands Chr6:87183834-87184101
and Chr6:87184721-87184987

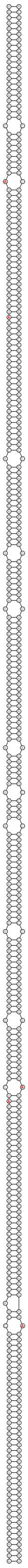

CSN3

Strands Chr6:87381295-87381518
and Chr6:87381703-87381936

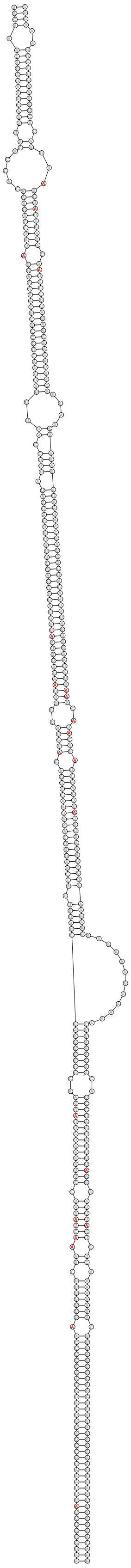

CWF19L1

Strands Chr26:21012089-21012152
and Chr26:21012940-21013007

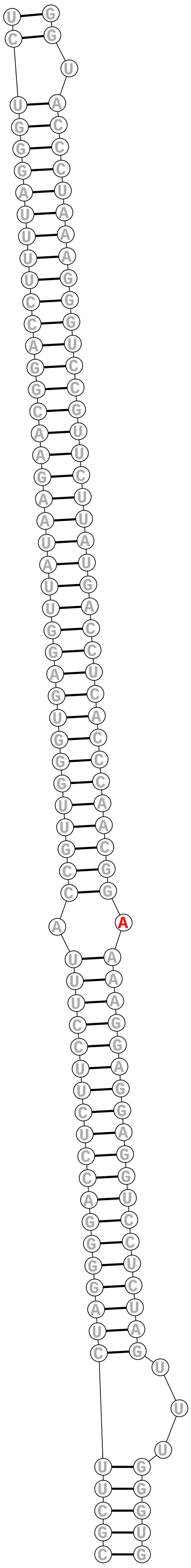

CYP11A1

Strands Chr21:34730622-34730675
and Chr21:34731420-34731473

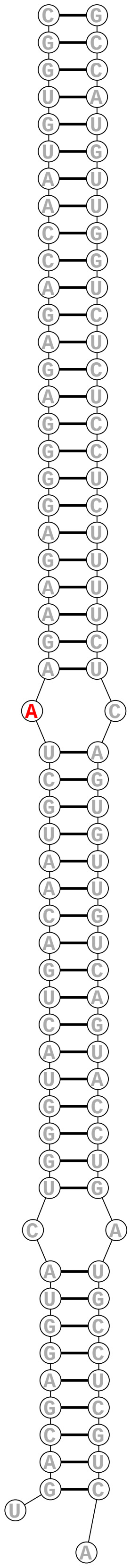

CYP27A1 part A

Strands Chr2:107500071-107500240
and Chr2:107500820-107500991

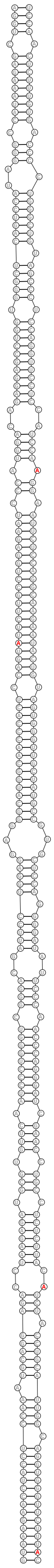

CYP27A1 part B

Strands Chr2:107500824-107501015
and Chr2:107501703-107501894

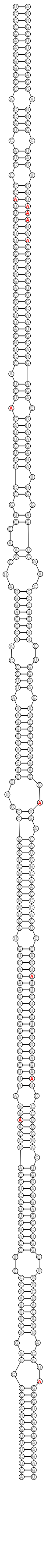

D2HGDH

Strands Chr3:121212877-121213053
and Chr3:121213141-121213334

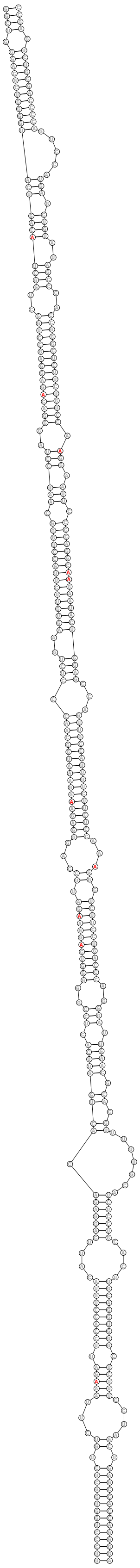

DAP

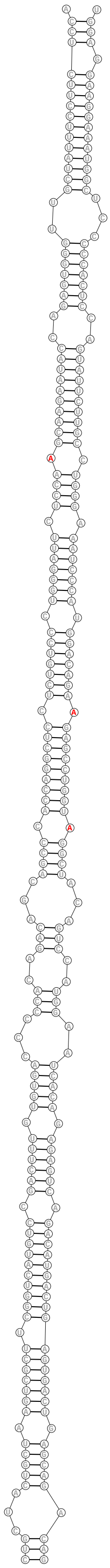

Strands Chr20:62690128-62690257
and Chr20:62695362-62695489

DBP

Strands Chr18:55724913-55725035
and Chr18:55725373-55725496

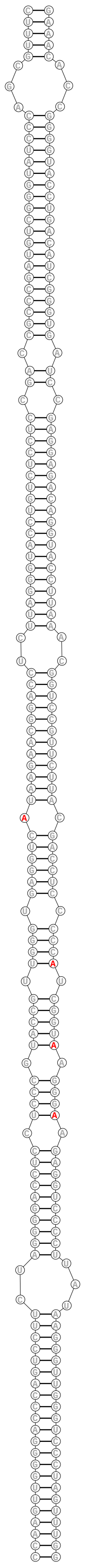

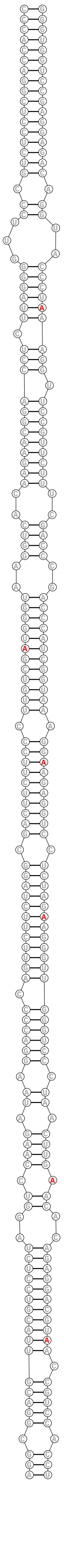

DCP2

Strands Chr10:930136-930261
and Chr10:930723-930847

DCPS part A

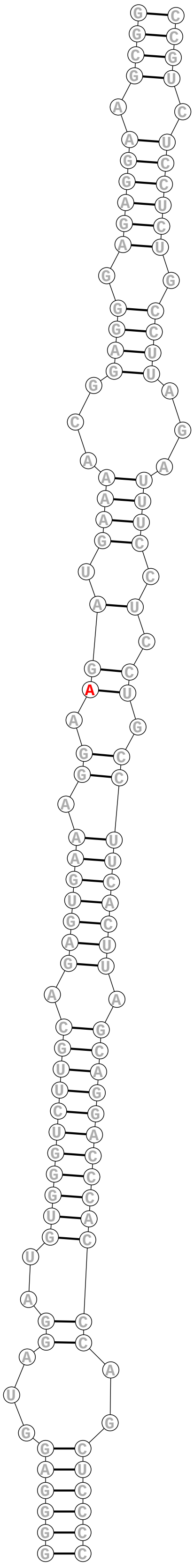

Strands Chr29:30064590-30064652
and Chr29:30065018-30065077

DCPS part B

Strands Chr29:30064515-30064558
and Chr29:30065119-30065158

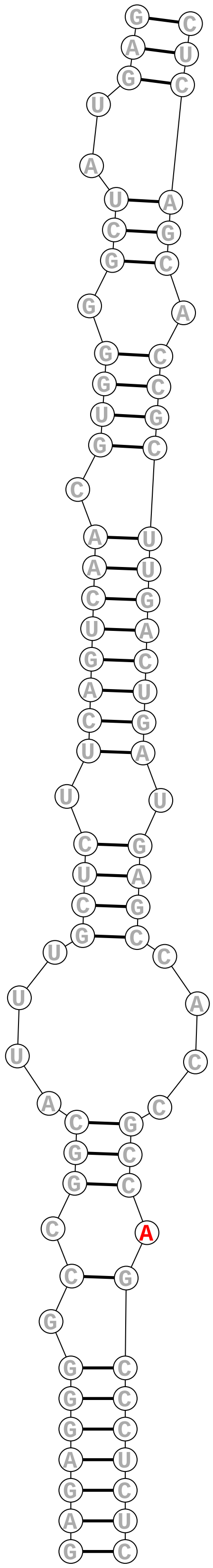

DDX11

Strands Chr5:107474199-107474383
and Chr5:107475488-107475662

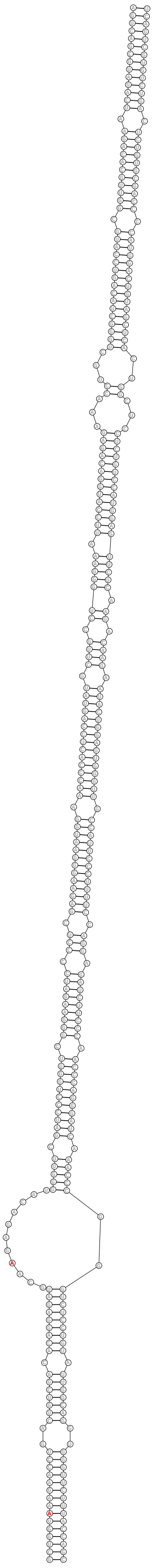

DEXI

Strands Chr25:9668665-9668734
and Chr25:9668993-9669065

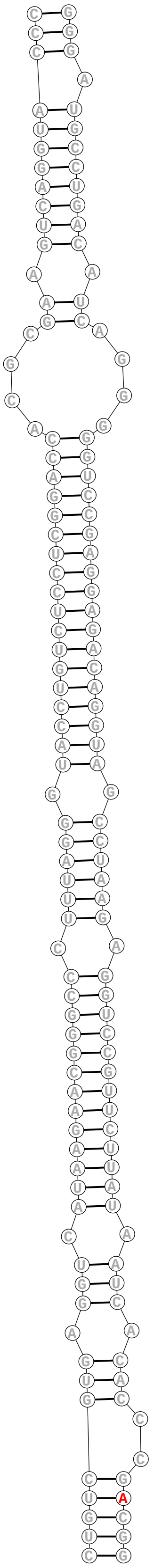

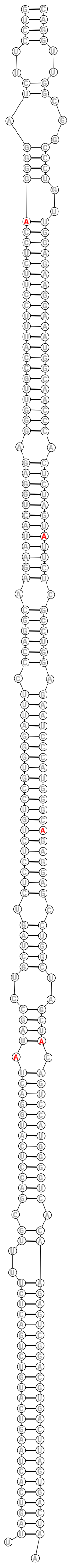

DHRS1 part A

Strands Chr10:20708944-20709075
and Chr10:20711422-20711555

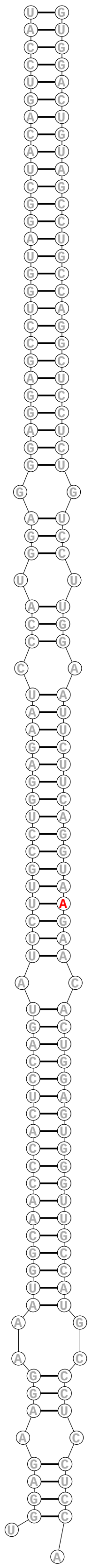

DHRS1 part B

Strands Chr10:20709991-20710072
and Chr10:20710277-20710358

DHTKD1

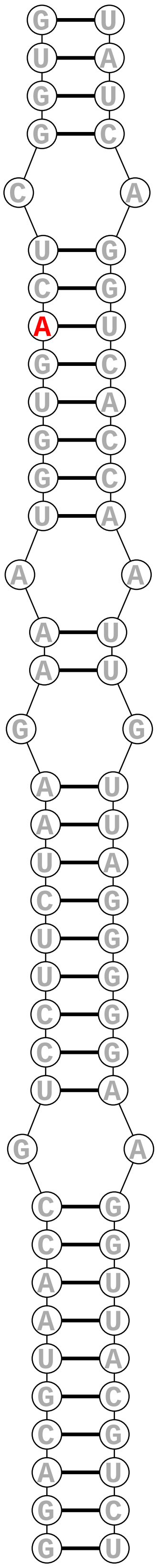

Strands Chr13:12303413-12303449
and Chr13:12304576-12304612

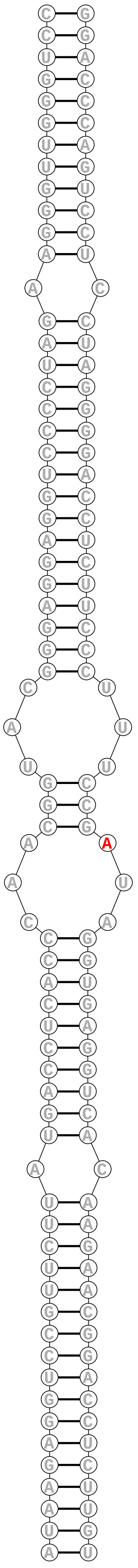

DHX58

Strands Chr19:42880511-42880577
and Chr19:42881169-42881235

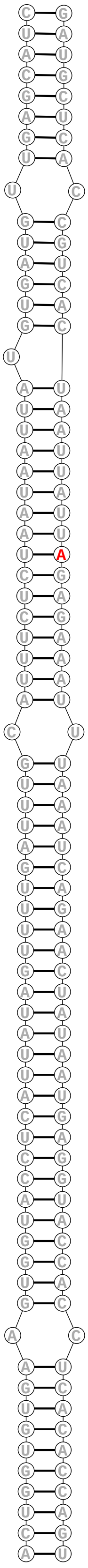

DIABLO

Strands Chr17:55414718-55414788
and Chr17:55415377-55415446

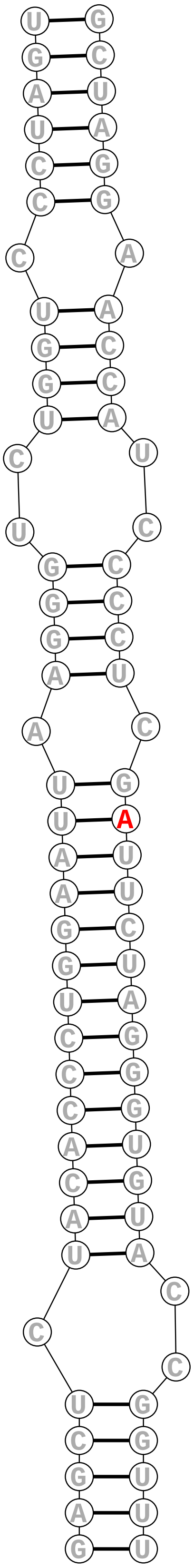

DNAJC12 part A

Strands Chr28:24401461-24401498
and Chr28:24402263-24402301

DNAJC12 part B

Strands Chr28:24414121-24414291
and Chr28:24415004-24415174

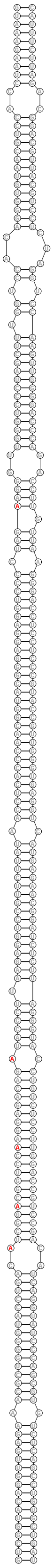

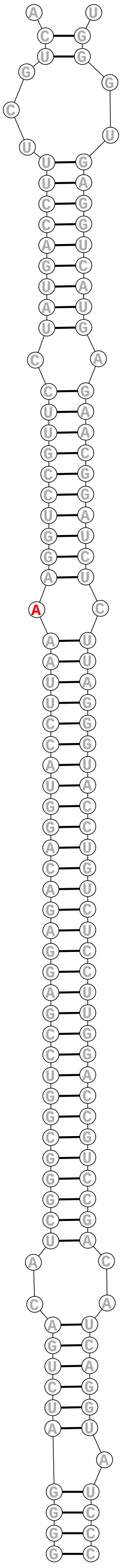

DNAJC12 part C

Strands Chr28:24414937-24415005
and Chr28:24415083-24415151

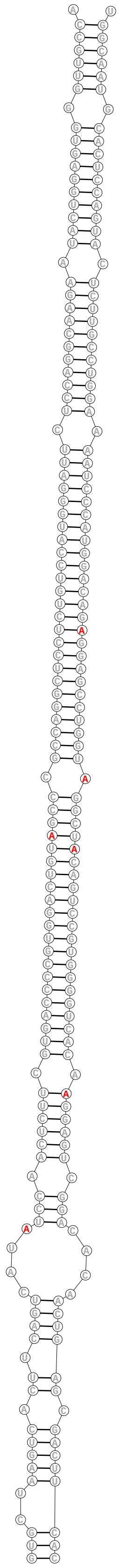

DNTTIP1 part A

Strands Chr13:75286696-75286806
and Chr13:75287866-75287973

DNTTIP1 part B

Strands Chr13:75287063-75287147
and Chr13:75287874-75287961

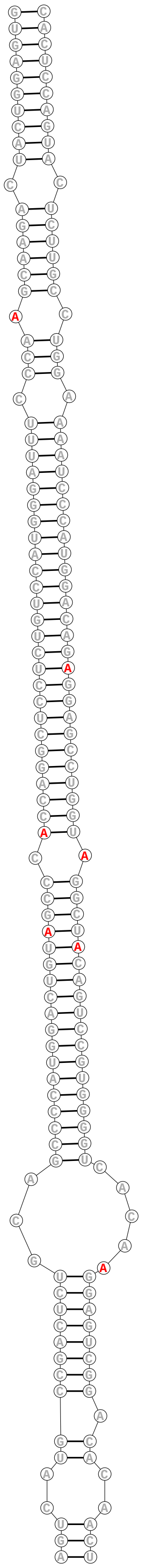

DNTTIP1 part C

Strands Chr13:75287877-75287967
and Chr13:75292611-75292693

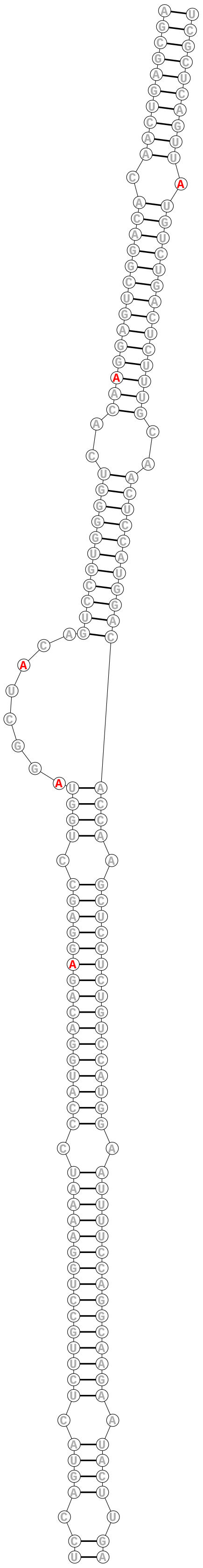

DONSON

Strands Chr1:1230423-1230629
and Chr1:1230842-1231049

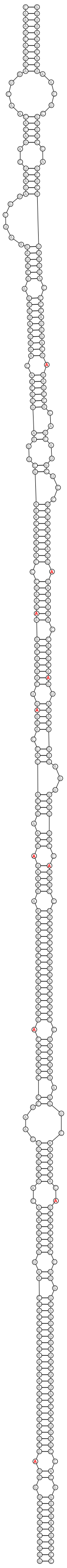

DPH7

Strands Chr11:105587582-105587681
and Chr11:105587878-105587977

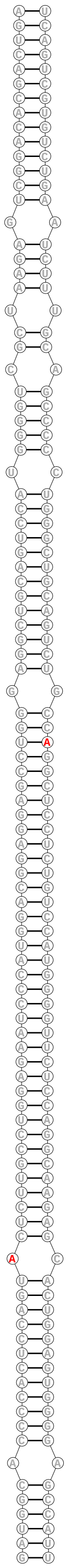

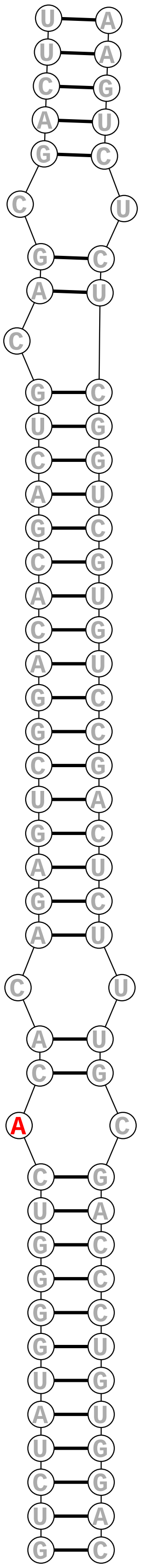

DTNB

Strands Chr11:73878532-73878573
and Chr11:73878627-73878667

DTX4

Strands Chr15:83637870-83637902
and Chr15:83639339-83639372

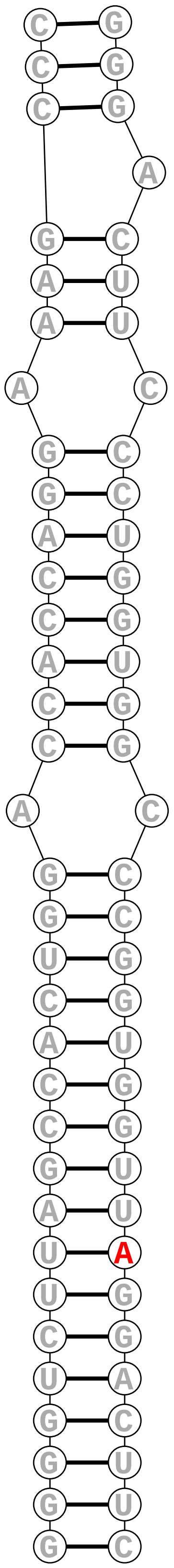

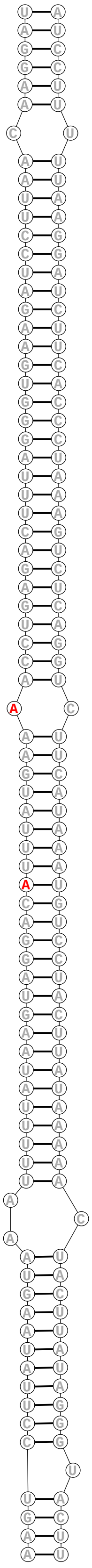

DUSP19

Strands Chr2:13514106-13514184
and Chr2:13514309-13514387

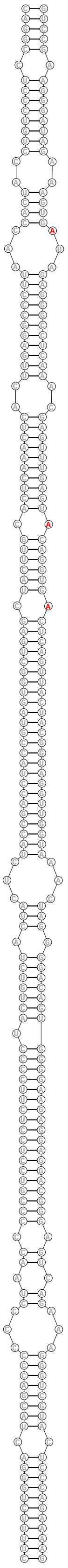

EAPP

Strands Chr21:45354423-45354562
and Chr21:45355922-45356060

ECHDC3 part A

Strands Chr13:12578396-12578482
and Chr13:12579207-12579292

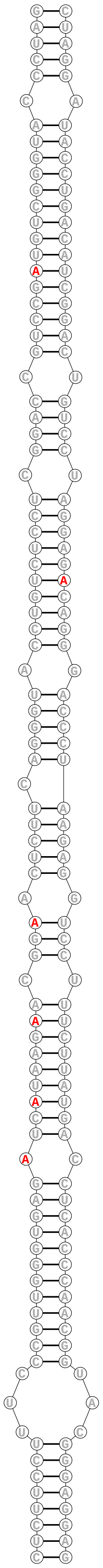

ECHDC3 part B

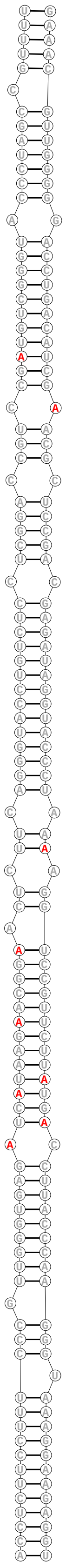

Strands Chr13:12578394-12578489
and Chr13:12583029-12583122

EFHD1 part A

Strands Chr3:113032085-113032198
and Chr3:113033385-113033499

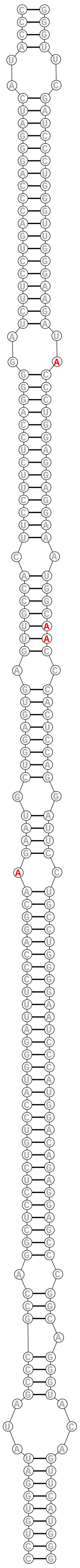

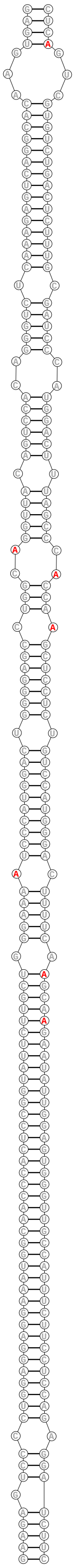

EFHD1 part B

Strands Chr3:113035435-113035556
and Chr3:113036547-113036667

EFHD1 part C

Strands Chr3:113035435-113035556
and Chr3:113036547-113036667

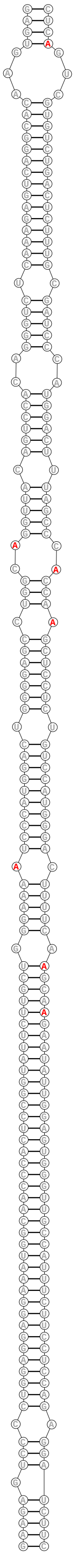

EFHD1 part D

Strands Chr3:113035434-113035573
and Chr3:113037527-113037673

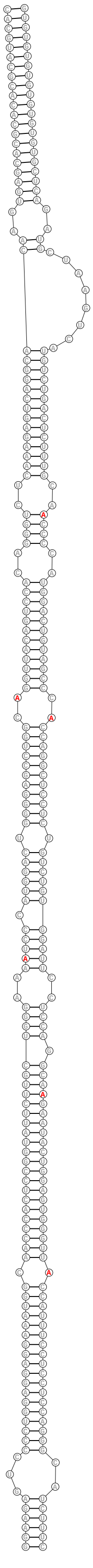

EFHD1 part E

Strands Chr3:113046323-113046440
and Chr3:113047820-113047936

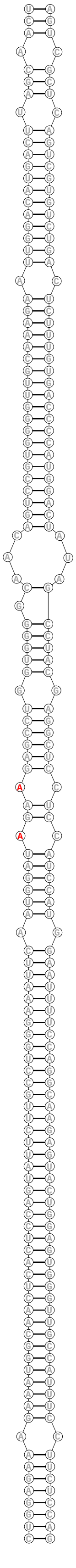

EHD2

Strands Chr18:55081274-55081329
and Chr18:55082164-55082219

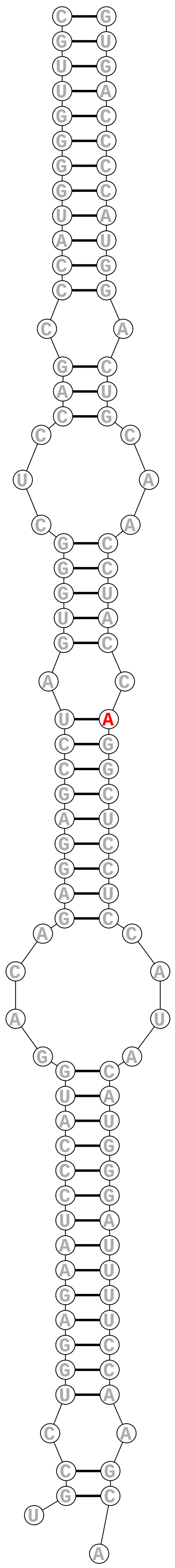

EIF2AK2 part A

Strands Chr11:19587066-19587291
and Chr11:19588194-19588417

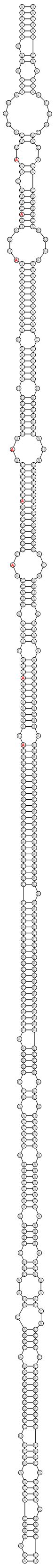

EIF2AK2 part B

Strands Chr11:19587154-19587234
and Chr11:19589578-19589659

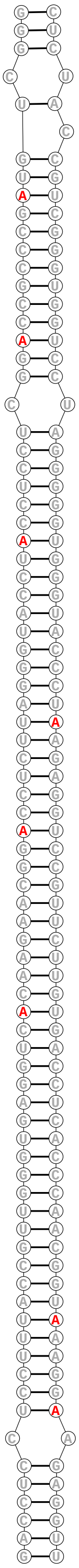

EIF2AK4

Strands Chr10:35782597-35782823
and Chr10:35782900-35783115

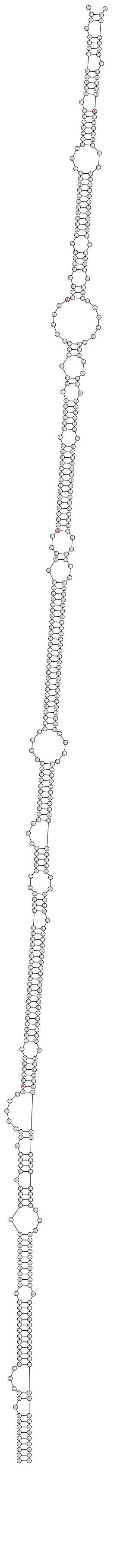

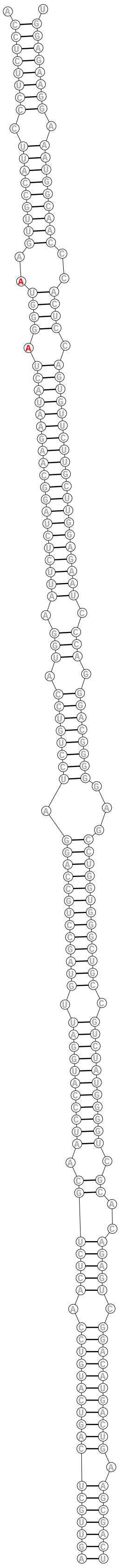

EIF3M part A

Strands Chr15:64100907-64101018
and Chr15:64101320-64101436

EIF3M part B

Strands Chr15:64100017-64100182
and Chr15:64102457-64102624

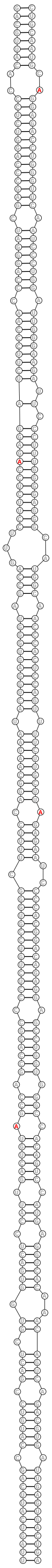

EIF3M part C

Strands Chr15:64100905-64101067
and Chr15:64103539-64103714

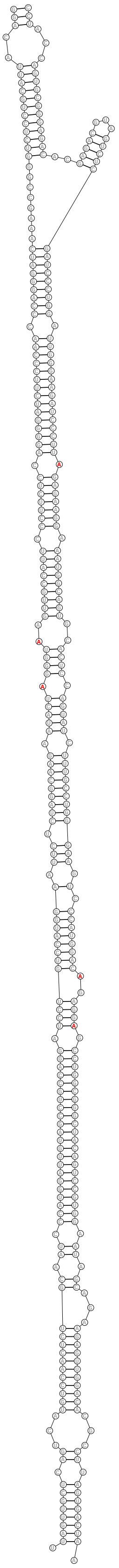

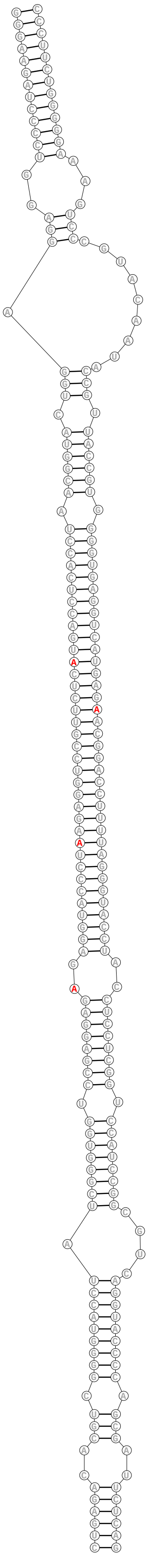

ELF5 part A

Strands Chr15:65836402-65836508
and Chr15:65836906-65837024

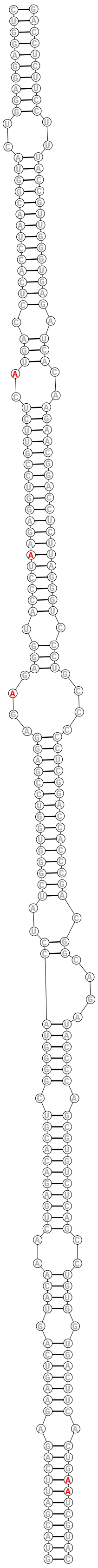

ELF5 part B

Strands Chr15:65836376-65836497
and Chr15:65837069-65837193

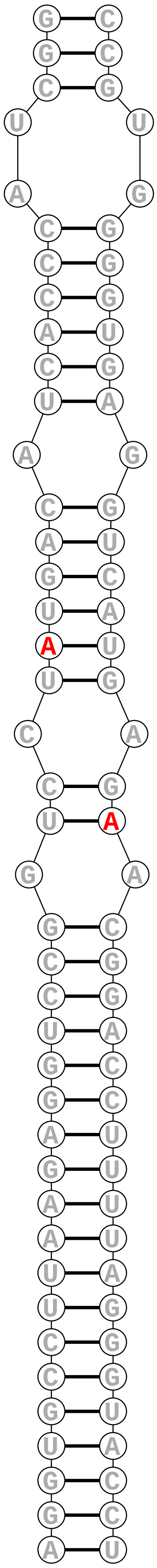

ELF5 part C

Strands Chr15:65836658-65836698
and Chr15:65836940-65836980

ELF5 part D

Strands Chr15:65842306-65842378
and Chr15:65843628-65843700

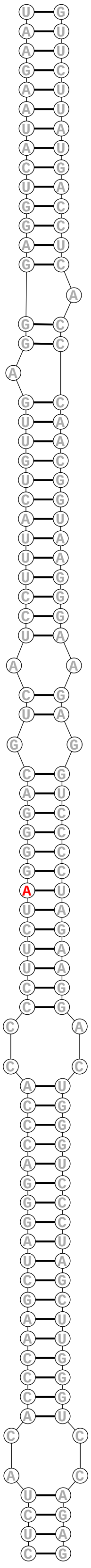

ELMOD3 part A

Strands Chr11:49444140-49444359
and Chr11:49444966-49445159

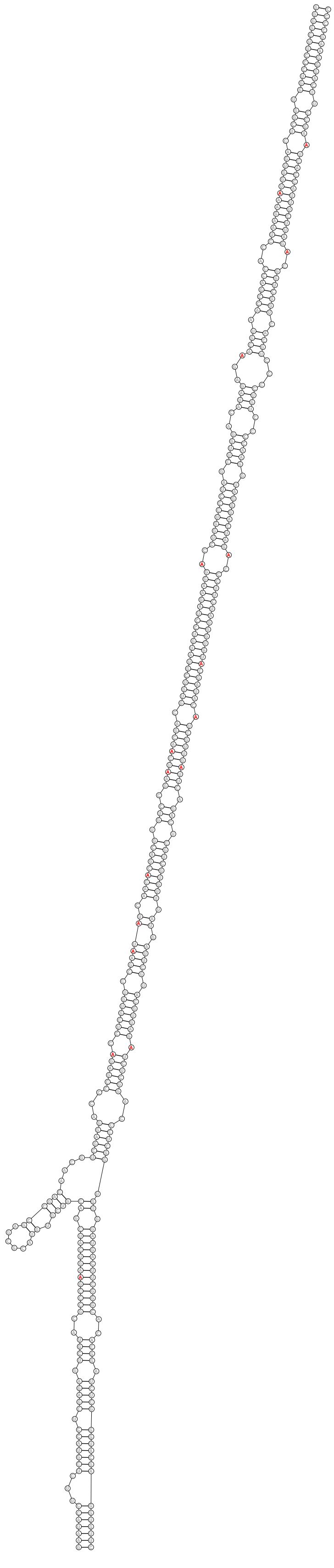

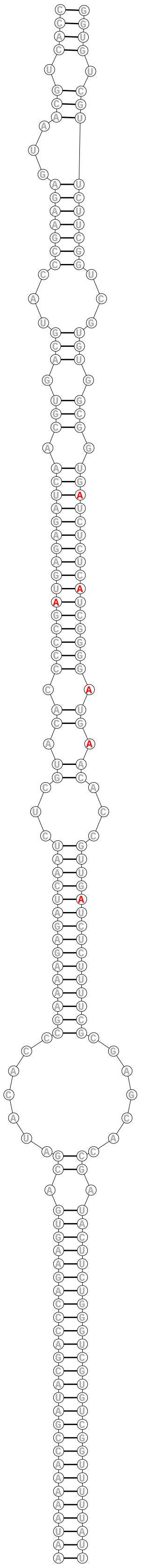

ELMOD3 part B

Strands Chr11:49444034-49444139
and Chr11:49445369-49445471

ELMOD3 part C

Strands Chr11:49445161-49445369
and Chr11:49445727-49445949

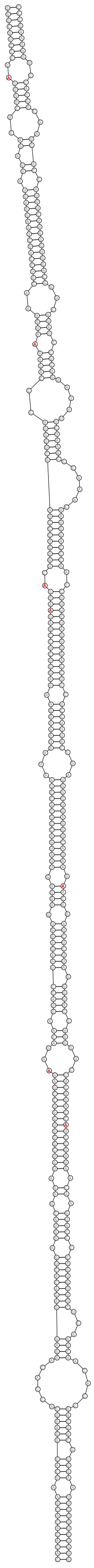

ELMOD3 part D

Strands Chr11:49445773-49445948
and Chr11:49448539-49448717

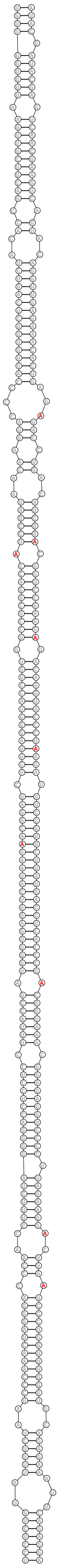

ELMOD3 part E

Strands Chr11:49445726-49445953
and Chr11:49449336-49449562

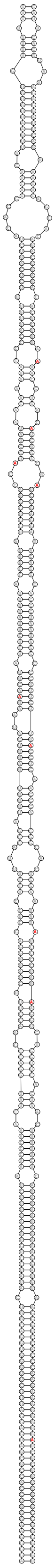

ELMOD3 part F

Strands Chr11:49449347-49449559
and Chr11:49450768-49450982

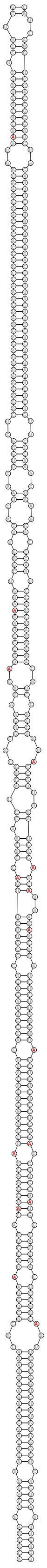

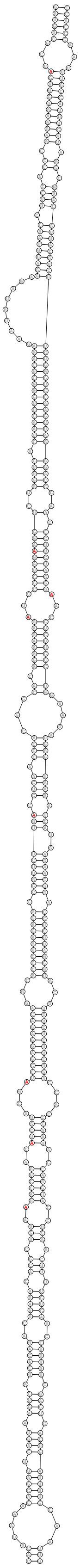

ELMOD3 part G

Strands Chr11:49449340-49449551
and Chr11:49452079-49452279

ENO4 part A

Strands Chr26:37582666-37582771
and Chr26:37585951-37586055

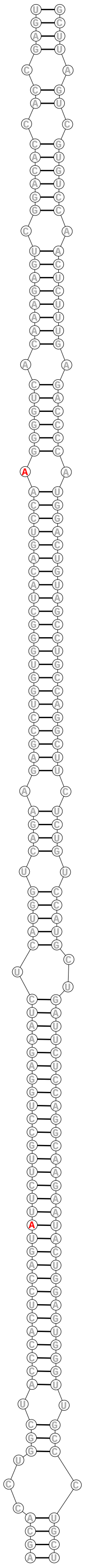

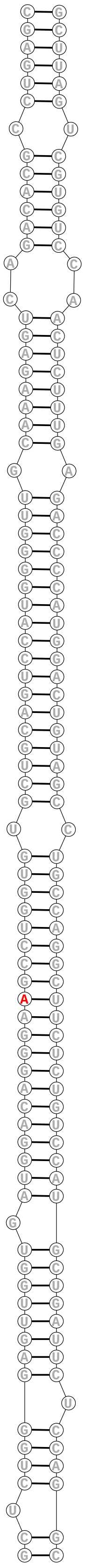

ENO4 part B

Strands Chr26:37584612-37584690
and Chr26:37585951-37586028

ENOPH1 part A

Strands Chr6:99016552-99016695
and Chr6:99016992-99017133

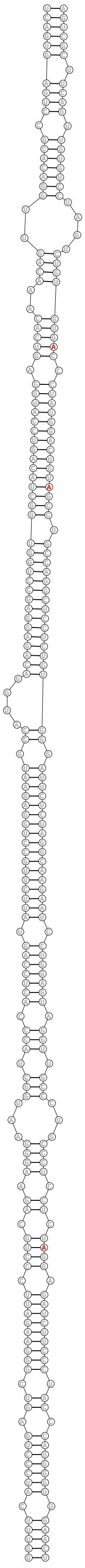

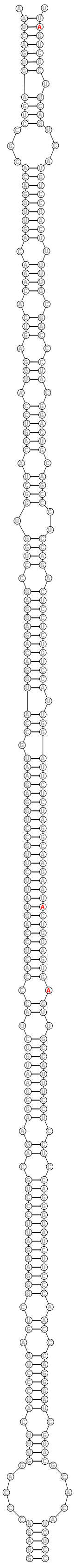

ENOPH1 part B

Strands Chr6:99016543-99016697
and Chr6:99027056-99027212

ENSBTAG00000000144

Strands Chr4:83311122-83311165
and Chr4:83311750-83311793

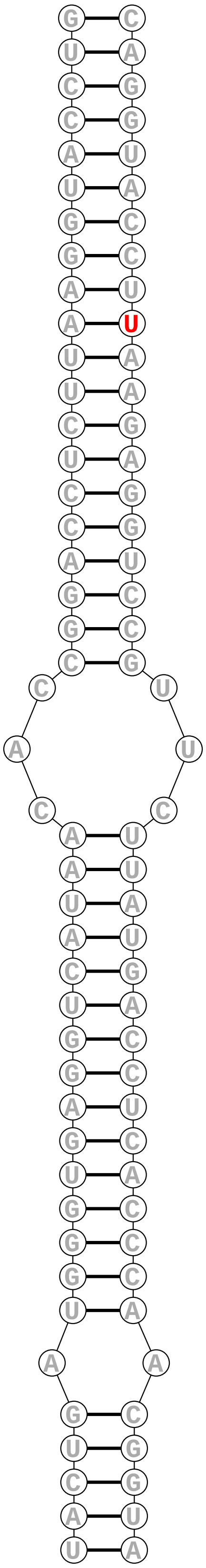

ENSBTAG00000000269 part A

Strands Chr8:44593972-44594123
and Chr8:44594769-44594921

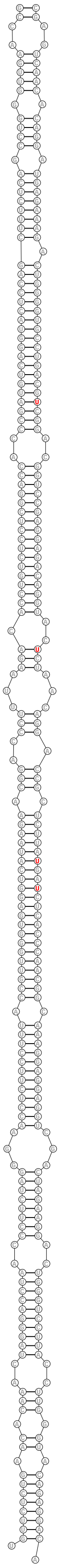

ENSBTAG00000000269 part B

Strands Chr8:44594829-44594892
and Chr8:44595067-44595130

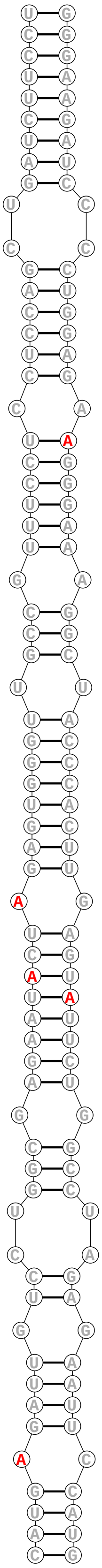

ENSBTAG00000000269 part C

Strands Chr8:44594767-44594873
and Chr8:44595461-44595568

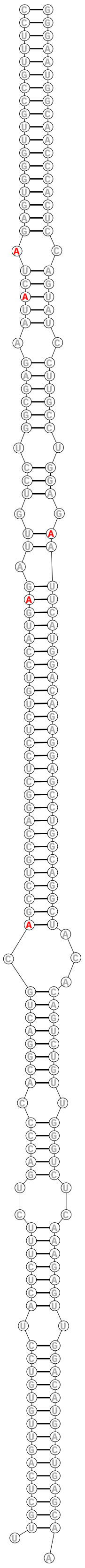

ENSBTAG00000002605 part A

Strands Chr19:33632382-33632414
and Chr19:33632430-33632462

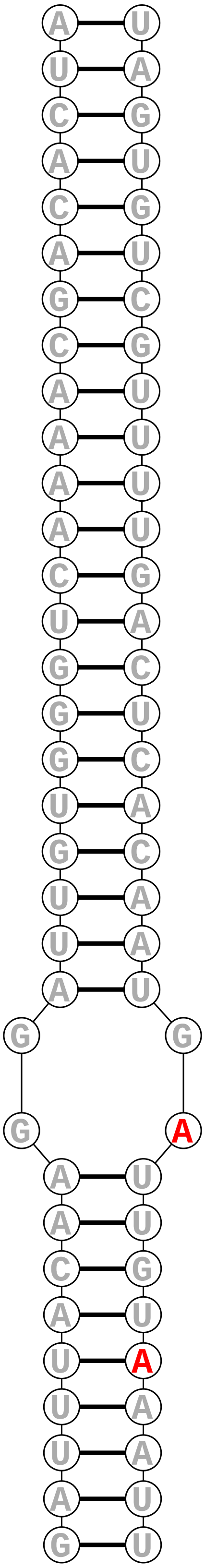

ENSBTAG00000002605 part B

Strands Chr19:33632467-33632591
and Chr19:33632906-33633030

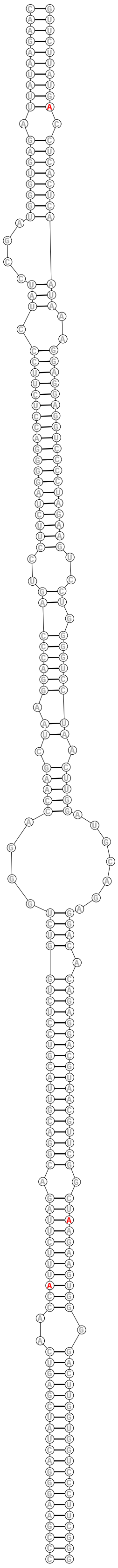

ENSBTAG00000002605 part C

Strands Chr19:33631512-33631623
and Chr19:33632817-33632926

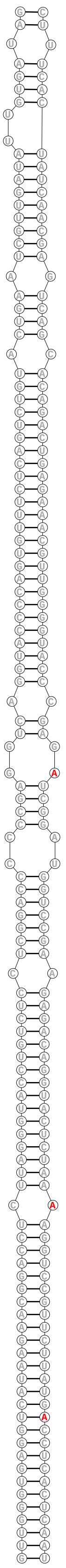

ENSBTAG00000002605 part D

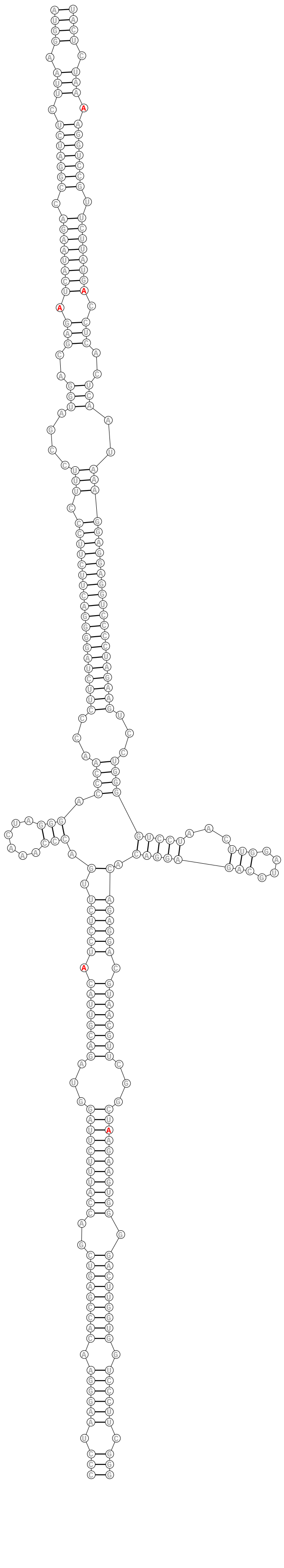

Strands Chr19:33632591-33632725
and Chr19:33632891-33633030

ENSBTAG00000002605 part E

Strands Chr19:33632828-33632992
and Chr19:33634255-33634417

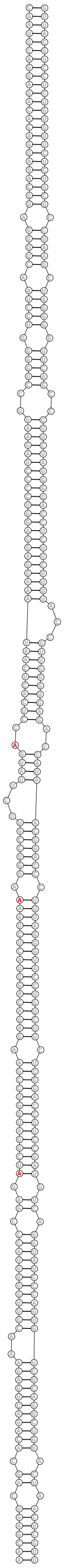

ENSBTAG00000002633

Strands Chr19:55122563-55122658
and Chr19:55123391-55123485

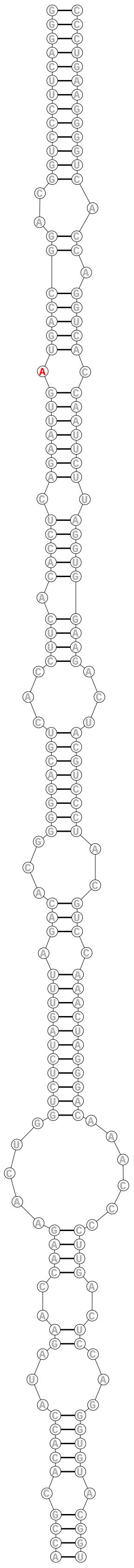

ENSBTAG00000003367 part A

Strands Chr5:114968755-114968875
and Chr5:114969605-114969724

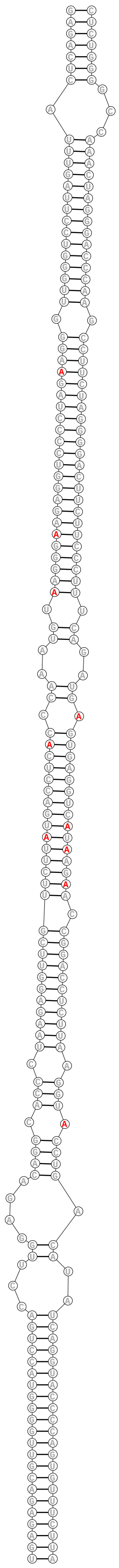

ENSBTAG00000003367 part B

Strands Chr5:114968739-114968847
and Chr5:114970737-114970856

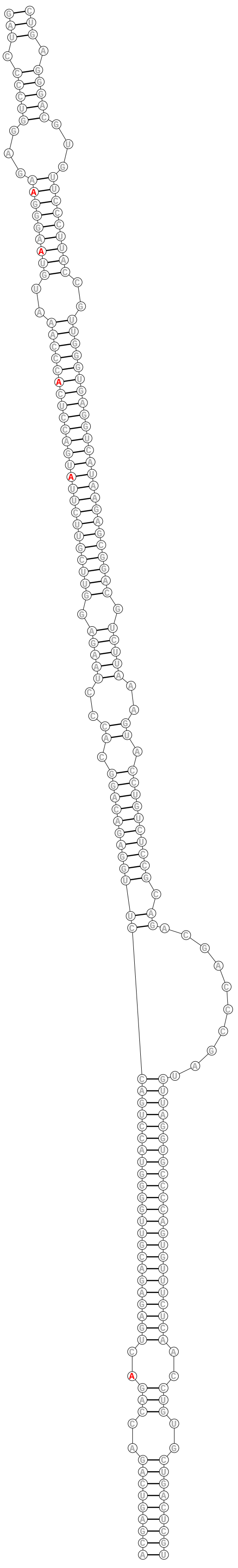

ENSBTAG00000003367 part C

Strands Chr5:114969593-114969748
and Chr5:114970432-114970600

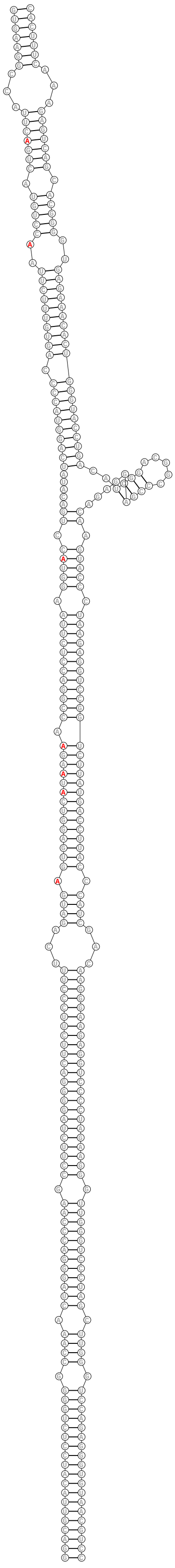

ENSBTAG00000005495

Strands Chr8:77084154-77084356
and Chr8:77086029-77086225

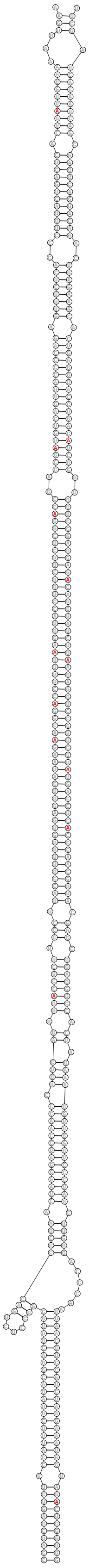

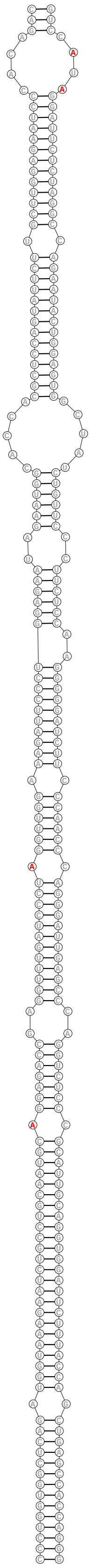

ENSBTAG00000008032 part A

Strands Chr4:115927773-115927905
and Chr4:115928171-115928305

ENSBTAG00000008032 part B

Strands Chr4:115928171-115928297
and Chr4:115929456-115929580

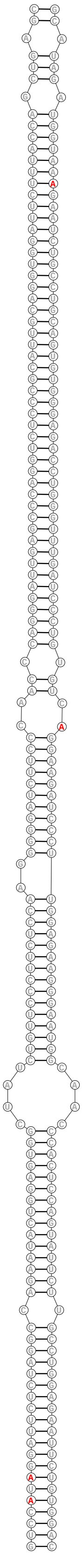

ENSBTAG00000016957

Strands Chr8:112133121-112133249
and Chr8:112133597-112133723

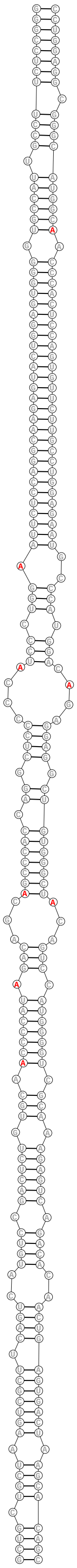

ENSBTAG00000024604

Strands Chr11:94271956-94272021
and Chr11:94272151-94272217

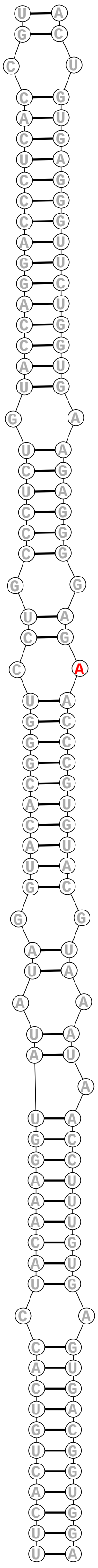

ENSBTAG00000031548

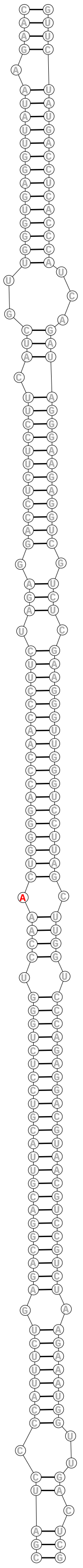

Strands Chr4:99879551-99879654
and Chr4:99881217-99881321

ENSBTAG00000040392 part A

Strands Chr18:61210742-61210936
and Chr18:61211686-61211880

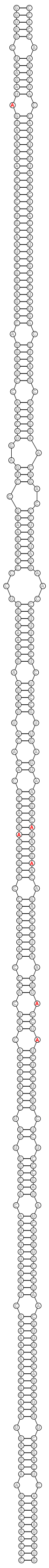

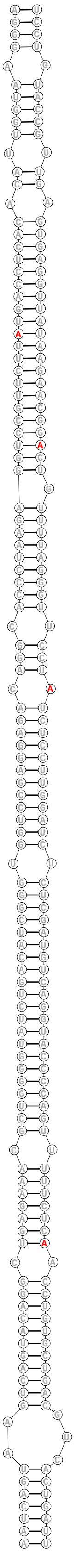

ENSBTAG00000040392 part B

Strands Chr18:61210745-61210856
and Chr18:61212373-61212486

ENSBTAG00000040392 part C

Strands Chr18:61211927-61212045
and Chr18:61212377-61212496

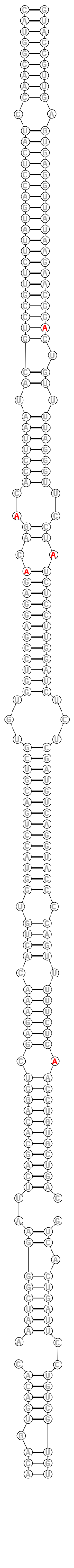

ENSBTAG00000040392 part D

Strands Chr18:61211754-61211867
and Chr18:61211948-61212060

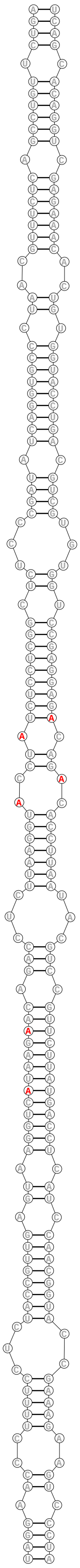

ENSBTAG00000040602

Strands Chr2:131413149-131413195
and Chr2:131413620-131413666

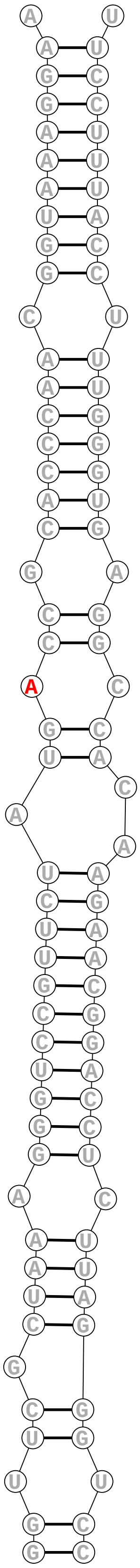

ENSBTAG00000045683

Strands Chr7:54132716-54132752
and Chr7:54132806-54132848

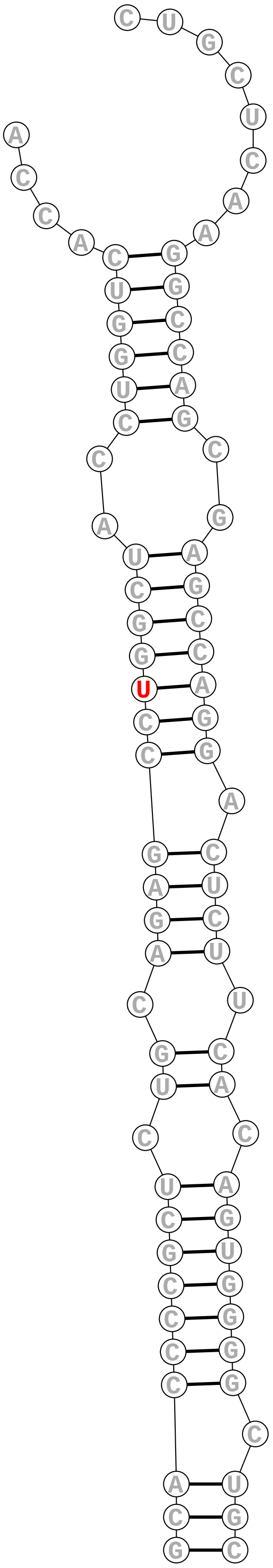

ENSBTAG00000046117

Strands Chr11:63370202-63370271
and Chr11:63370518-63370586

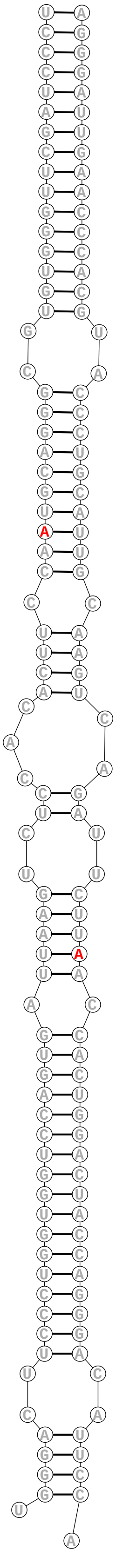

EPB41L4A

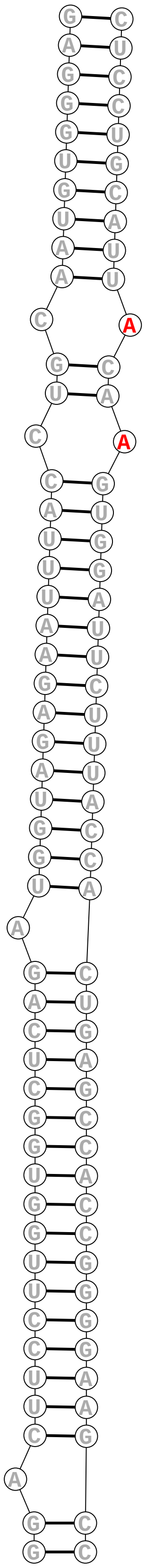

Strands Chr10:1747595-1747644
and Chr10:1748936-1748983

EPHA2 part A

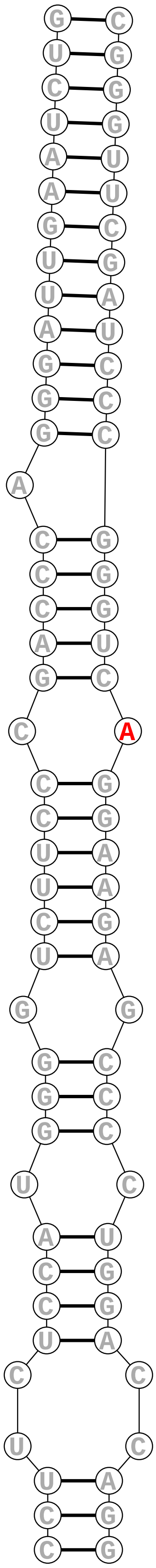

Strands Chr2:136549193-136549232
and Chr2:136550560-136550598

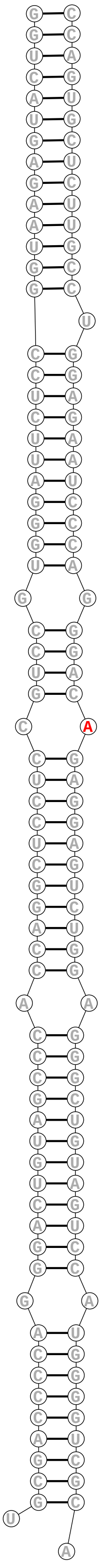

EPHA2 part B

Strands Chr2:136549112-136549177
and Chr2:136550614-136550680

ERH

Strands Chr10:81522298-81522344
and Chr10:81523469-81523515

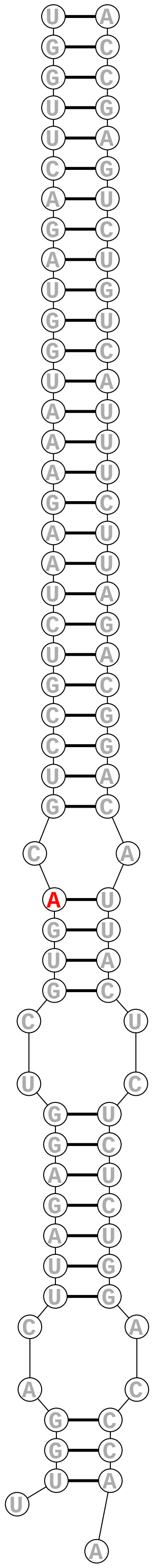

EWSR1

Strands Chr17:70702847-70703000
and Chr17:70703621-70703773

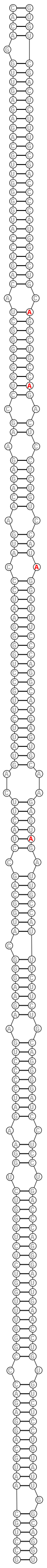

EXOSC1

Strands Chr26:18527438-18527509
and Chr26:18528099-18528171

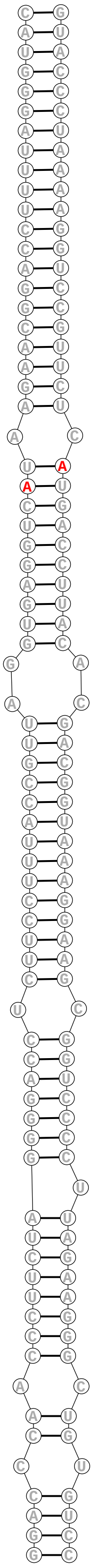

EXOSC7 part A

Strands Chr22:54828424-54828512
and Chr22:54830133-54830220

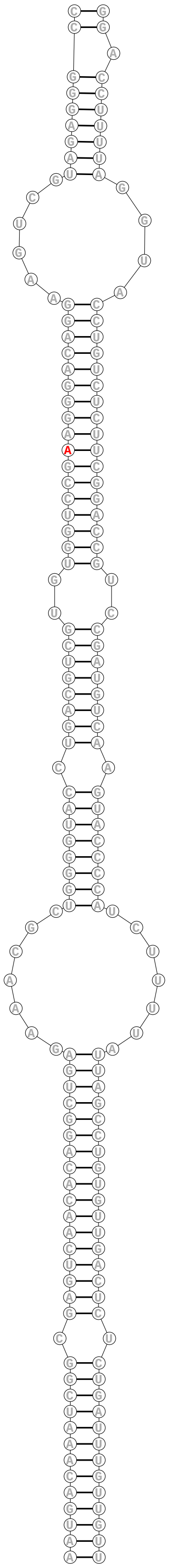

EXOSC7 part B

Strands Chr22:54829747-54829853
and Chr22:54830086-54830186

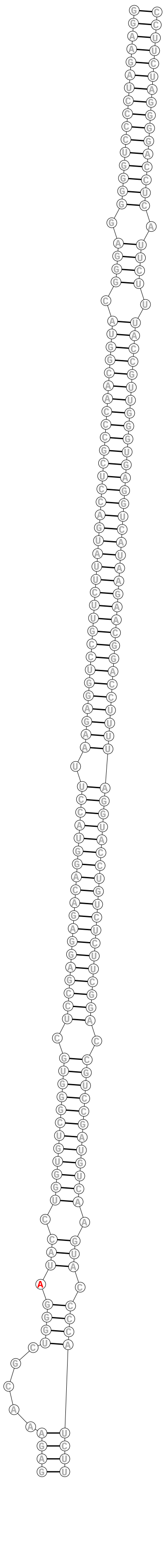

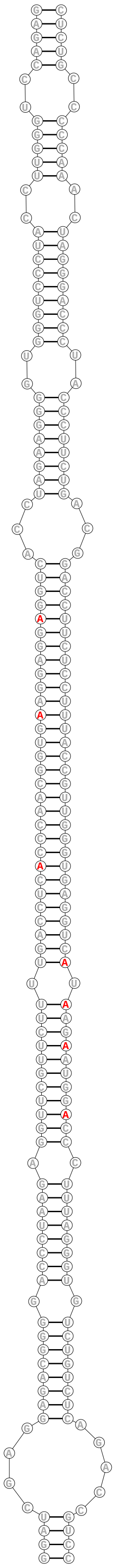

EXOSC9

Strands Chr6:3420505-3420608
and Chr6:3421269-3421373

FAM129B

Strands Chr11:98246723-98246800
and Chr11:98247068-98247145

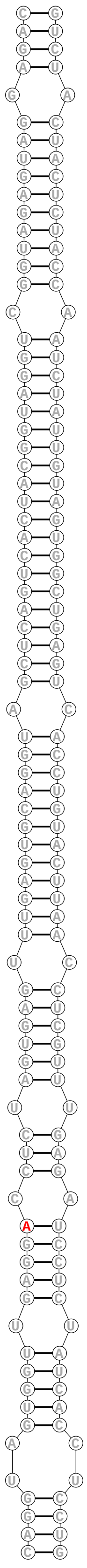

FAM134B

Strands Chr20:56756853-56756938
and Chr20:56757574-56757661

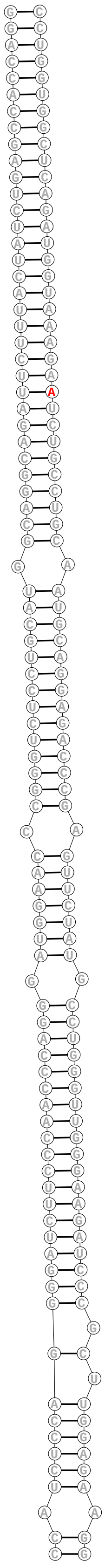

FAM20A part A

Strands Chr19:62272202-62272340
and Chr19:62272521-62272658

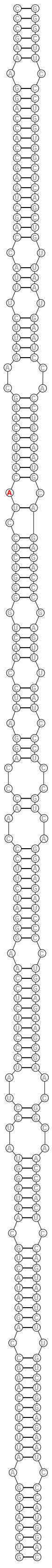

FAM20A part B

Strands Chr19:62287590-62287761
and Chr19:62288276-62288452

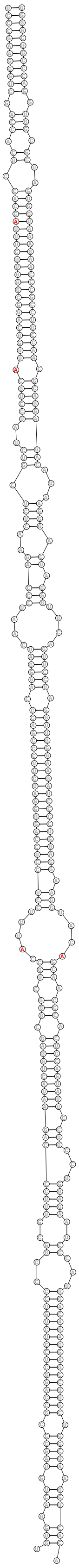

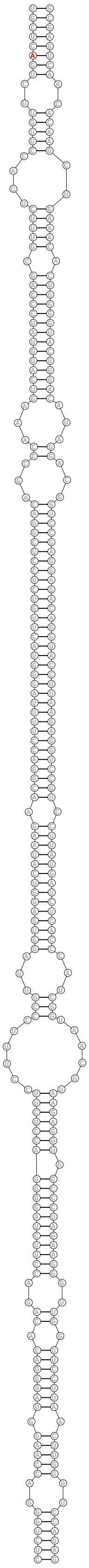

FAM210A

Strands Chr24:43938908-43939057
and Chr24:43939694-43939842

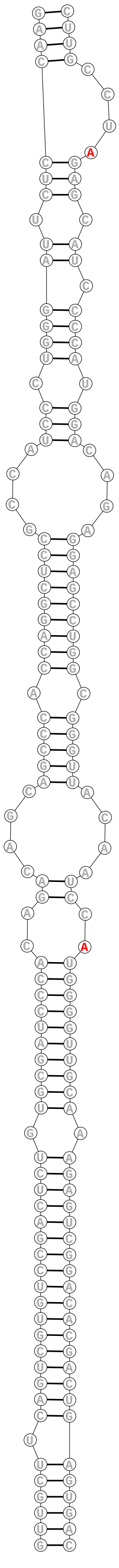

FAM35A

Strands Chr28:42089158-42089237
and Chr28:42089669-42089752

FAM73B part A

Strands Chr11:99536866-99537063
and Chr11:99540179-99540384

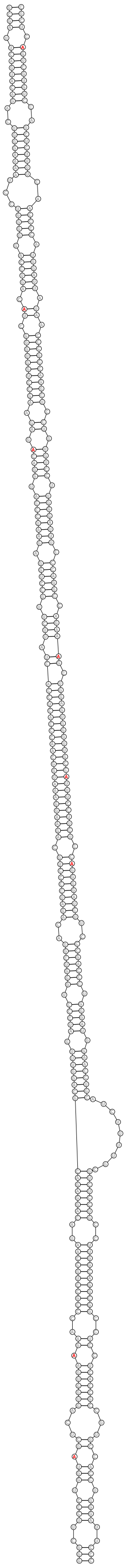

FAM73B part B

Strands Chr11:99536953-99537053
and Chr11:99538925-99539024

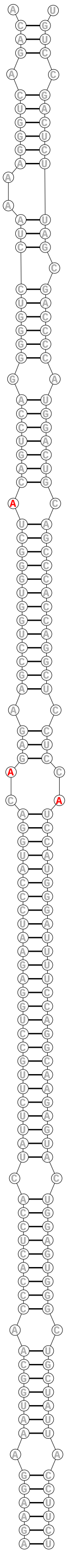

FAM73B part C

Strands Chr11:99538769-99538885
and Chr11:99539830-99539930

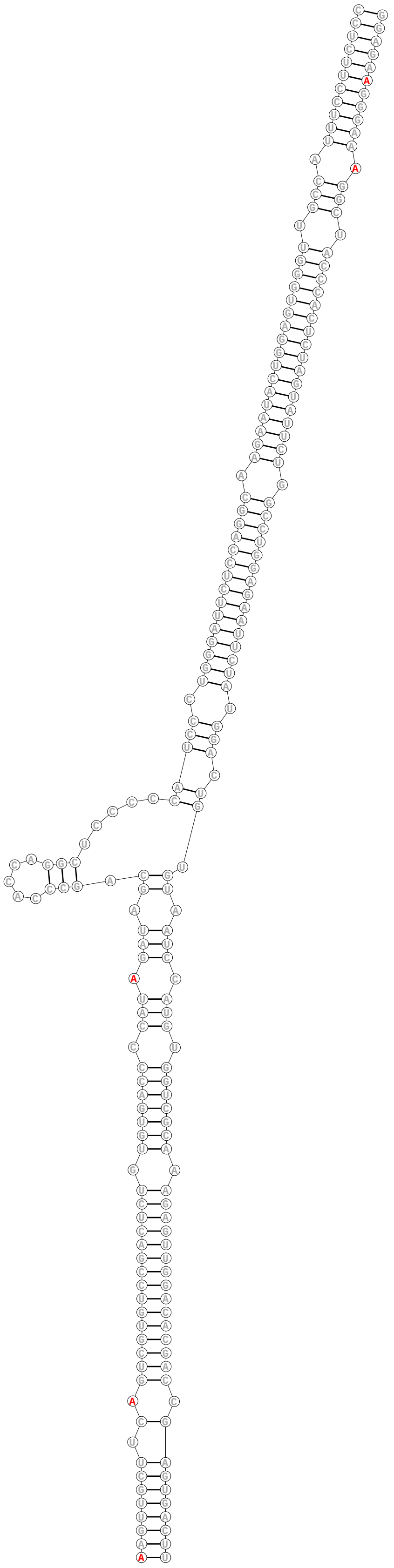

FAM73B part D

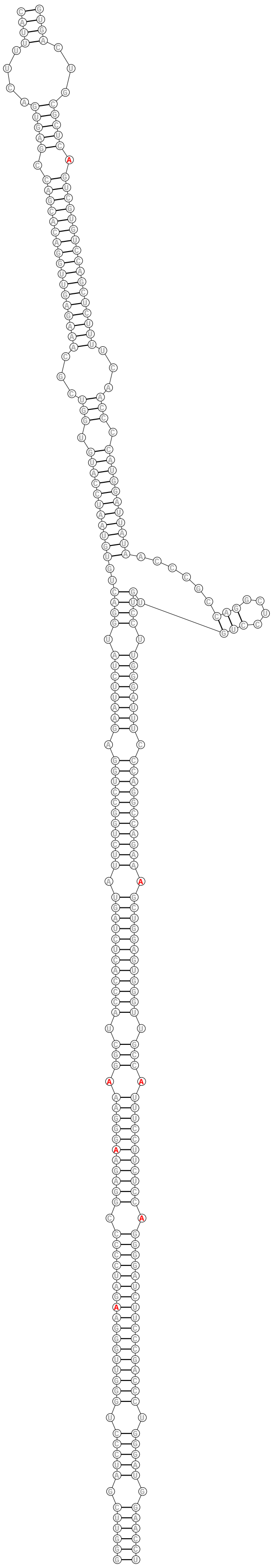

Strands Chr11:99539799-99539934
and Chr11:99540172-99540321

FAM73B part E

Strands Chr11:99537149-99537251
and Chr11:99538007-99538111

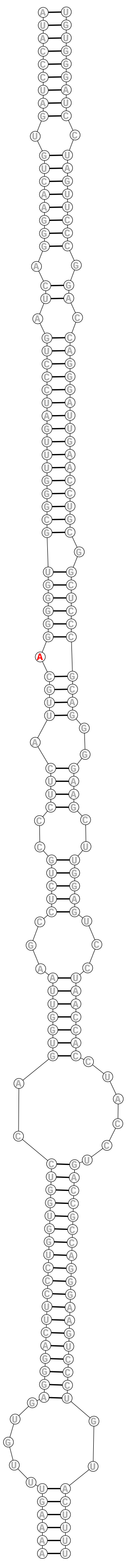

FAM96A

Strands Chr10:45948632-45948733
and Chr10:45949713-45949813

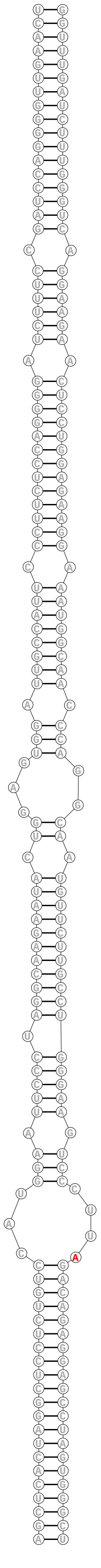

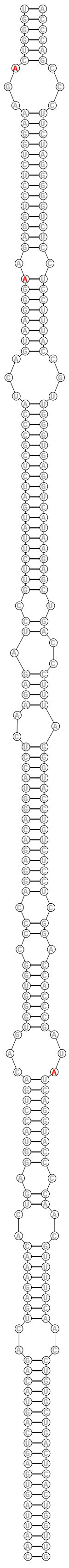

FBXL12

Strands Chr7:15514369-15514505
and Chr7:15516708-15516844

FBXL19

Strands Chr25:27329271-27329390
and Chr25:27331919-27332030

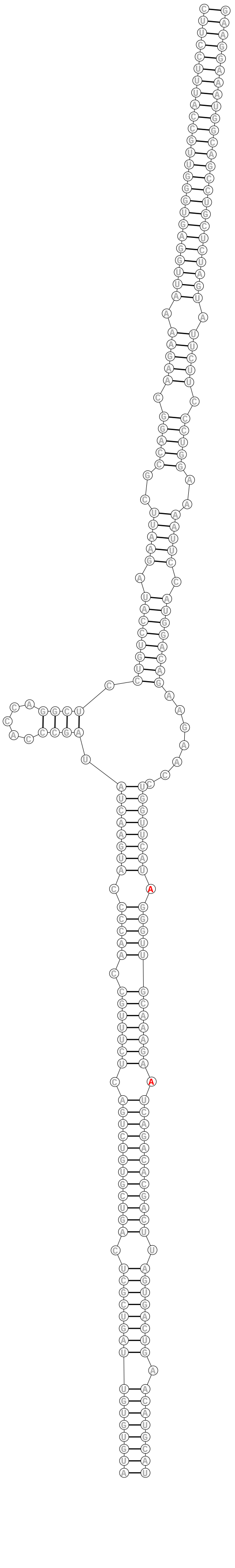

FBXW12 part A

Strands Chr8:91669420-91669531
and Chr8:91669783-91669894

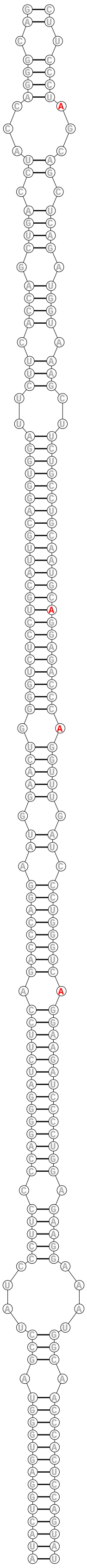

FBXW12 part B

Strands Chr8:91669214-91669422
and Chr8:91670760-91670955

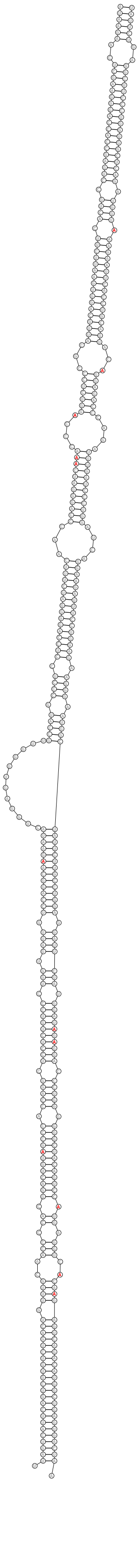

FBXW12 part C

Strands Chr8:91669812-91669861
and Chr8:91670638-91670687

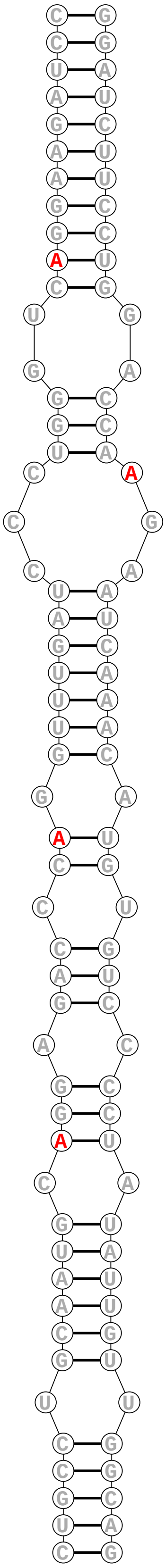

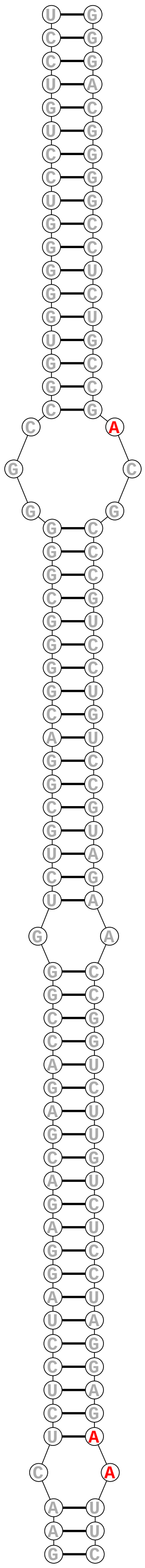

FBXW8

Strands Chr17:60340414-60340477
and Chr17:60340991-60341054

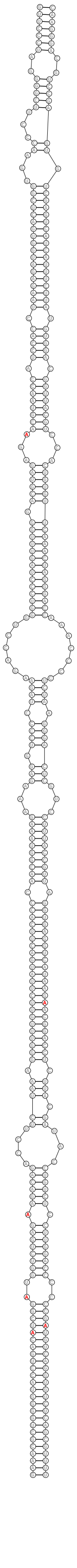

FDFT1

Strands Chr8:7438726-7438913
and Chr8:7440042-7440222

FECH part A

Strands Chr24:57303196-57303364
and Chr24:57304513-57304690

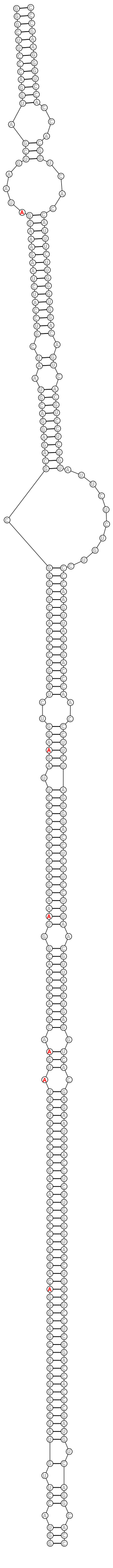

FECH part B

Strands Chr24:57303985-57304115
and Chr24:57305961-57306091

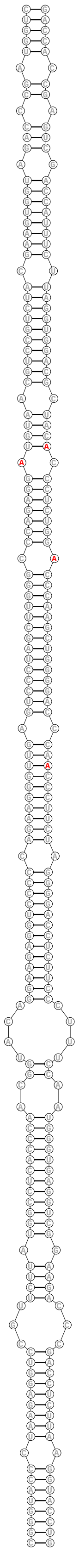

FER1L5

Strands Chr11:2649884-2650082
and Chr11:2650720-2650917

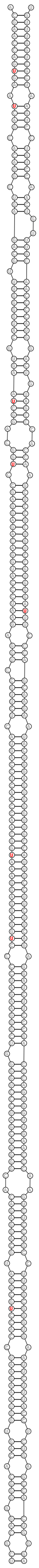

FKBP1A part A

Strands Chr13:60298329-60298464
and Chr13:60299291-60299425

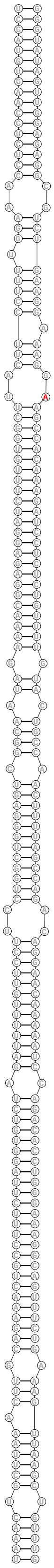

FKBP1A part B

Strands Chr13:60298205-60298343
and Chr13:60299697-60299833

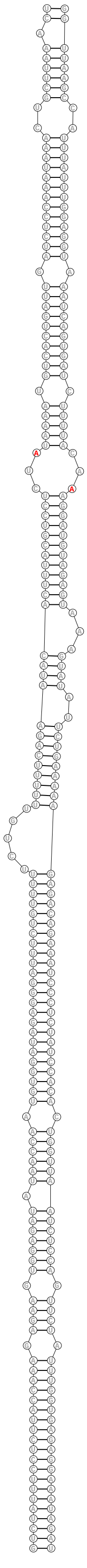

FLNB

Strands Chr22:43703705-43703762
and Chr22:43704012-43704069

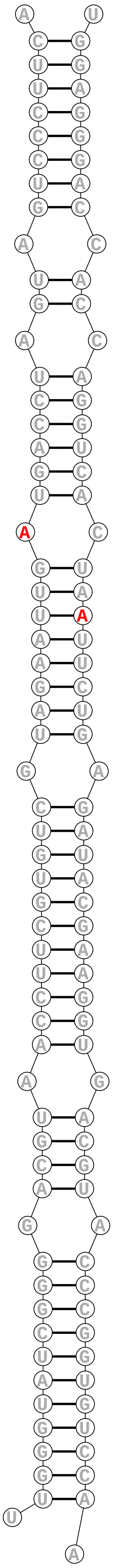

FLVCR1

Strands Chr16:72570307-72570433
and Chr16:72571482-72571604

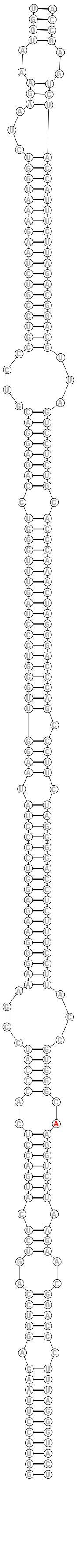

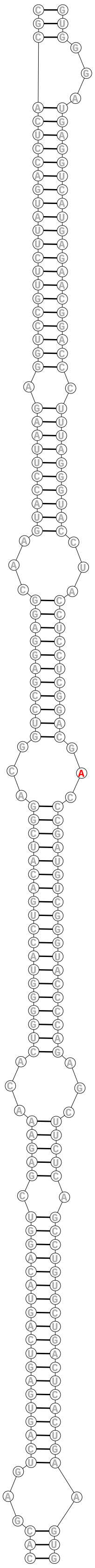

FUT1

Strands Chr18:55829529-55829632
and Chr18:55830094-55830198

FUT11

Strands Chr28:29839027-29839065
and Chr28:29839101-29839138

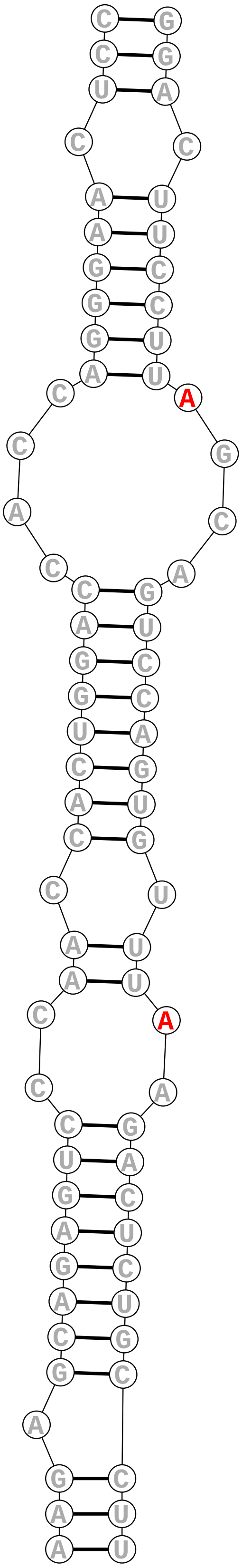

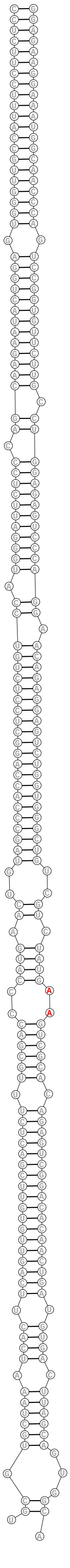

GALNT12

Strands Chr8:64333399-64333523
and Chr8:64334046-64334172

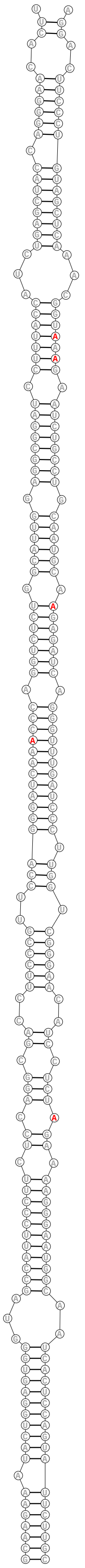

GBAS

Strands Chr25:27940583-27940705
and Chr25:27943205-27943324

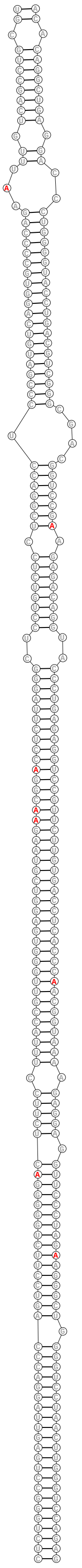

GGT1 part A

Strands Chr17:73443294-73443433
and Chr17:73444334-73444477

GGT1 part B

Strands Chr17:73443262-73443441
and Chr17:73445611-73445795

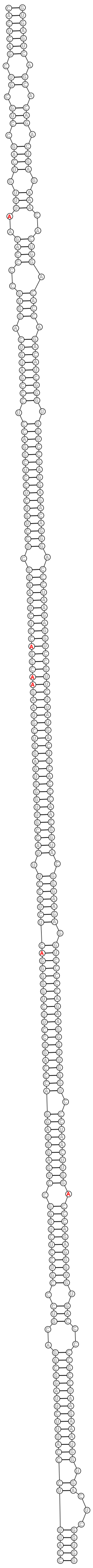

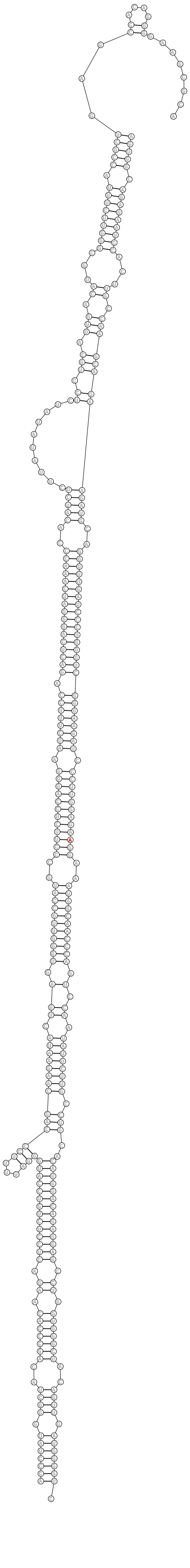

GNG5 part A

Strands Chr3:59786345-59786527
and Chr3:59787654-59787800

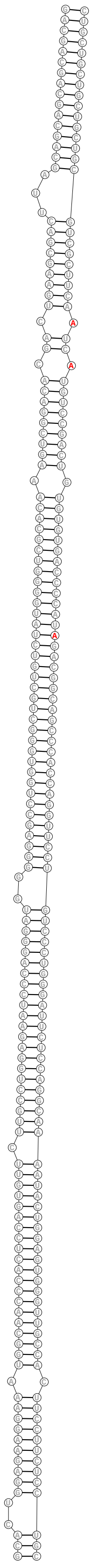

GNG5 part B

Strands Chr3:59789232-59789370
and Chr3:59791142-59791272

GNPTG part A

Strands Chr25:1071280-1071336
and Chr25:1071817-1071874

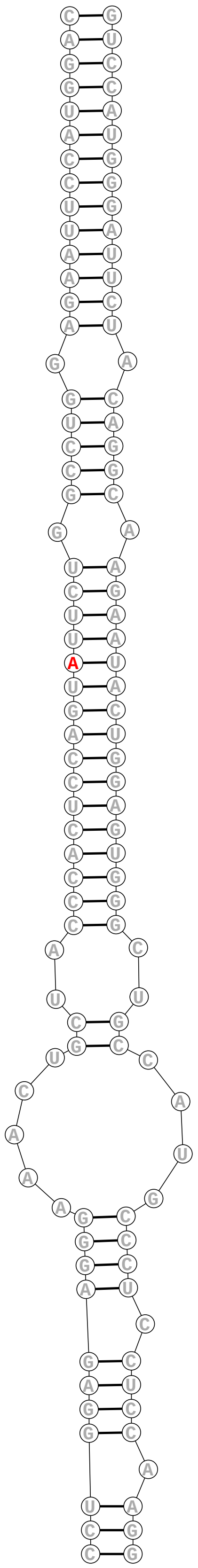

GNPTG part B

Strands Chr25:1071808-1071901
and Chr25:1072747-1072838

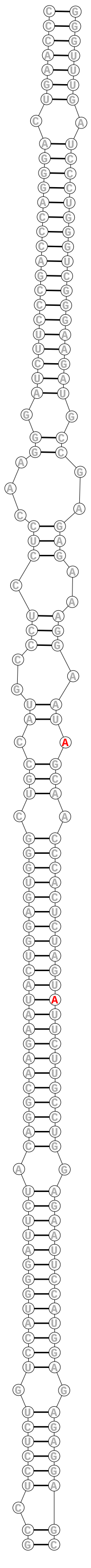

GOLGB1

Strands Chr1:66765344-66765433
and Chr1:66766428-66766518

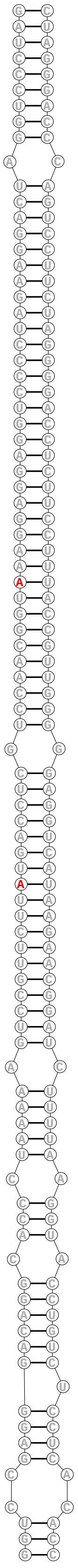

GPAM

Strands Chr26:32977056-32977267
and Chr26:32977782-32978002

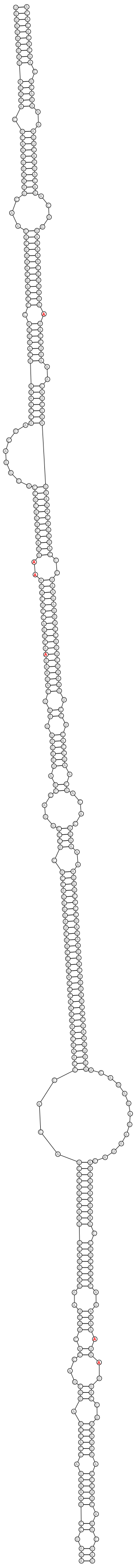

GPR161

Strands Chr3:594340-594463
and Chr3:594584-594714

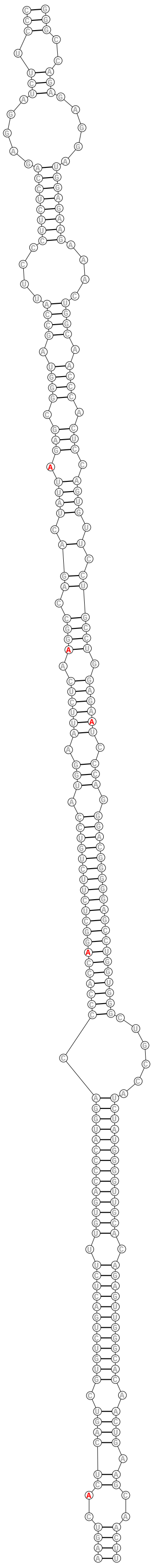

GPRC5B

Strands Chr25:17597401-17597495
and Chr25:17597794-17597888

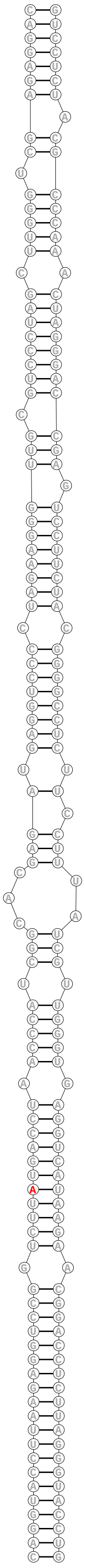

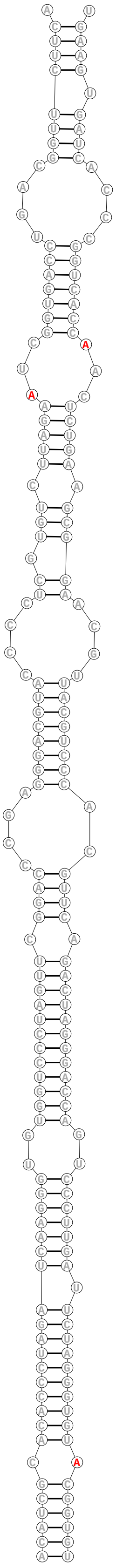

GPRC5C

Strands Chr19:57649878-57649971
and Chr19:57653179-57653271

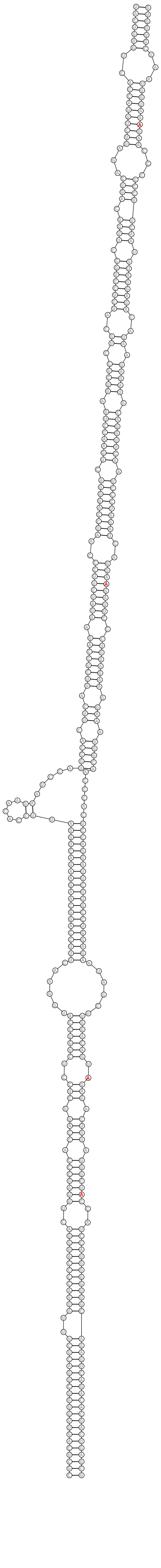

GRAMD1B

Strands Chr15:34755855-34756058
and Chr15:34756896-34757088

GRAMD3

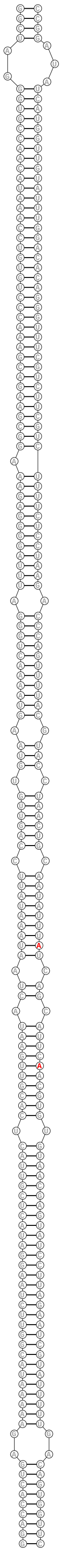

Strands Chr7:28658747-28658890
and Chr7:28660148-28660291

GRHL2

Strands Chr14:64912771-64912891
and Chr14:64913378-64913500

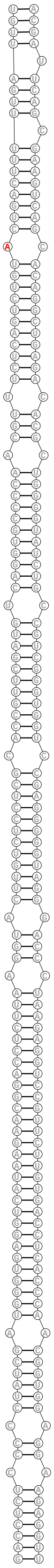

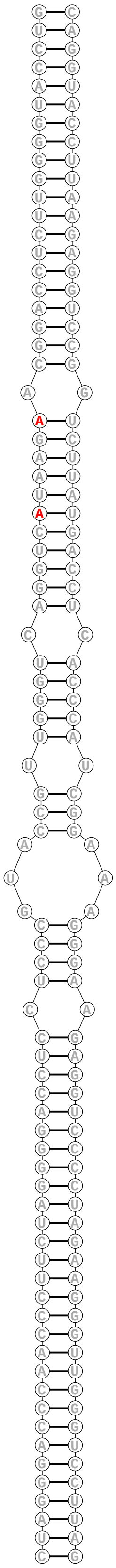

GRK4

Strands Chr6:107891580-107891658
and Chr6:107892412-107892490

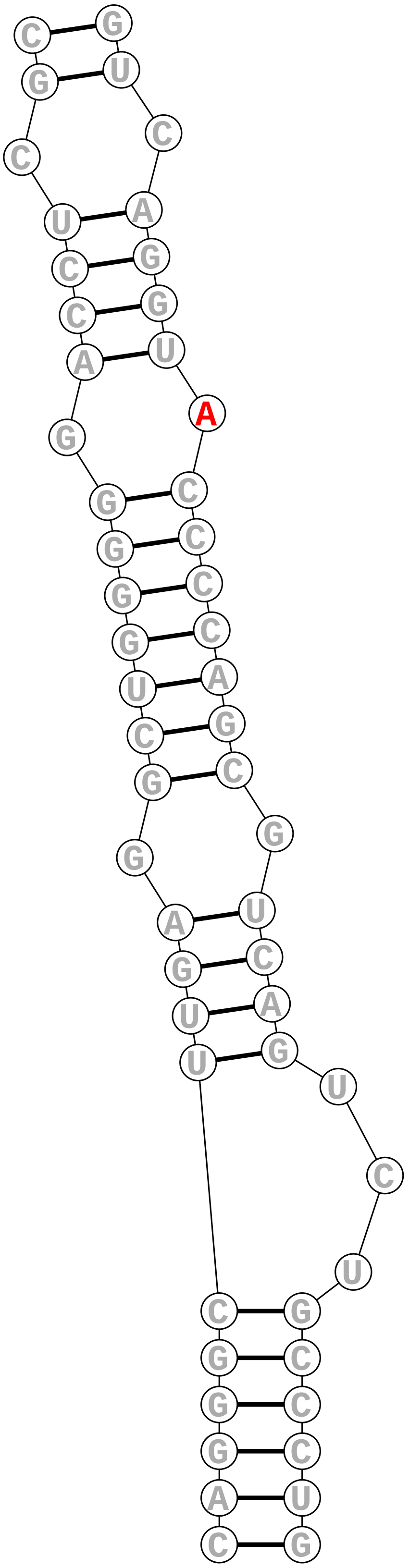

GRTP1

Strands Chr12:90627867-90627892
and Chr12:90629262-90629290

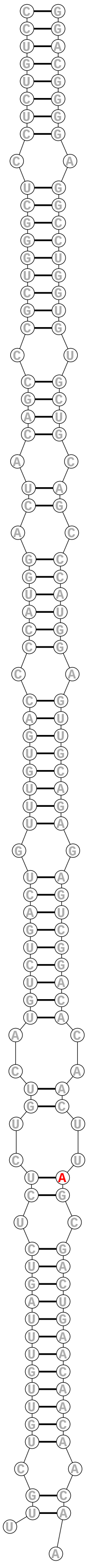

GTPBP8

Strands Chr1:58173719-58173795
and Chr1:58174600-58174676

HAPLN3 part A

Strands Chr21:20880181-20880367
and Chr21:20881004-20881188

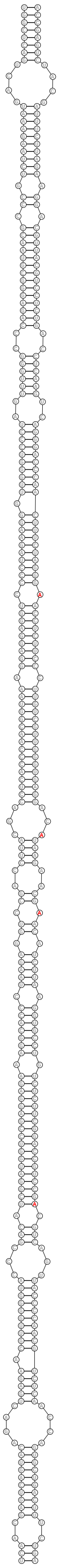

HAPLN3 part B

Strands Chr21:20881565-20881749
and Chr21:20883415-20883625

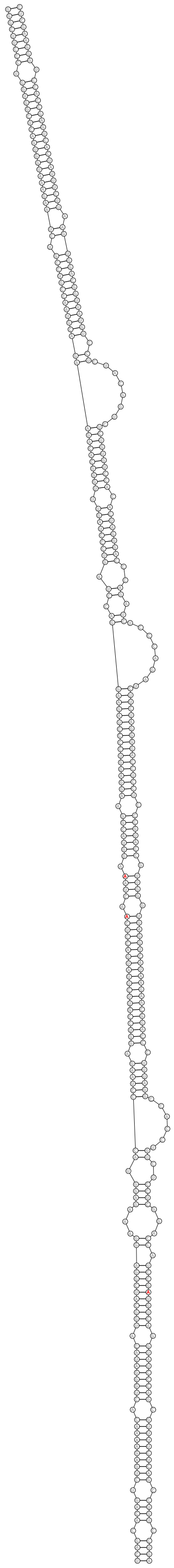

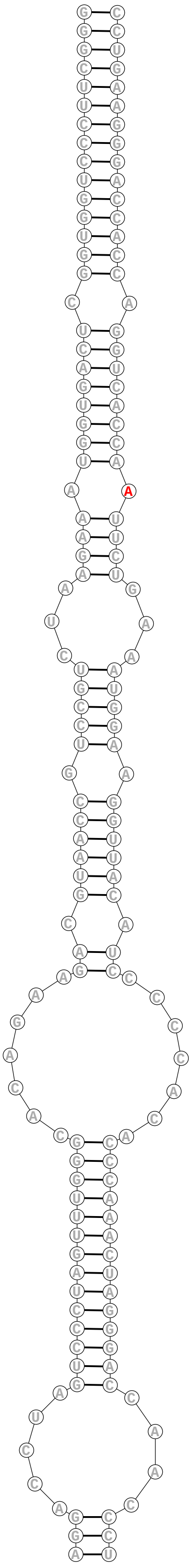

HAPLN3 part C

Strands Chr21:20881674-20881749
and Chr21:20882189-20882263

HAPLN3 part D

Strands Chr21:20882474-20882654
and Chr21:20883421-20883619

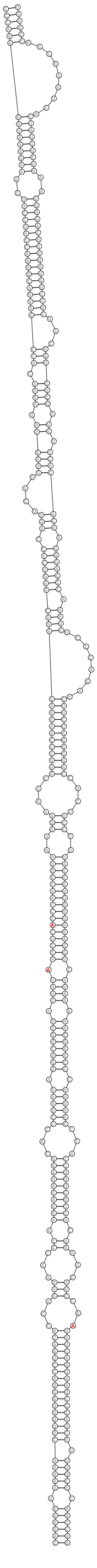

HAUS4

Strands Chr10:21725883-21725955
and Chr10:21726008-21726080

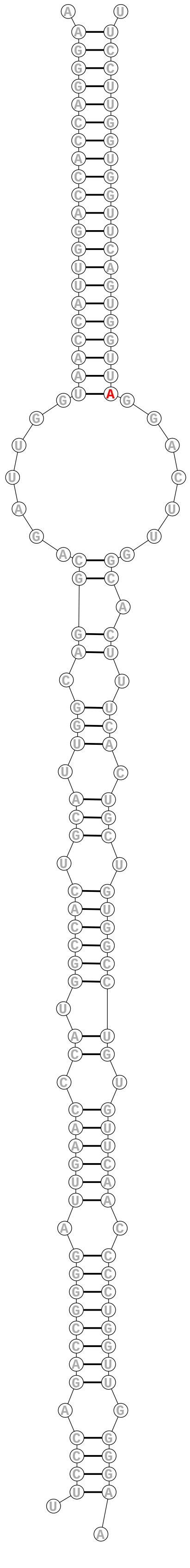

HBP1

Strands Chr4:48572032-48572067
and Chr4:48572773-48572808

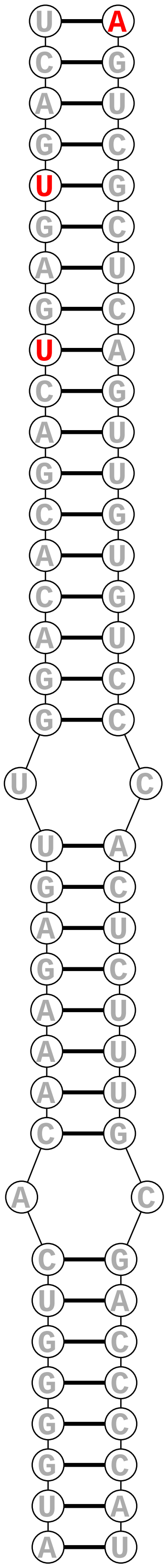

HDDC2

Strands Chr9:26303150-26303243
and Chr9:26303938-26304032

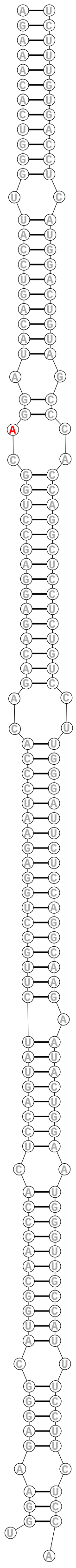

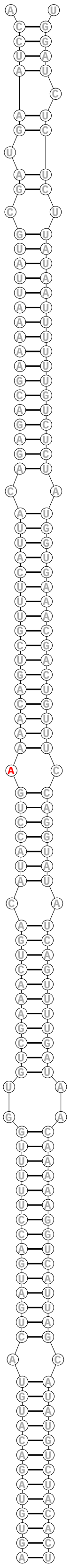

HERC6

Strands Chr6:37756226-37756322
and Chr6:37757440-37757536

HHIPL2 part A

Strands Chr16:26717543-26717690
and Chr16:26718471-26718611

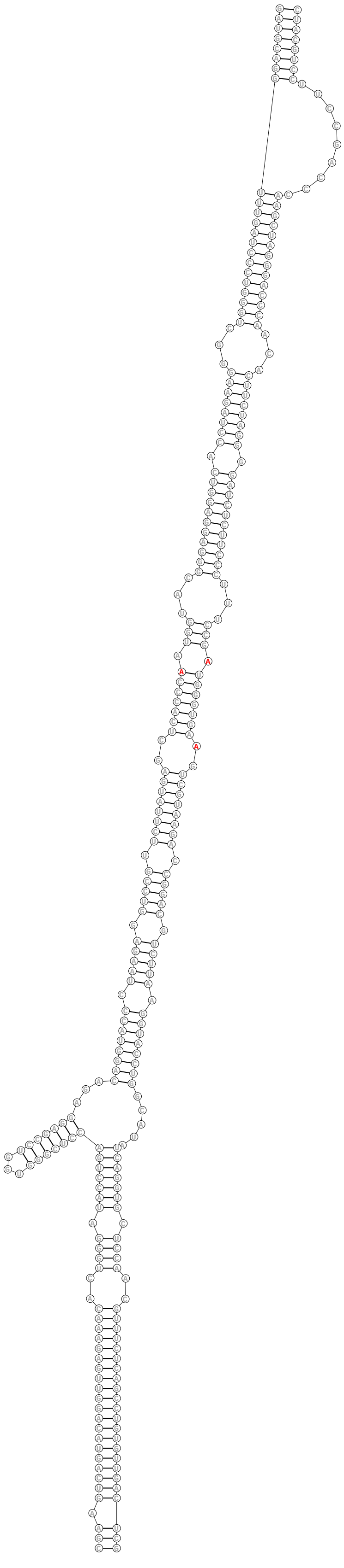

HHIPL2 part B

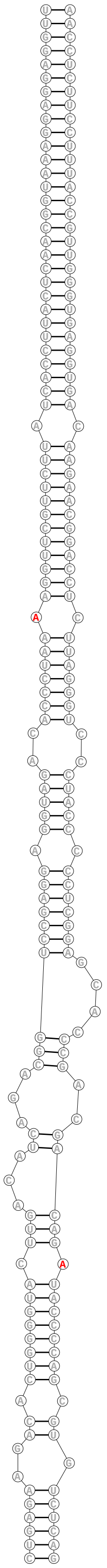

Strands Chr16:26719839-26719938
and Chr16:26721422-26721520

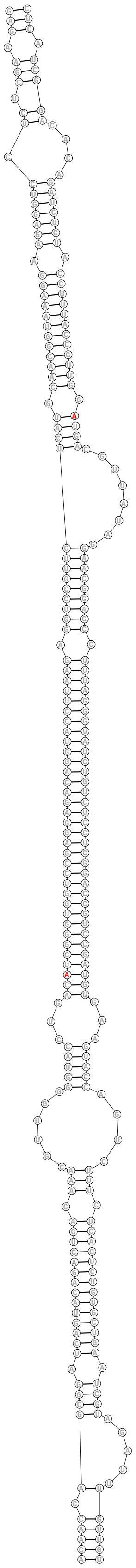

HHIPL2 part C

Strands Chr16:26722108-26722231
and Chr16:26724636-26724771

HIGD1D

Strands Chr22:15514601-15514653
and Chr22:15515069-15515121

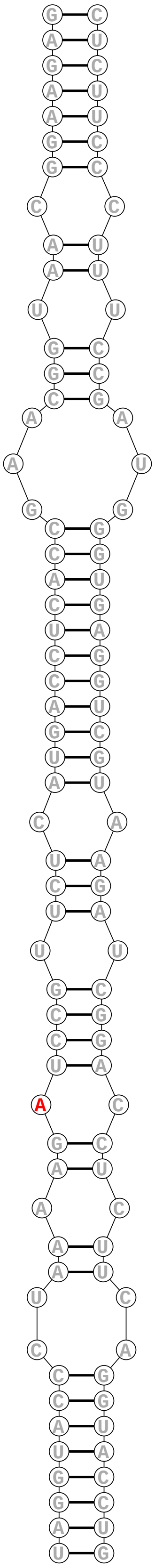

HIKESHI part A

Strands Chr29:9266131-9266210
and Chr29:9266901-9266983

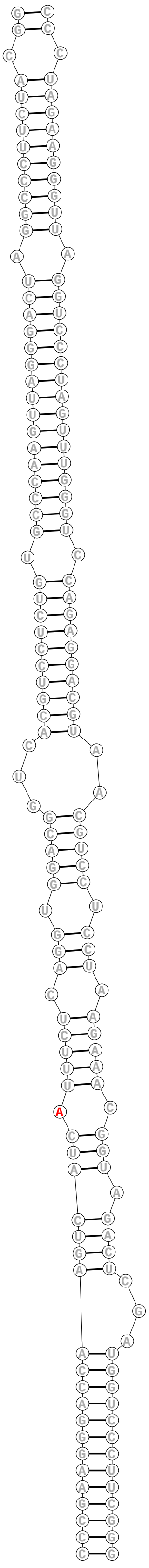

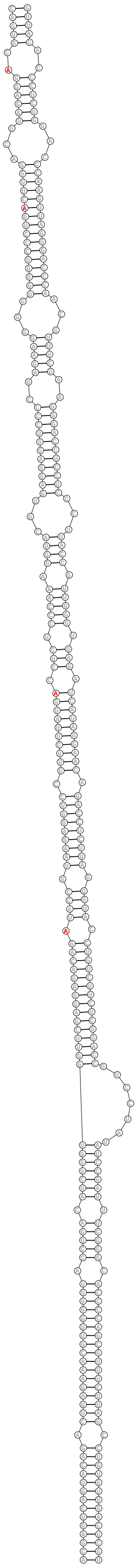

HIKESHI part B

Strands Chr29:9284028-9284185
and Chr29:9284970-9285134

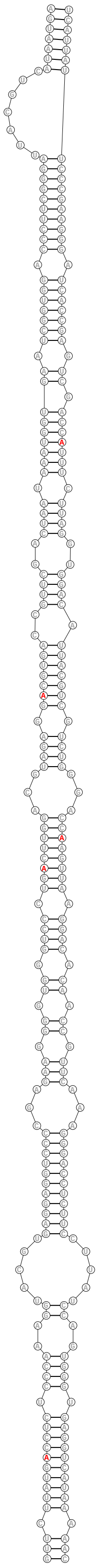

HIKESHI part C

Strands Chr29:9284105-9284239
and Chr29:9284804-9284931

HMGN3 part A

Strands Chr9:19002387-19002541
and Chr9:19002586-19002750

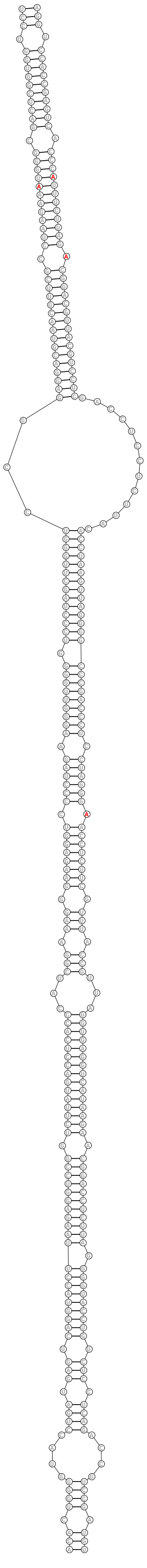

HMGN3 part B

Strands Chr9:19004453-19004700
and Chr9:19005837-19006078

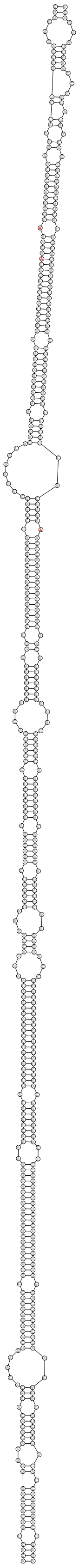

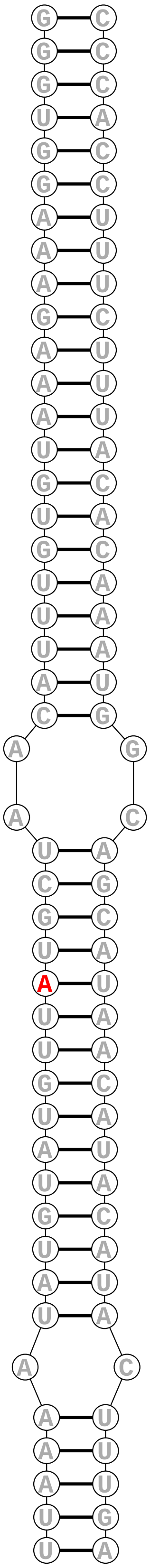

HMGXB3

Strands Chr7:63338422-63338466
and Chr7:63338731-63338775

HOOK3

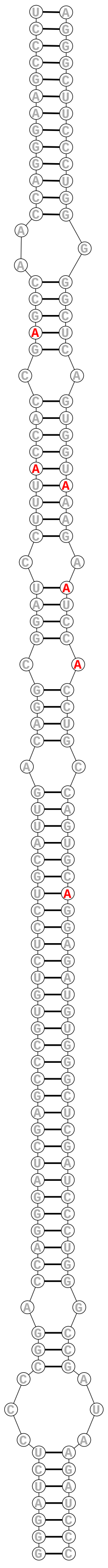

Strands Chr27:37355446-37355530
and Chr27:37356243-37356326

HPSE part A

Strands Chr6:99861958-99862122
and Chr6:99862360-99862517

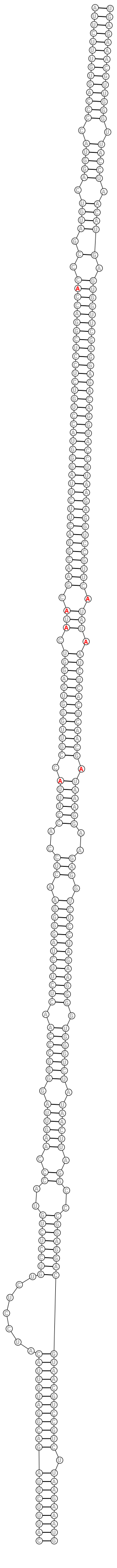

HPSE part B

Strands Chr6:99861989-99862148
and Chr6:99862802-99862962

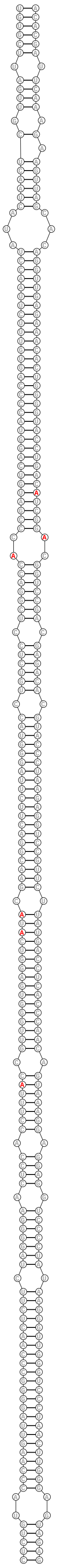

HS1BP3

Strands Chr11:78306038-78306245
and Chr11:78308600-78308807

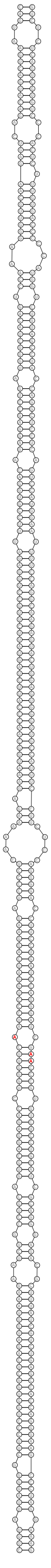

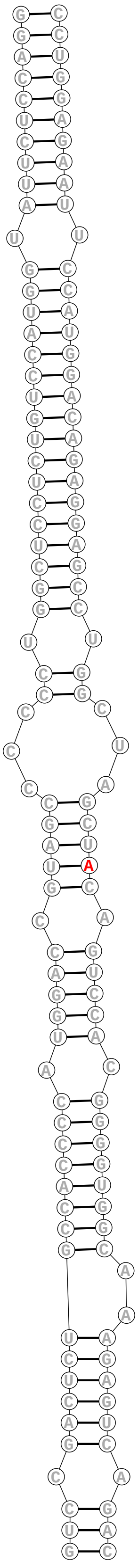

HSD17B1

Strands Chr19:43266907-43266970
and Chr19:43268393-43268458

HSPA14

Strands Chr13:29785322-29785376
and Chr13:29785610-29785664

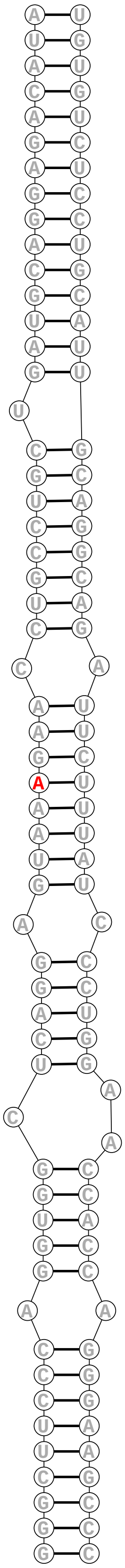

HTATIP2

Strands Chr29:24809600-24809795
and Chr29:24810978-24811171

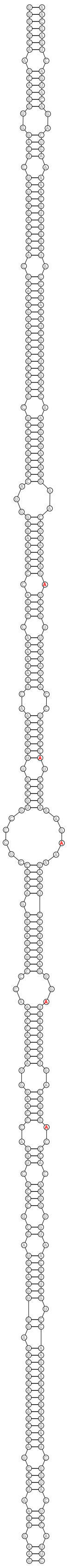

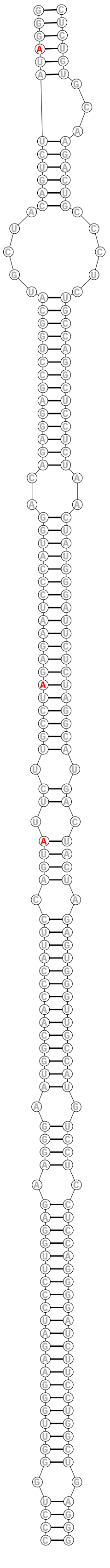

HTSN

Strands Chr6:87193790-87193896
and Chr6:87195322-87195431

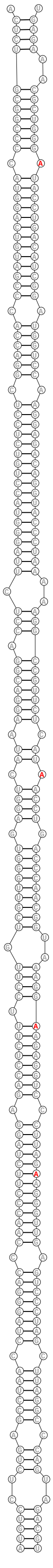

IAH1 part A

Strands Chr11:87948531-87948667
and Chr11:87949484-87949622

IAH1 part B

Strands Chr11:87949467-87949513
and Chr11:87949879-87949928

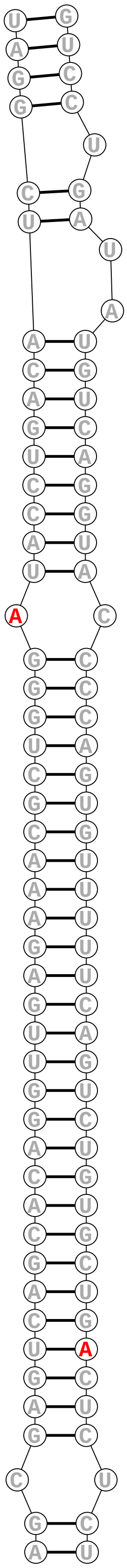

IAH1 part C

Strands Chr11:87949541-87949661
and Chr11:87949751-87949866

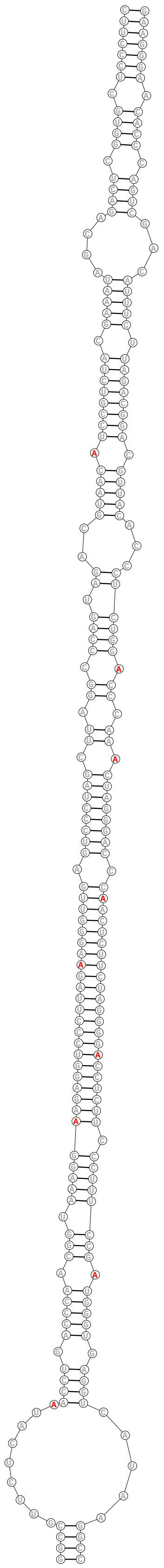

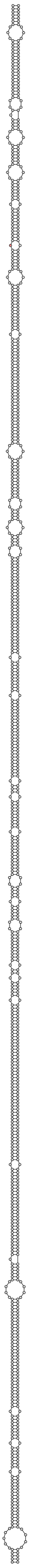

IARS2

Strands Chr16:24322824-24323284
and Chr16:24323724-24324181

ICA1 part A

Strands Chr4:17180503-17180678
and Chr4:17182069-17182243

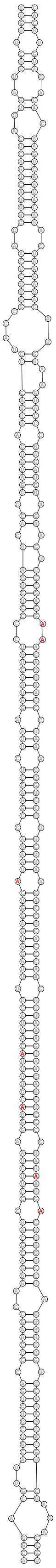

ICA1 part B

Strands Chr4:17180471-17180580
and Chr4:17183791-17183900

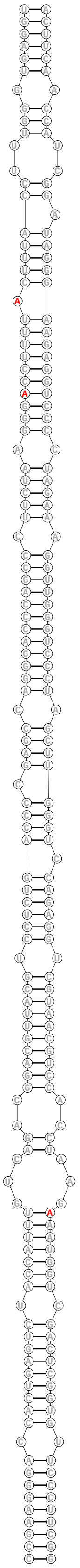

ICAM3

Strands Chr7:16091794-16091822
and Chr7:16092497-16092526

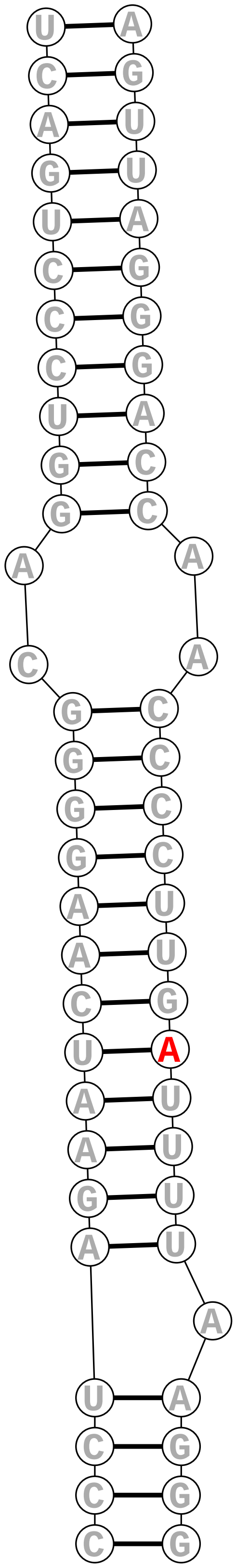

IFNGR2 part A

Strands Chr1:1377586-1377806
and Chr1:1378444-1378660

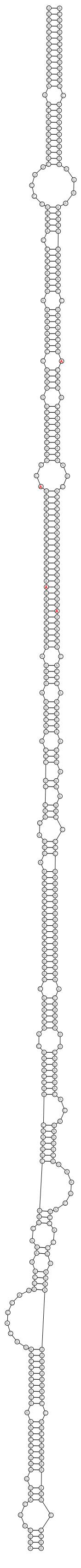

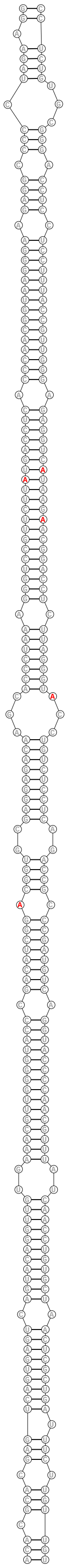

IFNGR2 part B

Strands Chr1:1378438-1378575
and Chr1:1380012-1380150

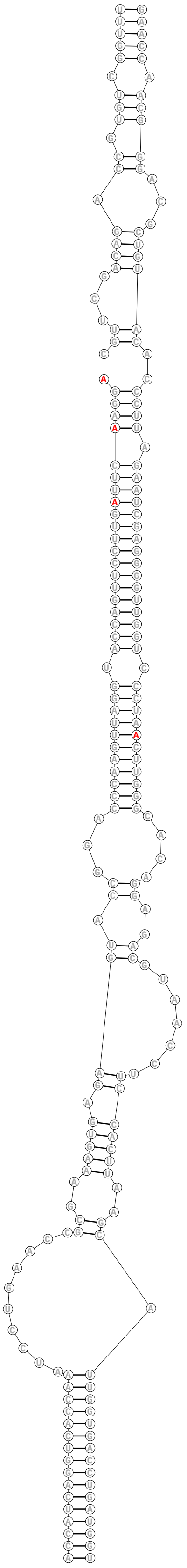

IFNGR2 part C

Strands Chr1:1383192-1383293
and Chr1:1385256-1385355

IFNGR2 part D

Strands Chr1:1384933-1385038
and Chr1:1385263-1385365

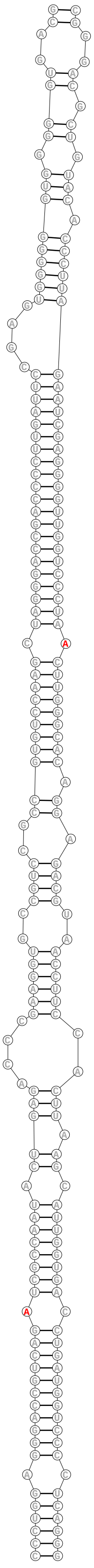

IL27RA part A

Strands Chr7:12744884-12745107
and Chr7:12745970-12746194

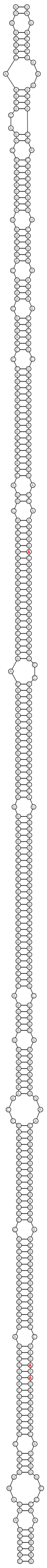

IL27RA part B

Strands Chr7:12746217-12746419
and Chr7:12751499-12751703

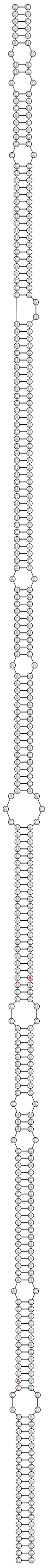

IL27RA part C

Strands Chr7:12746218-12746443
and Chr7:12752769-12752987

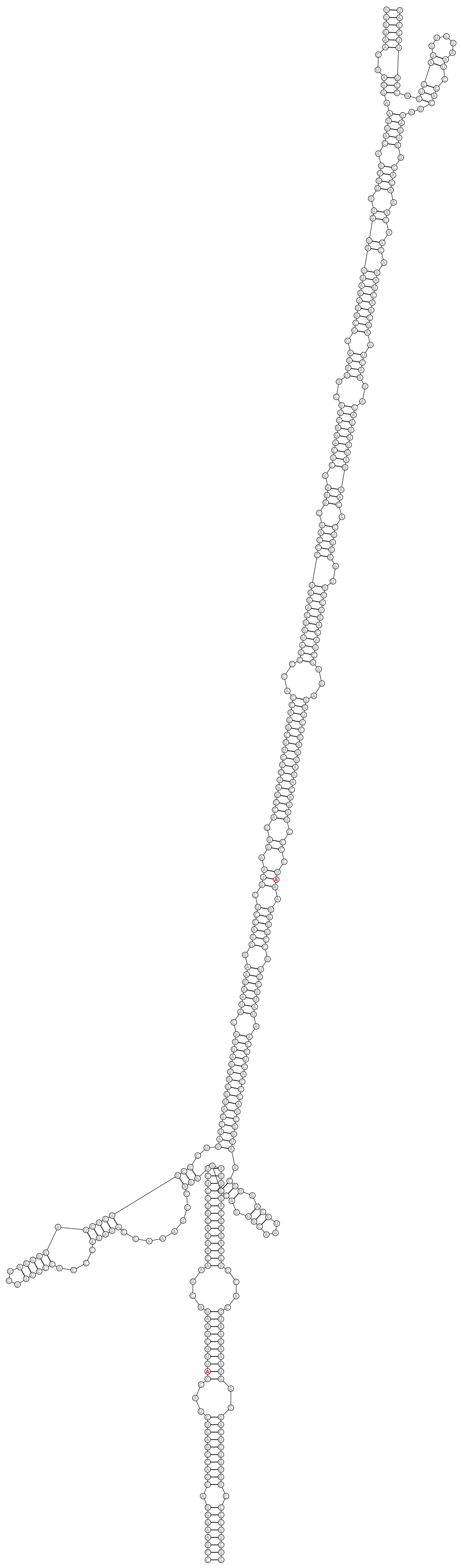

IL27RA part D

Strands Chr7:12748865-12748996
and Chr7:12749529-12749662

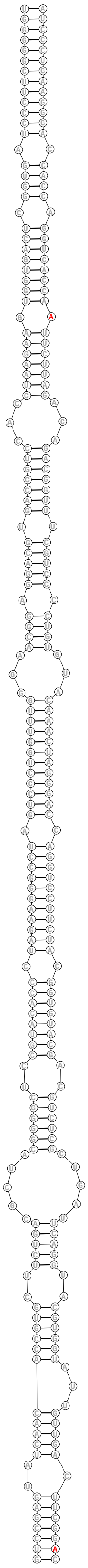

IL27RA part E

Strands Chr7:12748658-12748827
and Chr7:12749641-12749797

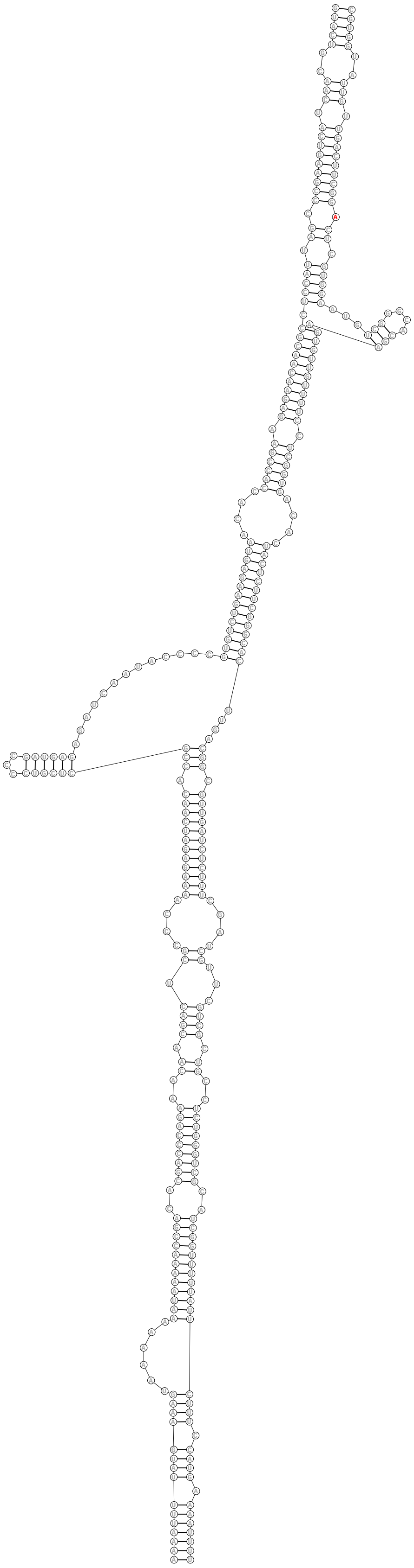

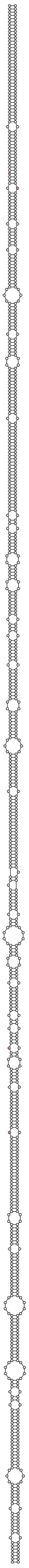

IL33

Strands Chr8:38733758-38734198
and Chr8:38736733-38737173

INO80C part A

Strands Chr24:21916015-21916105
and Chr24:21916253-21916335

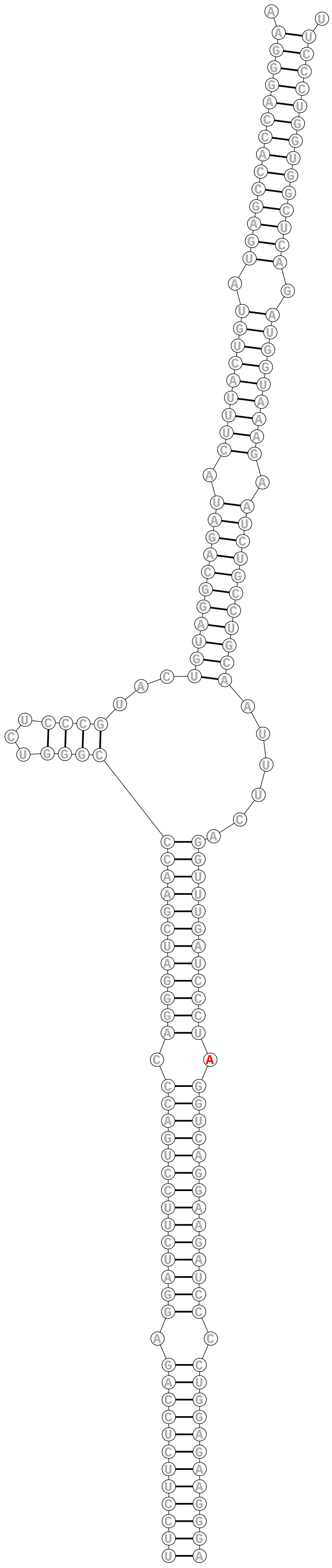

INO80C part B

Strands Chr24:21918187-21918296
and Chr24:21918585-21918687

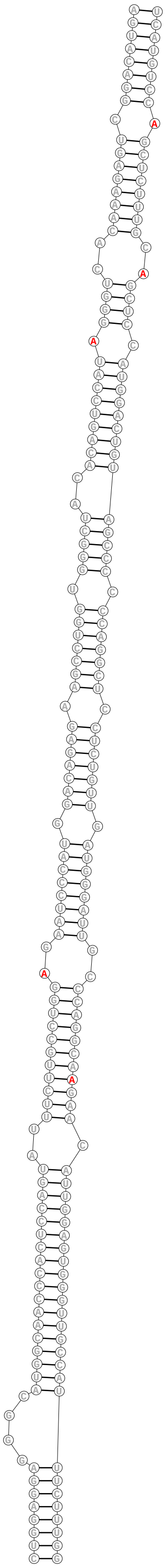

INPP1

Strands Chr2:5881853-5881950
and Chr2:5883817-5883913

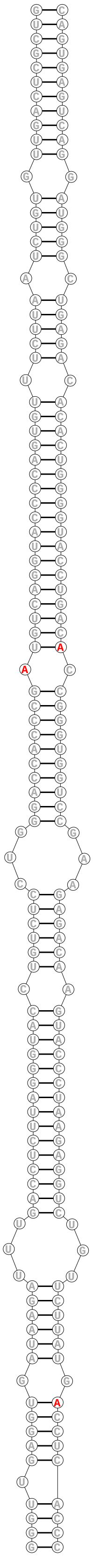

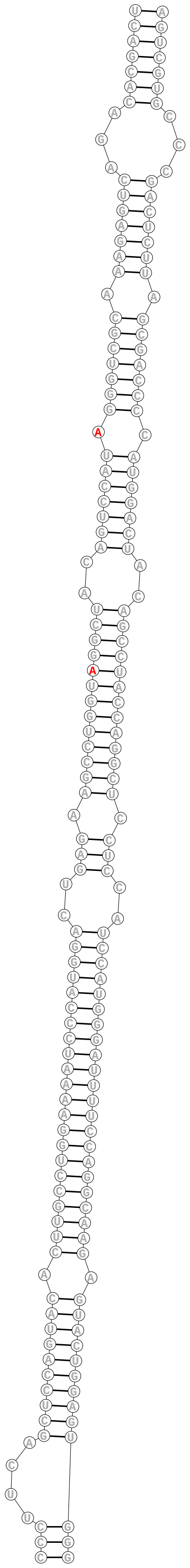

INPP5B

Strands Chr3:108646630-108646723
and Chr3:108647495-108647584

IQCE

Strands Chr25:41228584-41228664
and Chr25:41229796-41229874

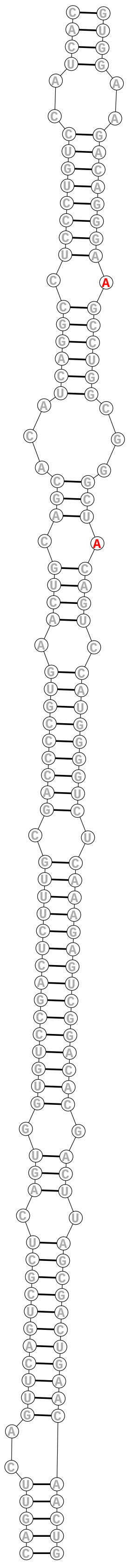

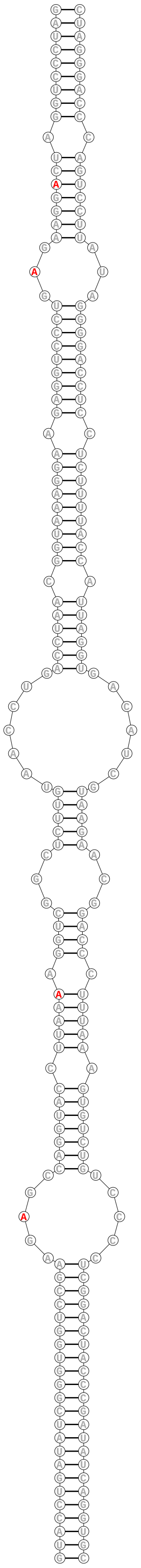

ITGA6

Strands Chr2:24134828-24134934
and Chr2:24135212-24135318

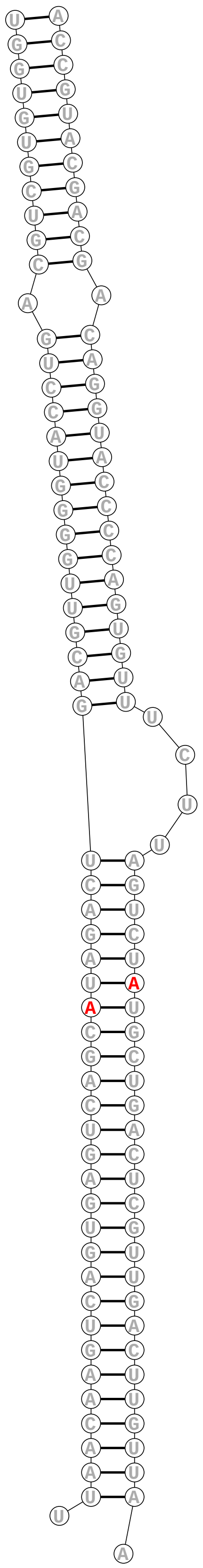

KANK3 part A

Strands Chr7:18202289-18202344
and Chr7:18202808-18202867

KANK3 part B

Strands Chr7:18202369-18202391
and Chr7:18202772-18202795

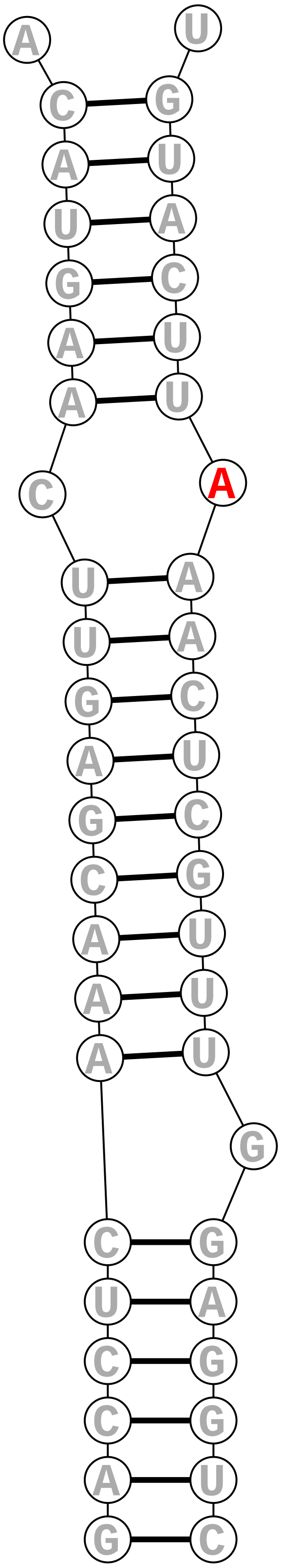

KCNK6 part A

Strands Chr18:48400414-48400564
and Chr18:48401848-48401994

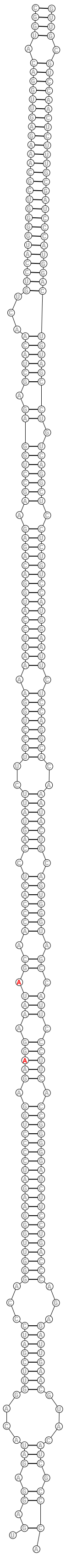

KCNK6 part B

Strands Chr18:48403945-48404045
and Chr18:48404355-48404455

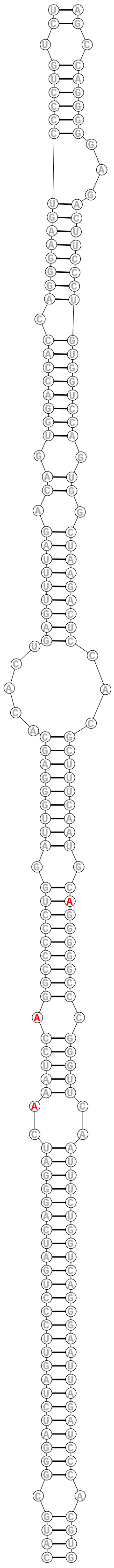

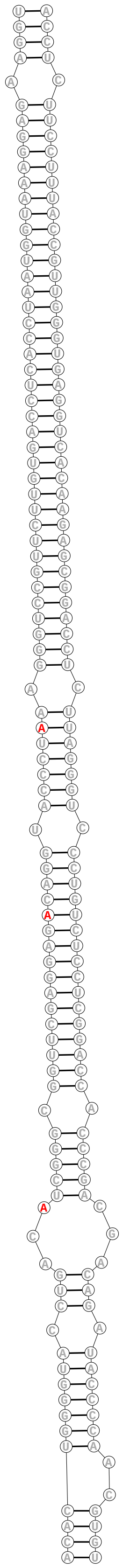

KDELR1

Strands Chr18:55531969-55532059
and Chr18:55532659-55532751

KIAA0196

Strands Chr14:16713444-16713498
and Chr14:16714326-16714381

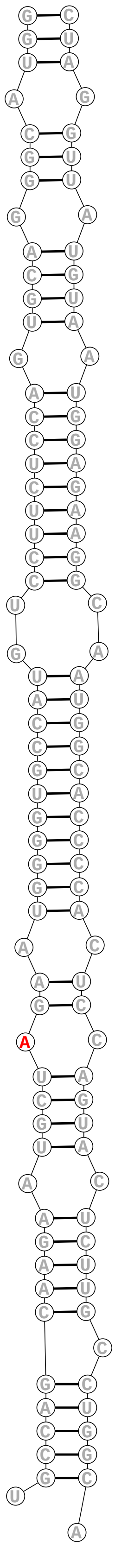

KIF1BP

Strands Chr28:25457507-25457633
and Chr28:25457706-25457832

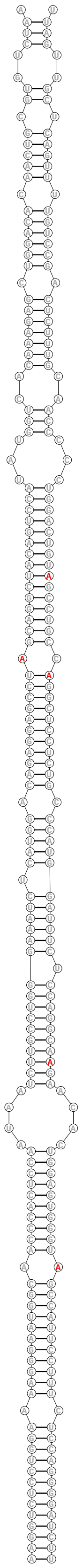

KIF1C part A

Strands Chr19:27020705-27020898
and Chr19:27021129-27021325

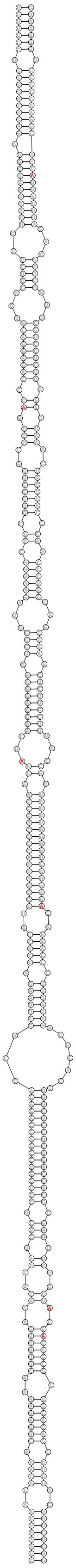

KIF25

Strands Chr9:103956880-103957061
and Chr9:103957531-103957712

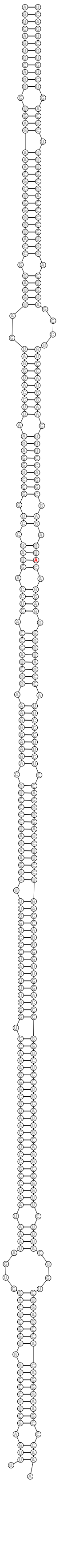

KIN part A

Strands Chr13:16208656-16208694
and Chr13:16208892-16208928

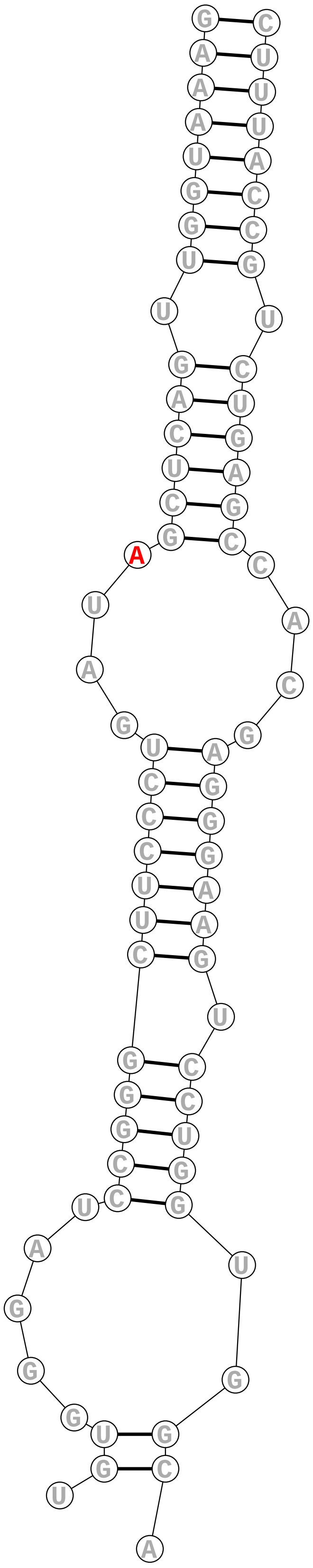

KIN part B

Strands Chr13:16208712-16208897
and Chr13:16209008-16209194

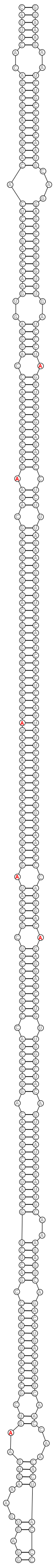

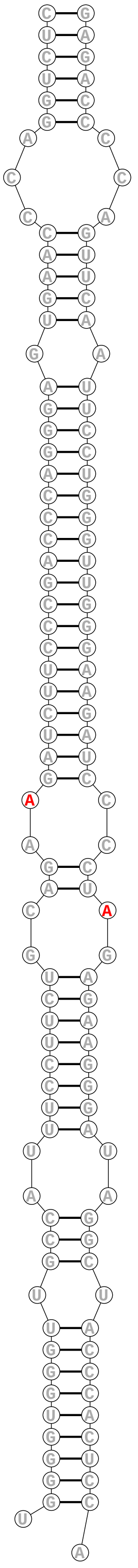

KIN part C

Strands Chr13:16208812-16208875
and Chr13:16208935-16208998

KIN part D

Strands Chr13:16208784-16208920
and Chr13:16209209-16209344

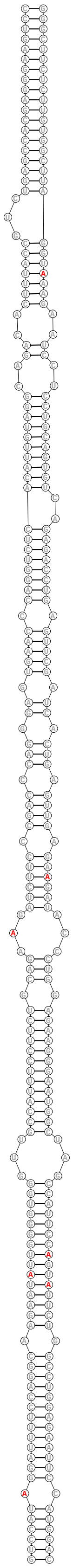

KLHL40

Strands Chr22:15453972-15454018
and Chr22:15454097-15454140

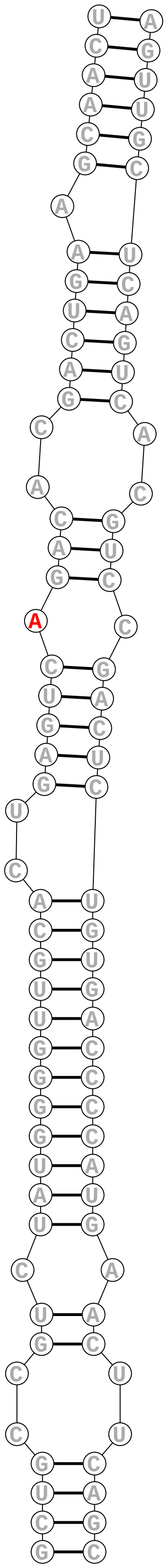

LARP7

Strands Chr6:14140229-14140272
and Chr6:14141069-14141113

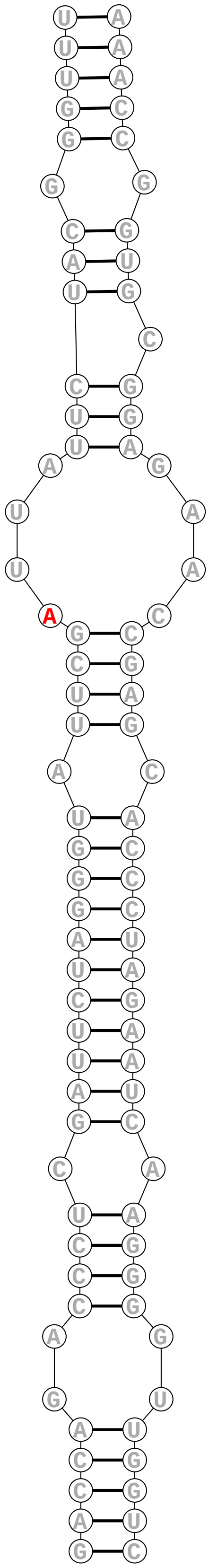

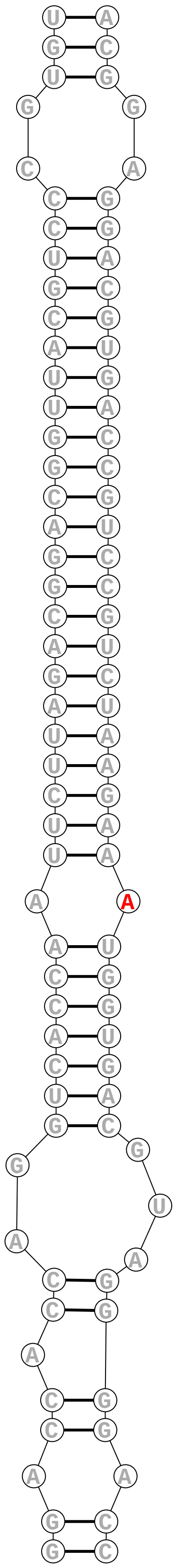

LCP2

Strands Chr20:2036903-2036948
and Chr20:2037936-2037981

LMAN2L part A

Strands Chr11:2641966-2642175
and Chr11:2647296-2647496

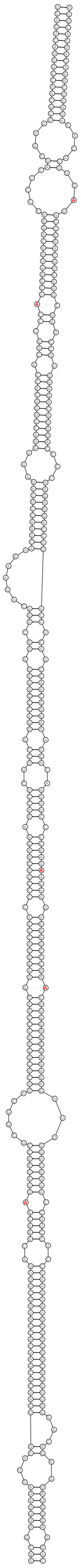

LMAN2L part B

Strands Chr11:2643758-2643962
and Chr11:2647281-2647492

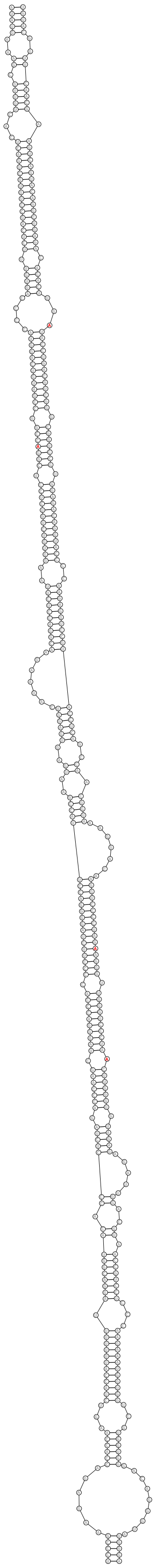

LMAN2L part C

Strands Chr11:2649868-2650098
and Chr11:2650711-2650932

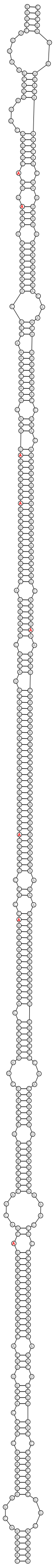

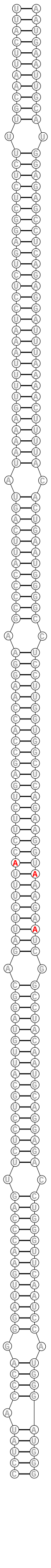

LPL part A

Strands Chr8:67505229-67505354
and Chr8:67505821-67505945

LPL part B

Strands Chr8:67506964-67507073
and Chr8:67508349-67508458

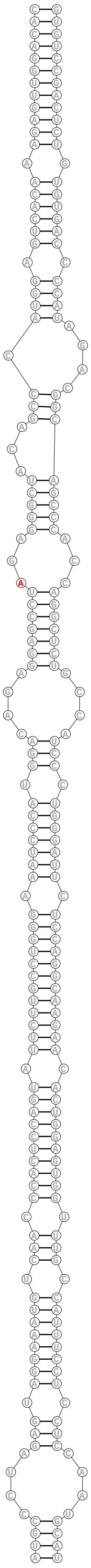

LPO part A

Strands Chr19:9446298-9446424
and Chr19:9446557-9446681

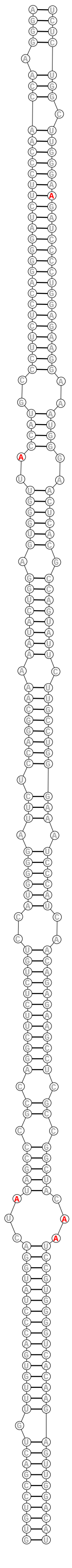

LPO part B

Strands Chr19:9475487-9475525
and Chr19:9475922-9475961

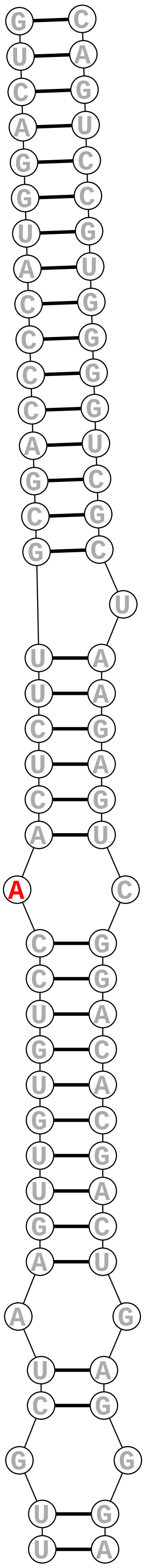

LRCH4

Strands Chr25:36501864-36501911
and Chr25:36502567-36502613

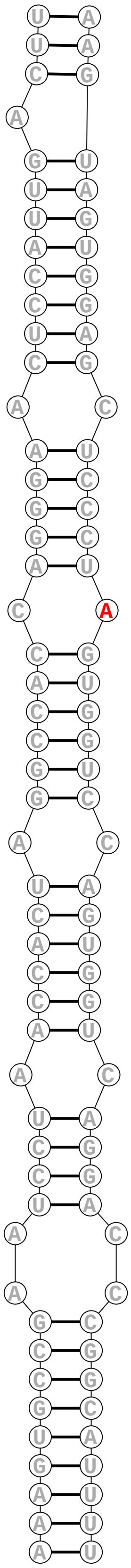

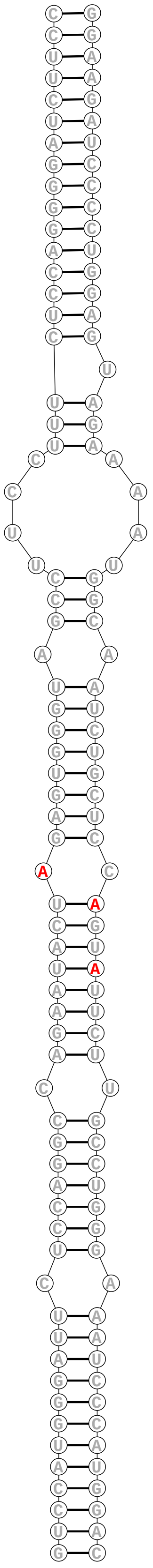

LRRFIP1

Strands Chr3:117862976-117863040
and Chr3:117863825-117863890

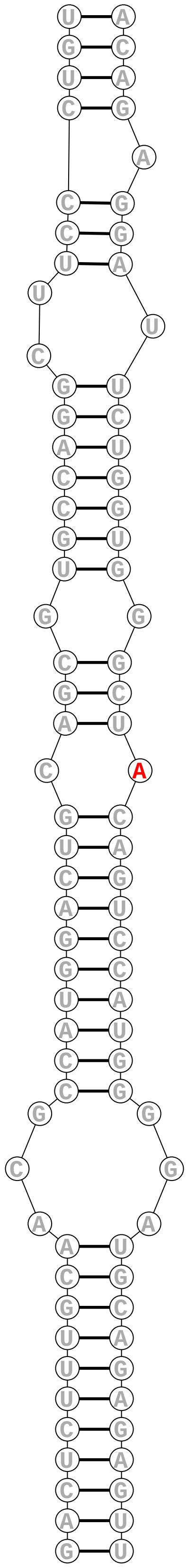

LSG1

Strands Chr1:73503701-73503745
and Chr1:73504482-73504526

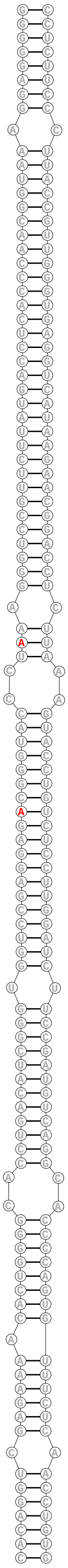

LSM6 part A

Strands Chr17:12336074-12336177
and Chr17:12337166-12337268

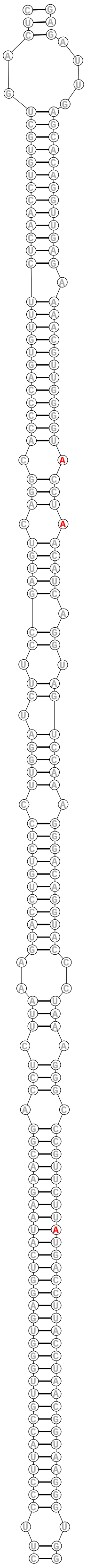

LSM6 part B

Strands Chr17:12337169-12337275
and Chr17:12337938-12338047

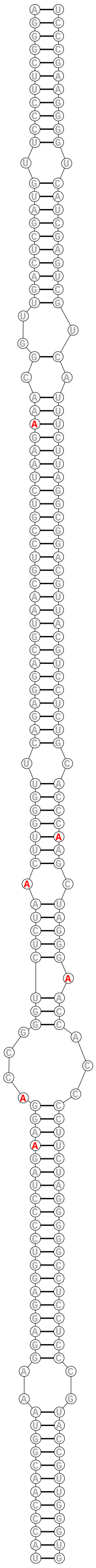

LTBR

Strands Chr5:104351221-104351328
and Chr5:104351945-104352051

LTF part A

Strands Chr22:53539209-53539370
and Chr22:53540466-53540636

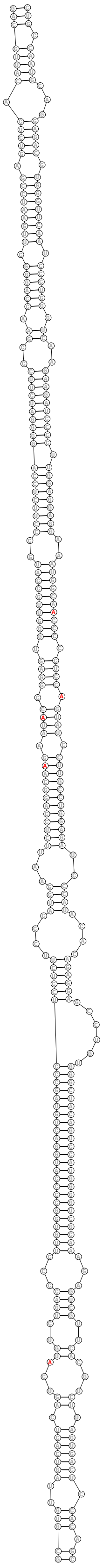

LTF part B

Strands Chr22:53540449-53540639
and Chr22:53544890-53545080

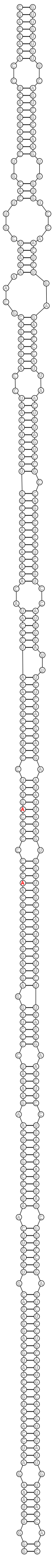

LTF part C

Strands Chr22:53540515-53540639
and Chr22:53547841-53547967

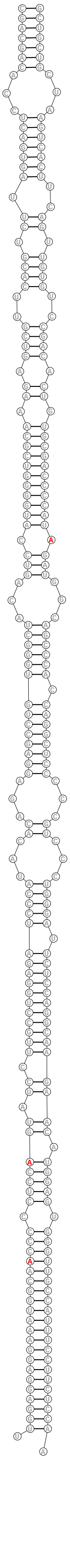

LTF part D

Strands Chr22:53546815-53547082
and Chr22:53547838-53548104

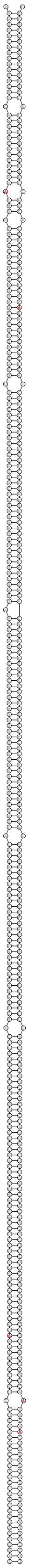

LUC7L

Strands Chr25:246285-246381
and Chr25:247921-248013

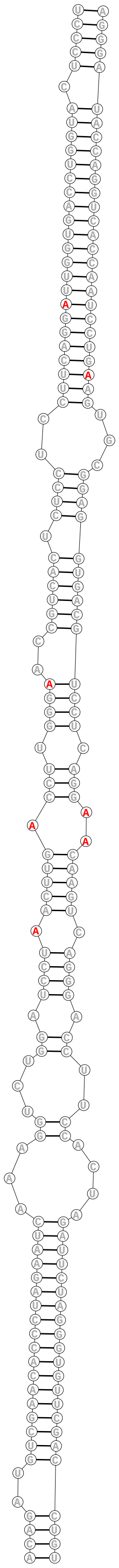

LYRM1

Strands Chr25:18847014-18847132
and Chr25:18848075-18848179

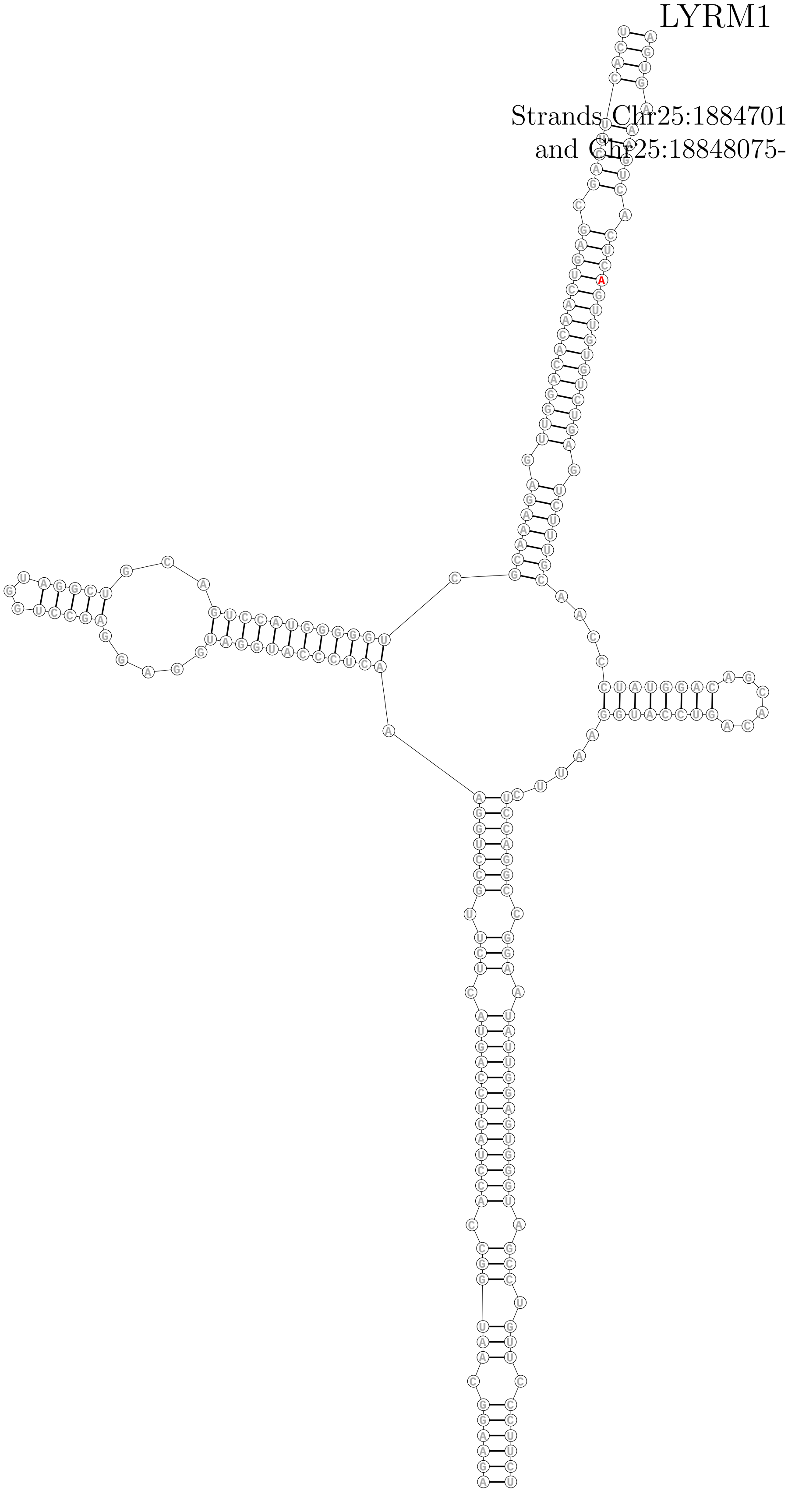

MAP2K2

Strands Chr7:21141733-21141797
and Chr7:21142589-21142660

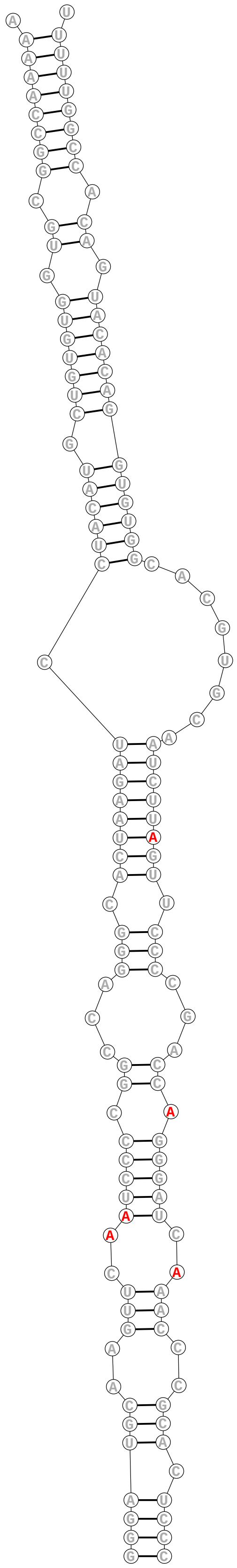

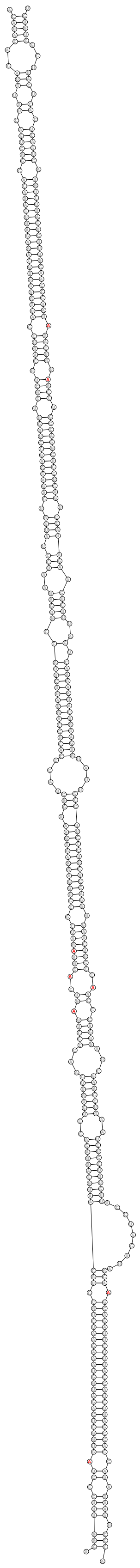

MARC1 part A

Strands Chr16:24990488-24990697
and Chr16:24991201-24991420

MARC1 part B

Strands Chr16:24990382-24990692
and Chr16:24991965-24992275

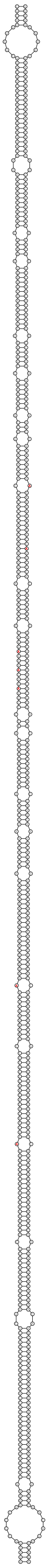

MARC1 part C

Strands Chr16:24991999-24992179
and Chr16:24994813-24994994

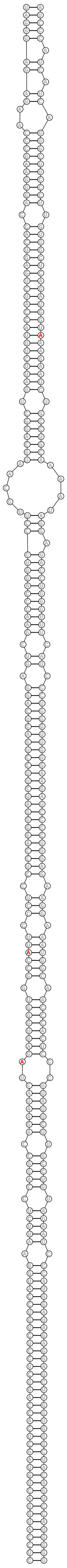

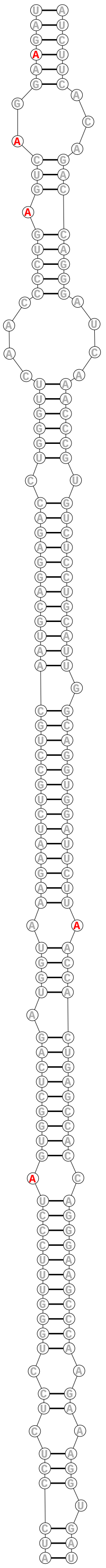

MARC1 part D

Strands Chr16:24990478-24990568
and Chr16:24994698-24994789

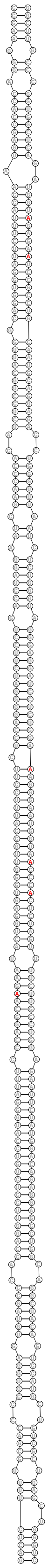

MARCH2 part A

Strands Chr7:18269930-18270111
and Chr7:18271996-18272178

MARCH2 part B

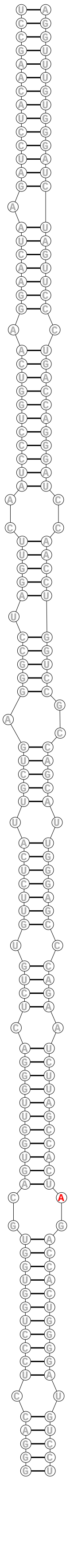

Strands Chr7:18271397-18271493
and Chr7:18272208-18272303

ME3

Strands Chr29:9165159-9165339
and Chr29:9166165-9166354

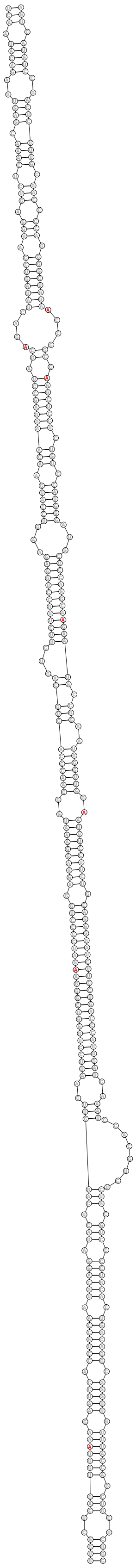

MED4

Strands Chr12:17974934-17975036
and Chr12:17976382-17976485

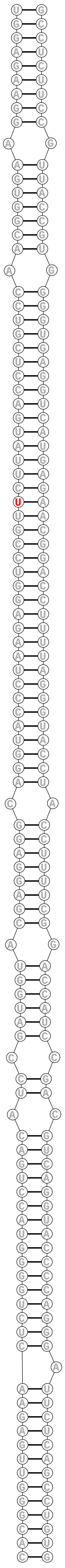

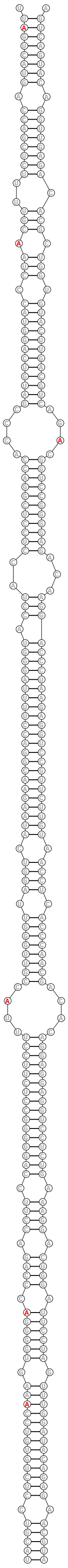

MED6

Strands Chr10:82557497-82557650
and Chr10:82558204-82558356

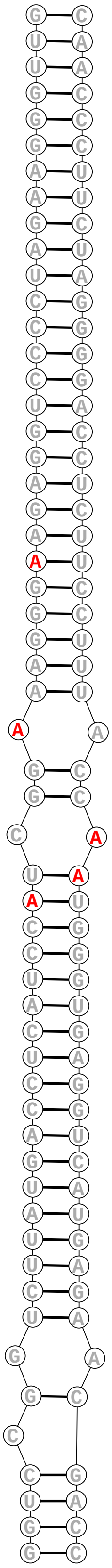

METTL23 part A

Strands Chr19:55672337-55672392
and Chr19:55673182-55673236

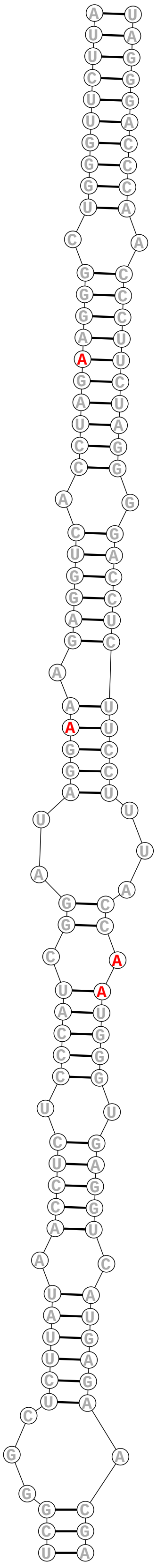

METTL23 part B

Strands Chr19:55672444-55672506
and Chr19:55673174-55673234

METTL23 part C

Strands Chr19:55672511-55672568
and Chr19:55673113-55673168

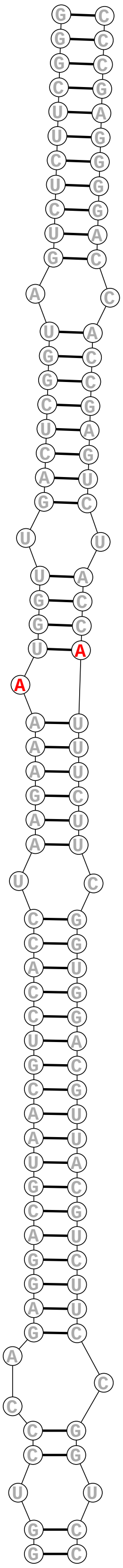

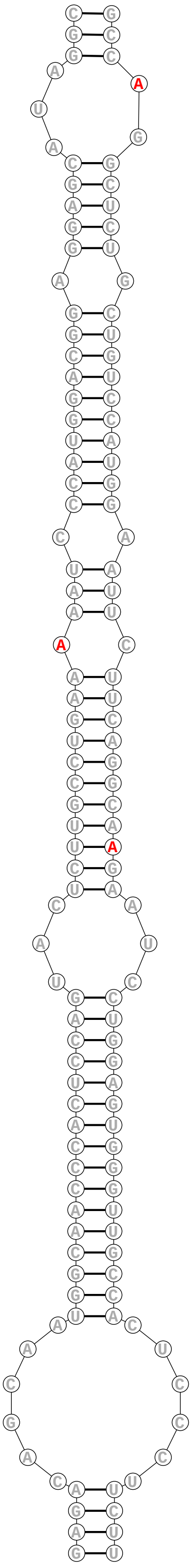

MFSD4A part A

Strands Chr16:3172817-3172883
and Chr16:3175199-3175264

MFSD4A part B

Strands Chr16:3172934-3173062
and Chr16:3175134-3175264

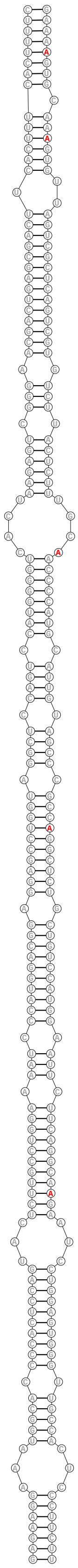

MGST2

Strands Chr17:18335499-18335626
and Chr17:18336528-18336655

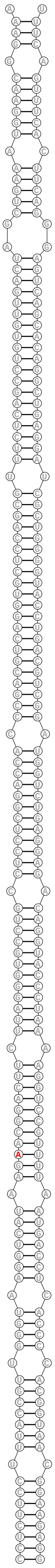

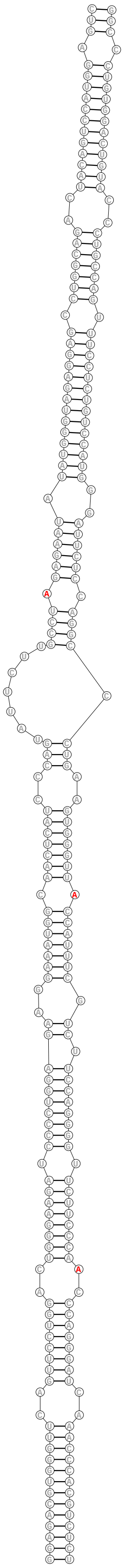

MIOS

Strands Chr4:15577143-15577267
and Chr4:15577529-15577648

MMAB

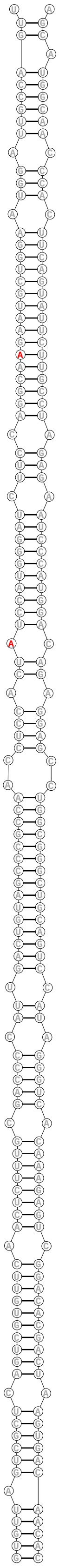

Strands Chr17:65888181-65888292
and Chr17:65889268-65889379

MMACHC

Strands Chr3:101168707-101168781
and Chr3:101169506-101169576

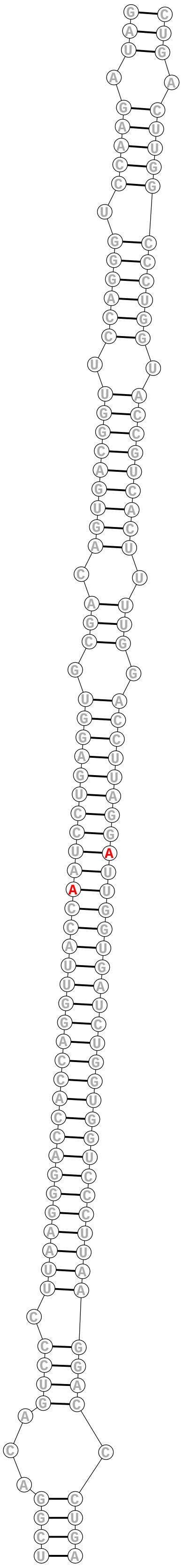

MOCS1 part A

Strands Chr23:13823948-13824122
and Chr23:13824756-13824941

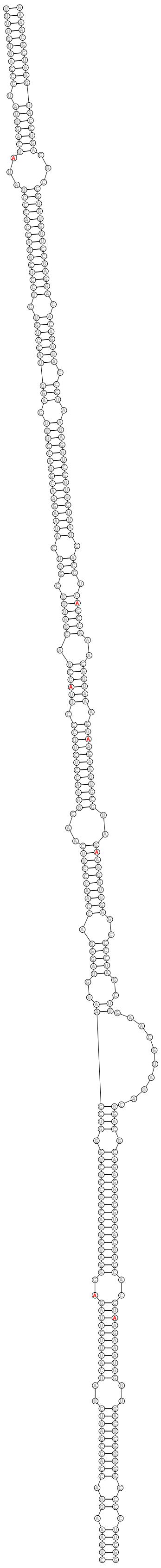

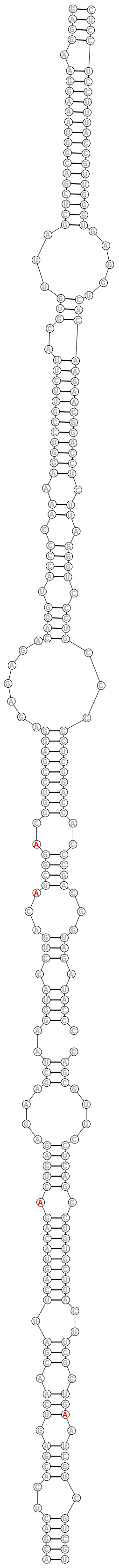

MOCS1 part B

Strands Chr23:13860698-13860828
and Chr23:13861336-13861461

MPND

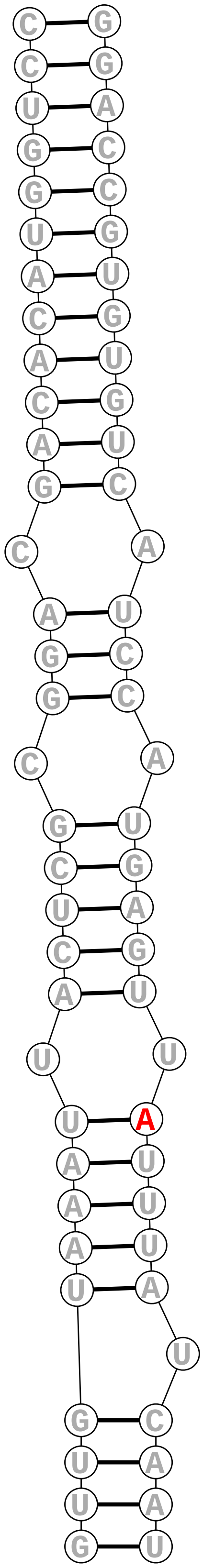

Strands Chr7:20954515-20954546
and Chr7:20955118-20955150

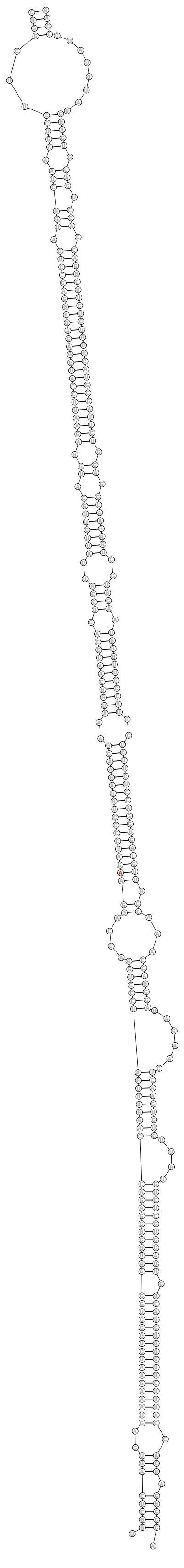

MPST part A

Strands Chr5:75819533-75819689
and Chr5:75822679-75822852

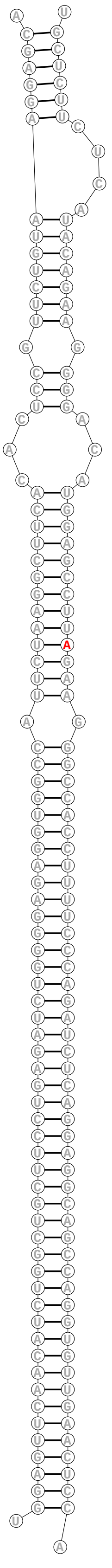

MPST part B

Strands Chr5:75824386-75824467
and Chr5:75824892-75824977

MRPL51

Strands Chr5:104275549-104275689
and Chr5:104276194-104276333

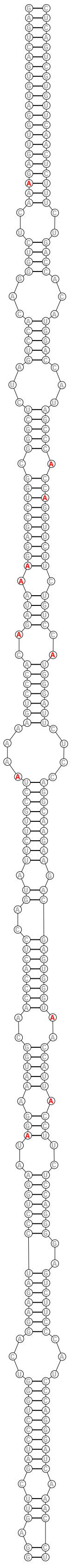

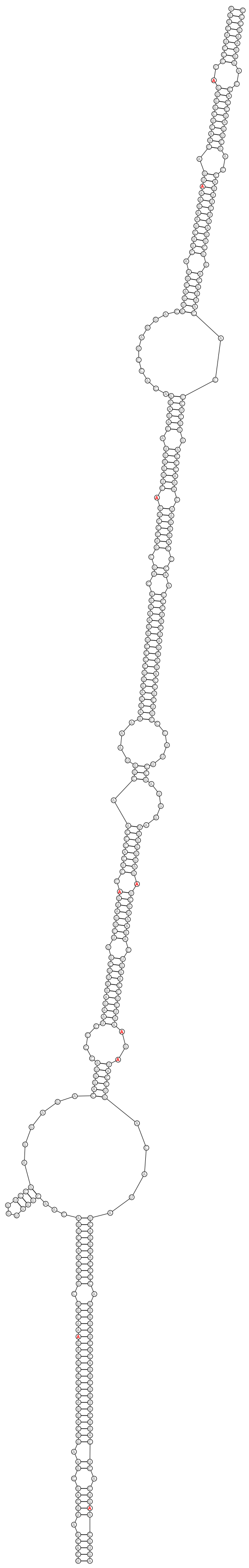

MRPS18A

Strands Chr23:17160519-17160734
and Chr23:17160833-17161027

MRPS5

Strands Chr11:1899043-1899114
and Chr11:1899708-1899789

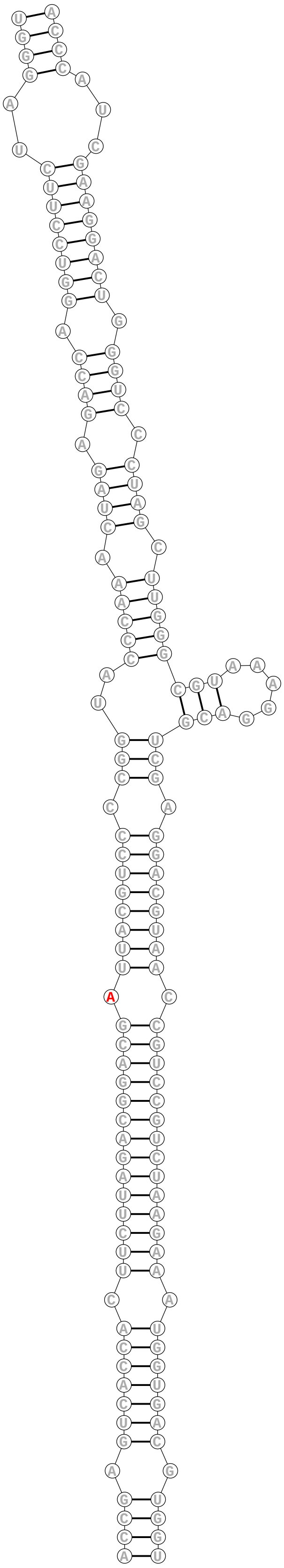

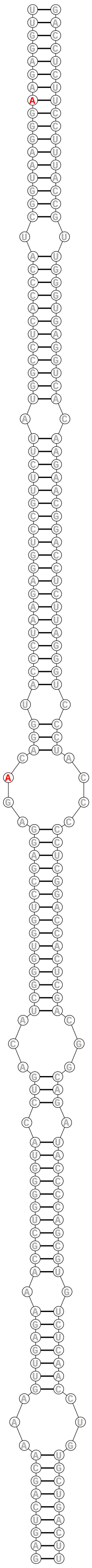

MTFR1L

Strands Chr2:127846072-127846183
and Chr2:127847480-127847591

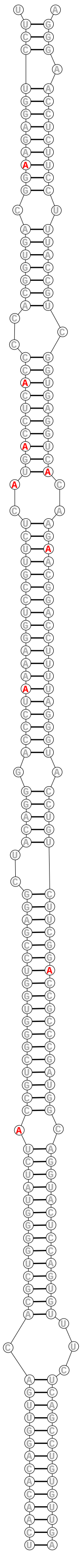

MTG1

Strands Chr26:25824130-25824239
and Chr26:25825039-25825148

MTREX part A

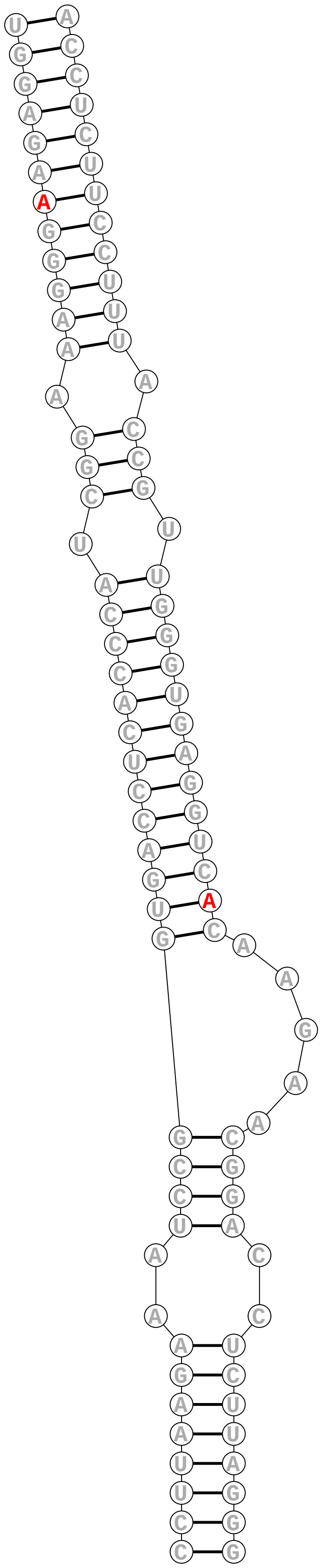

Strands Chr20:23783230-23783273
and Chr20:23784464-23784512

MTREX part B

Strands Chr20:23783167-23783227
and Chr20:23784403-23784459

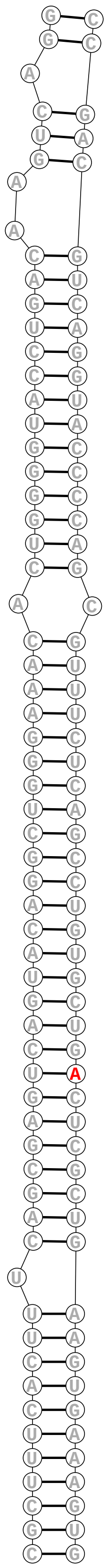

MTREX part C

Strands Chr20:23783343-23783392
and Chr20:23784464-23784517

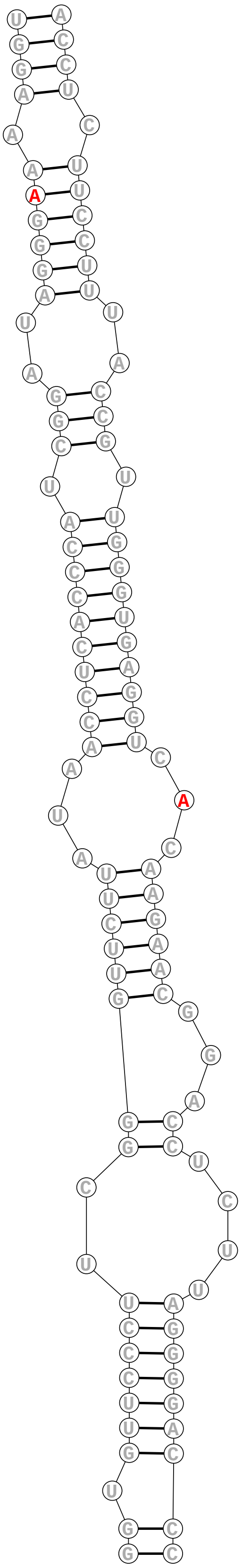

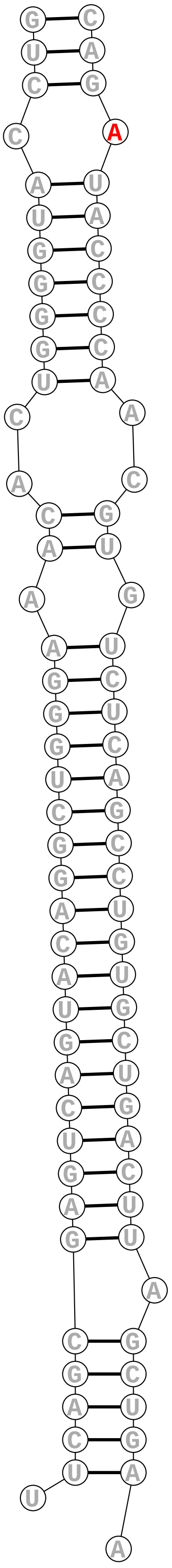

MTREX part D

Strands Chr20:23783177-23783217
and Chr20:23784541-23784582

MTRF1L

Strands Chr9:91086937-91087034
and Chr9:91087588-91087688

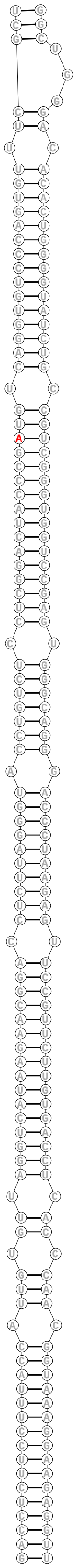

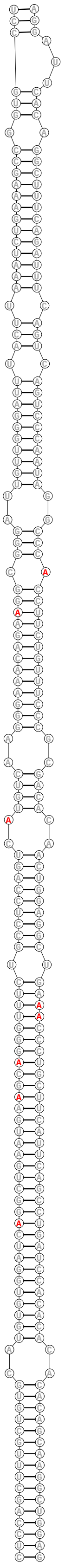

MTSS1 part A

Strands Chr14:17121359-17121480
and Chr14:17122643-17122767

MTSS1 part B

Strands Chr14:17127489-17127573
and Chr14:17127741-17127826

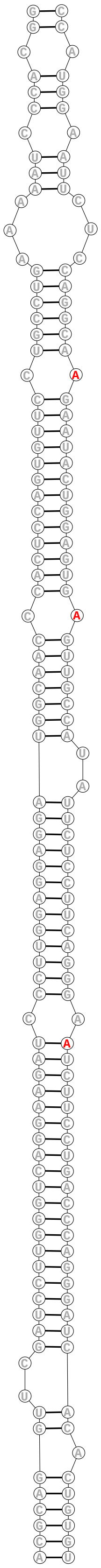

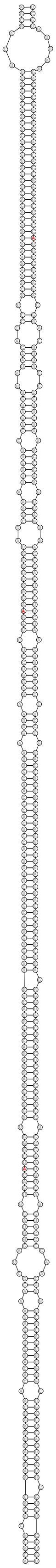

MTX2 part A

Strands Chr2:20604082-20604302
and Chr2:20605163-20605386

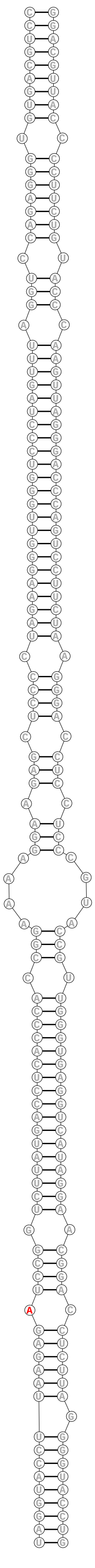

MTX2 part B

Strands Chr2:20604342-20604445
and Chr2:20605209-20605313

MYL9

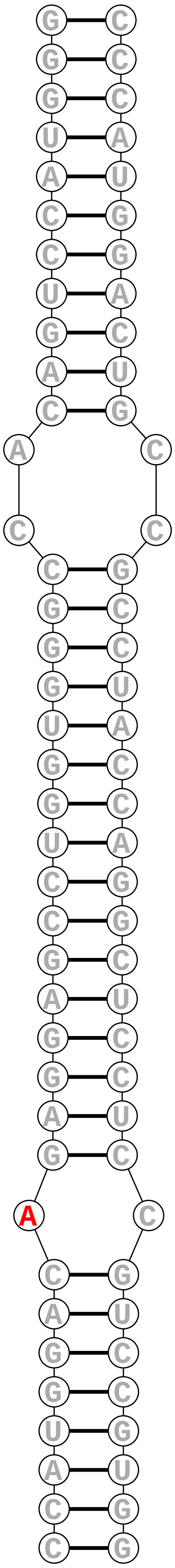

Strands Chr13:66310615-66310652
and Chr13:66311916-66311953

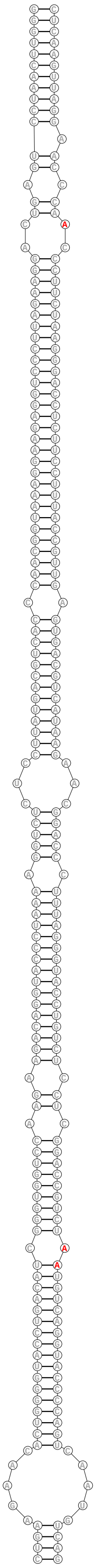

MYOC

Strands Chr16:39961019-39961145
and Chr16:39961751-39961878

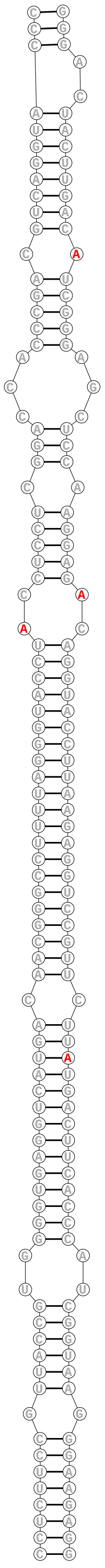

NDUFA6

Strands Chr5:113571696-113571779
and Chr5:113572764-113572849

NDUFB4

Strands Chr1:65924127-65924278
and Chr1:65925407-65925557

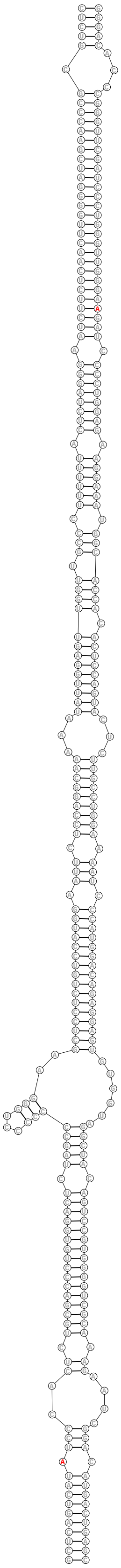

NDUFC2

Strands Chr29:18075878-18075947
and Chr29:18076430-18076498

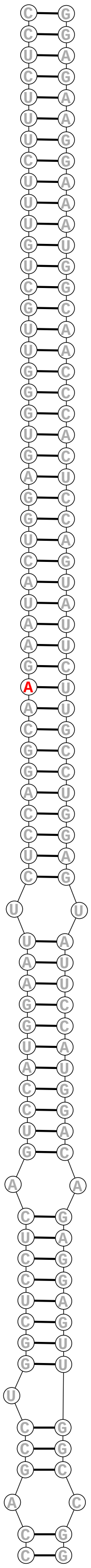

NDUFS5

Strands Chr3:107559274-107559392
and Chr3:107561479-107561612

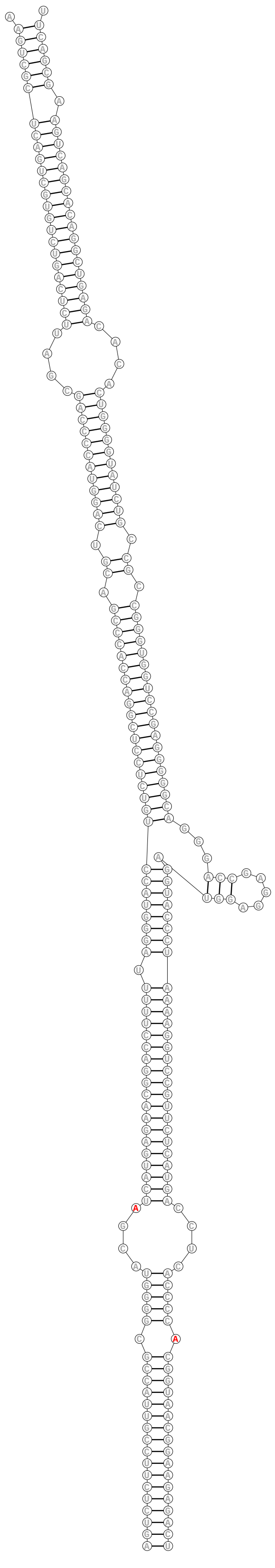

NDUFS6 part A

Strands Chr20:70989401-70989486
and Chr20:70989646-70989731

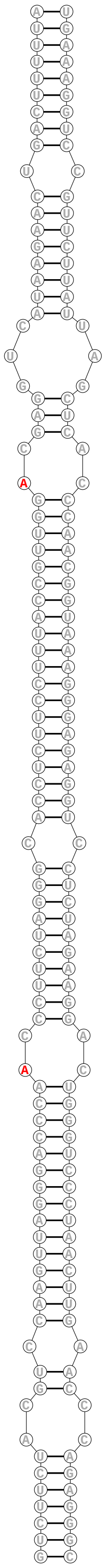

NDUFS6 part B

Strands Chr20:70989506-70989554
and Chr20:70989579-70989627

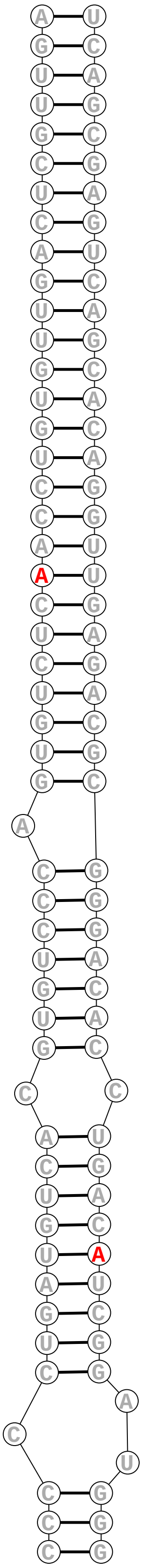

NELFA

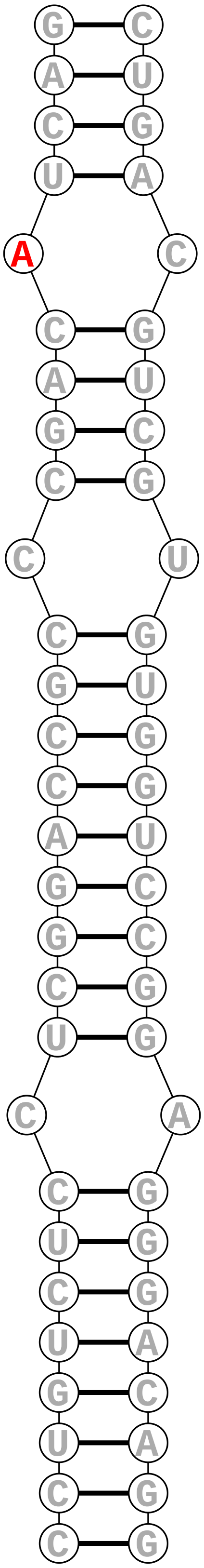

Strands Chr6:109839740-109839767
and Chr6:109839931-109839958

NHLRC3

Strands Chr12:23232517-23232595
and Chr12:23233952-23234030

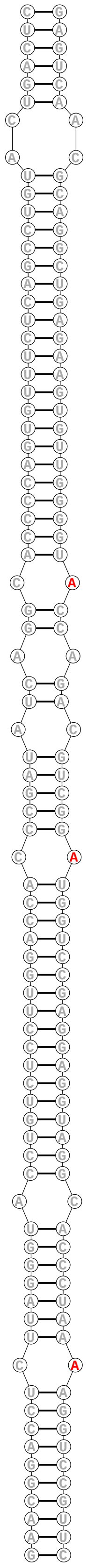

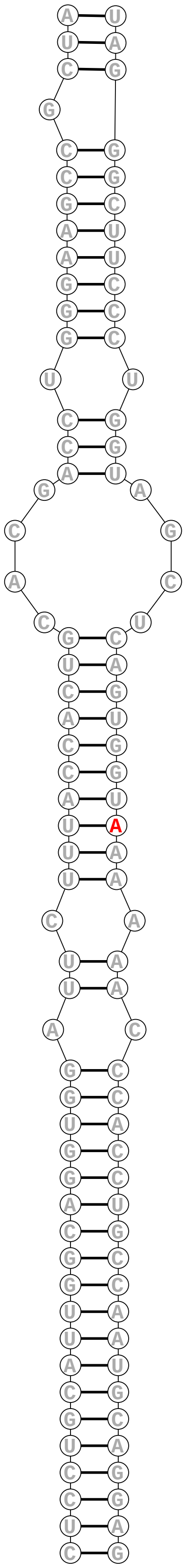

NIPAL2

Strands Chr14:68337357-68337409
and Chr14:68338820-68338871

NMI

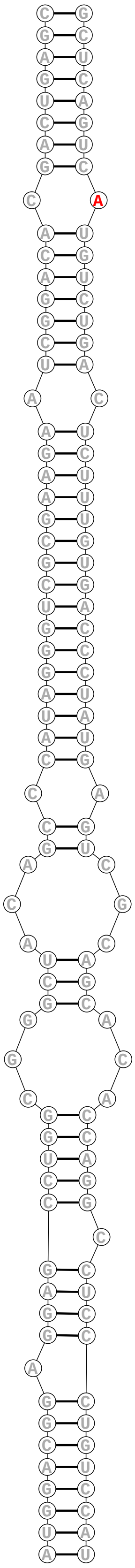

Strands Chr2:44940775-44940837
and Chr2:44948925-44948987

NMT1

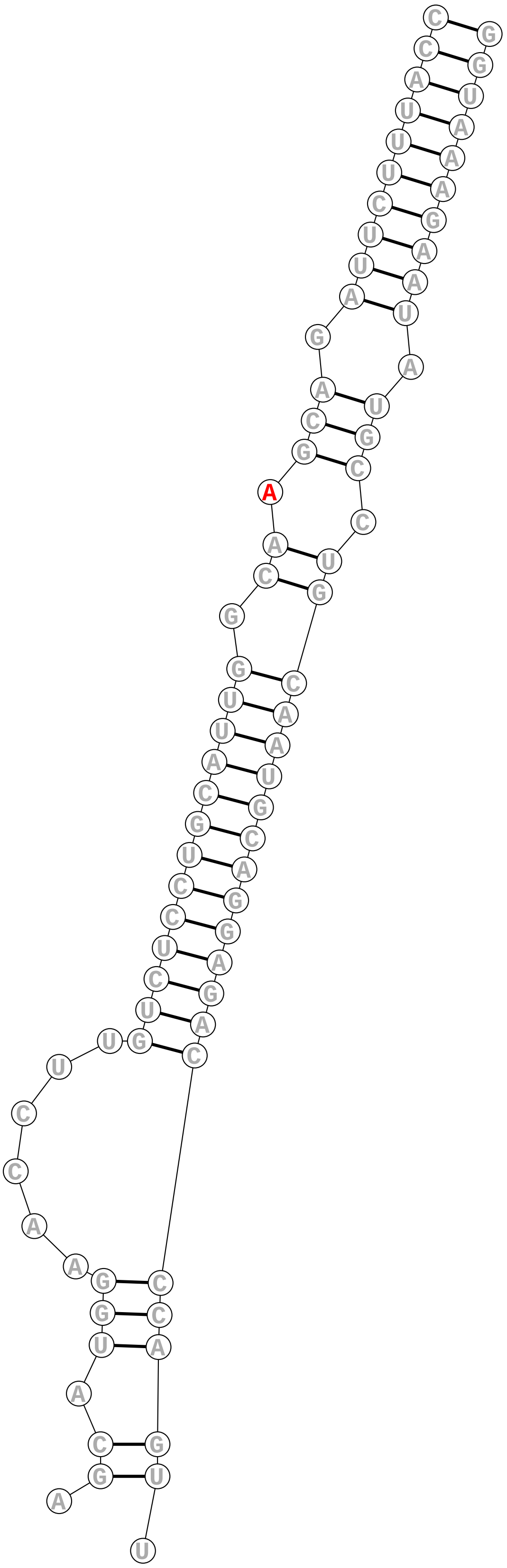

Strands Chr19:45401816-45401859
and Chr19:45401933-45401968

NOL8

Strands Chr8:85368475-85368583
and Chr8:85368650-85368759

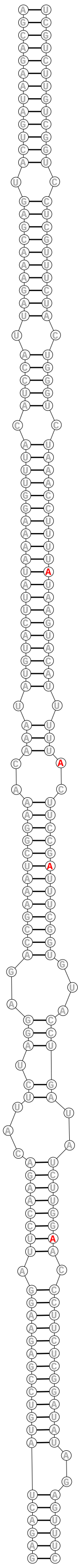

NPAS2

Strands Chr11:5999690-5999734
and Chr11:6000074-6000112

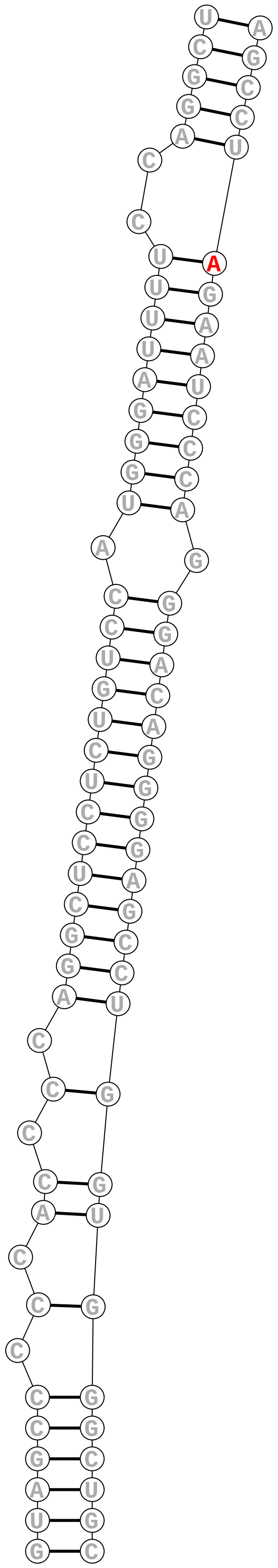

NSUN2

Strands Chr20:66736724-66736776
and Chr20:66737486-66737535

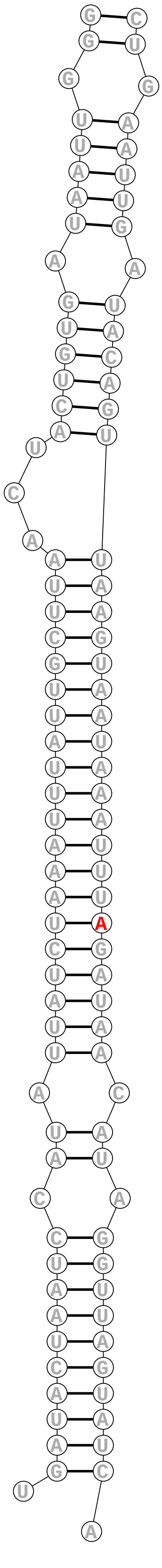

NXF1

Strands Chr29:41805231-41805296
and Chr29:41805906-41805971

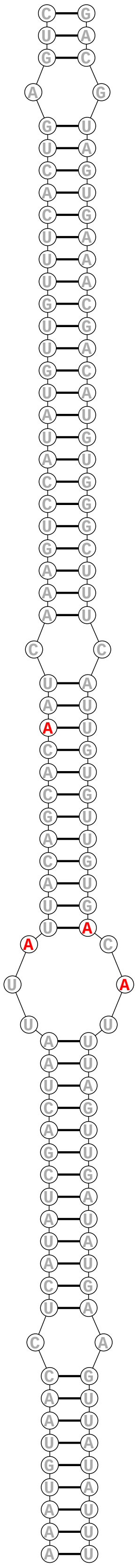

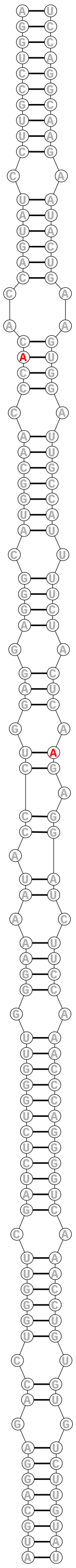

NXPE2

Strands Chr15:25522718-25522802
and Chr15:25523366-25523450

NXPE3

Strands Chr1:46500436-46500616
and Chr1:46501146-46501330

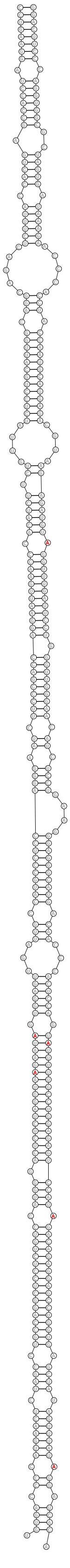

NYNRIN

Strands Chr10:20595899-20595944
and Chr10:20596085-20596131

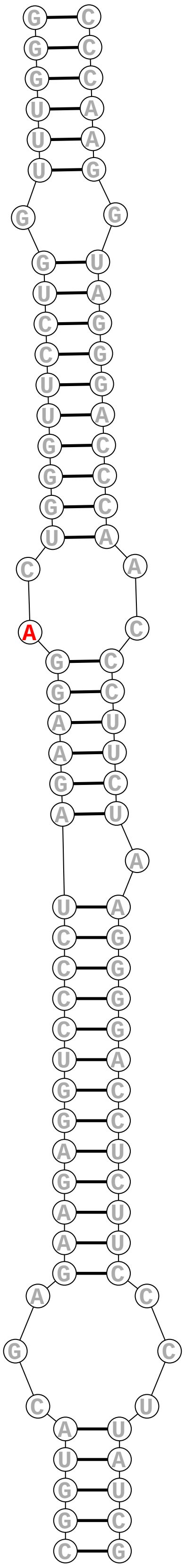

OCLN

Strands Chr20:10181301-10181352
and Chr20:10182453-10182506

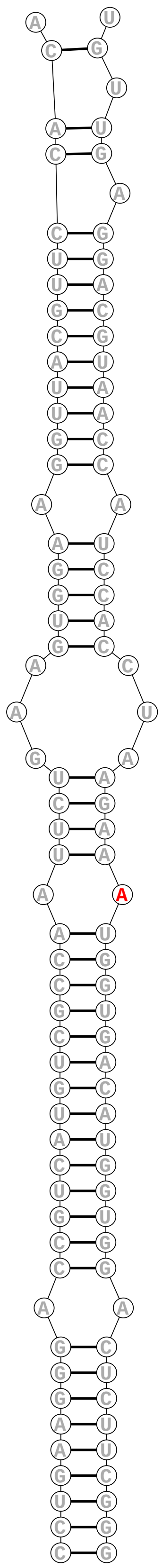

OSBPL1A part A

Strands Chr24:32894249-32894378
and Chr24:32896440-32896560

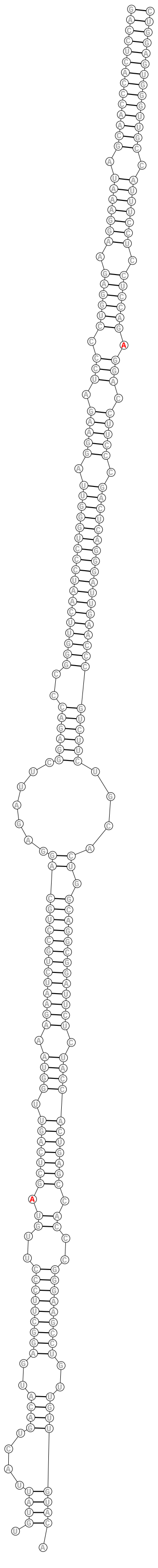

OSBPL1A part B

Strands Chr24:32894566-32894633
and Chr24:32896440-32896505

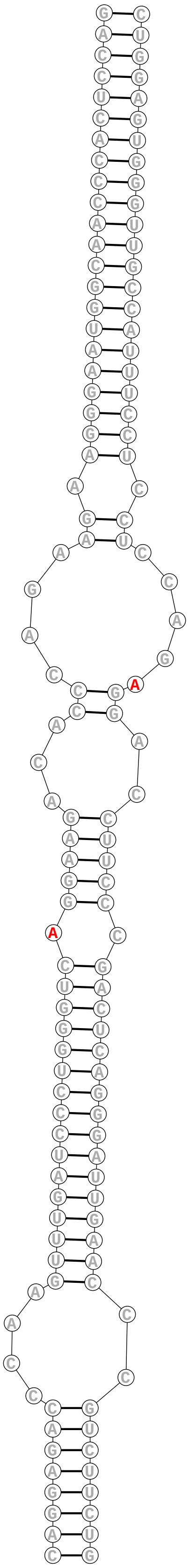

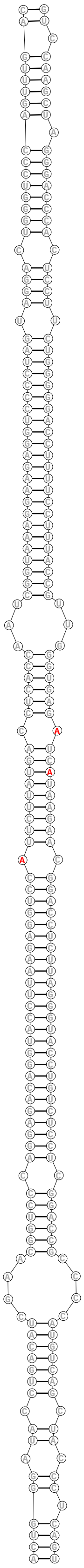

OSTC

Strands Chr6:17796743-17796860
and Chr6:17797015-17797135

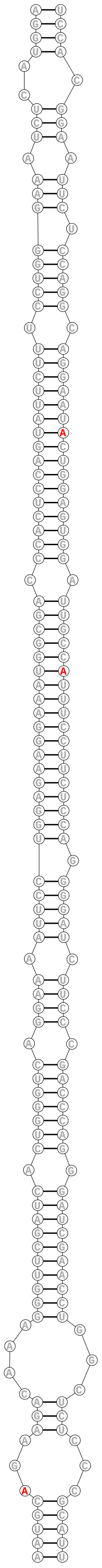

P3H1 part A

Strands Chr3:104069500-104069597
and Chr3:104070777-104070874

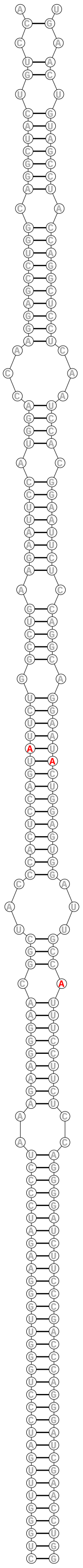

P3H1 part B

Strands Chr3:104069979-104070091
and Chr3:104070750-104070862

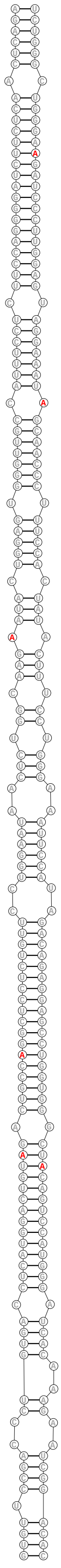

PAIP2

Strands Chr7:52263901-52264023
and Chr7:52265039-52265162

PALLD

Strands Chr8:908236-908434
and Chr8:909109-909309

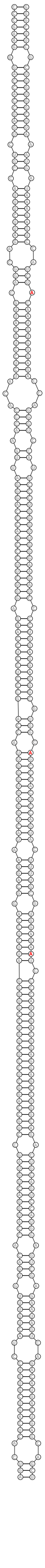

PANK2 part A

Strands Chr13:51741605-51741768
and Chr13:51741838-51742008

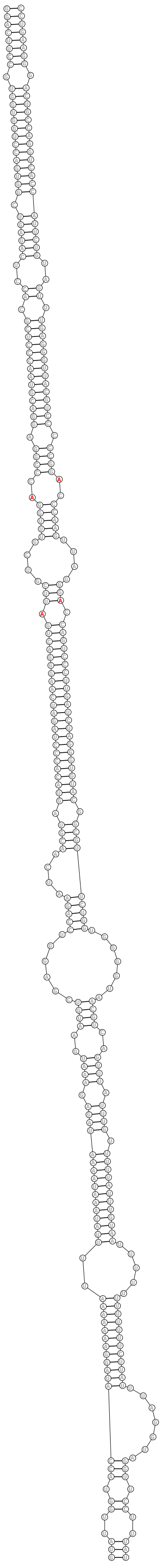

PANK2 part B

Strands Chr13:51747742-51747896
and Chr13:51750286-51750437

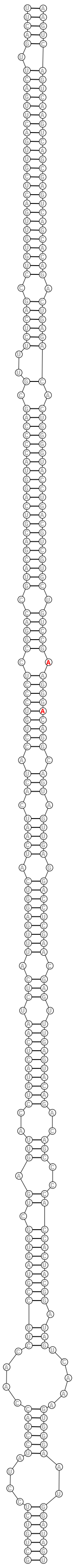

PANK2 part C

Strands Chr13:51749996-51750196
and Chr13:51751018-51751214

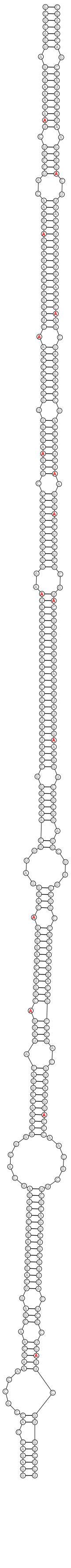

PAOX

Strands Chr26:25830231-25830299
and Chr26:25830958-25831039

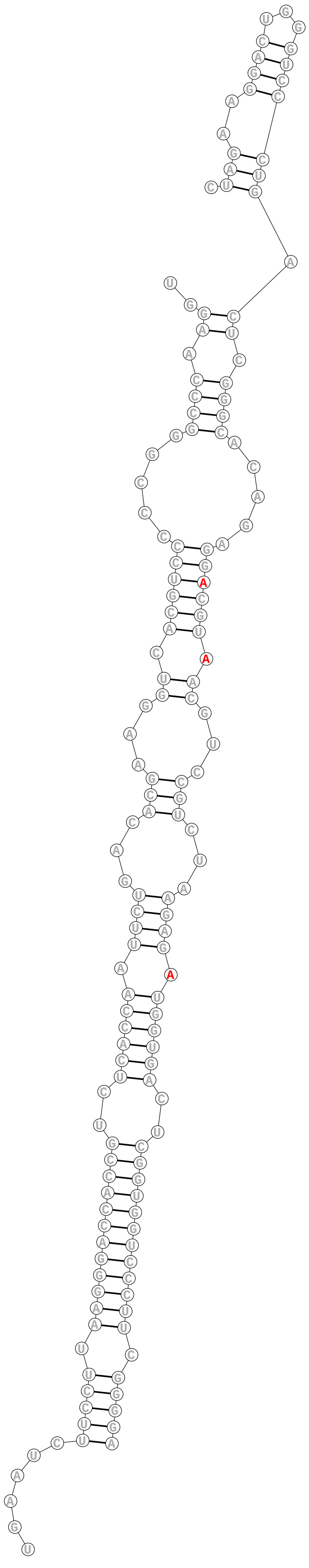

PAPOLA

Strands Chr21:63005161-63005205
and Chr21:63005867-63005908

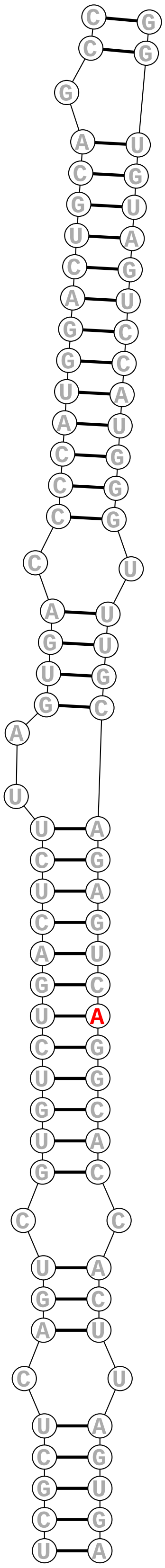

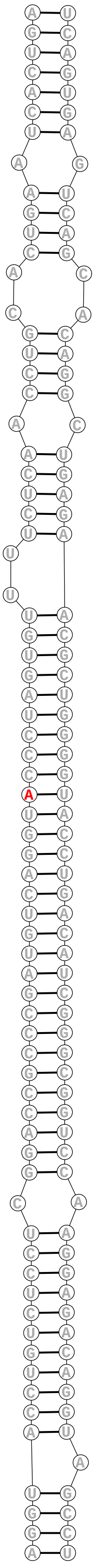

PCMTD1 part A

Strands Chr14:22691379-22691449
and Chr14:22694105-22694174

PCMTD1 part B

Strands Chr14:22691390-22691435
and Chr14:22693883-22693929

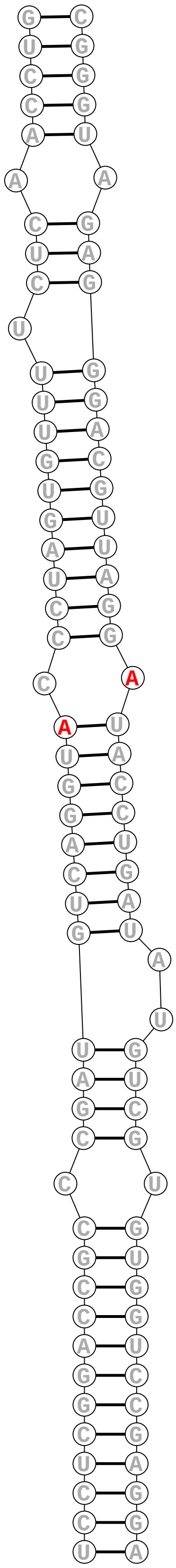

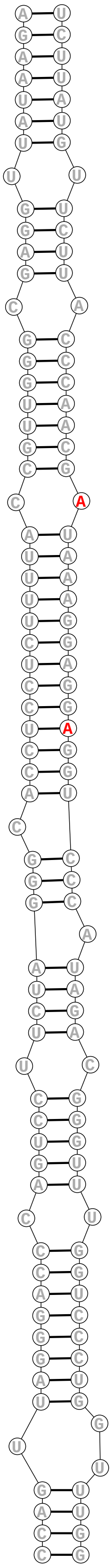

PCMTD1 part C

Strands Chr14:22691316-22691377
and Chr14:22693954-22694016

PCSK7 part A

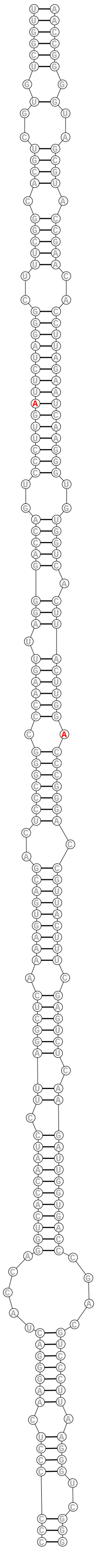

Strands Chr15:28329652-28329765
and Chr15:28331058-28331171

PCSK7 part B

Strands Chr15:28331893-28332003
and Chr15:28333033-28333152

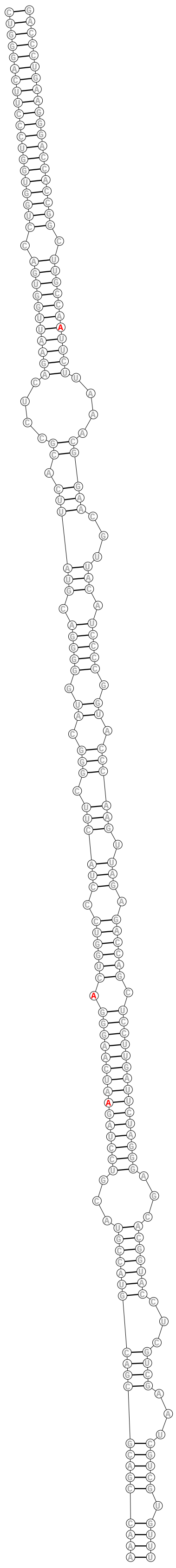

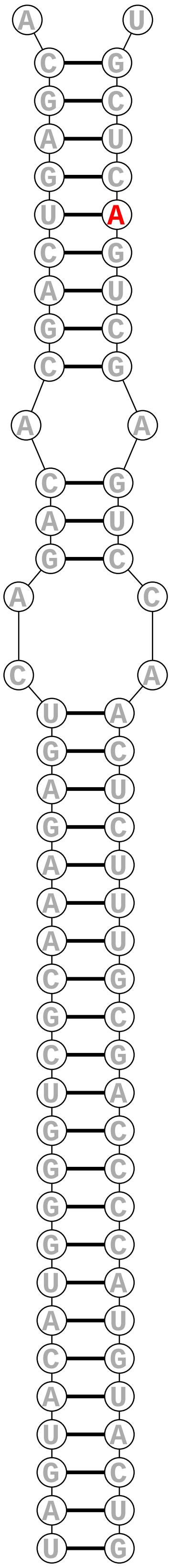

PDAP1 part A

Strands Chr25:37534055-37534093
and Chr25:37534884-37534922

PDAP1 part B

Strands Chr25:37534018-37534049
and Chr25:37534941-37534972

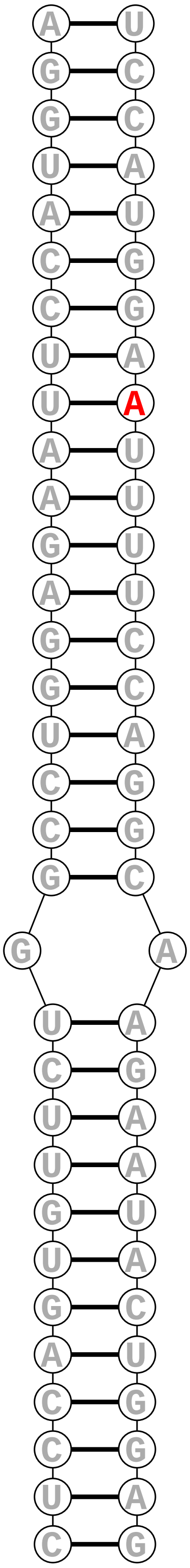

PDCD4

Strands Chr26:31726876-31726956
and Chr26:31728257-31728338

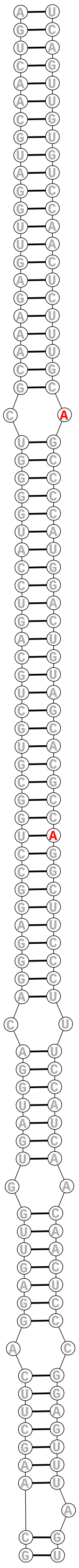

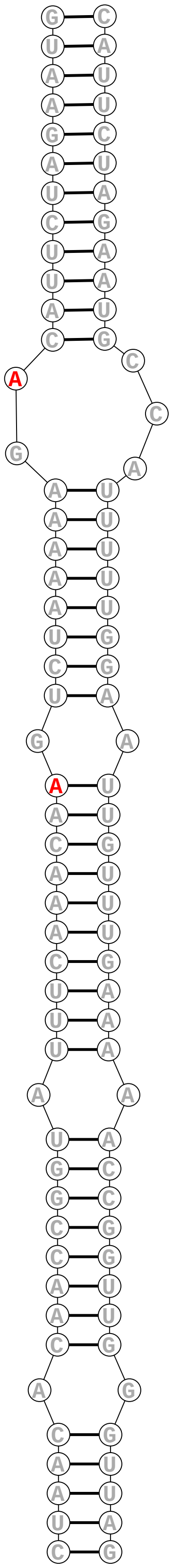

PDPN

Strands Chr16:55409180-55409227
and Chr16:55409216-55409264

PERP

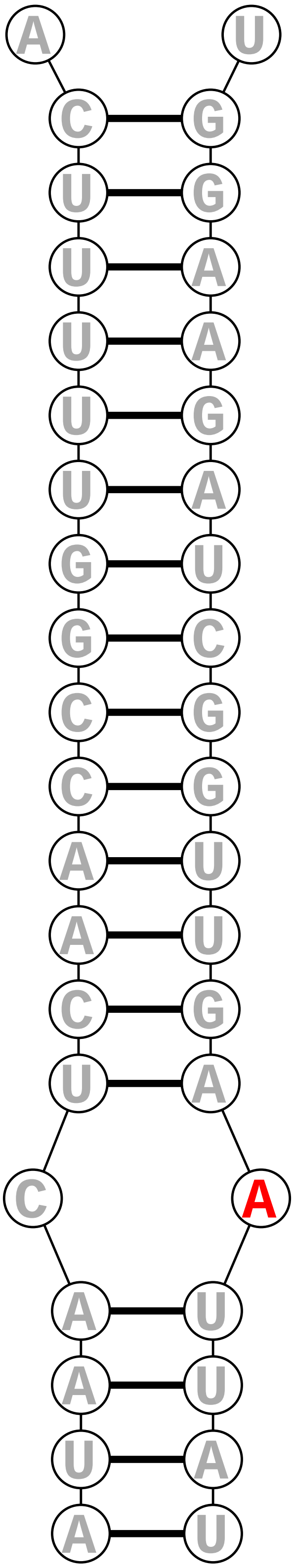

Strands Chr9:76938957-76938976
and Chr9:76939020-76939039

PEX11G part A

Strands Chr7:17575114-17575300
and Chr7:17576632-17576820

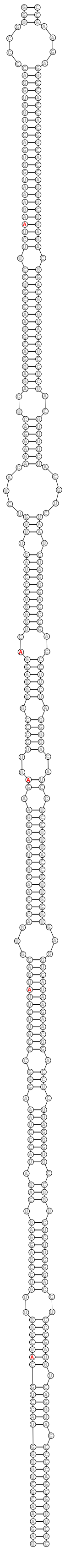

PEX11G part B

Strands Chr7:17576642-17576835
and Chr7:17577728-17577912

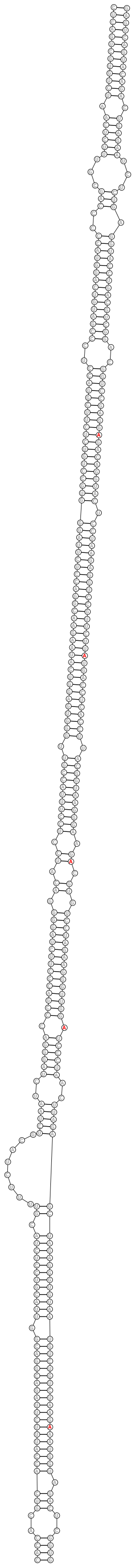

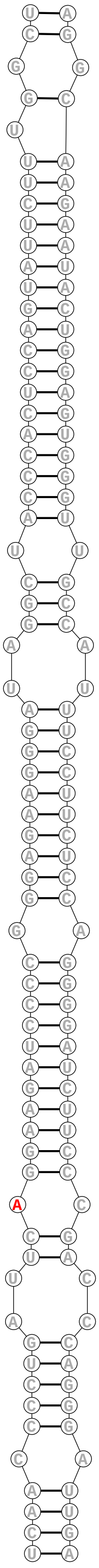

PEX3 part A

Strands Chr9:81997830-81997895
and Chr9:81998874-81998938

PEX3 part B

Strands Chr9:81997915-81998032
and Chr9:81998854-81998971

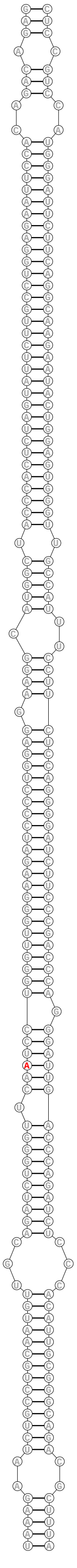

PEX6

Strands Chr23:16581574-16581721
and Chr23:16583154-16583301

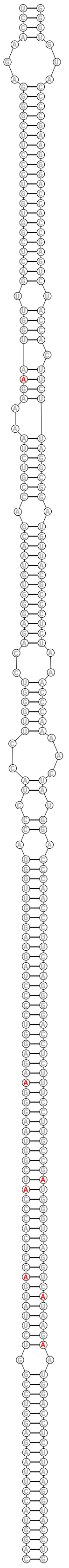

PFKFB2 part A

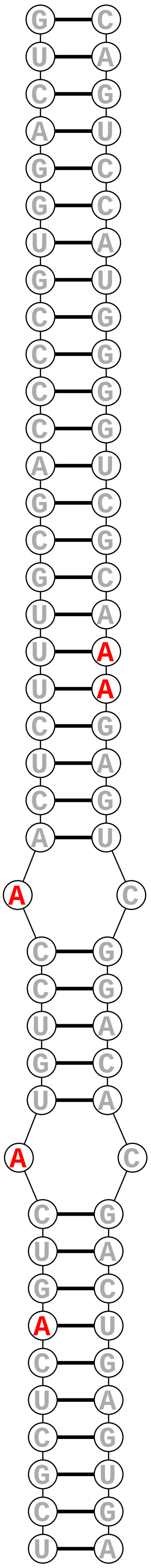

Strands Chr16:4746998-4747037
and Chr16:4747458-4747497

PFKFB2 part B

Strands Chr16:4747057-4747197
and Chr16:4747310-4747449

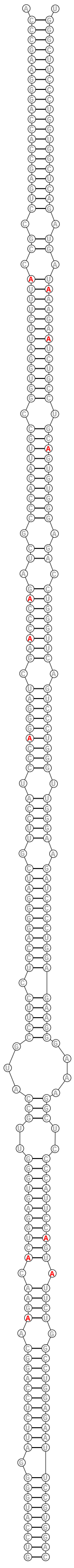

PGM2

Strands Chr6:58783135-58783295
and Chr6:58783634-58783808

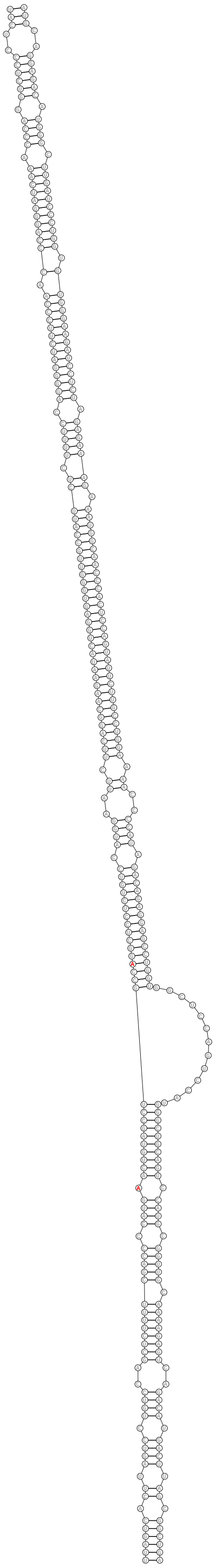

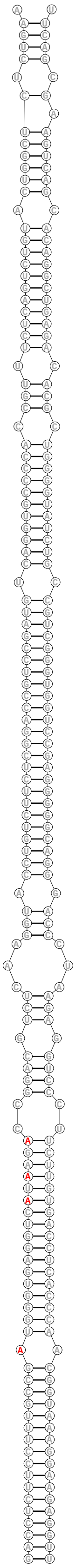

PGPEP1 part A

Strands Chr7:4764522-4764639
and Chr7:4766069-4766187

PGPEP1 part B

Strands Chr7:4765205-4765386
and Chr7:4768183-4768379

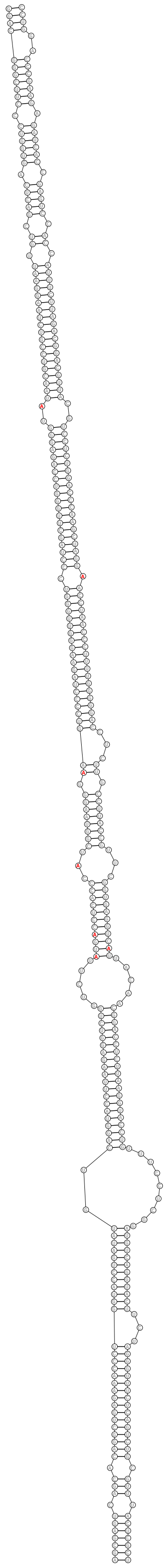

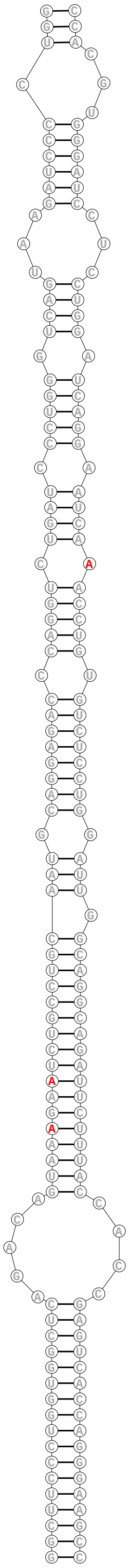

PHF11 part A

Strands Chr12:19174490-19174575
and Chr12:19176365-19176452

PHF11 part B

Strands Chr12:19174490-19174588
and Chr12:19180264-19180361

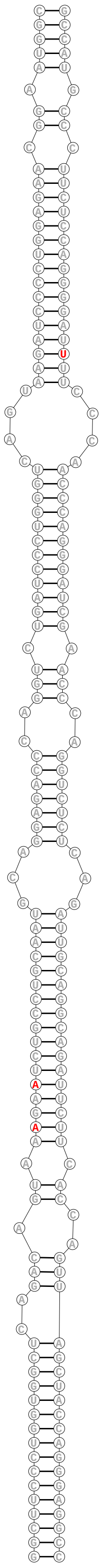

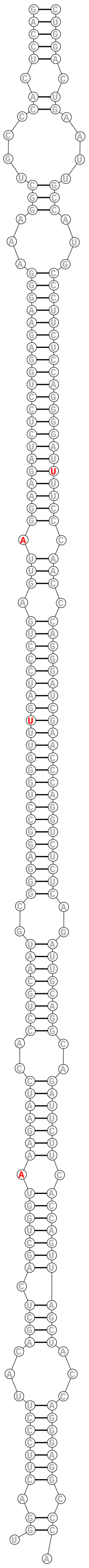

PHF11 part C

Strands Chr12:19179849-19179961
and Chr12:19180252-19180363

PHF11 part D

Strands Chr12:19179849-19179949
and Chr12:19181192-19181278

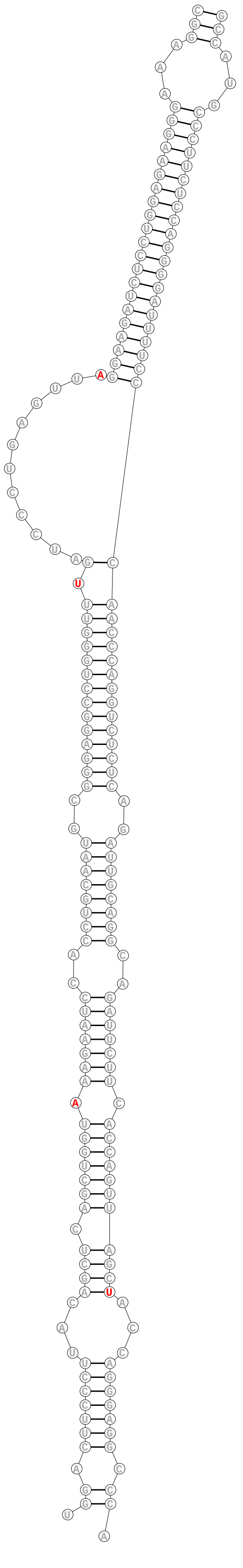

PHF5A

Strands Chr5:113080396-113080517
and Chr5:113083190-113083326

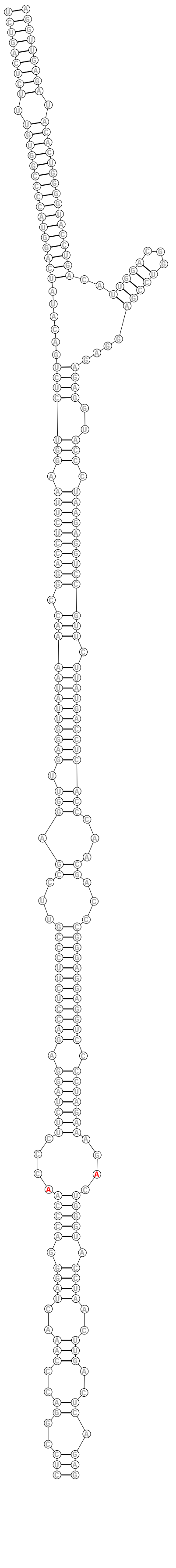

PHYHIPL

Strands Chr28:15002510-15002557
and Chr28:15002616-15002663

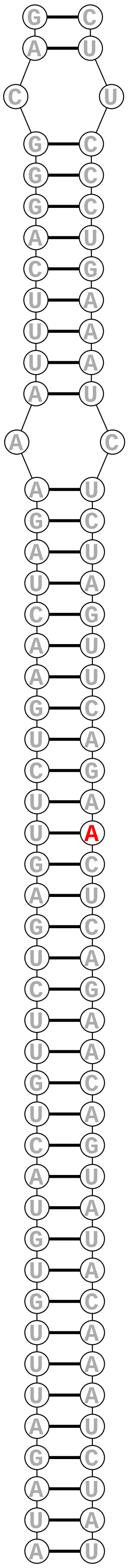

PICALM part A

Strands Chr29:9660528-9660565
and Chr29:9661081-9661117

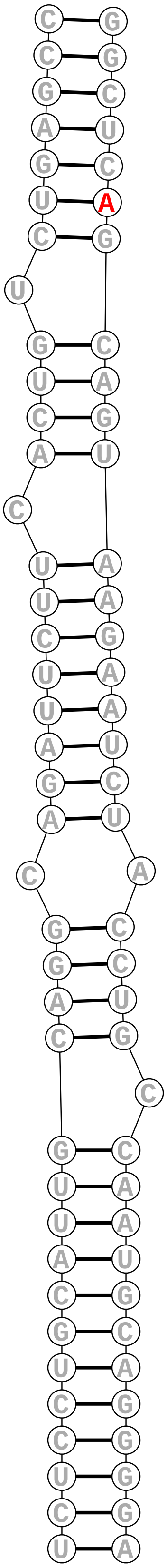

PICALM part B

Strands Chr29:9660467-9660511
and Chr29:9661134-9661180

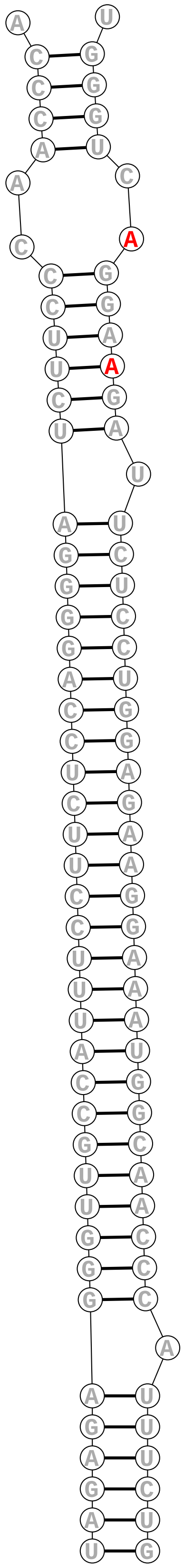

PIGS

Strands Chr19:20575330-20575393
and Chr19:20576170-20576233

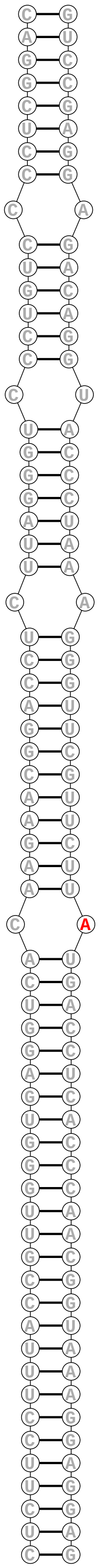

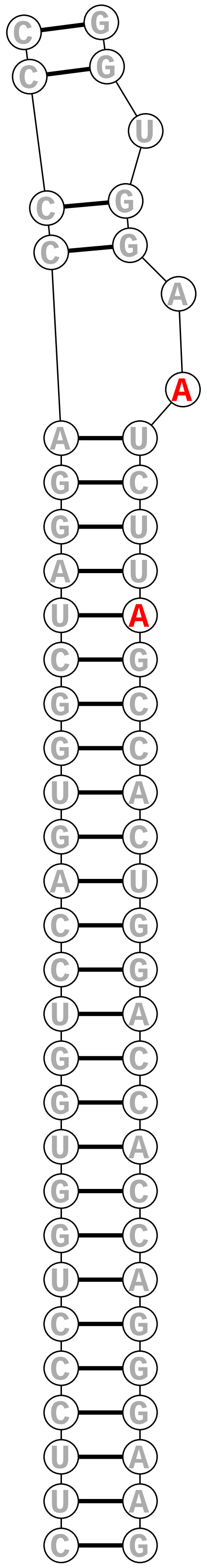

PIGT

Strands Chr13:74483165-74483194
and Chr13:74484552-74484584

PIGZ

Strands Chr1:72028470-72028536
and Chr1:72029102-72029168

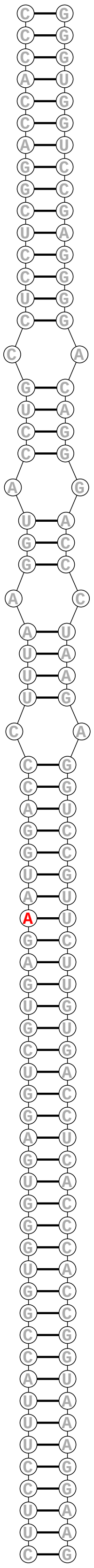

PKD2

Strands Chr6:38043911-38043998
and Chr6:38044724-38044810

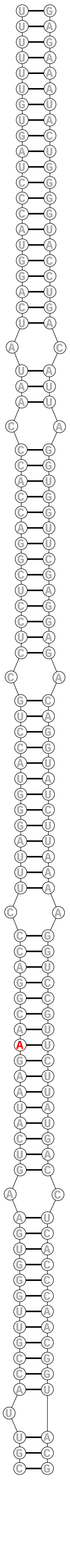

PKN1 part A

Strands Chr7:12410040-12410164
and Chr7:12411086-12411222

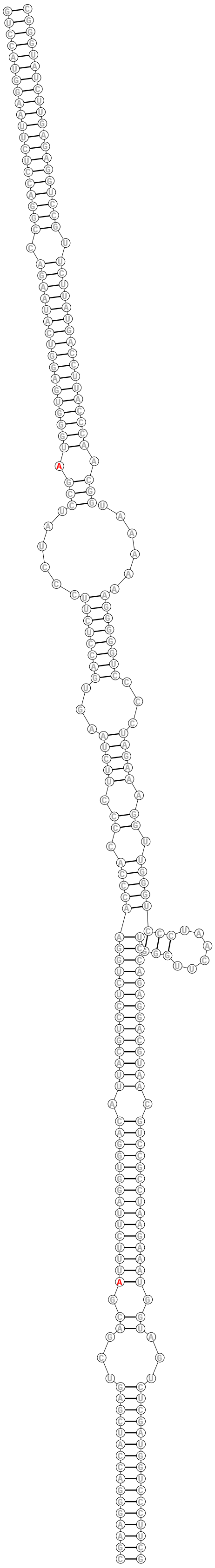

PKN1 part B

Strands Chr7:12411142-12411222
and Chr7:12412070-12412154

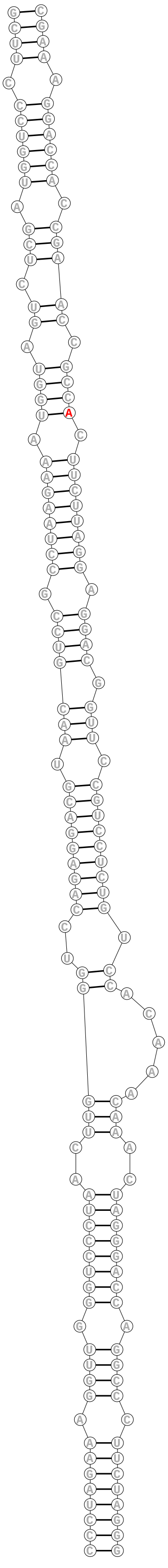

PLEKHF1

Strands Chr18:40430813-40430911
and Chr18:40431869-40431966

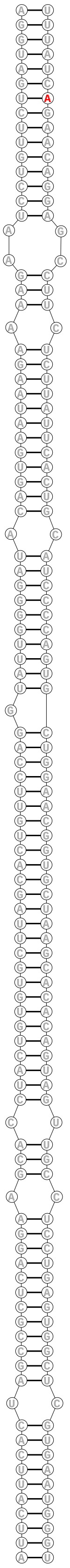

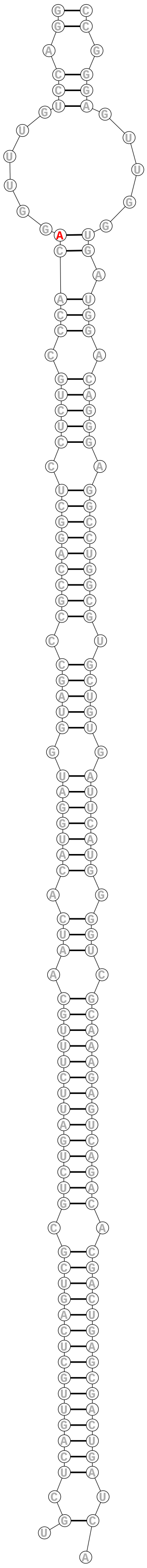

PLEKHG3

Strands Chr10:77202603-77202687
and Chr10:77203626-77203710

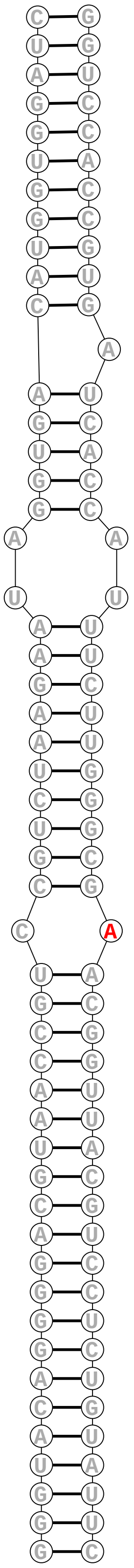

PLIN1 part A

Strands Chr21:21502597-21502646
and Chr21:21503660-21503710

PLIN1 part B

Strands Chr21:21503244-21503301
and Chr21:21503803-21503860

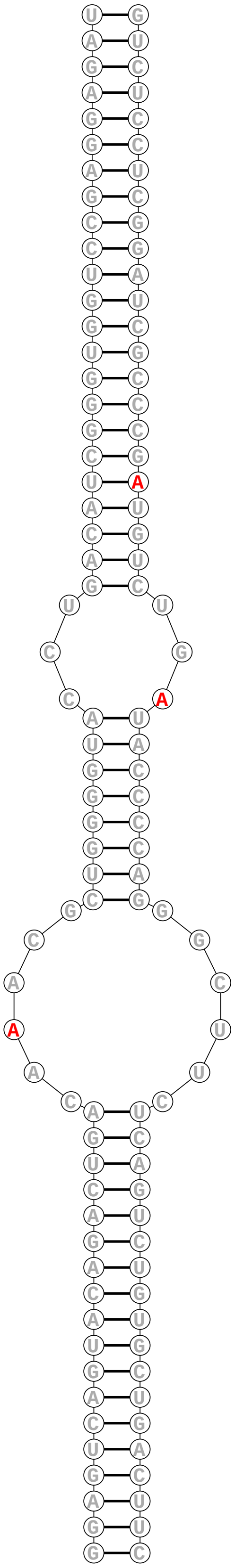

PLIN1 part C

Strands Chr21:21503309-21503355
and Chr21:21503742-21503790

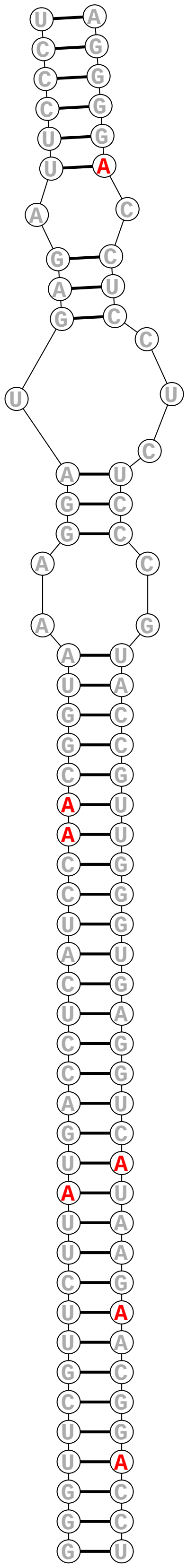

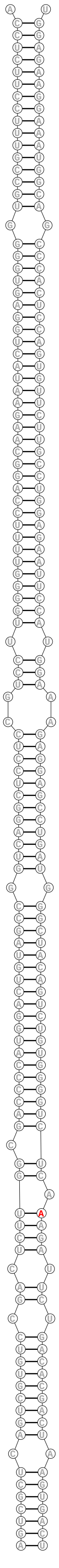

PLIN3 part A

Strands Chr7:20507564-20507679
and Chr7:20507784-20507899

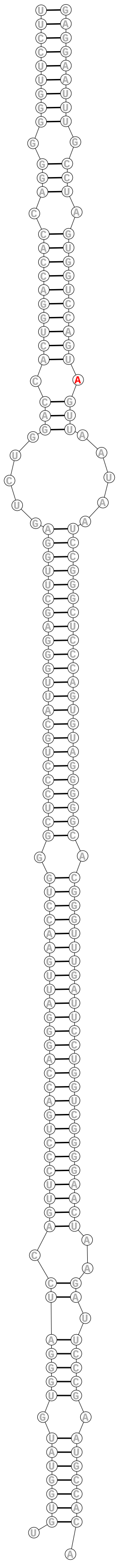

PLIN3 part B

Strands Chr7:20516643-20516742
and Chr7:20517406-20517507

PLIN3 part C

Strands Chr7:20520838-20520866
and Chr7:20522148-20522176

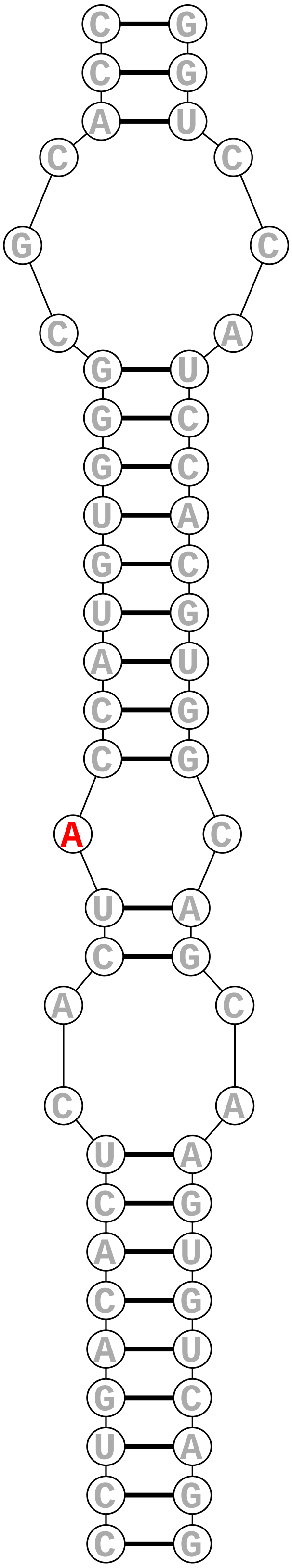

PNPLA7

Strands Chr11:105652474-105653438
and Chr11:105655116-105656058

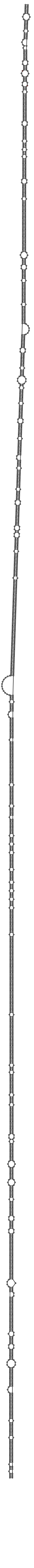

POLG2

Strands Chr19:49324748-49324902
and Chr19:49326251-49326412

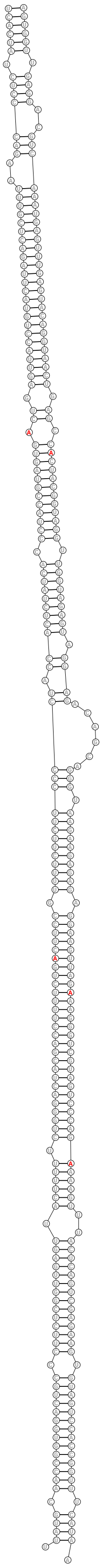

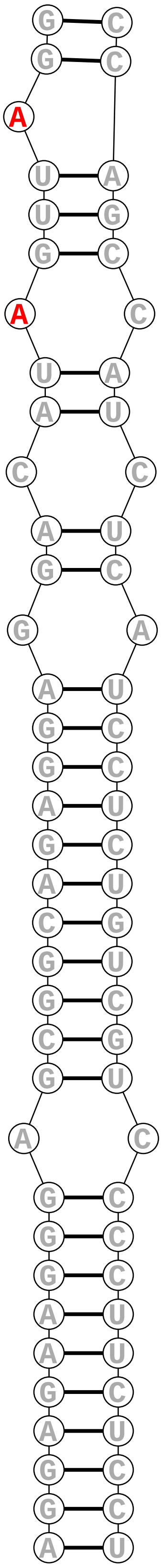

POLR2L

Strands Chr29:50690216-50690250
and Chr29:50690834-50690867

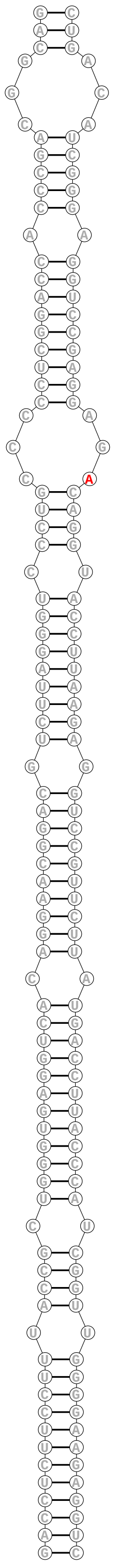

PPAT

Strands Chr6:73456717-73456796
and Chr6:73457087-73457166

PPIL2

Strands Chr17:74083921-74084053
and Chr17:74085094-74085225

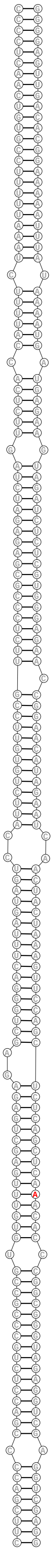

PPP6R2

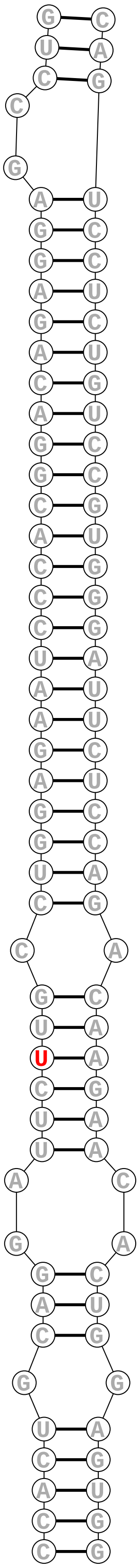

Strands Chr5:119969011-119969057
and Chr5:119969878-119969922

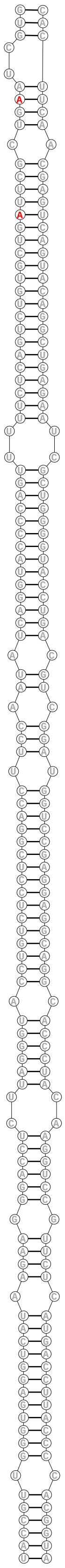

PPT2

Strands Chr23:27029439-27029549
and Chr23:27029762-27029870

PSAT1

Strands Chr8:54529216-54529243
and Chr8:54529246-54529273

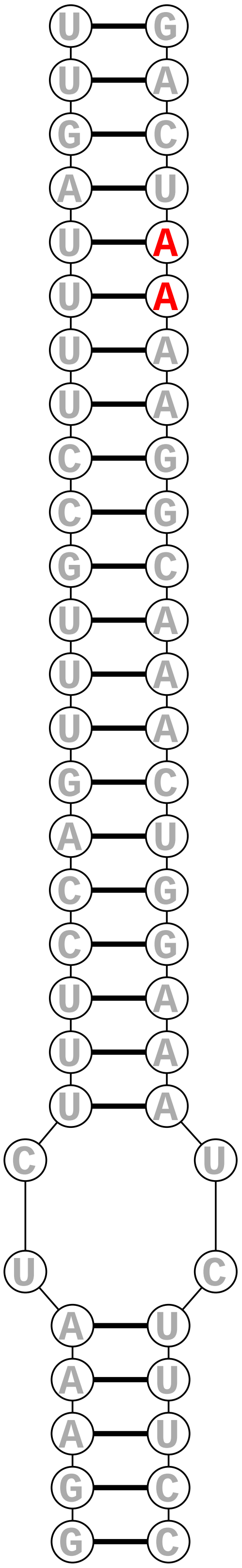

PSMC6

Strands Chr10:11403527-11403649
and Chr10:11404805-11404927

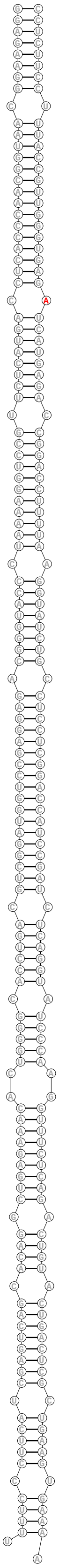

PSPC1 part A

Strands Chr12:36575192-36575285
and Chr12:36576164-36576257

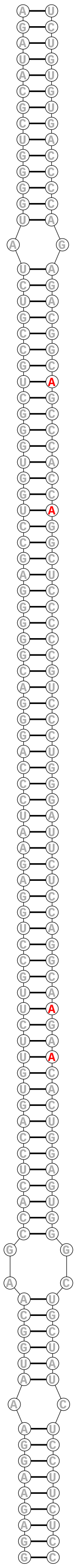

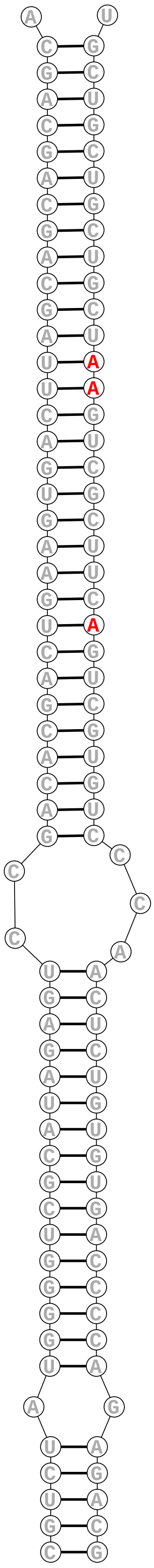

PSPC1 part B

Strands Chr12:36575291-36575346
and Chr12:36576127-36576183

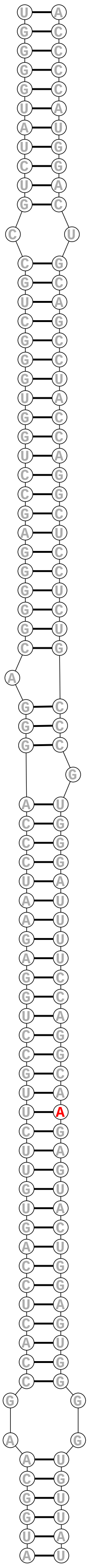

PSPC1 part C

Strands Chr12:36575202-36575277
and Chr12:36576316-36576391

PTK2B

Strands Chr8:75838556-75838769
and Chr8:75838780-75838992

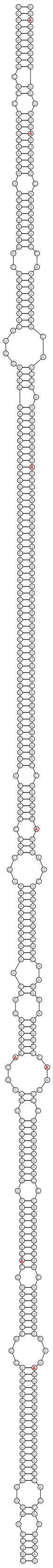

PTK7

Strands Chr23:16735118-16735363
and Chr23:16736410-16736595

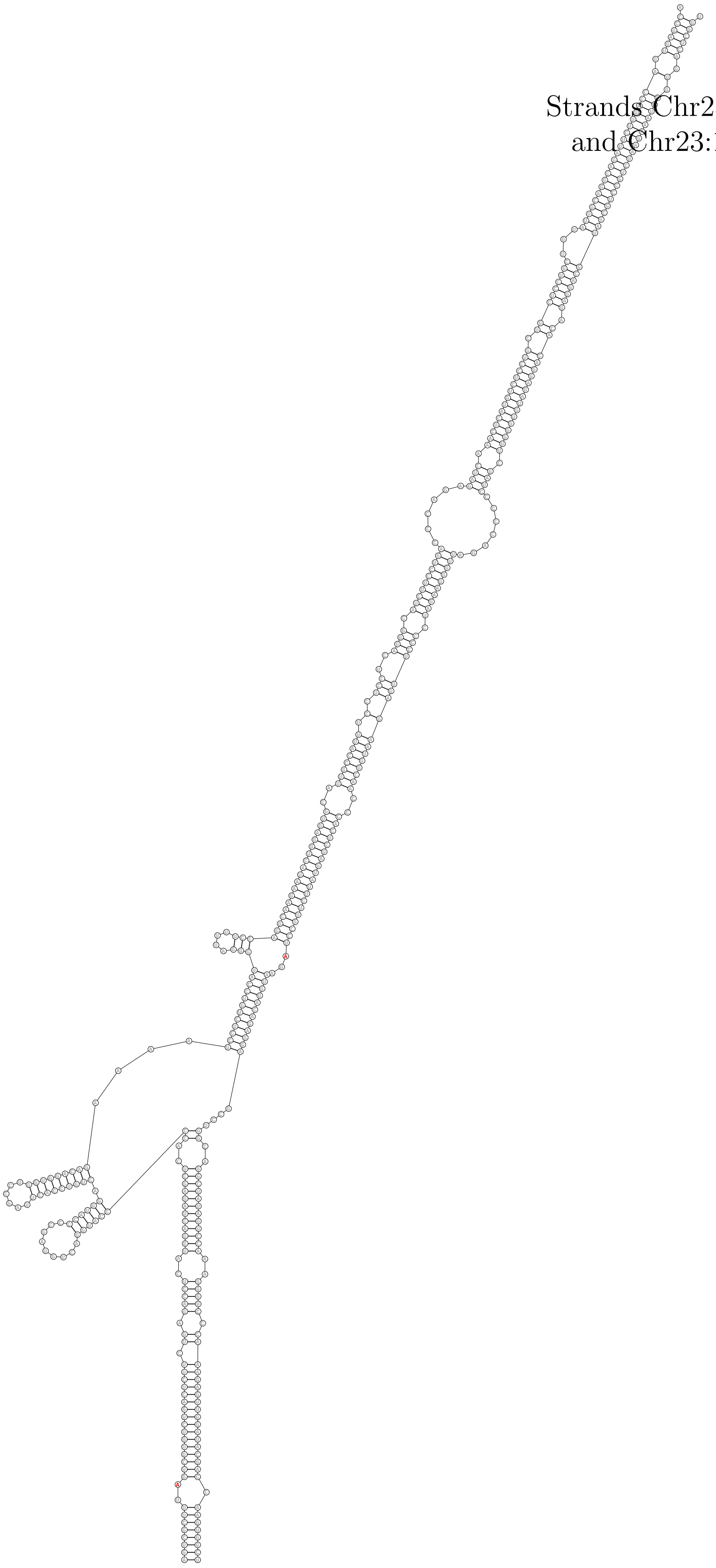

PTPRM

Strands Chr24:41408491-41408551
and Chr24:41409067-41409129

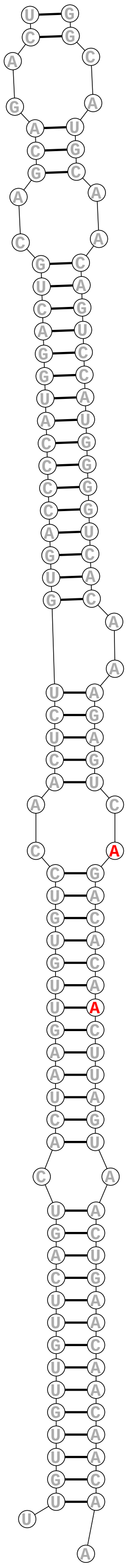

QDPR

Strands Chr6:117741087-117741207
and Chr6:117742476-117742600

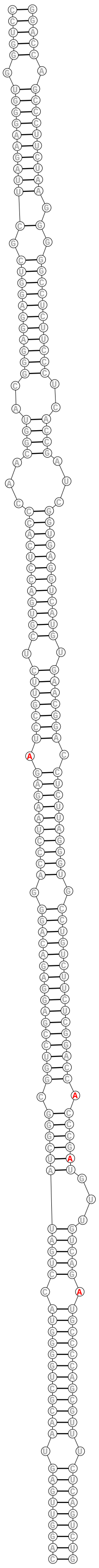

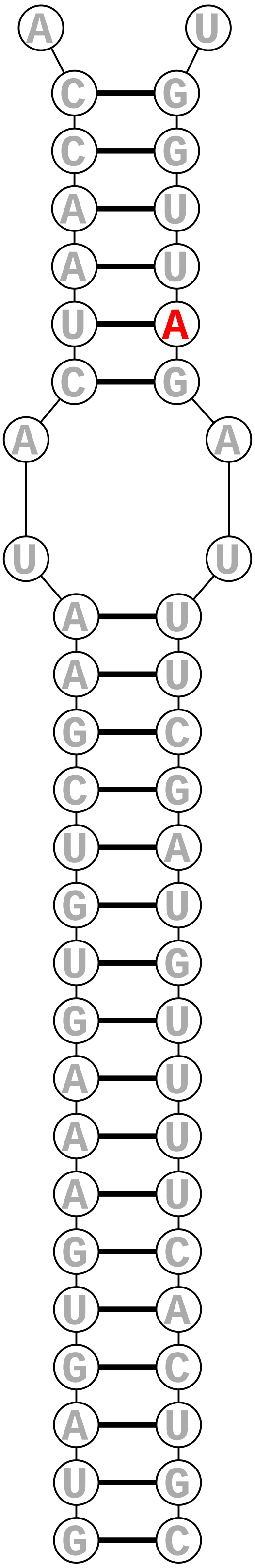

QRICH1

Strands Chr22:51528019-51528044
and Chr22:51528329-51528354

QSOX1

Strands Chr16:62825397-62825581
and Chr16:62827279-62827454

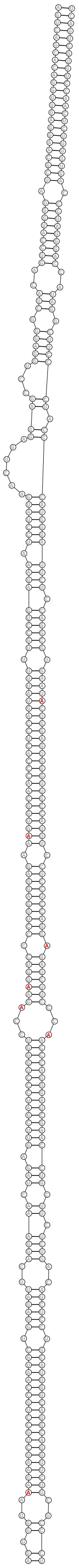

RAB12

Strands Chr24:41533758-41533868
and Chr24:41534953-41535062

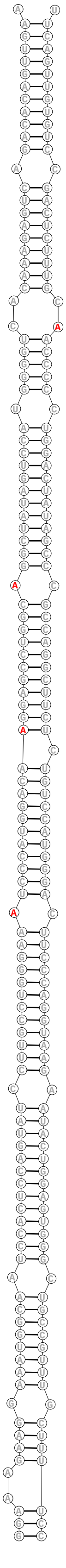

RAB18 part A

Strands Chr13:37329529-37329626
and Chr13:37330353-37330435

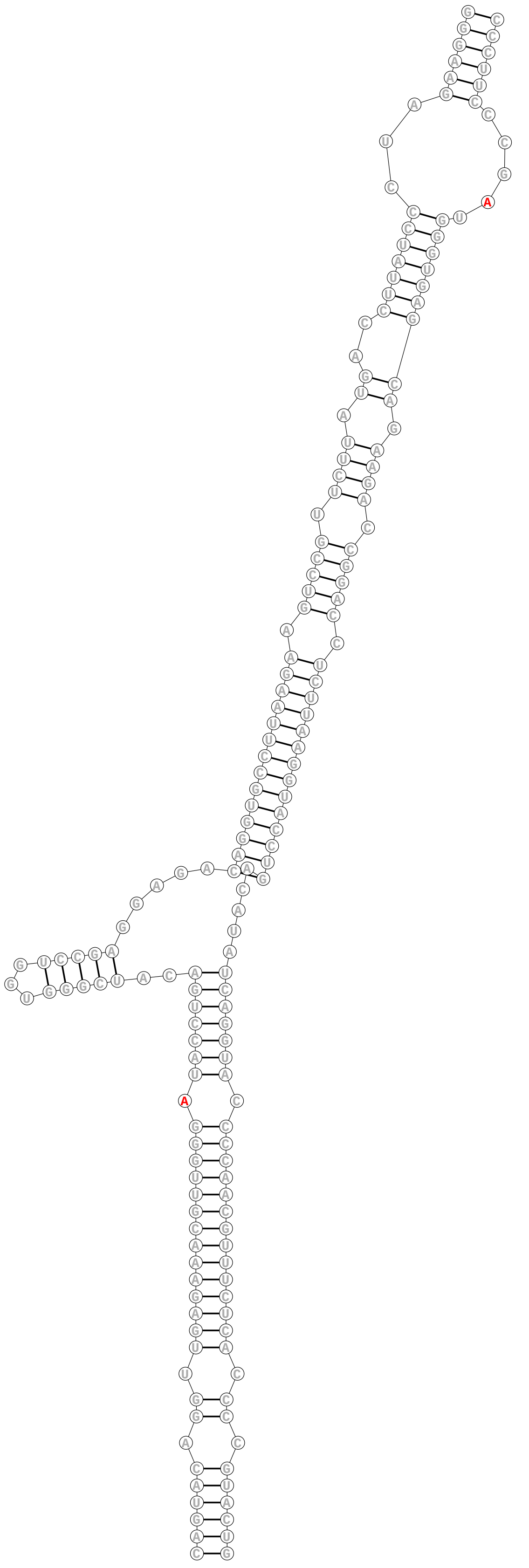

RAB18 part B

Strands Chr13:37329803-37329942
and Chr13:37330261-37330398

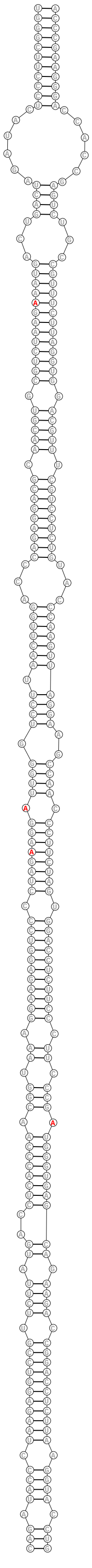

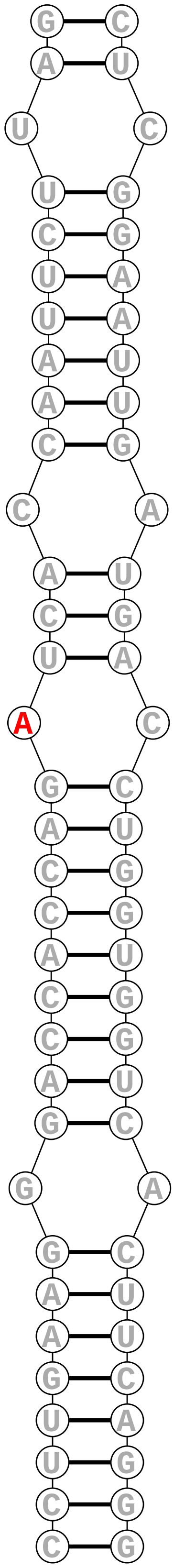

RAB3D

Strands Chr7:16963048-16963080
and Chr7:16964458-16964490

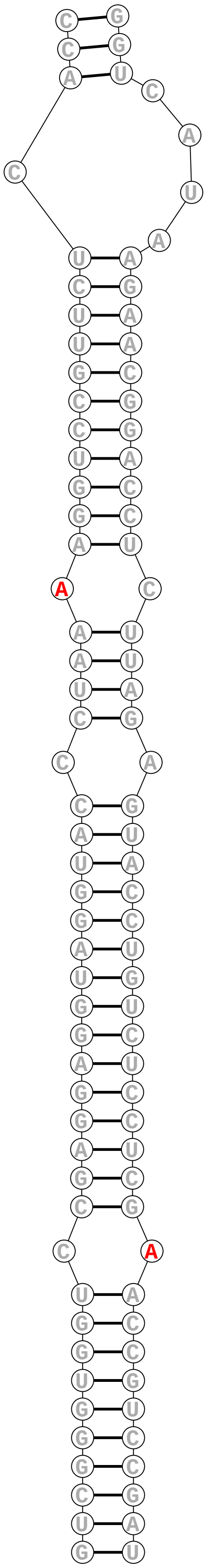

RAB3IP part A

Strands Chr5:43897022-43897068
and Chr5:43897439-43897488

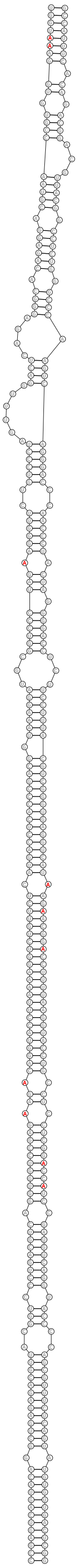

RAB3IP part B

Strands Chr5:43932842-43933021
and Chr5:43933873-43934047

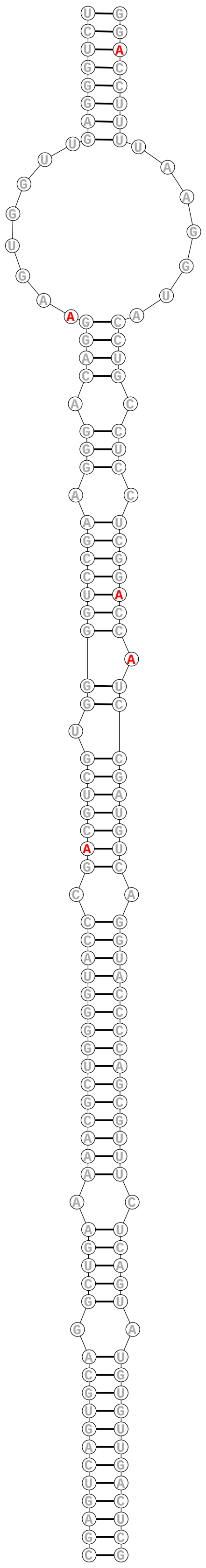

RAB3IP part C

Strands Chr5:43933523-43933599
and Chr5:43933971-43934046

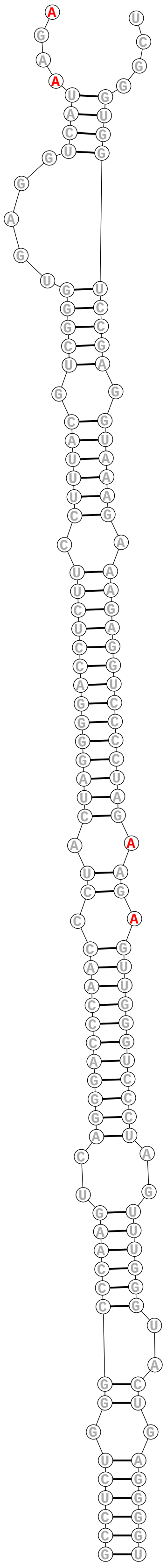

RAB3IP part D

Strands Chr5:43933897-43933968
and Chr5:43935162-43935230

RAB3IP part E

Strands Chr5:43933935-43934046
and Chr5:43935301-43935414

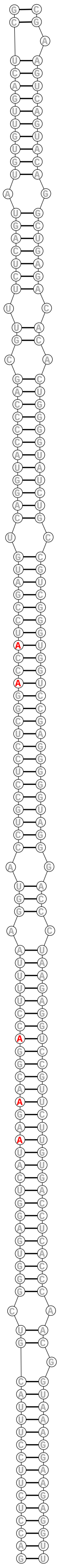

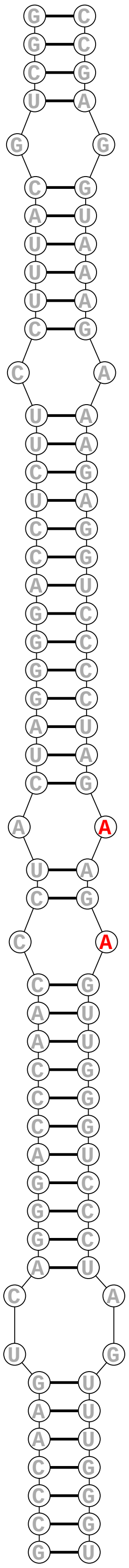

RAB3IP part F

Strands Chr5:43933905-43933954
and Chr5:43935171-43935220

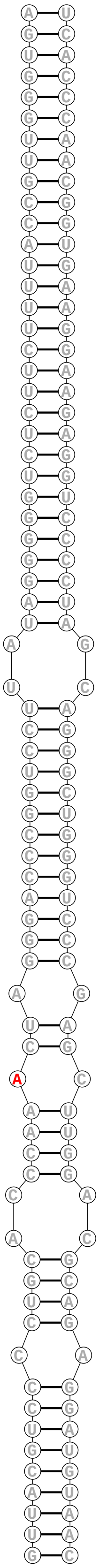

RAB8A part A

Strands Chr7:7908542-7908610
and Chr7:7909478-7909546

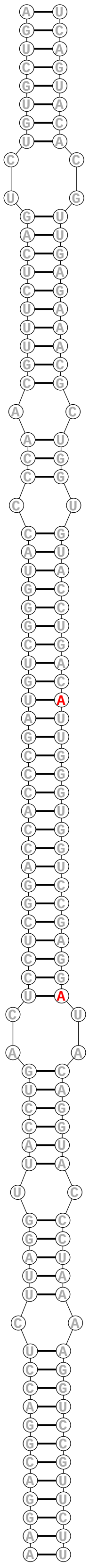

RAB8A part B

Strands Chr7:7908617-7908694
and Chr7:7909355-7909432

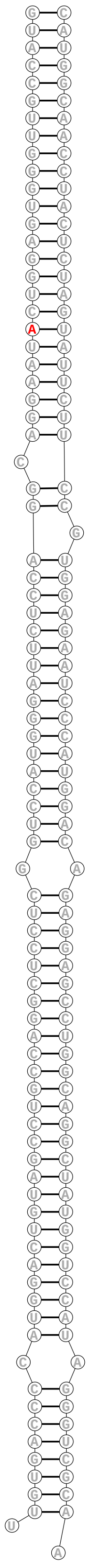

RAD51C

Strands Chr19:9916945-9917026
and Chr19:9917332-9917413

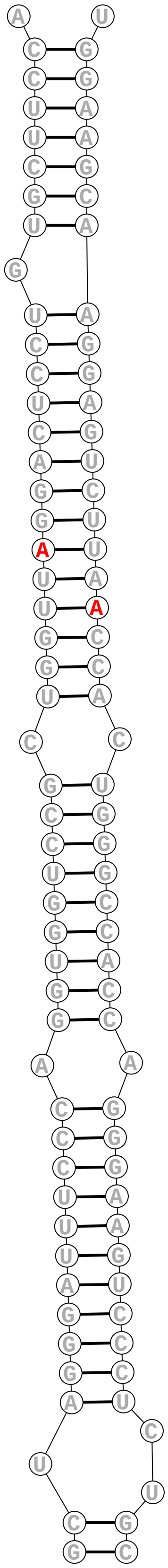

RAD51D part A

Strands Chr19:15300628-15300675
and Chr19:15301299-15301346

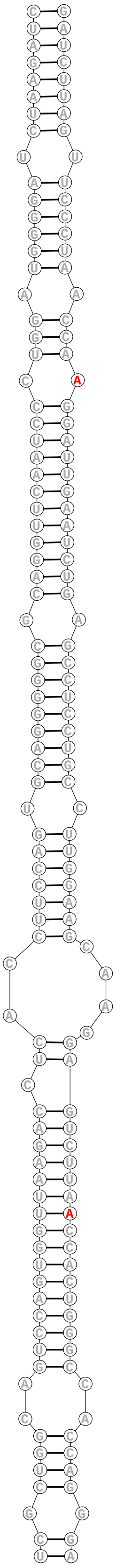

RAD51D part B

Strands Chr19:15300369-15300448
and Chr19:15301255-15301335

RAMP3

Strands Chr4:77171361-77171462
and Chr4:77172835-77172937

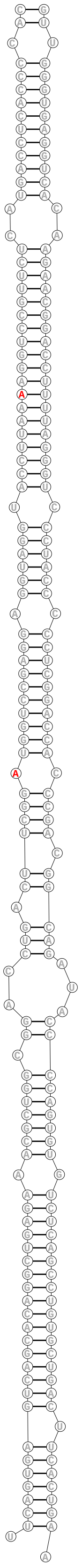

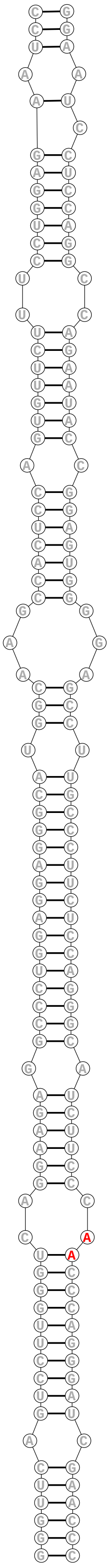

RANBP9

Strands Chr23:42788832-42788909
and Chr23:42789457-42789535

RASSF4

Strands Chr28:44945642-44945741
and Chr28:44946676-44946775

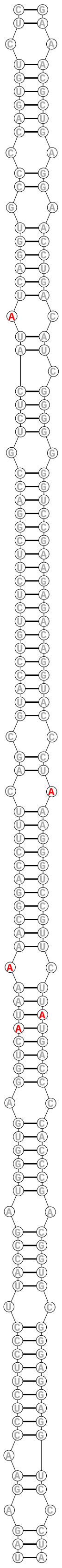

RBBP4RB part A

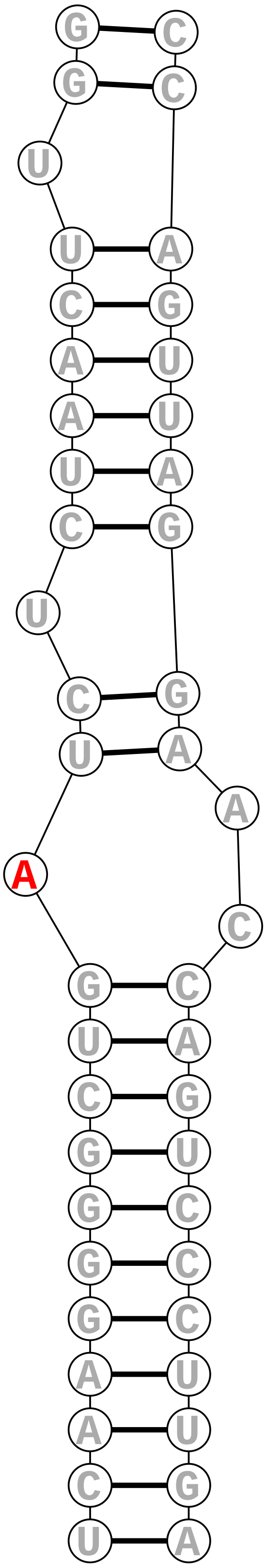

Strands Chr2:121799003-121799026
and Chr2:121799473-121799495

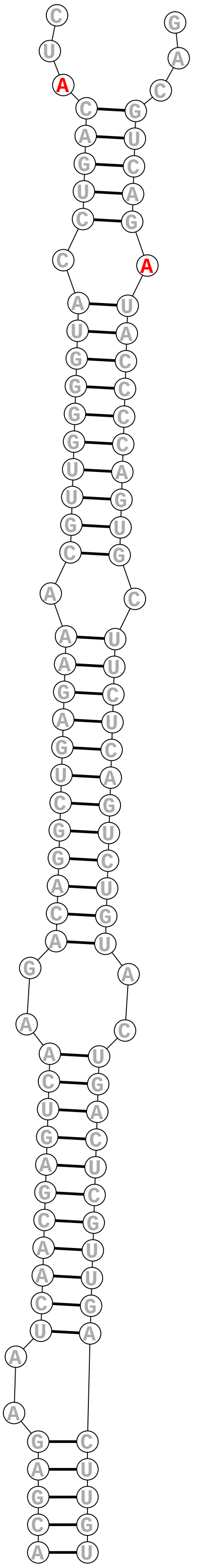

RBL1

Strands Chr13:66624259-66624310
and Chr13:66624736-66624785

RBM14 part A

Strands Chr29:45303485-45303515
and Chr29:45303811-45303841

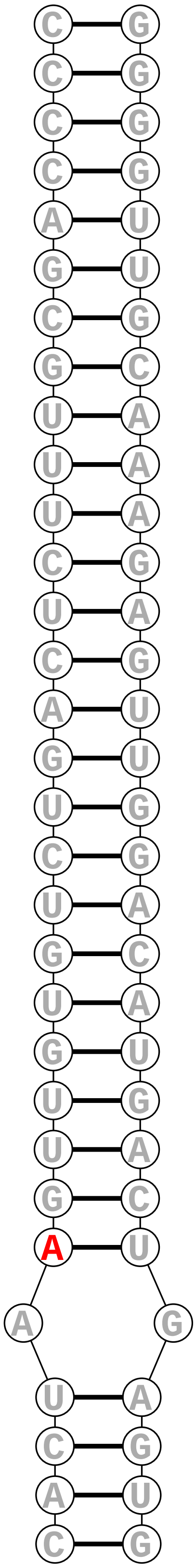

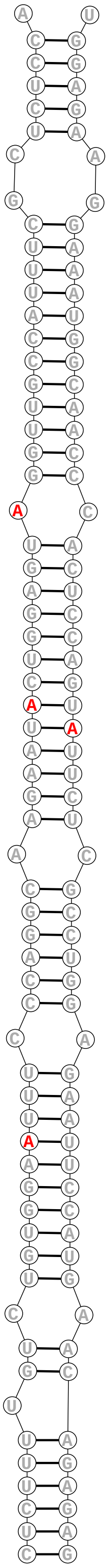

RBM14 part B

Strands Chr29:45303535-45303596
and Chr29:45303727-45303787

RBMS2

Strands Chr5:57169385-57169570
and Chr5:57169798-57169984

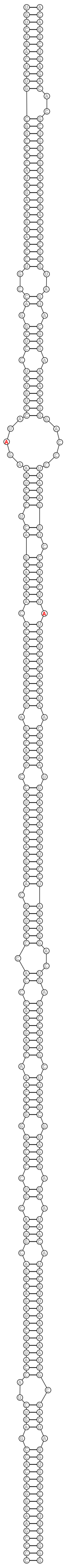

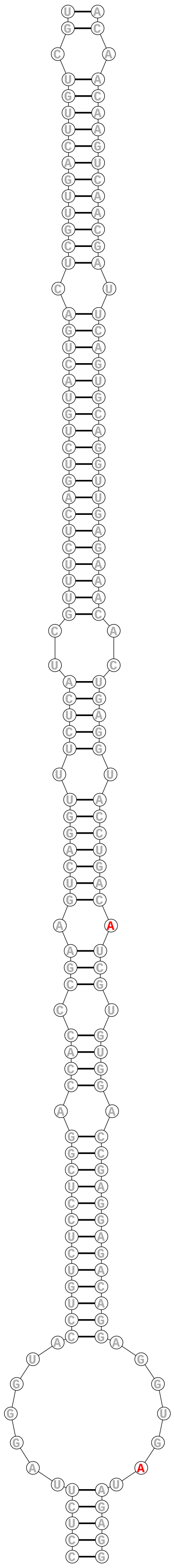

RBP7

Strands Chr16:44427370-44427454
and Chr16:44427944-44428028

RCBTB1 part A

Strands Chr12:19179862-19179949
and Chr12:19180264-19180350

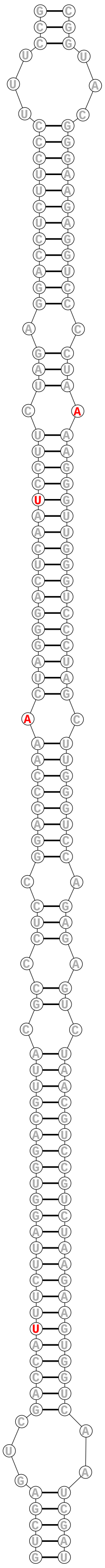

RCBTB1 part B

Strands Chr12:19179862-19179958
and Chr12:19181183-19181265

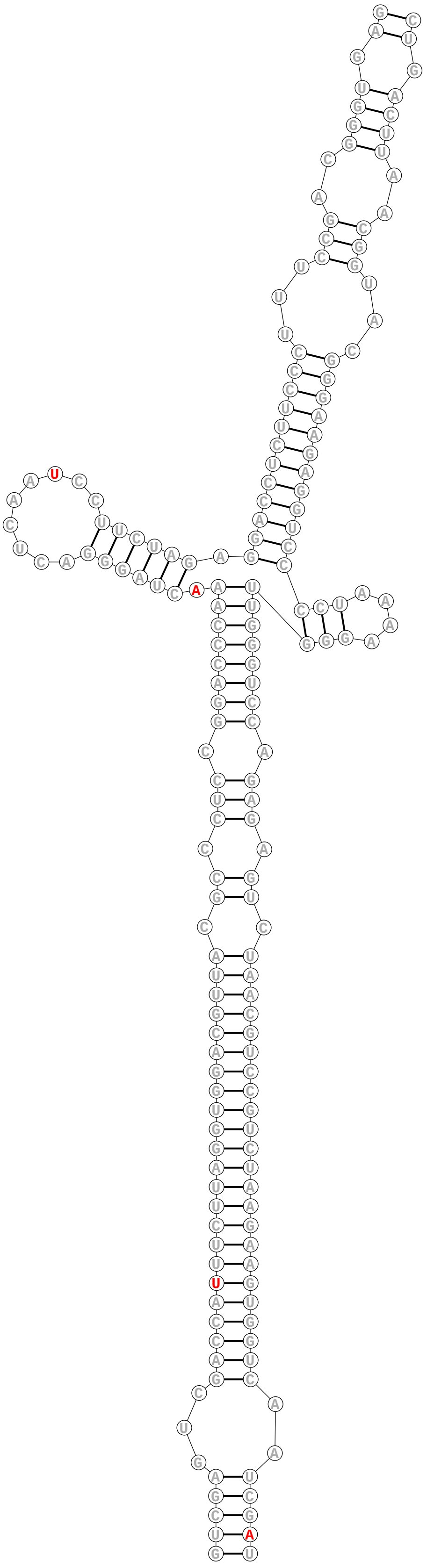

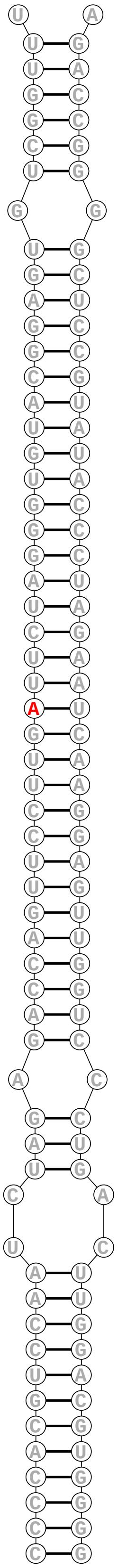

RCHY1

Strands Chr6:92062312-92062369
and Chr6:92063162-92063219

RDH13

Strands Chr18:62794891-62795005
and Chr18:62796017-62796131

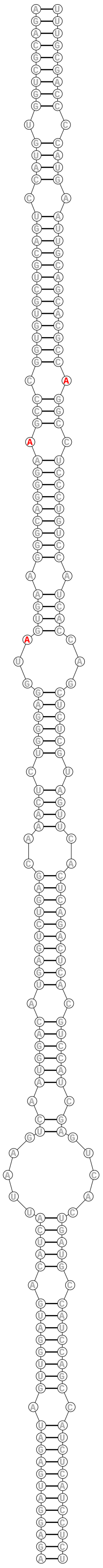

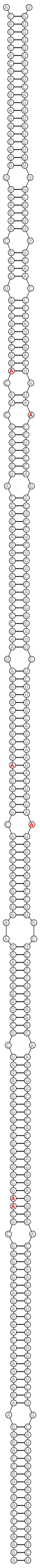

RETSAT part A

Strands Chr11:49483509-49483694
and Chr11:49484805-49484990

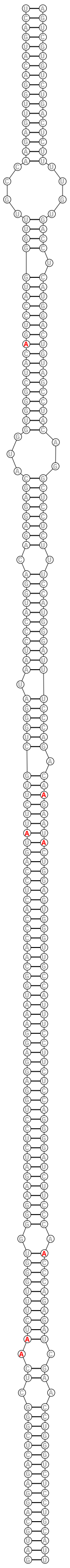

RETSAT part B

Strands Chr11:49483532-49483683
and Chr11:49485188-49485339

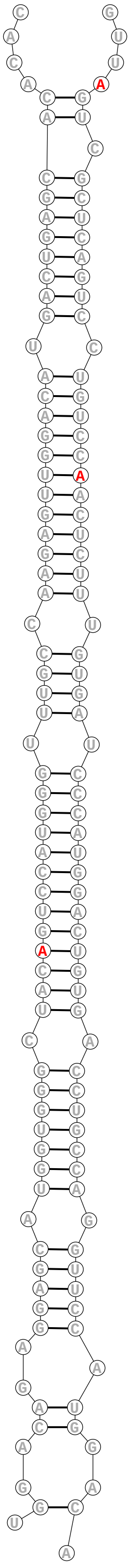

RETSAT part C

Strands Chr11:49483626-49483693
and Chr11:49486273-49486340

RFTN2 part A

Strands Chr2:86506924-86507065
and Chr2:86509234-86509374

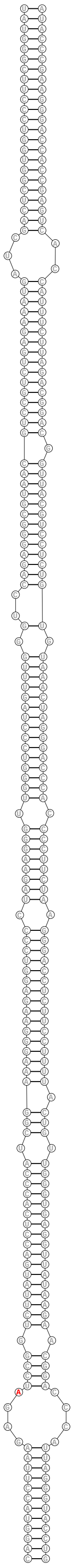

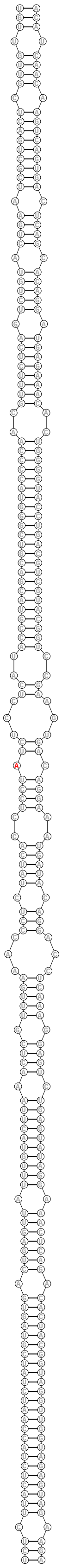

RFTN2 part B

Strands Chr2:86509615-86509763
and Chr2:86511018-86511166

RGCC part A

Strands Chr12:11630912-11631026
and Chr12:11631420-11631535

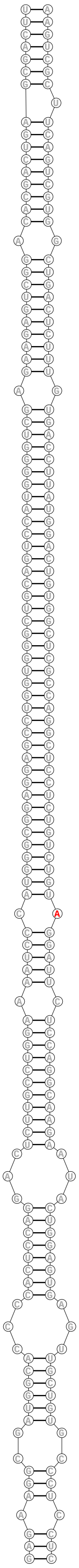

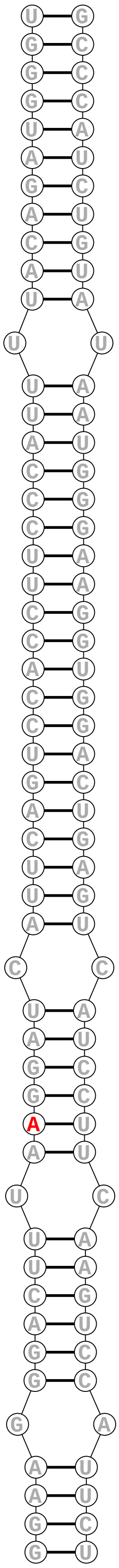

RGCC part B

Strands Chr12:11635077-11635127
and Chr12:11635136-11635186

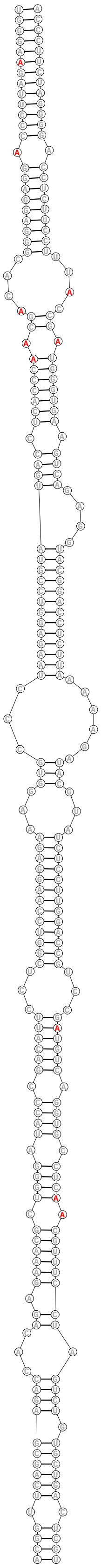

RHBDF1 part A

Strands Chr25:123442-123565
and Chr25:123798-123926

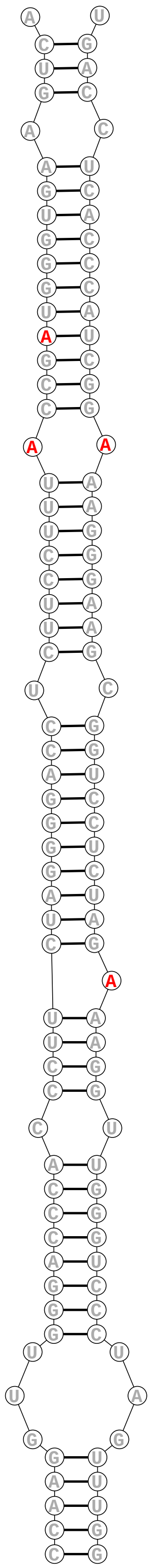

RHBDF1 part B

Strands Chr25:123783-123839
and Chr25:124231-124288

RHBDF1 part C

Strands Chr25:124159-124200
and Chr25:125356-125397

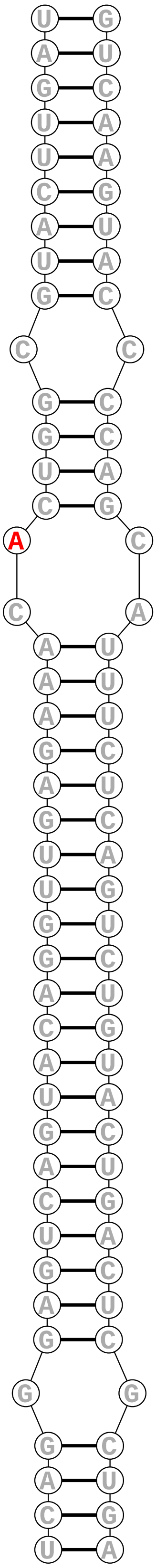

RIOK3

Strands Chr24:33528481-33528601
and Chr24:33528955-33529074

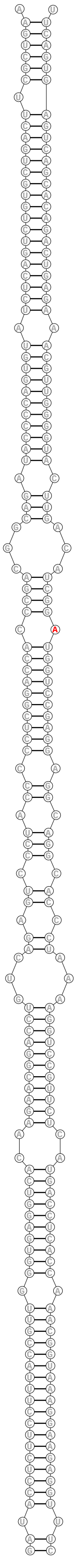

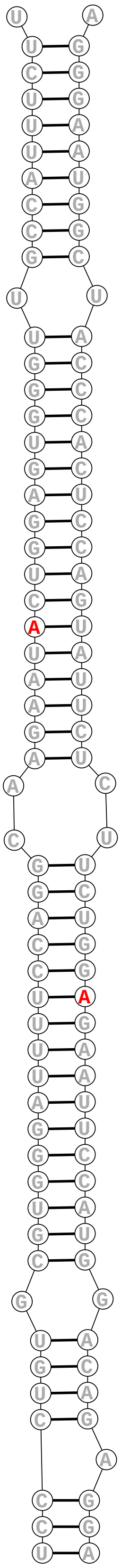

RMND5B

Strands Chr7:40595701-40595754
and Chr7:40595940-40595994

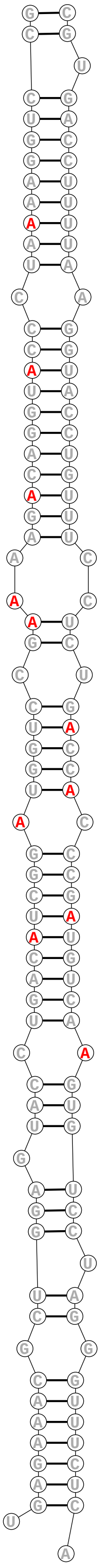

RNASE4

Strands Chr10:26428235-26428296
and Chr10:26428769-26428831

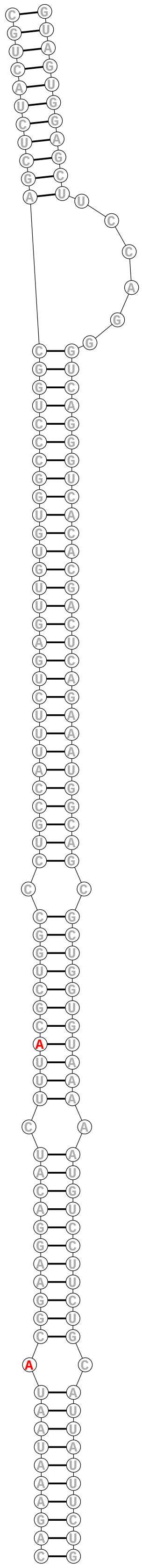

RNF130

Strands Chr7:1202572-1202646
and Chr7:1203804-1203884

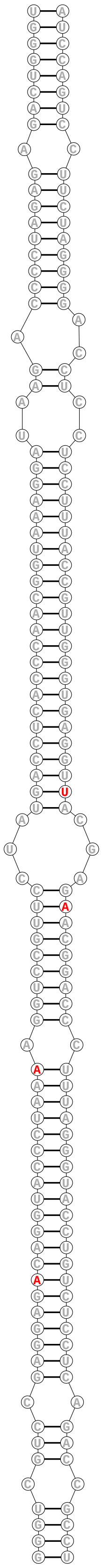

RNF149

Strands Chr11:6205734-6205821
and Chr11:6206196-6206284

RNF31

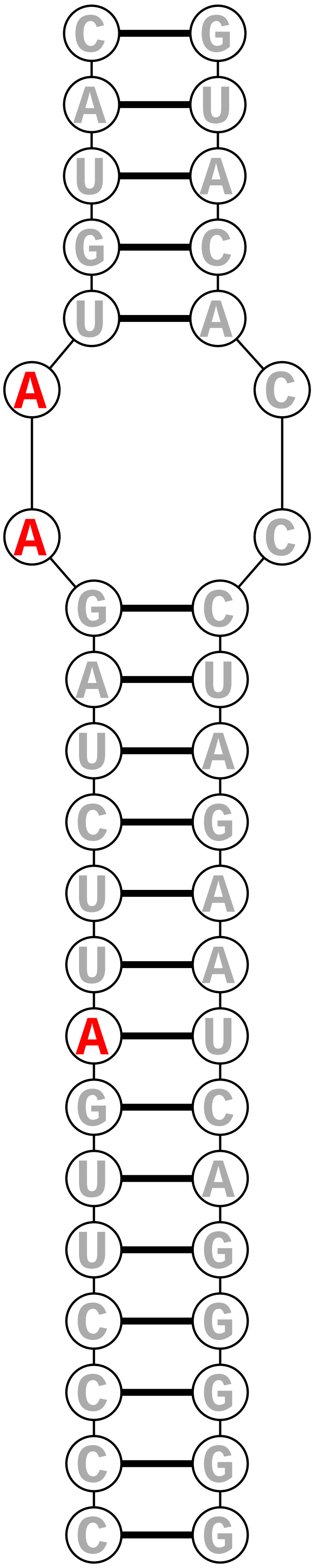

Strands Chr10:20829492-20829512
and Chr10:20829863-20829883

RNF4 part A

Strands Chr6:108313731-108313853
and Chr6:108316780-108316905

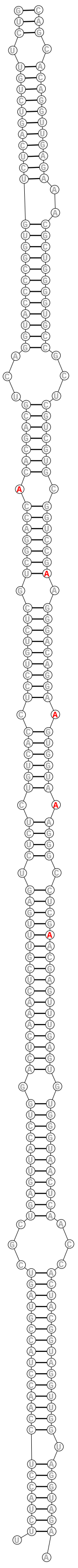

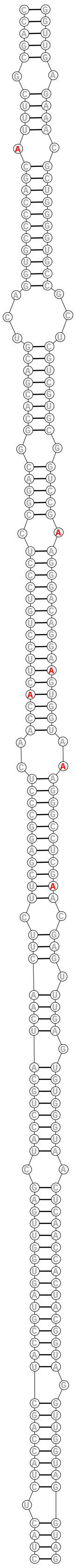

RNF4 part B

Strands Chr6:108315809-108315923
and Chr6:108316787-108316903

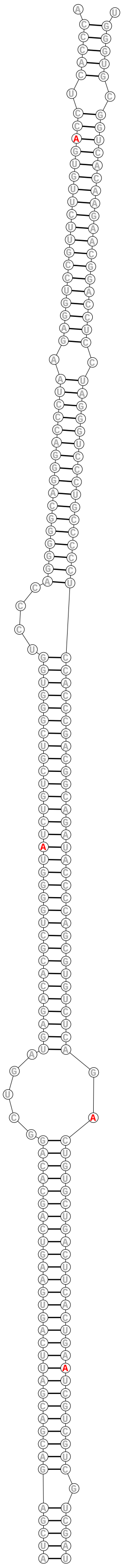

RPAP2

Strands Chr3:51195739-51195855
and Chr3:51197090-51197200

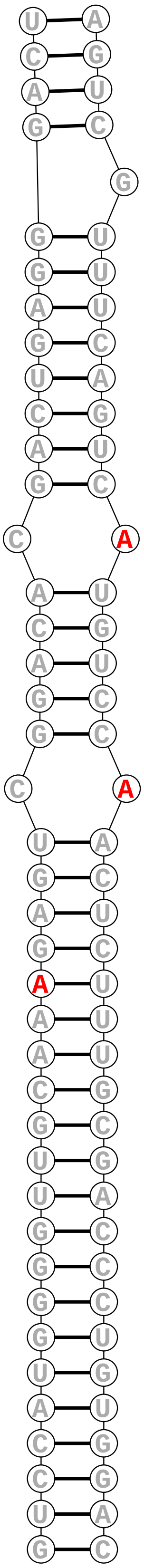

RPS6KA4 part A

Strands Chr29:43278323-43278362
and Chr29:43278406-43278446

RPS6KA4 part B

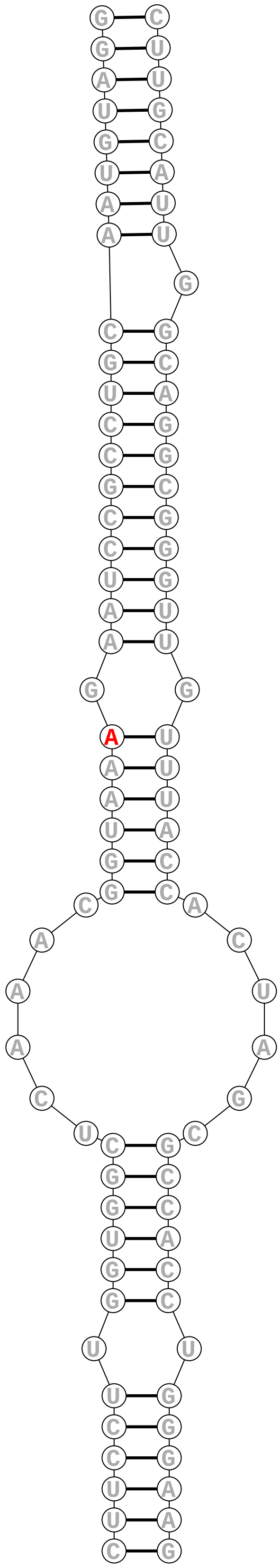

Strands Chr29:43277102-43277146
and Chr29:43278569-43278614

RPS6KA4 part C

Strands Chr29:43277142-43277195
and Chr29:43278511-43278564

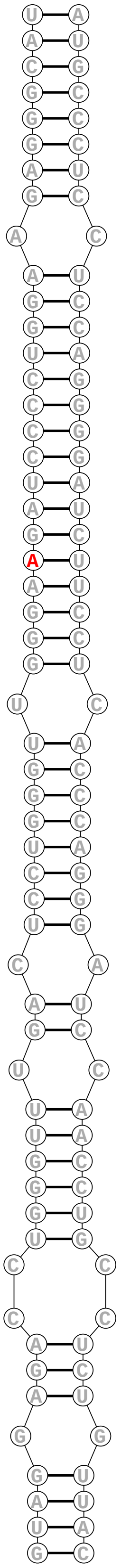

RPS9 part A

Strands Chr18:63384137-63384221
and Chr18:63385641-63385728

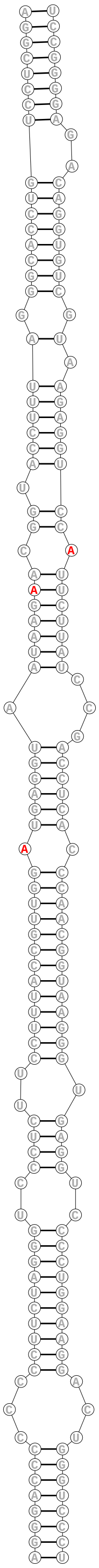

RPS9 part B

Strands Chr18:63385755-63385837
and Chr18:63385870-63385951

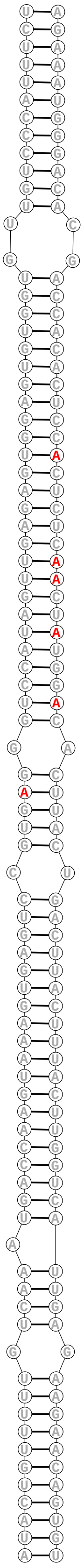

RRN3 part A

Strands Chr25:13794138-13794258
and Chr25:13794674-13794793

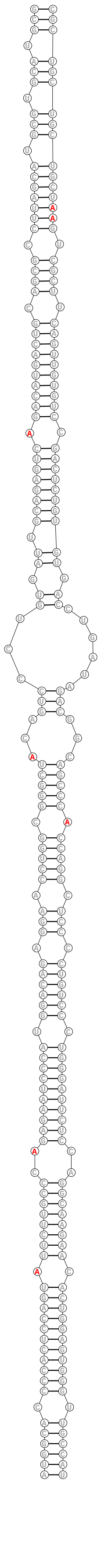

RRN3 part B

Strands Chr25:13794123-13794209
and Chr25:13794865-13794950

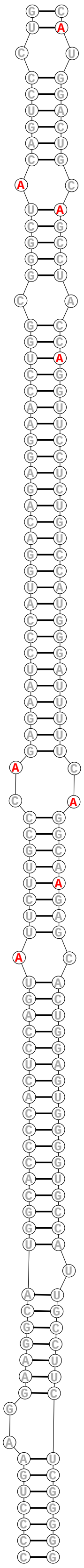

RSG1

Strands Chr2:136459744-136459882
and Chr2:136460074-136460212

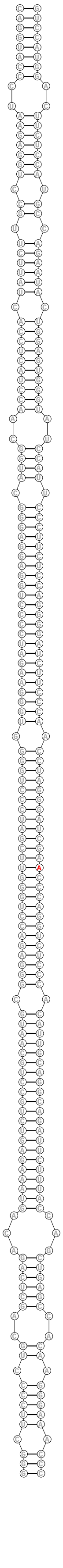

RUFY3 part A

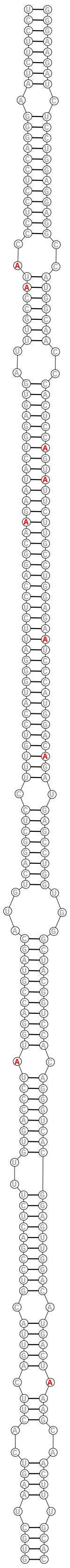

Strands Chr6:87902575-87902709
and Chr6:87903338-87903470

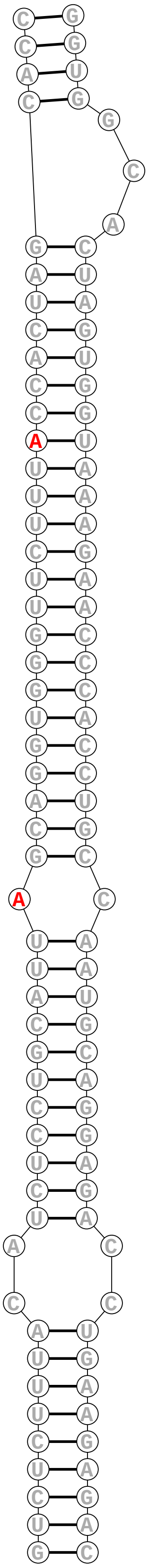

RUFY3 part B

Strands Chr6:87902730-87902779
and Chr6:87903264-87903316

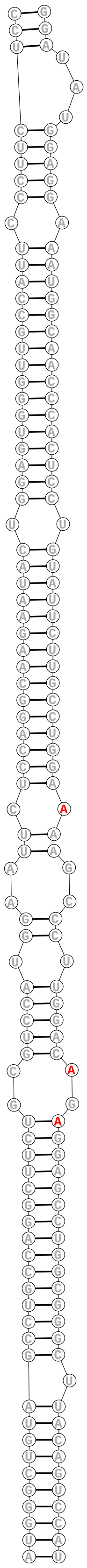

RWDD2B

Strands Chr1:6532180-6532260
and Chr1:6532464-6532548

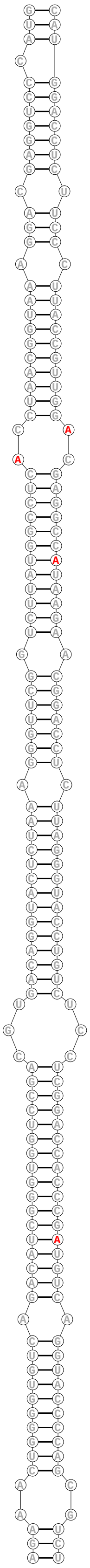

SAMM50

Strands Chr5:114923392-114923490
and Chr5:114926086-114926183

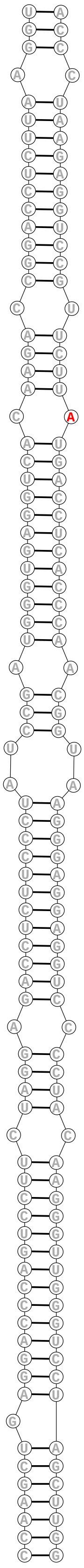

SARAF

Strands Chr27:25393387-25393465
and Chr27:25393582-25393659

SCN1B part A

Strands Chr18:45965593-45965789
and Chr18:45966978-45967142

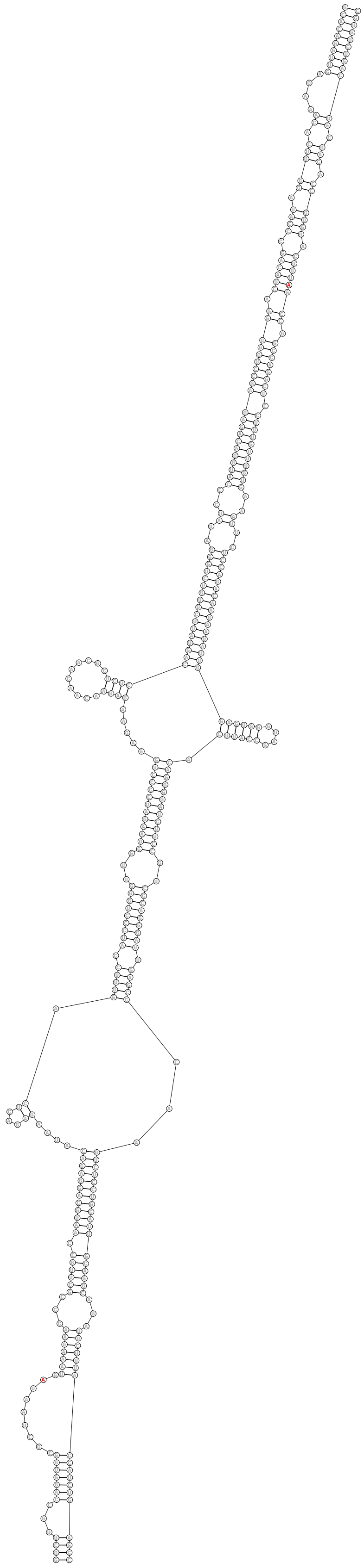

SCN1B part B

Strands Chr18:45965602-45965814
and Chr18:45967301-45967521

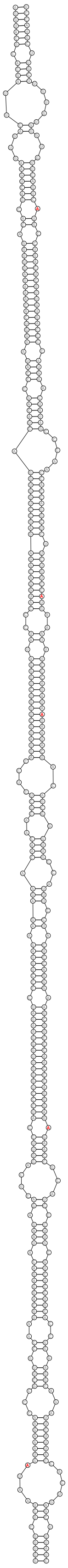

SCN1B part C

Strands Chr18:45967307-45967436
and Chr18:45967864-45967997

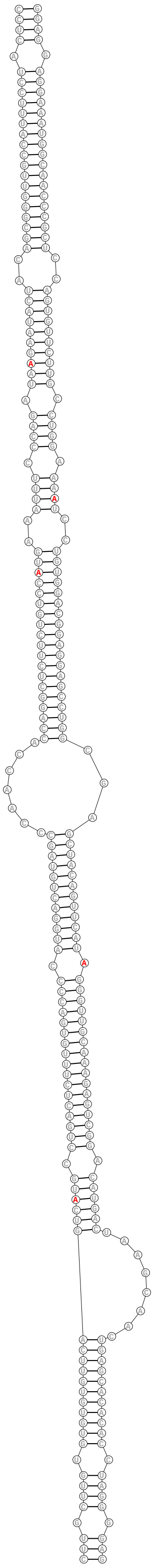

SCNN1A

Strands Chr5:104394330-104394418
and Chr5:104394588-104394677

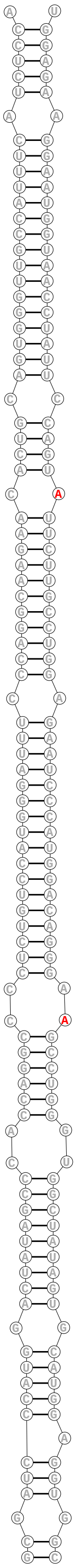

SEC11C

Strands Chr24:58668315-58668411
and Chr24:58668681-58668776

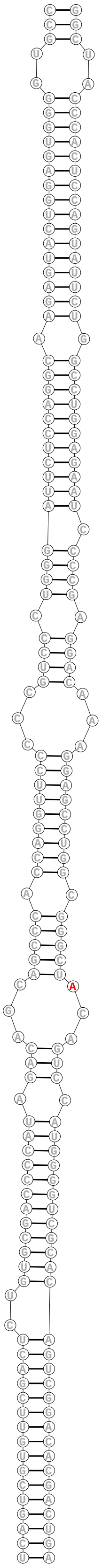

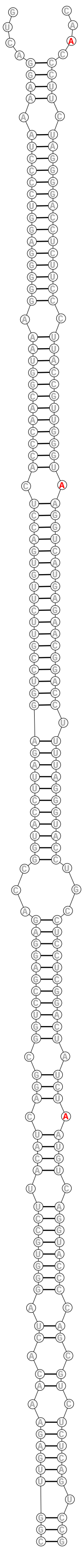

SEC61A1

Strands Chr22:60226036-60226150
and Chr22:60226826-60226942

SEPN1

Strands Chr2:127851282-127851386
and Chr2:127851839-127851935

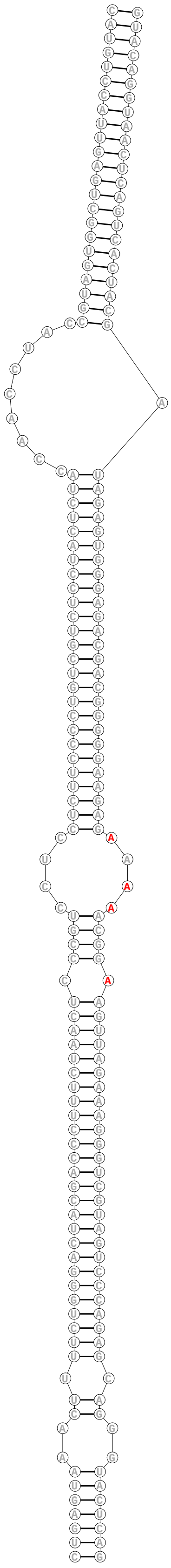

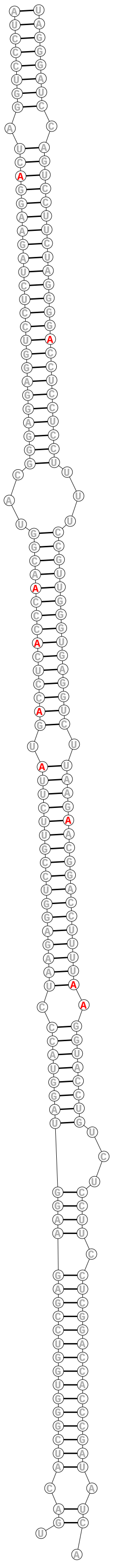

SETMAR part A

Strands Chr22:22109291-22109391
and Chr22:22111350-22111454

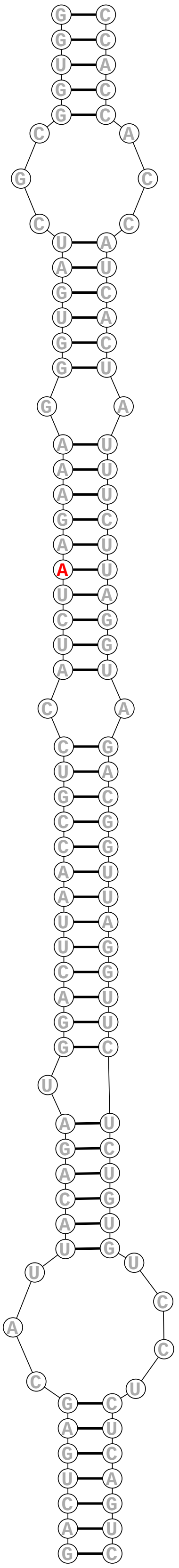

SETMAR part B

Strands Chr22:22109400-22109455
and Chr22:22111289-22111344

SH2D4A

Strands Chr27:38030154-38030350
and Chr27:38030889-38031073

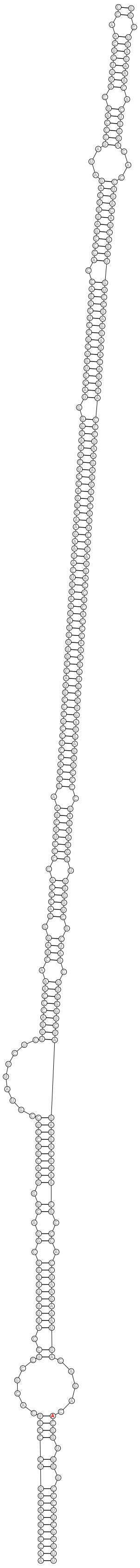

SH3D21

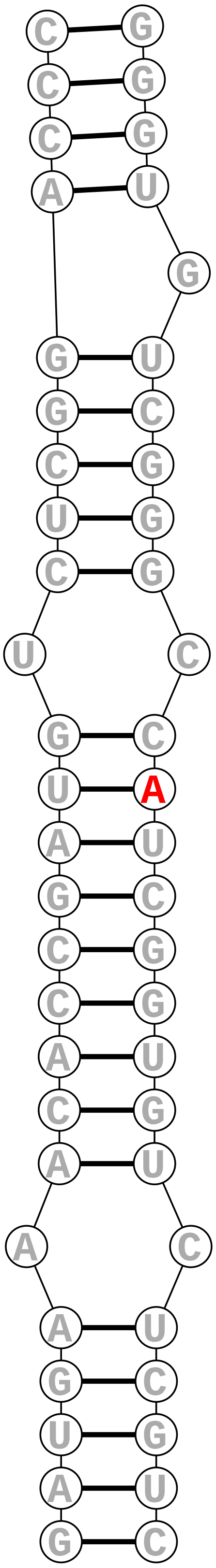

Strands Chr3:110124979-110125003
and Chr3:110125054-110125079

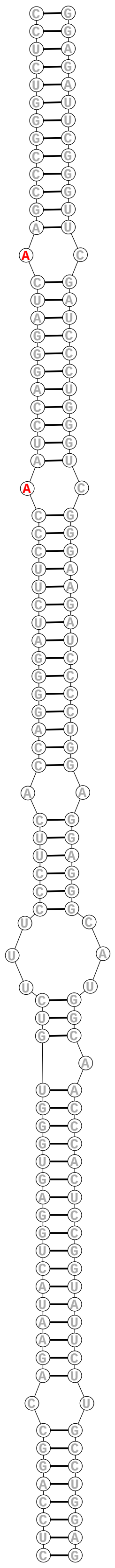

SH3TC1

Strands Chr6:119353945-119354024
and Chr6:119354158-119354238

SIAE

Strands Chr29:28490710-28490832
and Chr29:28491743-28491865

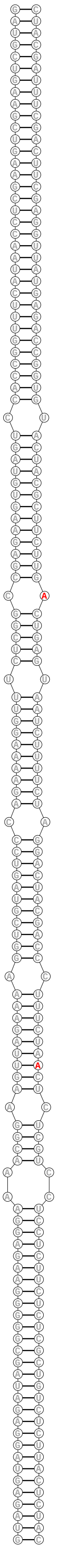

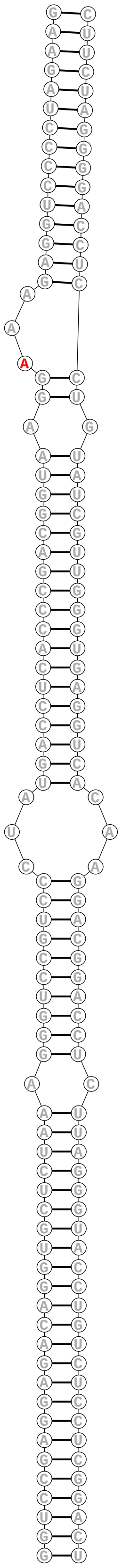

SIRT5

Strands Chr23:42807761-42807837
and Chr23:42808481-42808554

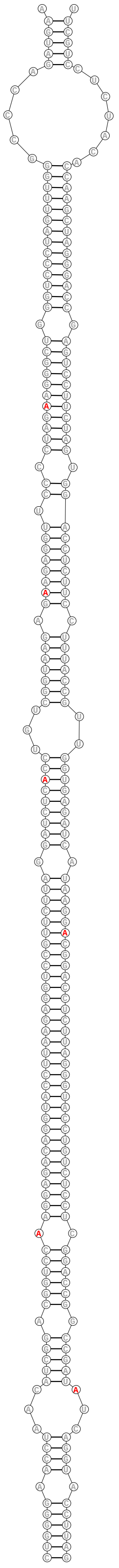

SLC18B1

Strands Chr9:71962040-71962168
and Chr9:71962320-71962448

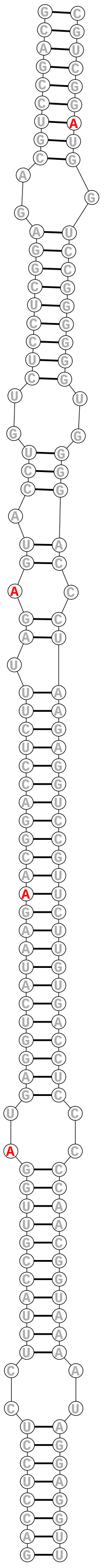

SLC1A1 part A

Strands Chr8:40193586-40193663
and Chr8:40195005-40195079

SLC1A1 part B

Strands Chr8:40194711-40194786
and Chr8:40195006-40195080

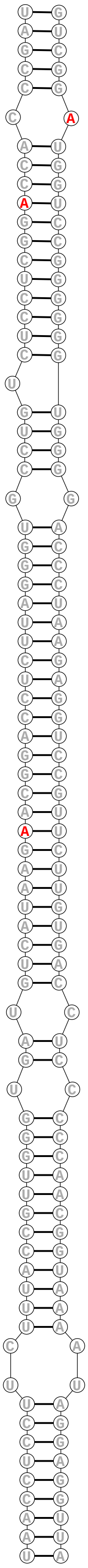

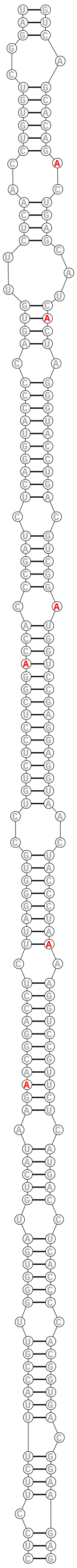

SLC1A1 part C

Strands Chr8:40194715-40194821
and Chr8:40195099-40195205

SLC20A1

Strands Chr11:46227687-46227795
and Chr11:46227927-46228038

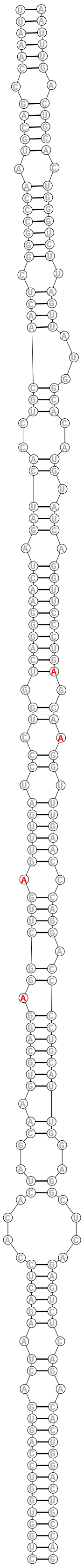

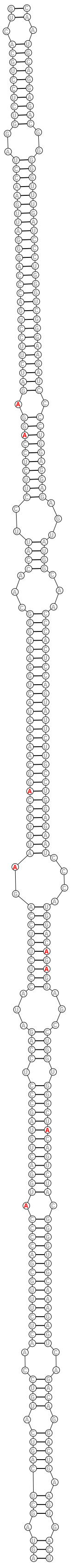

SLC22A16 part A

Strands Chr9:40247293-40247450
and Chr9:40248583-40248744

SLC22A16 part B

Strands Chr9:40247293-40247450
and Chr9:40248583-40248744

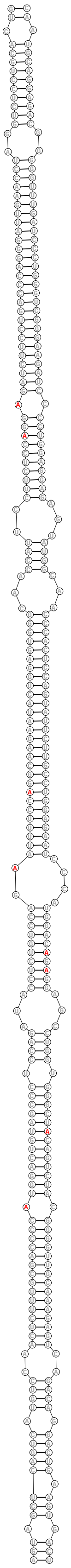

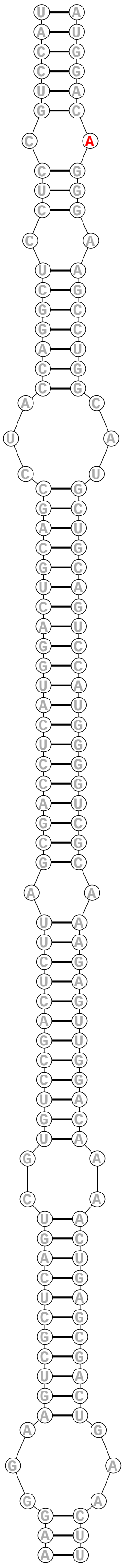

SLC22A16 part C

Strands Chr9:40249346-40249418
and Chr9:40249740-40249812

SLC22A16 part D

Strands Chr9:40254591-40254645
and Chr9:40255115-40255169

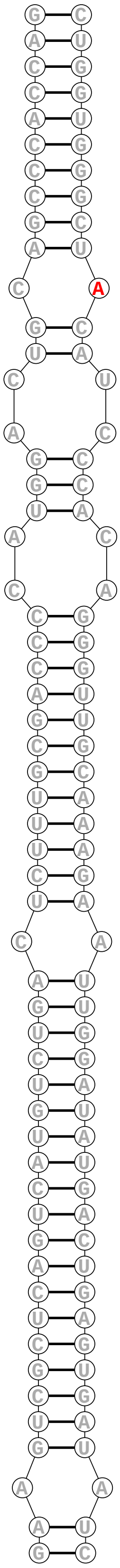

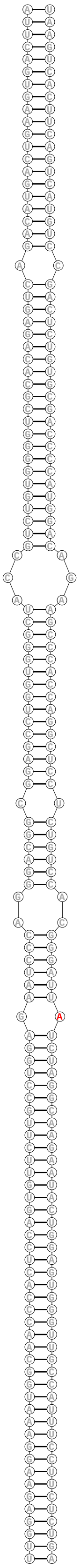

SLC25A38

Strands Chr22:12701832-12701951
and Chr22:12702306-12702425

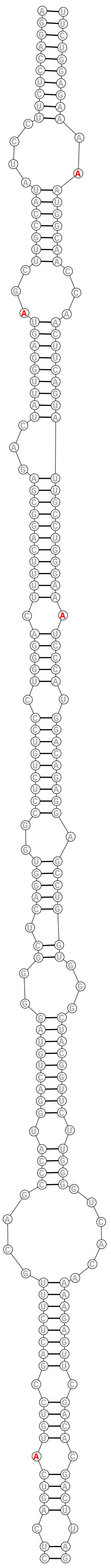

SLC25A46

Strands Chr7:112335137-112335250
and Chr7:112335274-112335383

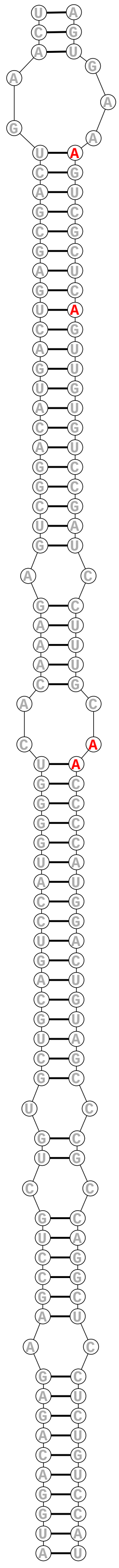

SLC30A2

Strands Chr2:127651461-127651532
and Chr2:127652398-127652470

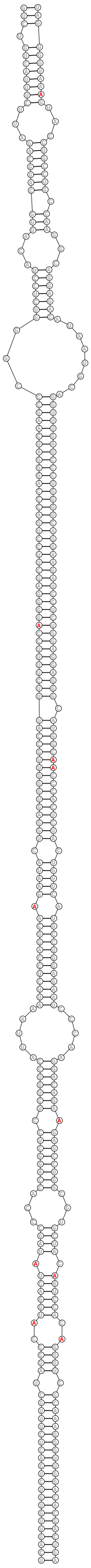

SLC34A2

Strands Chr6:46726713-46726888
and Chr6:46728151-46728332

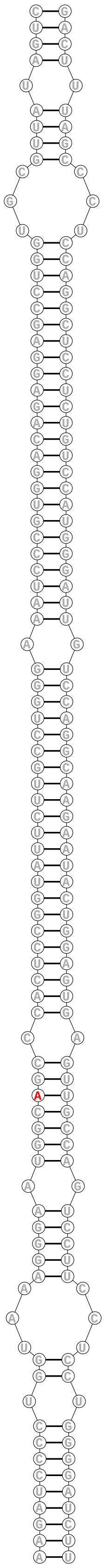

SLC37A1

Strands Chr1:144432801-144432888
and Chr1:144433328-144433415

SLC39A11 part A

Strands Chr19:59070561-59070711
and Chr19:59070868-59071015

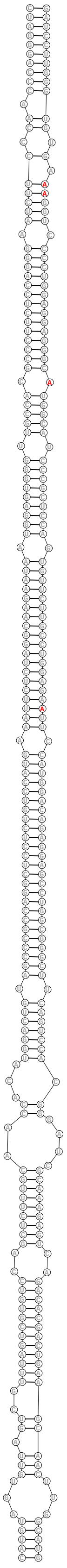

SLC39A11 part B

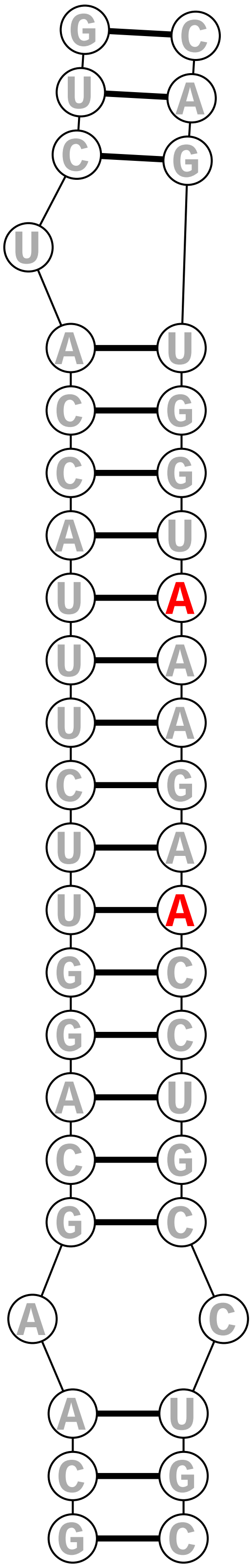

Strands Chr19:59070732-59070754
and Chr19:59070817-59070838

SLC39A14

Strands Chr8:70233833-70233880
and Chr8:70234135-70234182

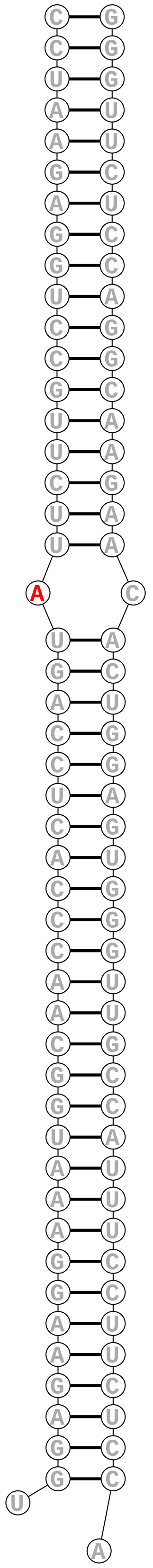

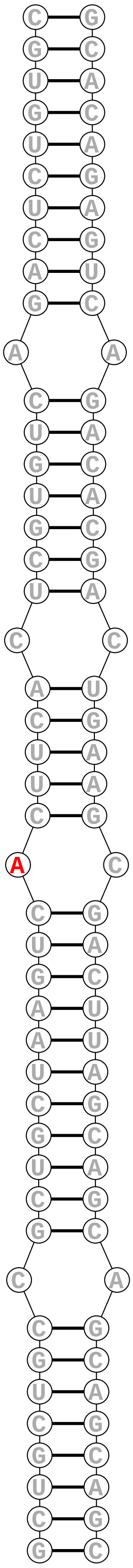

SLC39A7

Strands Chr23:7329732-7329776
and Chr23:7330280-7330324

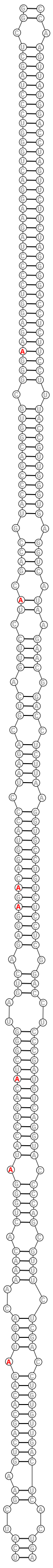

SLC44A3 part A

Strands Chr3:48786233-48786379
and Chr3:48789601-48789745

SLC44A3 part B

Strands Chr3:48786260-48786319
and Chr3:48789754-48789828

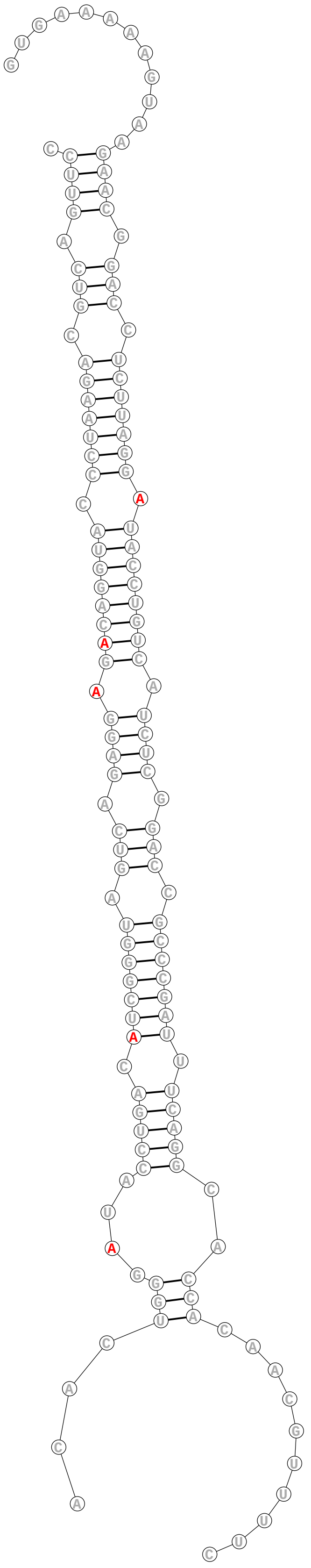

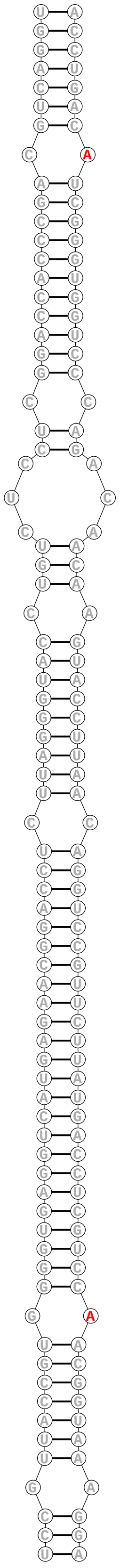

SLC5A9

Strands Chr3:98572303-98572377
and Chr3:98572803-98572877

SLC6A16

Strands Chr18:56245193-56245289
and Chr18:56246212-56246308

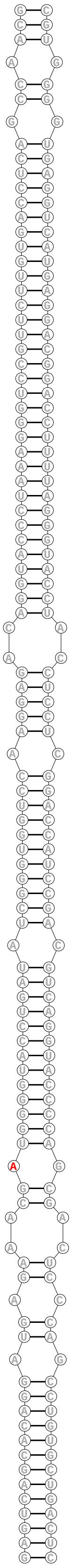

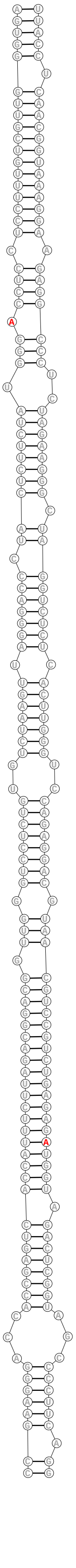

SLC8B1

Strands Chr17:63568701-63568807
and Chr17:63569694-63569802

SMAD1

Strands Chr17:12908100-12908214
and Chr17:12908577-12908688

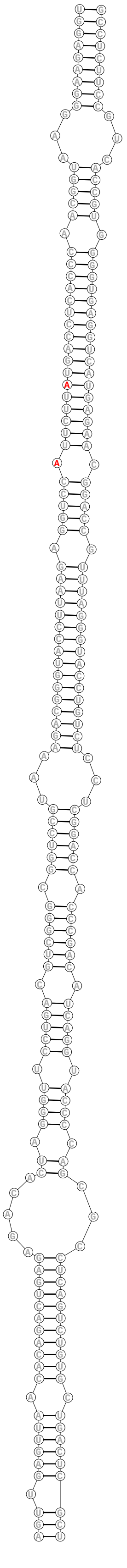

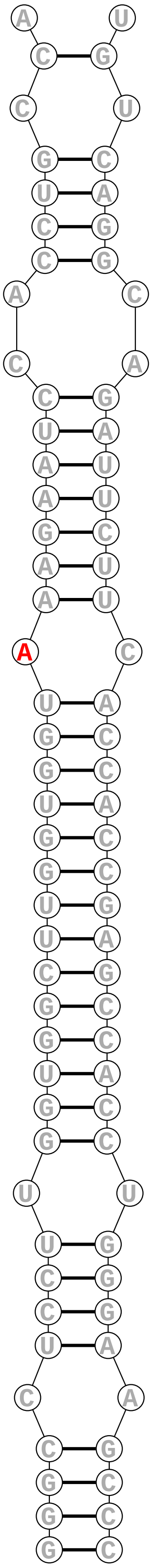

SMAP1 part A

Strands Chr9:10192737-10192777
and Chr9:10193372-10193412

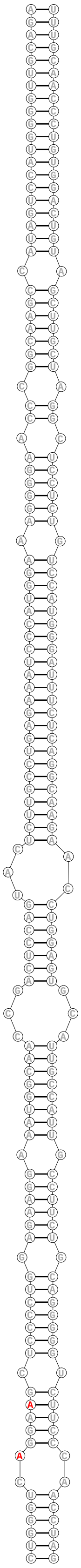

SMAP1 part B

Strands Chr9:10192802-10192913
and Chr9:10193238-10193348

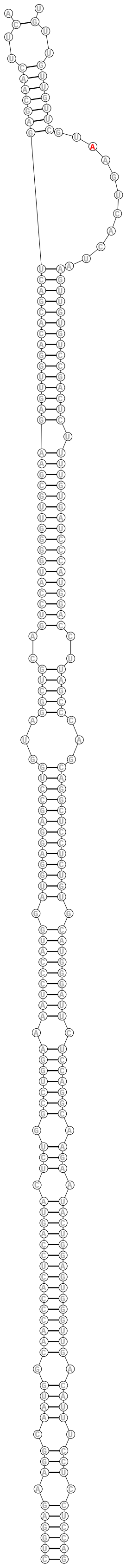

SNRNP40 part A

Strands Chr2:122869841-122869960
and Chr2:122870439-122870570

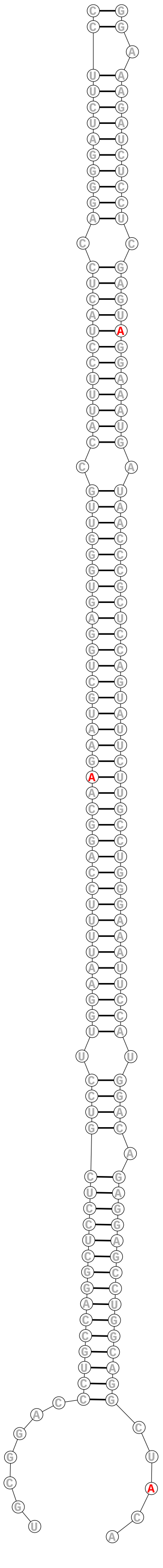

SNRNP40 part B

Strands Chr2:122899056-122899143
and Chr2:122900307-122900394

SNRNP40 part C

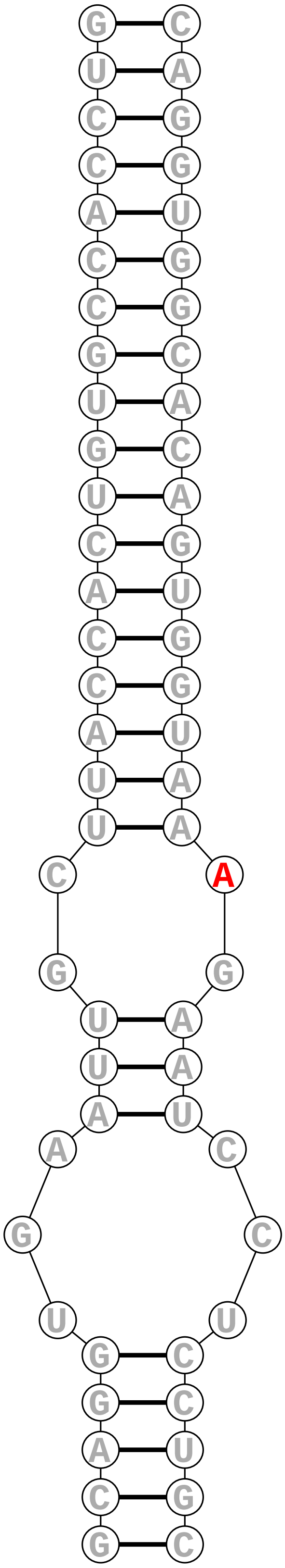

Strands Chr2:122899181-122899211
and Chr2:122900234-122900264

SNRNP40 part D

Strands Chr2:122899059-122899143
and Chr2:122900307-122900398

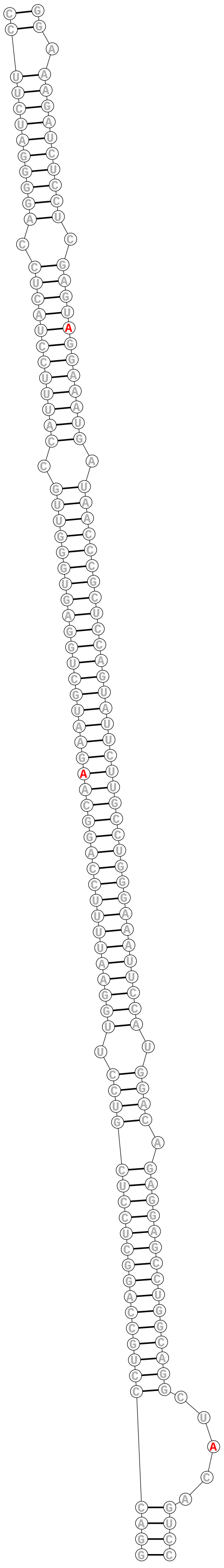

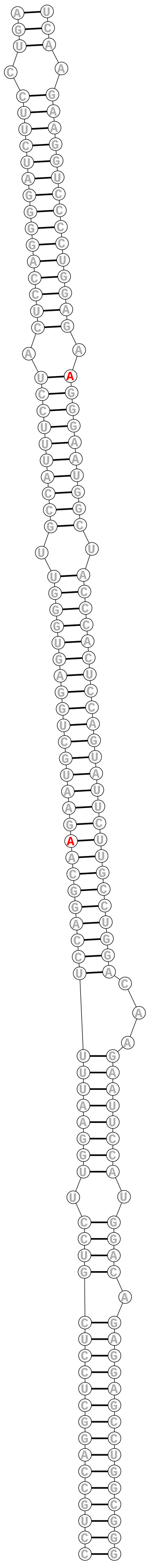

SNRNP40 part E

Strands Chr2:122899063-122899146
and Chr2:122900917-122901004

SNRNP40 part F

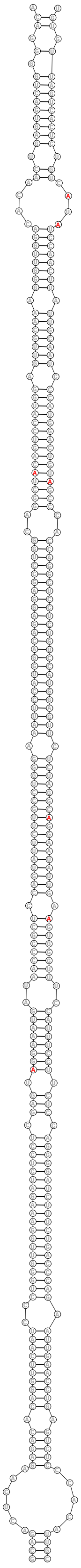

Strands Chr2:122900272-122900441
and Chr2:122901522-122901688

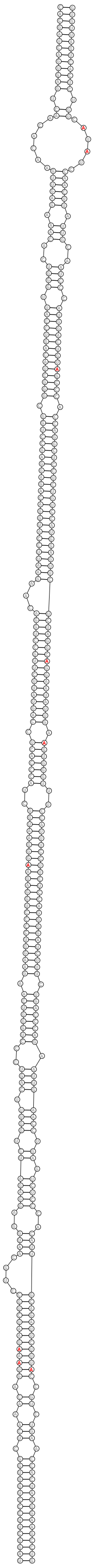

SNRNP40 part G

Strands Chr2:122900846-122901054
and Chr2:122901523-122901723

SNRNP40 part H

Strands Chr2:122900224-122900270
and Chr2:122901785-122901830

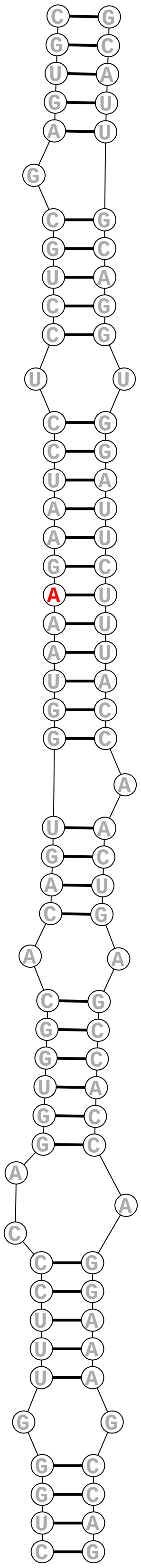

SNX14

Strands Chr9:64810830-64810971
and Chr9:64811260-64811400

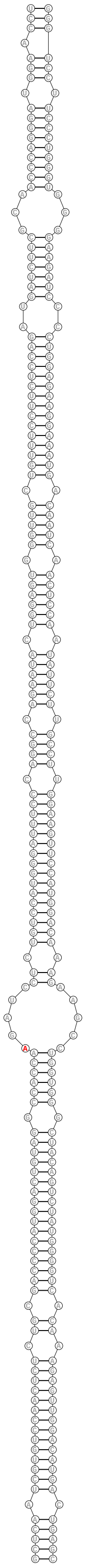

SNX3

Strands Chr9:42414079-42414135
and Chr9:42414466-42414521

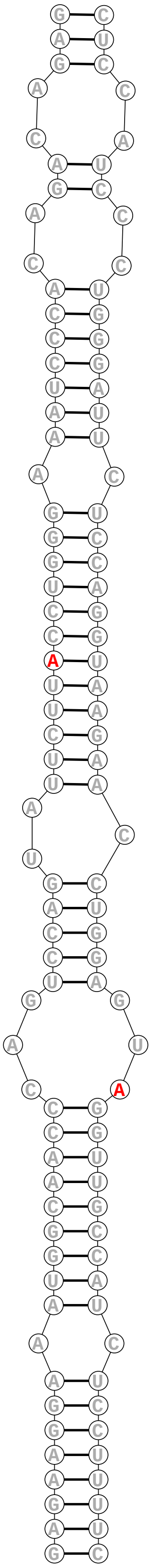

SNX33

Strands Chr21:33626977-33627024
and Chr21:33627604-33627651

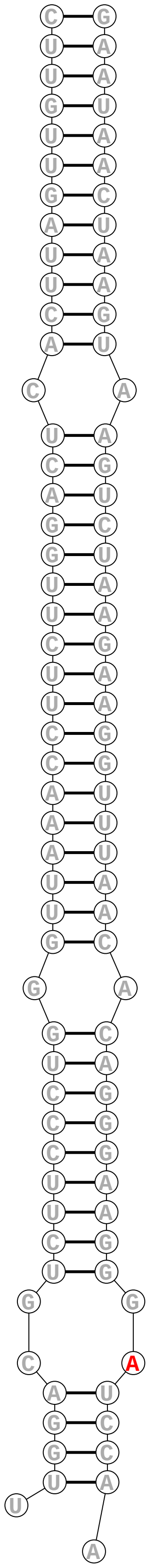

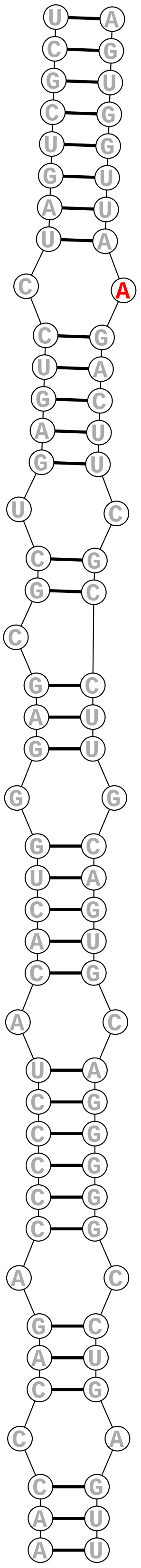

SPINT2

Strands Chr18:48319662-48319703
and Chr18:48321089-48321129

SPRTN

Strands Chr28:3353082-3353166
and Chr28:3354508-3354592

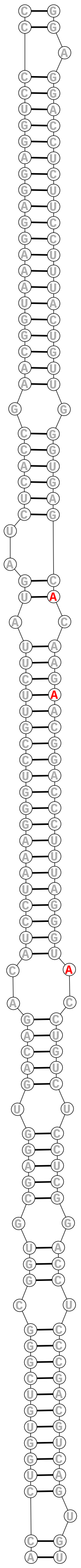

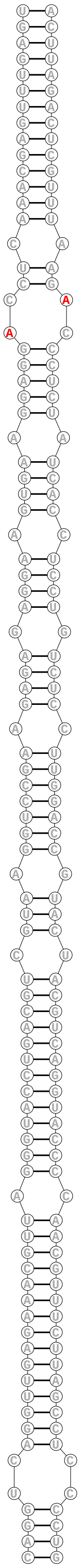

SPTBN2

Strands Chr29:45415960-45416046
and Chr29:45417997-45418083

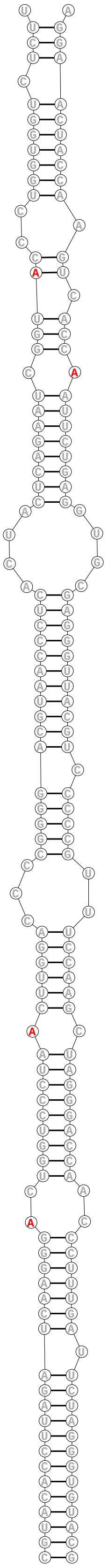

SPTSSA

Strands Chr21:45304189-45304277
and Chr21:45305304-45305392

SRP14

Strands Chr10:35798511-35798644
and Chr10:35802174-35802303

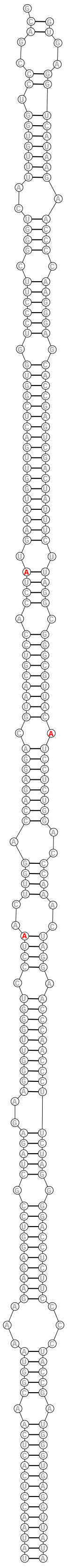

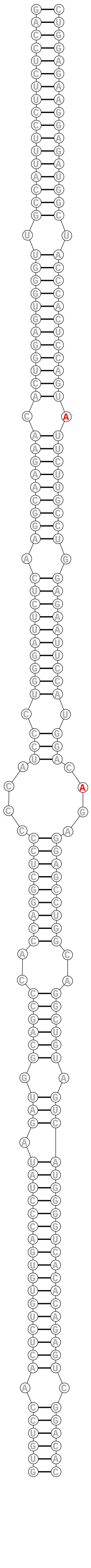

SRP54

Strands Chr21:45754959-45755063
and Chr21:45756458-45756561

ST3GAL3 part A

Strands Chr3:102719984-102720036
and Chr3:102720406-102720458

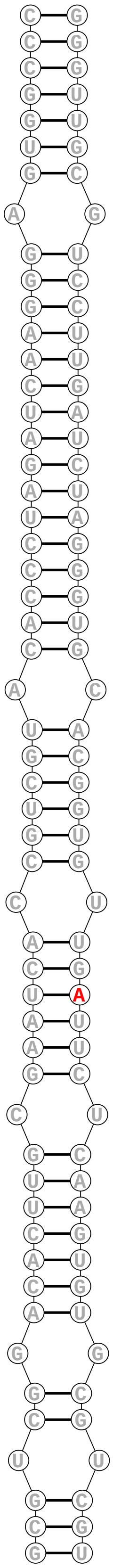

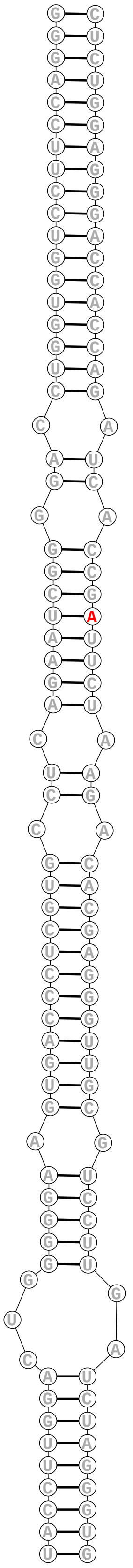

ST3GAL3 part B

Strands Chr3:102720038-102720101
and Chr3:102720367-102720429

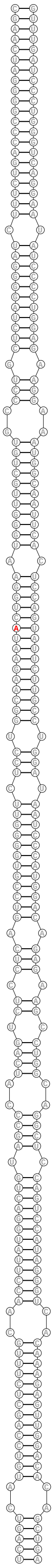

STARD4

Strands Chr10:87276143-87276280
and Chr10:87276398-87276535

STK19 part A

Strands Chr23:27198820-27198974
and Chr23:27199155-27199330

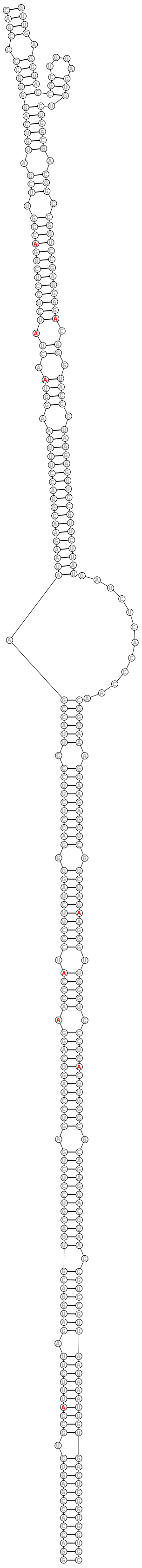

STK19 part B

Strands Chr23:27203700-27203884
and Chr23:27204236-27204409

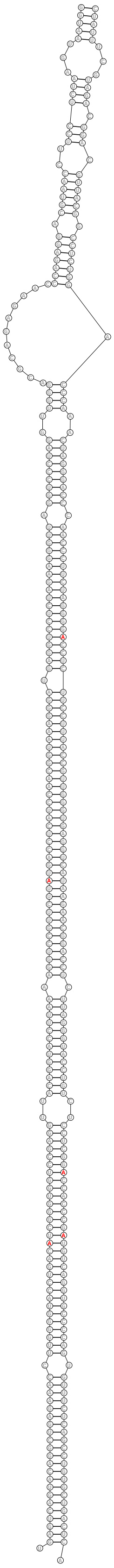

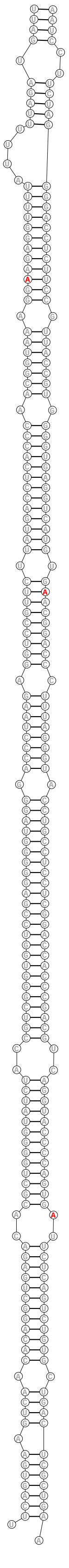

STK19 part C

Strands Chr23:27202228-27202362
and Chr23:27203049-27203179

STT3B part A

Strands Chr22:6183307-6183395
and Chr22:6183653-6183741

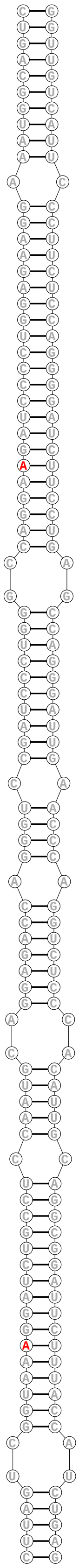

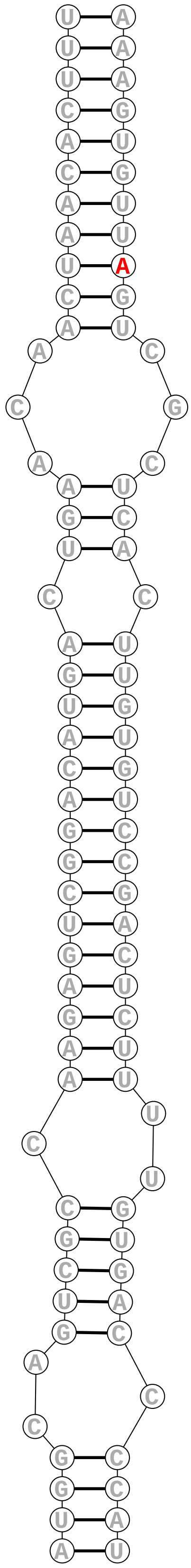

STT3B part B

Strands Chr22:6183387-6183431
and Chr22:6183549-6183593

STX12

Strands Chr2:126141163-126141308
and Chr2:126142262-126142400

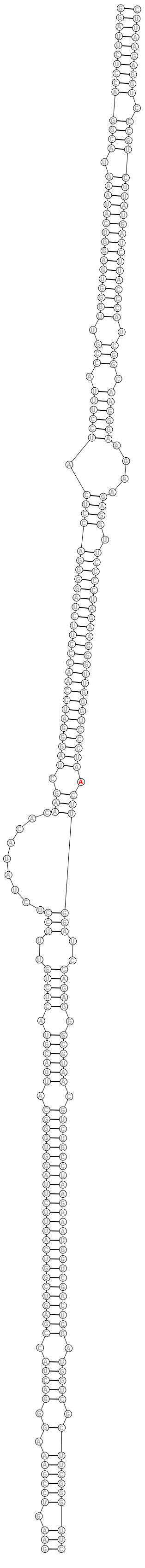

SUCO

Strands Chr16:40969985-40970055
and Chr16:40970871-40970941

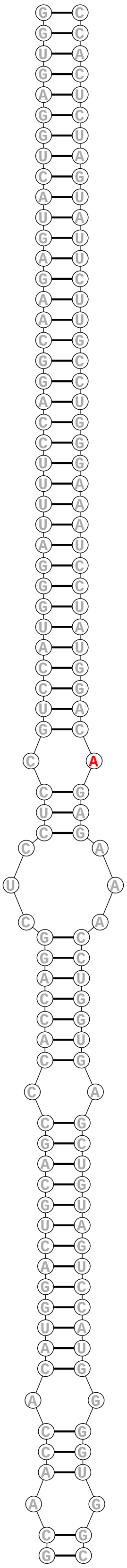

SUGT1 part A

Strands Chr12:11112114-11112262
and Chr12:11113105-11113250

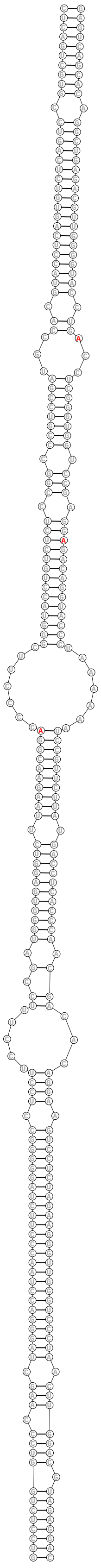

SUGT1 part B

Strands Chr12:11113143-11113304
and Chr12:11114572-11114728

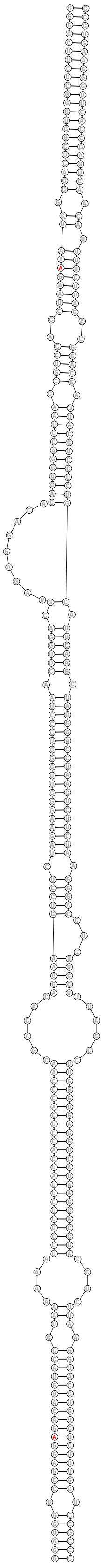

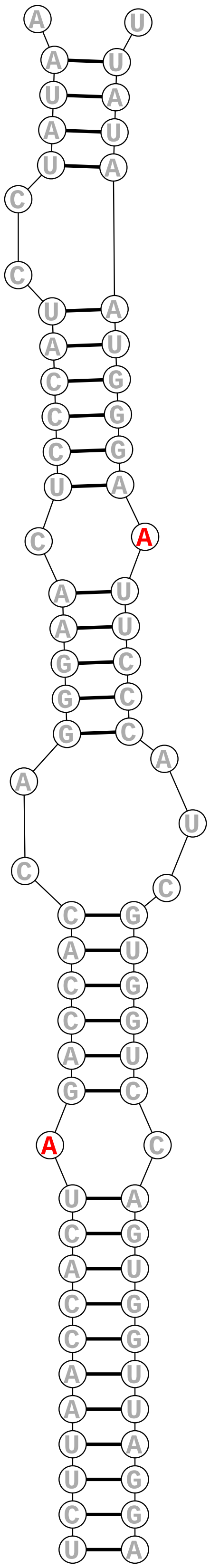

SYNC

Strands Chr2:121799053-121799091
and Chr2:121799417-121799454

TADA1

Strands Chr3:2095739-2095782
and Chr3:2096174-2096217

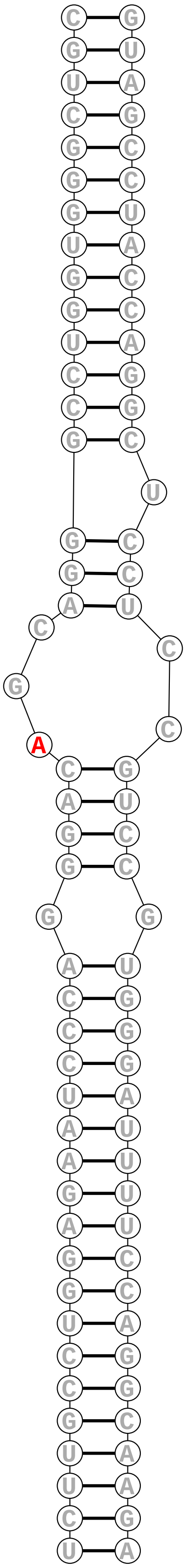

TAGLN2

Strands Chr3:9883374-9883495
and Chr3:9883736-9883857

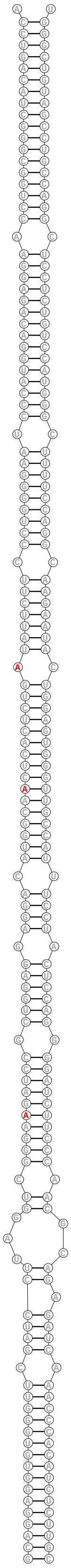

TARBP1

Strands Chr28:7246935-7247025
and Chr28:7247341-7247431

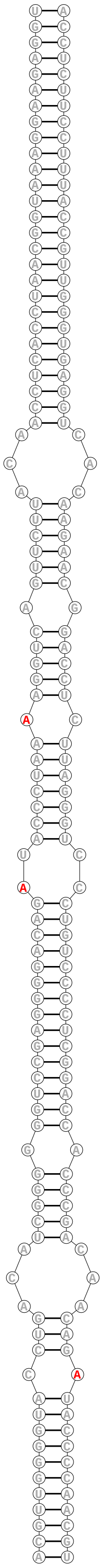

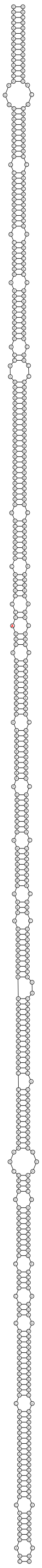

TAX1BP1

Strands Chr4:68802678-68802939
and Chr4:68804062-68804326

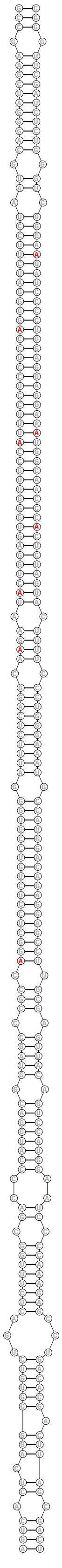

TCN2

Strands Chr17:71723146-71723293
and Chr17:71724177-71724324

TEKT3

Strands Chr19:33427705-33427886
and Chr19:33428139-33428318

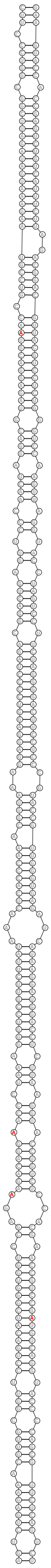

TGFB2

Strands Chr16:22586580-22586634
and Chr16:22586757-22586811

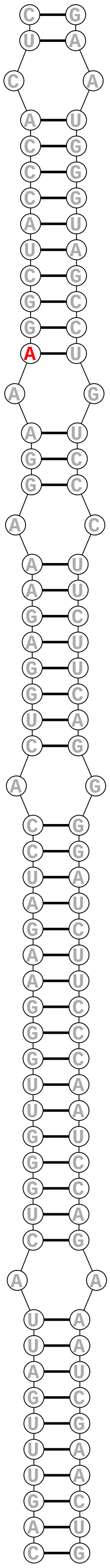

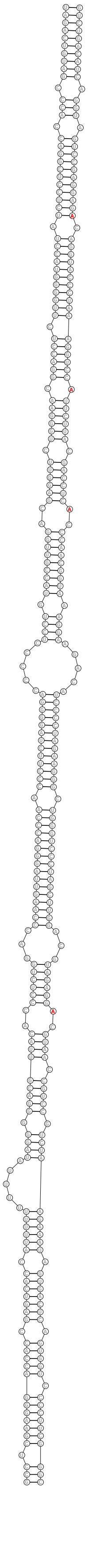

TINAGL1

Strands Chr2:122691439-122691609
and Chr2:122691655-122691820

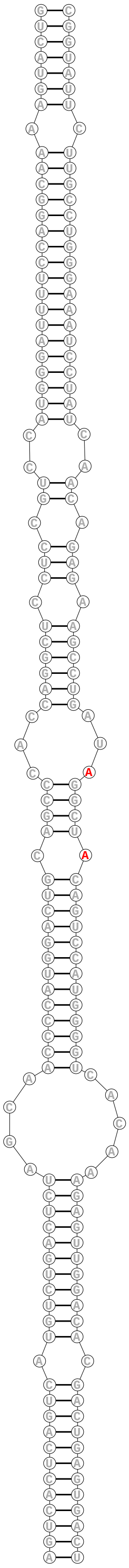

TJP2 part A

Strands Chr8:45683293-45683382
and Chr8:45684074-45684164

TJP2 part B

Strands Chr8:45683145-45683296
and Chr8:45685674-45685829

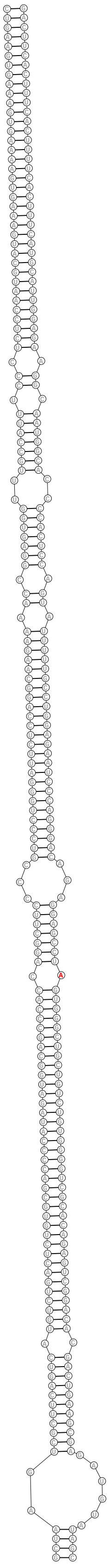

TJP3

Strands Chr7:21453419-21453559
and Chr7:21453864-21454003

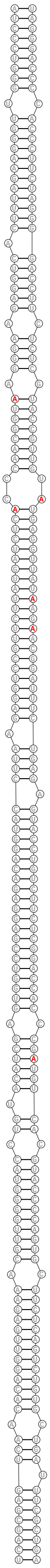

TKT

Strands Chr22:48287139-48287182
and Chr22:48287343-48287386

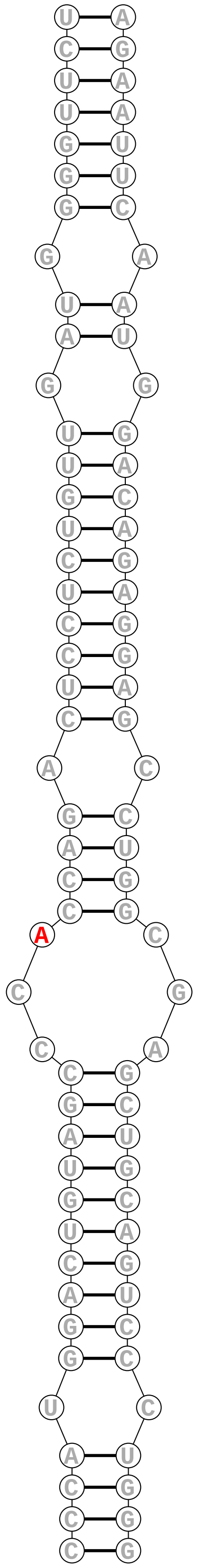

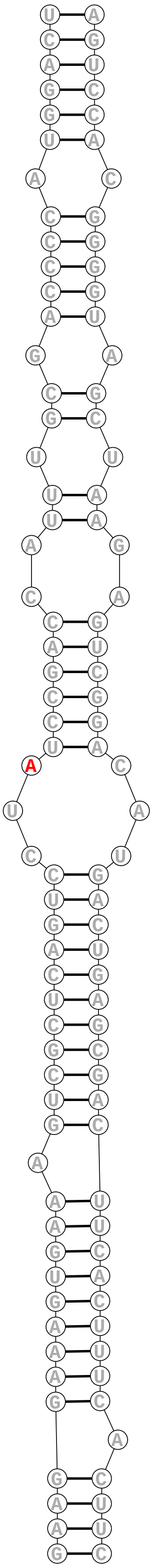

TLR3

Strands Chr27:15242289-15242342
and Chr27:15242607-15242660

TLR6 part A

Strands Chr6:59699767-59699819
and Chr6:59701236-59701289

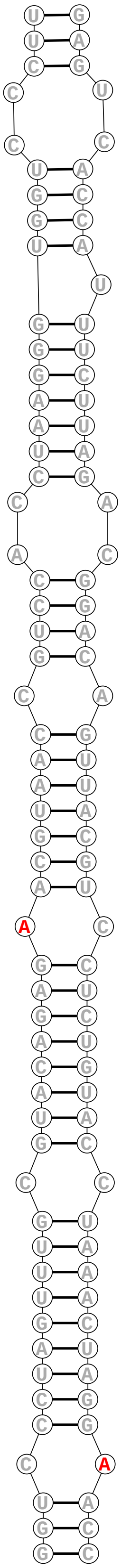

TLR6 part B

Strands Chr6:59702553-59702692
and Chr6:59703543-59703681

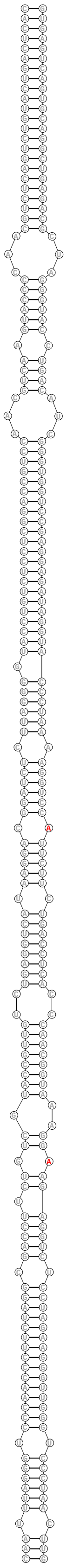

TM2D2

Strands Chr27:33801503-33801605
and Chr27:33802119-33802221

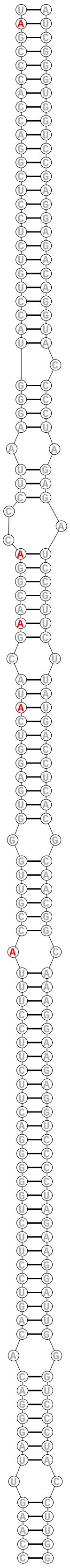

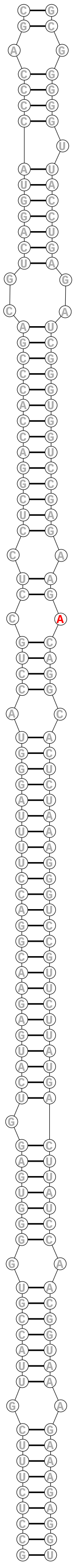

TMED2

Strands Chr17:54331585-54331673
and Chr17:54332089-54332177

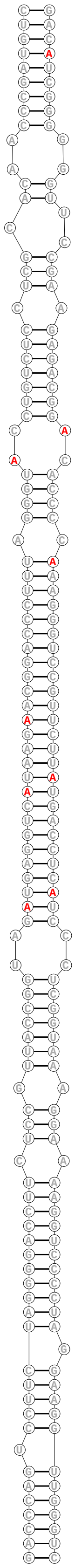

TMEM120B part A

Strands Chr17:55790716-55790811
and Chr17:55791860-55791956

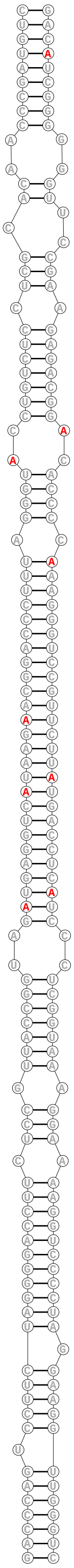

TMEM120B part B

Strands Chr17:55790716-55790811
and Chr17:55791860-55791956

TMEM120B part C

Strands Chr17:55791825-55791932
and Chr17:55792683-55792789

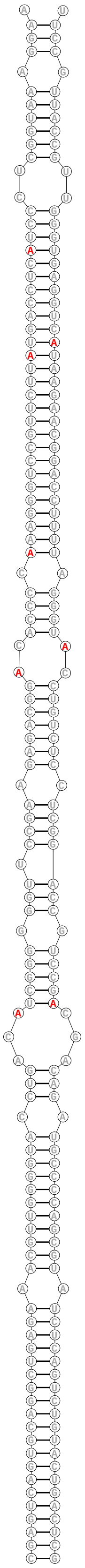

TMEM120B part D

Strands Chr17:55791825-55791880
and Chr17:55792880-55792934

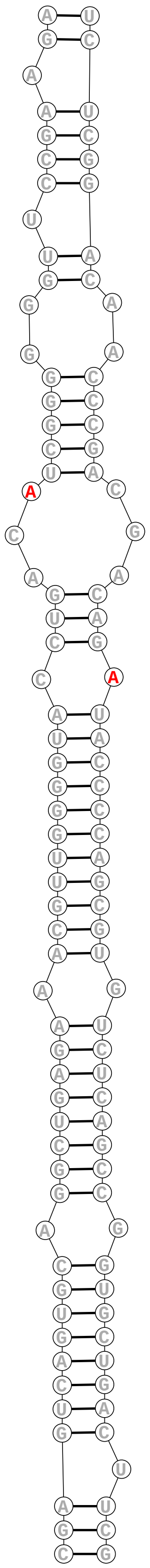

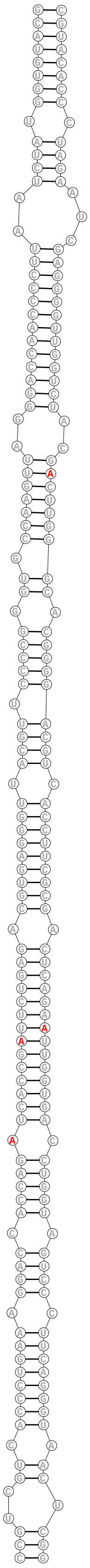

TMEM120B part E

Strands Chr17:55808754-55808857
and Chr17:55809458-55809559

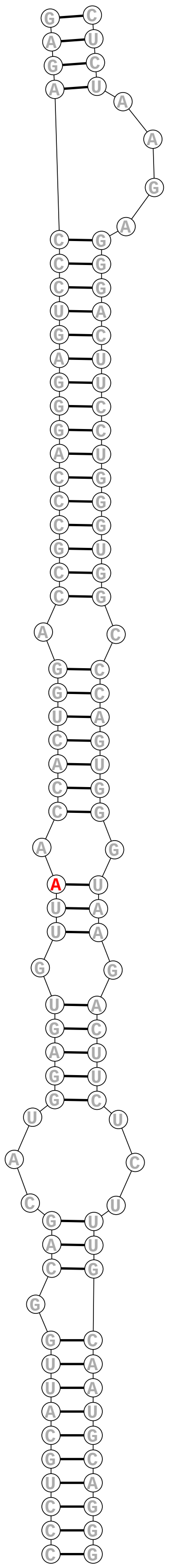

TMEM132A

Strands Chr29:37851256-37851310
and Chr29:37851631-37851688

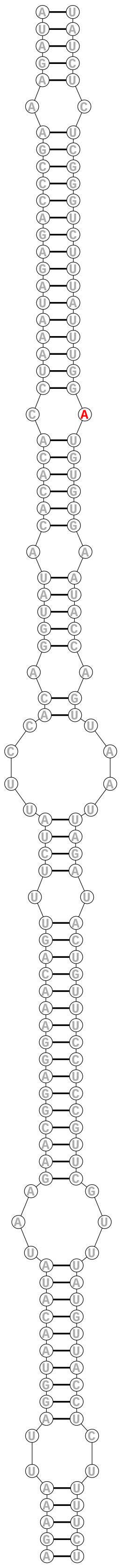

TMEM192

Strands Chr17:162817-162899
and Chr17:163937-164019

TMEM248

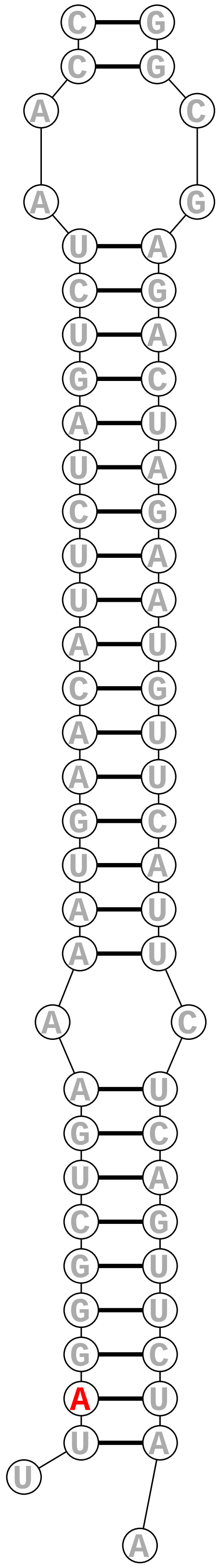

Strands Chr25:28569900-28569931
and Chr25:28570304-28570335

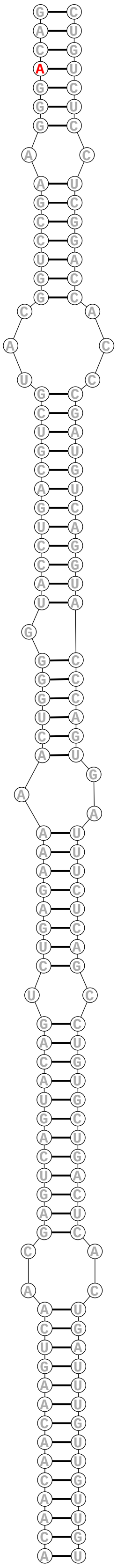

TMEM39A

Strands Chr1:64783047-64783121
and Chr1:64783769-64783843

TMEM39B part A

Strands Chr2:122197107-122197217
and Chr2:122197674-122197785

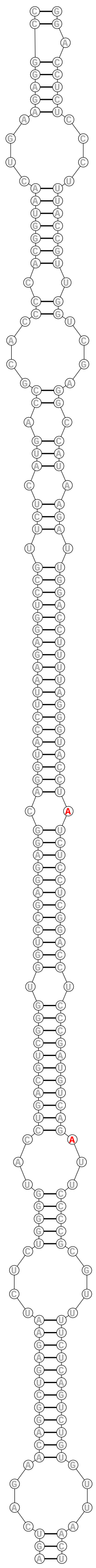

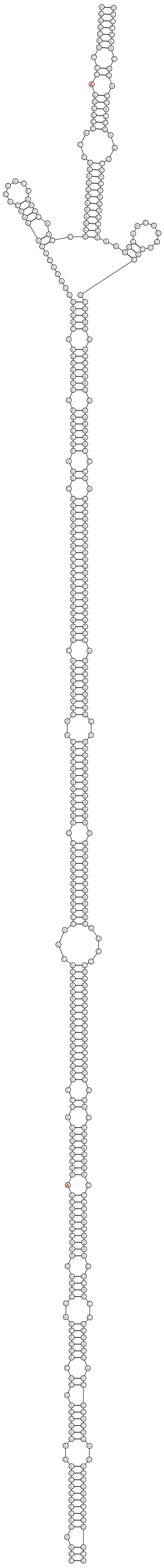

TMEM39B part B

Strands Chr2:122202685-122202912
and Chr2:122204048-122204263

TMEM63A

Strands Chr16:29640315-29640492
and Chr16:29640856-29641062

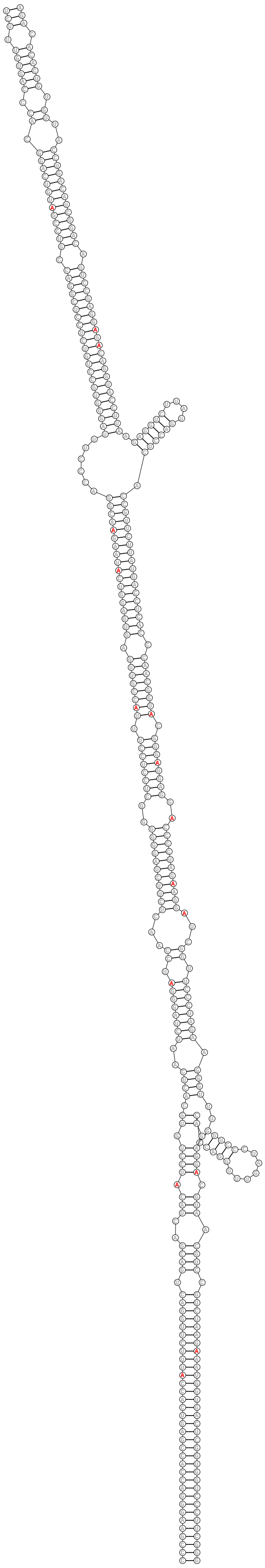

TMEM68

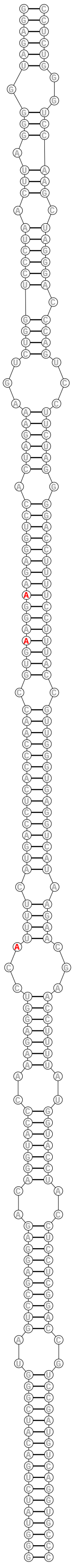

Strands Chr14:24717869-24717990
and Chr14:24718230-24718352

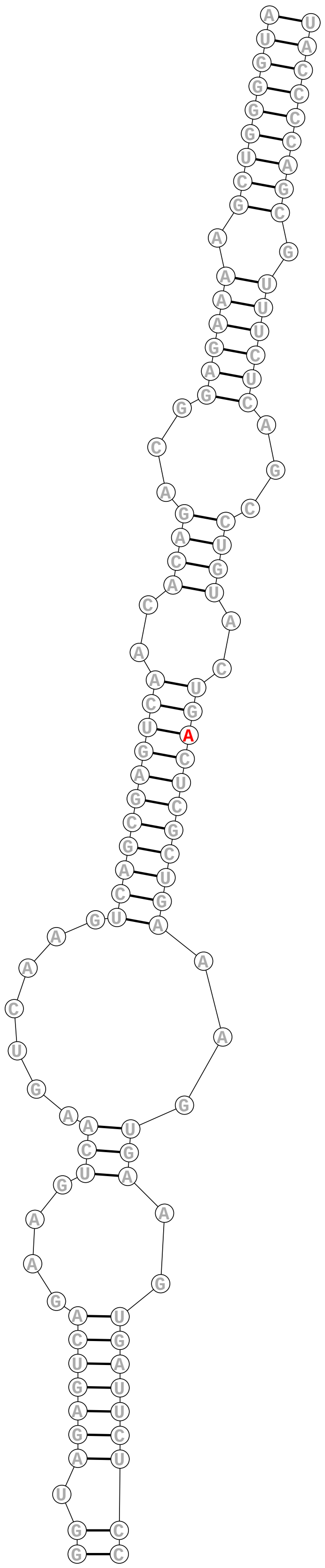

TMTC4 part A

Strands Chr12:81269908-81269967
and Chr12:81273443-81273495

TMTC4 part B

Strands Chr12:81268566-81268656
and Chr12:81273407-81273485

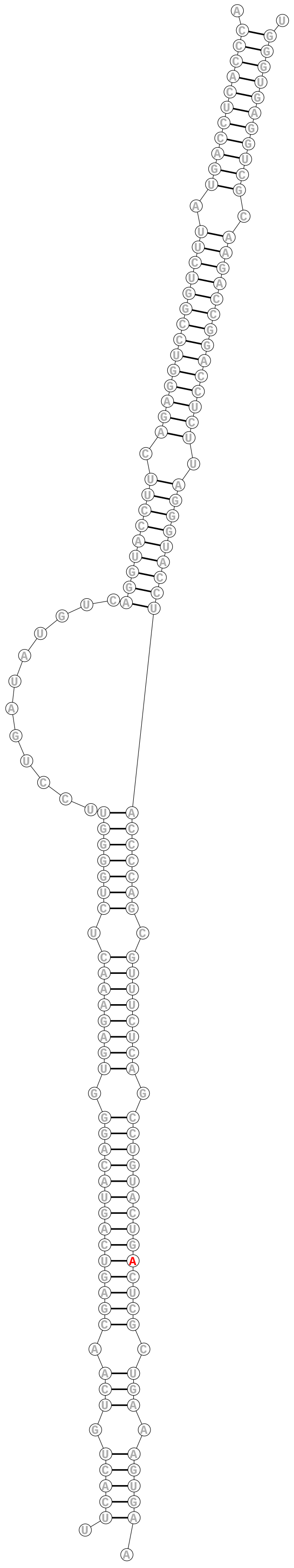

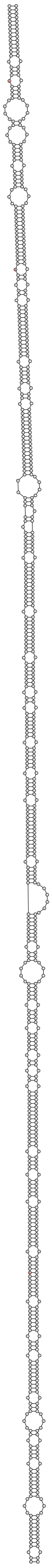

TNFSF10

Strands Chr1:95757337-95757687
and Chr1:95757823-95758181

TNPO2

Strands Chr7:13913665-13913750
and Chr7:13914358-13914434

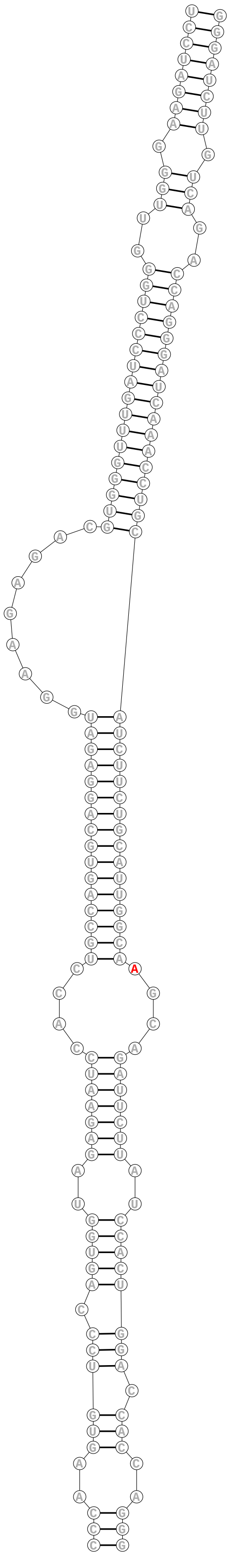

TOLLIP

Strands Chr29:51321681-51321982
and Chr29:51322598-51322899

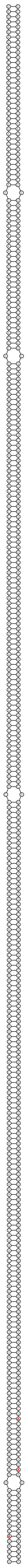

TOM1L1 part A

Strands Chr19:5285053-5285274
and Chr19:5286168-5286387

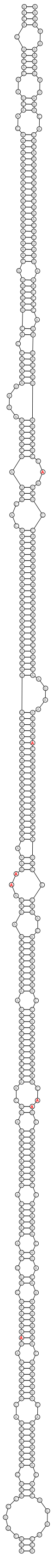

TOM1L1 part B

Strands Chr19:5285128-5285209
and Chr19:5286002-5286091

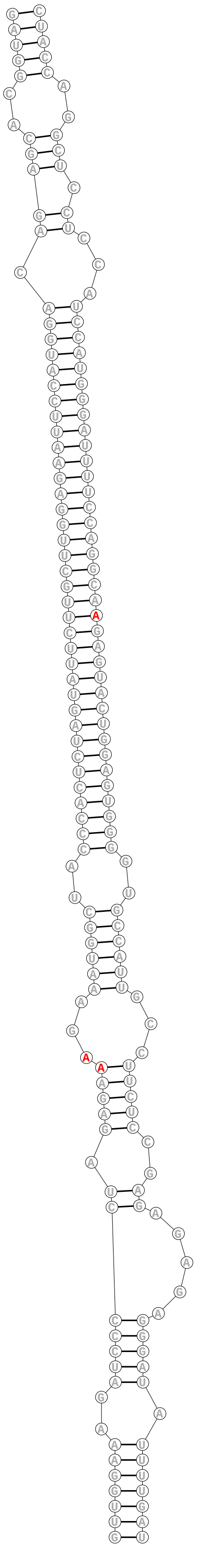

TP53RK

Strands Chr13:76084852-76084899
and Chr13:76085057-76085103

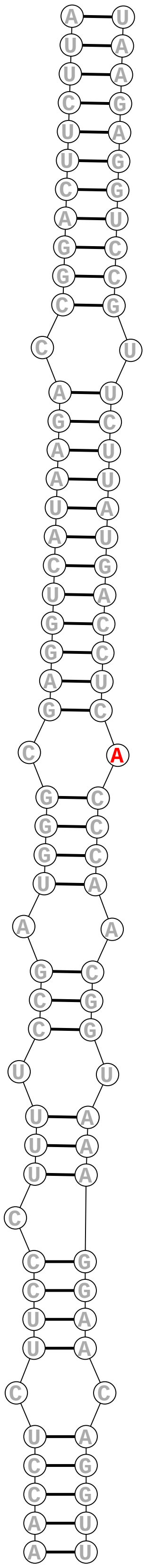

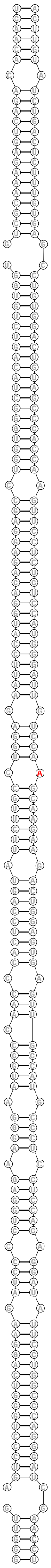

TPMT

Strands Chr23:39227814-39227952
and Chr23:39228681-39228818

TRAPPC13

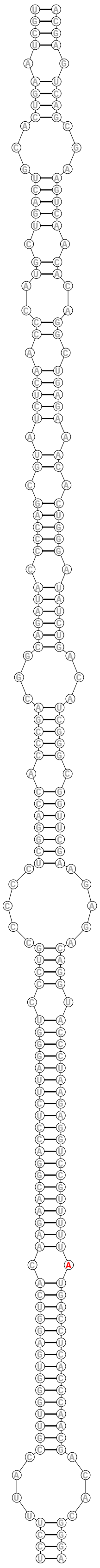

Strands Chr20:13857278-13857393
and Chr20:13857475-13857590

TRIM21

Strands Chr15:51479411-51479488
and Chr15:51480101-51480177

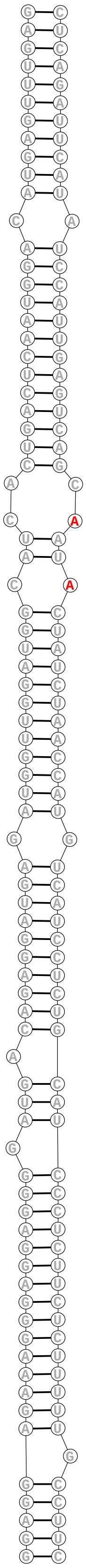

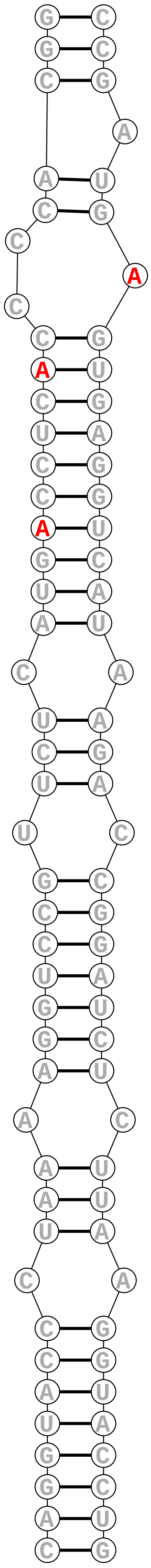

TRMT13 part A

Strands Chr3:43280243-43280284
and Chr3:43281107-43281148

TRMT13 part B

Strands Chr3:43279175-43279282
and Chr3:43281045-43281151

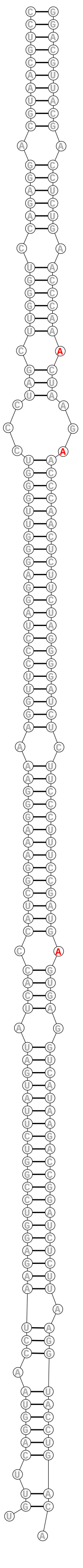

TRMT13 part C

Strands Chr3:43280045-43280088
and Chr3:43281143-43281188

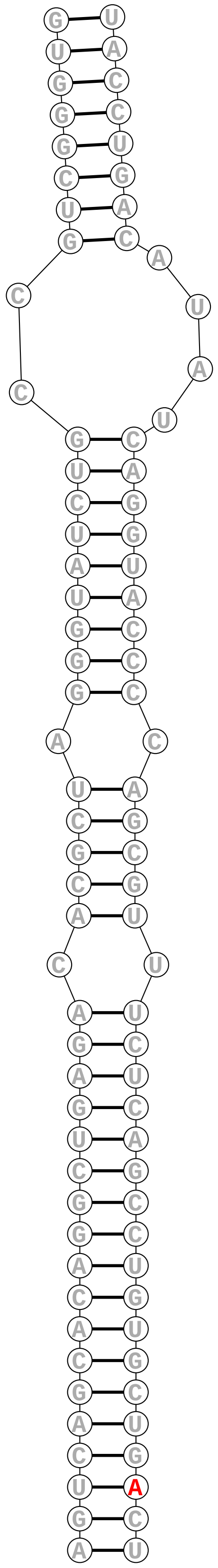

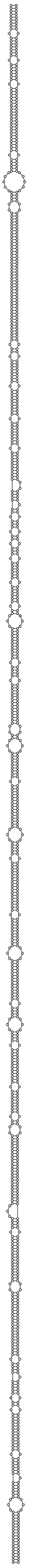

TRNAU1AP

Strands Chr2:125441267-125441742
and Chr2:125442060-125442537

TROVE2

Strands Chr16:12787250-12787348
and Chr16:12787438-12787534

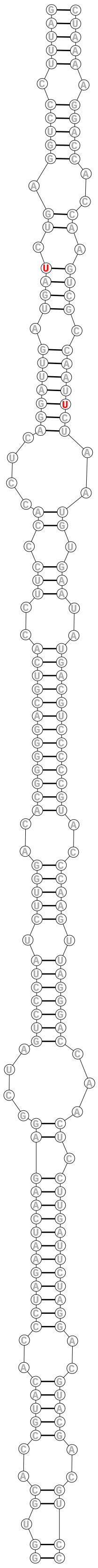

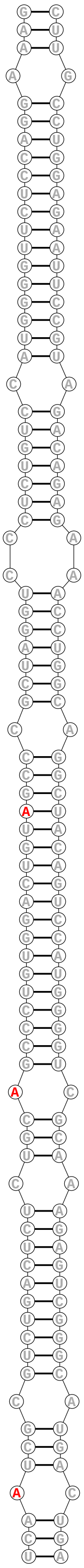

TRPM4

Strands Chr18:56118631-56118708
and Chr18:56118945-56119022

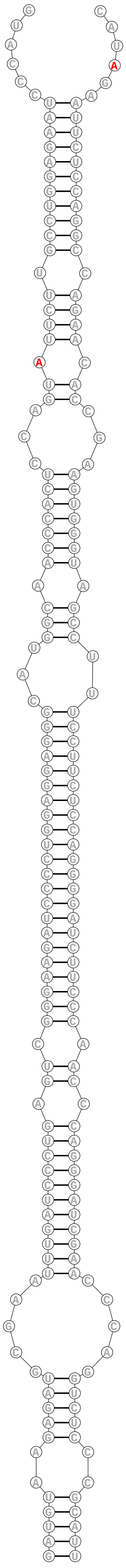

TSPAN15 part A

Strands Chr28:26009140-26009235
and Chr28:26009471-26009565

TSPAN15 part B

Strands Chr28:26009602-26009723
and Chr28:26011084-26011203

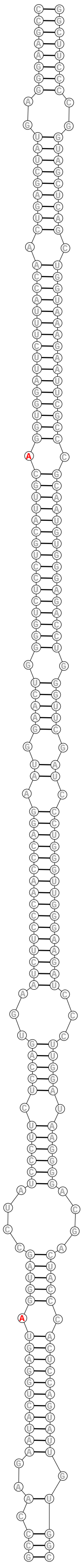

TSPAN15 part C

Strands Chr28:26009090-26009142
and Chr28:26009673-26009724

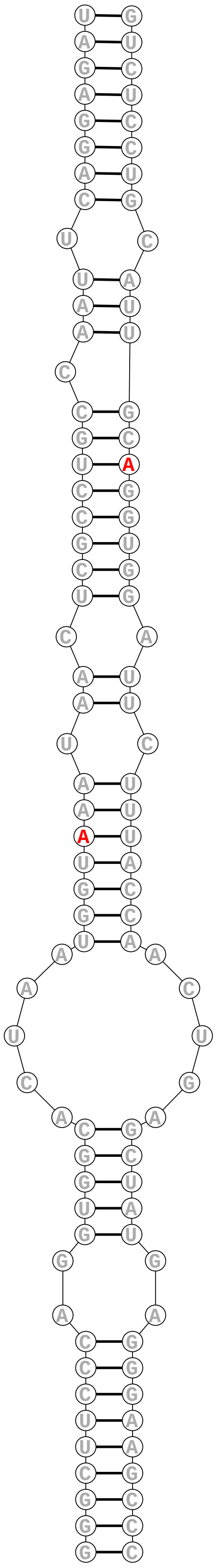

TSPO

Strands Chr5:114606456-114606541
and Chr5:114606655-114606739

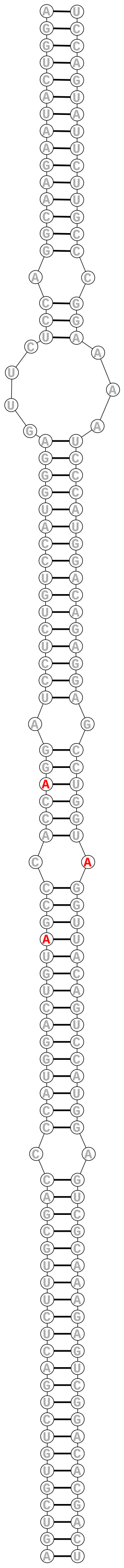

TTC7B

Strands Chr10:103222151-103222365
and Chr10:103222871-103223080

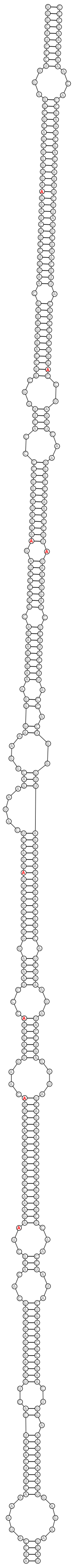

TTF1 part A

Strands Chr11:102511596-102511803
and Chr11:102513041-102513259

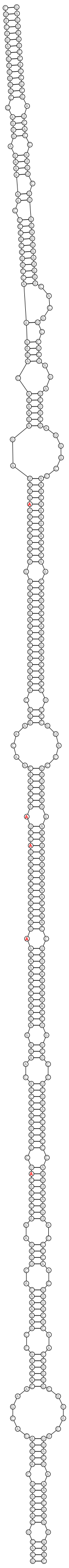

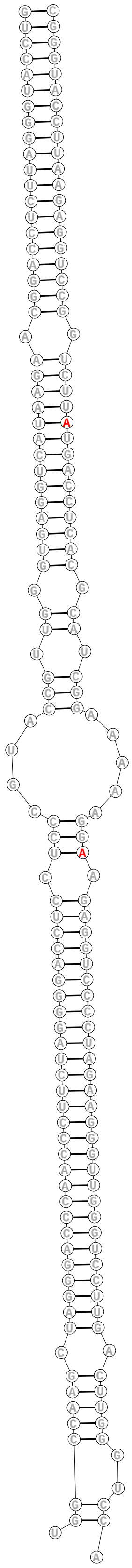

TTF1 part B

Strands Chr11:102513100-102513186
and Chr11:102513793-102513882

TTLL3

Strands Chr22:16937859-16937957
and Chr22:16938488-16938619

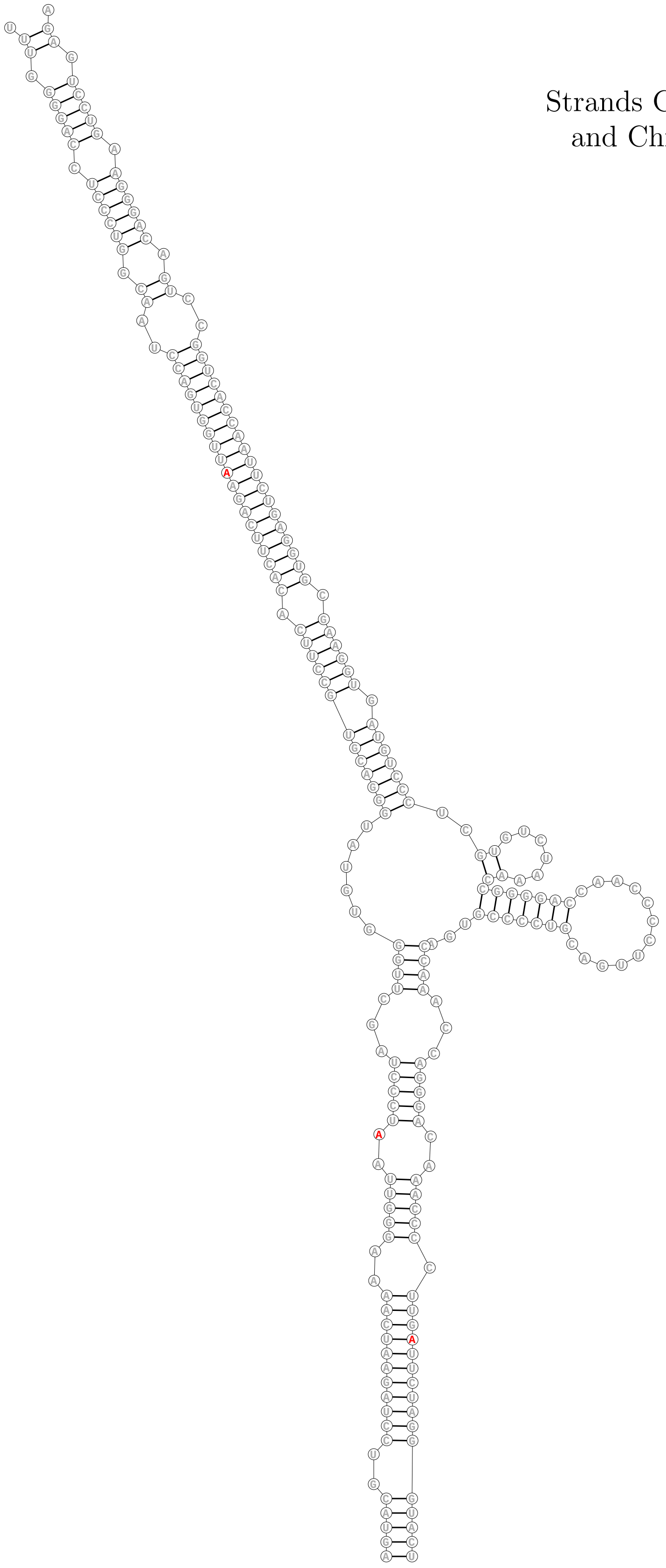

TUBGCP3

Strands Chr12:90203202-90203463
and Chr12:90204056-90204328

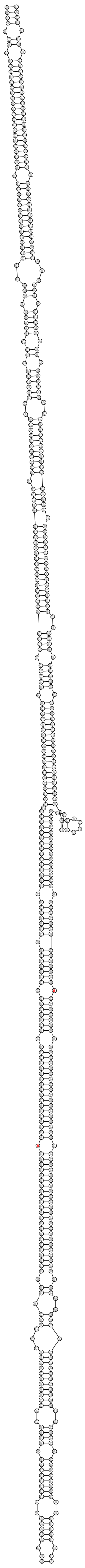

TULP2

Strands Chr18:55932422-55932639
and Chr18:55933456-55933632

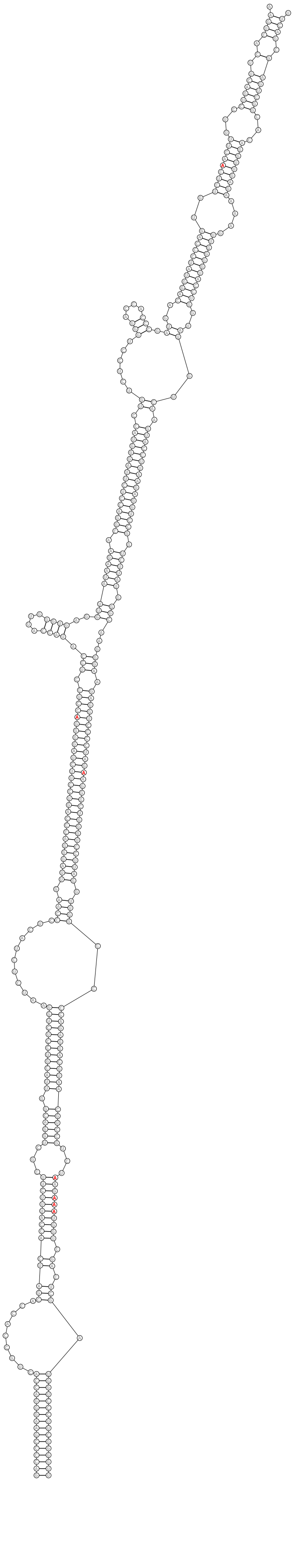

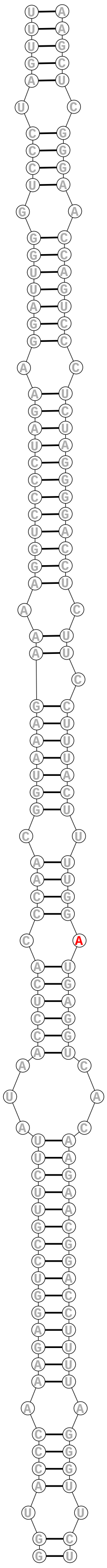

TXN

Strands Chr8:101488006-101488084
and Chr8:101488918-101488997

TXNL4B

Strands Chr18:39164244-39164333
and Chr18:39165482-39165569

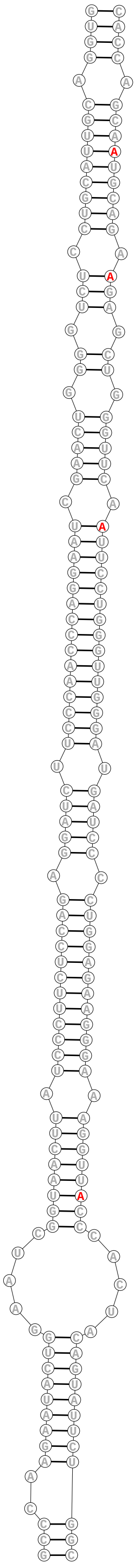

UBA5

Strands Chr1:137965434-137965502
and Chr1:137965544-137965599

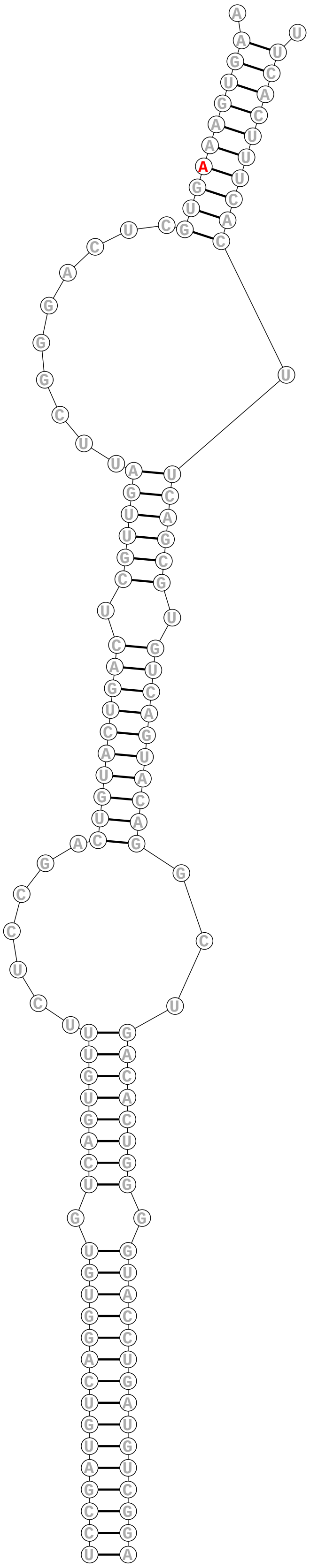

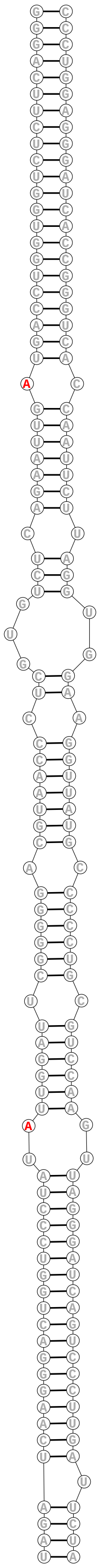

UBALD2

Strands Chr19:56073304-56073388
and Chr19:56073800-56073884

UCHL5

Strands Chr16:12815877-12815997
and Chr16:12816709-12816833

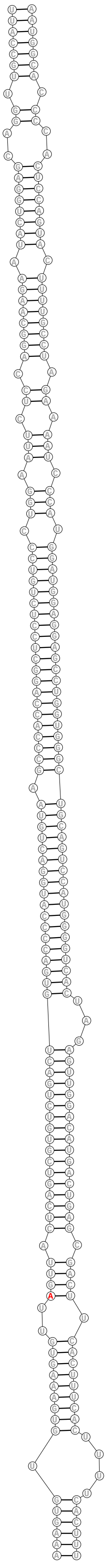

UFSP2 part A

Strands Chr27:14709201-14709264
and Chr27:14710673-14710736

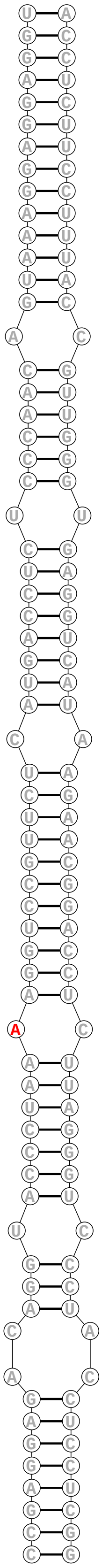

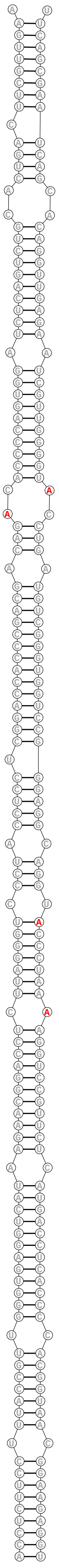

UFSP2 part B

Strands Chr27:14710673-14710790
and Chr27:14711531-14711646

UNC50 part A

Strands Chr11:3795531-3795730
and Chr11:3795850-3796051

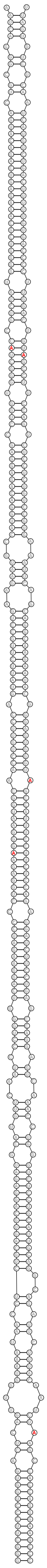

UNC50 part B

Strands Chr11:3795531-3795735
and Chr11:3797234-3797425

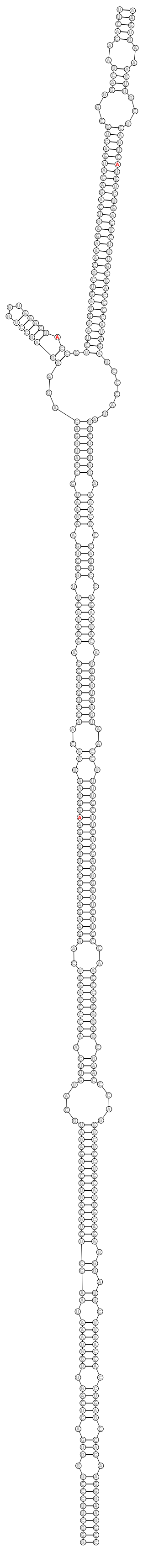

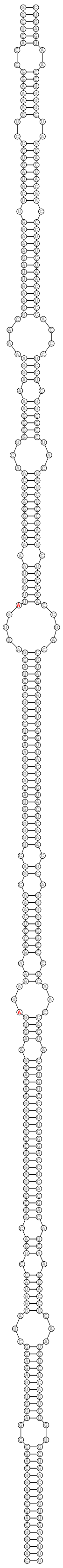

UNC93B1

Strands Chr29:46164622-46164822
and Chr29:46165022-46165222

URB2 part A

Strands Chr28:622733-622866
and Chr28:623871-624012

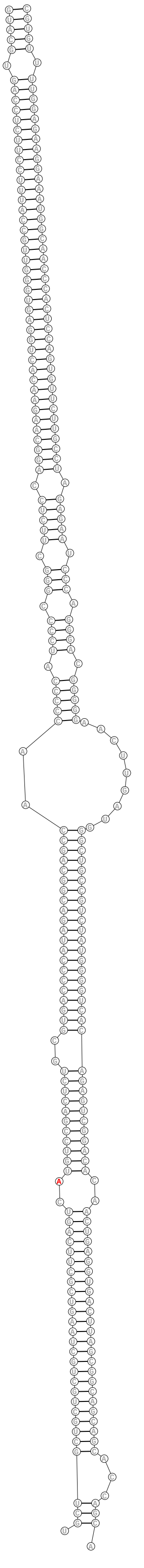

URB2 part B

Strands Chr28:622883-623009
and Chr28:623872-623996

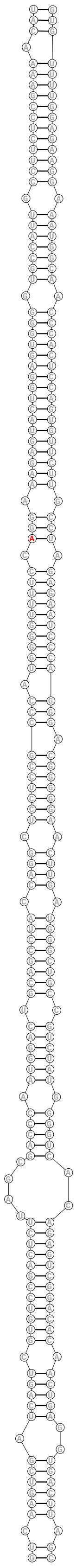

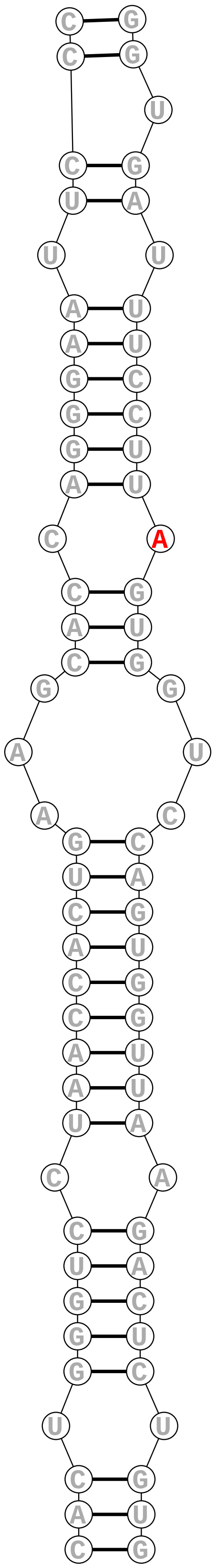

USP4

Strands Chr22:51366901-51366937
and Chr22:51367210-51367247

VAMP5 part A

Strands Chr11:49251198-49251405
and Chr11:49252768-49252960

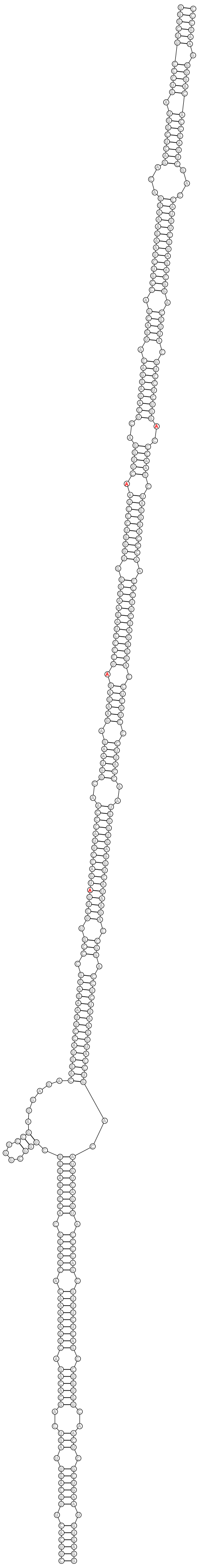

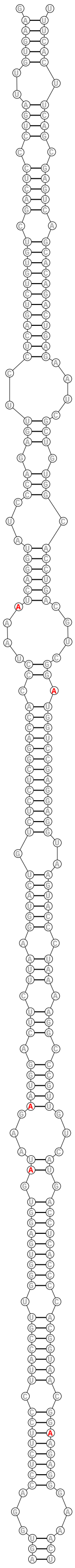

VAMP5 part B

Strands Chr11:49255037-49255164
and Chr11:49256082-49256209

VDR part A

Strands Chr5:32600739-32600870
and Chr5:32601458-32601589

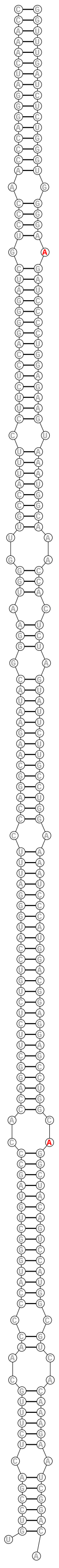

VDR part B

Strands Chr5:32600726-32600924
and Chr5:32603375-32603587

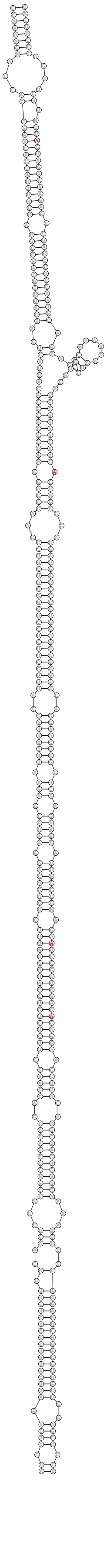

VPS11

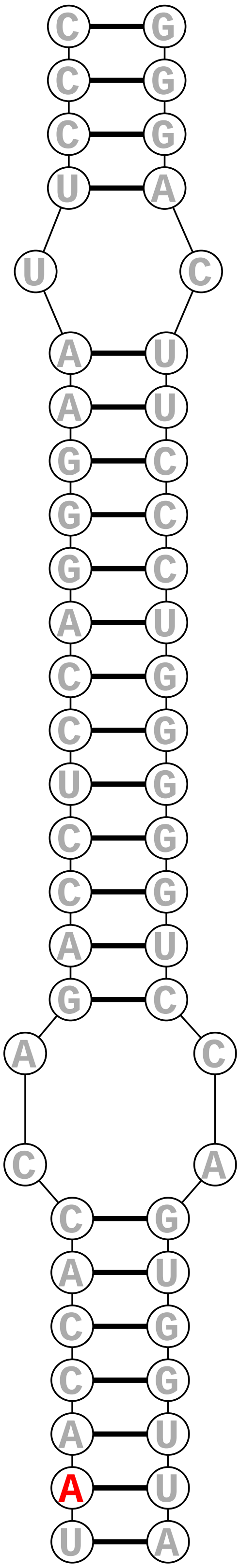

Strands Chr15:30187223-30187249
and Chr15:30187846-30187872

VTI1B

Strands Chr10:80089693-80089815
and Chr10:80090923-80091045

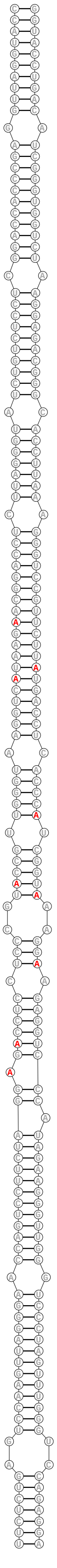

VWF part A

Strands Chr5:104724518-104724633
and Chr5:104724985-104725100

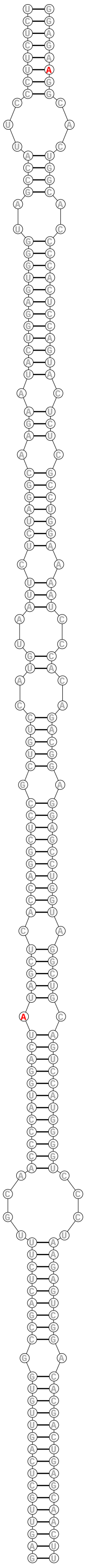

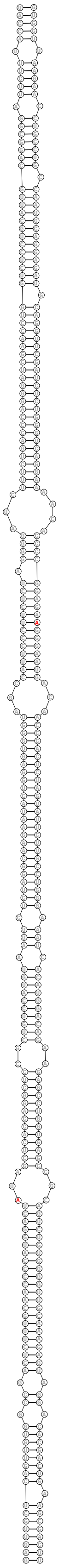

VWF part B

Strands Chr5:104724518-104724697
and Chr5:104726191-104726373

WDR59

Strands Chr18:2342521-2342560
and Chr18:2342939-2342979

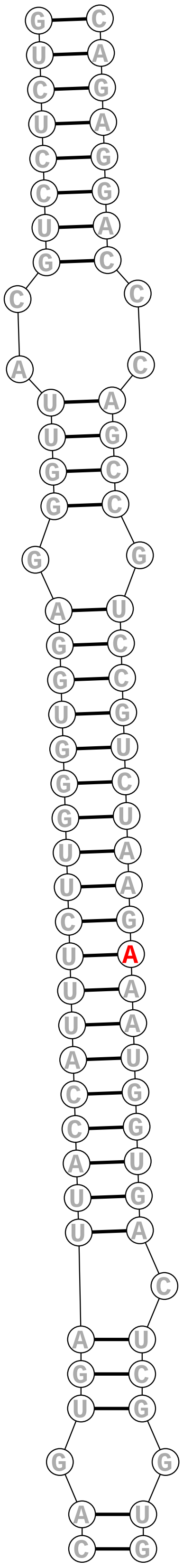

WDYHV1

Strands Chr14:18025524-18025614
and Chr14:18026705-18026797

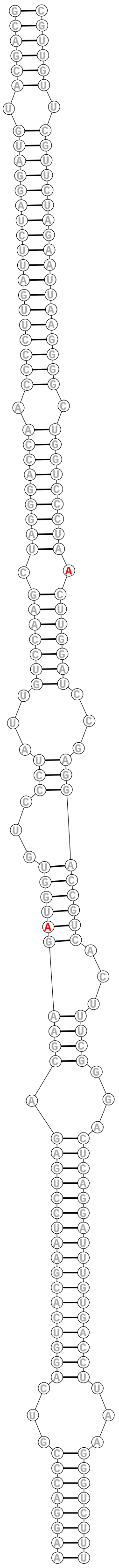

WIPF3

Strands Chr4:66860236-66860257
and Chr4:66861019-66861041

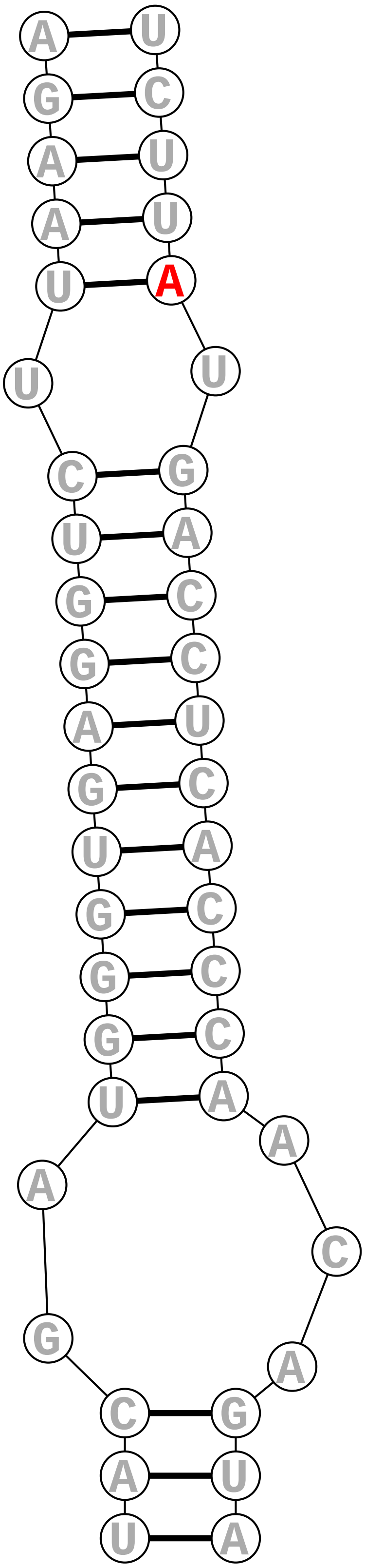

WISP2

Strands Chr13:73843530-73843551
and Chr13:73844924-73844945

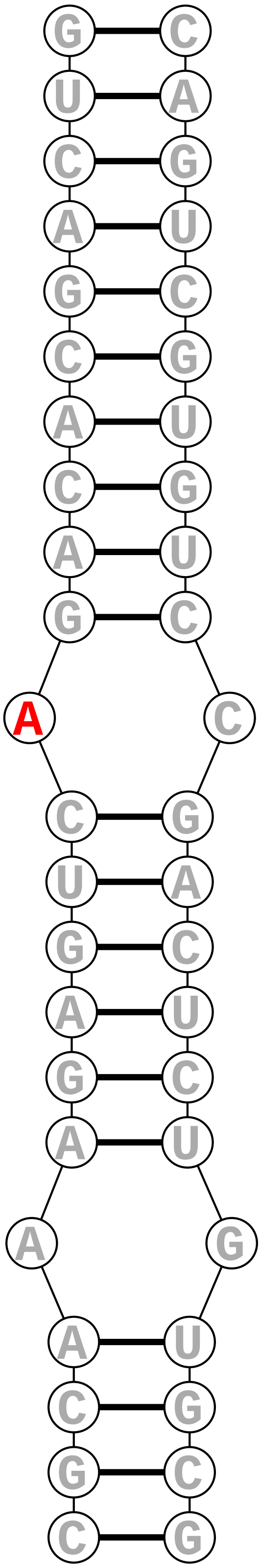

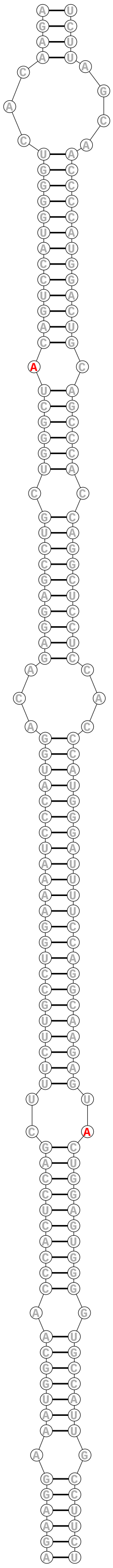

WTAP

Strands Chr9:97456221-97456311
and Chr9:97456988-97457079

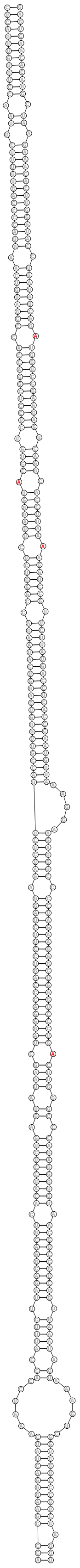

XDH part A

Strands Chr11:14203659-14203849
and Chr11:14205040-14205236

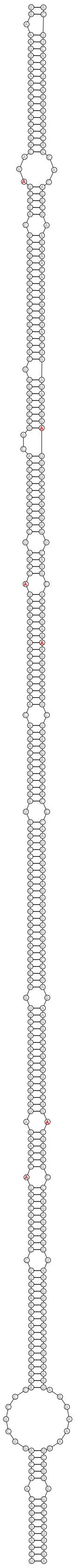

XDH part B

Strands Chr11:14203909-14204119
and Chr11:14205027-14205233

XPA

Strands Chr8:63359266-63359288
and Chr8:63359593-63359613

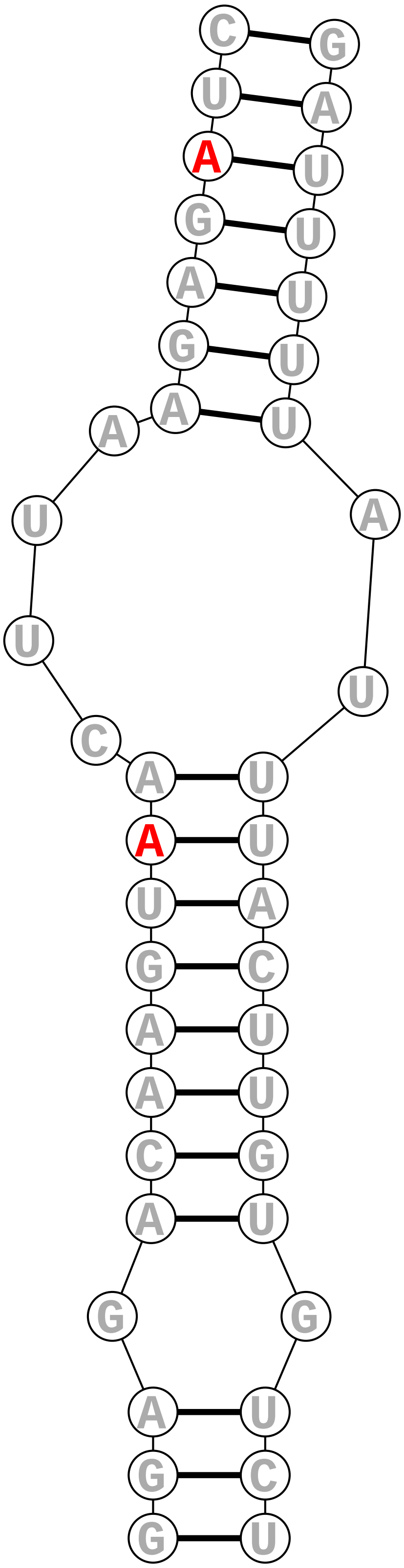

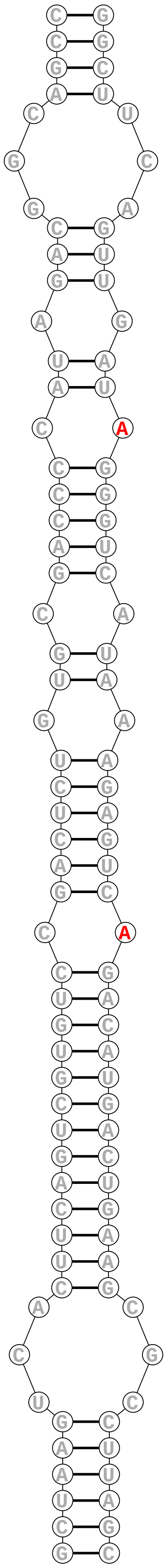

YY1

Strands Chr21:66824649-66824700
and Chr21:66825288-66825339

ZC3H13

Strands Chr12:16086958-16087132
and Chr12:16087296-16087471

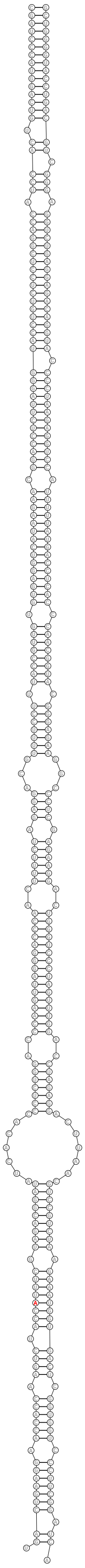

ZCCHC24

Strands Chr28:35192487-35192638
and Chr28:35192752-35192903

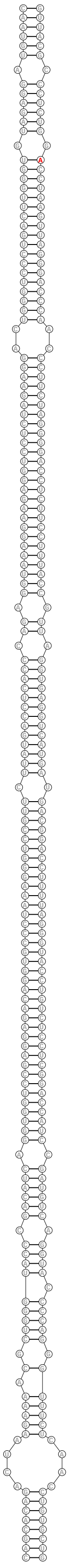

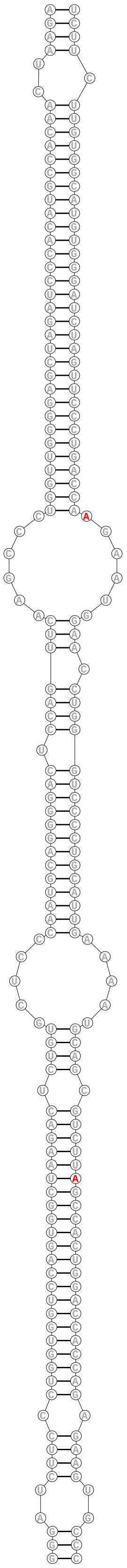

ZCCHC8

Strands Chr17:55206590-55206695
and Chr17:55207929-55208033

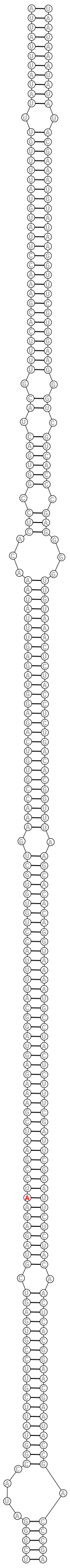

ZDHHC13

Strands Chr29:26028144-26028299
and Chr29:26028371-26028523

ZFAND1

Strands Chr14:83243318-83243390
and Chr14:83243893-83243965

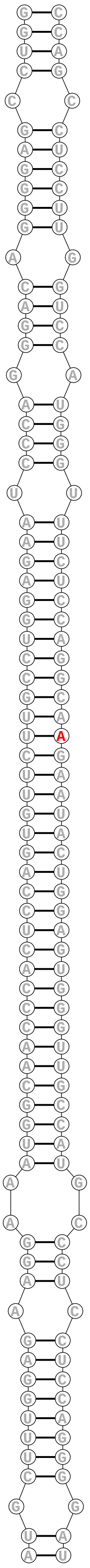

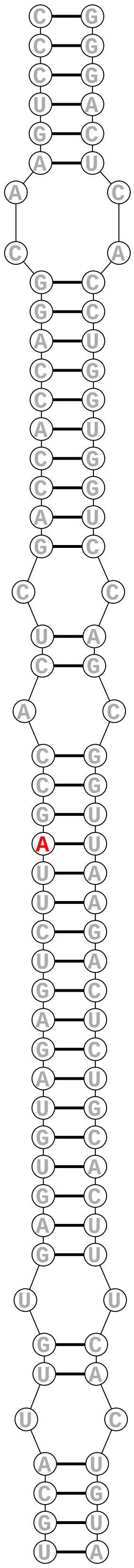

ZFP82

Strands Chr18:47293109-47293156
and Chr18:47293227-47293274

ZFYVE27

Strands Chr26:18763540-18763678
and Chr26:18763820-18763956

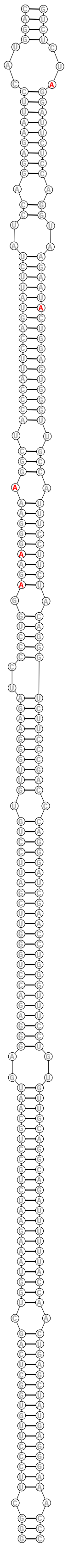

ZMYM5

Strands Chr12:36511513-36511575
and Chr12:36512959-36513031

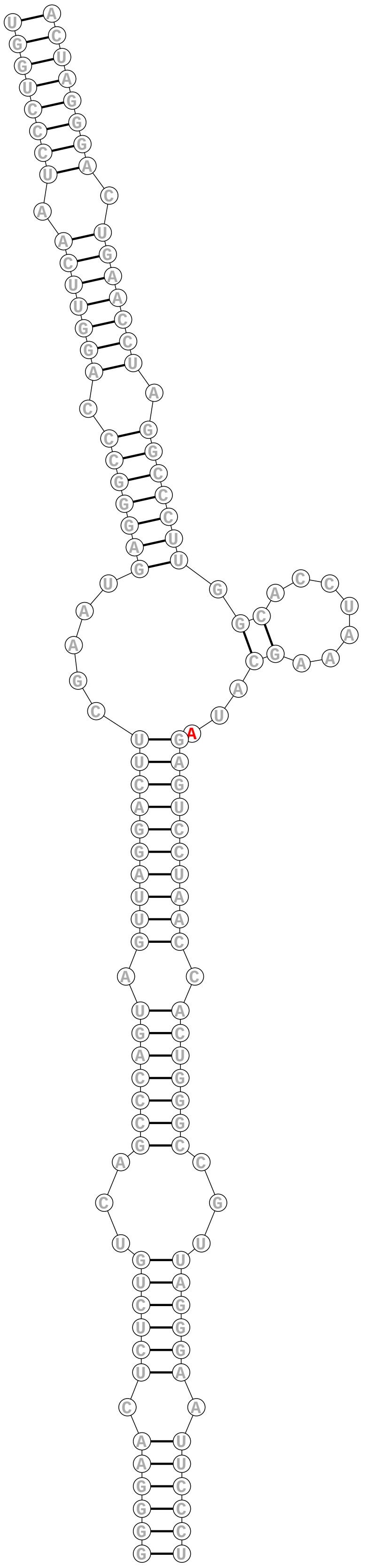

ZNF394 part A

Strands Chr25:37448626-37448812
and Chr25:37450513-37450697

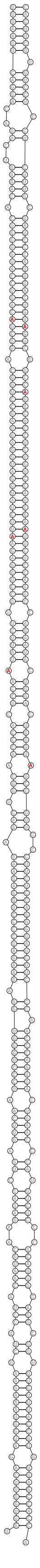

ZNF394 part B

Strands Chr25:37449290-37449410
and Chr25:37450530-37450650

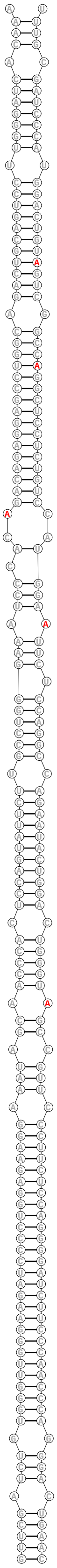

ZNF532

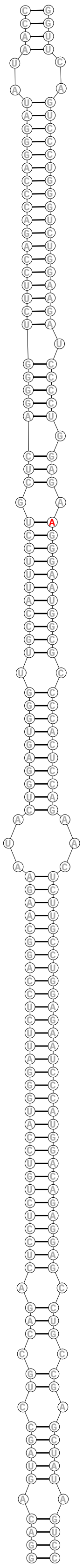

Strands Chr24:58499824-58499930
and Chr24:58500435-58500543

ZNF621 part A

Strands Chr22:13380872-13380989
and Chr22:13381483-13381576

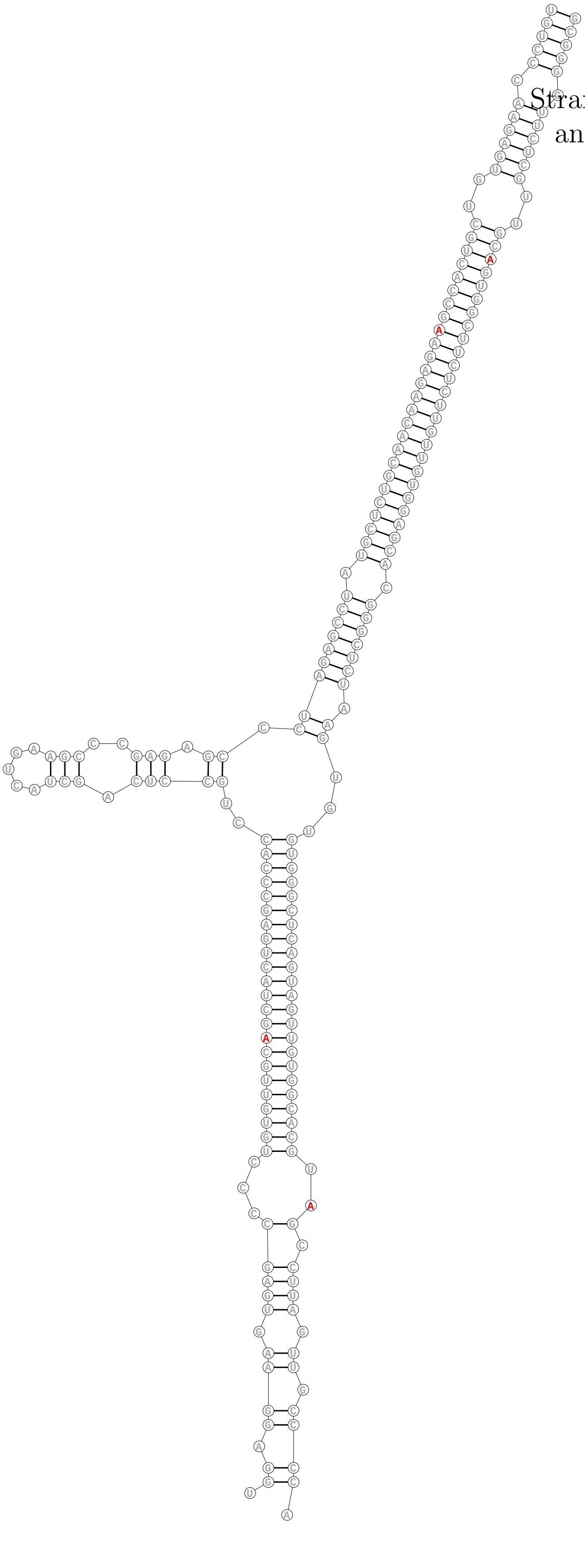

ZNF621 part B

Strands Chr22:13415716-13415788
and Chr22:13416214-13416288

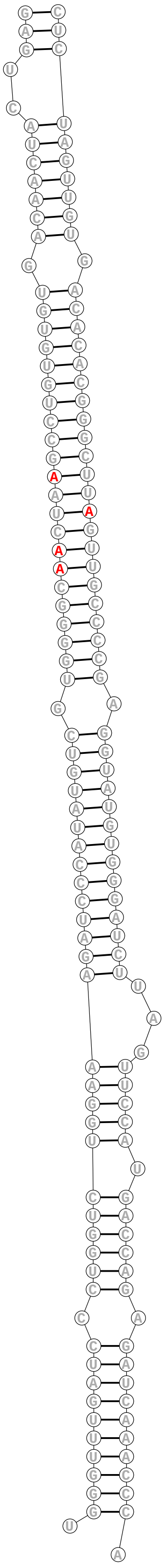

ZNF621 part C

Strands Chr22:13415855-13415922
and Chr22:13416635-13416694

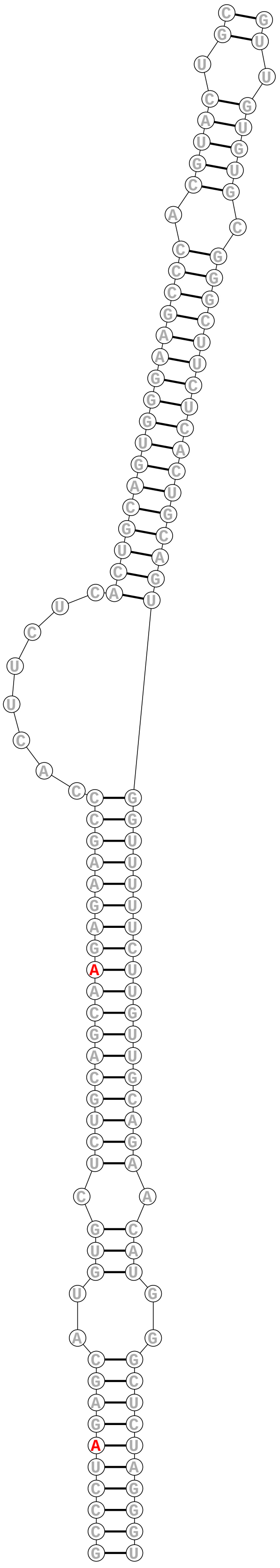

ZNF653

Strands Chr7:17074540-17074647
and Chr7:17083217-17083322

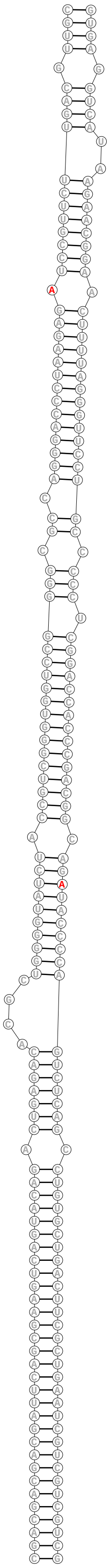

ZNF654

Strands Chr1:35717045-35717106
and Chr1:35717693-35717754

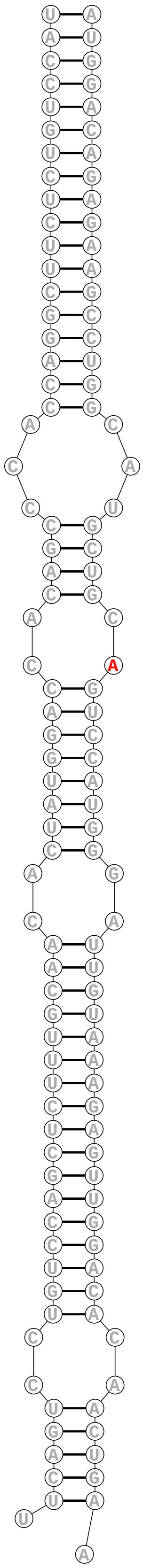

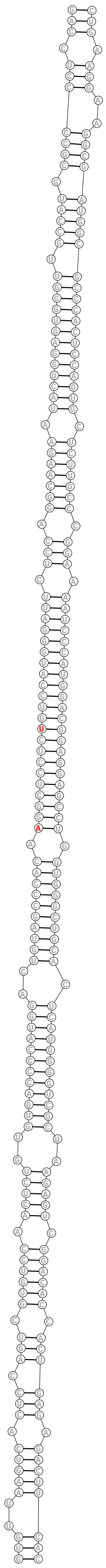

ZNF839 part A

Strands Chr21:68792812-68792935
and Chr21:68794245-68794364

ZNF839 part B

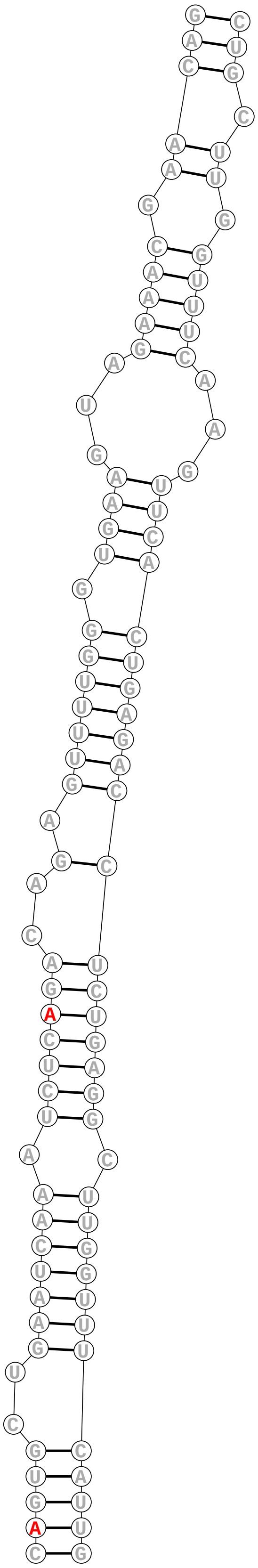

Strands Chr21:68791618-68791669
and Chr21:68792495-68792541

ZNHIT3

Strands Chr19:13151828-13151899
and Chr19:13153327-13153397

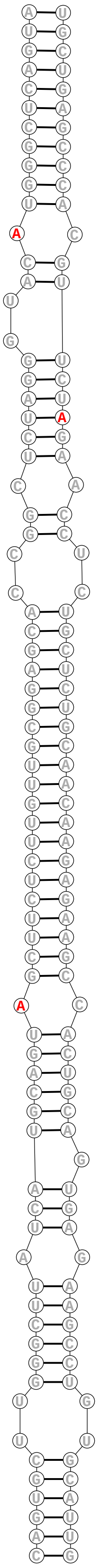

Supplement: Supplemental Material [file supp_066902.118_Supplemental_Figure_S3.pdf]
