# Supplementary figures and images for: Widespread cis-regulation of RNA editing in a large mammal
Source: RNA. 2019 Mar;25(3):319–35. doi: 10.1261/rna.066902.118 (PMC6380278; doi:10.1261/rna.066902.118)

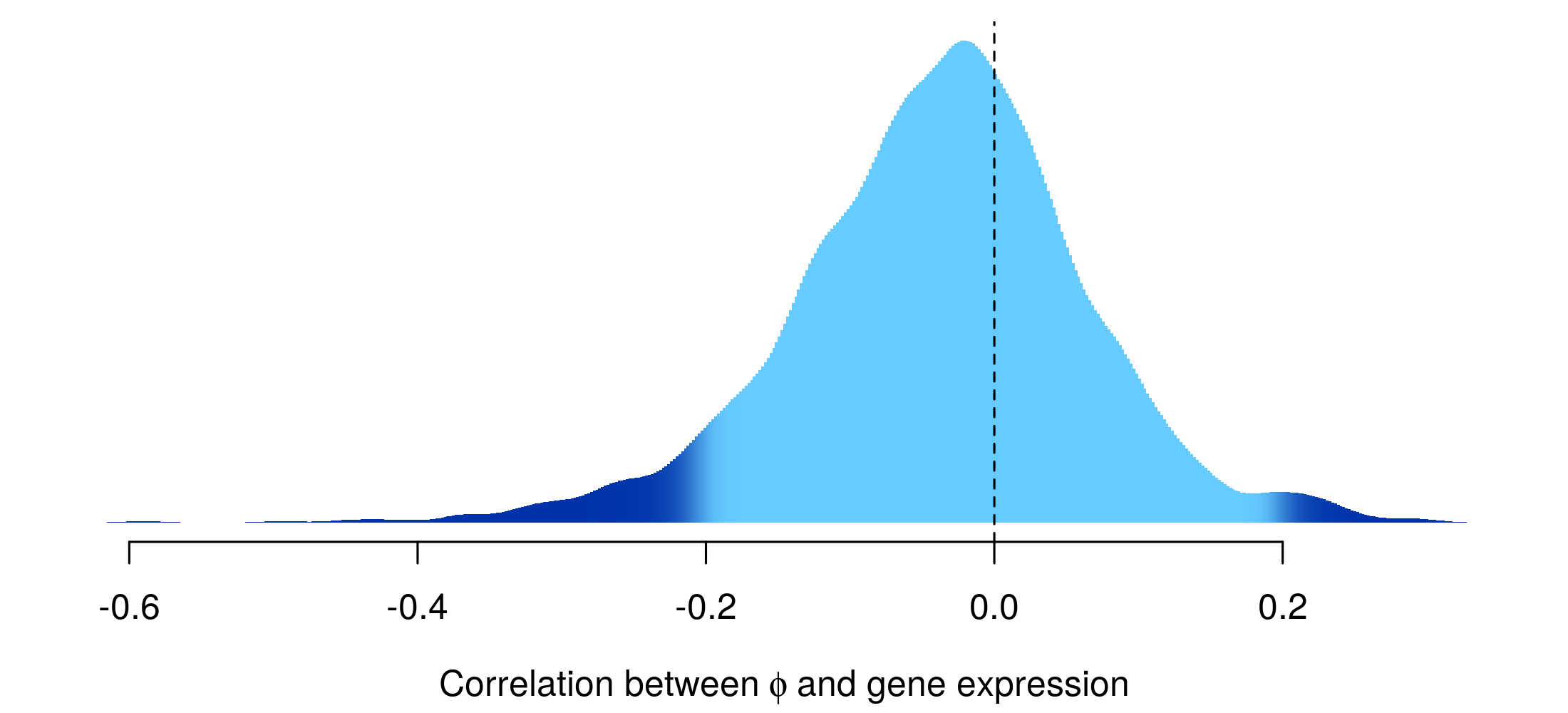

Supplement: Supplemental Material [file supp_066902.118_Supplemental_Figure_S4.tif]

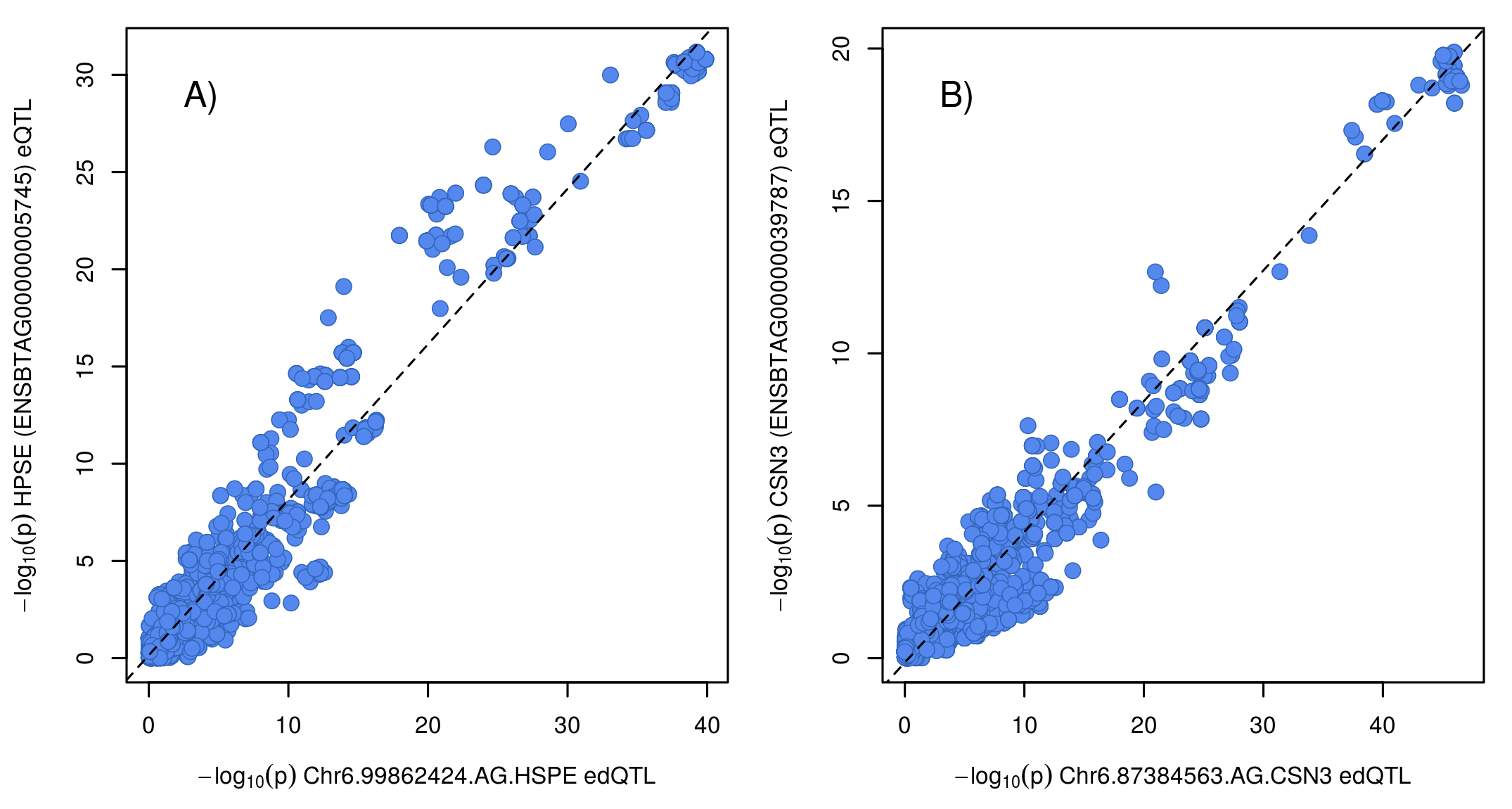

Supplement: Supplemental Material [file supp_066902.118_Supplemental_Figure_S6.tif]

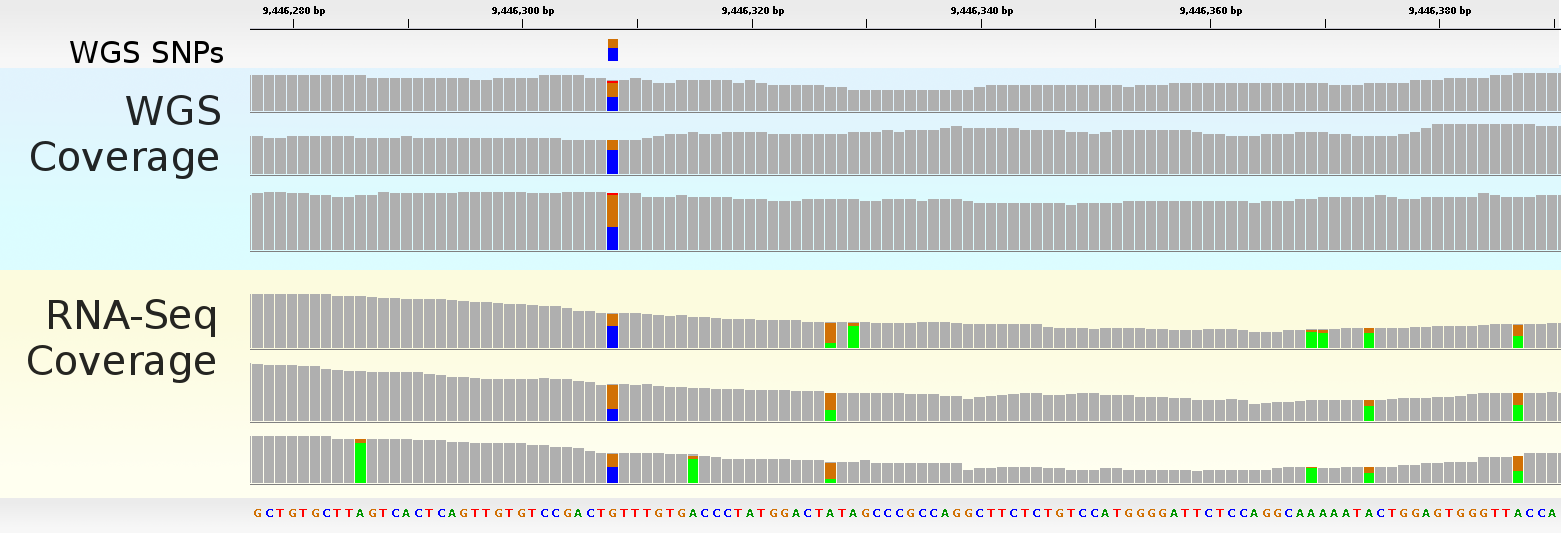

Supplement: Supplemental Material [file supp_066902.118_Supplemental_Figure_S7.tif]
