# Supplementary material for: Widespread cis-regulation of RNA editing in a large mammal
Source: RNA. 2019 Mar;25(3):319–35. doi: 10.1261/rna.066902.118 (PMC6380278; doi:10.1261/rna.066902.118)
Supplement: Supplemental Material [file supp_066902.118_Supplemental_Figure_S5.pdf]

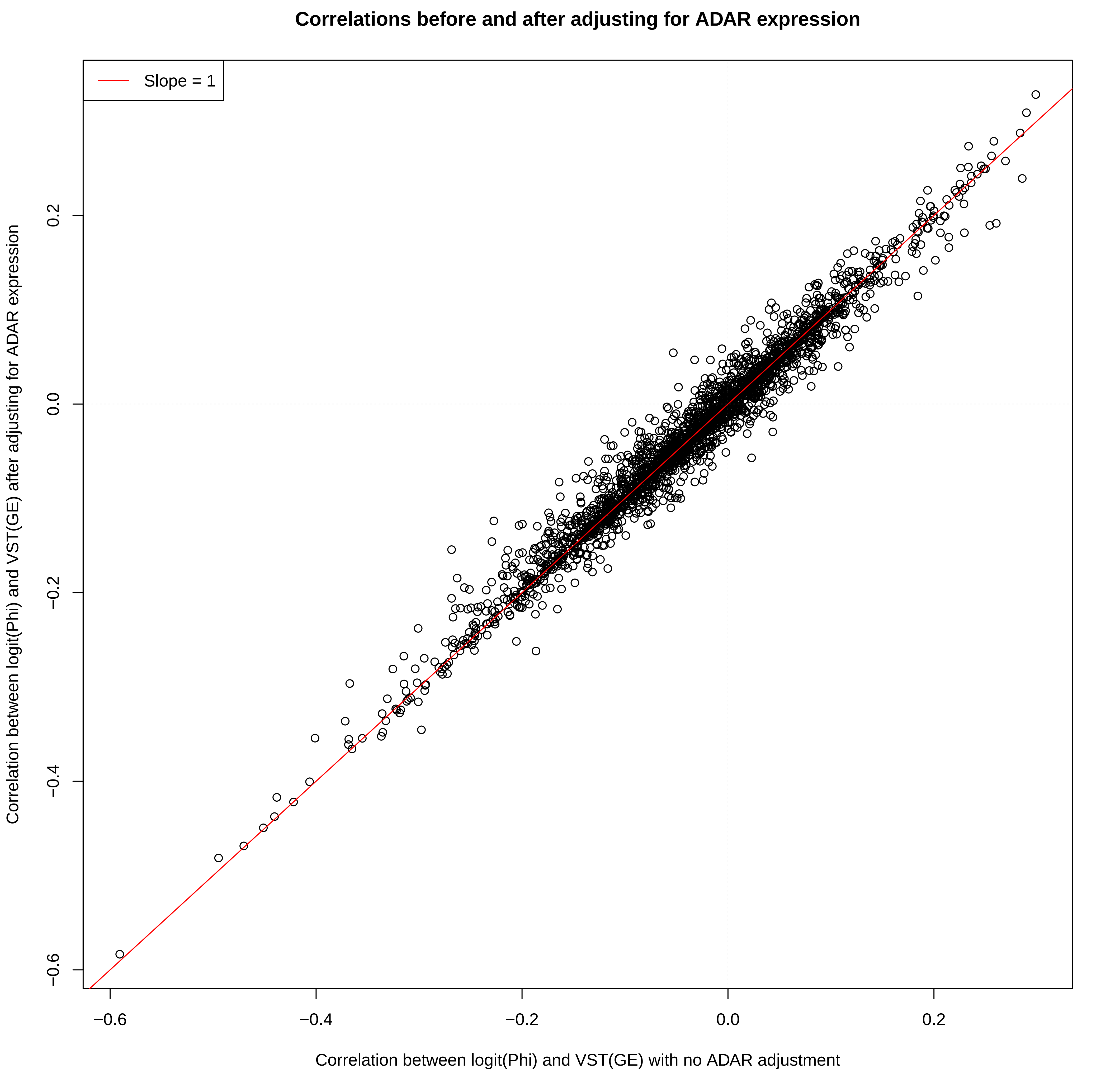
Correlations before and after adjusting for ADAR expression
Slope = 1
Correlation between logit(Phi) and VST(GE) after adjusting for ADAR expression
Correlation between logit(Phi) and VST(GE) with no ADAR adjustment
−0.6
−0.4
−0.2
0.0
0.2

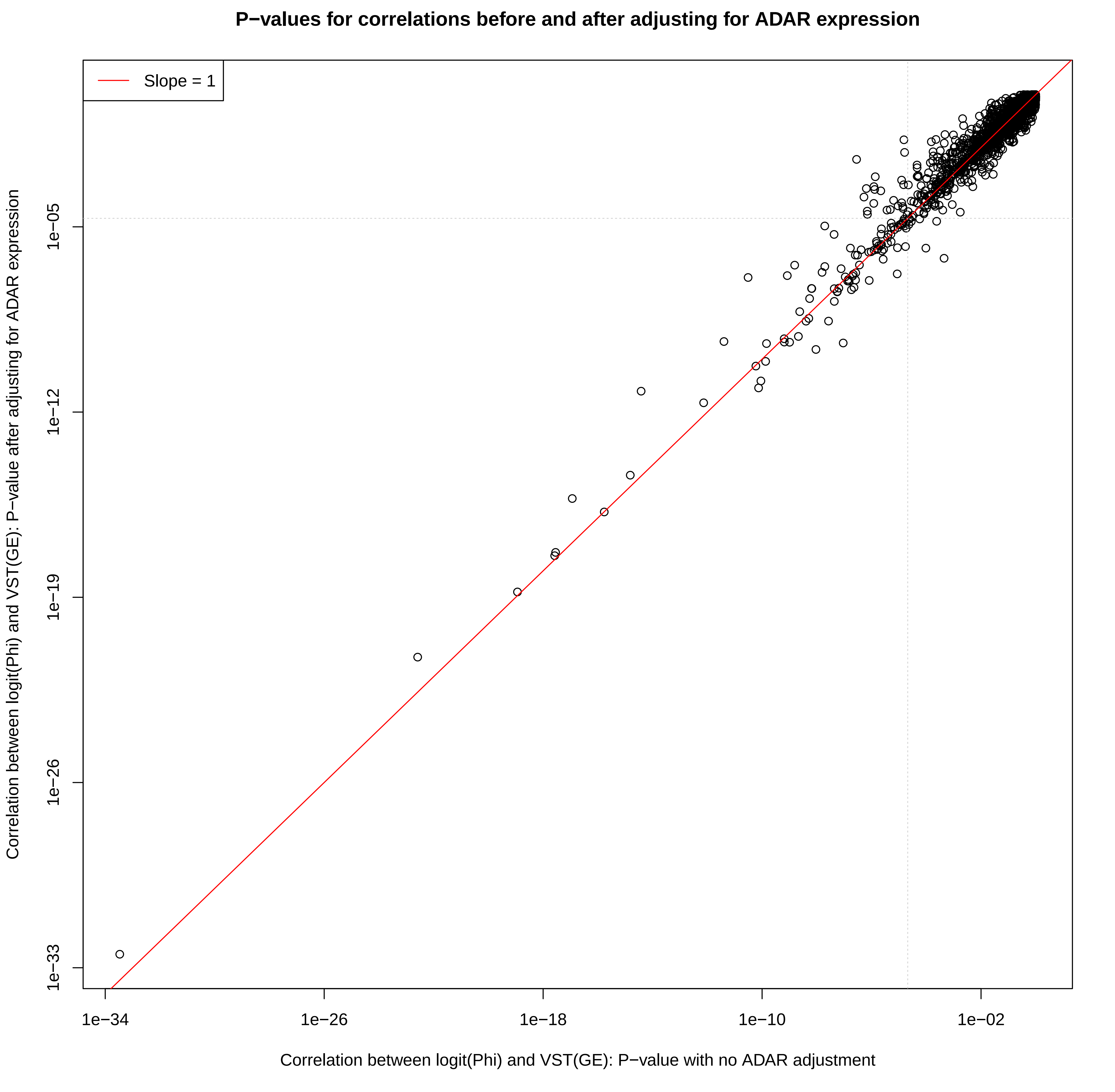
P−values for correlations before and after adjusting for ADAR expression
Slope = 1
Correlation between logit(Phi) and VST(GE): P−value after adjusting for ADAR expression
1e−05
1e−12
1e−19
1e−26
1e−33
1e−34
1e−26
1e−18
1e−10
1e−02
Correlation between logit(Phi) and VST(GE): P−value with no ADAR adjustment
