# Supplementary material for: Widespread cis-regulation of RNA editing in a large mammal
Source: RNA. 2019 Mar;25(3):319–35. doi: 10.1261/rna.066902.118 (PMC6380278; doi:10.1261/rna.066902.118)
Supplement: Supplemental Material [file supp_066902.118_Supplemental_Legends.docx]

# Supporting Information

## Figure S1

Traces for edit sites targeted for verification by Sanger sequencing. Traces are shown for the five of eight sites that yielded usable traces (see Materials and Methods).

## Figure S2

Dot plots of genome sequences centred on RNA editing sites. Sequences are plotted against their complement, with dots indicating that at least 11 of the 15 surrounding nucleotides are complementary, yielding diagonal black lines where long strands of complementarity are present, indicating potential double-stranded regions. Red dotted lines indicate the positions of RNA editing sites, showing that these tend to cluster within the double-stranded regions.

## Figure S3

Structures predicted using the ‘bifold’ program from the RNAstructure (Reuter and Mathews, 2010) software package, for double-stranded regions predicted from the dot plots shown in Figure S2. Edited sites are indicated in red.

## Figure S4

The distribution of Pearson correlation statistics calculated between editing rates (logit-transformed Φ values) and the expression of the genes to which they map (VST-transformed; see Materials and Methods). Dark blue indicates correlations which are significant after Bonferroni correction (*p*<2*.*62×10*^−^*^5^).

## Figure S5

Pearson correlation statistics (*r*) calculated between editing rates (logit-transformed Φ values) and the expression of the genes to which they map (VST-transformed), before and after adjusting for the level of *ADAR* expression. The second plot shows the p-values for the null hypothesis *r*=0. On both plots, the red line passes through the intercept with a unitary slope.

## Figure S6

Two examples of co-located, co-segregating eQTL and edQTL. Each point represents the *−*log_10_ p-values for one variant for an edQTL (x-axis) and eQTL (y-axis). A) The edQTL for the Chr6.99862424.AG.HSPE site, against the *HSPE* eQTL, with correlation *r*=0.921. B) The edQTL for the Chr6.87384563.AG.CSN3 site, against the *CSN3* gene, with correlation *r*=0.849.

## Figure S7

An example comparison between WGS and RNAseq for three animals, illustrating the difference between SNPs and RNA editing. The region shown is part of intron 1 of the *LPO* (lactoperoxidase) gene. The top row shows the single SNP called in this region from a large WGS study. The section with the blue background shows the WGS coverage mapped for three animals, where grey represents the reference base (indicated at the bottom of the figure), and with blue and brown representing cytosine and guanine respectively. The section with the yellow background shows the RNAseq coverage for the same three animals (green = adenine). Edit sites appear in the RNAseq coverage as bars of mixed green and brown (A-to-G(I) edits), while SNPs appear at the same location in both the WGS and RNAseq sequences.

## Table S1

Summary data for edited sites. Tab one (“Edited Sites”) contains the chromosome and base position of each site on the bovine UMD 3.1 reference genome, along with the gene in which the site is located by VGNC symbol and Ensembl ID, plus the reference and edited base, complemented when the strand is negative. Tab two (“VEP Results”) contains the outputs from the Variant Effect Predictor for each edited site. The reference and edited base are not complemented in this tab. Only A-to-I sites are included.

## Table S2

Details of the 134 edit sites exhibiting genome-wide significant *cis*-edQTL. Gene symbols and Ensembl identifications are provided for genes containing the edit sites. Also included are the minimum p-values for each edQTL, as well as the strength of the *cis*-eQTL for the appropriate gene. The Spearman correlation between the association (*χ*^2^) statistics for the edQTL and eQTL are also included, in the second tab.

## Table S3

Correlations between all 134 *cis*-edQTL and milk production QTL. Edit sites are named by position (UMD 3.1 reference), reference and edited bases, and gene symbol. Tab one contains Pearson correlations, and tab two contains Spearman correlations. On both tabs, correlations greater than 0.707 ($\sqrt{0.5}$) are highlighted in bold, those greater than 0.5 are italicised, and those less than zero are in grey.

## Table S4

Primer sequences used for validation of eight RNA editing sites. Forward and reverse primer sequences are given, along with the expected length of the PCR product.
